# *C*. *elegans* Demonstrates Distinct Behaviors within a Fixed and Uniform Electric Field

**DOI:** 10.1371/journal.pone.0151320

**Published:** 2016-03-21

**Authors:** Steven D. Chrisman, Christopher B. Waite, Alison G. Scoville, Lucinda Carnell

**Affiliations:** Department of Biological Sciences, Central Washington University, Ellensburg, Washington, United States of America; Brown University/Harvard, UNITED STATES

## Abstract

*C*. *elegans* will orient and travel in a straight uninterrupted path directly towards the negative pole of a DC electric field. We have sought to understand the strategy worms use to navigate to the negative pole in a uniform electric field that is fixed in both direction and magnitude. We examined this behavior by quantifying three aspects of electrotaxis behavior in response to different applied field strengths: the mean approach trajectory angles of the animals’ tracks, turning behavior (pirouettes) and average population speeds. We determined that *C*. *elegans* align directly to the negative pole of an electric field at sub-preferred field strength and alter approach trajectories at higher field strengths to maintain taxis within a preferred range we have calculated to be ~ 5V/cm. We sought to identify the sensory neurons responsible for the animals’ tracking to a preferred field strength. *eat-4* mutant animals defective in glutamatergic signaling of the amphid sensory neurons are severely electrotaxis defective and *ceh-36* mutant animals, which are defective in the terminal differentiation of two types of sensory neurons, AWC and ASE, are partially defective in electrotaxis. To further elucidate the role of the AWC neurons, we examined the role of each of the pair of AWC neurons (AWC^OFF^ and AWC^ON^), which are functionally asymmetric and express different genes. *nsy-5*/*inx-19* mutant animals, which express both neurons as AWC^OFF^, are severely impaired in electrotaxis behavior while *nsy-1* mutants, which express both neurons as AWC^ON^, are able to differentiate field strengths required for navigation to a specific field strength within an electric field. We also tested a strain with targeted genetic ablation of AWC neurons and found that these animals showed only slight disruption of directionality and turning behavior. These results suggest a role for AWC neurons in which complete loss of function is less disruptive than loss of functional asymmetry in electrotaxis behavior within a uniform fixed field.

## Introduction

An underlying question in understanding behavior is how a sensory stimulus is integrated into the neural signals and pathways needed to elicit a motor response. *C*. *elegans* is widely used to study the genetic basis for behavior [1–4] due to its simple, but well described nervous system. Despite this simplicity, *C*. *elegans* has the ability to respond with a complex set of behaviors to external stimuli such as chemical odorants, temperature, touch and electric fields [5–8].

Electrotaxis is a directed movement of an organism in response to electric field stimuli and is a behavior that is conserved across many species. Electroreception has been widely observed in aquatic animals such as elasmobranchs, non-teleost bony fishes, and dolphins [9–11]. While not as prevalent, it is also found in terrestrial organisms such as amphibians [12], monotremes [13], dictyostelium [14], cockroaches [15], and bees [16]. Electroreception is used for navigation, the search for food (prey), and for predator detection [17]. The roundworm, *C*. *elegans*, also has the ability to sense and navigate to the negative pole in a direct current (DC) electric field [7, 18]. *C*. *elegans* move by propagating in a sinusoidal dorsal ventral contractile wave. Animals employ three basic crawling modes: a forward run, a reversal, and a turn to navigate to attractive environmental gradients. In the absence of sensory stimuli, animals utilize a reorientation behavior of random trial and error turns [19–22]. The animals also employ a more methodical approach to stimuli with a shallow turn in which the animal alters trajectory with a small angle maneuver while maintaining the sinusoidal wave. We were interested in the behavioral strategies and sensory pathway needed for this animal to integrate electric field stimuli into a motor response in a fixed uniform electric field under different field strengths. Previous studies examining electrotaxis behavior [7, 18] utilized a rotating field, which measures the worm’s ability to respond to a field varying in both direction and strength requiring reorientation and reversal maneuvers. Our studies utilize a uniform field fixed in direction to measure the ability of the worm to sense field strength and alter its approach trajectory by small shallow anterior head movements. Using an automated tracking system [23], we measured the animals’ directionality, speed and turning behavior to determine the neural basis for the widening of animal trajectories at higher field strengths in an approximate uniform electric field.

*C*. *elegans* sense and respond to external stimuli such as chemical and temperature gradients with left and right pairs of neurons located in sensory organs in the anterior (amphid) and posterior (phasmid) of the animal [24]. The primary sensory organ is a collection of amphid neurons in the anterior region of the animal near the pharyngeal bulb with axons that associate with the nerve ring. The winged amphid sensory neuron, AWC, has been demonstrated to serve an important sensory function in chemotaxis, thermotaxis and local search behaviors in foraging [21, 25–31]. AWC neurons are a left-right bilateral pair of asymmetric neurons; in a stochastic manner, one neuron, AWC^ON^, expresses the G protein-coupled receptor, STR-2, while the other neuron, AWC^OFF^, does not [32]. This asymmetry along with presumably additional undetermined asymmetry, has been shown to play a functional role in odor specificity and discrimination between odorants [33–35]. We have found that mutant animals that are defective in expression of genes involved in late differentiation and/or functional asymmetry of the AWC neuron display defects in electrotaxis. These data implicate a role for the AWC neurons in detecting and responding to a fixed and uniform DC electric field.

## Material and Methods

Strains were cultivated using standard procedures [1]. The following strains were provided by the *Caenorhabditis* Genetics Center (Minneapolis, MN), which is funded by a NIH-National Center Grant for Research Support: MT6308 *eat-4*(*ky5*), CX5893 *kyIs140* [*str-2*::*gfp +* co-injection marker *lin-15*(+)] I; *ceh-36(ky646*) X, CX4998 *kyIs140* I; *nsy-1*(*ky397*) II, CX6161 *nsy-5*/*inx-19*(*ky634*) I, CX6827 *eat-4*(*ky5*) III; *kyEx844* [“AWC::*eat- 4*”] P_*odr-3*_::*eat-4*, + co-injection marker *elt-2*::*gfp*], and AWC:: caspase, PY7502, *oyIs85* [*ceh-36*p::TU#813 + *ceh-36*p::TU#814 + *srtx-1*p::GFP + co-injection marker *unc-122*p::dsRed]. The ASJ::caspase strain *daf-2*(*e1368*)III; *jxEx102*(pQZ37[*trx-1*::ICE] + co-injection marker *ofm-1*::gfp) was a gift from Dr. Joy Alcedo (Wayne State University, Detroit, MI); the *jxEx102* extrachromosomal array was crossed into a wild type background for testing. The ASH::caspase strain, KP7442, *npr-1*(*ky13*);*nuEx1684*[*sra-6*::*ced-3*;*sra-6*::;mCherry; + co-injection marker vha-6::mcherry) was a gift from Dr. Joshua Kaplan (Massachusetts General Hospital/Harvard University, Boston, MA); the *nuEx1684* extrachromosomal array was crossed into a wild type background for testing.

### Generation of a Uniform Electric Field

**Fig 1:**
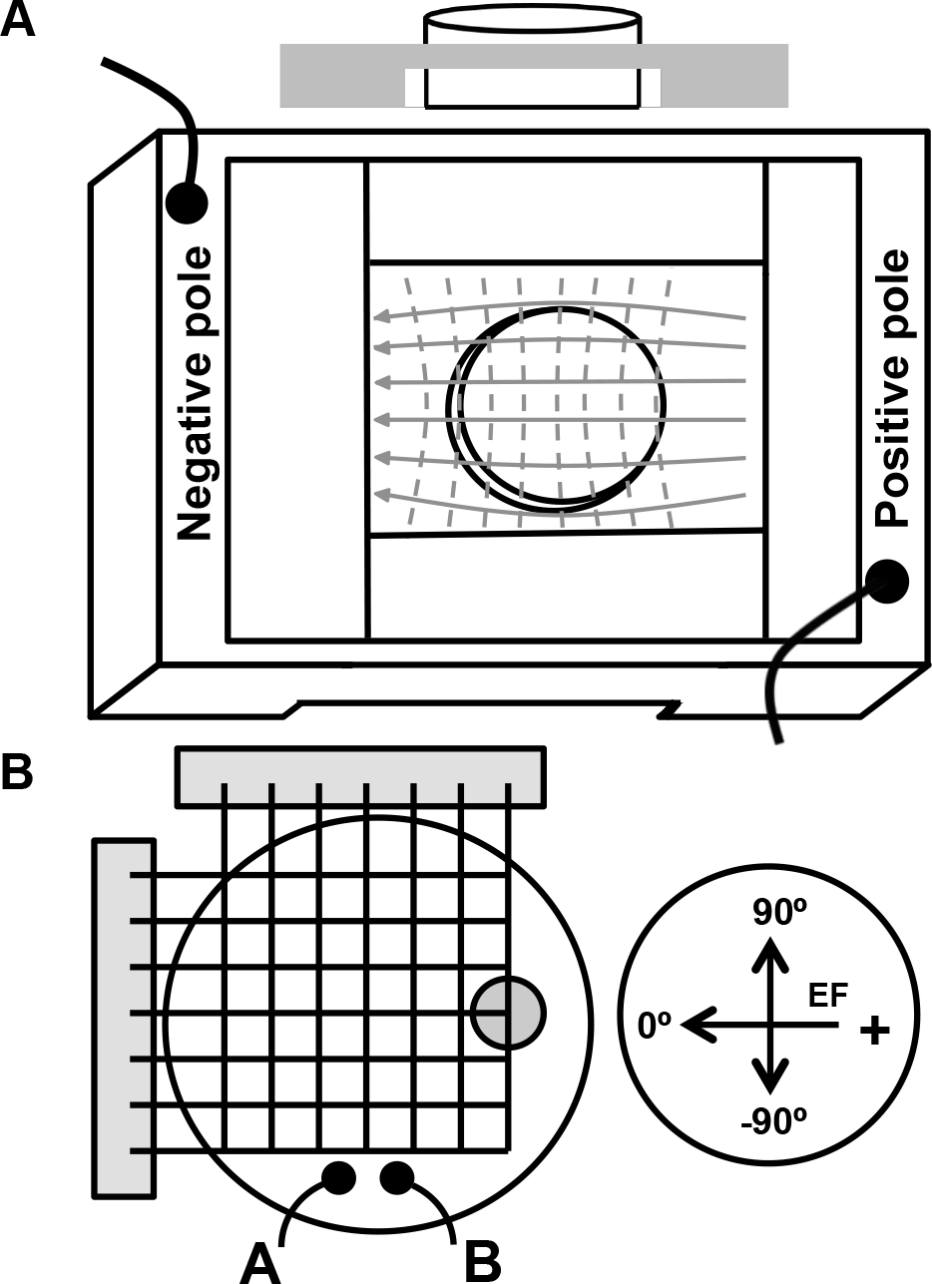
Apparatus for measuring *C*. *elegans* electrotaxis behavior. (A) A modified gel electrophoresis tank is used to generate a uniform electric field in an agar gel. The agar rests in a Plexiglas chamber with a channel that allows for the gel to be semi-submerged in a salt bath while the crawling surface remains above the liquid. The solid gray arrows represent the electric field direction created by applying a potential difference between the baths. (B) Applied electric field strength was determined by measuring voltage between two fixed platinum probes (A and B) spaced 10 mm apart in the direction of the field. The equivalent field strength of trajectory angle was determined by measuring the voltage difference at varying angles and distance from a single point (round circle). Angle coordinates were assigned -90 to 90°. The negative pole is designated 0°.

The electric field apparatus consists of a modified mini-gel electrophoresis chamber (BioRad, Emeryville, CA) that supplies an electric field across an agar disc. The agar disc rests in a Plexiglas channel connecting the two electrophoresis chambers to allow liquid from each chamber to flow around the agar disc (Fig 1A). To make the agar disc, 6 mL of molten agar consisting of 1.7% agar (0.017 g/mL), 7.5 mM HEPES (pH 6.9) and 2 mM ammonium nitrate is poured into a 3-cm petri plate. After cooling, the agar is incubated at 37°C for 6 hours before each experiment to ensure a consistent and uniform crawling surface. The Plexiglas chamber holds the agar gel at mid-level in the bath, keeping it well above the surface of the liquid, to provide a suitable crawling surface for the worms. To confirm that the behavioral response was due to voltage and not current, we utilized a lower concentration of salt in the agar than the bath to ensure that the current across the agar was negligible. This difference in solutions ensured the agar was more dielectric than the bath and less likely to conduct electrical charges. The bath solution is composed of 20 mM ammonium nitrate and 7.5 mM HEPES (pH 6.9). The salt concentrations in both the bath and agar were also selected to control resistive heating (< 0.1°C increase). In order to minimize the effects of resistive heating, the bath solution is circulated between the two reservoirs using a small pump. The HEPES serves to prevent pH changes across the agar surface that would otherwise occur in the presence of an applied electric field and could potentially elicit a behavioral response.

The electric field was generated with a Heath Company Model IP-32 vacuum tube regulated power supply (Saint Joseph, MI). The applied electric field strength (V/cm) was determined by measuring voltage between two platinum probes spaced 10 mm apart in the direction of the field near the edge of the agar disc and outside of the area used for video capture (see Fig 1B). These probes were used to test for field uniformity, monitor electric field strength during experiments, and calculate electric field strength at known trajectory angles. To determine the uniformity of the electric field generated in the agar gel, we constructed a grid using a circuit breadboard containing tungsten coated copper pins in rows and columns spaced 2.5 mm apart (see Fig 1B). Voltage measurements at varying positions were conducted across the entirety of the agar gel. From these measurements, the magnitude and direction of the electric field could be determined at any given point across the agar disc. This method was used to also determine measured field strength at different angles and applied field strengths (S1 Fig).

### Electrotaxis Assay

To conduct electrotaxis experiments, one-day old adult animals were rinsed from the cultivation plate with M9 buffer and transferred to a microfuge tube. The rinsed worms were then centrifuged briefly to remove all but 100 μL of the liquid. Ten μL of liquid, equating to approximately 30 animals, was then pipetted to a thin dry strip of Whatman filter paper after which the animals were transferred to an agar disc (see above) by gently pressing the filter paper to the agar disc. Immediately upon transfer a DC voltage was applied to generate an electric field stimulus and videos were collected.

### Image Acquisition and Analysis

Images were captured using an Imaging Source monochrome CCD camera (DMK 21AF04, The Imaging Source, LLC, Charlotte, North Carolina) with 640X480 pixel resolution, attached to a 6X magnification Navitar zoom lens system (Navitar Inc., Rochester, NY). A Harvard Apparatus V-Lux 1000 cold fiber optic light source (Holliston, MA) was used to illuminate the stage from below through a non-reflective mirror on a Leica Microsystems base (Buffalo Grove, IL). For consistency, all images were captured with animals moving from right to left (positive to negative pole). The VideoCapture function, provided by the WormTracker software [23], captured digital images (15 frames/sec) as four 15-second movies with 30 second intervals between each movie. This captured a total of one minute of locomotion during a 2.5-minute period.

A single replicate trial consists of a compilation of the instantaneous speed and direction data produced by the WormTracker software for each of the four 15-second movies taken of the same population of animals. Within each movie, approximately 20 individual animals were tracked (mean 20.42, standard deviation 8.60). For each track, analysis of speed, the directional component of animal movements, and pirouette identification was performed in the Matlab 2009 (a) software environment using the WormTracker program [23]. The default WormTracker pirouette setting of an angular speed exceeding 110° per second was used to evaluate if a pirouette had occurred within an individual animal tracks. A minimum of three replicates were used for each combination of condition and genotype tested.

To determine the mean speed and the mean trajectory for an entire population, all measurements in the compiled data set were averaged using the WormTracker program. The trajectory diagram and distribution of trajectory angles were generated by additional processing of the WormTracker derived data using the Matlab software environment. Specifically, distributions of trajectory angles were generated by first binning all points from the corresponding angular data sets in one degree increments. Following this, the resultant data was smoothed with a seven-point average sliding window. Finally, in order to allow direct comparisons across populations, values were converted to percentages through division by the total number of points included in the distribution. Graphs of distribution of trajectory angles consist of this normalized frequency data plotted against trajectory angles.

### Statistical Analysis

We applied general or generalized linear mixed-models predicting each response variable (angle, speed, or the probability of an animal making at least one pirouette) as a function of strain, applied field strength, strain-by-applied field strength interaction, replicate, and movie. Replicate and movie were treated as random effects, with movie nested within replicate and replicate nested within each combination of strain and applied field strength. For angle and speed, we used the identity link, assumed a normal distribution of error terms, and allowed for a different error variance within each combination of strain and applied field strength. Angle was square-root transformed prior to analysis in order to conform to normality. For pirouette data, we used the log-link function and a binomial distribution of error terms (i.e., a nested logistic regression). Parameter estimates were optimized via maximum likelihood, and significance was assessed via Wald t-tests (angle and speed data) or Wald Z-tests (pirouette data). We conducted our analyses with open source R statistical software (R Core team 2015) using the lme function of the nlme package [36]. The lsmeans function from the lsmeans package [37] was used to extract estimated means and standard errors for each combination of strain and applied field strength, and to perform pairwise comparisons between these means. We report raw p-values from these tests, but use an adjusted α value of 0.0286 (angle data), 0.0299 (speed data), or 0.0262 (pirouette data) to limit our false discovery rate (FDR) to 0.05 for each response variable.

## Results

### *C*. *elegans* alter trajectory angles to select a specific field strength in a uniform electric field

In order to study the nature of *C*. *elegans* electrosensory behavior, we constructed an apparatus capable of producing a fixed and uniform DC field using a modified standard DNA gel electrophoresis chamber (Fig 1A). A uniform field was generated by placing the agar in a narrow channel between the two baths, which assisted in minimizing the capacitive edge effects. To examine field uniformity, we measured voltages in the direction of the field (X-axis) and perpendicular to it (Y-axis) at electric field strengths of 1.5, 3, 6 and 9 V/cm (Fig 1B). The ratio of voltage to distance, or electric field (V/cm) remained constant in the direction of the field along the X-axis, while voltage measurements taken between any two points along the Y-axis had no voltage difference between them indicating that they are equipotential. Since the field is approximate in uniformity, the animals will experience different electric field strengths based on their trajectory angle across the gel. For example, if the animals move directly toward the negative pole (0°) they will experience the maximum field strength (i.e. 3 V/cm for an applied field strength of 3 V/cm). Alternatively, if the animals move at an angle relative to the negative pole, the animals will experience an electric field strength that is less than the applied electric field. For different applied field strengths, we have measured the electric field strength at different trajectory angles and have shown that the measured field strength decreases with increasing trajectory angles (S1 Fig). Therefore, we have determined our experimental apparatus produces an electric field that is sufficient in uniformity to evaluate behavior on a population of animals at different applied field strengths and to calculate the corresponding field strength for any particular direction of movement.

**Fig 2:**
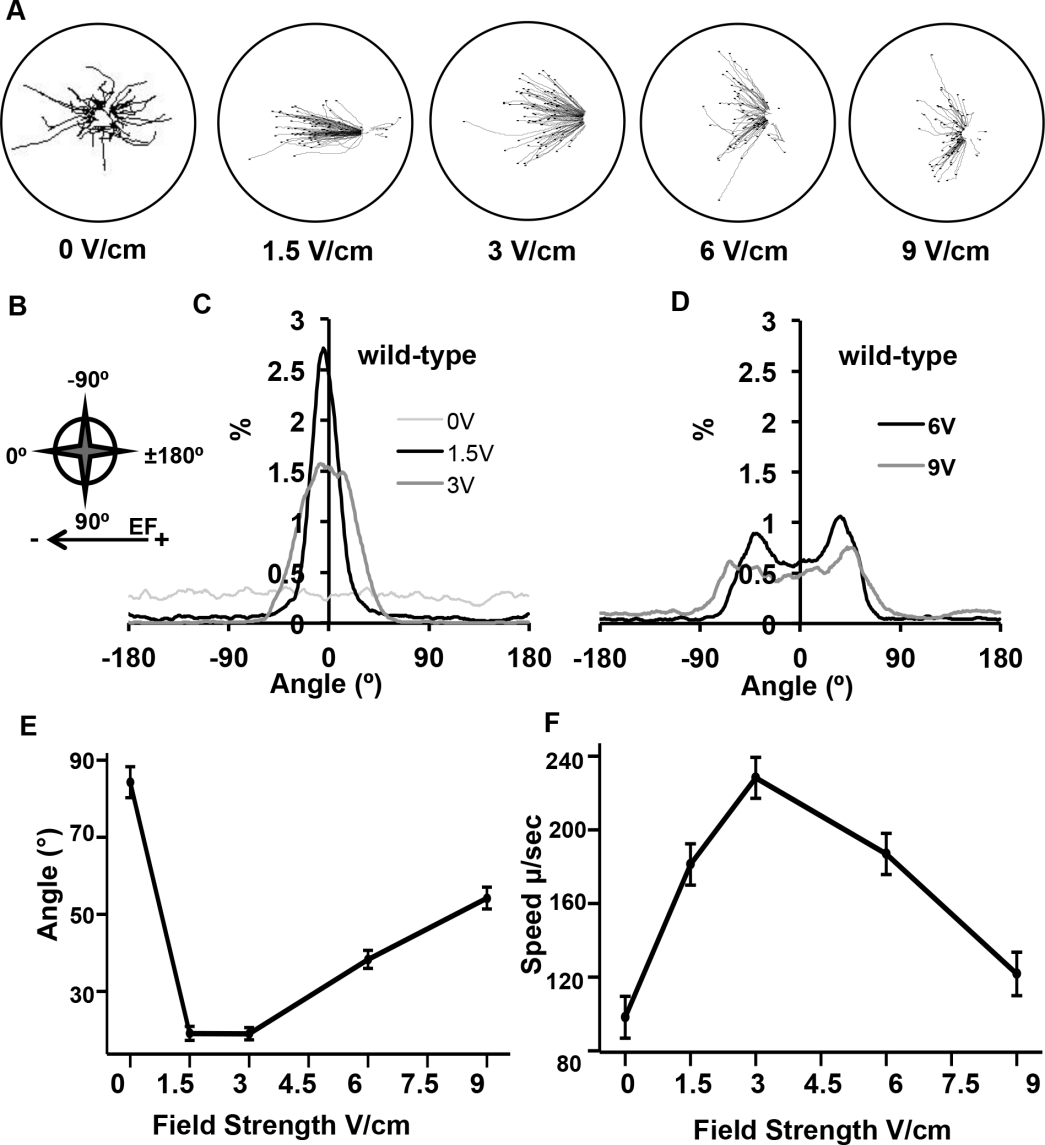
Electrotaxis behavior in wild-type animals at multiple field strengths. (A) Trajectories of individual animals within a population at applied field strengths of 0, 1.5, 3, 6 and 9 V/cm. (B) Orientation of trajectory angles. Angle coordinates were assigned in 1° increments from 0 to ± 180°. Negative and positive angles represent direction above and below X-axis. (C-D) Distribution of trajectory angles for different electric field strengths. Lines are smoothed using a seven point moving average. (E) Mean trajectory angles of wild-type animals are significantly different at all electric field strengths in comparison to non-stimulus at 0 V/cm (*p* ≤ 0.0001). The trajectory angles above (+) and below the X-axis (-) were combined and plotted as absolute values. (F) Mean speed of wild-type animals increases significantly at electric field strengths of 1.5–9 V/cm relative to non-stimulus speed (*p* < 0.0001). For (E) and (F), error bars represent SEM; number of individual animal tracks per genotype (*N*) ≥ 154. *P*-values were determined from post-hoc pairwise comparisons of estimates obtained via general linear mixed-models. Significance was assessed using a false discovery rate of 0.05.

To characterize *C*. *elegans* electrotaxis behavior, we first measured directionality of the animals’ locomotory path to electric field stimuli. We recorded the trajectory tracks generated from a population of animals at applied field strengths of 0, 1.5, 3, 6 and 9 V/cm (Fig 2A). To quantify the trajectory pathways, we divided the agar disc circumference into one-degree increments and assigned the negative pole the 0° coordinate and the positive pole the ±180° coordinate along an X-axis. Above and below the X-axis we assigned coordinates either plus or minus 1° to 179°, respectively (Fig 2B). In the absence of electric field stimulus (0 V/cm) the animals’ trajectory tracks are evenly dispersed in all directions. At 1.5 V/cm, the majority of the animals have aligned their trajectories to the field and at 3 V/cm all of the animals are moving towards the negative pole. We generated distribution curves of the tracks at different field strengths in order to measure the percent of animals aligned with the field. At 1.5 V/cm and 3 V/cm, there is a peak of the trajectory angles centered at 0° (Fig 2C), which indicates that the animals are aligned with the direction of the field. Without electric field stimulus (0 V/cm) there is no peak distribution. The mean trajectory angles at different field strengths were also calculated. Under no electric field stimulus (0 V/cm) the animals had a mean trajectory angle of 84 ± 1.5° compared to mean trajectory angles of 19.3 ± 1.5° and 19.1 ± 1.2° for 1.5 and 3.0 V/cm, respectively (Fig 2E) indicating that measured trajectory angles are lowest at these field strengths.

It has been previously reported that animals prefer to crawl at increasing angles to increasing field strengths [18]. We suspected that there is a correlation between animal trajectories and a preference for a specific field strength. We also hypothesized that animals would need to meet two behavioral criteria in order to demonstrate that they alter trajectories to select for an optimal field strength during electrotaxis. First, at lower applied field strengths, below their preferred field strength, animals would select a trajectory aligned with the field strength in the direction of the electric field (0°) such as is seen at 1.5 and 3.0 V/cm. Second, at higher applied field strengths, above the preferred field strength, animals would avoid the higher field strengths and widen the angles of their trajectories above and below the X-axis where the field strength is highest. At an applied electric field strength of 6 V/cm, the distribution of trajectory angles is distinctively bimodal (Fig 2D) with a mean trajectory angle of 38.5 ± 1.8° (Fig 2E). At 9 V/cm, the animals’ trajectory increases to a mean trajectory angle of 54 ± 3° (Fig 2D and 2E). Because the field is uniform, we could measure the field strength associated with a trajectory angle on the agar surface. For the different applied field strengths, we plotted measured field strength versus specific trajectory angles on the agar plate to generate a slope formula which was used to calculate the electric field corresponding to an animal’s mean trajectory angle (S1 Fig). For field strengths 6 V/cm and 9 V/cm, we determined that the animals’ trajectories corresponded to an electric field strength of 4.95 and 5.08 V/cm, respectively. These results suggest that the animals exposed to a sub-preferred field strength (< 5 V/cm) will align their trajectory with the highest electric field strength; animals exposed to a larger than preferred electric field (> 5 V/cm) will adjust their trajectories to align with electric field strengths of approximately 5 V/cm.

The electrotaxis response is also reflected in the animals’ changes in average speed upon exposure to different applied field strengths. Without an electric field stimulus, the animals have an average speed of 98 ± 10 μm/s (Fig 2F). As animals sense and begin actively crawling towards the negative pole at 1.5 V/cm, their speed increases to 180 ± 9.8 μm/s. At 3 V/cm, where 63% of the animals are aligning their trajectories in a direction of 0 ± 20° towards the negative pole the average speed reaches 228 ± 10 μm/s (Fig 2A and 2F). We speculate that the increase in speed is indicative of long uninterrupted runs that the population of animals displays at these field strengths (see below). The average speed begins to decrease at the higher electric field strengths. At 6 V/cm and 9 V/cm the mean speed is reduced to 186 ± 10 μm/s and to 122 ± 12 μm/s, respectively, possibly due to the animals reorienting their movements to align with a field strength of 5 V/cm.

We focused on three quantitative parameters that allowed us to examine electroreception behavior in wild-type and mutant strains of *C*. *elegans*. First, we determined sensitivity of the electrotaxis response by (1) measuring the animals’ trajectory angles in response to increasing electric field strengths. Secondly, we examined the animals’ change in search behavior during electrotaxis by measuring (2) the frequency of turning behavior (pirouettes) and (3) the average population speed, which inversely correlates to the amount of turning by the animals.

### EAT-4, CEH-36 and NSY-5/INX-19 are necessary for navigation in a uniform electric field

**Fig 3:**
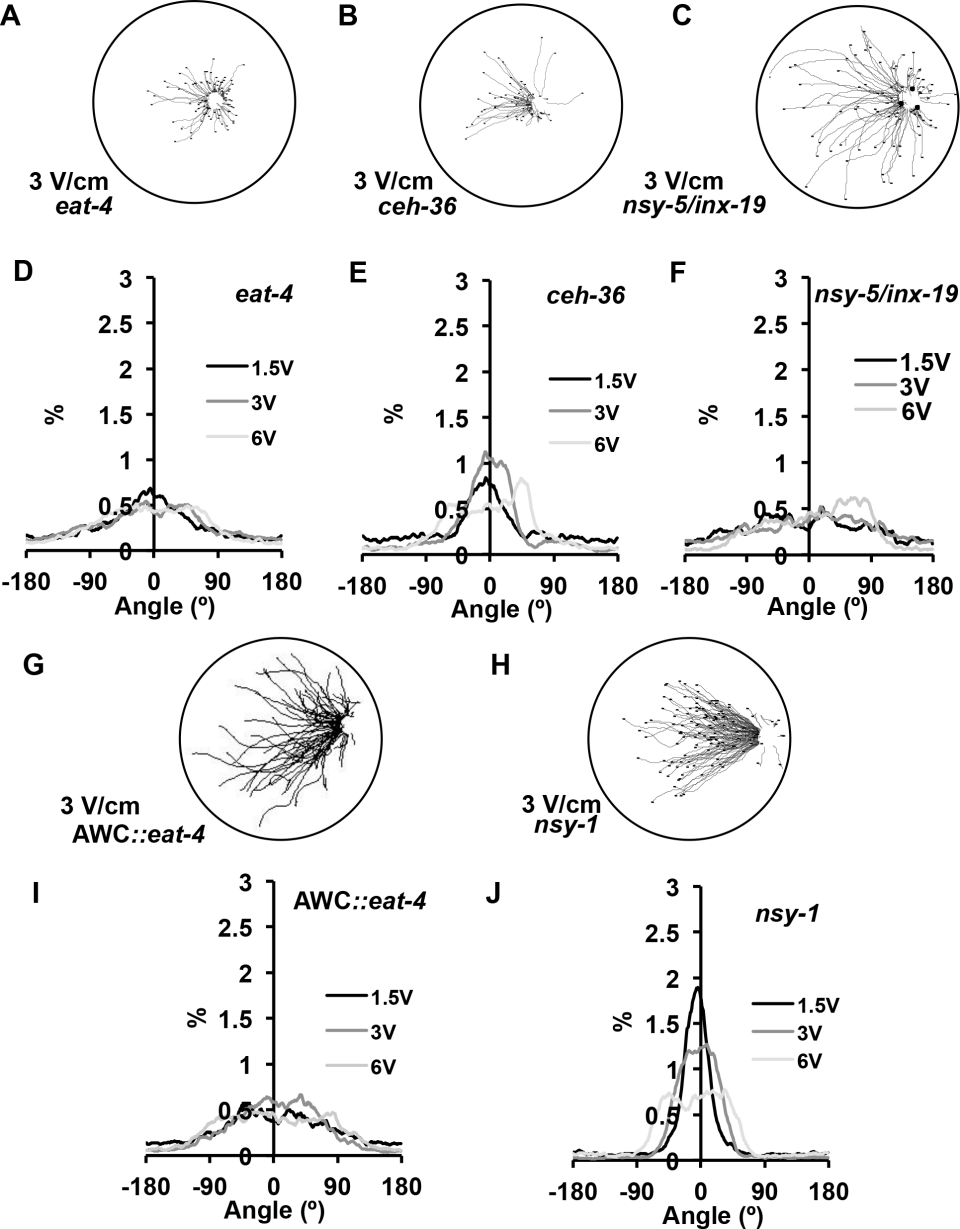
EAT-4, CEH-36 and NSY-5/INX-19-dependent neurons mediate electrotaxis behavior. (A-C) *eat-4(ky5)* mutant animals are defective in glutamatergic signaling and fail to consistently orient and migrate toward the negative pole. *ceh-36* mutant animals, which are defective in terminal differentiation of AWC and ASE neurons, and *inx-19/nsy-5* (AWC^OFF/OFF^) mutant animals, which alter the asymmetric fate of AWC^ON^ neurons also display defects in migration to the negative pole in response to an electric field stimulus. (D-F) Distribution of animal trajectory angles at different electric field strengths. (G-H) *eat-4(*+*)* rescue in AWC neurons (AWC::*eat-4*) animals are defective in response to electric field stimuli. *nsy-1* (AWC^ON/ON^) mutant animals respond strongly to electric field stimuli. (I-J) Distribution of AWC::*eat-4* and *nsy-1* animals’ trajectory angles at different electric field strengths.

**Fig 4:**
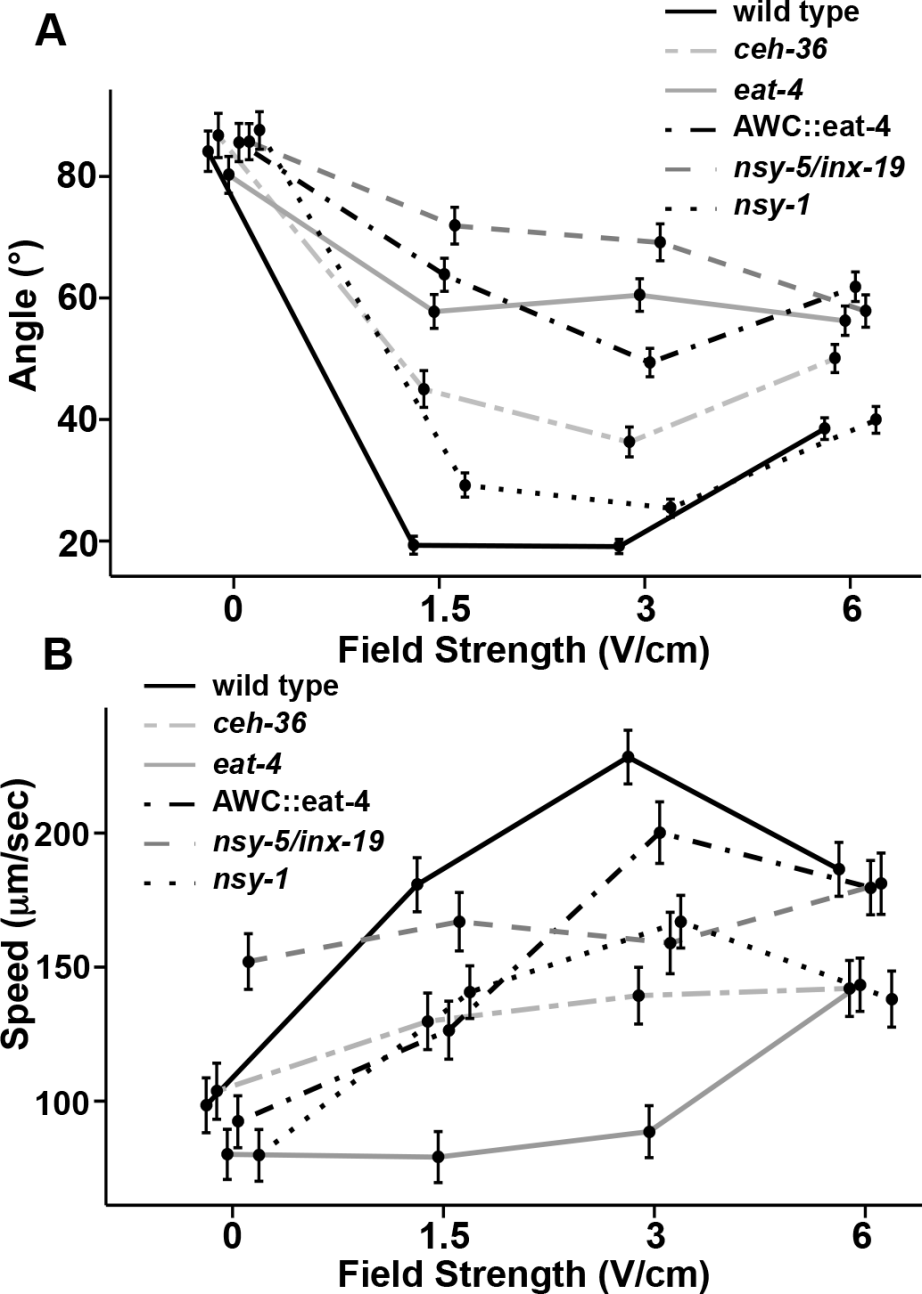
Mean trajectory angles and speeds at different field strengths. (A) Mean trajectory angles of *eat-4(ky5)*, *ceh-36(ky646)* and *nsy-5/inx-19* (AWC^OFF/OFF^) mutant animals are significantly different from wild type at 1.5 and 3 V/cm (*p* ≤ 0.0001). *nsy-1* (AWC^ON/ON^) mutant animals’ mean trajectory angle are slightly wider than that of wild-type animals at 1.5 and 3 V/cm (Fig 4A; *p* ≥ 0.0035). *eat-4(*+*)* rescue in AWC neurons (AWC::*eat-4*) results in a slight lowering of mean trajectory angle compared to *eat-4* mutant animals at 3 V/cm (*p* ≥ 0.0057). (B) Mean speed of different strains. *eat-4* and *ceh-36* mutant animals fail to increase speeds upon application of an electric field stimuli (1.5-6V/cm) when compared to wild type (*p* ≤ 0.007 and *p* ≤ 0.0067,respectively). *nsy-5/inx-19* (AWC^OFF/OFF^) animals do not differ statistically from non-stimulus speeds (0 V/cm) at 1.5V/cm and 3V/cm (*p* ≥ 0.0702). *nsy-1* (AWC^ON/ON^) animals have a significant increase in speed at all field strengths compared to non-stimulus (0 V/cm) speeds (*p* ≤ 0.0005). *eat-4(*+*)* rescue in AWC neurons (AWC::*eat-4*) results in increased speeds at all field strengths (1.5–6 V/cm; *p* ≤ 0.007). Error bars represent SEM; number of individual animal tracks per genotype (*N*) ≥ 154 per calculated average. Data points for different strains were staggered for better visualization. *P*-values were determined from post-hoc pairwise comparisons of estimates obtained via general linear mixed-models. Significance was assessed using a false discovery rate of 0.05.

To determine the neural basis for electrotaxis, we tested existing candidate mutants for responses to electric field stimuli. Previous studies by Gabel *et al*. [18] demonstrated that electrotaxis behavior is dependent on a subset of amphid sensory neurons. To test for a role of the amphid sensory neurons under our electric field conditions, we examined *eat-4* mutant animals. *eat-4* encodes a glutamate vesicular transporter found in approximately 30 neurons, including most of the amphid sensory neurons [38–40]. *eat-4* mutant animals are defective in several glutamate-dependent behaviors including chemotaxis, mechanosensation, thermotaxis and electrotaxis within a rotating field [18, 38, 41]. We tested *eat-4(ky5)* mutant animals in our electrotaxis assay and found the directional component of electrotaxis behavior is severely disrupted. Only 10% of the *eat-4* animals oriented their trajectories in the directional range of 0 ± 20° at 3 V/cm, which is reflected in the slight peak in the distribution at 0° (Fig 3A and 3D). The mean trajectory angles at 1.5 and 3 V/cm are 58 ± 2.8° and 60 ± 2.7°, respectively, which are significantly higher than wild type at those field strengths (Fig 4A; *p* ≤ 0.0001). The average speed of *eat-4* mutant animals in response to electric field strengths of 1.5 V/cm and 3 V/cm were 79 ± 9 μm/s and 89 ± 10 μm/s and were significantly lower than wild type speeds, which were 181 ± 10 μm/s and 228 ± 10 μm/s, respectively, (Fig 4B; *p* ≤ 0.00005). These data support the idea that the loss of synaptic transmission in the glutamatergic amphid neurons in *eat-4* mutant animals results in a significant disruption in both average speed and directionality of movement to electric field stimulus.

To determine which of the EAT-4 expressing amphid sensory neurons might be required for response to a uniform electric field, we examined *ceh-36* mutant animals, which are defective in an OTD/OTX homeodomain protein transcription factor that is required for terminal differentiation of AWC neurons and ASE neurons [42, 43]. An illustration of the defective differentiation of the AWC neurons in the *ceh-36* mutant animals is the lack of expression of the ODR-1 protein in both AWC neurons, and the STR-2 receptor in the AWC^ON^ neuron [25, 42]. The *ceh-36* mutants display a disruption of tracking towards the negative pole with a diminished and broader peak for their trajectory angle distribution (Fig 3B and 3E) and at applied field strengths of 1.5 and 3 V/cm, wider mean trajectory angles of 45 ± 3.0° and 36 ± 2.5° compared to wild-type animals (Fig 4A; *p* ≤ 0.0001). At 3V/cm, *ceh-3*6 mutant animals display a small, although statistically significant increase in speed from 104 ± 10 μm/s to 139 ± 10 μm/s (Fig 4B; *p* = 0.0001), which is significantly lower than wild-type animal speeds of 228 ± 10 μm/s at 3V/cm (*p* = 0.00005). These results indicate that defective differentiation of the AWC and/or ASE neurons severely disrupts the directionality and speed changes associated with the electrotaxis response. *ceh-36* mutant animals have narrower mean trajectory angles compared to *eat-4* mutants (Fig 4A; *p* < 0.0001) suggesting that in addition to AWC and/or ASE, other *eat-4* expressing neurons might contribute to the animals’ response to electric field stimuli.

The AWC neurons are a pair of left-right sensory neurons that show asymmetry in gene expression and chemotaxis responses to different odorants [32, 33]. The AWC neuron expressing the G protein-coupled receptor STR-2 is referred to as AWC^ON^, while the AWC neuron that does not express STR-2 is called AWC^OFF^ [44]. We tested if either or both of the AWC^ON^ and AWC^OFF^ neurons contribute to the behavioral responses to different applied field strengths. We examined *nsy-1(ky397)* mutant animals, which produce an AWC^ON^ phenotype in both neurons (AWC^ON/ON^) [45] and *nsy-5*/*inx-19(ky634)* mutant animals, which produce an AWC^OFF^ phenotype in both neurons (AWC^OFF/OFF^) [46]. We found that the AWC^ON/ON^ mutant animals’ mean trajectory angles of 29 ± 2.0° and 25 ± 1.5° at 1.5 and 3 V/cm are slightly wider than that of wild-type animals, which are 19 ± 1.5° and 19 ± 1.2°, respectively (Fig 4A; *p* ≥ 0.0035). However, these animals still strongly electrotax. The speeds of the AWC^ON/ON^ mutant animals were overall slower than wild-type animals (*p* ≤ 0.0107); however, the speed of the AWC^ON/ON^ animals increased in response to stimulus at all field strengths (*p* ≤ 0.0005), which is consistent with the ability of the AWC^ON/ON^ animals to detect and respond to the electric field, although not as well as wild type. We then tested for a role of the AWC^OFF^ neuron using *nsy-5*/*inx-19(ky634)* mutant animals, and found the ability of the AWC^OFF/OFF^ animals to direct motion towards the negative pole while in an electric field was severely disrupted (Fig 3C and 3F). The mean trajectory angles of the AWC^OFF/OFF^ animals were 72 ± 3° and 69 ± 3° at 1.5 and 3 V/cm, which is significantly wider than both wild type and *nsy-1* mutant animals, (Fig 4A; *p* ≤ 0.0001). Average speed of the AWC^OFF/OFF^ animals did not increase significantly at 1.5 or 3V/cm (Fig 4B; *p* ≥ 0.0702). The comparison of the *nsy-1* and *nsy-5*/*inx-19* mutant animals supports the idea of a role for the AWC^ON^ neuron being the predominant AWC neuron in sensing and/or responding to an electric field stimulus. To confirm a role for AWC neurons in mediating an electrotaxis response, we tested the strain CX6827 (*eat-4(ky5) III; kyEx844* [AWC::*eat- 4*]), which uses the *osm-3* gene promoter to express a functional *eat-4* gene strongly in the pair of AWC neurons, weakly in the AWB neurons and faintly in the AWA, ASH and ADF neurons [47]. Restoration of *eat-4* function in these neurons has been previously shown to restore AWC neuron functionality in the *eat-4* mutant [31]. Interestingly, rescue of *eat-4* gene in AWC neurons produced differential effects on electrotaxis responses. The rescued strain did not show a recovery of directionality in response to an electric field (Fig 4A); there was a slight narrowing of trajectory angle at 3V/cm (49 ± 3°) in the rescued animals compared to *eat-4* mutant animals without the transgene (60 ± 3°; *p* < 0.0057). In contrast, there was a strong response to field stimulus in regard to speed which increased from 92 ± 10 μm/s to 222 ± 11 μm/s at 3V/cm (Fig 4B; *p* < 0.00005), which is significantly higher than *eat-4* mutant animal speeds (89 ± 10 μm/s) at the same field strength (*p* = 0.00005). AWC::*eat-4* animals appear to sense the field stimulus, but are not able to fully recover functionality.

**Fig 5:**
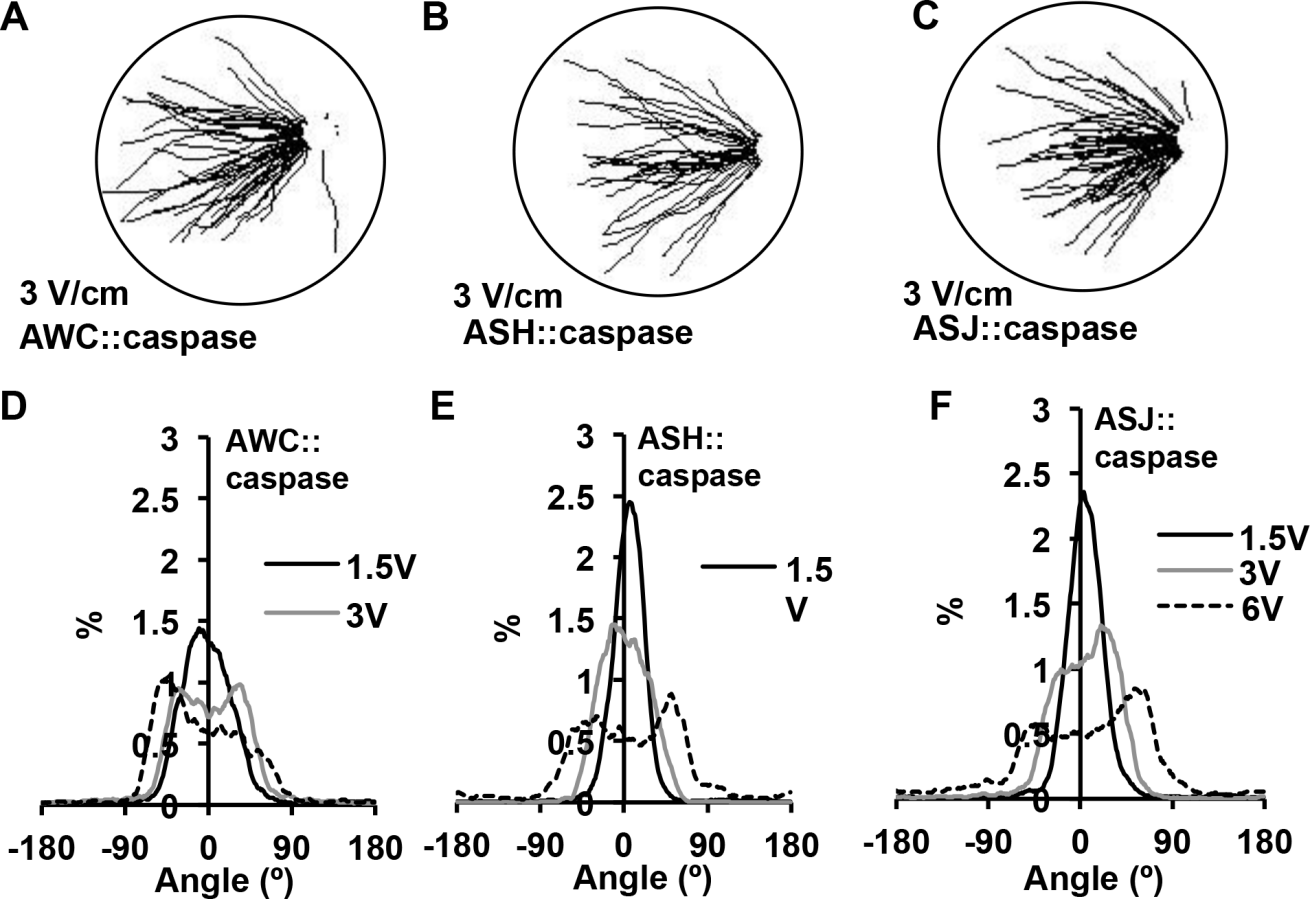
Role of AWC, ASH or ASJ on electrotaxis behavior. (A-C) Trajectory paths of individual animals at 3 V/cm for AWC::caspase, ASH::caspase and ASJ::caspase strains. (D-F) Distribution of animal trajectory angles for different electric field strengths in AWC::caspase, ASH::caspase and ASJ::caspase strains.

**Fig 6:**
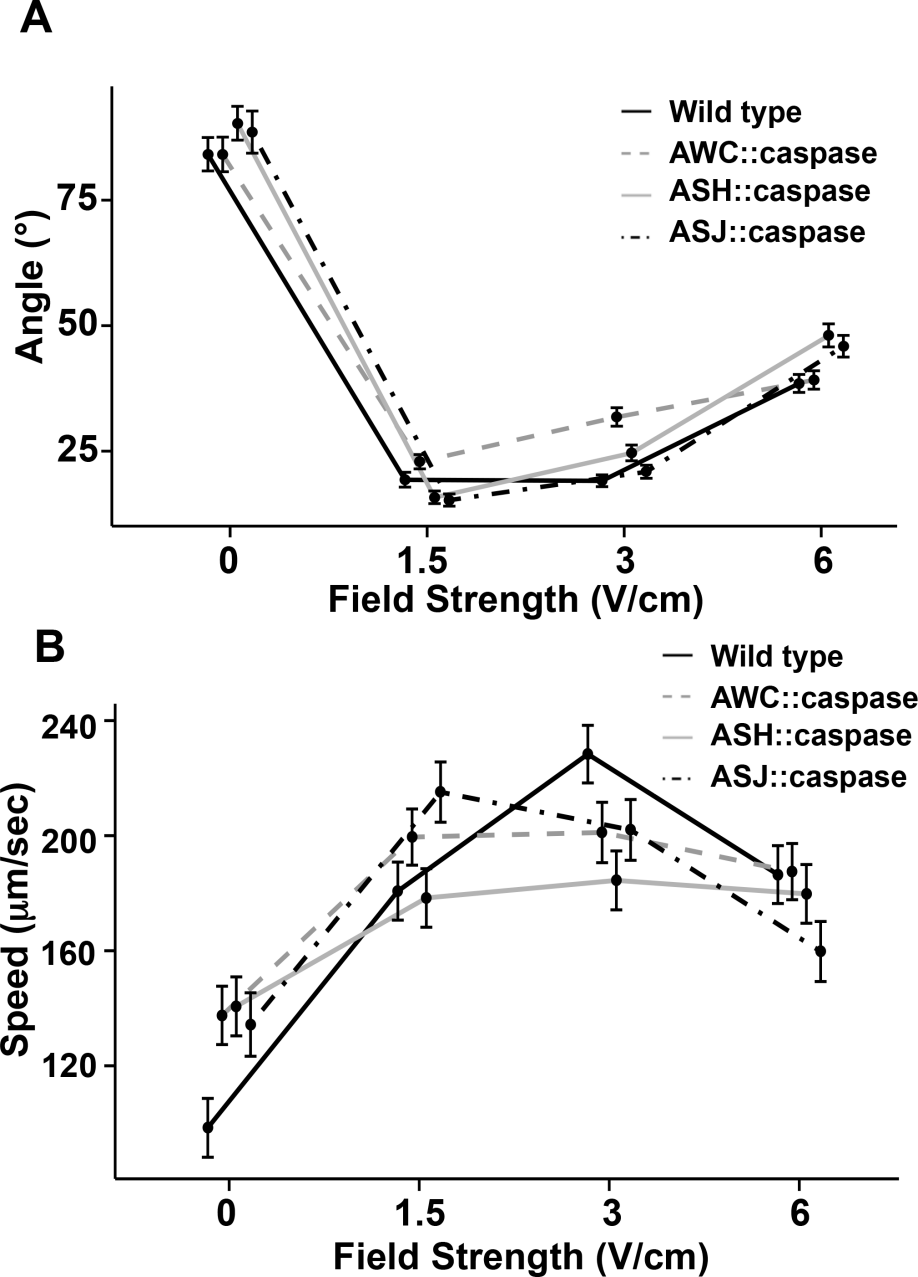
Role of AWC, ASH or ASJ on angle trajectory and speed during electric field stimulus. (A) Mean trajectory angle of AWC::caspase, ASH::caspase and ASJ::caspase strains. At 3V/cm AWC::caspase and ASH::caspase animals show small but statistical difference from wild-type (*p* ≤ 0.0105). (B) Speeds of different strains under electric field stimuli. At all field strengths all strain displayed increases in average speed (*p* ≤ 0.005). Error bars represent SEM; number of individual animal tracks per genotype (*N*) ≥ 154. Data points for different strains were staggered for better visualization. *P*-values were determined from post-hoc pairwise comparisons of estimates obtained via general linear mixed-models. Significance was assessed using a false discovery rate of 0.05.

To further test for a role for AWC, we also examined a strain, PY7502, that produces a caspase-mediated genetic ablation of AWC. The caspase is expressed using a DNA fragment from the *ceh-36* gene promoter that produces expression solely in AWC [28]. This strain, compared with wild-type animals, displayed a broader distribution peak with a lower percentage of trajectory angles around 0° (Fig 5D). The mean trajectory angle (23 ± 1.4°) at 1.5 V/cm was not statistically different from wild type (Fig 6A; *p* = 0.0991), but at 3V/cm the mean trajectory angle of 32 ± 1.8°, is statistically greater than the wild-type trajectory angle of 19 ± 1.2° (*p* = 0.0001). While this difference is statistically significant, the animals are able to perform electrotaxis, as indicated by speed increases that are comparable to wild-type animals across all field strengths (*p* < 0.0768; Fig 6B).

Previous studies have implicated a role for ASH and ASJ neurons in electrotaxis behavior in a rotating electric field. Therefore, we tested two transgenic strains (+*/*+;*nuEx1684* and (+*/*+;*jxEx102)* that produce ablation of the ASH and ASJ neurons, respectively [48, 49]. The ASH genetically ablated strain displayed a slight widening of trajectory angle (25 ± 1.6°; *p =* 0.0105) and the ASJ genetically ablated strain was comparable to wild type at 3V/cm (Fig 6A; *p =* 0.3195). For average speeds, the ASH genetically ablated strain displayed lower speeds (184 ± 10) μm/s than wild type (228 ± 10 μm/s) at 3V/cm (Fig 6B; *p =* 0.0067), while the ASJ genetically ablated strain was comparable to wild type (*p* = 0.087). These results suggest that these neurons are not absolutely required for electrotaxis within a fixed and uniform field, but do support a minor role for ASH in this behavior. It is possible these neurons serve a role in reorientations and reversals associated with large changes in field direction and strength that occurs within a rotating field.

### *C*. *elegans* inhibit exploratory behavior when animal trajectories are aligned in an electric field

**Fig 7:**
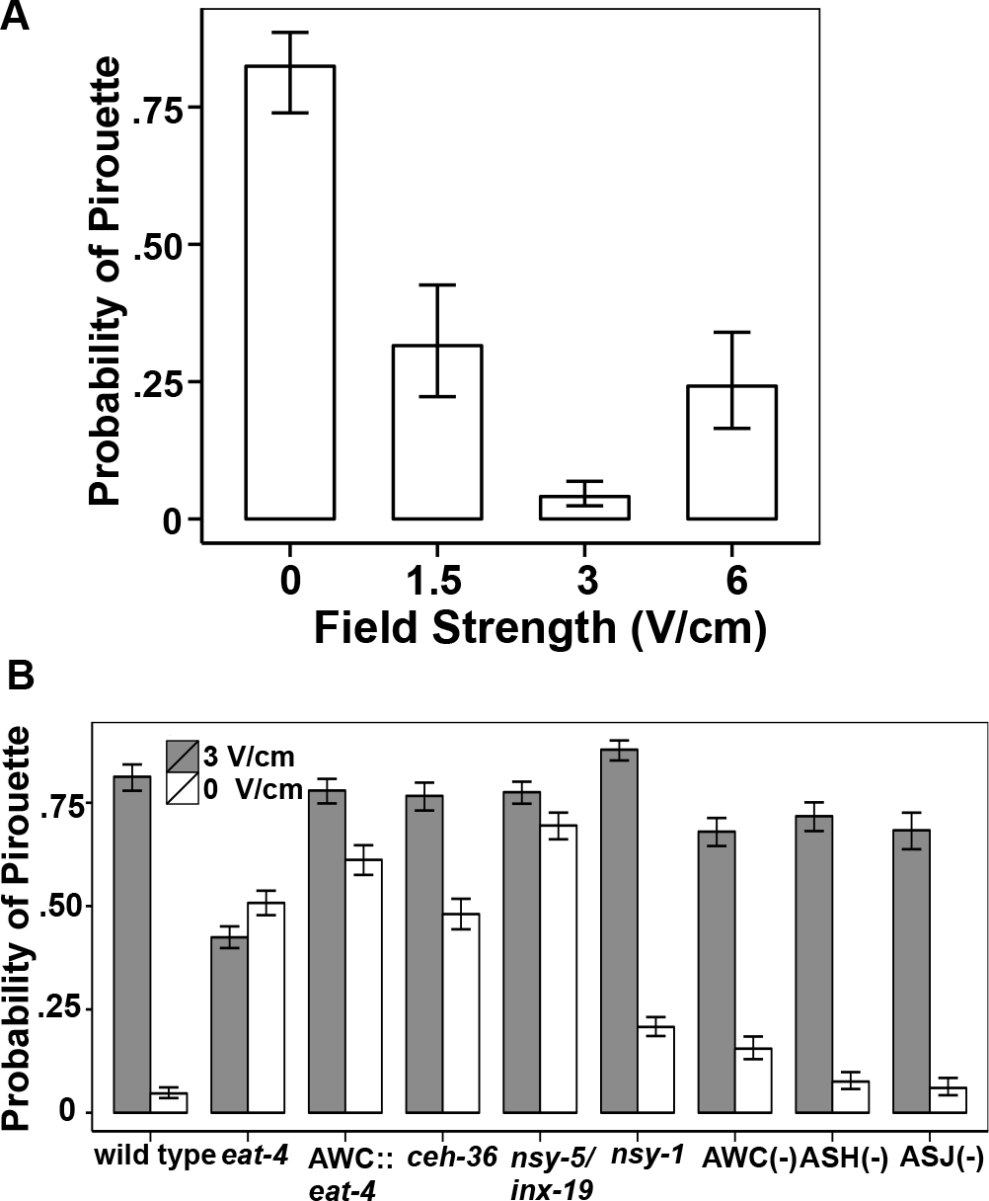
Electric field stimuli suppress pirouette probability. (A) Probability of pirouette behaviors in wild-type animals tracking at different electric field strengths. A single pirouette event was identified if the angular speed equaled or exceeded 110°/second in an individual track. The pirouette probability is suppressed with electric field stimuli of 1.5, 3, and 6 V/cm in comparison to non-stimulus (0 V/cm; *p* < 0.002). (B) Field stimulus and pirouette suppression in different strains. *eat-4(ky5)*, *nsy-5*/*inx-19* (AWC^*OFF/OFF*^) *ceh-36(ky646)* and AWC::*eat-4* mutant animals display defects in suppression of local search behavior with application of an electric field stimulus compared to wild type (*p* ≤ 0.00005). *nsy-1* (AWC^ON/ON^) animals display significant suppression of local search behavior (*p* ≤ 0.00005). AWC::caspase exhibited small, but statistically significant deficits in the suppression of search behavior (*p* = 0.0002). For all trials, error bars represent SEM; number of individual animal tracks per genotype (*N*) ≥ 154. *P*-values were determined from post-hoc pairwise comparisons of estimates obtained via general linear mixed-models. Significance was assessed using a false discovery rate of 0.05.

*C*. *elegans* have a characteristic pattern of uninterrupted runs when aligned to specific electric field strength [7, 18]. We suspected that this behavioral response is due to a switch from local search behavior to a taxis behavior upon exposure to electric field stimulus. We quantified this suppression of search behavior at different field strengths by measuring the frequency of pirouette behavior, which consists of a reversal followed by sharp turns that typically reorient the direction of locomotion [19, 50, 51]. A pirouette event was identified when the angular speed of the animal exceeded a threshold of 110° per second [19, 23]. Using this criterion, we measured the occurrence of at least one pirouette in each animal track to determine the probability of a single animal performing a pirouette. Without an electric field stimulus the wild-type animals have a 0.82 probability of having at least one pirouette event (Fig 7A). In sharp contrast, at a field strength of 3 V/cm, the probability of a pirouette event decreases to 0.05. This suggests that, at a field strength below the preferred field strength, the animals are undergoing long uninterrupted runs toward the negative pole, which corresponds to the significant increases in average speed observed at 3 V/cm.

To determine if a correlation between electrotaxis response and the probability of a pirouette event exists, we tested the mutant strains found to have disruption in electrotaxis function. Compared to wild-type animals, *eat-4(ky5)* mutant animals have a statistically significant reduction in pirouette probability (0.42) under non-stimulus conditions; this probability changes slightly to 0.51 at 3 V/cm (*p* = 0.0261; Fig 7B). This observation is consistent with the known role of amphid sensory neurons, particularly ASK and AWC, in controlling pirouette movements during local search behavior [21]. Consistent with the previous deficits in electrotaxis behavior, *ceh-36* mutant animals showed only a partial suppression of pirouette events from 0.78 with no stimulus to 0.48 at 3 V/cm (Fig 7B; *p* = 0.0005). *nsy-5/inx-19* animals displayed a marginal difference in pirouette probability at 3V/cm (0.78) compared to non-stimulus (Fig 7B; 0.70; *p* = 0.046). *nsy-1* mutant animals significantly suppressed their local search behavior, but not completely to wild type levels (*p* = 0.00005). *eat-4(*+*)* rescue in AWC neurons (AWC::*eat-4*) has negligible effects on suppression of local behavior (0.78 to 0.61; *p* < 0.0003), which is consistent with lack of ability to isotrack to the field stimuli. The AWC::caspase strain showed a statistically significant increase in pirouette probability to 0.12 compared to wild-type (0.05) at 0 V/cm (*p* < 0.0002), while ASH::caspase and ASJ::caspase both showed wild-type responses in probability of pirouette events (Fig 7B). These data are consistent with AWC-influenced suppression of pirouettes playing a role in the navigational strategy used by *C*.*elegans* during electrotaxis.

## Discussion

We have sought to elucidate the navigational strategy that *C*. *elegans* employs to widen their trajectory angle when exposed to increasing field strengths. By defining the relationship between animal trajectory angle and electric field strength in a fixed and uniform field, we have demonstrated that animals will align to the field at lower field strengths (1.5 and 3 V/cm) and will widen their angle of trajectory with higher applied field strengths (6 and 9 V/cm) to match a specific electric field preference (~ 5 V/cm). In addition, we identified several aspects of the electrotaxis behavior that we could quantify using population-based assays. We have demonstrated that animals at a sub-preference field strength of 3V/cm display the lowest mean trajectory angles, the highest average speeds and the greatest suppression of turning behavior. At higher field strengths, the trajectory of the animals bifurcates into wider angles resulting in higher mean trajectories and lower average speeds and less suppression of turning. The changes in navigational strategies in response to increasing field strengths support the notion that in a fixed uniform field the animals are tracking to a preferred field strength.

### *C*. *elegans* employs a klinotaxis strategy for electrotaxis to a preferred field strength in a uniform fixed field

Transforming a static electric field into time varying with movement would be essential for navigation within a specific field range. While an electroreceptor would be insensitive to a static DC electric field, the angular movement generated as the animal undulates in a wavelike motion would cause head sweeps and electroreceptive neurons to travel through multiple field strengths during navigation. *C*. *elegans* may be similar to aquatic organisms with ampullary organs, where angular movement is essential for electroreception in a fixed uniform DC electric field [52].

Within a fixed field, electrotaxis is also strikingly similar to the deterministic behavior *C*. *elegans* employ to track isotherms near their cultivation temperature [6]. In isothermal tracking, the animals’ side to side movement drives the time variation in a thermal gradient to correctly align to a fixed spatial thermal gradient with a sensitivity within 0.1°C [53]. When *C*. *elegans* are aligned in a fixed electric field they have a similar distinctive pattern of uninterrupted tracking devoid of local search behavior [7, 18]. There are several proposed behavioral strategies to explain the spatial orientation that *C*. *elegans* uses to navigate to a sensory stimulus. One is a klinokinesis behavior, which includes a series of random sharp turns that are initiated by reversals in locomotion followed by a sharp body bend and then resumption in forward movement [54]. Another strategy is klinotaxis, where course corrections to a stimulus occur during alterations of the dorsal ventral head sweep over the course of normal animal movement [55]. For an animal to track in a trajectory corridor aligned to specific field strength, it must reliably sense higher and lower variations in electric field gradient within the sweep of the animal’s head.

### Contribution of AWC Neurons to Electrotaxis Responses

To identify the sensory neurons responsible for taxis to select electric field strengths, we examined *eat-4* mutant animals and found they were severely disrupted in electrotaxis as previous reported [18]. The AWC::*eat-4* animal strain, which recovered *eat-4* in AWC and a subset of other neurons, resulted in increased average speeds however, these animals were still largely defective in approach trajectories, suggesting that while they can sense the field these animals cannot isotrack properly. This observation indicates other aspects of neuronal function beside glutamatergic synaptic transmission may be required for AWC function during electrotaxis. *ceh-36* mutant animals, which are defective in terminal differentiation of both AWC neurons and the functional asymmetry between the two ASE neurons, showed significant defects in their ability to respond to and sense direction in an electric field, but not to the same degree as *eat-4* mutant animals. These results, while not definitive suggest a possible role for AWC and/or ASE in contributing to sensing electric field stimuli in *ceh-36* mutant animals. We tested *nsy-1* mutant animals (AWC^ON/ON^) and *nsy-5*/*inx-19* (AWC^OFF/OFF^) mutant animals and determined that AWC^ON/ON^ animals are comparable to wild type, while AWC^OFF/OFF^ animals are severely disrupted in electrotaxis (Figs 3 and 4). NSY-5/INX-19 is expressed in a large number of neurons including the sensory neurons AWC, ASH, AFD, ASI, ADL, ASK, BAG, AWB, ADF, PHA and PHB [46], but not ASE. While the disruption in electrotaxis in the *ceh-36* and *nsy-5/inx-19* mutant animals and lack of disruption in *nsy-1* mutant animals implicate an important role for AWC^ON^, we cannot rule out involvement of other neurons. Surprisingly, in a strain where AWC was genetically ablated, only minor disruptions in electrotaxis behavior were observed. The minimal effect of AWC ablation on electrotaxis is puzzling given the weight of evidence from multiple other genetic manipulations that alter AWC functionality. Collectively, these data suggest that there is a profound difference between mutants that eliminate AWC neurons (AWC::caspase) and mutants that are defective in terminal differentiation and/or their functional asymmetry.

Evidence exists to support the premise that specific neurons function to allow worms to isotrack within a specific stimulus range. The AFD neuron serves as a bidirectional sensor that links both cooling and warming temperature changes to ionic currents with the sweep of the animal’s head [56] and AWC neurons are essential for isothermal tracking and the suppression of turning while at cultivation temperature [30]. We propose that AWC neurons could perform a similar role in electrotaxis as in thermotaxis to allow the animals to detect and migrate to specific field strengths.

AWC neurons have basal activity and tonic release of neurotransmitter and sensory stimulation can inhibit their activity [31, 34]. Specifically, during odor-evoked responses, AWC cells can function as OFF cells, inhibiting neural activity and turning frequency [31, 57]. Gabel *et al*. [18] also observed small calcium transients in AWC neurons with electric field stimuli that occurred when the animal’s anterior was aligned with the positive pole (non-stimulus) supporting the idea that during electrotaxis to the negative pole, neuronal activity is inhibited. We believe that at preferred field strengths, AWC^ON^ could be functioning in a manner similar to its role in chemotaxis by suppressing its activity to cause decreased turning frequency.

Gabel *et al*. [18], using a rotating field, in which the worm alters its direction by reorientation maneuvers, identified a subset of electroreceptive amphid neurons, with ASJ and ASH causing more severe defects and ASK, AWC and AWB causing weaker or little effect. We tested the role of either the ASJ or ASH neurons in a fixed field and found near wild-type responses. We speculate that in a fixed field a neuron such as AWC is important to respond and sense direction, while in a rotating field the ASJ and ASH neurons may be required for the animals to generate large directional changes requiring reversals and pirouettes to track the changing field direction. While we did not find major defects in the absence of ASH function on behavior within a fixed field, we did make a qualitative observation that upon reversing the field, which normally results in a reversal and redirection of locomotion to the new field direction, the ASH-ablated worms produced a significant delay in reorientation after their reversal. This supports the notion that ASH may be important for responding to changing field direction, which would not be necessary in a fixed field, where the animal alters trajectory angles by shallow movements, rather than reversals and reorientation.

We have identified a novel behavioral response in which animals adjust taxis behavior to specific levels of field strength, but questions remain. Future research is needed to elucidate the mechanism by which electric field is sensed. If an electric field is sensed by a receptor protein, then STR-2 is the only currently identified GPCR with specific expression in AWC^ON^ [32] and we have shown that STR-2 is not required for electrotaxis (S2 Fig). There must be other expressed genes/proteins other than *str-2* in AWC^ON^ or in other neurons that are responsible for the ability to detect and/or respond to electric fields. The number of species with known electroreceptive capabilities continues to expand and may be more evolutionary conserved than previously thought. Undoubtedly, electrotaxis behavior will continue to provide insight into how a sensory stimulus is integrated into motor behavior.

## Supporting Information

S1 Dataset Data for trajectory angles and speeds of individual tracks. (PDF)

S2 Dataset Data for pirouettes for each individual track. Tracks were scored (1) for pirouette and (0) no pirouette.
(PDF)

S3 Dataset Measurements for determining angle and measurement field strengths on agar surface (S1 Fig). (PDF)

S1 Fig Measured field strength versus angle at different applied field strength. Voltages were measured at 2.5 mm increments across an agar disc using a grid that contained copper pins at multiple angles and distances from a reference point. The field strength was determined by the voltage and distance for each angle measured. Only distances ≥ 1 cm between measurement points for each angle were plotted. These data points were used to calculate measured field strength at the different applied field strengths of 1.5, 3, 6 and 9 V/cm. The measured field strength versus angle was plotted in a scatter graph in order to characterize their linear relationship. The slope formula was used to determine the specific field strength for the mean animal trajectories at voltages 6 V/cm and 9 V/cm, which corresponded to 4.95 and 5.08, respectively.
(TIF)

S2 Fig Mean trajectory angles and speeds at different field strengths of *str-2* mutant animals. (A) Mean trajectory angles of *str-2* mutant animals. (B) Mean speed of *str-2* mutant animals. Error bars represent SEM; number of individual animal tracks per genotype (*N*) ≥ 154.
(TIF)
