# Supplementary material for: C. elegans Demonstrates Distinct Behaviors within a Fixed and Uniform Electric Field
Source: PLoS One. 2016 Mar 21;11(3):e0151320. doi: 10.1371/journal.pone.0151320 (PMC4801214; doi:10.1371/journal.pone.0151320)
Supplement: S1 Dataset — (PDF) [file pone.0151320.s001.pdf]

strain	volt	rep	movie	track	angle	speed
wild-type	0	1	1	1	61.0497	89.2250
wild-type	0	1	1	2	41.0814	145.0079
wild-type	0	1	1	3	86.0366	49.2994
wild-type	0	1	1	4	90.4583	96.7002
wild-type	0	1	1	5	66.4278	130.1661
wild-type	0	1	1	6	80.5725	88.4269
wild-type	0	1	1	7	89.7882	58.5251
wild-type	0	1	1	8	93.7399	70.4122
wild-type	0	1	1	9	65.4882	85.0306
wild-type	0	1	1	10	92.7558	97.9654
wild-type	0	1	1	11	82.8024	75.6320
wild-type	0	1	1	12	111.2445	104.9078
wild-type	0	1	1	13	56.7533	152.9310
wild-type	0	1	1	14	114.6055	52.2845
wild-type	0	1	2	1	56.1066	34.7420
wild-type	0	1	2	2	18.5205	182.7242
wild-type	0	1	2	3	21.6497	124.3129
wild-type	0	1	2	4	85.1509	81.5682
wild-type	0	1	2	5	100.7682	151.0427
wild-type	0	1	2	6	96.0903	86.0681
wild-type	0	1	2	7	137.8946	132.2820
wild-type	0	1	2	8	124.1582	62.7248
wild-type	0	1	2	9	63.6872	111.7252
wild-type	0	1	2	10	103.8381	21.6397
wild-type	0	1	2	11	145.4105	44.2426
wild-type	0	1	2	12	110.1514	74.9509
wild-type	0	1	2	13	160.0448	219.9344
wild-type	0	1	3	1	95.5817	91.1882
wild-type	0	1	3	2	58.3525	110.9640
wild-type	0	1	3	3	71.3493	113.1933
wild-type	0	1	3	4	82.7788	8.0110
wild-type	0	1	3	5	85.5360	133.3065
wild-type	0	1	3	6	77.3310	50.1287
wild-type	0	1	3	7	85.8451	61.0813
wild-type	0	1	3	8	132.9051	114.2667
wild-type	0	1	3	9	66.9644	186.9394
wild-type	0	1	3	10	171.6239	167.0954
wild-type	0	1	3	11	68.5508	24.1695
wild-type	0	1	4	1	45.9137	82.5355
wild-type	0	1	4	2	137.6249	103.1816
wild-type	0	1	4	3	95.9825	3.0291
wild-type	0	1	4	4	77.0548	4.1739
wild-type	0	1	4	5	122.4290	121.8870
wild-type	0	1	4	6	22.8121	159.9157
wild-type	0	1	4	7	103.0374	123.7442
wild-type	0	1	4	8	91.0523	91.0838
wild-type	0	1	4	9	152.0812	102.3207
wild-type	0	1	4	10	105.5087	67.8782
wild-type	0	1	4	11	138.8295	104.1879
wild-type	0	1	4	12	114.1962	59.4019
wild-type	0	1	4	13	60.4036	34.9698
wild-type	0	1	4	14	40.8054	79.7919
wild-type	0	2	1	1	70.6643	110.4662
wild-type	0	2	1	2	95.0040	45.9166
wild-type	0	2	1	3	94.8767	65.4893
wild-type	0	2	1	4	83.8102	30.9366
wild-type	0	2	1	5	79.7984	71.3923
wild-type	0	2	1	6	99.8606	32.4691
wild-type	0	2	1	7	79.5452	142.7254
wild-type	0	2	1	8	112.5795	41.0538
wild-type	0	2	1	9	96.4706	72.1913
wild-type	0	2	1	10	77.5487	90.7621
wild-type	0	2	1	11	99.1562	44.9712
wild-type	0	2	1	12	62.2079	95.3663
wild-type	0	2	1	13	75.0511	110.0288
wild-type	0	2	1	14	92.5034	98.8935
wild-type	0	2	2	1	111.0463	96.6804

wild-type	0	2	2	2	118.4657	76.4430
wild-type	0	2	2	3	30.9427	194.2530
wild-type	0	2	2	4	108.5234	23.2205
wild-type	0	2	2	5	91.2782	98.8007
wild-type	0	2	2	6	106.6614	67.5019
wild-type	0	2	2	7	29.5772	167.0973
wild-type	0	2	2	8	86.0445	116.9415
wild-type	0	2	2	9	153.6871	188.0457
wild-type	0	2	2	10	75.9991	129.1187
wild-type	0	2	2	11	69.9413	111.5605
wild-type	0	2	3	1	98.8111	116.4587
wild-type	0	2	3	2	48.0121	35.4308
wild-type	0	2	3	3	63.5507	33.6325
wild-type	0	2	3	4	27.2044	79.8107
wild-type	0	2	3	5	89.3488	28.0816
wild-type	0	2	3	6	47.7584	183.5791
wild-type	0	2	4	1	78.1447	128.6116
wild-type	0	2	4	2	82.4588	75.9056
wild-type	0	2	4	3	105.9868	144.3534
wild-type	0	2	4	4	71.4386	33.1129
wild-type	0	2	4	5	102.5080	297.8690
wild-type	0	2	4	6	94.9801	34.4630
wild-type	0	2	4	7	27.2799	140.0415
wild-type	0	2	4	8	166.2504	128.3065
wild-type	0	2	4	9	77.9587	94.5609
wild-type	0	2	4	10	62.4782	108.1511
wild-type	0	2	4	11	29.5866	25.5504
wild-type	0	2	4	12	96.5427	77.6875
wild-type	0	2	4	13	67.7281	71.2755
wild-type	0	2	4	14	83.6089	131.6847
wild-type	0	2	4	15	155.7606	85.5510
wild-type	0	2	4	16	137.7849	74.0028
wild-type	0	2	4	17	85.1953	142.9721
wild-type	0	2	4	18	110.7580	101.5963
wild-type	0	3	1	1	79.4866	75.2942
wild-type	0	3	1	2	74.0458	181.7103
wild-type	0	3	1	3	93.7078	68.1455
wild-type	0	3	1	4	106.0962	38.3030
wild-type	0	3	1	5	109.2726	118.9242
wild-type	0	3	1	6	97.0782	11.1938
wild-type	0	3	1	7	76.6230	44.9276
wild-type	0	3	1	8	100.5519	109.7148
wild-type	0	3	1	9	87.8421	86.3435
wild-type	0	3	1	10	112.8625	107.2663
wild-type	0	3	1	11	97.4069	84.8861
wild-type	0	3	1	12	165.3759	72.6773
wild-type	0	3	1	13	108.2075	71.8224
wild-type	0	3	1	14	47.1041	43.6504
wild-type	0	3	1	15	143.8692	250.6461
wild-type	0	3	1	16	42.8844	109.4370
wild-type	0	3	1	17	45.6647	153.7924
wild-type	0	3	1	18	93.1208	103.0423
wild-type	0	3	1	19	139.7023	17.1432
wild-type	0	3	2	1	81.3738	9.4627
wild-type	0	3	2	2	80.3505	7.4526
wild-type	0	3	2	3	84.4347	77.2963
wild-type	0	3	2	4	86.2789	101.1675
wild-type	0	3	2	5	58.1456	97.8455
wild-type	0	3	2	6	91.0091	10.4179
wild-type	0	3	2	7	92.6163	90.3192
wild-type	0	3	2	8	72.0077	136.2588
wild-type	0	3	2	9	98.2958	113.4430
wild-type	0	3	2	10	78.9142	59.2853
wild-type	0	3	3	1	37.0497	155.9761
wild-type	0	3	3	2	82.6906	282.1475
wild-type	0	3	3	3	86.7828	6.3550
wild-type	0	3	3	4	93.2381	201.2370
wild-type	0	3	3	5	80.7619	35.5286

wild-type	0	3	3	6	96.0274	178.1338
wild-type	0	3	3	7	58.1446	125.1621
wild-type	0	3	3	8	132.3525	158.8977
wild-type	0	3	3	9	63.9392	127.9624
wild-type	0	3	3	10	38.8162	158.9341
wild-type	0	3	3	11	139.3327	17.9838
wild-type	0	3	3	12	131.4215	122.1083
wild-type	0	3	3	13	74.8409	192.5310
wild-type	0	3	3	14	39.1110	177.7987
wild-type	0	3	4	1	119.5770	151.0563
wild-type	0	3	4	2	92.0508	7.3194
wild-type	0	3	4	3	122.6567	132.5136
wild-type	0	3	4	4	105.4470	37.7549
wild-type	0	3	4	5	78.7281	111.4367
wild-type	0	3	4	6	114.1013	116.5663
wild-type	0	3	4	7	62.2087	168.2349
wild-type	0	3	4	8	28.2538	68.4531
wild-type	0	3	4	9	123.5972	171.8939
wild-type	0	3	4	10	58.2271	137.6962
wild-type	1.5	1	1	1	19.6171	206.1145
wild-type	1.5	1	1	2	42.8080	249.9990
wild-type	1.5	1	1	3	13.3483	230.9629
wild-type	1.5	1	1	4	29.4020	149.8728
wild-type	1.5	1	1	5	8.8180	157.1612
wild-type	1.5	1	2	1	4.8725	191.6512
wild-type	1.5	1	2	2	12.3483	255.3936
wild-type	1.5	1	2	3	15.1204	187.2126
wild-type	1.5	1	2	4	19.0157	198.4947
wild-type	1.5	1	2	5	12.1283	321.7307
wild-type	1.5	1	2	6	6.8827	185.8986
wild-type	1.5	1	2	7	5.2143	194.3700
wild-type	1.5	1	2	8	7.4668	137.7100
wild-type	1.5	1	2	9	12.2482	145.4952
wild-type	1.5	1	2	10	6.5615	201.3318
wild-type	1.5	1	2	11	9.6461	140.6708
wild-type	1.5	1	2	12	10.9070	178.9982
wild-type	1.5	1	2	13	17.7460	254.0002
wild-type	1.5	1	2	14	40.8998	205.1251
wild-type	1.5	1	2	15	4.7504	270.7254
wild-type	1.5	1	2	16	7.9901	275.3071
wild-type	1.5	1	2	17	6.6208	154.8625
wild-type	1.5	1	2	18	8.8861	189.5046
wild-type	1.5	1	2	19	6.2283	190.5903
wild-type	1.5	1	2	20	71.0580	25.1245
wild-type	1.5	1	2	21	3.9877	178.3384
wild-type	1.5	1	2	22	15.5156	197.4246
wild-type	1.5	1	2	23	8.2075	228.2242
wild-type	1.5	1	2	24	9.8845	196.6493
wild-type	1.5	1	3	1	7.2704	202.7102
wild-type	1.5	1	3	2	11.6773	175.7969
wild-type	1.5	1	3	3	13.3697	229.4776
wild-type	1.5	1	3	4	13.6139	234.1701
wild-type	1.5	1	3	5	18.3404	156.2776
wild-type	1.5	1	3	6	4.7599	163.1626
wild-type	1.5	1	3	7	6.6984	204.0586
wild-type	1.5	1	3	8	7.3022	207.9022
wild-type	1.5	1	3	9	10.0996	196.7491
wild-type	1.5	1	3	10	87.1072	100.0237
wild-type	1.5	1	3	11	20.3219	132.0686
wild-type	1.5	1	3	12	5.5225	236.3624
wild-type	1.5	1	3	13	12.1044	233.2856
wild-type	1.5	1	3	14	115.1012	151.2633
wild-type	1.5	1	3	15	10.1002	219.4209
wild-type	1.5	1	3	16	35.1582	226.7476
wild-type	1.5	1	3	17	26.4429	151.7710
wild-type	1.5	1	3	18	83.8624	111.8383
wild-type	1.5	1	3	19	8.5913	198.3704
wild-type	1.5	1	3	20	11.4230	212.7180

wild-type	1.5	1	3	21	4.2405	255.0526
wild-type	1.5	1	3	22	12.0868	143.4644
wild-type	1.5	1	3	23	5.0706	267.3187
wild-type	1.5	1	3	24	3.5862	201.0289
wild-type	1.5	1	3	25	7.3428	178.5342
wild-type	1.5	1	3	26	15.5233	84.4585
wild-type	1.5	1	3	27	48.0133	205.1670
wild-type	1.5	1	3	28	15.1753	238.3817
wild-type	1.5	1	3	29	9.9562	254.9305
wild-type	1.5	1	3	30	4.5003	214.0454
wild-type	1.5	1	3	31	5.6488	327.6342
wild-type	1.5	1	3	32	19.5236	155.7329
wild-type	1.5	1	4	1	12.2672	235.9926
wild-type	1.5	1	4	2	24.9045	192.0604
wild-type	1.5	1	4	3	9.9975	240.5029
wild-type	1.5	1	4	4	7.2526	115.9325
wild-type	1.5	1	4	5	10.2777	241.0701
wild-type	1.5	1	4	6	4.2333	184.9301
wild-type	1.5	1	4	7	5.6664	254.2627
wild-type	1.5	1	4	8	10.5668	95.5146
wild-type	1.5	1	4	9	12.6918	181.1026
wild-type	1.5	1	4	10	58.2865	215.7047
wild-type	1.5	1	4	11	10.7852	163.1188
wild-type	1.5	1	4	12	6.0444	146.4669
wild-type	1.5	1	4	13	11.9170	174.5756
wild-type	1.5	1	4	14	12.2704	186.3468
wild-type	1.5	1	4	15	12.2443	172.0421
wild-type	1.5	1	4	16	12.5854	125.1526
wild-type	1.5	1	4	17	29.3821	148.8537
wild-type	1.5	1	4	18	5.3375	209.8747
wild-type	1.5	1	4	19	100.4057	89.6898
wild-type	1.5	1	4	20	7.2978	206.6131
wild-type	1.5	1	4	21	7.0444	183.1575
wild-type	1.5	1	4	22	5.5079	175.8405
wild-type	1.5	2	1	1	13.1451	160.6343
wild-type	1.5	2	1	2	11.9285	82.5729
wild-type	1.5	2	1	3	3.9091	184.8312
wild-type	1.5	2	1	4	48.4932	204.6675
wild-type	1.5	2	1	5	6.9809	130.9749
wild-type	1.5	2	1	6	9.4412	72.4017
wild-type	1.5	2	1	7	30.9253	65.4133
wild-type	1.5	2	1	8	14.4281	152.5234
wild-type	1.5	2	1	9	6.4475	154.7945
wild-type	1.5	2	1	10	12.1375	187.6199
wild-type	1.5	2	1	11	95.2366	28.0607
wild-type	1.5	2	1	12	9.5978	225.3950
wild-type	1.5	2	1	13	63.0488	40.6266
wild-type	1.5	2	1	14	16.3426	95.1736
wild-type	1.5	2	2	1	14.4820	142.5906
wild-type	1.5	2	2	2	8.9136	189.4268
wild-type	1.5	2	2	3	15.3328	286.3949
wild-type	1.5	2	2	4	6.1085	231.8390
wild-type	1.5	2	2	5	6.2176	162.8090
wild-type	1.5	2	2	6	22.0189	105.0923
wild-type	1.5	2	2	7	6.0830	116.8654
wild-type	1.5	2	2	8	8.5466	132.5596
wild-type	1.5	2	2	9	8.4006	265.7750
wild-type	1.5	2	2	10	6.1321	271.9101
wild-type	1.5	2	2	11	11.6994	93.9322
wild-type	1.5	2	2	12	5.3992	170.2948
wild-type	1.5	2	2	13	8.1214	175.0898
wild-type	1.5	2	2	14	10.1227	114.1041
wild-type	1.5	2	2	15	6.1797	122.3675
wild-type	1.5	2	2	16	12.8877	192.0475
wild-type	1.5	2	2	17	5.8349	174.6047
wild-type	1.5	2	2	18	17.3712	48.3651
wild-type	1.5	2	2	19	63.6326	27.8420
wild-type	1.5	2	2	20	5.8357	198.0987

wild-type	1.5	2	2	21	5.4972	202.0752
wild-type	1.5	2	2	22	6.8843	315.5510
wild-type	1.5	2	2	23	7.9547	228.0304
wild-type	1.5	2	2	24	7.4491	198.4853
wild-type	1.5	2	2	25	6.0415	180.9941
wild-type	1.5	2	2	26	6.3822	157.4949
wild-type	1.5	2	2	27	3.5578	169.6791
wild-type	1.5	2	2	28	6.4687	143.0147
wild-type	1.5	2	3	1	18.6449	154.1555
wild-type	1.5	2	3	2	13.8333	290.7682
wild-type	1.5	2	3	3	5.9589	288.0439
wild-type	1.5	2	3	4	5.7878	237.9540
wild-type	1.5	2	3	5	10.6331	276.6561
wild-type	1.5	2	3	6	17.6328	168.2753
wild-type	1.5	2	3	7	7.8033	239.2966
wild-type	1.5	2	3	8	4.7191	267.0336
wild-type	1.5	2	3	9	6.5993	249.2524
wild-type	1.5	2	3	10	7.1778	258.2193
wild-type	1.5	2	3	11	10.5845	191.3502
wild-type	1.5	2	3	12	7.4409	205.4104
wild-type	1.5	2	3	13	7.5769	222.0502
wild-type	1.5	2	3	14	5.4984	265.5353
wild-type	1.5	2	3	15	5.9396	167.5630
wild-type	1.5	2	3	16	13.0580	202.3122
wild-type	1.5	2	3	17	12.5321	189.4012
wild-type	1.5	2	3	18	38.3790	129.3861
wild-type	1.5	2	3	19	5.9352	229.5635
wild-type	1.5	2	3	20	6.8586	191.2850
wild-type	1.5	2	3	21	10.3420	203.1895
wild-type	1.5	2	3	22	7.1374	176.3774
wild-type	1.5	2	3	23	12.9884	138.4581
wild-type	1.5	2	3	24	12.0638	219.5461
wild-type	1.5	2	3	25	2.7936	126.0865
wild-type	1.5	2	3	26	11.3210	82.2544
wild-type	1.5	2	3	27	8.6248	141.5930
wild-type	1.5	2	3	28	7.0595	155.5964
wild-type	1.5	2	3	29	19.1925	176.4509
wild-type	1.5	2	3	30	8.2536	192.1040
wild-type	1.5	2	3	31	9.8078	202.2895
wild-type	1.5	2	3	32	5.4173	211.6389
wild-type	1.5	2	3	33	12.0617	165.2550
wild-type	1.5	2	3	34	7.0280	206.4594
wild-type	1.5	2	3	35	10.2977	234.1665
wild-type	1.5	2	3	36	25.0950	180.9924
wild-type	1.5	2	3	37	5.5284	206.7580
wild-type	1.5	2	3	38	6.7035	212.5208
wild-type	1.5	2	3	39	39.8314	132.7732
wild-type	1.5	2	3	40	14.5655	191.2389
wild-type	1.5	2	3	41	11.7551	178.4344
wild-type	1.5	2	3	42	14.1953	201.8736
wild-type	1.5	2	3	43	7.1999	123.5492
wild-type	1.5	2	3	44	14.4694	161.1931
wild-type	1.5	2	3	45	4.4986	215.3694
wild-type	1.5	2	3	46	24.4761	97.0423
wild-type	1.5	2	4	1	19.5274	198.4103
wild-type	1.5	2	4	2	12.7627	179.8373
wild-type	1.5	2	4	3	24.4665	131.8627
wild-type	1.5	2	4	4	29.9788	157.3301
wild-type	1.5	2	4	5	40.0270	153.7552
wild-type	1.5	2	4	6	42.6355	131.9707
wild-type	1.5	2	4	7	21.1668	157.1903
wild-type	1.5	2	4	8	13.3236	141.5635
wild-type	1.5	2	4	9	10.0744	167.2535
wild-type	1.5	2	4	10	6.7070	254.1542
wild-type	1.5	2	4	11	6.6430	165.4036
wild-type	1.5	2	4	12	9.1956	159.2545
wild-type	1.5	2	4	13	31.1700	149.9437
wild-type	1.5	2	4	14	28.0956	149.3423

wild-type	1.5	2	4	15	26.3450	97.1307
wild-type	1.5	2	4	16	30.4581	122.8191
wild-type	1.5	2	4	17	9.2894	150.1113
wild-type	1.5	2	4	18	6.3579	156.5229
wild-type	1.5	2	4	19	10.7025	140.6940
wild-type	1.5	2	4	20	33.4522	99.9304
wild-type	1.5	2	4	21	15.3313	158.0903
wild-type	1.5	2	4	22	16.1729	120.2369
wild-type	1.5	2	4	23	21.3541	227.1196
wild-type	1.5	2	4	24	23.9664	234.2847
wild-type	1.5	2	4	25	32.2643	176.9882
wild-type	1.5	2	4	26	120.3051	36.8489
wild-type	1.5	2	4	27	12.9912	178.6208
wild-type	1.5	2	4	28	49.8659	49.8361
wild-type	1.5	2	4	29	9.1573	147.6106
wild-type	1.5	2	4	30	17.5898	177.5006
wild-type	1.5	2	4	31	32.2622	152.1347
wild-type	1.5	2	4	32	9.0366	205.6789
wild-type	1.5	2	4	33	13.8311	159.9848
wild-type	1.5	2	4	34	12.0280	255.8890
wild-type	1.5	2	4	35	6.5949	165.4501
wild-type	1.5	2	4	36	7.9724	194.5766
wild-type	1.5	2	4	37	39.6969	106.4489
wild-type	1.5	2	4	38	37.6413	81.0872
wild-type	1.5	2	4	39	8.1333	157.2805
wild-type	1.5	2	4	40	39.3584	89.8595
wild-type	1.5	2	4	41	4.0969	198.9465
wild-type	1.5	2	4	42	8.2319	166.4053
wild-type	1.5	2	4	43	7.0945	80.5382
wild-type	1.5	2	4	44	6.3750	181.3484
wild-type	1.5	2	4	45	21.8094	233.5984
wild-type	1.5	2	4	46	31.5655	183.5901
wild-type	1.5	2	4	47	32.2262	36.6023
wild-type	1.5	2	4	48	11.9618	159.5831
wild-type	1.5	2	4	49	6.0172	175.4011
wild-type	1.5	3	1	1	105.0041	140.9320
wild-type	1.5	3	1	2	90.6600	167.5463
wild-type	1.5	3	1	3	55.4261	112.7475
wild-type	1.5	3	1	4	103.4209	27.4904
wild-type	1.5	3	1	5	14.0022	237.0504
wild-type	1.5	3	1	6	87.9975	52.5456
wild-type	1.5	3	1	7	48.7548	158.6664
wild-type	1.5	3	1	8	100.4400	131.8148
wild-type	1.5	3	1	9	126.4731	118.8670
wild-type	1.5	3	2	1	6.3670	393.1978
wild-type	1.5	3	2	2	7.8748	321.0721
wild-type	1.5	3	2	3	14.0909	282.3210
wild-type	1.5	3	2	4	8.6969	246.5539
wild-type	1.5	3	2	5	77.6848	74.3467
wild-type	1.5	3	2	6	23.8953	129.4173
wild-type	1.5	3	2	7	16.6183	161.0620
wild-type	1.5	3	2	8	55.8294	157.6030
wild-type	1.5	3	2	9	54.0420	205.2591
wild-type	1.5	3	2	10	12.9271	289.3472
wild-type	1.5	3	2	11	7.2250	135.5371
wild-type	1.5	3	2	12	91.3071	85.8665
wild-type	1.5	3	2	13	40.4677	88.5629
wild-type	1.5	3	2	14	85.1914	133.6095
wild-type	1.5	3	2	15	125.6063	377.7460
wild-type	1.5	3	2	16	48.6125	99.2733
wild-type	1.5	3	2	17	82.5444	96.5067
wild-type	1.5	3	2	18	49.5986	98.9576
wild-type	1.5	3	2	19	5.9009	345.3603
wild-type	1.5	3	2	20	90.3402	71.7302
wild-type	1.5	3	3	1	10.8252	283.2512
wild-type	1.5	3	3	2	12.2871	315.5694
wild-type	1.5	3	3	3	7.0514	302.7222
wild-type	1.5	3	3	4	14.9847	353.4126

wild-type	1.5	3	3	5	8.0495	251.4195
wild-type	1.5	3	3	6	18.4595	270.2756
wild-type	1.5	3	3	7	7.0566	241.3994
wild-type	1.5	3	3	8	40.9317	259.2821
wild-type	1.5	3	3	9	80.7378	111.1967
wild-type	1.5	3	3	10	88.5351	98.7206
wild-type	1.5	3	3	11	40.3457	234.8834
wild-type	1.5	3	3	12	63.1394	242.0283
wild-type	1.5	3	3	13	6.3443	263.4856
wild-type	1.5	3	3	14	14.7448	188.1179
wild-type	1.5	3	3	15	55.9279	178.8706
wild-type	1.5	3	3	16	42.2744	52.4119
wild-type	1.5	3	3	17	8.5956	162.5299
wild-type	1.5	3	3	18	98.4005	56.4243
wild-type	1.5	3	3	19	78.4978	257.4555
wild-type	1.5	3	3	20	80.6673	3.9986
wild-type	1.5	3	3	21	21.5750	62.8579
wild-type	1.5	3	3	22	9.0979	291.0973
wild-type	1.5	3	3	23	28.1891	285.3177
wild-type	1.5	3	3	24	10.1770	337.5490
wild-type	1.5	3	3	25	10.2772	256.3688
wild-type	1.5	3	3	26	24.3462	342.8853
wild-type	1.5	3	3	27	9.3416	102.2515
wild-type	1.5	3	4	1	3.5839	312.6075
wild-type	1.5	3	4	2	34.5376	211.0436
wild-type	1.5	3	4	3	10.6483	204.0181
wild-type	1.5	3	4	4	60.0554	149.2124
wild-type	1.5	3	4	5	36.3717	111.9873
wild-type	1.5	3	4	6	12.4793	232.9140
wild-type	1.5	3	4	7	120.3185	113.5477
wild-type	1.5	3	4	8	8.5774	174.1721
wild-type	1.5	3	4	9	40.0642	104.7067
wild-type	1.5	3	4	10	84.4937	92.0750
wild-type	1.5	3	4	11	10.9185	271.2007
wild-type	3	1	1	1	10.3584	212.7993
wild-type	3	1	1	2	18.4129	250.4307
wild-type	3	1	1	3	44.2044	217.9760
wild-type	3	1	1	4	7.6292	202.8302
wild-type	3	1	1	5	33.1505	226.0017
wild-type	3	1	1	6	35.4068	226.6780
wild-type	3	1	1	7	41.2790	247.4752
wild-type	3	1	1	8	28.0128	226.6772
wild-type	3	1	1	9	32.4366	219.7304
wild-type	3	1	1	10	11.8957	234.7948
wild-type	3	1	1	11	47.8389	127.0396
wild-type	3	1	1	12	30.2412	214.6582
wild-type	3	1	1	13	26.9174	233.6109
wild-type	3	1	2	1	14.6704	349.8872
wild-type	3	1	2	2	28.8384	184.1939
wild-type	3	1	2	3	24.9912	184.2122
wild-type	3	1	2	4	32.8633	133.0576
wild-type	3	1	2	5	6.0551	143.8140
wild-type	3	1	2	6	44.9995	130.5115
wild-type	3	1	2	7	32.6001	204.2628
wild-type	3	1	2	8	29.8851	153.4619
wild-type	3	1	2	9	30.8913	226.8125
wild-type	3	1	2	10	9.4782	172.3819
wild-type	3	1	2	11	8.9228	117.6026
wild-type	3	1	2	12	43.9229	189.7914
wild-type	3	1	2	13	32.9861	184.0355
wild-type	3	1	2	14	14.8958	216.5185
wild-type	3	1	2	15	31.4193	194.8152
wild-type	3	1	2	16	22.7339	221.2453
wild-type	3	1	2	17	17.3368	184.1519
wild-type	3	1	2	18	27.2860	137.8057
wild-type	3	1	2	19	13.8937	152.1444
wild-type	3	1	2	20	11.0023	215.5286
wild-type	3	1	3	1	46.2227	131.3371

wild-type	3	1	3	2	32.7747	182.2170
wild-type	3	1	3	3	20.8087	100.1822
wild-type	3	1	3	4	28.6664	210.1017
wild-type	3	1	3	5	45.1062	192.4540
wild-type	3	1	3	6	20.0089	167.2812
wild-type	3	1	3	7	15.7554	193.4698
wild-type	3	1	3	8	23.4248	188.5340
wild-type	3	1	3	9	7.5365	125.6197
wild-type	3	1	3	10	23.6473	175.9741
wild-type	3	1	3	11	21.0994	163.8512
wild-type	3	1	3	12	6.4818	214.4345
wild-type	3	1	3	13	20.0425	237.8855
wild-type	3	1	3	14	24.6984	174.4146
wild-type	3	1	3	15	28.3784	240.3771
wild-type	3	1	3	16	42.0289	119.5124
wild-type	3	1	3	17	34.5578	213.4115
wild-type	3	1	3	18	44.5652	70.7712
wild-type	3	1	3	19	39.2797	178.1917
wild-type	3	1	3	20	22.5643	155.1921
wild-type	3	1	3	21	12.8989	142.4125
wild-type	3	1	3	22	23.4378	214.3335
wild-type	3	1	3	23	16.0771	86.3085
wild-type	3	1	3	24	13.5956	185.1522
wild-type	3	1	3	25	10.4926	211.4835
wild-type	3	1	3	26	18.1492	144.1425
wild-type	3	1	3	27	8.0227	259.1722
wild-type	3	1	3	28	6.9297	139.0555
wild-type	3	1	3	29	10.8572	174.0823
wild-type	3	1	4	1	21.7438	174.8964
wild-type	3	1	4	2	10.2077	164.6105
wild-type	3	1	4	3	19.7656	150.5808
wild-type	3	1	4	4	11.7077	233.0704
wild-type	3	1	4	5	11.8881	188.7580
wild-type	3	1	4	6	19.3223	217.7789
wild-type	3	1	4	7	19.5902	203.6793
wild-type	3	1	4	8	12.7730	180.7262
wild-type	3	1	4	9	11.0061	191.7647
wild-type	3	1	4	10	20.6989	133.9120
wild-type	3	1	4	11	12.5059	233.5715
wild-type	3	1	4	12	9.6881	170.0399
wild-type	3	1	4	13	4.9039	154.9678
wild-type	3	1	4	14	21.9375	96.6112
wild-type	3	1	4	15	10.6345	171.2604
wild-type	3	1	4	16	20.3564	228.0677
wild-type	3	1	4	17	11.5268	269.1937
wild-type	3	1	4	18	12.3596	101.8381
wild-type	3	1	4	19	7.3751	181.7692
wild-type	3	1	4	20	29.1894	237.8261
wild-type	3	1	4	21	17.7374	119.4460
wild-type	3	1	4	22	60.8716	52.2903
wild-type	3	1	4	23	20.3837	160.8693
wild-type	3	1	4	24	27.2479	27.5065
wild-type	3	2	1	1	40.5652	223.5803
wild-type	3	2	1	2	40.8190	184.6361
wild-type	3	2	1	3	10.1633	301.9002
wild-type	3	2	1	4	9.8224	298.2556
wild-type	3	2	1	5	55.0496	81.7957
wild-type	3	2	1	6	35.5251	301.3905
wild-type	3	2	1	7	42.9317	294.3442
wild-type	3	2	1	8	26.7914	354.2225
wild-type	3	2	1	9	16.6841	309.4171
wild-type	3	2	1	10	58.7266	73.2748
wild-type	3	2	1	11	18.3292	178.6031
wild-type	3	2	1	12	15.1709	284.2008
wild-type	3	2	1	13	19.3143	290.5009
wild-type	3	2	1	14	24.5875	318.6881
wild-type	3	2	1	15	24.2877	264.8269
wild-type	3	2	2	1	13.1012	266.7073

wild-type	3	2	2	2	18.1421	220.7622
wild-type	3	2	2	3	12.4901	277.9384
wild-type	3	2	2	4	16.8688	347.8751
wild-type	3	2	2	5	11.6608	318.2125
wild-type	3	2	2	6	27.4669	280.3089
wild-type	3	2	2	7	16.9626	288.9929
wild-type	3	2	2	8	8.2233	251.6814
wild-type	3	2	2	9	21.4859	290.1636
wild-type	3	2	2	10	17.1324	343.3137
wild-type	3	2	2	11	12.2824	287.1260
wild-type	3	2	2	12	14.4580	248.9509
wild-type	3	2	2	13	17.9458	328.8646
wild-type	3	2	2	14	19.2950	273.3301
wild-type	3	2	2	15	15.5990	306.1590
wild-type	3	2	2	16	14.9141	250.0668
wild-type	3	2	2	17	9.6412	263.5767
wild-type	3	2	2	18	7.0907	203.7117
wild-type	3	2	2	19	7.6974	273.2313
wild-type	3	2	2	20	9.1427	347.3138
wild-type	3	2	2	21	17.7844	274.3928
wild-type	3	2	2	22	15.7027	245.4361
wild-type	3	2	2	23	16.9506	279.1324
wild-type	3	2	2	24	5.9713	323.6976
wild-type	3	2	2	25	21.6345	280.1931
wild-type	3	2	2	26	10.5017	278.2852
wild-type	3	2	2	27	16.3867	390.3240
wild-type	3	2	2	28	11.9304	218.8486
wild-type	3	2	2	29	9.3115	292.3531
wild-type	3	2	2	30	10.1780	268.8948
wild-type	3	2	2	31	20.1063	334.6273
wild-type	3	2	2	32	19.8856	242.8661
wild-type	3	2	2	33	12.5286	193.1929
wild-type	3	2	2	34	18.4482	255.6273
wild-type	3	2	2	35	8.6566	277.5592
wild-type	3	2	2	36	33.5644	335.3916
wild-type	3	2	2	37	22.6784	284.2451
wild-type	3	2	3	1	8.9400	289.5164
wild-type	3	2	3	2	8.4370	295.3437
wild-type	3	2	3	3	15.5597	266.3745
wild-type	3	2	3	4	9.5354	268.7287
wild-type	3	2	3	5	18.9135	205.2911
wild-type	3	2	3	6	10.7013	259.0259
wild-type	3	2	3	7	19.2130	261.0963
wild-type	3	2	3	8	37.0921	219.4567
wild-type	3	2	3	9	43.6515	241.9791
wild-type	3	2	3	10	20.6235	218.3149
wild-type	3	2	3	11	8.9738	323.6966
wild-type	3	2	3	12	7.3945	179.3047
wild-type	3	2	3	13	15.4515	346.5295
wild-type	3	2	3	14	9.7973	246.2384
wild-type	3	2	3	15	13.4746	331.2144
wild-type	3	2	3	16	20.6062	95.3165
wild-type	3	2	3	17	15.9569	224.6858
wild-type	3	2	3	18	8.7783	253.3577
wild-type	3	2	3	19	10.4127	303.8941
wild-type	3	2	3	20	23.6012	210.2371
wild-type	3	2	3	21	14.1717	244.5380
wild-type	3	2	3	22	23.3353	308.0250
wild-type	3	2	3	23	26.4981	328.1016
wild-type	3	2	3	24	9.6701	302.8334
wild-type	3	2	3	25	8.2867	209.8199
wild-type	3	2	3	26	4.8983	264.2300
wild-type	3	2	3	27	7.8474	277.8051
wild-type	3	2	3	28	11.3855	267.4711
wild-type	3	2	3	29	9.6986	265.7197
wild-type	3	2	3	30	13.6946	202.4964
wild-type	3	2	3	31	5.1506	245.0727
wild-type	3	2	3	32	13.8802	182.9026

wild-type	3	2	3	33	18.4936	125.4824
wild-type	3	2	3	34	9.3919	278.4152
wild-type	3	2	3	35	38.9427	183.7747
wild-type	3	2	3	36	24.6619	290.7807
wild-type	3	2	3	37	5.9970	335.9758
wild-type	3	2	3	38	22.8164	258.1850
wild-type	3	2	3	39	9.4843	215.7947
wild-type	3	2	4	1	7.3348	237.0219
wild-type	3	2	4	2	40.4654	244.6678
wild-type	3	2	4	3	10.1583	247.2358
wild-type	3	2	4	4	11.0033	241.8027
wild-type	3	2	4	5	7.8141	245.7678
wild-type	3	2	4	6	6.2505	193.8892
wild-type	3	2	4	7	10.7706	222.1145
wild-type	3	2	4	8	7.8864	263.1131
wild-type	3	2	4	9	5.6246	270.8065
wild-type	3	2	4	10	4.6190	278.0075
wild-type	3	2	4	11	13.3483	317.4588
wild-type	3	2	4	12	38.8579	206.7580
wild-type	3	2	4	13	7.4013	144.4509
wild-type	3	2	4	14	11.0166	287.1152
wild-type	3	2	4	15	91.0003	10.4492
wild-type	3	2	4	16	19.8129	65.6741
wild-type	3	2	4	17	30.2800	96.4043
wild-type	3	2	4	18	13.0956	245.2932
wild-type	3	2	4	19	19.1801	102.2875
wild-type	3	2	4	20	5.3020	258.3963
wild-type	3	3	1	1	29.8487	356.7044
wild-type	3	3	1	2	6.4379	314.2386
wild-type	3	3	1	3	16.7578	355.2402
wild-type	3	3	1	4	26.1204	180.2039
wild-type	3	3	1	5	21.6672	299.5768
wild-type	3	3	1	6	26.6159	304.7311
wild-type	3	3	1	7	11.6174	216.8791
wild-type	3	3	1	8	44.1921	103.9347
wild-type	3	3	1	9	109.2201	3.2709
wild-type	3	3	1	10	51.0535	54.4823
wild-type	3	3	1	11	30.9641	312.0777
wild-type	3	3	1	12	12.5990	323.1645
wild-type	3	3	2	1	12.4589	333.7055
wild-type	3	3	2	2	13.2633	249.4449
wild-type	3	3	2	3	22.9953	219.3981
wild-type	3	3	2	4	23.7859	253.2613
wild-type	3	3	2	5	19.3461	293.0119
wild-type	3	3	2	6	21.9817	260.7304
wild-type	3	3	2	7	23.9994	308.6295
wild-type	3	3	2	8	18.0352	237.8402
wild-type	3	3	2	9	10.2927	376.0804
wild-type	3	3	2	10	7.9163	296.6087
wild-type	3	3	2	11	11.2081	271.6094
wild-type	3	3	2	12	32.4451	260.2134
wild-type	3	3	2	13	13.1449	295.8588
wild-type	3	3	2	14	24.6798	372.4360
wild-type	3	3	2	15	11.8614	189.7092
wild-type	3	3	2	16	11.5441	179.8551
wild-type	3	3	2	17	24.4393	390.7155
wild-type	3	3	2	18	17.0906	171.8859
wild-type	3	3	2	19	93.5738	13.1190
wild-type	3	3	2	20	20.4687	345.9064
wild-type	3	3	2	21	14.6223	283.1602
wild-type	3	3	2	22	15.7972	379.9372
wild-type	3	3	2	23	15.4352	208.2310
wild-type	3	3	2	24	33.1244	266.9555
wild-type	3	3	2	25	15.2707	318.8174
wild-type	3	3	2	26	23.6410	268.1778
wild-type	3	3	2	27	17.0085	275.7403
wild-type	3	3	2	28	14.2230	268.5175
wild-type	3	3	2	29	22.4607	328.6203

wild-type	3	3	2	30	15.9493	210.2983
wild-type	3	3	2	31	5.1428	303.4222
wild-type	3	3	2	32	14.4125	302.7732
wild-type	3	3	2	33	26.7812	327.7728
wild-type	3	3	2	34	13.5842	269.0701
wild-type	3	3	2	35	42.6021	324.0944
wild-type	3	3	3	1	13.1930	308.9249
wild-type	3	3	3	2	25.3407	244.5130
wild-type	3	3	3	3	17.0685	280.9669
wild-type	3	3	3	4	14.6123	300.0165
wild-type	3	3	3	5	13.4505	303.7345
wild-type	3	3	3	6	7.7690	133.9377
wild-type	3	3	3	7	20.5028	216.1031
wild-type	3	3	3	8	20.8845	171.0505
wild-type	3	3	3	9	21.6416	152.5462
wild-type	3	3	3	10	7.8189	198.8418
wild-type	3	3	3	11	9.5538	212.9465
wild-type	3	3	3	12	16.9160	298.1984
wild-type	3	3	3	13	9.2216	292.8273
wild-type	3	3	3	14	10.6418	271.2968
wild-type	3	3	3	15	18.1306	142.6578
wild-type	3	3	3	16	20.7097	250.6733
wild-type	3	3	3	17	25.6841	400.3644
wild-type	3	3	3	18	9.2318	269.2655
wild-type	3	3	3	19	37.7067	348.9331
wild-type	3	3	3	20	17.1178	106.0849
wild-type	3	3	4	1	23.4090	191.3351
wild-type	3	3	4	2	9.6186	286.6571
wild-type	3	3	4	3	14.0743	257.4563
wild-type	3	3	4	4	31.8796	194.8357
wild-type	3	3	4	5	24.9178	191.2973
wild-type	3	3	4	6	29.3987	240.7353
wild-type	3	3	4	7	10.7470	165.2783
wild-type	3	3	4	8	19.1024	210.8798
wild-type	3	3	4	9	11.5165	207.9753
wild-type	3	3	4	10	17.5956	73.9065
wild-type	3	3	4	11	8.5963	261.1678
wild-type	3	3	4	12	13.2504	278.1228
wild-type	3	3	4	13	18.6345	163.8251
wild-type	3	3	4	14	6.1779	200.2911
wild-type	3	3	4	15	14.2703	188.6592
wild-type	3	3	4	16	9.1360	82.3220
wild-type	6	1	1	1	45.1115	254.4192
wild-type	6	1	1	2	52.2021	282.6557
wild-type	6	1	1	3	56.4456	256.7274
wild-type	6	1	1	4	11.5634	171.9082
wild-type	6	1	1	5	32.5298	174.7509
wild-type	6	1	1	6	24.3776	174.0672
wild-type	6	1	1	7	31.7649	221.5512
wild-type	6	1	2	1	37.1318	162.6266
wild-type	6	1	2	2	34.7615	178.4284
wild-type	6	1	2	3	30.6062	104.2595
wild-type	6	1	2	4	19.3158	171.3841
wild-type	6	1	2	5	32.1058	155.7724
wild-type	6	1	2	6	39.7628	137.1376
wild-type	6	1	2	7	37.3675	211.0391
wild-type	6	1	2	8	50.4725	86.0008
wild-type	6	1	2	9	14.6005	158.0526
wild-type	6	1	2	10	38.5736	216.3408
wild-type	6	1	2	11	52.8585	211.1561
wild-type	6	1	2	12	42.8327	171.8385
wild-type	6	1	2	13	58.3278	197.4848
wild-type	6	1	2	14	42.4155	213.5731
wild-type	6	1	2	15	40.0352	86.2695
wild-type	6	1	2	16	30.8342	92.6962
wild-type	6	1	3	1	44.2195	97.5903
wild-type	6	1	3	2	59.0980	108.0440
wild-type	6	1	3	3	18.5587	114.5394

wild-type	6	1	3	4	22.2454	81.6556
wild-type	6	1	3	5	29.5769	114.7522
wild-type	6	1	3	6	62.8231	77.1338
wild-type	6	1	3	7	48.1410	95.6155
wild-type	6	1	3	8	38.2919	122.2386
wild-type	6	1	3	9	44.7812	42.9853
wild-type	6	1	3	10	47.5026	258.1725
wild-type	6	1	3	11	55.8748	193.1797
wild-type	6	1	3	12	44.9471	134.8042
wild-type	6	1	3	13	40.9544	171.4555
wild-type	6	1	3	14	43.5605	80.3982
wild-type	6	1	3	15	32.2189	140.9409
wild-type	6	1	3	16	73.9433	131.9597
wild-type	6	1	3	17	35.3641	73.2702
wild-type	6	1	3	18	43.2566	212.3401
wild-type	6	1	3	19	38.4555	30.4523
wild-type	6	1	4	1	41.8782	128.8398
wild-type	6	1	4	2	37.0646	206.8289
wild-type	6	1	4	3	49.2767	172.1199
wild-type	6	1	4	4	20.4591	110.6495
wild-type	6	1	4	5	82.3356	157.8057
wild-type	6	1	4	6	69.9864	92.1323
wild-type	6	1	4	7	17.5163	94.1764
wild-type	6	1	4	8	47.0402	134.7816
wild-type	6	1	4	9	47.7112	214.0720
wild-type	6	1	4	10	78.8274	48.7742
wild-type	6	1	4	11	57.7196	142.6909
wild-type	6	1	4	12	84.4877	5.2941
wild-type	6	1	4	13	36.0762	128.0050
wild-type	6	1	4	14	70.0656	107.6234
wild-type	6	1	4	15	59.7695	51.8277
wild-type	6	1	4	16	52.3709	65.3606
wild-type	6	1	4	17	34.4630	61.3307
wild-type	6	1	4	18	28.1985	78.3262
wild-type	6	1	4	19	34.4613	137.5775
wild-type	6	1	4	20	66.9477	14.3922
wild-type	6	1	4	21	48.3730	145.9781
wild-type	6	1	4	22	69.9767	111.5122
wild-type	6	1	4	23	33.8969	163.7889
wild-type	6	1	4	24	20.7377	72.0867
wild-type	6	1	4	25	89.1763	5.5948
wild-type	6	1	4	26	80.9232	47.5338
wild-type	6	1	4	27	62.0700	46.4802
wild-type	6	2	1	1	50.1848	313.8742
wild-type	6	2	1	2	47.9416	235.9019
wild-type	6	2	1	3	46.2269	306.5067
wild-type	6	2	1	4	40.2315	259.6290
wild-type	6	2	1	5	8.5895	228.3732
wild-type	6	2	1	6	35.1763	239.8074
wild-type	6	2	1	7	45.7927	306.1643
wild-type	6	2	1	8	33.3974	101.7546
wild-type	6	2	1	9	28.6086	296.5931
wild-type	6	2	1	10	46.9456	292.7692
wild-type	6	2	1	11	29.4156	262.4033
wild-type	6	2	1	12	15.0405	276.3317
wild-type	6	2	1	13	51.7621	328.9463
wild-type	6	2	1	14	36.4580	316.6765
wild-type	6	2	1	15	41.9317	287.5232
wild-type	6	2	1	16	12.6340	264.2761
wild-type	6	2	1	17	7.8259	294.2191
wild-type	6	2	2	1	53.8187	223.6308
wild-type	6	2	2	2	14.3864	193.9732
wild-type	6	2	2	3	49.2273	174.4892
wild-type	6	2	2	4	8.0025	234.3639
wild-type	6	2	2	5	21.1726	257.2710
wild-type	6	2	2	6	37.9234	237.1621
wild-type	6	2	2	7	30.3463	265.2784
wild-type	6	2	2	8	28.1241	215.0606

wild-type	6	2	2	9	20.6822	183.2807
wild-type	6	2	2	10	39.3033	222.1210
wild-type	6	2	2	11	14.1569	171.0440
wild-type	6	2	2	12	26.9716	220.8308
wild-type	6	2	2	13	47.5122	260.5561
wild-type	6	2	2	14	28.4090	245.5965
wild-type	6	2	2	15	41.5266	215.3870
wild-type	6	2	2	16	22.8355	250.1147
wild-type	6	2	2	17	48.8727	217.5354
wild-type	6	2	2	18	33.0858	221.5290
wild-type	6	2	2	19	24.6281	188.3209
wild-type	6	2	2	20	19.6701	195.3477
wild-type	6	2	2	21	84.3716	14.4981
wild-type	6	2	2	22	45.7693	210.0007
wild-type	6	2	2	23	27.8439	198.5079
wild-type	6	2	2	24	30.9294	237.0697
wild-type	6	2	2	25	7.4703	212.5168
wild-type	6	2	2	26	18.9696	209.9066
wild-type	6	2	2	27	19.5567	246.8672
wild-type	6	2	2	28	29.6794	185.0255
wild-type	6	2	2	29	12.4189	197.6697
wild-type	6	2	2	30	35.7027	282.0324
wild-type	6	2	2	31	53.2329	12.1297
wild-type	6	2	2	32	30.5991	144.4707
wild-type	6	2	2	33	19.4540	260.8073
wild-type	6	2	2	34	29.5418	181.8946
wild-type	6	2	3	1	36.3033	168.4640
wild-type	6	2	3	2	39.2958	243.3133
wild-type	6	2	3	3	22.1022	165.1772
wild-type	6	2	3	4	31.5864	225.0597
wild-type	6	2	3	5	42.9934	186.2117
wild-type	6	2	3	6	24.1327	219.0288
wild-type	6	2	3	7	13.0059	138.9837
wild-type	6	2	3	8	39.4414	196.7015
wild-type	6	2	3	9	20.9777	171.0000
wild-type	6	2	3	10	19.0661	203.8213
wild-type	6	2	3	11	37.3214	182.2343
wild-type	6	2	3	12	21.4322	202.5679
wild-type	6	2	3	13	22.0933	215.2832
wild-type	6	2	3	14	30.8589	235.5895
wild-type	6	2	3	15	45.3862	175.8282
wild-type	6	2	3	16	32.6888	198.0054
wild-type	6	2	3	17	29.0656	233.6170
wild-type	6	2	3	18	22.7079	275.1914
wild-type	6	2	3	19	17.5310	177.0074
wild-type	6	2	3	20	19.8218	150.4276
wild-type	6	2	3	21	50.5327	256.0298
wild-type	6	2	3	22	13.3852	144.6223
wild-type	6	2	3	23	34.1951	232.0862
wild-type	6	2	3	24	37.8306	98.0358
wild-type	6	2	3	25	52.5045	275.5612
wild-type	6	2	3	26	36.5369	172.7286
wild-type	6	2	3	27	31.9754	198.2325
wild-type	6	2	3	28	39.7846	204.8375
wild-type	6	2	3	29	30.6222	159.9551
wild-type	6	2	3	30	28.4191	169.1388
wild-type	6	2	3	31	27.2746	288.4375
wild-type	6	2	3	32	47.3166	189.2328
wild-type	6	2	3	33	29.8546	193.3237
wild-type	6	2	3	34	24.6217	227.3060
wild-type	6	2	4	1	31.0040	168.5703
wild-type	6	2	4	2	21.6439	170.4112
wild-type	6	2	4	3	25.4678	176.4198
wild-type	6	2	4	4	28.2791	165.0357
wild-type	6	2	4	5	45.7374	198.3479
wild-type	6	2	4	6	38.4755	207.6664
wild-type	6	2	4	7	47.1862	155.4068
wild-type	6	2	4	8	66.6664	188.7184

wild-type	6	2	4	9	42.8760	192.0441
wild-type	6	2	4	10	28.9351	205.0040
wild-type	6	2	4	11	21.0628	183.9225
wild-type	6	2	4	12	47.0831	73.6519
wild-type	6	2	4	13	34.5223	274.2414
wild-type	6	2	4	14	25.3250	234.3338
wild-type	6	2	4	15	43.2952	145.4026
wild-type	6	2	4	16	37.4421	155.7673
wild-type	6	2	4	17	34.1856	185.3123
wild-type	6	2	4	18	43.3775	197.9218
wild-type	6	2	4	19	41.3320	208.3137
wild-type	6	2	4	20	16.1486	124.4530
wild-type	6	2	4	21	33.8990	161.9783
wild-type	6	2	4	22	45.2981	250.2025
wild-type	6	3	1	1	25.4167	267.1271
wild-type	6	3	1	2	28.1757	296.4370
wild-type	6	3	1	3	14.5513	313.9079
wild-type	6	3	1	4	59.1279	233.9746
wild-type	6	3	1	5	27.1090	201.3181
wild-type	6	3	1	6	55.1462	185.6623
wild-type	6	3	1	7	34.3793	245.3474
wild-type	6	3	1	8	46.4264	356.0540
wild-type	6	3	1	9	62.1155	253.0279
wild-type	6	3	1	10	31.7453	337.3955
wild-type	6	3	1	11	17.8840	341.2166
wild-type	6	3	1	12	31.4258	294.8932
wild-type	6	3	1	13	48.4178	315.9912
wild-type	6	3	1	14	71.6078	34.5402
wild-type	6	3	1	15	62.7400	259.5389
wild-type	6	3	2	1	29.1900	200.5996
wild-type	6	3	2	2	42.5500	239.6721
wild-type	6	3	2	3	25.2129	260.9504
wild-type	6	3	2	4	18.3342	201.0192
wild-type	6	3	2	5	24.4995	149.5902
wild-type	6	3	2	6	43.5780	176.4371
wild-type	6	3	2	7	28.3068	215.9820
wild-type	6	3	2	8	32.4293	221.5565
wild-type	6	3	2	9	40.5801	305.7886
wild-type	6	3	2	10	89.3746	151.7215
wild-type	6	3	2	11	101.5053	13.0961
wild-type	6	3	2	12	71.3391	112.9488
wild-type	6	3	2	13	10.9309	272.5719
wild-type	6	3	2	14	27.6352	245.5124
wild-type	6	3	2	15	61.9549	82.7954
wild-type	6	3	2	16	43.6592	186.9419
wild-type	6	3	2	17	24.9223	159.3707
wild-type	6	3	2	18	31.2210	357.0926
wild-type	6	3	2	19	113.3562	129.9254
wild-type	6	3	2	20	33.0076	206.4575
wild-type	6	3	2	21	24.6937	267.4303
wild-type	6	3	2	22	9.9380	308.1837
wild-type	6	3	2	23	48.2505	266.7089
wild-type	6	3	3	1	36.3716	220.6595
wild-type	6	3	3	2	35.6618	199.1676
wild-type	6	3	3	3	33.6020	175.7120
wild-type	6	3	3	4	17.0637	268.2278
wild-type	6	3	3	5	27.5456	161.6154
wild-type	6	3	3	6	42.8873	187.9861
wild-type	6	3	3	7	112.0897	241.5351
wild-type	6	3	3	8	32.0714	163.6094
wild-type	6	3	3	9	29.1970	214.2570
wild-type	6	3	3	10	35.0645	249.4590
wild-type	6	3	3	11	66.1250	147.4622
wild-type	6	3	3	12	21.8301	315.9595
wild-type	6	3	3	13	65.7445	30.6200
wild-type	6	3	3	14	46.2834	278.3897
wild-type	6	3	3	15	49.4253	187.7974
wild-type	6	3	3	16	30.2305	271.5086

wild-type	6	3	3	17	35.4143	195.3132
wild-type	6	3	3	18	106.2170	335.2761
wild-type	6	3	3	19	55.3940	29.8514
wild-type	6	3	3	20	117.1783	16.0478
wild-type	6	3	3	21	45.6711	222.5792
wild-type	6	3	3	22	47.2354	254.0163
wild-type	6	3	3	23	17.1492	185.1979
wild-type	6	3	3	24	36.2005	252.6370
wild-type	6	3	3	25	54.1317	243.1470
wild-type	6	3	3	26	33.2297	217.7928
wild-type	6	3	3	27	13.3806	222.5821
wild-type	6	3	3	28	104.0375	34.2800
wild-type	6	3	3	29	42.6819	191.2150
wild-type	6	3	3	30	152.6092	215.3004
wild-type	6	3	3	31	40.8197	87.8800
wild-type	6	3	3	32	66.6319	166.2488
wild-type	6	3	3	33	15.4107	213.4171
wild-type	6	3	4	1	30.5466	157.6544
wild-type	6	3	4	2	57.0324	161.9087
wild-type	6	3	4	3	34.0364	123.6484
wild-type	6	3	4	4	30.9065	135.5693
wild-type	6	3	4	5	26.1071	197.0848
wild-type	6	3	4	6	45.9161	224.9806
wild-type	6	3	4	7	104.5191	31.5285
wild-type	6	3	4	8	43.9386	219.8637
wild-type	6	3	4	9	24.4078	180.8299
wild-type	6	3	4	10	118.3203	60.6529
wild-type	6	3	4	11	34.9900	270.8399
wild-type	6	3	4	12	13.8789	176.1291
wild-type	6	3	4	13	22.2778	187.7429
wild-type	6	3	4	14	27.8166	230.0335
wild-type	6	3	4	15	23.4104	121.3179
wild-type	6	3	4	16	97.4485	19.2894
wild-type	6	3	4	17	17.8593	128.4067
wild-type	6	3	4	18	41.8050	203.9366
wild-type	6	3	4	19	65.9218	231.1017
wild-type	6	3	4	20	41.6764	238.6914
wild-type	6	3	4	21	29.3268	351.4041
wild-type	6	3	4	22	56.1191	96.1498
wild-type	6	3	4	23	24.3580	237.2577
wild-type	6	3	4	24	12.0906	249.4453
wild-type	6	3	4	25	40.1298	256.2451
wild-type	6	3	4	26	29.5252	185.3396
wild-type	6	3	4	27	25.3807	243.3561
wild-type	6	3	4	28	53.9269	218.5111
wild-type	6	3	4	29	37.0044	263.2101
wild-type	6	3	4	30	36.2392	131.7205
wild-type	6	3	4	31	63.0764	305.3657
wild-type	9	1	1	1	62.8054	223.9986
wild-type	9	1	1	2	64.5739	48.4988
wild-type	9	1	1	3	56.2991	199.5157
wild-type	9	1	1	4	16.0644	170.4691
wild-type	9	1	1	5	39.5115	111.6105
wild-type	9	1	1	6	76.5767	142.8740
wild-type	9	1	1	7	50.6276	150.4436
wild-type	9	1	2	1	42.2648	148.4050
wild-type	9	1	2	2	31.4946	91.6301
wild-type	9	1	2	3	31.0161	159.5843
wild-type	9	1	2	4	90.7069	12.3375
wild-type	9	1	2	5	53.1073	73.7776
wild-type	9	1	2	6	42.5528	87.5327
wild-type	9	1	2	7	38.4820	141.1078
wild-type	9	1	2	8	78.4570	30.8648
wild-type	9	1	2	9	55.5518	145.6950
wild-type	9	1	2	10	27.9745	148.2504
wild-type	9	1	2	11	54.0437	168.9435
wild-type	9	1	2	12	25.7586	73.7375
wild-type	9	1	2	13	40.1086	52.3022

wild-type	9	1	2	14	43.1248	85.7313
wild-type	9	1	2	15	37.6050	138.8563
wild-type	9	1	2	16	48.5085	208.1849
wild-type	9	1	2	17	70.2272	144.7109
wild-type	9	1	2	18	45.5219	104.5532
wild-type	9	1	2	19	52.1847	157.0816
wild-type	9	1	3	1	53.7831	77.0746
wild-type	9	1	3	2	37.2475	125.1526
wild-type	9	1	3	3	75.0986	103.8991
wild-type	9	1	3	4	75.2492	37.0567
wild-type	9	1	3	5	39.2364	144.4850
wild-type	9	1	3	6	63.2057	17.7784
wild-type	9	1	3	7	76.7342	54.6695
wild-type	9	1	3	8	53.7024	30.0759
wild-type	9	1	3	9	46.9531	28.2335
wild-type	9	1	3	10	123.8176	9.9025
wild-type	9	1	3	11	66.4428	43.2311
wild-type	9	1	3	12	33.1770	52.2905
wild-type	9	1	3	13	52.0106	79.7734
wild-type	9	1	3	14	55.1492	160.2048
wild-type	9	1	3	15	34.3598	201.2211
wild-type	9	1	3	16	117.3414	55.8947
wild-type	9	1	3	17	55.9513	155.9651
wild-type	9	1	3	18	56.9478	110.6527
wild-type	9	1	3	19	42.5035	99.1256
wild-type	9	1	4	1	51.6639	139.4028
wild-type	9	1	4	2	80.8789	139.1675
wild-type	9	1	4	3	38.0959	81.7852
wild-type	9	1	4	4	48.9567	24.4807
wild-type	9	1	4	5	50.7416	81.1985
wild-type	9	1	4	6	83.3401	7.0693
wild-type	9	1	4	7	53.1940	18.9397
wild-type	9	1	4	8	34.7001	81.4396
wild-type	9	1	4	9	79.2508	24.4701
wild-type	9	1	4	10	47.2322	106.6917
wild-type	9	1	4	11	67.1199	146.3900
wild-type	9	1	4	12	61.2408	30.2043
wild-type	9	1	4	13	55.0402	71.2453
wild-type	9	1	4	14	91.4934	10.0824
wild-type	9	1	4	15	87.5894	48.1734
wild-type	9	1	4	16	33.6068	105.3887
wild-type	9	1	4	17	35.2833	131.3518
wild-type	9	1	4	18	68.9837	125.2090
wild-type	9	1	4	19	38.8560	31.6278
wild-type	9	1	4	20	43.8394	67.9281
wild-type	9	1	4	21	35.6268	74.0354
wild-type	9	1	4	22	41.8520	194.7780
wild-type	9	2	1	1	6.7018	140.1908
wild-type	9	2	1	2	31.2506	139.6985
wild-type	9	2	1	3	77.9851	9.0790
wild-type	9	2	1	4	10.2401	252.8654
wild-type	9	2	1	5	72.2159	7.9646
wild-type	9	2	1	6	111.8366	7.9591
wild-type	9	2	1	7	87.6416	12.2654
wild-type	9	2	1	8	80.5331	24.0116
wild-type	9	2	1	9	21.7518	138.8985
wild-type	9	2	1	10	52.5564	47.9573
wild-type	9	2	2	1	44.5848	270.7041
wild-type	9	2	2	2	38.4814	199.4405
wild-type	9	2	2	3	22.7791	123.9379
wild-type	9	2	2	4	38.5581	189.4893
wild-type	9	2	2	5	39.6866	247.5748
wild-type	9	2	2	6	76.0607	88.0761
wild-type	9	2	2	7	54.4527	138.8719
wild-type	9	2	2	8	46.1207	23.1076
wild-type	9	2	2	9	35.8773	229.7940
wild-type	9	2	2	10	71.6712	50.1173
wild-type	9	2	2	11	47.9926	281.4033

wild-type	9	2	2	12	58.0360	253.7910
wild-type	9	2	2	13	91.9782	31.8580
wild-type	9	2	2	14	57.9081	241.4001
wild-type	9	2	2	15	69.1750	77.3591
wild-type	9	2	2	16	39.5462	256.0440
wild-type	9	2	2	17	57.3253	422.6282
wild-type	9	2	3	1	18.6998	179.1898
wild-type	9	2	3	2	90.8157	12.3085
wild-type	9	2	3	3	50.3183	114.3080
wild-type	9	2	3	4	30.1065	48.6457
wild-type	9	2	3	5	43.8230	195.1711
wild-type	9	2	3	6	77.8386	19.7009
wild-type	9	2	3	7	46.5904	182.9689
wild-type	9	2	3	8	45.2240	208.1213
wild-type	9	2	3	9	99.5194	9.8134
wild-type	9	2	3	10	45.4888	39.3883
wild-type	9	2	3	11	43.7572	225.5990
wild-type	9	2	3	12	68.1908	228.4448
wild-type	9	2	3	13	63.6208	145.7267
wild-type	9	2	3	14	84.8395	4.9420
wild-type	9	2	3	15	30.5959	46.3248
wild-type	9	2	3	16	34.0558	205.6044
wild-type	9	2	3	17	38.1446	98.4981
wild-type	9	2	3	18	32.4561	251.2496
wild-type	9	2	3	19	51.0798	173.3850
wild-type	9	2	3	20	65.9407	224.8642
wild-type	9	2	3	21	56.8789	187.2383
wild-type	9	2	3	22	15.5387	157.8661
wild-type	9	2	3	23	67.2241	113.7230
wild-type	9	2	4	1	90.1651	388.8882
wild-type	9	2	4	2	60.7729	232.4751
wild-type	9	2	4	3	47.5794	156.4330
wild-type	9	2	4	4	68.1985	113.6967
wild-type	9	2	4	5	79.2165	52.0340
wild-type	9	2	4	6	46.1927	62.0836
wild-type	9	2	4	7	114.7558	19.0814
wild-type	9	2	4	8	96.0563	6.4602
wild-type	9	2	4	9	84.9381	16.7066
wild-type	9	2	4	10	18.3783	12.8304
wild-type	9	2	4	11	72.7183	186.7350
wild-type	9	2	4	12	79.6711	57.2737
wild-type	9	2	4	13	59.0858	10.9455
wild-type	9	2	4	14	17.1797	144.8142
wild-type	9	2	4	15	109.7116	323.3337
wild-type	9	2	4	16	91.0247	4.9737
wild-type	9	2	4	17	67.9074	21.7909
wild-type	9	2	4	18	63.4527	59.4210
wild-type	9	2	4	19	61.3938	156.3339
wild-type	9	2	4	20	22.5265	146.1062
wild-type	9	2	4	21	61.7474	60.5640
wild-type	9	2	4	22	52.8433	75.5088
wild-type	9	3	1	1	31.0578	247.7934
wild-type	9	3	1	2	37.2432	222.7628
wild-type	9	3	1	3	47.1200	232.5350
wild-type	9	3	1	4	53.7389	121.6028
wild-type	9	3	1	5	30.2759	254.3763
wild-type	9	3	1	6	26.1992	291.3099
wild-type	9	3	1	7	60.2960	312.2194
wild-type	9	3	1	8	21.0130	283.4677
wild-type	9	3	1	9	49.6839	158.6050
wild-type	9	3	1	10	54.5994	221.6620
wild-type	9	3	1	11	94.9572	13.7418
wild-type	9	3	2	1	38.5828	227.0746
wild-type	9	3	2	2	64.9761	90.3572
wild-type	9	3	2	3	82.9864	385.9314
wild-type	9	3	2	4	47.1338	156.9339
wild-type	9	3	2	5	90.8834	62.5211
wild-type	9	3	2	6	65.3362	27.3854

wild-type	9	3	2	7	54.5234	222.4024
wild-type	9	3	2	8	29.4585	101.7727
wild-type	9	3	2	9	49.6537	125.3976
wild-type	9	3	2	10	54.1966	251.8236
wild-type	9	3	2	11	49.6016	141.2687
wild-type	9	3	2	12	50.4141	330.5116
wild-type	9	3	2	13	70.2123	51.8538
wild-type	9	3	2	14	81.4928	6.9182
wild-type	9	3	2	15	46.2876	198.5609
wild-type	9	3	2	16	66.6672	28.2646
wild-type	9	3	2	17	36.0492	42.4229
wild-type	9	3	2	18	37.7695	152.6592
wild-type	9	3	2	19	38.8010	203.8345
wild-type	9	3	2	20	35.5140	93.0615
wild-type	9	3	2	21	35.0362	283.4369
wild-type	9	3	2	22	67.1174	18.4940
wild-type	9	3	2	23	45.3443	192.5863
wild-type	9	3	2	24	65.9000	226.6912
wild-type	9	3	2	25	55.8932	111.7619
wild-type	9	3	3	1	42.5914	241.4874
wild-type	9	3	3	2	24.4466	175.1065
wild-type	9	3	3	3	73.8467	23.6260
wild-type	9	3	3	4	53.6302	195.6215
wild-type	9	3	3	5	62.8813	210.3004
wild-type	9	3	3	6	85.9740	38.0187
wild-type	9	3	3	7	24.9297	183.8167
wild-type	9	3	3	8	96.1256	10.0047
wild-type	9	3	3	9	69.0620	174.9949
wild-type	9	3	3	10	70.9575	12.6087
wild-type	9	3	3	11	62.4812	9.7426
wild-type	9	3	3	12	39.9716	172.7464
wild-type	9	3	3	13	75.4584	151.6810
wild-type	9	3	3	14	91.9936	4.4149
wild-type	9	3	3	15	53.9212	28.9886
wild-type	9	3	3	16	75.1189	62.4808
wild-type	9	3	3	17	59.6798	189.3267
wild-type	9	3	3	18	90.0075	120.9379
wild-type	9	3	3	19	42.1114	60.0885
wild-type	9	3	3	20	49.7311	199.1484
wild-type	9	3	3	21	52.5556	156.7614
wild-type	9	3	3	22	33.0113	200.0896
wild-type	9	3	3	23	81.4655	9.9051
wild-type	9	3	3	24	52.7062	263.1774
wild-type	9	3	4	1	35.0948	163.4992
wild-type	9	3	4	2	81.5650	58.6135
wild-type	9	3	4	3	32.9576	154.9485
wild-type	9	3	4	4	59.0794	115.5889
wild-type	9	3	4	5	49.1198	24.8172
wild-type	9	3	4	6	61.4725	51.4797
wild-type	9	3	4	7	77.4423	38.4350
wild-type	9	3	4	8	65.5132	39.9079
wild-type	9	3	4	9	84.3808	11.8559
wild-type	9	3	4	10	67.5702	48.4116
wild-type	9	3	4	11	46.2355	270.9503
wild-type	9	3	4	12	47.8468	199.3258
wild-type	9	3	4	13	38.2757	125.0578
wild-type	9	3	4	14	51.3271	192.4637
wild-type	9	3	4	15	44.3469	31.3021
wild-type	9	3	4	16	28.1599	251.2751
wild-type	9	3	4	17	51.0818	41.1294
wild-type	9	3	4	18	110.5231	7.4488
wild-type	9	3	4	19	55.0603	195.1660
wild-type	9	3	4	20	46.2242	21.0492
wild-type	9	3	4	21	62.2800	126.2444
wild-type	9	3	4	22	60.7073	55.6602
wild-type	9	3	4	23	78.9583	16.2698
wild-type	9	3	4	24	71.6633	84.6193
wild-type	9	3	4	25	68.5719	31.9007

wild-type	9	3	4	26	35.5256	36.4152
wild-type	9	3	4	27	62.0269	55.2400
wild-type	9	3	4	28	93.3682	4.8964
wild-type	9	3	4	29	34.4348	153.9467
wild-type	9	3	4	30	54.9063	176.3178
wild-type	9	3	4	31	66.1879	225.8125
inx-19/nsy-5	0	1	1	1	105.0436	148.7007
inx-19/nsy-5	0	1	1	2	53.9828	254.3867
inx-19/nsy-5	0	1	1	3	72.5783	199.2218
inx-19/nsy-5	0	1	1	4	94.6192	74.5665
inx-19/nsy-5	0	1	1	5	99.0543	101.1951
inx-19/nsy-5	0	1	1	6	138.4120	226.4250
inx-19/nsy-5	0	1	1	7	119.7810	246.0476
inx-19/nsy-5	0	1	1	8	110.2639	143.0837
inx-19/nsy-5	0	1	1	9	111.2276	206.6042
inx-19/nsy-5	0	1	1	10	117.8582	90.0083
inx-19/nsy-5	0	1	1	11	78.2273	190.5683
inx-19/nsy-5	0	1	2	1	23.2401	168.0929
inx-19/nsy-5	0	1	2	2	73.2286	105.7902
inx-19/nsy-5	0	1	2	3	75.3900	61.6942
inx-19/nsy-5	0	1	2	4	103.4852	93.1557
inx-19/nsy-5	0	1	2	5	85.7665	166.1727
inx-19/nsy-5	0	1	2	6	88.2495	2.2710
inx-19/nsy-5	0	1	2	7	81.6529	161.5577
inx-19/nsy-5	0	1	2	8	105.8148	113.3749
inx-19/nsy-5	0	1	2	9	95.5538	157.2585
inx-19/nsy-5	0	1	2	10	92.2984	162.7785
inx-19/nsy-5	0	1	2	11	134.1123	308.1352
inx-19/nsy-5	0	1	2	12	103.3048	116.7683
inx-19/nsy-5	0	1	2	13	112.8590	90.1785
inx-19/nsy-5	0	1	2	14	144.9474	103.3681
inx-19/nsy-5	0	1	2	15	82.0817	81.3136
inx-19/nsy-5	0	1	2	16	80.8788	106.5579
inx-19/nsy-5	0	1	2	17	100.9993	78.3474
inx-19/nsy-5	0	1	2	18	62.2658	153.5577
inx-19/nsy-5	0	1	2	19	48.7538	227.3090
inx-19/nsy-5	0	1	2	20	151.8227	111.7867
inx-19/nsy-5	0	1	2	21	130.1556	97.8271
inx-19/nsy-5	0	1	2	22	67.4949	74.8282
inx-19/nsy-5	0	1	2	23	98.2886	139.5513
inx-19/nsy-5	0	1	2	24	116.6366	119.5273
inx-19/nsy-5	0	1	2	25	94.8864	133.5331
inx-19/nsy-5	0	1	2	26	145.5288	201.2287
inx-19/nsy-5	0	1	2	27	106.0137	300.6549
inx-19/nsy-5	0	1	2	28	160.3197	291.1342
inx-19/nsy-5	0	1	2	29	61.2930	74.9954
inx-19/nsy-5	0	1	2	30	108.1164	74.4704
inx-19/nsy-5	0	1	3	1	61.4448	122.5734
inx-19/nsy-5	0	1	3	2	91.4819	273.5383
inx-19/nsy-5	0	1	3	3	82.1319	93.5570
inx-19/nsy-5	0	1	3	4	105.6390	123.9517
inx-19/nsy-5	0	1	3	5	94.5555	52.1844
inx-19/nsy-5	0	1	3	6	92.3091	146.2235
inx-19/nsy-5	0	1	3	7	72.5732	153.6174
inx-19/nsy-5	0	1	3	8	79.6212	36.7172
inx-19/nsy-5	0	1	3	9	82.5147	70.7662
inx-19/nsy-5	0	1	3	10	91.3183	92.7450
inx-19/nsy-5	0	1	3	11	101.8449	67.4214
inx-19/nsy-5	0	1	3	12	104.4178	68.3978
inx-19/nsy-5	0	1	3	13	111.8755	44.5516
inx-19/nsy-5	0	1	3	14	83.7616	44.8055
inx-19/nsy-5	0	1	3	15	78.2882	27.4771
inx-19/nsy-5	0	1	3	16	86.4625	49.2653
inx-19/nsy-5	0	1	3	17	80.9520	2.9401
inx-19/nsy-5	0	1	3	18	97.6099	73.6106
inx-19/nsy-5	0	1	3	19	73.1505	71.4132
inx-19/nsy-5	0	1	3	20	92.5580	107.7070
inx-19/nsy-5	0	1	3	21	103.5612	86.6842

inx-19/nsy-5	0	1	3	22	94.4594	170.3740
inx-19/nsy-5	0	1	3	23	117.1128	139.2324
inx-19/nsy-5	0	1	3	24	23.9967	92.6107
inx-19/nsy-5	0	1	3	25	87.0463	62.9498
inx-19/nsy-5	0	1	3	26	66.4650	43.5230
inx-19/nsy-5	0	1	4	1	77.9686	170.7512
inx-19/nsy-5	0	1	4	2	116.4503	127.2556
inx-19/nsy-5	0	1	4	3	108.2733	84.5543
inx-19/nsy-5	0	1	4	4	82.4921	64.7568
inx-19/nsy-5	0	1	4	5	83.3944	110.3469
inx-19/nsy-5	0	1	4	6	26.1379	185.6975
inx-19/nsy-5	0	1	4	7	68.9640	66.7929
inx-19/nsy-5	0	1	4	8	111.7009	193.4850
inx-19/nsy-5	0	1	4	9	58.1916	79.4800
inx-19/nsy-5	0	1	4	10	98.1349	78.4791
inx-19/nsy-5	0	1	4	11	97.1382	66.0482
inx-19/nsy-5	0	1	4	12	78.3418	30.6920
inx-19/nsy-5	0	1	4	13	34.2659	126.2885
inx-19/nsy-5	0	1	4	14	98.2122	47.8635
inx-19/nsy-5	0	1	4	15	91.4324	62.8048
inx-19/nsy-5	0	1	4	16	84.4751	138.0858
inx-19/nsy-5	0	1	4	17	102.3374	146.7127
inx-19/nsy-5	0	1	4	18	118.5500	156.4559
inx-19/nsy-5	0	1	4	19	96.7263	113.6499
inx-19/nsy-5	0	1	4	20	59.4962	78.9386
inx-19/nsy-5	0	1	4	21	87.7594	108.9489
inx-19/nsy-5	0	1	4	22	57.3877	54.3979
inx-19/nsy-5	0	1	4	23	93.8305	97.4844
inx-19/nsy-5	0	1	4	24	85.6422	48.8034
inx-19/nsy-5	0	1	4	25	73.4793	39.2222
inx-19/nsy-5	0	1	4	26	45.7247	50.7338
inx-19/nsy-5	0	1	4	27	127.5272	151.8254
inx-19/nsy-5	0	1	4	28	157.2335	117.8915
inx-19/nsy-5	0	2	1	1	78.0577	220.9958
inx-19/nsy-5	0	2	1	2	72.6954	221.4684
inx-19/nsy-5	0	2	1	3	150.4677	194.5111
inx-19/nsy-5	0	2	1	4	42.0769	327.0124
inx-19/nsy-5	0	2	1	5	45.4957	335.2311
inx-19/nsy-5	0	2	1	6	33.8606	282.5284
inx-19/nsy-5	0	2	1	7	112.8640	178.2939
inx-19/nsy-5	0	2	1	8	61.4636	209.9403
inx-19/nsy-5	0	2	1	9	16.2029	299.2061
inx-19/nsy-5	0	2	1	10	69.2578	231.9575
inx-19/nsy-5	0	2	1	11	28.2432	200.2598
inx-19/nsy-5	0	2	1	12	85.1949	241.7260
inx-19/nsy-5	0	2	1	13	84.6446	191.1846
inx-19/nsy-5	0	2	1	14	83.0135	161.1722
inx-19/nsy-5	0	2	1	15	98.4200	134.8532
inx-19/nsy-5	0	2	1	16	64.4992	149.3731
inx-19/nsy-5	0	2	1	17	81.7038	204.3107
inx-19/nsy-5	0	2	2	1	74.3261	175.2275
inx-19/nsy-5	0	2	2	2	94.8283	71.1953
inx-19/nsy-5	0	2	2	3	102.2134	239.0816
inx-19/nsy-5	0	2	2	4	93.2783	101.0461
inx-19/nsy-5	0	2	2	5	46.1947	201.5706
inx-19/nsy-5	0	2	2	6	68.2487	193.7623
inx-19/nsy-5	0	2	2	7	90.3591	237.0643
inx-19/nsy-5	0	2	2	8	75.7060	151.3671
inx-19/nsy-5	0	2	2	9	101.4665	78.1244
inx-19/nsy-5	0	2	2	10	100.5535	145.0862
inx-19/nsy-5	0	2	2	11	138.4706	214.8603
inx-19/nsy-5	0	2	2	12	30.0424	319.4828
inx-19/nsy-5	0	2	2	13	85.1892	116.9141
inx-19/nsy-5	0	2	2	14	97.4996	156.9656
inx-19/nsy-5	0	2	2	15	84.0370	74.5607
inx-19/nsy-5	0	2	2	16	74.7085	170.2373
inx-19/nsy-5	0	2	2	17	131.4532	299.0449
inx-19/nsy-5	0	2	2	18	55.3592	185.1037

inx-19/nsy-5	0	2	2	19	144.5598	47.5914
inx-19/nsy-5	0	2	2	20	166.8775	229.2370
inx-19/nsy-5	0	2	2	21	138.3319	221.5153
inx-19/nsy-5	0	2	2	22	113.0657	132.4362
inx-19/nsy-5	0	2	3	1	92.3541	131.7093
inx-19/nsy-5	0	2	3	2	70.7874	93.6184
inx-19/nsy-5	0	2	3	3	87.6890	103.0588
inx-19/nsy-5	0	2	3	4	79.3615	68.5247
inx-19/nsy-5	0	2	3	5	63.5952	114.6688
inx-19/nsy-5	0	2	3	6	49.0259	271.7298
inx-19/nsy-5	0	2	3	7	79.3109	35.9384
inx-19/nsy-5	0	2	3	8	82.7259	87.3447
inx-19/nsy-5	0	2	3	9	138.2517	164.2618
inx-19/nsy-5	0	2	3	10	157.9075	173.6132
inx-19/nsy-5	0	2	3	11	98.2580	75.2050
inx-19/nsy-5	0	2	3	12	92.4157	181.7980
inx-19/nsy-5	0	2	3	13	44.7224	86.7825
inx-19/nsy-5	0	2	3	14	43.8602	338.5787
inx-19/nsy-5	0	2	3	15	24.8096	51.9163
inx-19/nsy-5	0	2	3	16	148.8057	107.0458
inx-19/nsy-5	0	2	3	17	27.8197	204.7139
inx-19/nsy-5	0	2	3	18	63.3992	104.1406
inx-19/nsy-5	0	2	3	19	145.9189	202.7667
inx-19/nsy-5	0	2	4	1	123.2368	75.9502
inx-19/nsy-5	0	2	4	2	71.5854	73.3292
inx-19/nsy-5	0	2	4	3	94.1024	94.5024
inx-19/nsy-5	0	2	4	4	15.2466	176.1040
inx-19/nsy-5	0	2	4	5	84.2424	65.9358
inx-19/nsy-5	0	2	4	6	121.3285	105.1080
inx-19/nsy-5	0	2	4	7	55.3623	187.5043
inx-19/nsy-5	0	2	4	8	87.3807	65.0632
inx-19/nsy-5	0	2	4	9	34.1179	147.4414
inx-19/nsy-5	0	2	4	10	99.6156	185.4160
inx-19/nsy-5	0	2	4	11	88.5815	110.6699
inx-19/nsy-5	0	2	4	12	110.5104	50.4576
inx-19/nsy-5	0	2	4	13	145.2409	347.5545
inx-19/nsy-5	0	2	4	14	111.1713	72.1009
inx-19/nsy-5	0	2	4	15	127.9063	70.0023
inx-19/nsy-5	0	2	4	16	129.8034	94.4197
inx-19/nsy-5	0	2	4	17	16.3495	50.3223
inx-19/nsy-5	0	2	4	18	105.7913	142.8884
inx-19/nsy-5	0	2	4	19	84.2948	137.6074
inx-19/nsy-5	0	2	4	20	35.3588	233.5883
inx-19/nsy-5	0	2	4	21	94.2989	59.9545
inx-19/nsy-5	0	2	4	22	110.7108	40.5878
inx-19/nsy-5	0	2	4	23	59.8101	60.1016
inx-19/nsy-5	0	2	4	24	43.6877	245.9554
inx-19/nsy-5	0	2	4	25	154.2432	233.4083
inx-19/nsy-5	0	3	1	1	76.8799	237.5685
inx-19/nsy-5	0	3	1	2	47.1226	301.9547
inx-19/nsy-5	0	3	1	3	90.7087	342.1332
inx-19/nsy-5	0	3	1	4	91.0819	4.7552
inx-19/nsy-5	0	3	1	5	127.1399	341.8879
inx-19/nsy-5	0	3	1	6	116.8308	161.5464
inx-19/nsy-5	0	3	1	7	122.8749	238.7036
inx-19/nsy-5	0	3	1	8	129.5282	139.0684
inx-19/nsy-5	0	3	1	9	140.4578	285.1692
inx-19/nsy-5	0	3	1	10	73.6616	311.0879
inx-19/nsy-5	0	3	1	11	71.5729	278.5682
inx-19/nsy-5	0	3	1	12	147.4510	261.5084
inx-19/nsy-5	0	3	1	13	169.4921	251.1401
inx-19/nsy-5	0	3	1	14	87.3736	141.6267
inx-19/nsy-5	0	3	1	15	61.3289	73.6875
inx-19/nsy-5	0	3	1	16	146.6738	288.4187
inx-19/nsy-5	0	3	1	17	28.4737	419.0025
inx-19/nsy-5	0	3	1	18	14.3893	397.1099
inx-19/nsy-5	0	3	1	19	86.0698	201.0441
inx-19/nsy-5	0	3	1	20	62.9956	46.1464

inx-19/nsy-5	0	3	1	21	53.1957	248.4744
inx-19/nsy-5	0	3	1	22	43.9244	337.9829
inx-19/nsy-5	0	3	1	23	92.3061	104.3347
inx-19/nsy-5	0	3	1	24	106.0909	292.4139
inx-19/nsy-5	0	3	2	1	90.2916	155.4545
inx-19/nsy-5	0	3	2	2	37.4866	36.6302
inx-19/nsy-5	0	3	2	3	26.5798	264.3335
inx-19/nsy-5	0	3	2	4	131.8499	52.5305
inx-19/nsy-5	0	3	2	5	136.4531	89.1431
inx-19/nsy-5	0	3	2	6	88.1347	142.9579
inx-19/nsy-5	0	3	2	7	141.9591	255.4592
inx-19/nsy-5	0	3	2	8	120.2372	167.9978
inx-19/nsy-5	0	3	2	9	113.4396	132.9043
inx-19/nsy-5	0	3	2	10	130.0278	226.7797
inx-19/nsy-5	0	3	2	11	56.7178	207.8973
inx-19/nsy-5	0	3	2	12	108.2503	130.1657
inx-19/nsy-5	0	3	2	13	72.5617	33.0715
inx-19/nsy-5	0	3	2	14	90.5126	65.2139
inx-19/nsy-5	0	3	2	15	152.5198	395.2912
inx-19/nsy-5	0	3	2	16	51.5072	255.8526
inx-19/nsy-5	0	3	2	17	101.6251	332.2115
inx-19/nsy-5	0	3	2	18	130.4139	289.7860
inx-19/nsy-5	0	3	2	19	115.7290	165.3156
inx-19/nsy-5	0	3	2	20	99.6702	195.5380
inx-19/nsy-5	0	3	2	21	168.9742	303.5788
inx-19/nsy-5	0	3	2	22	92.2004	190.1225
inx-19/nsy-5	0	3	3	1	85.5880	94.8187
inx-19/nsy-5	0	3	3	2	77.9104	159.0447
inx-19/nsy-5	0	3	3	3	144.1880	164.2349
inx-19/nsy-5	0	3	3	4	88.7865	82.0587
inx-19/nsy-5	0	3	3	5	49.2313	28.8531
inx-19/nsy-5	0	3	3	6	66.2693	3.5660
inx-19/nsy-5	0	3	3	7	103.4295	124.4960
inx-19/nsy-5	0	3	3	8	105.8667	93.2257
inx-19/nsy-5	0	3	3	9	135.7773	267.6095
inx-19/nsy-5	0	3	3	10	142.5220	339.9339
inx-19/nsy-5	0	3	3	11	94.0544	102.7013
inx-19/nsy-5	0	3	3	12	79.3833	88.3152
inx-19/nsy-5	0	3	3	13	83.2146	170.4295
inx-19/nsy-5	0	3	3	14	42.1213	72.1279
inx-19/nsy-5	0	3	3	15	74.3779	218.6479
inx-19/nsy-5	0	3	3	16	52.2870	199.5663
inx-19/nsy-5	0	3	3	17	72.3202	24.3689
inx-19/nsy-5	0	3	3	18	33.9621	204.3277
inx-19/nsy-5	0	3	3	19	97.1577	84.2070
inx-19/nsy-5	0	3	3	20	80.2069	83.4870
inx-19/nsy-5	0	3	4	1	72.2656	318.1984
inx-19/nsy-5	0	3	4	2	163.3487	151.0180
inx-19/nsy-5	0	3	4	3	105.4927	86.6803
inx-19/nsy-5	0	3	4	4	71.2257	131.9983
inx-19/nsy-5	0	3	4	5	76.5902	3.7568
inx-19/nsy-5	0	3	4	6	51.2313	86.0483
inx-19/nsy-5	0	3	4	7	58.4781	97.7086
inx-19/nsy-5	0	3	4	8	59.4681	148.2529
inx-19/nsy-5	0	3	4	9	122.8737	39.5905
inx-19/nsy-5	0	3	4	10	36.2260	180.2252
inx-19/nsy-5	0	3	4	11	72.1254	68.4298
inx-19/nsy-5	0	3	4	12	38.9138	235.7565
inx-19/nsy-5	0	3	4	13	54.8972	203.6678
inx-19/nsy-5	0	3	4	14	75.9708	261.2024
inx-19/nsy-5	0	3	4	15	50.8319	132.5851
inx-19/nsy-5	0	3	4	16	59.1602	132.9705
inx-19/nsy-5	0	3	4	17	116.4989	102.4183
inx-19/nsy-5	0	3	4	18	129.7847	91.3507
inx-19/nsy-5	0	3	4	19	169.4804	305.9581
inx-19/nsy-5	1.5	1	1	1	77.2692	119.3902
inx-19/nsy-5	1.5	1	1	2	22.3241	118.7043
inx-19/nsy-5	1.5	1	1	3	35.1544	270.1328

inx-19/nsy-5	1.5	1	1	4	20.3983	205.1348
inx-19/nsy-5	1.5	1	1	5	162.2396	28.7142
inx-19/nsy-5	1.5	1	1	6	31.0739	289.4274
inx-19/nsy-5	1.5	1	1	7	64.3840	208.1967
inx-19/nsy-5	1.5	1	1	8	104.8244	87.9672
inx-19/nsy-5	1.5	1	1	9	97.1961	106.2322
inx-19/nsy-5	1.5	1	1	10	76.2485	98.6354
inx-19/nsy-5	1.5	1	2	1	97.1003	230.5609
inx-19/nsy-5	1.5	1	2	2	46.5619	148.4708
inx-19/nsy-5	1.5	1	2	3	28.6300	255.2703
inx-19/nsy-5	1.5	1	2	4	82.7087	81.9956
inx-19/nsy-5	1.5	1	2	5	48.2700	159.1743
inx-19/nsy-5	1.5	1	2	6	38.0729	68.0642
inx-19/nsy-5	1.5	1	2	7	121.6766	131.7378
inx-19/nsy-5	1.5	1	2	8	38.3781	202.2350
inx-19/nsy-5	1.5	1	2	9	87.1815	230.2301
inx-19/nsy-5	1.5	1	2	10	37.0829	210.7257
inx-19/nsy-5	1.5	1	2	11	115.4440	148.8018
inx-19/nsy-5	1.5	1	2	12	113.4117	114.4955
inx-19/nsy-5	1.5	1	2	13	112.2071	105.3837
inx-19/nsy-5	1.5	1	2	14	12.6730	244.3312
inx-19/nsy-5	1.5	1	2	15	47.8563	132.7463
inx-19/nsy-5	1.5	1	2	16	83.8495	111.0118
inx-19/nsy-5	1.5	1	2	17	54.7112	154.5195
inx-19/nsy-5	1.5	1	2	18	74.0442	218.2789
inx-19/nsy-5	1.5	1	2	19	115.2577	170.4737
inx-19/nsy-5	1.5	1	3	1	59.8881	115.8052
inx-19/nsy-5	1.5	1	3	2	21.4524	242.8388
inx-19/nsy-5	1.5	1	3	3	59.1010	282.6884
inx-19/nsy-5	1.5	1	3	4	58.4410	113.8927
inx-19/nsy-5	1.5	1	3	5	61.3730	88.9103
inx-19/nsy-5	1.5	1	3	6	30.9159	305.8541
inx-19/nsy-5	1.5	1	3	7	90.7832	42.0491
inx-19/nsy-5	1.5	1	3	8	78.5631	145.2388
inx-19/nsy-5	1.5	1	3	9	83.7257	83.4772
inx-19/nsy-5	1.5	1	3	10	49.1100	193.4464
inx-19/nsy-5	1.5	1	3	11	57.9222	90.1701
inx-19/nsy-5	1.5	1	3	12	70.3383	143.0312
inx-19/nsy-5	1.5	1	3	13	103.5729	124.6720
inx-19/nsy-5	1.5	1	3	14	38.4736	119.4964
inx-19/nsy-5	1.5	1	3	15	106.9687	36.2232
inx-19/nsy-5	1.5	1	3	16	48.7547	161.3026
inx-19/nsy-5	1.5	1	3	17	70.3130	68.6465
inx-19/nsy-5	1.5	1	3	18	32.6692	243.1901
inx-19/nsy-5	1.5	1	3	19	72.2847	105.8215
inx-19/nsy-5	1.5	1	3	20	90.7412	3.7314
inx-19/nsy-5	1.5	1	3	21	62.1447	243.0431
inx-19/nsy-5	1.5	1	3	22	29.0484	176.4152
inx-19/nsy-5	1.5	1	3	23	95.0731	49.3009
inx-19/nsy-5	1.5	1	3	24	138.2319	113.2970
inx-19/nsy-5	1.5	1	3	25	28.9087	170.9138
inx-19/nsy-5	1.5	1	3	26	94.1001	79.0256
inx-19/nsy-5	1.5	1	4	1	90.4832	103.6185
inx-19/nsy-5	1.5	1	4	2	78.4244	108.6698
inx-19/nsy-5	1.5	1	4	3	90.9217	126.2349
inx-19/nsy-5	1.5	1	4	4	70.9039	88.0211
inx-19/nsy-5	1.5	1	4	5	46.6407	174.0723
inx-19/nsy-5	1.5	1	4	6	101.4381	86.3438
inx-19/nsy-5	1.5	1	4	7	90.1115	195.3357
inx-19/nsy-5	1.5	1	4	8	143.4159	65.0554
inx-19/nsy-5	1.5	1	4	9	94.0042	200.7483
inx-19/nsy-5	1.5	1	4	10	86.3983	72.6166
inx-19/nsy-5	1.5	1	4	11	72.6146	125.8055
inx-19/nsy-5	1.5	1	4	12	42.1889	164.2063
inx-19/nsy-5	1.5	1	4	13	96.0598	57.2707
inx-19/nsy-5	1.5	1	4	14	23.8048	190.7475
inx-19/nsy-5	1.5	1	4	15	39.9924	83.5934
inx-19/nsy-5	1.5	1	4	16	73.0082	112.7002

inx-19/nsy-5	1.5	1	4	17	105.8280	71.0631
inx-19/nsy-5	1.5	1	4	18	88.4261	113.6772
inx-19/nsy-5	1.5	1	4	19	80.7954	4.5273
inx-19/nsy-5	1.5	1	4	20	115.2441	129.2325
inx-19/nsy-5	1.5	1	4	21	85.1129	83.4496
inx-19/nsy-5	1.5	1	4	22	105.4471	61.8998
inx-19/nsy-5	1.5	1	4	23	109.6939	262.3112
inx-19/nsy-5	1.5	2	1	1	20.7013	269.6314
inx-19/nsy-5	1.5	2	1	2	58.3505	48.3155
inx-19/nsy-5	1.5	2	1	3	60.5275	94.9755
inx-19/nsy-5	1.5	2	1	4	46.2592	219.8406
inx-19/nsy-5	1.5	2	1	5	10.2835	198.8641
inx-19/nsy-5	1.5	2	1	6	73.7733	215.1379
inx-19/nsy-5	1.5	2	1	7	100.3304	204.9141
inx-19/nsy-5	1.5	2	1	8	98.6800	52.8369
inx-19/nsy-5	1.5	2	1	9	93.6533	69.4512
inx-19/nsy-5	1.5	2	1	10	149.0274	218.6382
inx-19/nsy-5	1.5	2	1	11	54.9917	123.7769
inx-19/nsy-5	1.5	2	1	12	61.8525	236.1150
inx-19/nsy-5	1.5	2	1	13	79.5026	176.9307
inx-19/nsy-5	1.5	2	2	1	103.6454	98.9861
inx-19/nsy-5	1.5	2	2	2	60.0203	93.3832
inx-19/nsy-5	1.5	2	2	3	78.3722	195.8317
inx-19/nsy-5	1.5	2	2	4	126.8476	126.7585
inx-19/nsy-5	1.5	2	2	5	61.6334	158.7358
inx-19/nsy-5	1.5	2	2	6	58.9943	157.5722
inx-19/nsy-5	1.5	2	2	7	48.6625	51.0712
inx-19/nsy-5	1.5	2	2	8	33.3695	192.2710
inx-19/nsy-5	1.5	2	2	9	106.7687	21.1271
inx-19/nsy-5	1.5	2	2	10	91.2059	121.2691
inx-19/nsy-5	1.5	2	2	11	75.0557	223.4028
inx-19/nsy-5	1.5	2	2	12	71.6201	166.8520
inx-19/nsy-5	1.5	2	3	1	91.0148	184.3599
inx-19/nsy-5	1.5	2	3	2	95.9078	129.5379
inx-19/nsy-5	1.5	2	3	3	40.4172	124.6502
inx-19/nsy-5	1.5	2	3	4	79.2426	150.7088
inx-19/nsy-5	1.5	2	3	5	71.9997	51.9453
inx-19/nsy-5	1.5	2	3	6	81.5494	45.6319
inx-19/nsy-5	1.5	2	3	7	116.9736	10.9475
inx-19/nsy-5	1.5	2	3	8	76.3762	296.5440
inx-19/nsy-5	1.5	2	3	9	100.2938	84.6725
inx-19/nsy-5	1.5	2	4	1	105.7225	275.2180
inx-19/nsy-5	1.5	2	4	2	121.9903	264.4777
inx-19/nsy-5	1.5	2	4	3	59.0805	120.4748
inx-19/nsy-5	1.5	2	4	4	26.4976	213.6605
inx-19/nsy-5	1.5	2	4	5	108.1570	100.7447
inx-19/nsy-5	1.5	2	4	6	82.2289	94.9391
inx-19/nsy-5	1.5	2	4	7	119.1360	3.8774
inx-19/nsy-5	1.5	2	4	8	112.9513	123.0296
inx-19/nsy-5	1.5	2	4	9	118.3041	76.3904
inx-19/nsy-5	1.5	2	4	10	75.9807	102.4120
inx-19/nsy-5	1.5	2	4	11	114.3806	96.9260
inx-19/nsy-5	1.5	3	1	1	107.3414	165.3126
inx-19/nsy-5	1.5	3	1	2	81.0562	314.3661
inx-19/nsy-5	1.5	3	1	3	52.0688	328.4431
inx-19/nsy-5	1.5	3	1	4	86.6216	206.9515
inx-19/nsy-5	1.5	3	1	5	125.4136	132.7158
inx-19/nsy-5	1.5	3	1	6	120.6609	309.6010
inx-19/nsy-5	1.5	3	1	7	50.9165	317.7916
inx-19/nsy-5	1.5	3	1	8	81.8526	162.1638
inx-19/nsy-5	1.5	3	1	9	71.3880	105.0695
inx-19/nsy-5	1.5	3	2	1	65.7102	310.3070
inx-19/nsy-5	1.5	3	2	2	139.9580	212.2436
inx-19/nsy-5	1.5	3	2	3	85.9872	108.1690
inx-19/nsy-5	1.5	3	2	4	52.1944	279.5923
inx-19/nsy-5	1.5	3	2	5	45.1426	289.8314
inx-19/nsy-5	1.5	3	2	6	62.2181	275.8046
inx-19/nsy-5	1.5	3	2	7	78.0428	294.8198

inx-19/nsy-5	1.5	3	2	8	82.7632	251.8230
inx-19/nsy-5	1.5	3	2	9	58.3740	210.2166
inx-19/nsy-5	1.5	3	2	10	36.0406	286.6272
inx-19/nsy-5	1.5	3	2	11	12.4826	362.9417
inx-19/nsy-5	1.5	3	2	12	77.4387	54.1670
inx-19/nsy-5	1.5	3	2	13	128.5037	55.2805
inx-19/nsy-5	1.5	3	2	14	37.9301	186.4169
inx-19/nsy-5	1.5	3	2	15	32.6346	305.8858
inx-19/nsy-5	1.5	3	2	16	120.6077	261.8183
inx-19/nsy-5	1.5	3	3	1	68.4332	62.4596
inx-19/nsy-5	1.5	3	3	2	55.4968	231.0870
inx-19/nsy-5	1.5	3	3	3	132.1625	252.2836
inx-19/nsy-5	1.5	3	3	4	81.0163	192.8358
inx-19/nsy-5	1.5	3	3	5	65.8759	164.9558
inx-19/nsy-5	1.5	3	3	6	69.3031	119.1146
inx-19/nsy-5	1.5	3	3	7	76.7338	333.2153
inx-19/nsy-5	1.5	3	3	8	56.4423	230.6397
inx-19/nsy-5	1.5	3	3	9	14.6247	255.6892
inx-19/nsy-5	1.5	3	3	10	116.7268	32.5648
inx-19/nsy-5	1.5	3	3	11	55.9656	225.9031
inx-19/nsy-5	1.5	3	3	12	86.9992	131.3733
inx-19/nsy-5	1.5	3	3	13	84.2044	147.1834
inx-19/nsy-5	1.5	3	3	14	141.1032	70.6682
inx-19/nsy-5	1.5	3	3	15	89.1961	230.8474
inx-19/nsy-5	1.5	3	3	16	93.9745	242.5049
inx-19/nsy-5	1.5	3	3	17	82.0423	18.9774
inx-19/nsy-5	1.5	3	3	18	113.2173	69.4222
inx-19/nsy-5	1.5	3	3	19	136.6042	66.5706
inx-19/nsy-5	1.5	3	3	20	136.4618	224.8276
inx-19/nsy-5	1.5	3	4	1	99.4268	123.2123
inx-19/nsy-5	1.5	3	4	2	72.3715	215.6444
inx-19/nsy-5	1.5	3	4	3	65.3758	284.6146
inx-19/nsy-5	1.5	3	4	4	56.7219	102.3964
inx-19/nsy-5	1.5	3	4	5	81.6298	216.1005
inx-19/nsy-5	1.5	3	4	6	31.3994	233.7711
inx-19/nsy-5	1.5	3	4	7	89.3940	93.2869
inx-19/nsy-5	1.5	3	4	8	29.1216	383.6581
inx-19/nsy-5	1.5	3	4	9	65.9713	342.4299
inx-19/nsy-5	1.5	3	4	10	36.2815	341.8296
inx-19/nsy-5	1.5	3	4	11	73.9297	98.2442
inx-19/nsy-5	1.5	3	4	12	39.5008	301.4968
inx-19/nsy-5	1.5	3	4	13	90.4402	197.7669
inx-19/nsy-5	1.5	3	4	14	89.1321	211.8516
inx-19/nsy-5	1.5	3	4	15	62.0901	317.2583
inx-19/nsy-5	1.5	3	4	16	34.7821	132.7208
inx-19/nsy-5	1.5	3	4	17	55.8068	135.7322
inx-19/nsy-5	1.5	3	4	18	48.6147	171.0547
inx-19/nsy-5	1.5	3	4	19	87.6491	46.7718
inx-19/nsy-5	1.5	3	4	20	86.0504	232.7040
inx-19/nsy-5	1.5	3	4	21	24.7181	366.8477
inx-19/nsy-5	1.5	3	4	22	56.1425	169.6082
inx-19/nsy-5	1.5	3	4	23	112.5272	189.2936
inx-19/nsy-5	3	1	1	1	40.5229	286.8462
inx-19/nsy-5	3	1	1	2	33.8898	328.4642
inx-19/nsy-5	3	1	1	3	13.6317	317.0776
inx-19/nsy-5	3	1	1	4	29.5057	350.7694
inx-19/nsy-5	3	1	1	5	90.4818	93.5893
inx-19/nsy-5	3	1	1	6	41.1952	232.2848
inx-19/nsy-5	3	1	1	7	98.7899	14.1115
inx-19/nsy-5	3	1	1	8	57.6509	302.1322
inx-19/nsy-5	3	1	1	9	92.8039	163.2299
inx-19/nsy-5	3	1	1	10	10.5845	302.7687
inx-19/nsy-5	3	1	1	11	89.0006	174.6135
inx-19/nsy-5	3	1	1	12	91.0218	155.0178
inx-19/nsy-5	3	1	1	13	108.9649	142.6464
inx-19/nsy-5	3	1	2	1	70.6373	178.2953
inx-19/nsy-5	3	1	2	2	80.4030	139.9739
inx-19/nsy-5	3	1	2	3	78.3348	105.2158

inx-19/nsy-5	3	1	2	4	78.5350	138.3083
inx-19/nsy-5	3	1	2	5	106.5588	59.3263
inx-19/nsy-5	3	1	2	6	36.0186	169.7420
inx-19/nsy-5	3	1	2	7	79.8571	176.2474
inx-19/nsy-5	3	1	2	8	68.7147	95.9732
inx-19/nsy-5	3	1	2	9	111.9465	140.4822
inx-19/nsy-5	3	1	2	10	161.4102	86.4891
inx-19/nsy-5	3	1	2	11	121.3048	28.5531
inx-19/nsy-5	3	1	2	12	74.1758	94.1669
inx-19/nsy-5	3	1	2	13	49.8705	94.8793
inx-19/nsy-5	3	1	2	14	88.1597	179.5216
inx-19/nsy-5	3	1	2	15	52.9344	195.8967
inx-19/nsy-5	3	1	2	16	21.9302	323.5487
inx-19/nsy-5	3	1	2	17	95.2808	2.7933
inx-19/nsy-5	3	1	2	18	132.8037	60.7297
inx-19/nsy-5	3	1	2	19	80.6037	150.7011
inx-19/nsy-5	3	1	2	20	132.4385	72.5548
inx-19/nsy-5	3	1	2	21	58.5657	64.7945
inx-19/nsy-5	3	1	3	1	80.7071	130.4797
inx-19/nsy-5	3	1	3	2	75.4909	227.0427
inx-19/nsy-5	3	1	3	3	100.3222	73.6841
inx-19/nsy-5	3	1	3	4	99.8670	128.3981
inx-19/nsy-5	3	1	3	5	41.3615	320.7488
inx-19/nsy-5	3	1	3	6	31.3668	227.4322
inx-19/nsy-5	3	1	3	7	93.8972	8.4036
inx-19/nsy-5	3	1	3	8	11.1692	79.5821
inx-19/nsy-5	3	1	3	9	103.1493	60.4583
inx-19/nsy-5	3	1	3	10	90.4408	2.5822
inx-19/nsy-5	3	1	3	11	70.5441	53.2083
inx-19/nsy-5	3	1	3	12	48.0311	203.8064
inx-19/nsy-5	3	1	3	13	101.1081	3.9931
inx-19/nsy-5	3	1	3	14	30.0093	282.0127
inx-19/nsy-5	3	1	3	15	54.5125	243.3658
inx-19/nsy-5	3	1	3	16	127.8848	68.8108
inx-19/nsy-5	3	1	3	17	34.9631	151.5185
inx-19/nsy-5	3	1	3	18	72.4252	128.8581
inx-19/nsy-5	3	1	3	19	100.7184	69.2081
inx-19/nsy-5	3	1	4	1	75.9014	175.4591
inx-19/nsy-5	3	1	4	2	68.4695	152.9841
inx-19/nsy-5	3	1	4	3	100.8157	43.4568
inx-19/nsy-5	3	1	4	4	63.0930	116.8327
inx-19/nsy-5	3	1	4	5	60.7173	169.0347
inx-19/nsy-5	3	1	4	6	62.7999	166.5549
inx-19/nsy-5	3	1	4	7	89.6678	120.2309
inx-19/nsy-5	3	1	4	8	87.9570	33.0973
inx-19/nsy-5	3	1	4	9	54.4500	168.4796
inx-19/nsy-5	3	1	4	10	19.5899	293.8138
inx-19/nsy-5	3	1	4	11	42.2787	190.7669
inx-19/nsy-5	3	1	4	12	113.4620	55.7315
inx-19/nsy-5	3	1	4	13	65.8121	178.1191
inx-19/nsy-5	3	1	4	14	63.4953	197.7460
inx-19/nsy-5	3	1	4	15	70.8731	91.5484
inx-19/nsy-5	3	1	4	16	92.2337	153.2454
inx-19/nsy-5	3	1	4	17	80.5817	125.1396
inx-19/nsy-5	3	1	4	18	16.8343	327.9959
inx-19/nsy-5	3	1	4	19	5.6017	291.6268
inx-19/nsy-5	3	1	4	20	89.4107	91.4084
inx-19/nsy-5	3	1	4	21	80.4128	35.8166
inx-19/nsy-5	3	1	4	22	42.4453	201.9465
inx-19/nsy-5	3	1	4	23	88.4730	73.2693
inx-19/nsy-5	3	1	4	24	167.0623	65.2590
inx-19/nsy-5	3	1	4	25	110.3022	59.2481
inx-19/nsy-5	3	1	4	26	57.0297	124.9357
inx-19/nsy-5	3	1	4	27	108.8783	119.0331
inx-19/nsy-5	3	1	4	28	93.3466	13.7251
inx-19/nsy-5	3	1	4	29	73.5682	120.8383
inx-19/nsy-5	3	1	4	30	92.6101	1.7865
inx-19/nsy-5	3	1	4	31	48.7374	14.8362

inx-19/nsy-5	3	1	4	32	115.4015	49.0252
inx-19/nsy-5	3	2	1	1	51.1415	178.5797
inx-19/nsy-5	3	2	1	2	93.8950	264.8848
inx-19/nsy-5	3	2	1	3	8.2560	375.9864
inx-19/nsy-5	3	2	1	4	18.7760	58.9462
inx-19/nsy-5	3	2	1	5	126.9481	157.1567
inx-19/nsy-5	3	2	1	6	94.2233	28.8556
inx-19/nsy-5	3	2	1	7	87.9612	5.4302
inx-19/nsy-5	3	2	1	8	87.5730	36.0640
inx-19/nsy-5	3	2	1	9	60.7966	347.2819
inx-19/nsy-5	3	2	1	10	22.6045	275.0888
inx-19/nsy-5	3	2	1	11	78.7834	80.9177
inx-19/nsy-5	3	2	1	12	25.2700	339.9801
inx-19/nsy-5	3	2	1	13	39.9649	307.9190
inx-19/nsy-5	3	2	1	14	39.0470	233.3844
inx-19/nsy-5	3	2	1	15	57.8339	129.9827
inx-19/nsy-5	3	2	2	1	39.2449	376.1691
inx-19/nsy-5	3	2	2	2	78.9228	173.0218
inx-19/nsy-5	3	2	2	3	50.3113	87.7933
inx-19/nsy-5	3	2	2	4	63.3645	215.1059
inx-19/nsy-5	3	2	2	5	28.7296	47.7736
inx-19/nsy-5	3	2	2	6	52.7458	191.3182
inx-19/nsy-5	3	2	2	7	75.5046	129.4881
inx-19/nsy-5	3	2	2	8	68.4836	132.5523
inx-19/nsy-5	3	2	2	9	75.7068	31.8646
inx-19/nsy-5	3	2	2	10	92.5027	76.8467
inx-19/nsy-5	3	2	2	11	141.6056	33.4108
inx-19/nsy-5	3	2	2	12	78.0139	142.7785
inx-19/nsy-5	3	2	2	13	84.2097	96.4824
inx-19/nsy-5	3	2	2	14	65.8853	226.0816
inx-19/nsy-5	3	2	2	15	67.8416	145.1911
inx-19/nsy-5	3	2	2	16	89.9025	145.0824
inx-19/nsy-5	3	2	2	17	105.2824	83.3954
inx-19/nsy-5	3	2	2	18	104.8321	38.9284
inx-19/nsy-5	3	2	2	19	70.8049	199.7895
inx-19/nsy-5	3	2	3	1	90.9303	93.6756
inx-19/nsy-5	3	2	3	2	82.1255	263.1233
inx-19/nsy-5	3	2	3	3	24.8820	261.2819
inx-19/nsy-5	3	2	3	4	78.6191	107.8637
inx-19/nsy-5	3	2	3	5	27.8051	369.0413
inx-19/nsy-5	3	2	3	6	87.8709	64.8045
inx-19/nsy-5	3	2	3	7	25.6194	319.1054
inx-19/nsy-5	3	2	3	8	67.2419	60.9210
inx-19/nsy-5	3	2	3	9	22.4695	330.2089
inx-19/nsy-5	3	2	3	10	84.1725	226.8048
inx-19/nsy-5	3	2	3	11	95.0669	2.7386
inx-19/nsy-5	3	2	3	12	93.9651	55.2262
inx-19/nsy-5	3	2	3	13	88.6215	142.2885
inx-19/nsy-5	3	2	3	14	115.0099	68.3364
inx-19/nsy-5	3	2	3	15	76.5450	268.1510
inx-19/nsy-5	3	2	3	16	155.2360	67.0082
inx-19/nsy-5	3	2	4	1	87.7486	101.2406
inx-19/nsy-5	3	2	4	2	42.8679	240.9511
inx-19/nsy-5	3	2	4	3	91.5759	207.9772
inx-19/nsy-5	3	2	4	4	72.4350	89.2093
inx-19/nsy-5	3	2	4	5	99.0031	89.8515
inx-19/nsy-5	3	2	4	6	20.1449	129.9213
inx-19/nsy-5	3	2	4	7	96.8873	52.3926
inx-19/nsy-5	3	2	4	8	50.3085	20.9491
inx-19/nsy-5	3	2	4	9	132.7414	57.8644
inx-19/nsy-5	3	2	4	10	98.0535	103.9281
inx-19/nsy-5	3	2	4	11	37.1554	215.7915
inx-19/nsy-5	3	2	4	12	32.8492	267.7943
inx-19/nsy-5	3	3	1	1	83.1916	204.9857
inx-19/nsy-5	3	3	1	2	43.4271	336.6921
inx-19/nsy-5	3	3	1	3	24.5061	307.6145
inx-19/nsy-5	3	3	1	4	55.1802	343.7178
inx-19/nsy-5	3	3	1	5	115.5299	187.4983

inx-19/nsy-5	3	3	1	6	84.8506	311.2251
inx-19/nsy-5	3	3	1	7	59.3962	93.6448
inx-19/nsy-5	3	3	1	8	145.7801	34.4198
inx-19/nsy-5	3	3	1	9	20.1342	393.6324
inx-19/nsy-5	3	3	1	10	23.4671	354.6196
inx-19/nsy-5	3	3	1	11	77.7958	286.7427
inx-19/nsy-5	3	3	1	12	120.7767	114.0232
inx-19/nsy-5	3	3	2	1	104.7905	216.9704
inx-19/nsy-5	3	3	2	2	16.8361	257.4016
inx-19/nsy-5	3	3	2	3	131.2735	243.7588
inx-19/nsy-5	3	3	2	4	96.0712	177.0242
inx-19/nsy-5	3	3	2	5	28.6925	273.9598
inx-19/nsy-5	3	3	2	6	49.4248	151.6855
inx-19/nsy-5	3	3	2	7	92.7359	190.1297
inx-19/nsy-5	3	3	2	8	35.1860	249.8169
inx-19/nsy-5	3	3	2	9	36.3940	36.5233
inx-19/nsy-5	3	3	2	10	85.4062	5.7687
inx-19/nsy-5	3	3	2	11	26.1806	24.9141
inx-19/nsy-5	3	3	2	12	106.8554	193.0604
inx-19/nsy-5	3	3	2	13	91.9096	350.3804
inx-19/nsy-5	3	3	2	14	49.4763	354.1021
inx-19/nsy-5	3	3	2	15	103.8205	160.2932
inx-19/nsy-5	3	3	2	16	97.2331	271.3832
inx-19/nsy-5	3	3	2	17	32.1757	311.9470
inx-19/nsy-5	3	3	2	18	9.5970	266.0590
inx-19/nsy-5	3	3	2	19	69.3253	361.6490
inx-19/nsy-5	3	3	2	20	96.9619	173.7462
inx-19/nsy-5	3	3	2	21	158.1239	133.8143
inx-19/nsy-5	3	3	3	1	92.9969	188.7661
inx-19/nsy-5	3	3	3	2	54.8358	300.9389
inx-19/nsy-5	3	3	3	3	110.8341	73.8208
inx-19/nsy-5	3	3	3	4	127.3453	60.2770
inx-19/nsy-5	3	3	3	5	31.0752	26.3707
inx-19/nsy-5	3	3	3	6	100.0935	4.3912
inx-19/nsy-5	3	3	3	7	130.9603	40.1929
inx-19/nsy-5	3	3	3	8	91.7566	56.2705
inx-19/nsy-5	3	3	3	9	18.1750	337.0212
inx-19/nsy-5	3	3	3	10	102.9893	301.3082
inx-19/nsy-5	3	3	3	11	72.2301	105.1769
inx-19/nsy-5	3	3	3	12	55.4283	268.4183
inx-19/nsy-5	3	3	3	13	85.1442	73.8297
inx-19/nsy-5	3	3	3	14	89.7370	106.7303
inx-19/nsy-5	3	3	3	15	107.4019	113.4100
inx-19/nsy-5	3	3	3	16	113.3561	131.5257
inx-19/nsy-5	3	3	3	17	71.3543	150.4332
inx-19/nsy-5	3	3	4	1	127.7709	42.0770
inx-19/nsy-5	3	3	4	2	95.6418	169.4573
inx-19/nsy-5	3	3	4	3	118.4258	155.4071
inx-19/nsy-5	3	3	4	4	68.1600	359.2553
inx-19/nsy-5	3	3	4	5	83.3027	89.0435
inx-19/nsy-5	3	3	4	6	51.1225	19.6227
inx-19/nsy-5	3	3	4	7	54.6081	22.1342
inx-19/nsy-5	3	3	4	8	50.7467	121.3081
inx-19/nsy-5	3	3	4	9	98.0809	4.9931
inx-19/nsy-5	3	3	3	10	49.6634	150.8301
inx-19/nsy-5	3	3	4	11	91.5475	163.0066
inx-19/nsy-5	3	3	4	12	41.7789	268.6851
inx-19/nsy-5	3	3	4	13	41.7151	179.8500
inx-19/nsy-5	3	3	4	14	49.5468	78.0838
inx-19/nsy-5	3	3	4	15	154.6201	23.5064
inx-19/nsy-5	3	3	3	16	68.2420	103.1880
inx-19/nsy-5	3	3	3	17	11.0110	467.9630
inx-19/nsy-5	3	3	4	18	73.3698	3.7771
inx-19/nsy-5	3	3	4	19	88.4682	89.4745
inx-19/nsy-5	3	3	4	20	58.5498	36.8400
inx-19/nsy-5	6	1	1	1	52.3135	265.6880
inx-19/nsy-5	6	1	1	2	92.2160	91.9064
inx-19/nsy-5	6	1	1	3	17.3688	273.5763

inx-19/nsy-5	6	1	1	4	29.9947	138.3030
inx-19/nsy-5	6	1	1	5	10.8349	238.9270
inx-19/nsy-5	6	1	1	6	64.8987	138.5990
inx-19/nsy-5	6	1	1	7	84.0903	90.1744
inx-19/nsy-5	6	1	1	8	21.0643	210.4820
inx-19/nsy-5	6	1	1	9	69.0346	219.7107
inx-19/nsy-5	6	1	1	10	24.4972	330.0427
inx-19/nsy-5	6	1	1	11	60.0788	313.9542
inx-19/nsy-5	6	1	2	1	21.1907	225.4852
inx-19/nsy-5	6	1	2	2	71.6462	205.7920
inx-19/nsy-5	6	1	2	3	50.4607	299.5574
inx-19/nsy-5	6	1	2	4	19.8121	240.1039
inx-19/nsy-5	6	1	2	5	9.1043	336.8020
inx-19/nsy-5	6	1	2	6	33.9743	243.0202
inx-19/nsy-5	6	1	2	7	65.9185	304.4529
inx-19/nsy-5	6	1	2	8	84.6995	84.9325
inx-19/nsy-5	6	1	2	9	42.6526	313.5565
inx-19/nsy-5	6	1	2	10	110.0051	4.5511
inx-19/nsy-5	6	1	2	11	94.0075	144.2704
inx-19/nsy-5	6	1	2	12	63.8933	294.0914
inx-19/nsy-5	6	1	2	13	65.4067	283.7355
inx-19/nsy-5	6	1	2	14	57.3613	225.7262
inx-19/nsy-5	6	1	2	15	94.5781	33.6118
inx-19/nsy-5	6	1	2	16	93.1403	129.7113
inx-19/nsy-5	6	1	2	17	78.3498	108.8131
inx-19/nsy-5	6	1	2	18	50.0948	337.0389
inx-19/nsy-5	6	1	3	1	67.1611	245.4175
inx-19/nsy-5	6	1	3	2	35.6432	347.1290
inx-19/nsy-5	6	1	3	3	83.6422	216.0577
inx-19/nsy-5	6	1	3	4	76.2485	86.8999
inx-19/nsy-5	6	1	3	5	75.2832	80.9098
inx-19/nsy-5	6	1	3	6	34.5280	284.1540
inx-19/nsy-5	6	1	3	7	114.2673	118.3012
inx-19/nsy-5	6	1	3	8	85.8016	225.5506
inx-19/nsy-5	6	1	3	9	93.8955	177.1039
inx-19/nsy-5	6	1	3	10	46.4763	226.1857
inx-19/nsy-5	6	1	3	11	79.6010	277.2698
inx-19/nsy-5	6	1	3	12	88.6373	15.5848
inx-19/nsy-5	6	1	3	13	89.5564	18.7048
inx-19/nsy-5	6	1	3	14	51.7476	172.8801
inx-19/nsy-5	6	1	3	15	42.1111	185.4965
inx-19/nsy-5	6	1	3	16	39.6616	267.5444
inx-19/nsy-5	6	1	3	17	71.3741	149.5569
inx-19/nsy-5	6	1	3	18	47.7481	288.8483
inx-19/nsy-5	6	1	3	19	46.1725	329.3811
inx-19/nsy-5	6	1	3	20	18.5705	345.2991
inx-19/nsy-5	6	1	4	1	40.4182	262.9394
inx-19/nsy-5	6	1	4	2	47.9175	251.4673
inx-19/nsy-5	6	1	4	3	79.6248	166.3080
inx-19/nsy-5	6	1	4	4	72.3090	121.8911
inx-19/nsy-5	6	1	4	5	21.8998	239.0001
inx-19/nsy-5	6	1	4	6	118.3094	45.1061
inx-19/nsy-5	6	1	4	7	18.3020	245.4390
inx-19/nsy-5	6	1	4	8	97.8433	107.0701
inx-19/nsy-5	6	1	4	9	87.1498	73.4422
inx-19/nsy-5	6	1	4	10	18.9109	241.3406
inx-19/nsy-5	6	1	4	11	51.4348	83.5663
inx-19/nsy-5	6	1	4	12	33.2407	244.0915
inx-19/nsy-5	6	1	4	13	94.2083	124.9878
inx-19/nsy-5	6	1	4	14	64.9628	165.0057
inx-19/nsy-5	6	2	1	1	80.2405	214.6424
inx-19/nsy-5	6	2	1	2	53.1398	324.8198
inx-19/nsy-5	6	2	1	3	83.1483	7.3022
inx-19/nsy-5	6	2	1	4	67.2174	283.2719
inx-19/nsy-5	6	2	1	5	82.2774	5.8427
inx-19/nsy-5	6	2	1	6	105.3901	6.3058
inx-19/nsy-5	6	2	1	7	54.7538	301.1752
inx-19/nsy-5	6	2	1	8	16.3136	318.1637

inx-19/nsy-5	6	2	1	9	17.0058	307.7720
inx-19/nsy-5	6	2	1	10	43.6542	270.3399
inx-19/nsy-5	6	2	1	11	40.6510	142.9879
inx-19/nsy-5	6	2	1	12	61.2769	227.1674
inx-19/nsy-5	6	2	2	1	46.5787	270.8509
inx-19/nsy-5	6	2	2	2	39.5845	267.5236
inx-19/nsy-5	6	2	2	3	66.7073	137.4256
inx-19/nsy-5	6	2	2	4	77.4091	204.5192
inx-19/nsy-5	6	2	2	5	42.0717	306.0855
inx-19/nsy-5	6	2	2	6	57.3203	242.4344
inx-19/nsy-5	6	2	2	7	98.2421	38.3221
inx-19/nsy-5	6	2	2	8	113.5170	113.8429
inx-19/nsy-5	6	2	2	9	61.4806	222.5491
inx-19/nsy-5	6	2	2	10	18.2213	300.8054
inx-19/nsy-5	6	2	2	11	91.4769	5.6252
inx-19/nsy-5	6	2	2	12	105.7011	66.3203
inx-19/nsy-5	6	2	2	13	66.4455	259.8061
inx-19/nsy-5	6	2	2	14	29.4911	301.1223
inx-19/nsy-5	6	2	2	15	121.6118	69.9627
inx-19/nsy-5	6	2	2	16	60.7373	157.2226
inx-19/nsy-5	6	2	3	1	79.0722	124.4670
inx-19/nsy-5	6	2	3	2	21.8315	259.1496
inx-19/nsy-5	6	2	3	3	81.4530	165.1847
inx-19/nsy-5	6	2	3	4	76.8437	165.4748
inx-19/nsy-5	6	2	3	5	85.4580	76.8970
inx-19/nsy-5	6	2	3	6	51.3428	319.3597
inx-19/nsy-5	6	2	3	7	62.2136	299.1632
inx-19/nsy-5	6	2	2	8	81.8410	190.6180
inx-19/nsy-5	6	2	3	9	40.7757	255.9291
inx-19/nsy-5	6	2	3	10	46.4391	291.8460
inx-19/nsy-5	6	2	3	11	16.8472	201.1286
inx-19/nsy-5	6	2	3	12	82.0111	165.8391
inx-19/nsy-5	6	2	3	13	56.6536	353.3544
inx-19/nsy-5	6	2	3	14	99.8605	41.7784
inx-19/nsy-5	6	2	3	15	15.4330	135.6469
inx-19/nsy-5	6	2	3	16	79.5837	38.2957
inx-19/nsy-5	6	2	3	17	103.6522	15.0915
inx-19/nsy-5	6	2	3	18	38.0973	257.5388
inx-19/nsy-5	6	2	3	19	97.3907	7.9543
inx-19/nsy-5	6	2	4	1	57.0773	127.1957
inx-19/nsy-5	6	2	4	2	82.4195	79.4395
inx-19/nsy-5	6	2	4	3	45.3190	184.3797
inx-19/nsy-5	6	2	4	4	76.1471	99.1171
inx-19/nsy-5	6	2	4	5	89.6473	77.9954
inx-19/nsy-5	6	2	4	6	46.6699	245.9338
inx-19/nsy-5	6	2	4	7	41.6503	52.6545
inx-19/nsy-5	6	2	4	8	99.6453	6.0053
inx-19/nsy-5	6	2	4	9	57.9721	237.8882
inx-19/nsy-5	6	2	4	10	102.1432	180.4888
inx-19/nsy-5	6	2	4	11	81.0077	113.8139
inx-19/nsy-5	6	2	4	12	10.7147	289.5398
inx-19/nsy-5	6	3	1	1	68.5651	287.9695
inx-19/nsy-5	6	3	1	2	30.8332	343.4744
inx-19/nsy-5	6	3	1	3	10.4923	104.5748
inx-19/nsy-5	6	3	1	4	95.0125	147.6200
inx-19/nsy-5	6	3	1	5	50.2107	15.8669
inx-19/nsy-5	6	3	1	6	49.2189	208.1973
inx-19/nsy-5	6	3	1	7	74.6899	261.8025
inx-19/nsy-5	6	3	1	8	87.1178	186.5321
inx-19/nsy-5	6	3	1	9	24.1261	165.2459
inx-19/nsy-5	6	3	1	10	28.0977	194.4874
inx-19/nsy-5	6	3	1	11	53.4552	242.4041
inx-19/nsy-5	6	3	1	12	82.6113	37.1903
inx-19/nsy-5	6	3	1	13	13.0472	218.3480
inx-19/nsy-5	6	3	1	14	46.8756	345.4669
inx-19/nsy-5	6	3	1	15	100.9725	216.3864
inx-19/nsy-5	6	3	1	16	29.9498	238.9091
inx-19/nsy-5	6	3	1	17	13.9569	263.6766

inx-19/nsy-5	6	3	1	18	68.8810	80.1110
inx-19/nsy-5	6	3	2	1	60.7996	87.3770
inx-19/nsy-5	6	3	2	2	52.1396	207.9949
inx-19/nsy-5	6	3	2	3	91.5139	83.3521
inx-19/nsy-5	6	3	2	4	20.6589	217.5749
inx-19/nsy-5	6	3	2	5	101.3720	92.1467
inx-19/nsy-5	6	3	2	6	43.3355	18.0390
inx-19/nsy-5	6	3	2	7	89.3478	112.1166
inx-19/nsy-5	6	3	2	8	90.1642	159.5754
inx-19/nsy-5	6	3	2	9	11.9316	348.8937
inx-19/nsy-5	6	3	2	10	74.4440	77.2336
inx-19/nsy-5	6	3	2	11	29.6504	203.0341
inx-19/nsy-5	6	3	2	12	17.7566	225.3709
inx-19/nsy-5	6	3	2	13	35.0642	250.7637
inx-19/nsy-5	6	3	2	14	77.9832	195.5808
inx-19/nsy-5	6	3	2	15	15.7117	268.2518
inx-19/nsy-5	6	3	2	16	86.8300	50.7878
inx-19/nsy-5	6	3	2	17	53.3441	198.8561
inx-19/nsy-5	6	3	2	18	92.6218	46.6887
inx-19/nsy-5	6	3	2	19	57.1026	275.7998
inx-19/nsy-5	6	3	3	1	19.2114	177.0355
inx-19/nsy-5	6	3	3	2	77.1541	252.8725
inx-19/nsy-5	6	3	3	3	48.1335	293.8832
inx-19/nsy-5	6	3	3	4	21.4024	228.8227
inx-19/nsy-5	6	3	3	5	32.0129	261.8056
inx-19/nsy-5	6	3	3	6	67.9101	307.6814
inx-19/nsy-5	6	3	3	7	70.5496	208.2519
inx-19/nsy-5	6	3	3	8	84.9079	214.8348
inx-19/nsy-5	6	3	3	9	67.0592	202.9143
inx-19/nsy-5	6	3	3	10	106.0949	116.1222
inx-19/nsy-5	6	3	3	11	90.5064	37.9898
inx-19/nsy-5	6	3	3	12	54.5710	5.1426
inx-19/nsy-5	6	3	3	13	89.7989	6.2376
inx-19/nsy-5	6	3	3	14	87.3823	8.4954
inx-19/nsy-5	6	3	3	15	31.9018	160.8655
inx-19/nsy-5	6	3	3	16	48.7901	274.8046
inx-19/nsy-5	6	3	3	17	88.9311	73.9146
inx-19/nsy-5	6	3	3	18	80.1979	167.7606
inx-19/nsy-5	6	3	3	19	64.2197	44.3351
inx-19/nsy-5	6	3	3	20	77.3703	181.5334
inx-19/nsy-5	6	3	3	21	22.8528	228.2320
inx-19/nsy-5	6	3	3	22	50.2314	328.0255
inx-19/nsy-5	6	3	3	23	16.6540	276.4317
inx-19/nsy-5	6	3	3	24	68.8940	106.7427
inx-19/nsy-5	6	3	3	25	61.7567	177.5206
inx-19/nsy-5	6	3	3	26	48.2250	205.1795
inx-19/nsy-5	6	3	3	27	54.8566	264.4141
inx-19/nsy-5	6	3	3	28	48.7686	245.4206
inx-19/nsy-5	6	3	3	29	77.7832	230.1256
inx-19/nsy-5	6	3	4	1	77.3945	113.9886
inx-19/nsy-5	6	3	4	2	69.7441	162.4748
inx-19/nsy-5	6	3	4	3	79.4311	133.2534
inx-19/nsy-5	6	3	4	4	82.4592	102.2848
inx-19/nsy-5	6	3	4	5	39.5184	273.5760
inx-19/nsy-5	6	3	4	6	17.2700	152.8884
inx-19/nsy-5	6	3	4	7	88.0611	126.4144
inx-19/nsy-5	6	3	4	8	63.2433	307.9103
inx-19/nsy-5	6	3	4	9	133.0836	59.7080
inx-19/nsy-5	6	3	4	10	88.8640	118.4240
inx-19/nsy-5	6	3	4	11	60.6761	125.0091
inx-19/nsy-5	6	3	4	12	113.9225	90.0614
inx-19/nsy-5	6	3	4	13	85.5601	177.8402
inx-19/nsy-5	6	3	4	14	78.0911	5.0672
inx-19/nsy-5	6	3	4	15	78.3746	158.3669
inx-19/nsy-5	6	3	4	16	92.6639	154.5826
inx-19/nsy-5	6	3	4	17	110.4795	18.7902
inx-19/nsy-5	6	3	4	18	30.1832	275.3185
inx-19/nsy-5	6	3	4	19	36.8215	158.6833

inx-19/nsy-5	6	3	4	20	80.1959	106.4834
AWC::eat4	0	1	1	1	100.5036	84.8341
AWC::eat4	0	1	1	2	95.3806	58.0813
AWC::eat4	0	1	1	3	136.8917	91.8929
AWC::eat4	0	1	1	4	73.7679	236.0735
AWC::eat4	0	1	1	5	80.1805	60.4703
AWC::eat4	0	1	1	6	72.1395	112.2013
AWC::eat4	0	1	1	7	51.2504	120.8463
AWC::eat4	0	1	1	8	67.2069	8.2273
AWC::eat4	0	1	1	9	11.4457	232.2697
AWC::eat4	0	1	1	10	19.8034	35.4675
AWC::eat4	0	1	1	11	67.7735	178.1027
AWC::eat4	0	1	1	12	99.2784	208.0749
AWC::eat4	0	1	1	13	88.6998	55.2094
AWC::eat4	0	1	1	14	105.9361	26.1677
AWC::eat4	0	1	1	15	158.9758	236.2518
AWC::eat4	0	1	1	16	109.0905	170.2203
AWC::eat4	0	1	1	17	89.3910	2.2882
AWC::eat4	0	1	2	1	77.0894	100.0452
AWC::eat4	0	1	2	2	25.6412	61.7895
AWC::eat4	0	1	2	3	121.9397	18.3781
AWC::eat4	0	1	2	4	145.2648	202.4347
AWC::eat4	0	1	2	5	32.5203	43.2710
AWC::eat4	0	1	2	6	110.6868	56.9975
AWC::eat4	0	1	2	7	99.2571	88.4412
AWC::eat4	0	1	2	8	23.7625	82.7973
AWC::eat4	0	1	2	9	68.7399	75.3381
AWC::eat4	0	1	2	10	65.2159	79.1462
AWC::eat4	0	1	2	11	90.8449	97.6765
AWC::eat4	0	1	2	12	141.7913	101.3187
AWC::eat4	0	1	2	13	58.6457	117.7142
AWC::eat4	0	1	2	14	89.0420	98.4034
AWC::eat4	0	1	2	15	126.7819	85.0405
AWC::eat4	0	1	2	16	163.2861	116.4463
AWC::eat4	0	1	2	17	45.6149	115.5925
AWC::eat4	0	1	2	18	85.6476	20.9305
AWC::eat4	0	1	2	19	19.8893	89.8212
AWC::eat4	0	1	2	20	78.5534	61.0836
AWC::eat4	0	1	3	1	85.8991	121.8879
AWC::eat4	0	1	3	2	134.3762	77.2510
AWC::eat4	0	1	3	3	51.3207	50.5367
AWC::eat4	0	1	3	4	98.7100	83.6471
AWC::eat4	0	1	3	5	121.0323	81.4508
AWC::eat4	0	1	3	6	89.5548	2.9849
AWC::eat4	0	1	3	7	43.8783	65.6694
AWC::eat4	0	1	3	8	132.0437	124.1408
AWC::eat4	0	1	3	9	88.3937	37.1640
AWC::eat4	0	1	3	10	94.2379	2.7729
AWC::eat4	0	1	3	11	88.5268	38.7597
AWC::eat4	0	1	3	12	118.7923	12.9586
AWC::eat4	0	1	3	13	84.1666	183.6377
AWC::eat4	0	1	3	14	86.8576	77.2578
AWC::eat4	0	1	3	15	88.1090	65.8357
AWC::eat4	0	1	3	16	72.9698	71.2227
AWC::eat4	0	1	3	17	151.7372	101.3581
AWC::eat4	0	1	3	18	109.5696	69.6620
AWC::eat4	0	1	3	19	83.6302	107.6102
AWC::eat4	0	1	3	20	10.8637	156.8094
AWC::eat4	0	1	3	21	96.3785	19.4151
AWC::eat4	0	1	3	22	116.0956	95.5352
AWC::eat4	0	1	4	1	87.3880	40.8557
AWC::eat4	0	1	4	2	68.0135	58.4444
AWC::eat4	0	1	4	3	76.0127	109.3397
AWC::eat4	0	1	4	4	29.1424	185.8181
AWC::eat4	0	1	4	5	99.5641	3.0591
AWC::eat4	0	1	4	6	92.9046	111.8308
AWC::eat4	0	1	4	7	89.6503	16.4795
AWC::eat4	0	1	4	8	45.7159	78.0846

AWC::eat4	0	1	4	9	123.5639	48.3686
AWC::eat4	0	1	4	10	86.7074	70.6893
AWC::eat4	0	1	4	11	92.9296	222.3404
AWC::eat4	0	1	4	12	75.1674	59.7204
AWC::eat4	0	1	4	13	108.8576	150.7088
AWC::eat4	0	1	4	14	135.1787	50.1820
AWC::eat4	0	1	4	15	137.5776	36.5183
AWC::eat4	0	1	4	16	127.1183	83.0231
AWC::eat4	0	1	4	17	102.1378	112.4289
AWC::eat4	0	1	4	18	28.3121	130.3449
AWC::eat4	0	1	4	19	135.4047	63.9198
AWC::eat4	0	1	4	20	106.2284	38.1928
AWC::eat4	0	2	1	1	87.7631	88.6445
AWC::eat4	0	2	1	2	111.6893	68.2790
AWC::eat4	0	2	1	3	96.9321	129.7582
AWC::eat4	0	2	1	4	97.9189	106.0402
AWC::eat4	0	2	1	5	98.9313	140.3404
AWC::eat4	0	2	1	6	77.3291	133.7918
AWC::eat4	0	2	1	7	89.3525	107.6743
AWC::eat4	0	2	1	8	102.8465	119.0046
AWC::eat4	0	2	1	9	101.4405	69.8518
AWC::eat4	0	2	1	10	60.8890	121.7280
AWC::eat4	0	2	2	1	107.7085	108.2842
AWC::eat4	0	2	2	2	122.4408	158.6201
AWC::eat4	0	2	2	3	42.7857	62.0728
AWC::eat4	0	2	2	4	44.8842	55.8019
AWC::eat4	0	2	2	5	98.9431	81.4392
AWC::eat4	0	2	2	6	79.9351	106.7528
AWC::eat4	0	2	2	7	116.5316	94.7662
AWC::eat4	0	2	2	8	88.7343	133.7603
AWC::eat4	0	2	2	9	121.6536	116.9070
AWC::eat4	0	2	2	10	56.7159	155.0917
AWC::eat4	0	2	2	11	102.1065	95.7543
AWC::eat4	0	2	2	12	77.7454	118.6824
AWC::eat4	0	2	2	13	92.2194	137.8671
AWC::eat4	0	2	2	14	96.2805	97.0399
AWC::eat4	0	2	2	15	89.5887	92.5447
AWC::eat4	0	2	2	16	100.3185	214.9477
AWC::eat4	0	2	2	17	83.5427	181.6671
AWC::eat4	0	2	2	18	31.0065	105.2905
AWC::eat4	0	2	2	19	92.2554	63.6051
AWC::eat4	0	2	2	20	91.9292	102.7495
AWC::eat4	0	2	2	21	89.5651	157.1094
AWC::eat4	0	2	2	22	59.5359	99.2126
AWC::eat4	0	2	2	23	102.0604	51.5599
AWC::eat4	0	2	2	24	81.4742	127.9123
AWC::eat4	0	2	2	25	85.3617	129.2445
AWC::eat4	0	2	2	26	57.4292	175.3758
AWC::eat4	0	2	3	1	97.2419	69.4718
AWC::eat4	0	2	3	2	79.0068	97.6569
AWC::eat4	0	2	3	3	56.5029	57.9853
AWC::eat4	0	2	3	4	109.5233	165.1187
AWC::eat4	0	2	3	5	117.9724	57.4988
AWC::eat4	0	2	3	6	62.3362	133.4658
AWC::eat4	0	2	3	7	85.8663	123.9150
AWC::eat4	0	2	3	8	33.3084	104.4316
AWC::eat4	0	2	3	9	103.0559	76.3806
AWC::eat4	0	2	3	10	110.8329	98.7903
AWC::eat4	0	2	3	11	133.6857	103.6238
AWC::eat4	0	2	3	12	49.4323	174.1801
AWC::eat4	0	2	3	13	95.0059	102.1326
AWC::eat4	0	2	3	14	74.2799	77.2790
AWC::eat4	0	2	3	15	70.8273	91.4466
AWC::eat4	0	2	3	16	122.4154	162.2621
AWC::eat4	0	2	3	17	78.9317	90.5339
AWC::eat4	0	2	3	18	138.4711	205.4881
AWC::eat4	0	2	3	19	55.2135	67.0608
AWC::eat4	0	2	3	20	136.8936	122.1119

AWC::eat4	0	2	3	21	123.3111	78.6869
AWC::eat4	0	2	4	1	90.3875	166.0310
AWC::eat4	0	2	4	2	78.5818	153.3464
AWC::eat4	0	2	4	3	145.1257	134.6057
AWC::eat4	0	2	4	4	131.6600	127.1316
AWC::eat4	0	2	4	5	47.6728	158.4548
AWC::eat4	0	2	4	6	133.1636	128.5066
AWC::eat4	0	2	4	7	98.4594	116.3251
AWC::eat4	0	2	4	8	64.8863	127.8728
AWC::eat4	0	2	4	9	120.2388	117.8417
AWC::eat4	0	2	4	10	143.9819	81.2434
AWC::eat4	0	2	4	11	34.1472	186.6405
AWC::eat4	0	2	4	12	149.9926	59.0892
AWC::eat4	0	2	4	13	60.0183	110.0274
AWC::eat4	0	2	4	14	40.1842	140.3025
AWC::eat4	0	2	4	15	58.1142	116.6894
AWC::eat4	0	2	4	16	150.6145	162.8238
AWC::eat4	0	2	4	17	49.3333	140.9272
AWC::eat4	0	3	1	1	43.8297	102.3849
AWC::eat4	0	3	1	2	134.8377	93.1701
AWC::eat4	0	3	1	3	97.6583	12.0036
AWC::eat4	0	3	1	4	102.2717	57.5643
AWC::eat4	0	3	1	5	95.7490	23.4067
AWC::eat4	0	3	1	6	124.2349	65.8931
AWC::eat4	0	3	1	7	113.7655	19.2420
AWC::eat4	0	3	1	8	95.0724	77.0429
AWC::eat4	0	3	1	9	94.4869	76.5624
AWC::eat4	0	3	1	10	43.0689	23.0929
AWC::eat4	0	3	1	11	81.8983	127.6485
AWC::eat4	0	3	1	12	145.7454	97.7616
AWC::eat4	0	3	1	13	107.0795	56.0920
AWC::eat4	0	3	2	1	133.1600	95.7244
AWC::eat4	0	3	2	2	92.5198	31.0391
AWC::eat4	0	3	2	3	48.8764	67.0179
AWC::eat4	0	3	2	4	97.2262	41.2706
AWC::eat4	0	3	2	5	142.2281	45.9128
AWC::eat4	0	3	2	6	65.9205	71.8089
AWC::eat4	0	3	2	7	34.6497	75.7323
AWC::eat4	0	3	2	8	49.7990	75.4919
AWC::eat4	0	3	2	9	68.9028	78.7658
AWC::eat4	0	3	2	10	69.1385	82.4935
AWC::eat4	0	3	2	11	86.0568	67.9281
AWC::eat4	0	3	2	12	73.3356	121.0232
AWC::eat4	0	3	2	13	94.1229	37.4635
AWC::eat4	0	3	3	1	90.6657	94.8420
AWC::eat4	0	3	3	2	44.7566	30.2713
AWC::eat4	0	3	3	3	86.8172	140.3164
AWC::eat4	0	3	3	4	102.2616	52.9532
AWC::eat4	0	3	3	5	121.7668	69.8352
AWC::eat4	0	3	3	6	108.7516	90.8697
AWC::eat4	0	3	3	7	96.2538	56.2578
AWC::eat4	0	3	3	8	79.4797	101.1563
AWC::eat4	0	3	3	9	35.8934	70.6888
AWC::eat4	0	3	3	10	68.4530	61.6141
AWC::eat4	0	3	3	11	34.5239	99.9234
AWC::eat4	0	3	3	12	112.5008	81.4712
AWC::eat4	0	3	3	13	45.0010	10.9444
AWC::eat4	0	3	4	1	74.0169	102.5540
AWC::eat4	0	3	4	2	116.7661	28.5350
AWC::eat4	0	3	4	3	88.2698	71.6147
AWC::eat4	0	3	4	4	128.1261	97.9998
AWC::eat4	0	3	4	5	81.6692	118.5689
AWC::eat4	0	3	4	6	124.4009	34.3869
AWC::eat4	0	3	4	7	96.3433	71.7268
AWC::eat4	0	3	4	8	88.9262	50.1986
AWC::eat4	0	3	4	9	119.9907	110.5382
AWC::eat4	0	3	4	10	81.5906	58.3538
AWC::eat4	0	3	4	11	77.1183	192.7530

AWC::eat4	0	3	4	12	66.5526	22.5128
AWC::eat4	1.5	1	1	1	59.2632	273.8879
AWC::eat4	1.5	1	1	2	81.7942	99.4746
AWC::eat4	1.5	1	1	3	68.3786	175.6549
AWC::eat4	1.5	1	1	4	36.9456	196.5005
AWC::eat4	1.5	1	1	5	79.3111	155.7009
AWC::eat4	1.5	1	1	6	13.2742	248.0828
AWC::eat4	1.5	1	1	7	29.9879	262.1423
AWC::eat4	1.5	1	1	8	93.8234	9.7141
AWC::eat4	1.5	1	1	9	60.3333	143.2319
AWC::eat4	1.5	1	1	10	67.9286	185.6183
AWC::eat4	1.5	1	1	11	85.1732	44.0726
AWC::eat4	1.5	1	2	1	80.2040	102.7623
AWC::eat4	1.5	1	2	2	18.7300	196.6597
AWC::eat4	1.5	1	2	3	35.2293	278.1964
AWC::eat4	1.5	1	2	4	48.4770	138.0106
AWC::eat4	1.5	1	2	5	82.8490	282.6954
AWC::eat4	1.5	1	2	6	93.1111	118.5184
AWC::eat4	1.5	1	2	7	27.5791	234.3966
AWC::eat4	1.5	1	2	8	52.9506	118.7073
AWC::eat4	1.5	1	2	9	90.0228	12.8723
AWC::eat4	1.5	1	2	10	137.4835	39.1363
AWC::eat4	1.5	1	2	11	95.1332	54.1248
AWC::eat4	1.5	1	2	12	71.0515	82.9445
AWC::eat4	1.5	1	2	13	65.3193	91.6026
AWC::eat4	1.5	1	2	14	32.0729	76.3267
AWC::eat4	1.5	1	2	15	64.3378	122.0065
AWC::eat4	1.5	1	2	16	49.5870	116.3965
AWC::eat4	1.5	1	2	17	37.9976	252.2612
AWC::eat4	1.5	1	2	18	54.0773	230.9740
AWC::eat4	1.5	1	2	19	93.0037	73.0922
AWC::eat4	1.5	1	2	20	44.0848	163.8111
AWC::eat4	1.5	1	3	1	110.8913	328.2849
AWC::eat4	1.5	1	3	2	68.3708	139.0314
AWC::eat4	1.5	1	3	3	66.2803	100.9423
AWC::eat4	1.5	1	3	4	94.9990	54.0586
AWC::eat4	1.5	1	3	5	45.0071	205.2400
AWC::eat4	1.5	1	3	6	48.2188	54.5745
AWC::eat4	1.5	1	3	7	30.4081	242.8178
AWC::eat4	1.5	1	3	8	68.2178	63.1134
AWC::eat4	1.5	1	3	9	77.2855	140.2744
AWC::eat4	1.5	1	3	10	55.8258	85.7998
AWC::eat4	1.5	1	3	11	59.4074	99.3285
AWC::eat4	1.5	1	3	12	97.4789	50.5215
AWC::eat4	1.5	1	3	13	97.8204	39.6387
AWC::eat4	1.5	1	3	14	123.4197	24.3812
AWC::eat4	1.5	1	3	15	87.2494	96.1492
AWC::eat4	1.5	1	3	16	115.3384	19.7171
AWC::eat4	1.5	1	3	17	31.5269	178.0194
AWC::eat4	1.5	1	3	18	113.8721	213.9954
AWC::eat4	1.5	1	3	19	39.5683	261.7219
AWC::eat4	1.5	1	3	20	100.3415	94.7098
AWC::eat4	1.5	1	3	21	70.9452	116.8243
AWC::eat4	1.5	1	3	22	75.5788	61.3185
AWC::eat4	1.5	1	3	23	55.4774	75.9359
AWC::eat4	1.5	1	3	24	72.2212	155.5235
AWC::eat4	1.5	1	4	1	85.9113	169.7738
AWC::eat4	1.5	1	4	2	54.6286	244.4000
AWC::eat4	1.5	1	4	3	22.1626	253.1850
AWC::eat4	1.5	1	4	4	70.6050	198.4879
AWC::eat4	1.5	1	4	5	95.7693	89.5912
AWC::eat4	1.5	1	4	6	62.6586	133.8969
AWC::eat4	1.5	1	4	7	28.2586	306.5700
AWC::eat4	1.5	1	4	8	59.0501	45.8762
AWC::eat4	1.5	1	4	9	61.5128	152.2009
AWC::eat4	1.5	1	4	10	59.4124	166.7456
AWC::eat4	1.5	1	4	11	109.3070	108.8053
AWC::eat4	1.5	1	4	12	87.0315	112.3138

AWC::eat4	1.5	1	4	13	31.8836	116.9666
AWC::eat4	1.5	1	4	14	45.5993	113.2466
AWC::eat4	1.5	1	4	15	65.0227	105.8530
AWC::eat4	1.5	1	4	16	67.5581	200.2596
AWC::eat4	1.5	1	4	17	87.0580	50.0572
AWC::eat4	1.5	1	4	18	55.0176	93.3034
AWC::eat4	1.5	1	4	19	111.8273	63.7155
AWC::eat4	1.5	2	1	1	68.6891	144.8301
AWC::eat4	1.5	2	1	2	87.0443	56.7315
AWC::eat4	1.5	2	1	3	27.2951	113.7796
AWC::eat4	1.5	2	1	4	73.4343	84.9025
AWC::eat4	1.5	2	1	5	57.6237	116.3034
AWC::eat4	1.5	2	1	6	76.3435	38.6645
AWC::eat4	1.5	2	1	7	92.7347	147.9833
AWC::eat4	1.5	2	1	8	70.7152	120.3103
AWC::eat4	1.5	2	1	9	60.5885	169.8057
AWC::eat4	1.5	2	2	1	82.9046	95.5094
AWC::eat4	1.5	2	2	2	73.4476	146.4672
AWC::eat4	1.5	2	2	3	65.8045	123.6721
AWC::eat4	1.5	2	2	4	87.6962	362.6920
AWC::eat4	1.5	2	2	5	74.4612	104.9536
AWC::eat4	1.5	2	2	6	82.1259	55.4095
AWC::eat4	1.5	2	2	7	47.0533	147.0483
AWC::eat4	1.5	2	2	8	44.3143	281.1031
AWC::eat4	1.5	2	2	9	92.1958	107.5278
AWC::eat4	1.5	2	2	10	76.4475	94.0523
AWC::eat4	1.5	2	2	11	54.5233	144.9599
AWC::eat4	1.5	2	2	12	58.2127	216.9405
AWC::eat4	1.5	2	2	13	47.1903	130.3547
AWC::eat4	1.5	2	3	1	77.4460	83.7830
AWC::eat4	1.5	2	3	2	64.8820	132.3909
AWC::eat4	1.5	2	3	3	30.5337	118.4581
AWC::eat4	1.5	2	3	4	29.9386	248.7709
AWC::eat4	1.5	2	3	5	53.0625	179.5916
AWC::eat4	1.5	2	3	6	84.2232	68.2794
AWC::eat4	1.5	2	3	7	103.6190	71.2634
AWC::eat4	1.5	2	3	8	54.6984	73.4958
AWC::eat4	1.5	2	3	9	118.6966	59.3558
AWC::eat4	1.5	2	3	10	25.6607	337.1688
AWC::eat4	1.5	2	3	11	55.4582	258.5987
AWC::eat4	1.5	2	4	1	98.1758	107.8421
AWC::eat4	1.5	2	4	2	66.3277	120.9716
AWC::eat4	1.5	2	4	3	58.5113	101.5731
AWC::eat4	1.5	2	4	4	82.1308	164.2868
AWC::eat4	1.5	2	4	5	84.8473	85.8071
AWC::eat4	1.5	2	4	6	36.8036	59.3948
AWC::eat4	1.5	2	4	7	91.1646	87.2586
AWC::eat4	1.5	2	4	8	70.6703	128.6039
AWC::eat4	1.5	2	4	9	78.7388	24.4947
AWC::eat4	1.5	2	4	10	17.1642	184.0896
AWC::eat4	1.5	2	4	11	43.3862	149.0611
AWC::eat4	1.5	3	1	1	95.2963	66.1470
AWC::eat4	1.5	3	1	2	39.0938	136.8484
AWC::eat4	1.5	3	1	3	36.9443	26.1123
AWC::eat4	1.5	3	1	4	57.0148	121.7875
AWC::eat4	1.5	3	1	5	66.7651	14.9854
AWC::eat4	1.5	3	1	6	37.6910	67.5071
AWC::eat4	1.5	3	1	7	20.6303	159.7491
AWC::eat4	1.5	3	1	8	32.2595	184.7424
AWC::eat4	1.5	3	1	9	83.5052	75.8730
AWC::eat4	1.5	3	1	10	77.1381	36.9742
AWC::eat4	1.5	3	1	11	103.1210	2.1450
AWC::eat4	1.5	3	1	12	32.3687	77.5044
AWC::eat4	1.5	3	1	13	30.4848	206.6879
AWC::eat4	1.5	3	1	14	38.9606	181.5627
AWC::eat4	1.5	3	1	15	99.6003	14.7857
AWC::eat4	1.5	3	2	1	113.6765	36.1879
AWC::eat4	1.5	3	2	2	21.9346	255.1192

AWC::eat4	1.5	3	2	3	20.4414	129.3545
AWC::eat4	1.5	3	2	4	71.2193	69.7264
AWC::eat4	1.5	3	2	5	93.2765	105.0304
AWC::eat4	1.5	3	2	6	69.4289	42.3198
AWC::eat4	1.5	3	2	7	68.5568	101.3740
AWC::eat4	1.5	3	2	8	89.0993	128.5201
AWC::eat4	1.5	3	2	9	64.5519	68.6958
AWC::eat4	1.5	3	3	1	52.5283	173.0745
AWC::eat4	1.5	3	3	2	72.5157	90.2801
AWC::eat4	1.5	3	3	3	68.8802	35.3931
AWC::eat4	1.5	3	3	4	61.4998	169.0972
AWC::eat4	1.5	3	3	5	73.2547	72.3871
AWC::eat4	1.5	3	3	6	132.7681	147.3907
AWC::eat4	1.5	3	3	7	68.8625	84.6678
AWC::eat4	1.5	3	3	8	40.7789	157.1710
AWC::eat4	1.5	3	3	9	57.3931	36.3855
AWC::eat4	1.5	3	3	10	99.1892	33.7918
AWC::eat4	1.5	3	3	11	69.3207	86.8443
AWC::eat4	1.5	3	4	1	62.1003	117.8756
AWC::eat4	1.5	3	4	2	41.4520	113.3738
AWC::eat4	1.5	3	4	3	42.3928	104.9583
AWC::eat4	1.5	3	4	4	74.3107	105.3568
AWC::eat4	1.5	3	4	5	38.7825	142.6188
AWC::eat4	1.5	3	4	6	98.4687	53.6287
AWC::eat4	1.5	3	4	7	127.4710	44.6668
AWC::eat4	1.5	3	4	8	72.4177	106.8579
AWC::eat4	1.5	3	4	9	113.8255	40.6344
AWC::eat4	1.5	3	4	10	65.9671	46.2943
AWC::eat4	1.5	3	4	11	91.2042	47.9516
AWC::eat4	1.5	3	4	12	48.6380	40.9220
AWC::eat4	1.5	3	4	13	23.8420	324.9617
AWC::eat4	3	1	1	1	60.5785	343.5897
AWC::eat4	3	1	1	2	46.4017	345.8916
AWC::eat4	3	1	1	3	30.0494	346.7690
AWC::eat4	3	1	1	4	21.2623	297.2476
AWC::eat4	3	1	1	5	77.3759	274.5214
AWC::eat4	3	1	1	6	46.6619	270.7656
AWC::eat4	3	1	1	7	19.2392	300.3402
AWC::eat4	3	1	1	8	25.1595	358.2768
AWC::eat4	3	1	1	9	43.8109	264.7375
AWC::eat4	3	1	1	10	52.9848	295.8738
AWC::eat4	3	1	1	11	74.0371	276.0061
AWC::eat4	3	1	1	12	22.8536	295.3742
AWC::eat4	3	1	1	13	79.4047	81.1041
AWC::eat4	3	1	1	14	30.6076	294.3316
AWC::eat4	3	1	1	15	58.5307	73.3153
AWC::eat4	3	1	1	16	29.1879	142.7138
AWC::eat4	3	1	1	17	52.6513	218.1556
AWC::eat4	3	1	2	1	64.7941	212.0233
AWC::eat4	3	1	2	2	71.3840	217.7508
AWC::eat4	3	1	2	3	20.8449	422.9717
AWC::eat4	3	1	2	4	68.7925	168.7262
AWC::eat4	3	1	2	5	19.5976	321.1772
AWC::eat4	3	1	2	6	51.8009	112.0598
AWC::eat4	3	1	2	7	41.6026	335.5866
AWC::eat4	3	1	2	8	73.3833	195.5671
AWC::eat4	3	1	2	9	46.1425	229.8283
AWC::eat4	3	1	2	10	31.0736	70.3078
AWC::eat4	3	1	2	11	76.7880	119.8454
AWC::eat4	3	1	2	12	8.4301	247.7284
AWC::eat4	3	1	2	13	39.7524	282.4129
AWC::eat4	3	1	2	14	52.7190	301.1327
AWC::eat4	3	1	2	15	48.3487	88.7120
AWC::eat4	3	1	2	16	82.8460	49.7579
AWC::eat4	3	1	2	17	41.9224	33.4769
AWC::eat4	3	1	2	18	30.8241	342.0696
AWC::eat4	3	1	2	19	51.7540	324.5332
AWC::eat4	3	1	2	20	76.3466	131.0693

AWC::eat4	3	1	3	1	56.9040	136.6760
AWC::eat4	3	1	3	2	86.8524	127.3340
AWC::eat4	3	1	3	3	41.4425	251.1427
AWC::eat4	3	1	3	4	53.6458	161.2389
AWC::eat4	3	1	3	5	50.4246	204.8422
AWC::eat4	3	1	3	6	61.7593	259.9096
AWC::eat4	3	1	3	7	39.4410	293.6506
AWC::eat4	3	1	3	8	80.1524	227.8145
AWC::eat4	3	1	3	9	84.8480	91.0925
AWC::eat4	3	1	3	10	21.0682	343.5757
AWC::eat4	3	1	3	11	80.8209	39.2895
AWC::eat4	3	1	3	12	86.7974	49.7704
AWC::eat4	3	1	3	13	22.1969	281.7644
AWC::eat4	3	1	3	14	79.0729	235.8473
AWC::eat4	3	1	3	15	75.2405	107.8400
AWC::eat4	3	1	3	16	101.3291	11.8746
AWC::eat4	3	1	3	17	31.4313	238.4917
AWC::eat4	3	1	4	1	51.8947	195.4295
AWC::eat4	3	1	4	2	33.7313	217.4102
AWC::eat4	3	1	4	3	53.5535	280.8463
AWC::eat4	3	1	4	4	33.0834	288.0536
AWC::eat4	3	1	4	5	37.2177	303.4539
AWC::eat4	3	1	4	6	60.1888	265.0243
AWC::eat4	3	1	4	7	37.4806	267.1368
AWC::eat4	3	1	4	8	70.4531	71.4310
AWC::eat4	3	1	4	9	57.5809	32.1120
AWC::eat4	3	1	4	10	92.6908	145.1887
AWC::eat4	3	1	4	11	111.0374	69.1737
AWC::eat4	3	2	1	1	69.3226	82.5410
AWC::eat4	3	2	1	2	45.5464	119.5145
AWC::eat4	3	2	1	3	28.2257	283.8455
AWC::eat4	3	2	1	4	47.1874	230.7526
AWC::eat4	3	2	1	5	54.7946	176.1591
AWC::eat4	3	2	1	6	43.6220	178.1966
AWC::eat4	3	2	1	7	99.3433	12.1919
AWC::eat4	3	2	1	8	16.9700	178.4739
AWC::eat4	3	2	1	9	67.6395	152.8192
AWC::eat4	3	2	1	10	33.1774	392.2457
AWC::eat4	3	2	1	11	55.5903	114.1528
AWC::eat4	3	2	2	1	28.5779	397.6357
AWC::eat4	3	2	2	2	33.6946	271.3333
AWC::eat4	3	2	2	3	72.9541	193.5332
AWC::eat4	3	2	2	4	69.5744	214.8994
AWC::eat4	3	2	2	5	65.5709	229.3424
AWC::eat4	3	2	2	6	40.3305	234.9079
AWC::eat4	3	2	2	7	43.2914	158.7372
AWC::eat4	3	2	2	8	68.0428	129.4383
AWC::eat4	3	2	2	9	39.3411	274.8548
AWC::eat4	3	2	2	10	69.3047	110.0261
AWC::eat4	3	2	2	11	12.9233	305.2936
AWC::eat4	3	2	2	12	47.1544	290.4167
AWC::eat4	3	2	2	13	58.4537	87.6448
AWC::eat4	3	2	2	14	93.3333	79.8612
AWC::eat4	3	2	2	15	65.2322	95.3250
AWC::eat4	3	2	3	1	22.2404	287.6574
AWC::eat4	3	2	3	2	51.8270	244.6456
AWC::eat4	3	2	3	3	90.9899	103.5401
AWC::eat4	3	2	3	4	51.0109	214.3556
AWC::eat4	3	2	3	5	53.0337	134.5905
AWC::eat4	3	2	3	6	33.9320	311.7304
AWC::eat4	3	2	3	7	47.1378	250.8055
AWC::eat4	3	2	3	8	67.1922	140.2103
AWC::eat4	3	2	3	9	71.6734	114.1282
AWC::eat4	3	2	3	10	46.1324	212.8157
AWC::eat4	3	2	3	11	65.9201	213.2083
AWC::eat4	3	2	3	12	7.6078	294.9657
AWC::eat4	3	2	3	13	23.7130	262.4315
AWC::eat4	3	2	3	14	26.0296	211.6378

AWC::eat4	3	2	3	15	75.3344	41.5479
AWC::eat4	3	2	3	16	56.6489	153.5515
AWC::eat4	3	2	3	17	75.9228	262.0663
AWC::eat4	3	2	3	18	28.2212	205.8720
AWC::eat4	3	2	4	1	57.9189	161.3411
AWC::eat4	3	2	4	2	63.7636	185.5848
AWC::eat4	3	2	4	3	39.9496	182.1577
AWC::eat4	3	2	4	4	80.7511	91.6297
AWC::eat4	3	2	4	5	33.4224	283.7553
AWC::eat4	3	2	4	6	22.0823	320.6540
AWC::eat4	3	2	4	7	82.5837	66.4987
AWC::eat4	3	2	4	8	39.2243	289.1688
AWC::eat4	3	2	4	9	51.3091	216.0120
AWC::eat4	3	2	4	10	78.2988	164.8424
AWC::eat4	3	2	4	11	53.8694	110.3056
AWC::eat4	3	2	4	12	84.9685	83.8896
AWC::eat4	3	2	4	13	103.7845	69.7352
AWC::eat4	3	2	4	14	9.9697	316.3172
AWC::eat4	3	2	4	15	19.5809	312.8644
AWC::eat4	3	3	1	1	17.6323	281.4603
AWC::eat4	3	3	1	2	10.0789	350.5345
AWC::eat4	3	3	1	3	37.2111	256.5042
AWC::eat4	3	3	1	4	23.9120	401.7116
AWC::eat4	3	3	1	5	53.7227	142.0187
AWC::eat4	3	3	1	6	60.7144	170.6505
AWC::eat4	3	3	1	7	50.1793	136.8278
AWC::eat4	3	3	1	8	71.9593	268.6353
AWC::eat4	3	3	1	9	115.9853	5.3153
AWC::eat4	3	3	1	10	86.0536	56.3058
AWC::eat4	3	3	1	11	35.6340	403.0137
AWC::eat4	3	3	1	12	60.0219	169.3134
AWC::eat4	3	3	1	13	42.9896	303.8055
AWC::eat4	3	3	1	14	74.3120	43.9787
AWC::eat4	3	3	1	15	41.8667	180.2947
AWC::eat4	3	3	1	16	38.4589	279.8789
AWC::eat4	3	3	1	17	60.3431	105.5296
AWC::eat4	3	3	1	18	26.8117	81.1878
AWC::eat4	3	3	1	19	10.0935	260.3665
AWC::eat4	3	3	2	1	97.8986	108.2152
AWC::eat4	3	3	2	2	46.4130	216.8934
AWC::eat4	3	3	2	3	53.7669	125.3426
AWC::eat4	3	3	2	4	72.8671	80.6751
AWC::eat4	3	3	2	5	73.0797	164.7551
AWC::eat4	3	3	2	6	65.7170	69.5651
AWC::eat4	3	3	2	7	63.4610	94.6294
AWC::eat4	3	3	2	8	12.6978	254.1495
AWC::eat4	3	3	2	9	80.5568	156.1810
AWC::eat4	3	3	2	10	8.1616	197.1483
AWC::eat4	3	3	2	11	36.5037	219.7326
AWC::eat4	3	3	2	12	25.1685	299.7727
AWC::eat4	3	3	2	13	31.9862	340.5815
AWC::eat4	3	3	2	14	25.3622	413.1291
AWC::eat4	3	3	2	15	60.4771	178.2311
AWC::eat4	3	3	2	16	19.2164	276.7795
AWC::eat4	3	3	2	17	75.4431	148.5010
AWC::eat4	3	3	3	1	60.5463	125.2599
AWC::eat4	3	3	3	2	22.5847	211.3679
AWC::eat4	3	3	3	3	34.4428	246.1111
AWC::eat4	3	3	3	4	34.0285	256.9757
AWC::eat4	3	3	3	5	83.5044	35.7652
AWC::eat4	3	3	3	6	30.8668	332.4290
AWC::eat4	3	3	3	7	19.0859	296.6176
AWC::eat4	3	3	3	8	26.4858	220.6081
AWC::eat4	3	3	3	9	23.1177	270.6152
AWC::eat4	3	3	3	10	49.2865	215.5121
AWC::eat4	3	3	3	11	67.4106	193.7077
AWC::eat4	3	3	3	12	80.6293	78.4541
AWC::eat4	3	3	3	13	57.4494	81.3316

AWC::eat4	3	3	3	14	41.5857	321.2262
AWC::eat4	3	3	3	15	70.4386	119.1667
AWC::eat4	3	3	3	16	104.0656	82.5559
AWC::eat4	3	3	3	17	40.8741	141.2253
AWC::eat4	3	3	3	18	89.8662	200.5303
AWC::eat4	3	3	3	19	92.0730	134.1105
AWC::eat4	3	3	3	20	20.7282	102.2601
AWC::eat4	3	3	4	1	69.9509	151.0385
AWC::eat4	3	3	4	2	20.0965	245.2105
AWC::eat4	3	3	4	3	77.8238	242.0741
AWC::eat4	3	3	4	4	88.7692	61.3556
AWC::eat4	3	3	4	5	65.5042	180.9416
AWC::eat4	3	3	4	6	71.5310	258.5877
AWC::eat4	3	3	4	7	28.1222	273.4849
AWC::eat4	3	3	4	8	32.7953	325.2522
AWC::eat4	3	3	4	9	79.1702	47.7919
AWC::eat4	3	3	4	10	52.0094	96.4273
AWC::eat4	3	3	4	11	78.6633	89.1865
AWC::eat4	3	3	4	12	73.0217	3.7428
AWC::eat4	3	3	4	13	34.9867	133.3379
AWC::eat4	3	3	4	14	22.6293	224.2531
AWC::eat4	3	3	4	15	60.7027	134.7008
AWC::eat4	3	3	4	16	29.9912	257.3087
AWC::eat4	6	1	1	1	23.6632	213.3745
AWC::eat4	6	1	1	2	41.2665	206.4602
AWC::eat4	6	1	1	3	67.3130	146.2420
AWC::eat4	6	1	1	4	40.2484	183.6708
AWC::eat4	6	1	1	5	90.6005	60.4046
AWC::eat4	6	1	1	6	41.8993	173.9648
AWC::eat4	6	1	1	7	74.2601	50.6066
AWC::eat4	6	1	1	8	32.7970	259.6676
AWC::eat4	6	1	1	9	22.5340	216.1637
AWC::eat4	6	1	1	10	58.1228	115.2682
AWC::eat4	6	1	1	11	54.0556	261.8163
AWC::eat4	6	1	1	12	65.4232	208.8480
AWC::eat4	6	1	1	13	44.0198	218.1853
AWC::eat4	6	1	1	14	94.5816	88.5102
AWC::eat4	6	1	1	15	58.2319	147.6794
AWC::eat4	6	1	1	16	95.9697	70.5403
AWC::eat4	6	1	2	1	54.1763	162.7701
AWC::eat4	6	1	2	2	56.2357	222.0348
AWC::eat4	6	1	2	3	16.4520	235.5631
AWC::eat4	6	1	2	4	70.7314	228.6954
AWC::eat4	6	1	2	5	57.2261	229.0704
AWC::eat4	6	1	2	6	76.9413	148.1282
AWC::eat4	6	1	2	7	86.4580	112.0782
AWC::eat4	6	1	2	8	62.1116	133.3638
AWC::eat4	6	1	2	9	60.1479	152.6845
AWC::eat4	6	1	2	10	48.5268	150.6087
AWC::eat4	6	1	2	11	40.0205	222.7021
AWC::eat4	6	1	2	12	100.4046	140.3084
AWC::eat4	6	1	2	13	69.4183	148.5608
AWC::eat4	6	1	2	14	64.9033	95.1263
AWC::eat4	6	1	2	15	33.5748	178.1291
AWC::eat4	6	1	2	16	97.3427	259.2955
AWC::eat4	6	1	2	17	36.2529	222.3694
AWC::eat4	6	1	2	18	83.5288	204.5774
AWC::eat4	6	1	2	19	47.5163	141.5952
AWC::eat4	6	1	3	1	68.6015	170.9955
AWC::eat4	6	1	3	2	45.8460	238.2271
AWC::eat4	6	1	3	3	44.4547	249.9718
AWC::eat4	6	1	3	4	91.6373	165.5445
AWC::eat4	6	1	3	5	92.0124	111.4207
AWC::eat4	6	1	3	6	56.7960	145.0591
AWC::eat4	6	1	3	7	93.9588	134.4135
AWC::eat4	6	1	3	8	34.3784	132.4159
AWC::eat4	6	1	3	9	110.6044	191.7841
AWC::eat4	6	1	3	10	59.5564	191.3682

AWC::eat4	6	1	3	11	54.1134	76.3091
AWC::eat4	6	1	3	12	93.5116	200.4907
AWC::eat4	6	1	3	13	33.0890	239.0426
AWC::eat4	6	1	3	14	76.9756	123.0979
AWC::eat4	6	1	3	15	87.8246	175.8728
AWC::eat4	6	1	3	16	83.6195	120.4970
AWC::eat4	6	1	3	17	98.6592	62.5544
AWC::eat4	6	1	3	18	109.1687	104.8774
AWC::eat4	6	1	3	19	12.2009	215.6522
AWC::eat4	6	1	3	20	87.7788	119.6300
AWC::eat4	6	1	4	1	65.9291	227.1018
AWC::eat4	6	1	4	2	50.7059	256.0560
AWC::eat4	6	1	4	3	71.8270	156.9640
AWC::eat4	6	1	4	4	21.1356	263.4237
AWC::eat4	6	1	4	5	52.9688	130.3504
AWC::eat4	6	1	4	6	60.2372	233.7101
AWC::eat4	6	1	4	7	58.6525	150.5472
AWC::eat4	6	1	4	8	30.2522	212.6086
AWC::eat4	6	1	4	9	76.4125	128.7631
AWC::eat4	6	1	4	10	37.6295	195.1198
AWC::eat4	6	1	4	11	85.7998	78.7883
AWC::eat4	6	1	4	12	81.2300	201.2481
AWC::eat4	6	1	4	13	74.5651	179.6348
AWC::eat4	6	1	4	14	119.8095	206.0104
AWC::eat4	6	1	4	15	57.3827	112.0095
AWC::eat4	6	1	4	16	80.6531	136.9945
AWC::eat4	6	1	4	17	81.9957	150.5729
AWC::eat4	6	1	4	18	80.2615	146.7563
AWC::eat4	6	1	4	19	92.0927	149.6293
AWC::eat4	6	2	1	1	66.4942	323.8011
AWC::eat4	6	2	1	2	37.8067	234.5621
AWC::eat4	6	2	1	3	45.6525	195.2494
AWC::eat4	6	2	1	4	93.9503	96.8429
AWC::eat4	6	2	1	5	76.6754	165.8963
AWC::eat4	6	2	1	6	65.4605	284.9253
AWC::eat4	6	2	1	7	79.6768	231.5681
AWC::eat4	6	2	1	8	62.5577	197.5815
AWC::eat4	6	2	1	9	73.5297	168.4101
AWC::eat4	6	2	1	10	65.3901	220.8542
AWC::eat4	6	2	1	11	19.1821	223.0794
AWC::eat4	6	2	1	12	49.3043	254.2517
AWC::eat4	6	2	1	13	61.4160	294.7204
AWC::eat4	6	2	2	1	36.2670	284.6691
AWC::eat4	6	2	2	2	38.5660	223.8579
AWC::eat4	6	2	2	3	67.3458	245.8828
AWC::eat4	6	2	2	4	50.7817	155.8863
AWC::eat4	6	2	2	5	74.6791	184.0438
AWC::eat4	6	2	2	6	45.7556	156.7238
AWC::eat4	6	2	2	7	71.2510	192.3970
AWC::eat4	6	2	2	8	129.0886	116.2340
AWC::eat4	6	2	2	9	57.3758	237.5751
AWC::eat4	6	2	2	10	55.2470	301.8177
AWC::eat4	6	2	2	11	53.3419	269.6296
AWC::eat4	6	2	2	12	90.1109	259.0793
AWC::eat4	6	2	2	13	77.4559	99.4477
AWC::eat4	6	2	2	14	30.0189	217.3076
AWC::eat4	6	2	2	15	66.6665	154.3600
AWC::eat4	6	2	2	16	120.9893	178.0781
AWC::eat4	6	2	2	17	40.4590	104.7865
AWC::eat4	6	2	2	18	56.5881	161.9276
AWC::eat4	6	2	2	19	62.8034	134.9156
AWC::eat4	6	2	3	1	60.4070	192.2433
AWC::eat4	6	2	3	2	69.4340	168.7802
AWC::eat4	6	2	3	3	52.4487	205.7455
AWC::eat4	6	2	3	4	40.2691	201.4812
AWC::eat4	6	2	3	5	59.3031	179.3043
AWC::eat4	6	2	3	6	82.4221	196.5864
AWC::eat4	6	2	3	7	52.4317	207.9031

AWC::eat4	6	2	3	8	98.1933	193.3620
AWC::eat4	6	2	3	9	85.2653	150.6880
AWC::eat4	6	2	3	10	95.1572	198.6338
AWC::eat4	6	2	3	11	55.4228	184.4120
AWC::eat4	6	2	3	12	42.9284	182.6624
AWC::eat4	6	2	3	13	41.2564	208.8074
AWC::eat4	6	2	3	14	105.2321	143.2841
AWC::eat4	6	2	3	15	46.5707	220.4827
AWC::eat4	6	2	3	16	118.4774	231.7140
AWC::eat4	6	2	3	17	45.5860	338.7622
AWC::eat4	6	2	3	18	13.0606	380.1097
AWC::eat4	6	2	3	19	92.3014	84.2636
AWC::eat4	6	2	4	1	56.5657	244.5100
AWC::eat4	6	2	4	2	31.9815	287.9693
AWC::eat4	6	2	4	3	80.6315	187.7801
AWC::eat4	6	2	4	4	76.9090	172.3468
AWC::eat4	6	2	4	5	63.9666	258.4082
AWC::eat4	6	2	4	6	44.1025	268.7503
AWC::eat4	6	2	4	7	63.0033	47.4457
AWC::eat4	6	2	4	8	68.5341	166.9915
AWC::eat4	6	2	4	9	84.2694	97.0700
AWC::eat4	6	2	4	10	81.5435	165.9201
AWC::eat4	6	2	4	11	97.0998	170.9627
AWC::eat4	6	2	4	12	30.1624	257.5374
AWC::eat4	6	2	4	13	26.9012	206.8456
AWC::eat4	6	2	4	14	28.1393	281.2388
AWC::eat4	6	2	4	15	57.5082	207.1781
AWC::eat4	6	2	4	16	70.6110	233.7563
AWC::eat4	6	3	1	1	38.9719	232.8162
AWC::eat4	6	3	1	2	69.2547	116.5139
AWC::eat4	6	3	1	3	88.5682	110.9132
AWC::eat4	6	3	1	4	76.7922	170.3854
AWC::eat4	6	3	1	5	61.4153	32.1548
AWC::eat4	6	3	1	6	20.6439	221.3095
AWC::eat4	6	3	1	7	109.3555	3.6216
AWC::eat4	6	3	1	8	62.7556	195.3518
AWC::eat4	6	3	1	9	56.2930	223.9925
AWC::eat4	6	3	1	10	84.3642	207.9362
AWC::eat4	6	3	1	11	76.7458	168.7937
AWC::eat4	6	3	2	1	49.9538	203.5189
AWC::eat4	6	3	2	2	68.8451	143.1448
AWC::eat4	6	3	2	3	67.4929	112.8955
AWC::eat4	6	3	2	4	68.5372	103.6693
AWC::eat4	6	3	2	5	69.1703	180.4976
AWC::eat4	6	3	2	6	75.1337	155.5213
AWC::eat4	6	3	2	7	66.5094	175.4397
AWC::eat4	6	3	2	8	51.0822	96.6492
AWC::eat4	6	3	2	9	64.3659	192.2014
AWC::eat4	6	3	2	10	86.7761	241.5484
AWC::eat4	6	3	2	11	92.3568	5.3166
AWC::eat4	6	3	2	12	73.9916	218.4557
AWC::eat4	6	3	2	13	95.4912	9.4233
AWC::eat4	6	3	2	14	49.1152	223.9646
AWC::eat4	6	3	2	15	57.4188	129.7721
AWC::eat4	6	3	2	16	40.7911	268.0988
AWC::eat4	6	3	2	17	19.1947	211.2709
AWC::eat4	6	3	2	18	128.3655	49.3796
AWC::eat4	6	3	3	1	49.6802	298.2989
AWC::eat4	6	3	3	2	34.8211	155.4905
AWC::eat4	6	3	3	3	66.9407	172.3905
AWC::eat4	6	3	3	4	38.9407	176.0151
AWC::eat4	6	3	3	5	56.9282	136.5874
AWC::eat4	6	3	3	6	39.2538	213.0269
AWC::eat4	6	3	3	7	89.7040	120.3484
AWC::eat4	6	3	3	8	65.8399	228.8880
AWC::eat4	6	3	3	9	56.6319	149.7335
AWC::eat4	6	3	3	10	59.8383	265.6999
AWC::eat4	6	3	3	11	116.2222	159.8814

AWC::eat4	6	3	3	12	82.5684	93.4187
AWC::eat4	6	3	3	13	113.3906	181.9849
AWC::eat4	6	3	3	14	28.3613	232.0272
AWC::eat4	6	3	3	15	67.0759	138.8133
AWC::eat4	6	3	3	16	55.1856	239.4124
AWC::eat4	6	3	3	17	98.4304	172.9866
AWC::eat4	6	3	3	18	75.7925	118.5773
AWC::eat4	6	3	3	19	77.7767	94.6357
AWC::eat4	6	3	3	20	92.5975	233.7582
AWC::eat4	6	3	3	21	42.9179	171.4177
AWC::eat4	6	3	4	1	68.5387	183.2229
AWC::eat4	6	3	4	2	12.8165	293.7298
AWC::eat4	6	3	4	3	37.7462	321.9710
AWC::eat4	6	3	4	4	75.9595	139.1462
AWC::eat4	6	3	4	5	75.7539	125.4819
AWC::eat4	6	3	4	6	74.0668	141.9645
AWC::eat4	6	3	4	7	74.6507	50.8406
AWC::eat4	6	3	4	8	51.3425	75.5936
AWC::eat4	6	3	4	9	47.6810	159.7242
AWC::eat4	6	3	4	10	28.3357	211.1851
AWC::eat4	6	3	4	11	45.6754	151.3409
AWC::eat4	6	3	4	12	83.1235	154.2737
AWC::eat4	6	3	4	13	43.1586	226.6149
AWC::eat4	6	3	4	14	79.1848	162.8616
AWC::eat4	6	3	4	15	87.9029	294.6994
AWC::eat4	6	3	4	16	41.9001	149.6001
nsy-1	0	1	1	1	73.1619	31.6050
nsy-1	0	1	1	2	93.9780	73.8370
nsy-1	0	1	1	3	112.8314	109.3235
nsy-1	0	1	1	4	105.3795	33.6103
nsy-1	0	1	1	5	93.2962	104.6219
nsy-1	0	1	1	6	57.7250	103.2799
nsy-1	0	1	1	7	74.0311	82.2992
nsy-1	0	1	1	8	52.8085	80.5368
nsy-1	0	1	1	9	59.2313	42.9606
nsy-1	0	1	1	10	97.5838	74.5801
nsy-1	0	1	1	11	98.2031	83.0224
nsy-1	0	1	2	1	114.8850	57.1600
nsy-1	0	1	2	2	79.4517	120.3587
nsy-1	0	1	2	3	84.8449	3.7822
nsy-1	0	1	2	4	117.3409	101.5791
nsy-1	0	1	2	5	104.1891	81.1319
nsy-1	0	1	2	6	106.6892	78.7930
nsy-1	0	1	2	7	83.5659	38.4484
nsy-1	0	1	2	8	97.8488	116.9553
nsy-1	0	1	2	9	88.9925	102.6830
nsy-1	0	1	2	10	60.1159	55.6763
nsy-1	0	1	2	11	78.2948	113.6563
nsy-1	0	1	2	12	81.4657	31.1179
nsy-1	0	1	3	1	104.9791	125.6175
nsy-1	0	1	3	2	92.8511	60.4029
nsy-1	0	1	3	3	93.3949	35.5327
nsy-1	0	1	3	4	93.9761	81.6328
nsy-1	0	1	3	5	87.1490	18.4534
nsy-1	0	1	3	6	128.4746	130.1409
nsy-1	0	1	3	7	108.3153	88.5295
nsy-1	0	1	3	8	154.7697	57.8722
nsy-1	0	1	3	9	93.0607	72.5243
nsy-1	0	1	3	10	66.9831	104.7448
nsy-1	0	1	3	11	55.4570	70.4204
nsy-1	0	1	3	12	96.1394	52.8956
nsy-1	0	1	3	13	81.7858	34.8493
nsy-1	0	1	3	14	80.4425	88.1383
nsy-1	0	1	3	15	125.0456	13.8645
nsy-1	0	1	4	1	36.5469	108.1822
nsy-1	0	1	4	2	134.2595	101.5610
nsy-1	0	1	4	3	105.6977	61.4602
nsy-1	0	1	4	4	90.7733	3.5365

nsy-1	0	1	4	5	126.9546	15.3478
nsy-1	0	1	4	6	122.2373	101.6747
nsy-1	0	1	4	7	167.0991	158.7204
nsy-1	0	1	4	8	149.9526	112.8961
nsy-1	0	1	4	9	121.3272	88.5713
nsy-1	0	1	4	10	127.0315	95.5587
nsy-1	0	1	4	11	60.0619	116.3646
nsy-1	0	1	4	12	103.5072	92.8743
nsy-1	0	1	4	13	126.7929	51.0108
nsy-1	0	1	4	14	74.9765	141.9301
nsy-1	0	2	1	1	82.3222	47.8899
nsy-1	0	2	1	2	103.2618	89.7318
nsy-1	0	2	1	3	136.2131	60.2632
nsy-1	0	2	1	4	105.3018	57.6273
nsy-1	0	2	1	5	77.9514	118.1453
nsy-1	0	2	1	6	132.0734	28.4981
nsy-1	0	2	1	7	76.1166	209.8845
nsy-1	0	2	1	8	151.2790	197.2829
nsy-1	0	2	1	9	67.5674	76.0344
nsy-1	0	2	1	10	97.8769	65.5249
nsy-1	0	2	1	11	59.8073	66.2438
nsy-1	0	2	1	12	119.9708	113.3596
nsy-1	0	2	1	13	78.4537	90.7190
nsy-1	0	2	1	14	130.0909	53.7830
nsy-1	0	2	1	15	74.0337	27.0073
nsy-1	0	2	1	16	54.1312	30.4108
nsy-1	0	2	2	1	80.6009	17.7466
nsy-1	0	2	2	2	39.2689	80.7622
nsy-1	0	2	2	3	78.5061	55.5366
nsy-1	0	2	2	4	114.7172	18.4190
nsy-1	0	2	2	5	52.2188	70.8547
nsy-1	0	2	2	6	86.2174	4.9463
nsy-1	0	2	2	7	120.9506	25.6957
nsy-1	0	2	2	8	88.4253	23.5302
nsy-1	0	2	2	9	44.5921	90.2556
nsy-1	0	2	2	10	80.9027	139.7754
nsy-1	0	2	2	11	103.6516	28.4849
nsy-1	0	2	2	12	89.1715	23.3094
nsy-1	0	2	2	13	125.6070	79.7252
nsy-1	0	2	2	14	80.7013	35.1129
nsy-1	0	2	2	15	36.7560	60.7534
nsy-1	0	2	2	16	80.9006	54.9604
nsy-1	0	2	2	17	123.7535	11.0645
nsy-1	0	2	2	18	104.0226	90.8948
nsy-1	0	2	2	19	105.5675	95.5384
nsy-1	0	2	3	1	116.8144	101.2819
nsy-1	0	2	3	2	88.9508	22.5653
nsy-1	0	2	3	3	141.6493	58.9132
nsy-1	0	2	3	4	72.4746	25.9599
nsy-1	0	2	3	5	77.7062	130.8943
nsy-1	0	2	3	6	71.3172	101.7337
nsy-1	0	2	3	7	103.1552	63.2853
nsy-1	0	2	3	8	90.8377	17.9231
nsy-1	0	2	3	9	93.3168	56.3183
nsy-1	0	2	3	10	81.0701	46.6914
nsy-1	0	2	3	11	107.1045	57.4156
nsy-1	0	2	3	12	69.3232	117.1461
nsy-1	0	2	3	13	165.6769	63.1081
nsy-1	0	2	3	14	132.8266	40.4209
nsy-1	0	2	3	15	19.3950	127.2514
nsy-1	0	2	3	16	123.4522	48.1731
nsy-1	0	2	3	17	85.7157	30.3206
nsy-1	0	2	3	18	125.0181	35.5958
nsy-1	0	2	4	1	23.5308	162.7897
nsy-1	0	2	4	2	26.8883	91.6285
nsy-1	0	2	4	3	76.0319	33.3807
nsy-1	0	2	4	4	65.5966	99.4486
nsy-1	0	2	4	5	85.5118	110.9882

nsy-1	0	2	4	6	86.6492 52.1932
nsy-1	0	2	4	7	23.1468 160.4797
nsy-1	0	2	4	8	108.0635 39.0587
nsy-1	0	2	4	9	73.5285 59.6995
nsy-1	0	2	4	10	44.6469 91.3718
nsy-1	0	2	4	11	109.8582 40.8179
nsy-1	0	2	4	12	46.9102 138.5140
nsy-1	0	2	4	13	116.8851 77.5611
nsy-1	0	2	4	14	101.1631 34.7265
nsy-1	0	2	4	15	160.2173 58.1255
nsy-1	0	2	4	16	98.9759 22.7024
nsy-1	0	2	4	17	131.6993 85.2110
nsy-1	0	2	4	18	57.4218 45.5654
nsy-1	0	2	4	19	45.4539 160.9572
nsy-1	0	2	4	20	97.6765 62.9098
nsy-1	0	3	1	1	100.8668 73.1567
nsy-1	0	3	1	2	91.6208 163.0882
nsy-1	0	3	1	3	101.1437 26.0292
nsy-1	0	3	1	4	67.0698 88.2160
nsy-1	0	3	1	5	111.6921 103.3500
nsy-1	0	3	1	6	85.8300 150.8554
nsy-1	0	3	1	7	91.4315 146.6161
nsy-1	0	3	1	8	98.3672 51.2009
nsy-1	0	3	1	9	70.5814 49.6351
nsy-1	0	3	1	10	105.8834 83.8186
nsy-1	0	3	1	11	94.0625 71.4675
nsy-1	0	3	1	12	67.0474 117.4721
nsy-1	0	3	1	13	109.2753 50.3440
nsy-1	0	3	1	14	102.3434 35.6492
nsy-1	0	3	1	15	68.8615 61.1789
nsy-1	0	3	1	16	114.4025 87.8556
nsy-1	0	3	1	17	94.8349 109.2811
nsy-1	0	3	1	18	60.9919 146.3877
nsy-1	0	3	1	19	45.3071 51.8252
nsy-1	0	3	2	1	82.2478 125.1240
nsy-1	0	3	2	2	80.3130 148.5269
nsy-1	0	3	2	3	51.3386 161.0625
nsy-1	0	3	2	4	83.0235 166.2738
nsy-1	0	3	2	5	114.0898 80.0433
nsy-1	0	3	2	6	117.6245 92.5541
nsy-1	0	3	2	7	129.9930 77.5958
nsy-1	0	3	2	8	98.4696 56.0492
nsy-1	0	3	2	9	89.6885 109.2749
nsy-1	0	3	2	10	140.6401 59.4285
nsy-1	0	3	2	11	77.0442 71.9518
nsy-1	0	3	2	12	43.8247 119.7653
nsy-1	0	3	2	13	68.5470 108.3863
nsy-1	0	3	3	1	36.3525 145.1374
nsy-1	0	3	3	2	27.7513 102.6179
nsy-1	0	3	3	3	96.9030 149.0009
nsy-1	0	3	3	4	41.1361 41.0381
nsy-1	0	3	3	5	113.4393 165.8304
nsy-1	0	3	3	6	110.8513 67.3324
nsy-1	0	3	3	7	56.4234 85.2597
nsy-1	0	3	3	8	58.7454 36.4812
nsy-1	0	3	3	9	123.9315 75.7873
nsy-1	0	3	3	10	125.2322 25.5620
nsy-1	0	3	3	11	128.3773 35.6387
nsy-1	0	3	3	12	45.8016 147.4983
nsy-1	0	3	3	13	16.7274 155.1483
nsy-1	0	3	3	14	56.8140 41.9844
nsy-1	0	3	4	1	123.4329 106.4077
nsy-1	0	3	4	2	115.4838 141.7839
nsy-1	0	3	4	3	98.8748 118.0009
nsy-1	0	3	4	4	120.0561 57.5501
nsy-1	0	3	4	5	60.4821 136.6451
nsy-1	0	3	4	6	73.7765 59.3327
nsy-1	0	3	4	7	116.8302 81.8917

nsy-1	0	3	4	8	130.2768	97.6148
nsy-1	0	3	4	9	128.8248	120.6995
nsy-1	0	3	4	10	72.8257	43.8181
nsy-1	0	3	4	11	88.6841	55.8711
nsy-1	0	3	4	12	74.2914	33.7204
nsy-1	0	3	4	13	60.3024	139.7152
nsy-1	0	3	4	14	92.6482	95.7351
nsy-1	0	3	4	15	41.0770	57.3315
nsy-1	0	3	4	16	64.3932	43.9859
nsy-1	1.5	1	1	1	45.5196	155.5303
nsy-1	1.5	1	1	2	9.2177	189.4583
nsy-1	1.5	1	1	3	8.0557	153.5363
nsy-1	1.5	1	1	4	34.4023	160.2239
nsy-1	1.5	1	1	5	7.1867	156.1138
nsy-1	1.5	1	1	6	16.5337	202.0373
nsy-1	1.5	1	1	7	34.5012	103.1747
nsy-1	1.5	1	1	8	26.7397	193.1881
nsy-1	1.5	1	2	1	6.7239	126.2302
nsy-1	1.5	1	2	2	11.8142	178.6222
nsy-1	1.5	1	2	3	6.8025	154.4316
nsy-1	1.5	1	2	4	12.9722	123.2932
nsy-1	1.5	1	2	5	15.7039	135.9810
nsy-1	1.5	1	2	6	8.6445	131.2566
nsy-1	1.5	1	2	7	14.6966	197.9345
nsy-1	1.5	1	2	8	67.3504	68.7224
nsy-1	1.5	1	2	9	33.9709	112.7131
nsy-1	1.5	1	2	10	96.5147	85.6020
nsy-1	1.5	1	2	11	55.3057	74.2452
nsy-1	1.5	1	2	12	51.3066	145.3211
nsy-1	1.5	1	2	13	62.8801	60.5608
nsy-1	1.5	1	2	14	82.7589	174.4945
nsy-1	1.5	1	2	15	100.2433	42.8627
nsy-1	1.5	1	2	16	33.5668	136.3959
nsy-1	1.5	1	2	17	34.0206	44.2184
nsy-1	1.5	1	2	18	137.8042	103.3319
nsy-1	1.5	1	2	19	12.0116	116.9501
nsy-1	1.5	1	2	20	78.6261	79.7805
nsy-1	1.5	1	2	21	10.4641	270.7134
nsy-1	1.5	1	2	22	71.0808	136.6296
nsy-1	1.5	1	2	23	9.8660	168.8295
nsy-1	1.5	1	3	1	105.2831	190.2979
nsy-1	1.5	1	3	2	10.3463	130.1387
nsy-1	1.5	1	3	3	35.8442	115.7030
nsy-1	1.5	1	3	4	79.4533	125.7301
nsy-1	1.5	1	3	5	14.3453	120.5803
nsy-1	1.5	1	3	6	101.6352	105.7810
nsy-1	1.5	1	3	7	13.9485	182.2081
nsy-1	1.5	1	3	8	10.6795	154.3589
nsy-1	1.5	1	3	9	18.5428	150.5320
nsy-1	1.5	1	3	10	18.2588	110.1405
nsy-1	1.5	1	3	11	13.1936	132.9453
nsy-1	1.5	1	3	12	80.1366	52.3773
nsy-1	1.5	1	3	13	9.0803	106.8446
nsy-1	1.5	1	3	14	42.6089	77.8005
nsy-1	1.5	1	3	15	9.6760	117.2556
nsy-1	1.5	1	3	16	17.2979	108.4787
nsy-1	1.5	1	3	17	57.0559	85.0582
nsy-1	1.5	1	3	18	82.8830	111.6074
nsy-1	1.5	1	3	19	128.2063	210.9149
nsy-1	1.5	1	3	20	77.8538	76.1448
nsy-1	1.5	1	3	21	56.3537	156.4542
nsy-1	1.5	1	3	22	40.1805	151.2591
nsy-1	1.5	1	3	23	133.4701	91.6468
nsy-1	1.5	1	3	24	63.0890	63.3388
nsy-1	1.5	1	3	25	85.6984	90.8005
nsy-1	1.5	1	3	26	6.1904	139.1973
nsy-1	1.5	1	3	27	43.0397	93.6085
nsy-1	1.5	1	3	28	72.0673	64.7403

nsy-1	1.5	1	4	1	46.2107	105.9382
nsy-1	1.5	1	4	2	94.5645	93.4916
nsy-1	1.5	1	4	3	9.9650	167.0133
nsy-1	1.5	1	4	4	13.3157	226.2955
nsy-1	1.5	1	4	5	14.7936	163.6438
nsy-1	1.5	1	4	6	19.8115	46.1031
nsy-1	1.5	1	4	7	23.0009	123.8322
nsy-1	1.5	1	4	8	67.6403	35.1673
nsy-1	1.5	1	4	9	17.5628	177.4041
nsy-1	1.5	1	4	10	8.2487	214.6181
nsy-1	1.5	1	4	11	74.6940	57.6939
nsy-1	1.5	1	4	12	16.4189	162.6958
nsy-1	1.5	1	4	13	56.9293	63.6100
nsy-1	1.5	1	4	14	24.1964	229.6814
nsy-1	1.5	1	4	15	19.4284	143.9727
nsy-1	1.5	1	4	16	53.3650	184.9574
nsy-1	1.5	1	4	17	48.3874	82.4815
nsy-1	1.5	1	4	18	60.8309	116.5974
nsy-1	1.5	1	4	19	50.7214	87.1160
nsy-1	1.5	1	4	20	105.0221	45.7547
nsy-1	1.5	1	4	21	11.9292	122.9333
nsy-1	1.5	1	4	22	67.9043	114.0568
nsy-1	1.5	1	4	23	99.7364	13.4480
nsy-1	1.5	1	1	1	10.2553	205.4382
nsy-1	1.5	2	1	2	6.8749	129.7247
nsy-1	1.5	2	1	3	12.9403	154.9672
nsy-1	1.5	2	1	4	11.1980	143.3755
nsy-1	1.5	2	1	5	11.2321	231.5393
nsy-1	1.5	2	1	6	11.1706	180.3046
nsy-1	1.5	2	1	7	21.6026	164.2111
nsy-1	1.5	2	1	8	17.0709	162.4571
nsy-1	1.5	2	1	9	110.6709	51.0869
nsy-1	1.5	2	1	10	9.7164	172.2788
nsy-1	1.5	2	1	11	11.3440	284.4325
nsy-1	1.5	2	1	12	11.9418	186.9844
nsy-1	1.5	2	1	13	32.8729	126.4295
nsy-1	1.5	2	2	1	69.4753	55.4202
nsy-1	1.5	2	2	2	7.7750	272.6766
nsy-1	1.5	2	2	3	60.6644	63.8684
nsy-1	1.5	2	2	4	14.1637	229.7594
nsy-1	1.5	2	2	5	56.0586	93.3311
nsy-1	1.5	2	2	6	45.2363	93.4471
nsy-1	1.5	2	2	7	24.5077	104.7701
nsy-1	1.5	2	2	8	15.5435	276.9085
nsy-1	1.5	2	2	9	9.3997	226.9580
nsy-1	1.5	2	2	10	19.0162	158.5831
nsy-1	1.5	2	2	11	43.3219	125.8164
nsy-1	1.5	2	2	12	7.7053	204.3663
nsy-1	1.5	2	2	13	11.3898	186.9183
nsy-1	1.5	2	2	14	61.8227	165.3224
nsy-1	1.5	2	2	15	7.7508	271.0563
nsy-1	1.5	2	2	16	39.0631	199.5576
nsy-1	1.5	2	2	17	17.1140	101.9700
nsy-1	1.5	2	2	18	14.8082	108.8067
nsy-1	1.5	2	2	19	43.5135	106.7468
nsy-1	1.5	2	2	20	28.3123	97.3557
nsy-1	1.5	2	2	21	60.2707	184.2302
nsy-1	1.5	2	2	22	66.9239	81.3730
nsy-1	1.5	2	2	23	54.7031	115.7794
nsy-1	1.5	2	2	24	83.7938	147.6304
nsy-1	1.5	2	3	1	24.2120	177.8891
nsy-1	1.5	2	3	2	39.7984	114.0484
nsy-1	1.5	2	3	3	85.2952	100.0980
nsy-1	1.5	2	3	4	11.0019	145.0372
nsy-1	1.5	2	3	5	11.8392	194.7088
nsy-1	1.5	2	3	6	65.2458	103.5467
nsy-1	1.5	2	3	7	14.8200	109.8196
nsy-1	1.5	2	3	8	72.9599	19.1888

nsy-1	1.5	2	3	9	17.7727	110.1010
nsy-1	1.5	2	3	10	80.4982	41.4793
nsy-1	1.5	2	3	11	7.6549	157.0662
nsy-1	1.5	2	3	12	45.5175	132.9658
nsy-1	1.5	2	3	13	61.6910	141.6799
nsy-1	1.5	2	3	14	130.2672	154.9472
nsy-1	1.5	2	3	15	79.4798	19.3126
nsy-1	1.5	2	3	16	77.2338	60.3424
nsy-1	1.5	2	3	17	11.5711	110.0881
nsy-1	1.5	2	3	18	10.1368	94.1519
nsy-1	1.5	2	3	19	77.6158	19.0762
nsy-1	1.5	2	3	20	66.2207	95.1125
nsy-1	1.5	2	3	21	94.9615	114.1112
nsy-1	1.5	2	3	22	13.2424	217.9962
nsy-1	1.5	2	3	23	49.6483	100.0283
nsy-1	1.5	2	3	24	8.6115	164.3486
nsy-1	1.5	2	3	25	9.3918	241.2956
nsy-1	1.5	2	3	26	93.9623	219.6327
nsy-1	1.5	2	3	27	70.5966	63.6337
nsy-1	1.5	2	4	1	38.9228	47.9372
nsy-1	1.5	2	4	2	15.3407	92.4560
nsy-1	1.5	2	4	3	80.0748	51.7565
nsy-1	1.5	2	4	4	26.8036	113.5285
nsy-1	1.5	2	4	5	12.0199	100.7080
nsy-1	1.5	2	4	6	27.1924	262.2833
nsy-1	1.5	2	4	7	12.7126	157.2099
nsy-1	1.5	2	4	8	7.2064	264.4180
nsy-1	1.5	2	4	9	29.4175	97.2800
nsy-1	1.5	2	4	10	53.8944	158.2582
nsy-1	1.5	2	4	11	69.9328	5.4755
nsy-1	1.5	2	4	12	93.8757	60.4310
nsy-1	1.5	2	4	13	11.2014	184.8605
nsy-1	1.5	2	4	14	94.2190	3.3862
nsy-1	1.5	2	4	15	64.2719	129.4208
nsy-1	1.5	2	4	16	16.0209	217.7066
nsy-1	1.5	2	4	17	11.3725	153.4156
nsy-1	1.5	2	4	18	90.4027	67.4696
nsy-1	1.5	2	4	19	8.4167	165.3671
nsy-1	1.5	2	4	20	79.8772	18.5598
nsy-1	1.5	2	4	21	72.5029	30.8571
nsy-1	1.5	2	4	22	17.1590	163.6078
nsy-1	1.5	2	4	23	104.0028	37.8903
nsy-1	1.5	2	4	24	22.1096	258.7896
nsy-1	1.5	3	1	1	11.7319	191.1956
nsy-1	1.5	3	1	2	14.0232	223.0931
nsy-1	1.5	3	1	3	13.6548	202.1063
nsy-1	1.5	3	1	4	12.8055	246.1567
nsy-1	1.5	3	1	5	34.0777	188.8933
nsy-1	1.5	3	1	6	9.1168	183.9300
nsy-1	1.5	3	1	7	7.3746	251.2450
nsy-1	1.5	3	1	8	23.7969	182.3835
nsy-1	1.5	3	1	9	37.2703	187.5227
nsy-1	1.5	3	1	10	21.7681	231.1169
nsy-1	1.5	3	1	11	10.5593	170.3770
nsy-1	1.5	3	1	12	13.6776	165.2046
nsy-1	1.5	3	1	13	11.8257	223.7058
nsy-1	1.5	3	1	14	16.4139	218.1858
nsy-1	1.5	3	2	1	10.1954	190.4247
nsy-1	1.5	3	2	2	7.6251	180.7906
nsy-1	1.5	3	2	3	6.9638	163.6864
nsy-1	1.5	3	2	4	6.1861	228.8455
nsy-1	1.5	3	2	5	14.4695	165.2721
nsy-1	1.5	3	2	6	6.8277	181.1983
nsy-1	1.5	3	2	7	26.6440	149.9076
nsy-1	1.5	3	2	8	7.6677	188.5367
nsy-1	1.5	3	2	9	29.4839	75.6662
nsy-1	1.5	3	2	10	33.2240	136.9058
nsy-1	1.5	3	2	11	13.1133	181.8186

nsy-1	1.5	3	2	12	13.6441	175.1255
nsy-1	1.5	3	2	13	13.9353	183.5694
nsy-1	1.5	3	2	14	14.6510	212.1428
nsy-1	1.5	3	2	15	58.8934	80.7184
nsy-1	1.5	3	2	16	16.2437	197.9009
nsy-1	1.5	3	2	17	20.2386	225.6034
nsy-1	1.5	3	2	18	6.3815	287.5958
nsy-1	1.5	3	2	19	22.0470	180.1062
nsy-1	1.5	3	2	20	7.2926	157.6520
nsy-1	1.5	3	2	21	9.7858	162.2817
nsy-1	1.5	3	2	22	30.2379	131.8253
nsy-1	1.5	3	2	23	72.1313	109.5685
nsy-1	1.5	3	2	24	70.7902	81.0581
nsy-1	1.5	3	2	25	11.4950	167.1106
nsy-1	1.5	3	2	26	38.8940	118.4562
nsy-1	1.5	3	3	1	7.6186	176.9093
nsy-1	1.5	3	3	2	26.1061	162.0383
nsy-1	1.5	3	3	3	6.7405	112.7241
nsy-1	1.5	3	3	4	6.6250	133.3087
nsy-1	1.5	3	3	5	15.4644	118.8346
nsy-1	1.5	3	3	6	16.6620	145.0907
nsy-1	1.5	3	3	7	20.7774	142.2972
nsy-1	1.5	3	3	8	19.1062	206.4741
nsy-1	1.5	3	3	9	19.3181	221.4083
nsy-1	1.5	3	3	10	9.9544	152.1913
nsy-1	1.5	3	3	11	15.6050	154.2213
nsy-1	1.5	3	3	12	5.9152	158.0033
nsy-1	1.5	3	3	13	19.6772	175.9994
nsy-1	1.5	3	3	14	10.6026	91.8715
nsy-1	1.5	3	3	15	94.3050	10.6868
nsy-1	1.5	3	3	16	7.4335	83.4825
nsy-1	1.5	3	3	17	14.9339	172.4177
nsy-1	1.5	3	3	18	31.8597	185.9284
nsy-1	1.5	3	3	19	8.6066	171.8573
nsy-1	1.5	3	3	20	120.0205	16.7060
nsy-1	1.5	3	3	21	25.1551	101.7136
nsy-1	1.5	3	3	22	27.7234	160.3592
nsy-1	1.5	3	3	23	9.1896	107.0971
nsy-1	1.5	3	3	24	9.9123	180.8351
nsy-1	1.5	3	3	25	7.5629	222.1674
nsy-1	1.5	3	4	1	63.2035	31.1311
nsy-1	1.5	3	4	2	15.0360	91.3453
nsy-1	1.5	3	4	3	17.1882	97.3859
nsy-1	1.5	3	4	4	48.0907	166.1707
nsy-1	1.5	3	4	5	11.6998	216.1469
nsy-1	1.5	3	4	6	12.2455	140.8674
nsy-1	1.5	3	4	7	17.8485	130.0347
nsy-1	1.5	3	4	8	8.7661	173.6269
nsy-1	1.5	3	4	9	18.0954	106.1459
nsy-1	1.5	3	4	10	8.2363	144.9151
nsy-1	1.5	3	4	11	13.0866	216.0178
nsy-1	1.5	3	4	12	69.0531	58.0289
nsy-1	1.5	3	4	13	8.9689	193.7503
nsy-1	1.5	3	4	14	10.5316	166.8471
nsy-1	1.5	3	4	15	12.1919	80.6737
nsy-1	1.5	3	4	16	18.4193	151.0811
nsy-1	1.5	3	4	17	21.7148	39.0122
nsy-1	1.5	3	4	18	29.0236	172.0222
nsy-1	1.5	3	4	19	36.5793	41.9039
nsy-1	1.5	3	4	20	14.7049	81.6079
nsy-1	1.5	3	4	21	16.3677	187.4221
nsy-1	1.5	3	4	22	15.2365	116.3521
nsy-1	1.5	3	4	23	7.0459	176.8590
nsy-1	1.5	3	4	24	11.4748	229.4540
nsy-1	1.5	3	4	25	23.6623	35.5514
nsy-1	1.5	3	4	26	56.5375	50.9989
nsy-1	1.5	3	4	27	11.4698	263.2937
nsy-1	1.5	3	4	28	20.8003	101.6010

nsy-1	1.5	3	4	29	81.1849	43.6656
nsy-1	1.5	3	4	30	69.8163	96.9809
nsy-1	3	1	1	1	29.7014	209.1967
nsy-1	3	1	1	2	23.2419	137.7906
nsy-1	3	1	1	3	9.9024	145.0947
nsy-1	3	1	1	4	50.1610	102.8052
nsy-1	3	1	1	5	26.0343	107.7501
nsy-1	3	1	1	6	15.4511	231.5879
nsy-1	3	1	1	7	24.8090	267.6133
nsy-1	3	1	1	8	91.5538	31.1682
nsy-1	3	1	1	9	53.6352	141.9853
nsy-1	3	1	1	10	30.2679	202.4389
nsy-1	3	1	1	11	16.2530	179.2195
nsy-1	3	1	1	12	23.2159	196.7279
nsy-1	3	1	1	13	30.3649	60.1044
nsy-1	3	1	1	14	16.1055	209.4182
nsy-1	3	1	2	1	8.8102	133.2293
nsy-1	3	1	2	2	12.4492	185.9083
nsy-1	3	1	2	3	14.6275	238.2335
nsy-1	3	1	2	4	18.1867	167.2979
nsy-1	3	1	2	5	14.1795	148.5077
nsy-1	3	1	2	6	27.7713	132.3881
nsy-1	3	1	2	7	8.6142	219.8348
nsy-1	3	1	2	8	88.9370	95.8364
nsy-1	3	1	2	9	38.3310	152.2971
nsy-1	3	1	2	10	11.2920	171.9938
nsy-1	3	1	2	11	9.4565	141.3522
nsy-1	3	1	2	12	21.3712	167.0793
nsy-1	3	1	2	13	76.1112	44.7288
nsy-1	3	1	2	14	18.4399	165.6896
nsy-1	3	1	2	15	57.3872	155.9644
nsy-1	3	1	2	16	12.5546	170.5377
nsy-1	3	1	2	17	84.8554	27.7650
nsy-1	3	1	2	18	14.4763	222.5283
nsy-1	3	1	2	19	40.2264	234.8301
nsy-1	3	1	2	20	64.3124	1.4713
nsy-1	3	1	2	21	11.0837	120.8699
nsy-1	3	1	2	22	65.5172	66.5805
nsy-1	3	1	2	23	33.1755	122.4162
nsy-1	3	1	2	24	5.7258	290.3277
nsy-1	3	1	2	25	29.3091	222.8253
nsy-1	3	1	2	26	71.9636	55.9378
nsy-1	3	1	2	27	12.2809	208.1831
nsy-1	3	1	2	28	12.6881	156.5931
nsy-1	3	1	2	29	20.1611	266.5002
nsy-1	3	1	2	30	31.5935	259.4515
nsy-1	3	1	2	31	17.8457	215.6501
nsy-1	3	1	2	32	13.1874	170.2793
nsy-1	3	1	2	33	10.2658	178.9762
nsy-1	3	1	3	1	32.9767	82.2472
nsy-1	3	1	3	2	106.2475	48.0884
nsy-1	3	1	3	3	8.2642	145.9147
nsy-1	3	1	3	4	11.3576	247.3205
nsy-1	3	1	3	5	17.6376	172.9034
nsy-1	3	1	3	6	12.1136	189.3690
nsy-1	3	1	3	7	32.5514	116.5110
nsy-1	3	1	3	8	74.3294	140.6688
nsy-1	3	1	3	9	21.3902	178.9050
nsy-1	3	1	3	10	51.3317	52.2178
nsy-1	3	1	3	11	19.2270	88.5459
nsy-1	3	1	3	12	22.3625	207.9604
nsy-1	3	1	3	13	8.4384	211.0425
nsy-1	3	1	3	14	29.9303	191.2001
nsy-1	3	1	3	15	10.7263	101.6184
nsy-1	3	1	3	16	11.1772	225.2092
nsy-1	3	1	3	17	12.3174	132.3160
nsy-1	3	1	3	18	60.3206	110.0535
nsy-1	3	1	3	19	28.0064	202.9154

nsy-1	3	1	3	20	62.9394	72.3232
nsy-1	3	1	3	21	15.3286	286.5221
nsy-1	3	1	3	22	126.5521	12.8577
nsy-1	3	1	3	23	75.2612	28.0022
nsy-1	3	1	3	24	76.2722	2.0911
nsy-1	3	1	3	25	10.8658	138.5523
nsy-1	3	1	3	26	20.9907	199.1906
nsy-1	3	1	3	27	23.4645	91.9182
nsy-1	3	1	3	28	8.7014	120.6349
nsy-1	3	1	3	29	16.3679	155.4918
nsy-1	3	1	3	30	13.3836	168.8319
nsy-1	3	1	3	31	23.3839	151.0849
nsy-1	3	1	3	32	8.7711	300.5854
nsy-1	3	1	3	33	25.8886	169.4798
nsy-1	3	1	3	34	10.7803	164.2245
nsy-1	3	1	3	35	14.1707	148.4967
nsy-1	3	1	3	36	18.9510	119.3378
nsy-1	3	1	3	37	12.5891	193.6935
nsy-1	3	1	3	38	19.7761	85.5751
nsy-1	3	1	3	39	33.6362	91.5404
nsy-1	3	1	3	40	16.1134	163.3315
nsy-1	3	1	3	41	10.1530	228.3743
nsy-1	3	1	3	42	15.1007	93.5852
nsy-1	3	1	3	43	25.1867	239.9198
nsy-1	3	1	3	44	53.1170	87.8552
nsy-1	3	1	4	1	33.8926	149.4490
nsy-1	3	1	4	2	8.5915	159.7674
nsy-1	3	1	4	3	7.1283	204.3694
nsy-1	3	1	4	4	7.8013	153.1839
nsy-1	3	1	4	5	13.7415	156.1873
nsy-1	3	1	4	6	18.9314	123.2362
nsy-1	3	1	4	7	21.4740	156.8864
nsy-1	3	1	4	8	32.4833	145.3878
nsy-1	3	1	4	9	18.0700	255.8131
nsy-1	3	1	4	10	70.6103	125.8352
nsy-1	3	1	4	11	10.0635	139.4798
nsy-1	3	1	4	12	24.3219	129.6657
nsy-1	3	1	4	13	24.5473	149.2323
nsy-1	3	1	4	14	25.5450	193.3630
nsy-1	3	1	4	15	8.0260	123.3689
nsy-1	3	1	4	16	15.9217	213.6529
nsy-1	3	1	4	17	61.5189	56.2182
nsy-1	3	1	4	18	20.3639	167.6057
nsy-1	3	1	4	19	41.4051	88.8833
nsy-1	3	1	4	20	27.8023	170.0237
nsy-1	3	1	4	21	107.2277	4.5723
nsy-1	3	1	4	22	39.1390	271.6476
nsy-1	3	1	4	23	22.5921	206.7189
nsy-1	3	1	4	24	8.7710	162.0375
nsy-1	3	1	4	25	14.4385	119.1457
nsy-1	3	1	4	26	16.1237	141.0199
nsy-1	3	1	4	27	29.0708	125.6556
nsy-1	3	1	4	28	11.9169	194.6449
nsy-1	3	1	4	29	19.0526	159.3196
nsy-1	3	1	4	30	102.5071	129.4994
nsy-1	3	1	4	31	59.3504	93.2002
nsy-1	3	1	4	32	5.9319	228.1050
nsy-1	3	1	4	33	8.6976	150.9527
nsy-1	3	2	1	1	5.5137	273.2695
nsy-1	3	2	1	2	15.5034	226.8741
nsy-1	3	2	1	3	28.7946	264.0951
nsy-1	3	2	1	4	9.9938	234.5637
nsy-1	3	2	1	5	18.4028	273.4998
nsy-1	3	2	1	6	23.7554	226.0857
nsy-1	3	2	1	7	13.7808	263.7852
nsy-1	3	2	1	8	22.3740	219.0591
nsy-1	3	2	1	9	21.8162	209.0010
nsy-1	3	2	1	10	20.8685	183.9869

nsy-1	3	2	1	11	9.8027	293.8527
nsy-1	3	2	1	12	21.8030	301.7444
nsy-1	3	2	1	13	15.9102	239.1778
nsy-1	3	2	1	14	11.9497	228.0285
nsy-1	3	2	1	15	31.1167	291.9682
nsy-1	3	2	1	16	17.3001	231.4868
nsy-1	3	2	1	17	30.5460	162.6857
nsy-1	3	2	1	18	93.0751	3.8033
nsy-1	3	2	1	19	86.9219	19.0559
nsy-1	3	2	1	20	17.4577	224.8139
nsy-1	3	2	1	21	39.1406	130.4304
nsy-1	3	2	1	22	16.3709	206.8588
nsy-1	3	2	1	23	22.4838	210.8572
nsy-1	3	2	1	24	18.5058	159.2617
nsy-1	3	2	1	25	21.3219	156.6751
nsy-1	3	2	1	26	19.7866	240.2299
nsy-1	3	2	1	27	102.4667	46.8973
nsy-1	3	2	1	28	22.3386	297.1003
nsy-1	3	2	1	29	11.7753	221.9645
nsy-1	3	2	1	30	37.6349	156.0533
nsy-1	3	2	1	31	71.0358	102.6841
nsy-1	3	2	1	32	23.7503	199.5127
nsy-1	3	2	1	33	114.5485	101.6159
nsy-1	3	2	2	1	36.0687	126.8462
nsy-1	3	2	2	2	9.5712	151.3601
nsy-1	3	2	2	3	11.8843	184.1574
nsy-1	3	2	2	4	11.4766	107.2632
nsy-1	3	2	2	5	35.8801	227.4988
nsy-1	3	2	2	6	16.5816	213.8026
nsy-1	3	2	2	7	32.9908	206.5589
nsy-1	3	2	2	8	93.6526	73.0722
nsy-1	3	2	2	9	34.2200	292.1400
nsy-1	3	2	2	10	26.9506	181.2557
nsy-1	3	2	2	11	53.0760	113.5104
nsy-1	3	2	2	12	36.2467	99.7519
nsy-1	3	2	2	13	29.8360	25.1682
nsy-1	3	2	2	14	18.0638	170.8006
nsy-1	3	2	2	15	26.1187	228.8353
nsy-1	3	2	2	16	24.5690	194.6651
nsy-1	3	2	2	17	8.2359	156.5972
nsy-1	3	2	2	18	14.9499	140.9683
nsy-1	3	2	2	19	25.1659	188.9537
nsy-1	3	2	2	20	28.9809	277.7357
nsy-1	3	2	2	21	21.2236	213.1768
nsy-1	3	2	2	22	23.9054	231.8412
nsy-1	3	2	2	23	26.4868	206.2361
nsy-1	3	2	2	24	21.2697	152.1292
nsy-1	3	2	2	25	14.2605	221.7356
nsy-1	3	2	2	26	28.4600	125.3899
nsy-1	3	2	2	27	13.6621	250.7429
nsy-1	3	2	2	28	17.6199	137.7098
nsy-1	3	2	2	29	144.1144	15.4805
nsy-1	3	2	2	30	29.5864	222.9691
nsy-1	3	2	2	31	24.9549	210.7083
nsy-1	3	2	2	32	31.8375	228.7848
nsy-1	3	2	2	33	22.8126	187.8739
nsy-1	3	2	2	34	18.4314	238.6167
nsy-1	3	2	2	35	18.2827	158.6088
nsy-1	3	2	2	36	14.9441	188.0364
nsy-1	3	2	2	37	25.5040	157.8309
nsy-1	3	2	2	38	25.9289	113.6082
nsy-1	3	2	2	39	25.9206	86.8737
nsy-1	3	2	2	40	8.3667	228.1339
nsy-1	3	2	3	1	8.5445	215.9131
nsy-1	3	2	3	2	23.2067	167.2521
nsy-1	3	2	3	3	32.1618	192.3760
nsy-1	3	2	3	4	15.9887	161.1728
nsy-1	3	2	3	5	18.5896	189.0922

nsy-1	3	2	3	6	27.6027	154.8799
nsy-1	3	2	3	7	27.8444	224.7657
nsy-1	3	2	3	8	28.5541	95.5605
nsy-1	3	2	3	9	13.0964	106.5768
nsy-1	3	2	3	10	58.3184	54.7459
nsy-1	3	2	3	11	27.4466	65.6878
nsy-1	3	2	3	12	25.2213	173.5045
nsy-1	3	2	3	13	20.7185	125.5458
nsy-1	3	2	3	14	10.7244	136.8847
nsy-1	3	2	3	15	21.3961	190.2324
nsy-1	3	2	3	16	31.6089	163.3320
nsy-1	3	2	3	17	31.9609	138.1940
nsy-1	3	2	3	18	20.2346	150.4213
nsy-1	3	2	3	19	14.8085	220.0057
nsy-1	3	2	3	20	21.8891	251.0297
nsy-1	3	2	3	21	71.2338	28.8751
nsy-1	3	2	3	22	22.2019	181.9795
nsy-1	3	2	3	23	18.2153	93.3645
nsy-1	3	2	3	24	22.2060	156.5240
nsy-1	3	2	3	25	26.4559	194.1756
nsy-1	3	2	3	26	74.1802	11.6172
nsy-1	3	2	4	1	32.9245	74.8216
nsy-1	3	2	4	2	12.6398	140.9007
nsy-1	3	2	4	3	33.2724	200.0743
nsy-1	3	2	4	4	24.7381	101.3919
nsy-1	3	2	4	5	20.2810	152.6840
nsy-1	3	2	4	6	29.2876	140.7241
nsy-1	3	2	4	7	29.4448	125.5160
nsy-1	3	2	4	8	22.9531	142.7402
nsy-1	3	2	4	9	20.3428	190.1393
nsy-1	3	2	4	10	71.9717	22.4767
nsy-1	3	2	4	11	18.5148	79.3889
nsy-1	3	2	4	12	19.7723	77.9940
nsy-1	3	2	4	13	16.4231	137.1676
nsy-1	3	2	4	14	69.4208	38.3079
nsy-1	3	2	4	15	34.6012	175.8225
nsy-1	3	2	4	16	11.8366	110.7237
nsy-1	3	2	4	17	36.4127	90.9829
nsy-1	3	2	4	18	9.6114	92.4902
nsy-1	3	2	4	19	13.7646	286.0525
nsy-1	3	2	4	20	23.1518	146.6793
nsy-1	3	2	4	21	39.5818	156.0681
nsy-1	3	2	4	22	18.5940	215.6568
nsy-1	3	3	1	1	24.3104	244.3195
nsy-1	3	3	1	2	16.1253	261.9408
nsy-1	3	3	1	3	18.2490	238.5753
nsy-1	3	3	1	4	44.4587	45.3647
nsy-1	3	3	1	5	22.8487	190.3981
nsy-1	3	3	1	6	45.8458	134.2287
nsy-1	3	3	1	7	27.1264	261.0448
nsy-1	3	3	1	8	8.6814	295.1617
nsy-1	3	3	1	9	47.3578	179.2276
nsy-1	3	3	1	10	26.0348	298.7017
nsy-1	3	3	1	11	25.0243	237.6491
nsy-1	3	3	1	12	18.3564	303.2279
nsy-1	3	3	1	13	20.1104	117.5764
nsy-1	3	3	1	14	12.6565	256.2956
nsy-1	3	3	1	15	20.8925	182.8885
nsy-1	3	3	2	1	31.8255	164.7352
nsy-1	3	3	2	2	18.9943	274.8071
nsy-1	3	3	2	3	14.1366	257.0798
nsy-1	3	3	2	4	30.4991	323.0940
nsy-1	3	3	2	5	45.4113	209.8601
nsy-1	3	3	2	6	15.2011	105.1412
nsy-1	3	3	2	7	25.2361	222.9513
nsy-1	3	3	2	8	12.0433	238.0300
nsy-1	3	3	2	9	14.0290	154.7260
nsy-1	3	3	2	10	14.0410	169.0611

nsy-1	3	3	2	11	16.6700	176.1829
nsy-1	3	3	2	12	27.4231	232.4740
nsy-1	3	3	2	13	32.0291	244.4600
nsy-1	3	3	2	14	103.8891	9.1097
nsy-1	3	3	2	15	8.2164	220.1174
nsy-1	3	3	2	16	35.7370	170.8818
nsy-1	3	3	2	17	27.7192	224.9666
nsy-1	3	3	2	18	31.7525	219.4151
nsy-1	3	3	2	19	27.6286	124.6210
nsy-1	3	3	2	20	37.8037	234.9358
nsy-1	3	3	3	1	21.6530	223.0337
nsy-1	3	3	3	2	8.0096	216.9246
nsy-1	3	3	3	3	8.9443	193.3411
nsy-1	3	3	3	4	24.3743	72.8911
nsy-1	3	3	3	5	21.9898	143.9863
nsy-1	3	3	3	6	19.9242	204.2809
nsy-1	3	3	3	7	30.1651	246.8240
nsy-1	3	3	3	8	8.9821	222.5419
nsy-1	3	3	3	9	15.3135	142.7769
nsy-1	3	3	3	10	11.0490	152.1497
nsy-1	3	3	3	11	15.5578	160.4962
nsy-1	3	3	3	12	15.7020	149.4549
nsy-1	3	3	3	13	12.5401	213.7162
nsy-1	3	3	3	14	33.1821	292.4935
nsy-1	3	3	3	15	21.3418	214.6353
nsy-1	3	3	3	16	24.1777	110.1399
nsy-1	3	3	3	17	73.0691	12.9839
nsy-1	3	3	3	18	11.3314	87.1056
nsy-1	3	3	3	19	26.3737	209.5910
nsy-1	3	3	3	20	32.8839	259.0383
nsy-1	3	3	2	21	87.2409	46.1957
nsy-1	3	3	3	22	21.9250	150.6683
nsy-1	3	3	3	23	21.3401	222.3480
nsy-1	3	3	3	24	33.8678	117.5906
nsy-1	3	3	3	25	32.5676	255.2800
nsy-1	3	3	3	26	33.2783	190.8069
nsy-1	3	3	3	27	17.8014	163.7064
nsy-1	3	3	3	28	24.5583	84.8711
nsy-1	3	3	3	29	7.5796	168.6772
nsy-1	3	3	3	30	22.7486	78.8286
nsy-1	3	3	3	31	100.8504	115.9244
nsy-1	3	3	4	1	25.8135	199.0517
nsy-1	3	3	4	2	9.6219	100.6047
nsy-1	3	3	4	3	89.1178	144.2816
nsy-1	3	3	4	4	17.9248	185.8517
nsy-1	3	3	4	5	11.0355	253.9591
nsy-1	3	3	4	6	13.2472	186.6587
nsy-1	3	3	4	7	24.1513	98.1141
nsy-1	3	3	4	8	16.7011	164.2060
nsy-1	3	3	4	9	17.4604	204.3477
nsy-1	3	3	4	10	18.8592	158.2293
nsy-1	3	3	4	11	23.2594	165.3351
nsy-1	3	3	4	12	36.9323	119.4694
nsy-1	3	3	4	13	20.8823	189.8411
nsy-1	3	3	4	14	39.8828	146.5493
nsy-1	3	3	4	15	18.2609	144.1014
nsy-1	3	3	4	16	15.4630	109.7962
nsy-1	3	3	4	17	12.3298	79.9247
nsy-1	3	3	4	18	17.0791	86.8421
nsy-1	3	3	4	19	24.6551	86.7917
nsy-1	3	3	4	20	30.4734	289.8768
nsy-1	3	3	4	21	17.5216	164.4260
nsy-1	3	3	4	22	48.2076	99.2585
nsy-1	3	3	4	23	16.5230	218.7952
nsy-1	3	3	4	24	65.7302	110.0627
nsy-1	3	3	4	25	33.0006	104.3470
nsy-1	3	3	4	26	29.5925	186.1460
nsy-1	6	1	1	1	32.9964	207.5728

nsy-1	6	1	1	2	82.6296	155.9804
nsy-1	6	1	1	3	34.2920	178.5721
nsy-1	6	1	1	4	39.9530	204.0329
nsy-1	6	1	1	5	24.2642	205.6546
nsy-1	6	1	1	6	17.1199	207.7408
nsy-1	6	1	1	7	59.3083	108.9904
nsy-1	6	1	1	8	29.0651	217.3340
nsy-1	6	1	1	9	52.3935	185.7904
nsy-1	6	1	1	10	97.8722	3.1463
nsy-1	6	1	1	11	68.1461	92.5668
nsy-1	6	1	2	1	15.2798	174.8202
nsy-1	6	1	2	2	16.6678	162.0951
nsy-1	6	1	2	3	27.1974	156.6298
nsy-1	6	1	2	4	13.7897	95.1493
nsy-1	6	1	2	5	12.2858	129.2384
nsy-1	6	1	2	6	30.9845	174.4717
nsy-1	6	1	2	7	98.0406	1.1779
nsy-1	6	1	2	8	37.1905	111.6803
nsy-1	6	1	2	9	92.8179	6.0554
nsy-1	6	1	2	10	107.5223	33.5115
nsy-1	6	1	2	11	92.7703	1.8120
nsy-1	6	1	2	12	18.0764	174.9813
nsy-1	6	1	2	13	21.6585	133.0499
nsy-1	6	1	2	14	21.7461	189.1956
nsy-1	6	1	2	15	22.0376	166.4976
nsy-1	6	1	2	16	35.2442	152.1664
nsy-1	6	1	2	17	22.1399	183.8939
nsy-1	6	1	2	18	28.1321	101.5180
nsy-1	6	1	2	19	33.2772	56.8841
nsy-1	6	1	2	20	35.0670	162.1358
nsy-1	6	1	2	21	29.2956	157.2757
nsy-1	6	1	2	22	19.8436	171.9081
nsy-1	6	1	2	23	34.5960	193.4478
nsy-1	6	1	2	24	11.8256	169.6708
nsy-1	6	1	2	25	33.4957	122.1860
nsy-1	6	1	2	26	24.9060	189.5682
nsy-1	6	1	3	1	24.6653	141.8109
nsy-1	6	1	3	2	18.2582	167.0956
nsy-1	6	1	3	3	35.9704	181.9782
nsy-1	6	1	3	4	21.9967	113.2886
nsy-1	6	1	3	5	41.1796	86.3016
nsy-1	6	1	3	6	43.2323	171.4868
nsy-1	6	1	3	7	17.8458	155.0412
nsy-1	6	1	3	8	69.4007	98.7272
nsy-1	6	1	3	9	21.2344	121.7366
nsy-1	6	1	3	10	14.7008	134.3880
nsy-1	6	1	3	11	11.3596	157.3746
nsy-1	6	1	3	12	21.3313	134.1128
nsy-1	6	1	3	13	14.3542	162.7894
nsy-1	6	1	3	14	52.7324	193.2592
nsy-1	6	1	3	15	36.3138	87.8976
nsy-1	6	1	3	16	94.2130	3.6503
nsy-1	6	1	3	17	100.6868	1.6116
nsy-1	6	1	3	18	91.9297	1.6408
nsy-1	6	1	3	19	47.5376	258.6375
nsy-1	6	1	3	20	32.4037	180.5070
nsy-1	6	1	3	21	39.9694	179.1934
nsy-1	6	1	3	22	33.0079	248.2901
nsy-1	6	1	3	23	10.8977	123.7983
nsy-1	6	1	3	24	83.8754	116.6420
nsy-1	6	1	3	25	21.7545	151.1658
nsy-1	6	1	3	26	59.7714	197.5301
nsy-1	6	1	3	27	14.3049	191.2152
nsy-1	6	1	3	28	24.5686	133.6500
nsy-1	6	1	3	29	20.2805	249.3556
nsy-1	6	1	3	30	35.9784	82.8461
nsy-1	6	1	4	1	17.1933	109.1084
nsy-1	6	1	4	2	21.7805	135.8671

nsy-1	6	1	4	3	18.4742	112.7793
nsy-1	6	1	4	4	25.4322	224.2016
nsy-1	6	1	4	5	38.0115	146.0177
nsy-1	6	1	4	6	89.4614	11.3659
nsy-1	6	1	4	7	21.8425	252.1911
nsy-1	6	1	4	8	12.6715	111.5553
nsy-1	6	1	4	9	48.1860	71.0906
nsy-1	6	1	4	10	35.9663	189.4446
nsy-1	6	1	4	11	123.9859	114.0519
nsy-1	6	1	4	12	37.9856	207.7676
nsy-1	6	1	4	13	45.1271	197.0141
nsy-1	6	1	4	14	27.4361	177.0318
nsy-1	6	1	4	15	59.3311	279.4671
nsy-1	6	1	4	16	107.9229	9.3919
nsy-1	6	1	4	17	25.4342	170.0954
nsy-1	6	1	4	18	33.2930	181.4824
nsy-1	6	1	4	19	16.4806	191.4620
nsy-1	6	1	4	20	13.9661	128.5686
nsy-1	6	1	4	21	38.3256	215.4972
nsy-1	6	1	4	22	39.5944	75.4206
nsy-1	6	1	4	23	26.2152	140.0739
nsy-1	6	1	4	24	16.1185	167.4238
nsy-1	6	2	1	1	49.0851	222.2109
nsy-1	6	2	1	2	40.5950	155.4411
nsy-1	6	2	1	3	60.7650	83.0274
nsy-1	6	2	1	4	27.5838	224.9708
nsy-1	6	2	1	5	32.8630	108.0769
nsy-1	6	2	1	6	54.1803	124.5767
nsy-1	6	2	1	7	28.3820	197.7614
nsy-1	6	2	1	8	59.3872	51.2614
nsy-1	6	2	2	1	41.0959	127.9477
nsy-1	6	2	2	2	26.5297	174.5768
nsy-1	6	2	2	3	48.7661	231.2653
nsy-1	6	2	2	4	25.7437	126.2831
nsy-1	6	2	2	5	85.9341	20.0198
nsy-1	6	2	2	6	89.7529	4.2737
nsy-1	6	2	2	7	31.3349	26.0607
nsy-1	6	2	2	8	45.1558	121.4854
nsy-1	6	2	2	9	56.0754	146.5792
nsy-1	6	2	2	10	57.6545	61.4590
nsy-1	6	2	2	11	42.9212	89.3273
nsy-1	6	2	2	12	43.2169	51.9015
nsy-1	6	2	2	13	17.2452	154.1795
nsy-1	6	2	3	1	21.7615	51.1121
nsy-1	6	2	3	2	83.7264	38.1953
nsy-1	6	2	3	3	32.1001	72.4604
nsy-1	6	2	3	4	28.6409	42.0817
nsy-1	6	2	3	5	41.0211	39.2662
nsy-1	6	2	3	6	41.0736	116.9728
nsy-1	6	2	3	7	78.3280	16.2146
nsy-1	6	2	3	8	100.3687	72.0941
nsy-1	6	2	3	9	79.5383	53.7874
nsy-1	6	2	3	10	33.7316	23.3392
nsy-1	6	2	4	1	44.6560	72.6417
nsy-1	6	2	4	2	85.7201	14.6654
nsy-1	6	2	4	3	11.3016	157.2859
nsy-1	6	2	4	4	37.1094	62.8342
nsy-1	6	2	4	5	32.8543	75.1568
nsy-1	6	2	4	6	68.0625	79.3326
nsy-1	6	2	4	7	34.3277	58.5959
nsy-1	6	2	4	8	56.1630	32.6427
nsy-1	6	2	4	9	86.8146	24.2268
nsy-1	6	3	1	1	36.5178	229.8888
nsy-1	6	3	1	2	32.5186	246.5665
nsy-1	6	3	1	3	30.5929	80.1470
nsy-1	6	3	1	4	42.0698	277.2282
nsy-1	6	3	1	5	50.7551	264.6784
nsy-1	6	3	1	6	45.7567	213.0276

nsy-1	6	3	1	7	44.5976	186.2921
nsy-1	6	3	1	8	75.2538	25.9839
nsy-1	6	3	1	9	34.6269	194.9773
nsy-1	6	3	1	10	48.6219	197.9069
nsy-1	6	3	1	11	52.7569	207.8935
nsy-1	6	3	2	1	38.9608	243.5037
nsy-1	6	3	2	2	37.0590	110.9971
nsy-1	6	3	2	3	38.8828	247.4790
nsy-1	6	3	2	4	43.5513	110.9297
nsy-1	6	3	2	5	68.0336	278.1320
nsy-1	6	3	2	6	61.0932	293.6461
nsy-1	6	3	2	7	41.9909	146.7544
nsy-1	6	3	2	8	38.7638	241.8753
nsy-1	6	3	2	9	118.3180	64.3166
nsy-1	6	3	2	10	26.7640	262.5295
nsy-1	6	3	2	11	20.0031	194.5574
nsy-1	6	3	2	12	81.1590	38.9144
nsy-1	6	3	3	1	38.2174	226.7015
nsy-1	6	3	3	2	37.5879	96.8319
nsy-1	6	3	3	3	34.5433	209.4470
nsy-1	6	3	3	4	31.6655	163.6038
nsy-1	6	3	3	5	102.9176	13.6983
nsy-1	6	3	3	6	59.8065	193.5854
nsy-1	6	3	3	7	40.6497	201.6042
nsy-1	6	3	3	8	27.4912	216.9441
nsy-1	6	3	3	9	53.2308	174.8535
nsy-1	6	3	3	10	59.5808	169.5970
nsy-1	6	3	3	11	102.8887	23.1142
nsy-1	6	3	3	12	63.3627	194.1356
nsy-1	6	3	3	13	43.3842	45.4238
nsy-1	6	3	3	14	30.3700	61.3184
nsy-1	6	3	3	15	36.9173	233.0679
nsy-1	6	3	3	16	37.4051	87.2644
nsy-1	6	2	4	1	19.6982	154.3705
nsy-1	6	2	4	2	15.0617	146.0252
nsy-1	6	2	4	3	25.8026	177.1031
nsy-1	6	2	4	4	9.3920	168.0998
nsy-1	6	2	4	5	56.0412	81.9621
nsy-1	6	2	4	6	20.1795	50.6452
nsy-1	6	3	4	7	20.9437	177.9294
nsy-1	6	3	4	8	68.1596	19.5085
nsy-1	6	3	4	9	55.1829	88.1671
nsy-1	6	3	4	10	28.9816	62.3073
nsy-1	6	3	4	11	54.0454	89.4939
nsy-1	6	3	4	12	48.4947	184.4090
nsy-1	6	3	4	13	38.1955	165.3358
nsy-1	6	3	4	14	55.7755	194.1138
nsy-1	6	3	4	15	62.8606	102.6735
nsy-1	6	3	4	16	16.4677	107.2825
ceh-36	0	1	1	1	28.5933	75.7966
ceh-36	0	1	1	2	115.9745	127.6069
ceh-36	0	1	1	3	80.1286	64.3259
ceh-36	0	1	1	4	103.8090	54.8243
ceh-36	0	1	1	5	111.6542	89.4567
ceh-36	0	1	1	6	118.1347	191.0909
ceh-36	0	1	2	1	113.2418	68.9700
ceh-36	0	1	2	2	61.7292	142.2923
ceh-36	0	1	2	3	55.7525	142.1575
ceh-36	0	1	2	4	23.5906	285.2207
ceh-36	0	1	2	5	158.0086	237.5635
ceh-36	0	1	2	6	40.8643	175.3602
ceh-36	0	1	2	7	109.0863	200.5681
ceh-36	0	1	2	8	148.2402	95.8708
ceh-36	0	1	2	9	94.5596	112.6588
ceh-36	0	1	2	10	59.8390	79.8581
ceh-36	0	1	2	11	21.1348	51.1205
ceh-36	0	1	2	12	43.8125	111.8001
ceh-36	0	1	2	13	154.1277	92.6863

ceh-36	0	1	3	1	90.8468	136.2357
ceh-36	0	1	3	2	40.6141	42.2238
ceh-36	0	1	3	3	88.4343	45.5151
ceh-36	0	1	3	4	89.0307	186.9755
ceh-36	0	1	3	5	80.3867	40.9527
ceh-36	0	1	3	6	137.3021	94.9978
ceh-36	0	1	3	7	89.1790	51.9835
ceh-36	0	1	3	8	80.4924	93.2392
ceh-36	0	1	3	9	86.8429	135.3825
ceh-36	0	1	3	10	58.0172	161.0537
ceh-36	0	1	3	11	165.6583	31.6920
ceh-36	0	1	3	12	116.3269	66.0973
ceh-36	0	1	3	13	91.8651	55.1955
ceh-36	0	1	3	14	60.4587	26.0635
ceh-36	0	1	3	15	115.6924	108.1252
ceh-36	0	1	4	1	64.7057	203.3053
ceh-36	0	1	4	2	64.5712	32.7356
ceh-36	0	1	4	3	137.4666	62.0188
ceh-36	0	1	4	4	98.3026	55.7608
ceh-36	0	1	4	5	137.2065	142.5021
ceh-36	0	1	4	6	67.0146	107.8910
ceh-36	0	1	4	7	117.3085	72.9208
ceh-36	0	1	4	8	93.8287	161.9164
ceh-36	0	1	4	9	126.0726	209.3601
ceh-36	0	1	4	10	67.1963	119.3684
ceh-36	0	1	4	11	151.7541	92.6136
ceh-36	0	1	4	12	154.0030	40.2660
ceh-36	0	1	4	13	73.8948	122.8456
ceh-36	0	1	4	14	56.9313	47.0398
ceh-36	0	1	4	15	101.7695	109.2038
ceh-36	0	1	4	16	74.7119	142.9582
ceh-36	0	1	4	17	102.0505	166.2008
ceh-36	0	1	4	18	38.0025	314.0628
ceh-36	0	1	4	19	49.2664	64.6405
ceh-36	0	1	4	20	79.8371	60.2635
ceh-36	0	1	4	21	129.9135	56.7395
ceh-36	0	2	1	1	75.0729	85.9334
ceh-36	0	2	1	2	93.5346	16.5123
ceh-36	0	2	1	3	82.3853	136.6429
ceh-36	0	2	1	4	111.0073	168.7757
ceh-36	0	2	1	5	110.5310	156.4744
ceh-36	0	2	1	6	141.0522	105.0331
ceh-36	0	2	1	7	85.4462	35.6652
ceh-36	0	2	2	1	9.1779	162.9067
ceh-36	0	2	2	2	78.1474	64.2307
ceh-36	0	2	2	3	135.5981	82.3841
ceh-36	0	2	2	4	66.7992	88.1576
ceh-36	0	2	2	5	108.2249	42.5437
ceh-36	0	2	2	6	108.3066	75.3123
ceh-36	0	2	2	7	101.1114	125.9398
ceh-36	0	2	2	8	82.6558	100.6738
ceh-36	0	2	3	1	64.2006	91.3174
ceh-36	0	2	3	2	49.0101	51.2396
ceh-36	0	2	3	3	136.2455	81.1439
ceh-36	0	2	3	4	34.3777	66.7861
ceh-36	0	2	3	5	90.0300	122.3028
ceh-36	0	2	3	6	111.9120	87.5525
ceh-36	0	2	3	7	52.3862	55.8232
ceh-36	0	2	3	8	115.2104	38.6413
ceh-36	0	2	3	9	101.5593	73.2831
ceh-36	0	2	3	10	124.5141	44.7902
ceh-36	0	2	3	11	171.6688	81.0628
ceh-36	0	2	3	12	68.1043	49.5049
ceh-36	0	2	3	13	90.4044	175.4533
ceh-36	0	2	3	14	111.1833	135.9601
ceh-36	0	2	3	15	102.8617	41.6075
ceh-36	0	2	3	16	149.3492	84.2975
ceh-36	0	2	4	1	124.4339	72.1939

ceh-36	0	2	4	2	27.1731	96.7998
ceh-36	0	2	4	3	107.6540	58.0254
ceh-36	0	2	4	4	62.3506	125.6458
ceh-36	0	2	4	5	84.7847	56.3782
ceh-36	0	2	4	6	89.3648	31.4158
ceh-36	0	2	4	7	32.2701	109.6741
ceh-36	0	2	4	8	78.8420	53.5461
ceh-36	0	2	4	9	141.5238	80.0501
ceh-36	0	2	4	10	134.7079	79.8780
ceh-36	0	2	4	11	148.1683	250.0898
ceh-36	0	2	4	12	81.0900	79.3844
ceh-36	0	2	4	13	137.2541	95.1829
ceh-36	0	2	4	14	116.0030	32.3527
ceh-36	0	2	4	15	56.7838	211.0918
ceh-36	0	2	4	16	86.8443	40.3395
ceh-36	0	2	4	17	24.5244	48.2942
ceh-36	0	3	1	1	153.8974	40.4956
ceh-36	0	3	1	2	138.9682	90.0647
ceh-36	0	3	1	3	83.6323	35.8941
ceh-36	0	3	1	4	85.3361	89.8620
ceh-36	0	3	1	5	97.1106	82.7270
ceh-36	0	3	1	6	74.1114	94.8917
ceh-36	0	3	1	7	64.7763	166.0690
ceh-36	0	3	1	8	50.8945	305.8180
ceh-36	0	3	1	9	100.4193	10.6297
ceh-36	0	3	1	10	111.8020	29.7005
ceh-36	0	3	1	11	21.0257	178.2033
ceh-36	0	3	1	12	57.5557	88.6552
ceh-36	0	3	2	1	106.6625	218.4886
ceh-36	0	3	2	2	50.8788	86.3050
ceh-36	0	3	2	3	97.1347	162.5918
ceh-36	0	3	2	4	49.0384	88.3925
ceh-36	0	3	2	5	84.9183	48.3315
ceh-36	0	3	2	6	22.6635	181.4194
ceh-36	0	3	2	7	121.2087	151.7866
ceh-36	0	3	2	8	102.8842	32.2627
ceh-36	0	3	2	9	35.8075	127.7426
ceh-36	0	3	2	10	68.6502	127.2600
ceh-36	0	3	2	11	67.9514	65.3386
ceh-36	0	3	2	12	35.3000	133.8103
ceh-36	0	3	2	13	52.2089	107.4637
ceh-36	0	3	2	14	98.6442	3.1739
ceh-36	0	3	2	15	132.6966	234.8805
ceh-36	0	3	2	16	114.2436	14.7003
ceh-36	0	3	2	17	116.2640	189.2207
ceh-36	0	3	3	1	121.8093	196.2218
ceh-36	0	3	3	2	60.6940	107.1662
ceh-36	0	3	3	3	78.1534	121.1808
ceh-36	0	3	3	4	106.7887	55.7433
ceh-36	0	3	3	5	16.8557	83.1195
ceh-36	0	3	3	6	128.3341	116.0743
ceh-36	0	3	3	7	85.0002	8.5026
ceh-36	0	3	3	8	127.3871	44.1208
ceh-36	0	3	3	9	100.5165	44.7434
ceh-36	0	3	3	10	146.7219	40.5618
ceh-36	0	3	3	11	103.2927	64.6888
ceh-36	0	3	3	12	123.2499	137.4539
ceh-36	0	3	4	1	134.4157	169.0090
ceh-36	0	3	4	2	120.6095	127.8924
ceh-36	0	3	4	3	79.5737	133.5853
ceh-36	0	3	4	4	132.6462	64.9288
ceh-36	0	3	4	5	145.9580	99.9593
ceh-36	0	3	4	6	94.2622	9.9708
ceh-36	0	3	4	7	86.2301	56.9982
ceh-36	0	3	4	8	51.6509	33.3676
ceh-36	0	3	4	9	43.0406	99.5896
ceh-36	0	3	4	10	97.6608	74.5660
ceh-36	0	3	4	11	90.2617	35.5483

ceh-36	0	3	4	12	20.0987	186.4481
ceh-36	0	3	4	13	122.5545	55.7633
ceh-36	0	3	4	14	113.5144	354.1779
ceh-36	0	3	4	15	79.5931	19.8009
ceh-36	0	3	4	16	92.9852	56.8443
ceh-36	0	3	4	17	59.1097	134.7327
ceh-36	0	3	4	18	88.1310	107.7585
ceh-36	0	3	4	19	121.2074	87.5225
ceh-36	1.5	1	1	1	50.8350	93.3751
ceh-36	1.5	1	1	2	24.0318	121.7326
ceh-36	1.5	1	1	3	96.5452	74.9982
ceh-36	1.5	1	1	4	45.0423	28.6989
ceh-36	1.5	1	1	5	124.9333	165.2425
ceh-36	1.5	1	1	6	7.9234	286.7074
ceh-36	1.5	1	1	7	60.3298	133.2134
ceh-36	1.5	1	1	8	63.0583	60.0474
ceh-36	1.5	1	1	9	52.4527	223.9689
ceh-36	1.5	1	1	10	7.5953	205.4144
ceh-36	1.5	1	1	11	28.5701	170.8802
ceh-36	1.5	1	1	12	16.5871	100.8921
ceh-36	1.5	1	1	13	46.0399	55.6801
ceh-36	1.5	1	1	14	10.3584	219.8969
ceh-36	1.5	1	1	15	8.5156	220.9966
ceh-36	1.5	1	1	16	11.8720	135.9221
ceh-36	1.5	1	2	1	3.9533	222.4734
ceh-36	1.5	1	2	2	102.6957	74.4077
ceh-36	1.5	1	2	3	17.6155	133.1461
ceh-36	1.5	1	2	4	19.3974	164.0257
ceh-36	1.5	1	2	5	17.1573	142.8347
ceh-36	1.5	1	2	6	50.2678	107.5064
ceh-36	1.5	1	2	7	53.6519	122.5974
ceh-36	1.5	1	2	8	23.2455	222.4375
ceh-36	1.5	1	2	9	71.1999	123.0491
ceh-36	1.5	1	2	10	123.8501	101.0656
ceh-36	1.5	1	2	11	83.0848	66.5000
ceh-36	1.5	1	2	12	78.1557	26.7417
ceh-36	1.5	1	2	13	28.8707	61.2304
ceh-36	1.5	1	2	14	66.3668	35.5378
ceh-36	1.5	1	2	15	91.2950	106.4038
ceh-36	1.5	1	2	16	87.6577	131.7496
ceh-36	1.5	1	2	17	104.7381	56.9862
ceh-36	1.5	1	2	18	101.9838	4.6291
ceh-36	1.5	1	2	19	61.3372	32.0941
ceh-36	1.5	1	2	20	62.5981	30.2828
ceh-36	1.5	1	2	21	76.5551	20.4976
ceh-36	1.5	1	2	22	52.1439	168.7672
ceh-36	1.5	1	2	23	38.3087	90.3536
ceh-36	1.5	1	3	1	71.3314	167.2894
ceh-36	1.5	1	3	2	7.3574	162.9321
ceh-36	1.5	1	3	3	6.5598	171.1905
ceh-36	1.5	1	3	4	6.8991	220.4632
ceh-36	1.5	1	3	5	107.6952	87.0863
ceh-36	1.5	1	3	6	63.3018	79.2837
ceh-36	1.5	1	3	7	31.9007	147.5340
ceh-36	1.5	1	3	8	56.1358	61.7646
ceh-36	1.5	1	3	9	48.7491	69.6961
ceh-36	1.5	1	3	10	21.7047	98.3569
ceh-36	1.5	1	3	11	22.7522	43.3795
ceh-36	1.5	1	3	12	91.6033	43.8186
ceh-36	1.5	1	3	13	45.3043	72.0886
ceh-36	1.5	1	3	14	51.3099	198.3528
ceh-36	1.5	1	3	15	82.6848	69.0733
ceh-36	1.5	1	3	16	19.6711	129.0762
ceh-36	1.5	1	3	17	94.4108	91.2142
ceh-36	1.5	1	3	18	88.2463	70.0840
ceh-36	1.5	1	3	19	108.2831	50.4607
ceh-36	1.5	1	3	20	29.1747	154.7597
ceh-36	1.5	1	3	21	84.3977	98.4142

ceh-36	1.5	1	3	22	13.1730	44.7216
ceh-36	1.5	1	4	1	28.1382	107.5105
ceh-36	1.5	1	4	2	75.9434	124.2448
ceh-36	1.5	1	4	3	27.3093	30.7574
ceh-36	1.5	1	4	4	65.0871	73.7822
ceh-36	1.5	1	4	5	13.3602	51.3057
ceh-36	1.5	1	4	6	28.3173	101.8520
ceh-36	1.5	1	4	7	22.7621	211.1313
ceh-36	1.5	1	4	8	17.5352	156.8180
ceh-36	1.5	1	4	9	103.2754	48.9258
ceh-36	1.5	1	4	10	14.9998	93.5692
ceh-36	1.5	1	4	11	90.6251	79.8091
ceh-36	1.5	1	4	12	99.7827	112.1890
ceh-36	1.5	1	4	13	29.4576	267.8864
ceh-36	1.5	1	4	14	35.3316	66.3780
ceh-36	1.5	1	4	15	97.3343	92.5052
ceh-36	1.5	1	4	16	68.1237	47.0950
ceh-36	1.5	1	4	17	15.6323	151.7189
ceh-36	1.5	1	4	18	36.7557	141.8009
ceh-36	1.5	1	4	19	86.5677	48.1490
ceh-36	1.5	1	4	20	25.8925	109.2303
ceh-36	1.5	2	1	1	68.2038	156.4382
ceh-36	1.5	2	1	2	6.4639	123.0564
ceh-36	1.5	2	1	3	40.9255	130.9226
ceh-36	1.5	2	1	4	5.3084	205.9385
ceh-36	1.5	2	1	5	36.3796	208.1450
ceh-36	1.5	2	1	6	77.1227	117.7319
ceh-36	1.5	2	1	7	24.1302	204.8768
ceh-36	1.5	2	1	8	12.7306	277.4755
ceh-36	1.5	2	1	9	65.5833	185.4902
ceh-36	1.5	2	1	10	47.9633	120.7402
ceh-36	1.5	2	2	1	17.1473	162.5310
ceh-36	1.5	2	2	2	12.9840	231.4347
ceh-36	1.5	2	2	3	48.2263	132.3851
ceh-36	1.5	2	2	4	66.9405	80.9216
ceh-36	1.5	2	2	5	27.0939	205.4392
ceh-36	1.5	2	2	6	57.6821	214.2009
ceh-36	1.5	2	2	7	12.9399	221.9248
ceh-36	1.5	2	2	8	72.6287	136.2674
ceh-36	1.5	2	2	9	34.5241	131.7177
ceh-36	1.5	2	2	10	51.9908	172.8028
ceh-36	1.5	2	2	11	90.8420	113.9179
ceh-36	1.5	2	2	12	16.1674	151.0724
ceh-36	1.5	2	2	13	21.9420	277.2245
ceh-36	1.5	2	2	14	92.0695	112.8017
ceh-36	1.5	2	3	1	14.2059	119.8616
ceh-36	1.5	2	3	2	105.7363	168.5405
ceh-36	1.5	2	3	3	97.5963	47.3544
ceh-36	1.5	2	3	4	90.6216	47.6658
ceh-36	1.5	2	3	5	12.8294	150.3677
ceh-36	1.5	2	3	6	5.9474	268.8568
ceh-36	1.5	2	3	7	11.2004	287.7687
ceh-36	1.5	2	3	8	83.7445	17.1312
ceh-36	1.5	2	3	9	107.3049	47.8415
ceh-36	1.5	2	3	10	102.9680	5.4166
ceh-36	1.5	2	3	11	104.9628	18.1954
ceh-36	1.5	2	3	12	12.8280	208.4356
ceh-36	1.5	2	3	13	63.4357	105.9920
ceh-36	1.5	2	3	14	25.7261	123.8147
ceh-36	1.5	2	3	15	85.3558	44.3966
ceh-36	1.5	2	3	16	22.5935	41.1132
ceh-36	1.5	2	3	17	92.9425	216.9543
ceh-36	1.5	2	3	18	83.3209	65.5203
ceh-36	1.5	2	3	19	22.7461	75.9968
ceh-36	1.5	2	3	20	6.8090	281.2752
ceh-36	1.5	2	3	21	8.7469	157.7899
ceh-36	1.5	2	4	1	18.4580	246.8019
ceh-36	1.5	2	4	2	6.8364	190.7552

ceh-36	1.5	2	4	3	17.6239	184.0136
ceh-36	1.5	2	4	4	21.0634	86.1670
ceh-36	1.5	2	4	5	46.6046	98.5809
ceh-36	1.5	2	4	6	17.2511	113.3865
ceh-36	1.5	2	4	7	94.2083	50.9071
ceh-36	1.5	2	4	8	25.9317	45.7526
ceh-36	1.5	2	4	9	81.5357	41.2872
ceh-36	1.5	2	4	10	29.2657	172.9139
ceh-36	1.5	2	4	11	17.9833	28.9837
ceh-36	1.5	2	4	12	26.2721	37.6233
ceh-36	1.5	3	1	1	22.4892	206.2738
ceh-36	1.5	3	1	2	16.2706	239.3441
ceh-36	1.5	3	1	3	4.9222	332.8288
ceh-36	1.5	3	1	4	6.9684	296.2108
ceh-36	1.5	3	1	5	27.3927	232.7398
ceh-36	1.5	3	1	6	71.4750	91.1473
ceh-36	1.5	3	1	7	9.2477	184.8111
ceh-36	1.5	3	1	8	120.1965	88.8975
ceh-36	1.5	3	1	9	16.2745	130.4767
ceh-36	1.5	3	1	10	57.7479	169.7112
ceh-36	1.5	3	1	11	86.9417	44.1642
ceh-36	1.5	3	1	12	88.8126	56.7221
ceh-36	1.5	3	2	1	18.4253	212.4957
ceh-36	1.5	3	2	2	18.7283	223.5484
ceh-36	1.5	3	2	3	11.8964	210.9414
ceh-36	1.5	3	2	4	53.7007	93.3102
ceh-36	1.5	3	2	5	46.6863	150.9817
ceh-36	1.5	3	2	6	86.0020	168.8006
ceh-36	1.5	3	2	7	93.7273	30.9082
ceh-36	1.5	3	2	8	89.5825	59.9905
ceh-36	1.5	3	2	9	8.8830	189.7425
ceh-36	1.5	3	2	10	115.0924	126.5363
ceh-36	1.5	3	2	11	71.9149	62.6853
ceh-36	1.5	3	3	1	73.6099	126.4838
ceh-36	1.5	3	3	2	12.6803	112.0955
ceh-36	1.5	3	3	3	14.5392	189.6605
ceh-36	1.5	3	3	4	80.6354	24.9972
ceh-36	1.5	3	3	5	60.2246	253.6127
ceh-36	1.5	3	3	6	76.1159	157.8908
ceh-36	1.5	3	3	7	79.6449	138.5369
ceh-36	1.5	3	3	8	66.3822	125.1121
ceh-36	1.5	3	3	9	161.5035	45.7244
ceh-36	1.5	3	3	10	89.3865	36.5506
ceh-36	1.5	3	3	11	79.8434	63.3039
ceh-36	1.5	3	4	1	35.2175	96.1597
ceh-36	1.5	3	4	2	80.2456	90.3055
ceh-36	1.5	3	4	3	24.1589	181.0070
ceh-36	1.5	3	4	4	5.1379	309.2099
ceh-36	1.5	3	4	5	74.7376	275.8382
ceh-36	1.5	3	4	6	75.7754	70.3008
ceh-36	1.5	3	4	7	17.4466	155.5274
ceh-36	1.5	3	4	8	81.8100	85.2008
ceh-36	1.5	3	4	9	150.0905	200.1213
ceh-36	1.5	3	4	10	96.3743	9.7063
ceh-36	1.5	3	4	11	150.6487	23.0009
ceh-36	1.5	3	4	12	124.7505	180.1778
ceh-36	1.5	3	4	13	80.1778	26.5219
ceh-36	1.5	3	4	14	53.3413	208.0100
ceh-36	3	1	1	1	126.3043	69.3880
ceh-36	3	1	1	2	124.1825	53.4606
ceh-36	3	1	1	3	91.8433	15.3419
ceh-36	3	1	1	4	14.0587	208.8584
ceh-36	3	1	1	5	156.6182	210.3538
ceh-36	3	1	1	6	11.5111	308.2784
ceh-36	3	1	1	7	96.5124	98.9886
ceh-36	3	1	2	1	17.7347	220.5903
ceh-36	3	1	2	2	23.3544	83.1799
ceh-36	3	1	2	3	46.3372	212.5824

ceh-36	3	1	2	4	12.0441	159.8625
ceh-36	3	1	2	5	23.0529	226.4604
ceh-36	3	1	2	6	14.4112	136.7953
ceh-36	3	1	2	7	17.0050	209.6843
ceh-36	3	1	2	8	10.3875	117.2946
ceh-36	3	1	2	9	94.6945	113.1458
ceh-36	3	1	2	10	89.8577	13.1863
ceh-36	3	1	2	11	78.0029	48.2232
ceh-36	3	1	2	12	95.9320	136.1066
ceh-36	3	1	2	13	9.3713	150.7493
ceh-36	3	1	2	14	46.8550	247.9662
ceh-36	3	1	2	15	30.9421	81.4820
ceh-36	3	1	2	16	33.4930	67.1224
ceh-36	3	1	3	1	32.4568	133.3564
ceh-36	3	1	3	2	27.1309	175.2446
ceh-36	3	1	3	3	18.5705	157.3793
ceh-36	3	1	3	4	17.7995	111.6755
ceh-36	3	1	3	5	83.3797	110.8753
ceh-36	3	1	3	6	6.0440	149.9303
ceh-36	3	1	3	7	53.0731	55.6037
ceh-36	3	1	3	8	105.7887	151.6757
ceh-36	3	1	3	9	47.9455	22.8043
ceh-36	3	1	3	10	77.4542	147.0840
ceh-36	3	1	3	11	84.5003	174.5755
ceh-36	3	1	3	12	18.6105	68.9523
ceh-36	3	1	3	13	21.0841	112.3224
ceh-36	3	1	3	14	41.7167	299.2256
ceh-36	3	1	3	15	68.8378	32.3806
ceh-36	3	1	3	16	90.9179	30.5788
ceh-36	3	1	3	17	34.8691	113.9428
ceh-36	3	1	4	1	34.0423	88.6945
ceh-36	3	1	4	2	15.5248	77.7530
ceh-36	3	1	4	3	36.7308	37.1082
ceh-36	3	1	4	4	66.2757	68.8490
ceh-36	3	1	4	5	50.3916	70.6628
ceh-36	3	1	4	6	75.5852	161.5999
ceh-36	3	1	4	7	41.7488	97.6099
ceh-36	3	1	4	8	92.3899	190.1418
ceh-36	3	1	4	9	103.7423	88.3411
ceh-36	3	1	4	10	94.0762	28.3872
ceh-36	3	1	4	11	86.6133	126.7289
ceh-36	3	1	4	12	92.1231	13.9861
ceh-36	3	1	4	13	34.9642	44.9231
ceh-36	3	1	4	14	21.0233	196.6896
ceh-36	3	1	4	15	87.4373	15.0841
ceh-36	3	1	4	16	41.1491	122.2047
ceh-36	3	1	4	17	38.6504	57.2020
ceh-36	3	2	1	1	16.8521	266.4195
ceh-36	3	2	1	2	103.8438	21.1653
ceh-36	3	2	1	3	17.3973	207.3130
ceh-36	3	2	1	4	99.9581	134.0802
ceh-36	3	2	1	5	40.1356	226.7853
ceh-36	3	2	1	6	57.8487	70.8567
ceh-36	3	2	1	7	13.6413	97.9106
ceh-36	3	2	1	8	70.8214	107.6153
ceh-36	3	2	1	9	27.1243	176.8651
ceh-36	3	2	1	10	101.9073	25.2507
ceh-36	3	2	2	1	22.0135	264.0053
ceh-36	3	2	2	2	16.1280	226.9086
ceh-36	3	2	2	3	22.6644	281.5821
ceh-36	3	2	2	4	15.6394	138.1479
ceh-36	3	2	2	5	23.9983	109.8367
ceh-36	3	2	2	6	8.8331	136.0215
ceh-36	3	2	2	7	16.5626	106.7346
ceh-36	3	2	2	8	32.7835	92.8250
ceh-36	3	2	2	9	46.8479	91.0319
ceh-36	3	2	2	10	26.6182	314.2439
ceh-36	3	2	2	11	20.9434	244.1321

ceh-36	3	2	2	12	19.9814	194.0825
ceh-36	3	2	2	13	9.2005	164.5117
ceh-36	3	2	2	14	19.1661	200.2585
ceh-36	3	2	2	15	100.9586	75.7311
ceh-36	3	2	3	1	13.8784	301.8140
ceh-36	3	2	3	2	12.0067	129.4583
ceh-36	3	2	3	3	18.0948	138.0134
ceh-36	3	2	3	4	15.1996	113.3563
ceh-36	3	2	3	5	25.5750	265.7517
ceh-36	3	2	3	6	9.6752	162.3725
ceh-36	3	2	3	7	7.4903	113.2173
ceh-36	3	2	3	8	16.2566	289.5870
ceh-36	3	2	3	9	23.9691	143.2728
ceh-36	3	2	3	10	31.4401	142.4314
ceh-36	3	2	3	11	19.1113	104.3429
ceh-36	3	2	3	12	144.5285	26.4073
ceh-36	3	2	3	13	132.5173	180.4034
ceh-36	3	2	3	14	82.0169	57.7772
ceh-36	3	2	3	15	12.9054	158.8503
ceh-36	3	2	3	16	38.9731	60.6667
ceh-36	3	2	3	17	42.3767	83.7659
ceh-36	3	2	3	18	13.2144	85.8609
ceh-36	3	2	3	19	35.2057	25.3581
ceh-36	3	2	3	20	98.7283	55.1431
ceh-36	3	2	3	21	51.6594	181.3921
ceh-36	3	2	3	22	122.3067	62.9679
ceh-36	3	2	3	23	55.7805	20.8155
ceh-36	3	2	3	24	12.8336	136.4305
ceh-36	3	2	4	1	25.3357	139.5275
ceh-36	3	2	4	2	37.9295	124.2515
ceh-36	3	2	4	3	6.6799	170.5205
ceh-36	3	2	4	4	11.9891	91.9055
ceh-36	3	2	4	5	14.1768	124.2374
ceh-36	3	2	4	6	27.8691	226.3301
ceh-36	3	2	4	7	83.2731	28.1020
ceh-36	3	2	4	8	105.6407	26.5534
ceh-36	3	2	4	9	107.3202	59.2545
ceh-36	3	2	4	10	38.2222	46.6471
ceh-36	3	2	4	11	93.1892	122.9039
ceh-36	3	2	4	12	44.3459	32.3293
ceh-36	3	2	4	13	32.3360	61.9475
ceh-36	3	2	4	14	89.9211	96.7781
ceh-36	3	2	4	15	92.4982	113.4577
ceh-36	3	2	4	16	43.1378	154.6527
ceh-36	3	2	4	17	15.4921	76.8365
ceh-36	3	2	4	18	12.9130	99.4182
ceh-36	3	2	4	19	93.7285	60.5589
ceh-36	3	2	4	20	33.6438	230.1008
ceh-36	3	2	4	21	43.3892	196.2816
ceh-36	3	2	4	22	21.4719	113.6462
ceh-36	3	3	1	1	70.0165	179.6138
ceh-36	3	3	1	2	5.4462	162.4189
ceh-36	3	3	1	3	16.2458	231.6892
ceh-36	3	3	1	4	88.5368	70.3178
ceh-36	3	3	1	5	21.5978	230.1744
ceh-36	3	3	1	6	49.6607	116.2244
ceh-36	3	3	1	7	13.4845	230.3513
ceh-36	3	3	1	8	59.2244	142.3574
ceh-36	3	3	1	9	32.1310	203.8632
ceh-36	3	3	2	1	26.0925	194.9881
ceh-36	3	3	2	2	13.9142	145.6838
ceh-36	3	3	2	3	8.0689	168.0344
ceh-36	3	3	2	4	7.9025	171.3336
ceh-36	3	3	2	5	52.9217	74.3230
ceh-36	3	3	2	6	13.6174	214.7147
ceh-36	3	3	2	7	22.4718	209.8109
ceh-36	3	3	2	8	17.2568	240.5923
ceh-36	3	3	2	9	81.9087	30.3222

ceh-36	3	3	2	10	25.8760	106.8489
ceh-36	3	3	2	11	11.1581	227.9987
ceh-36	3	3	2	12	11.0299	307.6331
ceh-36	3	3	2	13	8.2843	201.0319
ceh-36	3	3	2	14	16.6083	175.2712
ceh-36	3	3	2	15	21.4887	107.6423
ceh-36	3	3	3	1	31.5951	195.4469
ceh-36	3	3	3	2	23.7825	164.7571
ceh-36	3	3	3	3	14.7715	161.6404
ceh-36	3	3	3	4	9.5894	215.0725
ceh-36	3	3	3	5	4.9614	285.8160
ceh-36	3	3	3	6	52.8998	114.0692
ceh-36	3	3	3	7	13.6370	122.6839
ceh-36	3	3	3	8	16.4957	160.0196
ceh-36	3	3	3	9	12.2356	75.1270
ceh-36	3	3	3	10	32.0187	65.4847
ceh-36	3	3	3	11	83.7022	43.8540
ceh-36	3	3	3	12	15.6992	176.7752
ceh-36	3	3	3	13	14.4475	301.7100
ceh-36	3	3	3	14	16.0625	121.4142
ceh-36	3	3	3	15	8.3725	219.0149
ceh-36	3	3	3	16	18.8790	343.0697
ceh-36	3	3	3	17	16.9573	137.0104
ceh-36	3	3	3	18	60.5327	40.7876
ceh-36	3	3	3	19	4.3972	369.0163
ceh-36	3	3	3	20	20.9580	173.1418
ceh-36	3	3	3	21	10.3918	163.5284
ceh-36	3	3	3	22	22.7898	194.9361
ceh-36	3	3	3	23	40.5108	38.4584
ceh-36	3	3	3	24	77.3622	91.1723
ceh-36	3	3	3	25	72.8556	135.8347
ceh-36	3	3	4	1	35.0400	81.7672
ceh-36	3	3	4	2	94.6208	43.4486
ceh-36	3	3	4	3	27.9130	159.6710
ceh-36	3	3	4	4	11.3234	219.3385
ceh-36	3	3	4	5	85.5800	47.3512
ceh-36	3	3	4	6	54.1262	160.3261
ceh-36	3	3	4	7	18.3184	53.0147
ceh-36	3	3	4	8	22.5399	167.2046
ceh-36	3	3	4	9	25.3008	71.8674
ceh-36	3	3	4	10	56.2320	72.6927
ceh-36	3	3	4	11	26.3099	117.1655
ceh-36	3	3	4	12	42.5142	207.3712
ceh-36	3	3	4	13	32.4779	317.6441
ceh-36	3	3	4	14	24.2228	78.4121
ceh-36	3	3	4	15	38.4029	163.0222
ceh-36	3	3	4	16	28.0627	105.2470
ceh-36	3	3	4	17	31.2625	257.9287
ceh-36	3	3	4	18	81.8997	87.5138
ceh-36	3	3	4	19	7.0664	137.8676
ceh-36	3	3	4	20	50.2232	41.6720
ceh-36	6	1	1	1	29.8549	120.6910
ceh-36	6	1	1	2	39.6088	232.4764
ceh-36	6	1	1	3	29.2606	335.7107
ceh-36	6	1	1	4	45.4026	180.2009
ceh-36	6	1	2	1	73.2145	33.1107
ceh-36	6	1	2	2	95.1893	2.2206
ceh-36	6	1	2	3	65.0068	83.4184
ceh-36	6	1	2	4	27.7797	266.5849
ceh-36	6	1	2	5	12.5577	161.2259
ceh-36	6	1	2	6	38.9065	313.6033
ceh-36	6	1	2	7	69.7716	124.6045
ceh-36	6	1	2	8	46.8414	77.2002
ceh-36	6	1	2	9	40.2814	242.0001
ceh-36	6	1	2	10	8.6562	181.4638
ceh-36	6	1	3	1	60.4283	188.0604
ceh-36	6	1	3	2	69.6991	155.3163
ceh-36	6	1	3	3	86.3436	27.3702

ceh-36	6	1	3	4	44.3153	244.6206
ceh-36	6	1	3	5	90.6039	154.8416
ceh-36	6	1	3	6	78.1137	43.1114
ceh-36	6	1	3	7	44.0555	50.9302
ceh-36	6	1	3	8	65.4089	36.2248
ceh-36	6	1	3	9	98.4641	2.2587
ceh-36	6	1	3	10	45.9001	141.1019
ceh-36	6	1	3	11	44.9718	109.2659
ceh-36	6	1	3	12	33.2100	266.4888
ceh-36	6	1	3	13	85.0156	18.5606
ceh-36	6	1	3	14	86.2687	157.9882
ceh-36	6	1	3	15	77.8774	141.5181
ceh-36	6	1	3	16	64.6299	77.0590
ceh-36	6	1	4	1	60.9836	77.2291
ceh-36	6	1	4	2	47.1358	65.1863
ceh-36	6	1	4	3	54.4138	186.7179
ceh-36	6	1	4	4	48.2822	78.6517
ceh-36	6	1	4	5	93.7320	53.8371
ceh-36	6	1	4	6	73.0015	29.6324
ceh-36	6	1	4	7	52.5302	83.5521
ceh-36	6	1	4	8	85.5979	174.0320
ceh-36	6	1	4	9	101.6100	42.8036
ceh-36	6	1	4	10	69.6234	33.6980
ceh-36	6	1	4	11	58.0498	115.2778
ceh-36	6	1	4	12	90.5980	3.0886
ceh-36	6	1	4	13	72.2847	21.7750
ceh-36	6	1	4	14	63.8389	84.8986
ceh-36	6	1	4	15	43.2718	141.3411
ceh-36	6	1	4	16	25.1879	47.1726
ceh-36	6	1	4	17	34.7000	83.0843
ceh-36	6	1	4	18	86.6698	166.0185
ceh-36	6	2	1	1	42.9706	107.3677
ceh-36	6	2	1	2	47.1752	148.4429
ceh-36	6	2	1	3	86.0454	257.9170
ceh-36	6	2	1	4	58.0009	235.7677
ceh-36	6	2	1	5	37.6795	248.0692
ceh-36	6	2	1	6	53.3038	213.5153
ceh-36	6	2	1	7	43.0061	152.0350
ceh-36	6	2	1	8	21.0055	173.9353
ceh-36	6	2	1	9	27.1744	186.7124
ceh-36	6	2	1	10	29.3854	240.3251
ceh-36	6	2	1	11	87.3190	86.0716
ceh-36	6	2	1	12	41.5101	82.0425
ceh-36	6	2	1	13	27.5966	129.4642
ceh-36	6	2	1	14	35.1667	217.3061
ceh-36	6	2	1	15	30.5852	218.3888
ceh-36	6	2	1	16	55.3934	218.7484
ceh-36	6	2	1	17	97.3273	16.4977
ceh-36	6	2	1	18	51.0679	237.8860
ceh-36	6	2	1	19	34.7012	169.1487
ceh-36	6	2	2	1	27.4355	95.2002
ceh-36	6	2	2	2	47.2059	251.5053
ceh-36	6	2	2	3	43.9452	197.1715
ceh-36	6	2	2	4	38.0301	100.8264
ceh-36	6	2	2	5	58.2323	209.2385
ceh-36	6	2	2	6	49.4338	263.9193
ceh-36	6	2	2	7	82.1061	218.8011
ceh-36	6	2	2	8	16.7916	152.5584
ceh-36	6	2	2	9	40.1723	128.9039
ceh-36	6	2	2	10	55.4603	181.4102
ceh-36	6	2	2	11	28.4671	140.5092
ceh-36	6	2	2	12	50.2513	65.0941
ceh-36	6	2	2	13	52.4754	144.7812
ceh-36	6	2	2	14	31.7921	160.9776
ceh-36	6	2	2	15	63.0050	28.1469
ceh-36	6	2	2	16	74.3051	68.7775
ceh-36	6	2	2	17	93.4947	137.4332
ceh-36	6	2	2	18	54.6608	294.6437

ceh-36	6	2	2	19	51.3141	256.2355
ceh-36	6	2	2	20	83.2446	164.3290
ceh-36	6	2	2	21	59.3103	209.5111
ceh-36	6	2	2	22	16.7642	243.6789
ceh-36	6	2	3	1	43.8758	57.6027
ceh-36	6	2	3	2	43.8730	174.3849
ceh-36	6	2	3	3	38.4170	63.6262
ceh-36	6	2	3	4	34.3751	182.4745
ceh-36	6	2	3	5	13.9708	179.0314
ceh-36	6	2	3	6	46.5195	224.2648
ceh-36	6	2	3	7	27.4552	208.0546
ceh-36	6	2	3	8	40.6024	163.6330
ceh-36	6	2	3	9	33.6061	96.6028
ceh-36	6	2	3	10	45.3755	34.9512
ceh-36	6	2	3	11	35.6300	160.2521
ceh-36	6	2	3	12	36.4497	66.3565
ceh-36	6	2	3	13	80.2265	104.1577
ceh-36	6	2	3	14	77.5691	102.4259
ceh-36	6	2	3	15	38.9289	120.5878
ceh-36	6	2	3	16	73.2349	196.3082
ceh-36	6	2	3	17	18.0991	103.7460
ceh-36	6	2	3	18	78.4974	229.1755
ceh-36	6	2	3	19	55.1781	114.3826
ceh-36	6	2	3	20	51.9878	59.8620
ceh-36	6	2	3	21	59.6495	207.3162
ceh-36	6	2	3	22	76.7692	157.2521
ceh-36	6	2	3	23	39.8680	238.8876
ceh-36	6	2	3	24	71.7101	145.3146
ceh-36	6	2	3	25	68.5984	67.1363
ceh-36	6	2	4	1	67.8637	22.8647
ceh-36	6	2	4	2	57.3830	74.3844
ceh-36	6	2	4	3	81.2395	56.7236
ceh-36	6	2	4	4	71.5049	140.2827
ceh-36	6	2	4	5	71.3798	58.5051
ceh-36	6	2	4	6	55.3235	263.8947
ceh-36	6	2	4	7	70.9264	112.4490
ceh-36	6	2	4	8	49.0744	47.4820
ceh-36	6	2	4	9	62.6576	26.8558
ceh-36	6	2	4	10	80.4080	226.4622
ceh-36	6	2	4	11	73.8846	47.1817
ceh-36	6	2	4	12	80.3865	104.5944
ceh-36	6	2	4	13	85.7093	133.3270
ceh-36	6	2	4	14	85.1862	21.9030
ceh-36	6	2	4	15	24.4926	51.7778
ceh-36	6	2	4	16	49.6033	224.1940
ceh-36	6	2	4	17	60.6782	132.7431
ceh-36	6	2	3	18	70.0297	183.5500
ceh-36	6	2	4	19	27.2235	86.8287
ceh-36	6	2	4	20	80.7755	165.7991
ceh-36	6	2	4	21	58.3777	28.5375
ceh-36	6	2	3	22	65.0411	152.3695
ceh-36	6	3	1	1	17.5661	143.7351
ceh-36	6	3	1	2	35.6438	220.8388
ceh-36	6	3	1	3	50.3181	202.6694
ceh-36	6	3	1	4	32.8348	276.4632
ceh-36	6	3	1	5	67.2790	148.9751
ceh-36	6	3	1	6	18.0098	77.7863
ceh-36	6	3	1	7	35.0706	204.4768
ceh-36	6	3	2	1	25.3773	263.2125
ceh-36	6	3	2	2	26.5143	182.4605
ceh-36	6	3	2	3	12.8713	165.2557
ceh-36	6	3	2	4	27.8479	171.8201
ceh-36	6	3	2	5	26.1998	197.8983
ceh-36	6	3	2	6	68.8681	194.7676
ceh-36	6	3	2	7	44.0815	147.0465
ceh-36	6	3	2	8	36.3332	320.0847
ceh-36	6	3	2	9	74.0173	102.4265
ceh-36	6	3	2	10	9.4292	244.0875

ceh-36	6	3	2	11	38.7278	115.2516
ceh-36	6	3	2	12	77.9150	110.9746
ceh-36	6	3	2	13	26.7271	191.5560
ceh-36	6	3	2	14	34.7376	200.3560
ceh-36	6	3	2	15	61.4515	222.0856
ceh-36	6	3	2	16	40.7009	182.6491
ceh-36	6	3	2	17	103.4577	75.8372
ceh-36	6	3	2	18	50.9175	159.1463
ceh-36	6	3	3	1	10.4380	150.5182
ceh-36	6	3	3	2	27.0740	136.3664
ceh-36	6	3	3	3	39.4095	175.6746
ceh-36	6	3	3	4	49.9594	325.4831
ceh-36	6	3	3	5	38.2302	174.7591
ceh-36	6	3	3	6	15.1950	169.0700
ceh-36	6	3	3	7	65.7571	87.9754
ceh-36	6	3	3	8	88.0206	137.8749
ceh-36	6	3	3	9	53.7859	91.1685
ceh-36	6	3	3	10	44.0391	42.9326
ceh-36	6	3	3	11	10.6371	76.3408
ceh-36	6	3	3	12	127.8159	71.7207
ceh-36	6	3	3	13	89.0522	138.4651
ceh-36	6	3	3	14	33.5311	96.8979
ceh-36	6	3	3	15	53.7341	96.3857
ceh-36	6	3	3	16	12.2816	95.2106
ceh-36	6	3	3	17	66.3639	131.4272
ceh-36	6	3	3	18	39.4606	155.8917
ceh-36	6	3	3	19	92.0432	181.9844
ceh-36	6	3	3	20	45.9095	337.7604
ceh-36	6	3	3	21	93.6809	137.9327
ceh-36	6	3	3	22	44.9227	53.7792
ceh-36	6	3	3	23	11.0766	293.8098
ceh-36	6	3	4	1	48.3280	161.9340
ceh-36	6	3	4	2	25.7209	166.2283
ceh-36	6	3	4	3	49.9341	79.6907
ceh-36	6	3	4	4	37.8624	114.8115
ceh-36	6	3	4	5	94.5643	146.4227
ceh-36	6	3	4	6	48.4442	88.3740
ceh-36	6	3	4	7	52.2490	85.5681
ceh-36	6	3	4	8	40.5460	66.1256
ceh-36	6	3	4	9	92.6450	104.0470
ceh-36	6	3	4	10	47.3906	104.0889
ceh-36	6	3	4	11	65.7522	75.9910
ceh-36	6	3	4	12	88.8152	144.7201
ceh-36	6	3	4	13	13.0358	177.7333
ceh-36	6	3	4	14	27.6742	179.3353
ceh-36	6	3	4	15	52.1040	124.5560
ceh-36	6	3	4	16	8.9594	63.4770
ceh-36	6	3	4	17	115.6193	66.4015
eat-4	0	1	1	1	79.7637	88.4019
eat-4	0	1	1	2	106.9998	76.1004
eat-4	0	1	1	3	106.2849	6.1474
eat-4	0	1	1	4	58.9419	72.8882
eat-4	0	1	1	5	45.2672	92.1744
eat-4	0	1	1	6	146.1731	69.2023
eat-4	0	1	1	7	87.7629	27.3594
eat-4	0	1	1	8	135.5072	131.9936
eat-4	0	1	1	9	9.9740	49.6841
eat-4	0	1	1	10	102.6385	46.1205
eat-4	0	1	1	11	135.6726	67.4173
eat-4	0	1	1	12	111.8683	64.5375
eat-4	0	1	1	13	158.3837	80.8357
eat-4	0	1	1	14	169.2196	133.0035
eat-4	0	1	1	15	15.8677	139.0306
eat-4	0	1	1	16	14.1280	35.4839
eat-4	0	1	1	17	24.6972	47.7261
eat-4	0	1	1	18	79.7664	8.1597
eat-4	0	1	1	19	139.7139	86.1595
eat-4	0	1	1	20	169.7334	159.7602

eat-4	0	1	1	21	67.0980	54.1004
eat-4	0	1	1	22	36.2535	29.4295
eat-4	0	1	1	23	143.0814	175.8097
eat-4	0	1	1	24	118.1194	25.4129
eat-4	0	1	1	25	73.8892	15.6527
eat-4	0	1	1	26	153.7185	52.0964
eat-4	0	1	1	27	117.6034	94.4004
eat-4	0	1	1	28	155.1886	89.0325
eat-4	0	1	1	29	21.0721	121.8734
eat-4	0	1	2	1	47.9452	114.4947
eat-4	0	1	2	2	34.7157	51.4123
eat-4	0	1	2	3	69.2703	79.1397
eat-4	0	1	2	4	50.3581	40.5291
eat-4	0	1	2	5	57.9648	103.4832
eat-4	0	1	2	6	58.8641	54.5762
eat-4	0	1	2	7	96.3957	2.8099
eat-4	0	1	2	8	47.3660	63.5326
eat-4	0	1	2	9	57.4655	52.8820
eat-4	0	1	2	10	63.8391	26.9282
eat-4	0	1	2	11	84.2746	34.2649
eat-4	0	1	2	12	37.7344	108.4086
eat-4	0	1	2	13	23.3289	92.7784
eat-4	0	1	2	14	140.0273	69.9587
eat-4	0	1	2	15	17.7461	104.3980
eat-4	0	1	2	16	87.1690	159.8964
eat-4	0	1	2	17	114.6210	57.7562
eat-4	0	1	2	18	25.4509	71.0858
eat-4	0	1	2	19	47.1225	81.0023
eat-4	0	1	2	20	138.3901	50.2376
eat-4	0	1	2	21	164.0752	73.4034
eat-4	0	1	2	22	87.0240	49.9876
eat-4	0	1	2	23	154.1858	79.8627
eat-4	0	1	2	24	132.6454	134.8381
eat-4	0	1	2	25	120.5618	80.0571
eat-4	0	1	2	26	64.7681	32.2805
eat-4	0	1	2	27	71.5183	103.2319
eat-4	0	1	2	28	146.7575	65.4594
eat-4	0	1	2	29	41.2056	33.4342
eat-4	0	1	2	30	91.4448	46.4372
eat-4	0	1	2	31	143.6692	20.3288
eat-4	0	1	2	32	87.9671	16.3936
eat-4	0	1	2	33	96.1946	44.0724
eat-4	0	1	2	34	126.1026	53.5632
eat-4	0	1	2	35	20.2527	132.3405
eat-4	0	1	2	36	140.9261	125.6019
eat-4	0	1	3	1	22.3912	48.9296
eat-4	0	1	3	2	169.4034	54.7049
eat-4	0	1	3	3	101.3320	55.1466
eat-4	0	1	3	4	124.4988	66.9611
eat-4	0	1	3	5	102.3847	31.9802
eat-4	0	1	3	6	18.9408	44.6752
eat-4	0	1	3	7	142.3516	125.1071
eat-4	0	1	3	8	118.8397	23.8335
eat-4	0	1	3	9	57.4260	26.8900
eat-4	0	1	3	10	15.9920	34.1878
eat-4	0	1	3	11	143.2359	85.1655
eat-4	0	1	3	12	106.0143	80.7302
eat-4	0	1	3	13	108.4820	9.8760
eat-4	0	1	3	14	140.9434	153.9855
eat-4	0	1	3	15	20.5892	49.3763
eat-4	0	1	3	16	38.8216	57.6324
eat-4	0	1	3	17	52.9509	34.4909
eat-4	0	1	3	18	18.1621	39.9612
eat-4	0	1	3	19	57.4323	77.4135
eat-4	0	1	3	20	88.5786	92.0394
eat-4	0	1	3	21	95.5896	3.1932
eat-4	0	1	3	22	115.6913	69.7555
eat-4	0	1	3	23	54.4691	57.1119

eat-4	0	1	3	24	105.8931	32.5919
eat-4	0	1	3	25	99.5819	72.2679
eat-4	0	1	3	26	76.0916	49.4022
eat-4	0	1	3	27	41.3444	79.1869
eat-4	0	1	3	28	70.6310	29.1964
eat-4	0	1	3	29	113.2875	38.5290
eat-4	0	1	3	30	153.1146	72.1253
eat-4	0	1	3	31	29.4471	54.7896
eat-4	0	1	3	32	103.8458	8.8707
eat-4	0	1	3	33	102.0744	17.6463
eat-4	0	1	3	34	163.5328	118.2800
eat-4	0	1	3	35	20.2239	46.0378
eat-4	0	1	3	36	94.3994	34.8196
eat-4	0	1	4	1	71.8855	57.0027
eat-4	0	1	4	2	110.6465	7.3086
eat-4	0	1	4	3	102.3165	20.0845
eat-4	0	1	4	4	106.6640	49.0499
eat-4	0	1	4	5	144.7611	72.9911
eat-4	0	1	4	6	69.8138	23.8296
eat-4	0	1	4	7	14.6979	30.3073
eat-4	0	1	4	8	48.5775	89.9669
eat-4	0	1	4	9	36.5527	66.1057
eat-4	0	1	4	10	92.0477	1.5889
eat-4	0	1	4	11	73.9179	15.7859
eat-4	0	1	4	12	65.4694	58.4550
eat-4	0	1	4	13	165.0784	151.2175
eat-4	0	1	4	14	109.2073	21.3029
eat-4	0	1	4	15	74.3438	92.6087
eat-4	0	1	4	16	129.7839	42.7333
eat-4	0	1	4	17	117.9912	47.5383
eat-4	0	1	4	18	32.9992	22.0586
eat-4	0	1	4	19	88.1305	40.4819
eat-4	0	1	4	20	128.4527	76.0449
eat-4	0	1	4	21	94.4030	83.3418
eat-4	0	1	4	22	121.2743	85.9130
eat-4	0	1	4	23	48.7735	21.4931
eat-4	0	1	4	24	18.8108	159.9561
eat-4	0	1	4	25	25.1872	67.7046
eat-4	0	1	4	26	127.6260	16.8250
eat-4	0	1	4	27	107.2369	28.9583
eat-4	0	1	4	28	84.6459	70.3191
eat-4	0	1	4	29	100.3751	25.4171
eat-4	0	1	4	30	66.9637	38.2481
eat-4	0	2	1	1	79.0782	122.7416
eat-4	0	2	1	2	30.3014	92.1548
eat-4	0	2	1	3	42.8000	139.4826
eat-4	0	2	1	4	86.8167	88.2933
eat-4	0	2	1	5	124.4242	33.2367
eat-4	0	2	1	6	43.5393	120.2210
eat-4	0	2	1	7	82.2291	87.1037
eat-4	0	2	1	8	171.9591	141.4239
eat-4	0	2	1	9	26.3129	147.2266
eat-4	0	2	1	10	141.9521	110.7893
eat-4	0	2	1	11	173.3013	100.7852
eat-4	0	2	1	12	65.2444	87.6500
eat-4	0	2	1	13	122.5266	96.6855
eat-4	0	2	1	14	131.2221	87.4041
eat-4	0	2	1	15	163.4444	78.6649
eat-4	0	2	1	16	161.4227	113.3197
eat-4	0	2	1	17	31.8880	97.8607
eat-4	0	2	1	18	34.5767	138.2017
eat-4	0	2	1	19	163.3777	72.9792
eat-4	0	2	1	20	97.2294	79.4922
eat-4	0	2	1	21	156.7975	69.4888
eat-4	0	2	1	22	85.4248	222.9846
eat-4	0	2	1	23	83.6285	74.7587
eat-4	0	2	1	24	16.2162	205.8710
eat-4	0	2	1	25	15.6518	114.0851

eat-4	0	2	1	26	54.9940	39.1785
eat-4	0	2	1	27	26.7294	56.5953
eat-4	0	2	1	28	105.9554	92.5760
eat-4	0	2	1	29	61.4145	110.2040
eat-4	0	2	1	30	39.7739	44.1691
eat-4	0	2	1	31	154.9143	78.0391
eat-4	0	2	1	32	50.1671	104.7632
eat-4	0	2	1	33	63.1208	73.3482
eat-4	0	2	1	34	173.6114	180.8952
eat-4	0	2	1	35	128.6781	116.7056
eat-4	0	2	1	36	109.0187	72.4413
eat-4	0	2	1	37	141.8419	109.8003
eat-4	0	2	1	38	53.0581	52.4255
eat-4	0	2	2	1	89.0154	117.4986
eat-4	0	2	2	2	63.0066	138.6571
eat-4	0	2	2	3	49.7055	51.8090
eat-4	0	2	2	4	67.1778	110.7937
eat-4	0	2	2	5	52.3952	105.8954
eat-4	0	2	2	6	76.0175	162.1804
eat-4	0	2	2	7	91.1180	53.9491
eat-4	0	2	2	8	99.4916	80.6113
eat-4	0	2	2	9	22.6175	104.6894
eat-4	0	2	2	10	60.8219	169.9784
eat-4	0	2	2	11	30.5442	37.1461
eat-4	0	2	2	12	95.3568	23.0296
eat-4	0	2	2	13	24.9140	64.7924
eat-4	0	2	2	14	122.6256	70.3844
eat-4	0	2	2	15	29.4425	109.3867
eat-4	0	2	2	16	157.7400	127.0688
eat-4	0	2	2	17	106.8615	49.3799
eat-4	0	2	2	18	104.3100	106.9658
eat-4	0	2	2	19	136.9238	92.9866
eat-4	0	2	2	20	163.5008	100.0652
eat-4	0	2	2	21	67.5675	70.4104
eat-4	0	2	2	22	121.3069	86.0674
eat-4	0	2	2	23	125.8040	38.5823
eat-4	0	2	2	24	145.0231	119.7705
eat-4	0	2	2	25	27.9139	54.1880
eat-4	0	2	2	26	161.2896	133.6047
eat-4	0	2	2	27	9.0929	159.4838
eat-4	0	2	2	28	136.1493	100.8984
eat-4	0	2	2	29	110.2561	32.0963
eat-4	0	2	2	30	85.5269	48.0597
eat-4	0	2	2	31	85.3610	69.6968
eat-4	0	2	2	32	83.5067	113.4882
eat-4	0	2	2	33	120.2400	20.3803
eat-4	0	2	2	34	132.1818	109.7524
eat-4	0	2	2	35	149.7950	54.4454
eat-4	0	2	2	36	41.9909	37.8985
eat-4	0	2	2	37	83.5384	124.4088
eat-4	0	2	2	38	113.4252	184.6183
eat-4	0	2	2	39	58.4303	82.5422
eat-4	0	2	2	40	55.1044	65.4683
eat-4	0	2	2	41	24.7790	129.0527
eat-4	0	2	2	42	42.9249	89.7942
eat-4	0	2	3	1	31.3261	39.4507
eat-4	0	2	3	2	49.6273	61.1418
eat-4	0	2	3	3	55.8920	81.1484
eat-4	0	2	3	4	37.1816	66.1216
eat-4	0	2	3	5	36.2624	125.1648
eat-4	0	2	3	6	119.4466	90.8778
eat-4	0	2	3	7	58.4700	79.1513
eat-4	0	2	3	8	82.4944	128.1778
eat-4	0	2	3	9	135.6091	55.6543
eat-4	0	2	3	10	63.6896	59.1345
eat-4	0	2	3	11	40.5735	37.8639
eat-4	0	2	3	12	66.8002	76.9224
eat-4	0	2	3	13	148.0636	93.3566

eat-4	0	2	3	14	23.7944	57.2371
eat-4	0	2	3	15	65.6845	108.2518
eat-4	0	2	3	16	88.5833	72.5418
eat-4	0	2	3	17	147.3153	66.9369
eat-4	0	2	3	18	76.1210	93.8968
eat-4	0	2	3	19	32.8998	97.9910
eat-4	0	2	3	20	21.1486	95.5074
eat-4	0	2	3	21	166.7078	67.1805
eat-4	0	2	3	22	116.2008	170.1703
eat-4	0	2	3	23	150.9946	148.1395
eat-4	0	2	3	24	18.9394	69.3629
eat-4	0	2	3	25	95.5814	109.0906
eat-4	0	2	3	26	10.5877	113.9609
eat-4	0	2	3	27	146.9400	93.0717
eat-4	0	2	3	28	26.7281	128.3869
eat-4	0	2	3	29	133.3304	55.5645
eat-4	0	2	3	30	35.1459	27.1828
eat-4	0	2	3	31	124.4037	92.1606
eat-4	0	2	3	32	171.9374	88.7735
eat-4	0	2	3	33	102.7217	110.5792
eat-4	0	2	3	34	162.4099	117.3200
eat-4	0	2	3	35	124.9405	60.1038
eat-4	0	2	3	36	164.6193	156.9597
eat-4	0	2	3	37	120.9511	125.3404
eat-4	0	2	3	38	152.4877	120.0069
eat-4	0	2	4	1	52.2425	46.1800
eat-4	0	2	4	2	136.0425	63.2503
eat-4	0	2	4	3	109.6031	43.4367
eat-4	0	2	4	4	48.0005	90.2434
eat-4	0	2	4	5	49.2333	93.4701
eat-4	0	2	4	6	124.9342	42.9848
eat-4	0	2	4	7	34.8064	61.4370
eat-4	0	2	4	8	42.2781	62.5188
eat-4	0	2	4	9	101.5373	65.9605
eat-4	0	2	4	10	101.7880	81.3209
eat-4	0	2	4	11	66.8126	58.4303
eat-4	0	2	4	12	47.0819	41.7814
eat-4	0	2	4	13	138.6295	127.8971
eat-4	0	2	4	14	62.5114	143.3239
eat-4	0	2	4	15	153.4025	80.0674
eat-4	0	2	4	16	97.1995	83.4597
eat-4	0	2	4	17	126.8003	98.4247
eat-4	0	2	4	18	53.4490	91.8352
eat-4	0	2	4	19	45.9395	67.2720
eat-4	0	2	4	20	113.1039	43.3951
eat-4	0	2	4	21	60.4436	95.0217
eat-4	0	2	4	22	18.0988	50.9341
eat-4	0	2	4	23	112.3112	49.8555
eat-4	0	2	4	24	100.3184	72.1410
eat-4	0	2	4	25	102.5434	52.9625
eat-4	0	2	4	26	151.1861	119.5755
eat-4	0	2	4	27	38.0201	86.0034
eat-4	0	2	4	28	154.2478	112.6766
eat-4	0	2	4	29	109.7513	65.8789
eat-4	0	2	4	30	149.8298	55.7960
eat-4	0	2	4	31	123.7578	24.4332
eat-4	0	2	4	32	53.0748	55.2053
eat-4	0	2	4	33	109.4834	84.2890
eat-4	0	2	4	34	59.9809	86.1957
eat-4	0	2	4	35	53.1529	53.8928
eat-4	0	2	4	36	75.5684	93.5479
eat-4	0	3	1	1	27.0150	78.7156
eat-4	0	3	1	2	55.7881	62.1034
eat-4	0	3	1	3	39.1896	128.2346
eat-4	0	3	1	4	64.1099	222.6661
eat-4	0	3	1	5	89.5309	219.5595
eat-4	0	3	1	6	161.7748	150.3302
eat-4	0	3	1	7	66.8106	58.8005

eat-4	0	3	1	8	78.1042	81.0624
eat-4	0	3	1	9	129.6134	214.3762
eat-4	0	3	1	10	79.3728	148.6686
eat-4	0	3	1	11	101.5999	44.4970
eat-4	0	3	1	12	144.8116	92.4836
eat-4	0	3	1	13	80.7557	98.1673
eat-4	0	3	1	14	59.8303	66.0873
eat-4	0	3	1	15	32.4913	104.8389
eat-4	0	3	1	16	117.7228	115.4903
eat-4	0	3	1	17	60.6406	122.7138
eat-4	0	3	1	18	6.5773	169.4165
eat-4	0	3	1	19	14.0441	61.4496
eat-4	0	3	1	20	59.4842	26.3590
eat-4	0	3	1	21	172.7393	141.6932
eat-4	0	3	2	1	21.0084	89.1377
eat-4	0	3	2	2	80.1866	57.4779
eat-4	0	3	2	3	86.5584	73.1788
eat-4	0	3	2	4	71.7597	76.7317
eat-4	0	3	2	5	91.6413	56.3499
eat-4	0	3	2	6	67.4854	37.6367
eat-4	0	3	2	7	143.2616	51.3518
eat-4	0	3	2	8	118.2830	35.2970
eat-4	0	3	2	9	65.6785	36.0642
eat-4	0	3	2	10	154.0689	62.5951
eat-4	0	3	2	11	112.2311	93.1901
eat-4	0	3	2	12	101.0537	46.6248
eat-4	0	3	2	13	88.0430	58.3370
eat-4	0	3	2	14	92.5261	110.5822
eat-4	0	3	2	15	152.1434	100.9541
eat-4	0	3	2	16	66.7301	116.8627
eat-4	0	3	2	17	22.4544	58.6612
eat-4	0	3	2	18	126.8502	31.8832
eat-4	0	3	2	19	68.5713	19.4402
eat-4	0	3	2	20	112.0833	155.8680
eat-4	0	3	2	21	80.4585	61.8964
eat-4	0	3	2	22	58.8948	251.3424
eat-4	0	3	2	23	158.0824	111.1207
eat-4	0	3	2	24	123.4935	27.6213
eat-4	0	3	2	25	90.0556	108.4426
eat-4	0	3	2	26	51.1946	62.9191
eat-4	0	3	2	27	84.4750	128.9870
eat-4	0	3	2	28	119.8157	79.0205
eat-4	0	3	2	29	95.8382	53.0413
eat-4	0	3	2	30	143.3211	155.4046
eat-4	0	3	2	31	93.0538	61.4892
eat-4	0	3	2	32	122.4867	40.8071
eat-4	0	3	2	33	88.9403	93.0579
eat-4	0	3	3	1	67.7637	135.7904
eat-4	0	3	3	2	63.8083	40.5920
eat-4	0	3	3	3	63.7051	67.6453
eat-4	0	3	3	4	136.4154	126.3558
eat-4	0	3	3	5	11.2392	55.9171
eat-4	0	3	3	6	66.9835	67.3515
eat-4	0	3	3	7	97.3991	116.2110
eat-4	0	3	3	8	61.4512	113.6048
eat-4	0	3	3	9	30.5322	103.8175
eat-4	0	3	3	10	74.4749	47.3583
eat-4	0	3	3	11	58.5491	50.3874
eat-4	0	3	3	12	72.9171	29.3853
eat-4	0	3	3	13	108.4527	107.3284
eat-4	0	3	3	14	120.4320	65.7957
eat-4	0	3	3	15	161.5085	117.6717
eat-4	0	3	3	16	42.4635	73.8985
eat-4	0	3	3	17	78.0022	17.3047
eat-4	0	3	3	18	51.6106	85.0563
eat-4	0	3	3	19	9.5423	95.1769
eat-4	0	3	3	20	126.5102	70.4307
eat-4	0	3	3	21	30.1918	164.6192

eat-4	0	3	3	22	81.0116	82.7938
eat-4	0	3	3	23	67.5505	212.8193
eat-4	0	3	3	24	69.4744	75.6130
eat-4	0	3	3	25	88.2009	49.3681
eat-4	0	3	3	26	114.9637	78.0305
eat-4	0	3	3	27	30.3663	62.8464
eat-4	0	3	3	28	12.8486	48.3215
eat-4	0	3	3	29	131.9514	84.6033
eat-4	0	3	3	1	51.3000	15.8905
eat-4	0	3	3	2	54.3749	50.5043
eat-4	0	3	3	3	61.9933	54.8855
eat-4	0	3	3	4	161.3215	74.1160
eat-4	0	3	4	5	114.2965	41.1998
eat-4	0	3	4	6	47.3446	69.4163
eat-4	0	3	4	7	30.5387	191.4371
eat-4	0	3	4	8	24.9351	47.4844
eat-4	0	3	4	9	133.0727	62.6290
eat-4	0	3	4	10	90.7200	95.0687
eat-4	0	3	4	11	85.0707	121.0911
eat-4	0	3	4	12	110.4020	88.3236
eat-4	0	3	4	13	135.9405	205.8365
eat-4	0	3	4	14	68.8568	108.4687
eat-4	0	3	4	15	134.2692	30.9639
eat-4	0	3	4	16	155.3156	204.5645
eat-4	0	3	4	17	139.0758	147.5484
eat-4	0	3	4	18	47.6257	51.5288
eat-4	0	3	4	19	17.6939	103.5704
eat-4	0	3	4	20	53.3934	49.2768
eat-4	0	3	4	21	125.3270	41.1290
eat-4	0	3	4	22	67.0966	120.8031
eat-4	0	3	4	23	136.2618	103.0992
eat-4	0	3	4	24	80.5845	63.5058
eat-4	0	3	4	25	101.7216	50.8809
eat-4	0	3	4	26	171.3300	99.5623
eat-4	0	3	4	27	103.2908	61.4339
eat-4	0	3	4	28	98.0151	96.3216
eat-4	0	3	4	29	25.6673	32.3370
eat-4	0	3	4	30	18.6705	72.6072
eat-4	0	3	4	31	22.7233	64.8308
eat-4	0	3	4	32	98.0190	13.5398
eat-4	0	3	4	33	80.0675	67.7534
eat-4	0	3	4	34	72.1230	65.5224
eat-4	0	3	4	35	62.8929	79.5324
eat-4	0	3	4	36	129.4498	61.5026
eat-4	0	3	4	37	12.8963	109.7711
eat-4	0	3	4	38	88.7421	45.4633
eat-4	0	3	4	39	14.5954	131.2080
eat-4	1.5	1	1	1	25.4563	158.8602
eat-4	1.5	1	1	2	46.8207	34.9695
eat-4	1.5	1	1	3	69.5421	43.8830
eat-4	1.5	1	1	4	74.6139	94.4474
eat-4	1.5	1	1	5	59.2337	124.6177
eat-4	1.5	1	1	6	13.0966	122.3801
eat-4	1.5	1	1	7	55.5343	34.1571
eat-4	1.5	1	2	1	11.2274	153.6914
eat-4	1.5	1	2	2	51.0232	69.1663
eat-4	1.5	1	2	3	108.8166	46.8222
eat-4	1.5	1	2	4	40.4545	99.5847
eat-4	1.5	1	2	5	85.4878	22.0889
eat-4	1.5	1	2	6	66.3128	15.3396
eat-4	1.5	1	2	7	71.0875	95.4042
eat-4	1.5	1	2	8	61.9568	48.0593
eat-4	1.5	1	2	9	76.2618	86.3226
eat-4	1.5	1	2	10	103.4718	40.0354
eat-4	1.5	1	2	11	94.0404	51.4158
eat-4	1.5	1	2	12	65.7266	168.0968
eat-4	1.5	1	2	13	31.9786	85.7025
eat-4	1.5	1	2	14	91.5787	48.9378

eat-4	1.5	1	2	15	12.4377	77.7370
eat-4	1.5	1	2	16	51.6331	125.2610
eat-4	1.5	1	3	1	28.2194	178.0948
eat-4	1.5	1	3	2	82.2117	114.3304
eat-4	1.5	1	3	3	89.2480	155.2266
eat-4	1.5	1	3	4	21.5217	67.6936
eat-4	1.5	1	3	5	49.2989	128.5034
eat-4	1.5	1	3	6	60.0455	98.3739
eat-4	1.5	1	3	7	42.1660	88.9972
eat-4	1.5	1	3	8	20.7533	159.4004
eat-4	1.5	1	3	9	32.0967	111.1688
eat-4	1.5	1	3	10	32.7605	107.8280
eat-4	1.5	1	3	11	14.6587	134.1307
eat-4	1.5	1	3	12	134.2062	139.5090
eat-4	1.5	1	3	13	31.0900	95.8125
eat-4	1.5	1	3	14	125.3606	24.6896
eat-4	1.5	1	3	15	10.9807	112.5320
eat-4	1.5	1	3	16	10.5191	149.7118
eat-4	1.5	1	3	17	86.6757	81.7034
eat-4	1.5	1	3	18	20.0723	123.7724
eat-4	1.5	1	3	19	66.0834	91.0976
eat-4	1.5	1	3	20	142.3826	57.3437
eat-4	1.5	1	3	21	96.2819	110.3569
eat-4	1.5	1	3	22	100.0451	153.8082
eat-4	1.5	1	4	1	53.2120	115.2773
eat-4	1.5	1	4	2	25.4240	145.2894
eat-4	1.5	1	4	3	13.6522	89.6371
eat-4	1.5	1	4	4	51.6493	105.7986
eat-4	1.5	1	4	5	55.8952	34.3047
eat-4	1.5	1	4	6	34.5514	140.0853
eat-4	1.5	1	4	7	65.4155	75.9582
eat-4	1.5	1	4	8	105.2598	78.1669
eat-4	1.5	1	4	9	17.8686	145.0672
eat-4	1.5	1	4	10	114.8788	24.9666
eat-4	1.5	1	4	11	58.5506	28.6854
eat-4	1.5	1	4	12	104.3715	128.2528
eat-4	1.5	1	4	13	110.6346	88.7112
eat-4	1.5	1	4	14	92.0922	18.6291
eat-4	1.5	1	4	15	87.1034	33.3114
eat-4	1.5	1	4	16	48.8895	106.6339
eat-4	1.5	1	4	17	80.4312	10.9074
eat-4	1.5	1	4	18	93.1860	25.9333
eat-4	1.5	1	4	19	38.5754	78.1256
eat-4	1.5	1	4	20	66.1215	138.7504
eat-4	1.5	1	4	21	149.2642	39.5074
eat-4	1.5	1	4	22	102.4203	13.7695
eat-4	1.5	1	4	23	21.4671	60.3930
eat-4	1.5	1	4	24	39.0095	145.6483
eat-4	1.5	1	4	25	102.1131	27.9393
eat-4	1.5	1	4	26	67.1358	102.4525
eat-4	1.5	1	4	27	10.7873	208.6874
eat-4	1.5	2	1	1	72.7845	114.5765
eat-4	1.5	2	1	2	6.3250	130.3186
eat-4	1.5	2	1	3	23.2294	62.9506
eat-4	1.5	2	1	4	27.2033	117.0563
eat-4	1.5	2	1	5	36.0731	67.7393
eat-4	1.5	2	1	6	104.8110	53.7540
eat-4	1.5	2	1	7	30.9993	33.5154
eat-4	1.5	2	1	8	132.6370	34.0491
eat-4	1.5	2	1	9	71.2780	56.3061
eat-4	1.5	2	1	10	127.0201	53.6040
eat-4	1.5	2	1	11	11.0984	191.6793
eat-4	1.5	2	1	12	42.2184	154.5725
eat-4	1.5	2	1	13	50.2445	128.7864
eat-4	1.5	2	1	14	80.5955	58.5450
eat-4	1.5	2	1	15	54.0447	43.5845
eat-4	1.5	2	1	16	50.2532	65.3330
eat-4	1.5	2	1	17	93.2017	79.8969

eat-4	1.5	2	1	18	47.7526	110.9903
eat-4	1.5	2	1	19	15.7322	130.6738
eat-4	1.5	2	1	20	130.6962	22.3213
eat-4	1.5	2	1	21	135.2076	49.5056
eat-4	1.5	2	1	22	116.3608	47.9467
eat-4	1.5	2	1	23	62.4857	48.7572
eat-4	1.5	2	1	24	140.8906	22.3092
eat-4	1.5	2	1	25	141.7439	48.4382
eat-4	1.5	2	1	26	30.6986	154.6071
eat-4	1.5	2	1	27	62.3271	31.0967
eat-4	1.5	2	1	28	21.6780	81.1543
eat-4	1.5	2	2	1	113.2153	107.3607
eat-4	1.5	2	2	2	19.4542	115.2258
eat-4	1.5	2	2	3	14.6311	79.4659
eat-4	1.5	2	2	4	7.9588	203.0891
eat-4	1.5	2	2	5	64.5500	35.2925
eat-4	1.5	2	2	6	108.0129	50.3346
eat-4	1.5	2	2	7	85.1603	43.2643
eat-4	1.5	2	2	8	124.1175	32.0898
eat-4	1.5	2	2	9	36.9783	75.6356
eat-4	1.5	2	2	10	33.0987	38.8055
eat-4	1.5	2	2	11	135.4501	92.3694
eat-4	1.5	2	2	12	134.2537	111.0679
eat-4	1.5	2	2	13	71.9672	105.7203
eat-4	1.5	2	2	14	20.4567	88.1960
eat-4	1.5	2	2	15	16.7780	120.0766
eat-4	1.5	2	2	16	99.7373	54.6879
eat-4	1.5	2	2	17	53.8723	67.4754
eat-4	1.5	2	2	18	86.1251	34.4883
eat-4	1.5	2	2	19	69.3629	56.4487
eat-4	1.5	2	2	20	13.4001	93.5937
eat-4	1.5	2	2	21	7.5625	136.4405
eat-4	1.5	2	2	22	94.7079	16.5812
eat-4	1.5	2	2	23	62.9195	11.1457
eat-4	1.5	2	2	24	58.1452	114.4829
eat-4	1.5	2	2	25	98.9097	49.2434
eat-4	1.5	2	2	26	32.4732	110.3606
eat-4	1.5	2	2	27	86.3760	111.5266
eat-4	1.5	2	3	1	28.1127	126.6514
eat-4	1.5	2	3	2	66.7137	53.9246
eat-4	1.5	2	3	3	114.3680	33.5630
eat-4	1.5	2	3	4	104.4711	45.1177
eat-4	1.5	2	3	5	24.2818	40.6158
eat-4	1.5	2	3	6	20.6324	28.9286
eat-4	1.5	2	3	7	13.6913	46.5550
eat-4	1.5	2	3	8	17.2976	113.6825
eat-4	1.5	2	3	9	30.9178	49.8156
eat-4	1.5	2	3	10	19.6196	71.2153
eat-4	1.5	2	3	11	48.5804	32.2769
eat-4	1.5	2	3	12	87.5336	49.7835
eat-4	1.5	2	3	13	23.9410	72.6431
eat-4	1.5	2	3	14	88.1127	29.0141
eat-4	1.5	2	3	15	25.1022	22.3787
eat-4	1.5	2	3	16	91.3525	16.5258
eat-4	1.5	2	3	17	85.9110	13.1369
eat-4	1.5	2	3	18	32.5461	56.2331
eat-4	1.5	2	3	19	99.0186	50.8828
eat-4	1.5	2	3	20	23.1686	98.3183
eat-4	1.5	2	3	21	27.6130	91.5278
eat-4	1.5	2	3	22	86.5306	60.6380
eat-4	1.5	2	3	23	69.0360	20.7884
eat-4	1.5	2	3	24	93.9577	42.2908
eat-4	1.5	2	3	25	81.0879	59.8132
eat-4	1.5	2	3	26	24.5011	132.3158
eat-4	1.5	2	3	27	17.7344	107.1329
eat-4	1.5	2	3	28	23.0255	63.7170
eat-4	1.5	2	3	29	79.1647	76.5978
eat-4	1.5	2	3	30	99.1434	19.9769

eat-4	1.5	2	3	31	71.0873	71.0579
eat-4	1.5	2	3	32	60.7884	114.4656
eat-4	1.5	2	3	33	45.9925	42.2577
eat-4	1.5	2	3	34	48.0381	31.9507
eat-4	1.5	2	3	35	81.0511	3.3593
eat-4	1.5	2	3	36	86.2959	24.8284
eat-4	1.5	2	3	37	30.6281	37.1679
eat-4	1.5	2	3	38	42.4756	88.2060
eat-4	1.5	2	3	39	90.4577	27.1386
eat-4	1.5	2	4	1	123.3056	51.2298
eat-4	1.5	2	4	2	76.0971	54.2390
eat-4	1.5	2	4	3	92.7817	29.1511
eat-4	1.5	2	4	4	11.0730	91.0259
eat-4	1.5	2	4	5	14.7018	59.8921
eat-4	1.5	2	4	6	67.1642	51.1823
eat-4	1.5	2	4	7	85.8390	45.9350
eat-4	1.5	2	4	8	126.7756	49.1962
eat-4	1.5	2	4	9	26.3521	85.4482
eat-4	1.5	2	4	10	17.1430	86.5269
eat-4	1.5	2	4	11	60.6236	66.4485
eat-4	1.5	2	4	12	76.6884	64.3331
eat-4	1.5	2	4	13	71.8689	85.7320
eat-4	1.5	2	4	14	88.7961	47.6499
eat-4	1.5	2	4	15	23.1609	122.6172
eat-4	1.5	2	4	16	12.0906	75.7173
eat-4	1.5	2	4	17	41.0153	81.7434
eat-4	1.5	2	4	18	90.9652	39.9459
eat-4	1.5	2	4	19	22.6734	107.9769
eat-4	1.5	2	4	20	12.7328	200.4683
eat-4	1.5	2	4	21	76.6508	81.0916
eat-4	1.5	2	4	22	6.2994	117.7303
eat-4	1.5	3	1	1	17.1746	61.4434
eat-4	1.5	3	1	2	76.5831	23.2827
eat-4	1.5	3	1	3	107.1613	17.0384
eat-4	1.5	3	1	4	74.8071	61.7550
eat-4	1.5	3	1	5	59.6933	62.3751
eat-4	1.5	3	1	6	62.6679	75.0624
eat-4	1.5	3	1	7	98.4036	49.1656
eat-4	1.5	3	1	8	88.3246	53.6705
eat-4	1.5	3	1	9	96.9933	115.8892
eat-4	1.5	3	1	10	41.5166	88.8992
eat-4	1.5	3	1	11	43.7714	135.3601
eat-4	1.5	3	1	12	60.8694	106.2231
eat-4	1.5	3	1	13	38.0779	84.4676
eat-4	1.5	3	1	14	66.0063	76.1481
eat-4	1.5	3	1	15	31.8090	109.1464
eat-4	1.5	3	1	16	103.8249	34.6438
eat-4	1.5	3	1	17	89.0799	63.6536
eat-4	1.5	3	1	18	13.9898	83.1488
eat-4	1.5	3	1	19	125.4206	22.9018
eat-4	1.5	3	1	20	100.4728	53.2640
eat-4	1.5	3	1	21	10.4498	63.0423
eat-4	1.5	3	1	22	111.6771	71.9409
eat-4	1.5	3	1	23	50.9527	54.9406
eat-4	1.5	3	2	1	86.0543	47.3917
eat-4	1.5	3	2	2	13.4360	172.6297
eat-4	1.5	3	2	3	124.7294	45.8211
eat-4	1.5	3	2	4	54.8906	117.8464
eat-4	1.5	3	2	5	81.6726	96.0442
eat-4	1.5	3	2	6	157.2917	47.3490
eat-4	1.5	3	2	7	127.0851	86.1952
eat-4	1.5	3	2	8	148.2700	43.0578
eat-4	1.5	3	2	9	56.0402	72.1260
eat-4	1.5	3	2	10	17.5947	69.5971
eat-4	1.5	3	2	11	49.9540	152.3860
eat-4	1.5	3	2	12	117.2890	76.7839
eat-4	1.5	3	2	13	79.0560	41.8196
eat-4	1.5	3	2	14	35.4771	96.2040

eat-4	1.5	3	2	15	15.8510	61.3238
eat-4	1.5	3	2	16	79.4175	136.8312
eat-4	1.5	3	2	17	21.2125	128.3044
eat-4	1.5	3	2	18	63.6439	60.8525
eat-4	1.5	3	2	19	143.5491	135.4215
eat-4	1.5	3	2	20	49.6832	51.3082
eat-4	1.5	3	2	21	9.6867	79.1681
eat-4	1.5	3	2	22	71.7754	12.5571
eat-4	1.5	3	2	23	89.0822	70.6741
eat-4	1.5	3	2	24	27.3188	155.3972
eat-4	1.5	3	3	1	96.4197	44.2351
eat-4	1.5	3	3	2	45.5590	97.5750
eat-4	1.5	3	3	3	17.3857	182.5717
eat-4	1.5	3	3	4	39.2132	153.1844
eat-4	1.5	3	3	5	61.0098	70.4318
eat-4	1.5	3	3	6	66.3818	170.3426
eat-4	1.5	3	3	7	116.0225	51.1461
eat-4	1.5	3	3	8	28.9688	61.0649
eat-4	1.5	3	3	9	20.3219	150.2415
eat-4	1.5	3	3	10	89.4748	54.0550
eat-4	1.5	3	3	11	34.7696	63.6868
eat-4	1.5	3	3	12	55.4619	146.8009
eat-4	1.5	3	3	13	129.0097	51.5480
eat-4	1.5	3	3	14	70.2537	100.0230
eat-4	1.5	3	3	15	23.4246	214.0799
eat-4	1.5	3	3	16	14.9474	146.7555
eat-4	1.5	3	3	17	70.1470	57.9829
eat-4	1.5	3	3	18	55.7847	43.4104
eat-4	1.5	3	3	19	11.4422	117.3414
eat-4	1.5	3	3	20	39.0451	58.1970
eat-4	1.5	3	3	21	79.1668	71.8299
eat-4	1.5	3	3	22	54.0157	38.6186
eat-4	1.5	3	4	1	47.1097	70.7221
eat-4	1.5	3	4	2	46.3090	81.5439
eat-4	1.5	3	4	3	139.8484	16.5750
eat-4	1.5	3	4	4	23.1063	147.5463
eat-4	1.5	3	4	5	67.4460	104.0750
eat-4	1.5	3	4	6	70.9169	57.6035
eat-4	1.5	3	4	7	87.2679	35.4803
eat-4	1.5	3	4	8	33.2144	146.2661
eat-4	1.5	3	4	9	15.0556	115.9294
eat-4	1.5	3	4	10	18.7232	105.6500
eat-4	1.5	3	4	11	72.6263	46.2447
eat-4	1.5	3	4	12	85.8939	45.9372
eat-4	1.5	3	4	13	16.8567	144.0142
eat-4	1.5	3	4	14	31.4843	93.3786
eat-4	1.5	3	4	15	29.0682	106.6708
eat-4	1.5	3	4	16	91.6383	60.5160
eat-4	1.5	3	4	17	86.8346	51.6155
eat-4	1.5	3	4	18	155.5033	50.4664
eat-4	1.5	3	4	19	95.5627	51.5302
eat-4	1.5	3	4	20	124.7977	48.2940
eat-4	1.5	3	4	21	126.8603	76.9374
eat-4	1.5	3	4	22	91.1312	64.3387
eat-4	1.5	3	4	23	35.1603	114.6980
eat-4	1.5	3	4	24	53.8214	166.1523
eat-4	1.5	3	4	25	96.8029	38.6917
eat-4	1.5	3	4	26	13.8414	193.4969
eat-4	1.5	3	4	27	17.0358	69.0368
eat-4	1.5	3	4	28	35.0379	126.1168
eat-4	1.5	3	4	29	82.8725	81.8545
eat-4	1.5	3	4	30	89.1180	138.9686
eat-4	1.5	3	4	31	124.0790	79.1864
eat-4	1.5	3	4	32	92.3897	80.5975
eat-4	1.5	3	4	33	108.7618	58.3732
eat-4	1.5	3	4	34	93.2234	67.7286
eat-4	1.5	3	4	35	109.8962	59.4972
eat-4	1.5	3	4	36	29.8671	118.2820

eat-4	1.5	3	4	37	68.8108	53.9128
eat-4	1.5	3	4	38	20.5862	63.8860
eat-4	1.5	3	4	39	31.1547	90.3933
eat-4	1.5	3	4	40	83.6110	20.0277
eat-4	3	1	1	1	16.5929	124.1662
eat-4	3	1	1	2	87.0884	39.4008
eat-4	3	1	1	3	52.0711	96.4553
eat-4	3	1	1	4	72.9499	68.0969
eat-4	3	1	1	5	83.0601	20.6687
eat-4	3	1	1	6	88.9922	27.9860
eat-4	3	1	1	7	99.1955	44.6783
eat-4	3	1	1	8	55.4809	16.8107
eat-4	3	1	1	9	85.4882	19.4289
eat-4	3	1	1	10	68.4589	24.0358
eat-4	3	1	1	11	92.4163	55.0685
eat-4	3	1	1	12	135.5976	18.7758
eat-4	3	1	1	13	55.8087	199.6234
eat-4	3	1	1	14	51.4182	95.2455
eat-4	3	1	1	15	65.2652	79.5169
eat-4	3	1	2	1	30.9360	84.9045
eat-4	3	1	2	2	22.0762	138.2766
eat-4	3	1	2	3	107.4816	45.3400
eat-4	3	1	2	4	114.8948	65.1109
eat-4	3	1	2	5	19.0706	93.2315
eat-4	3	1	2	6	135.1098	39.4564
eat-4	3	1	2	7	104.3021	8.4895
eat-4	3	1	2	8	69.5981	96.6602
eat-4	3	1	2	9	88.6711	39.1572
eat-4	3	1	2	10	83.4814	46.9801
eat-4	3	1	2	11	121.0666	16.2755
eat-4	3	1	2	12	103.4644	16.3494
eat-4	3	1	2	13	108.8582	97.0587
eat-4	3	1	2	14	123.9943	38.7175
eat-4	3	1	2	15	89.0381	7.1989
eat-4	3	1	2	16	100.0162	42.2955
eat-4	3	1	2	17	148.4384	32.3698
eat-4	3	1	2	18	108.3634	32.9291
eat-4	3	1	2	19	107.0495	20.4310
eat-4	3	1	2	20	97.0380	57.9928
eat-4	3	1	2	21	98.0753	24.1836
eat-4	3	1	2	22	48.5503	194.4705
eat-4	3	1	2	23	62.4823	56.2214
eat-4	3	1	2	24	157.0003	37.2553
eat-4	3	1	2	25	114.0265	58.7470
eat-4	3	1	2	26	74.7673	104.5151
eat-4	3	1	3	1	52.1541	49.8512
eat-4	3	1	3	2	52.0011	60.1005
eat-4	3	1	3	3	30.1228	33.0516
eat-4	3	1	3	4	134.8780	46.1359
eat-4	3	1	3	5	36.9487	61.3722
eat-4	3	1	3	6	141.1386	19.5771
eat-4	3	1	3	7	36.2574	109.6676
eat-4	3	1	3	8	42.3999	161.8181
eat-4	3	1	3	9	127.8604	62.1723
eat-4	3	1	3	10	99.6211	15.0996
eat-4	3	1	3	11	64.1326	22.8834
eat-4	3	1	3	12	101.2439	51.6773
eat-4	3	1	3	13	133.2361	55.5616
eat-4	3	1	3	14	116.1296	36.8628
eat-4	3	1	3	15	40.8063	207.0801
eat-4	3	1	3	16	122.5322	11.5759
eat-4	3	1	3	17	143.7454	51.0902
eat-4	3	1	3	18	88.9594	102.4266
eat-4	3	1	3	19	114.7129	19.2039
eat-4	3	1	3	20	114.7957	61.5362
eat-4	3	1	3	21	103.5452	33.7717
eat-4	3	1	3	22	37.6918	24.4738
eat-4	3	1	3	23	8.8360	294.4807

eat-4	3	1	4	1	15.4286	183.6781
eat-4	3	1	4	2	30.8078	44.9117
eat-4	3	1	4	3	73.9118	23.9617
eat-4	3	1	4	4	10.9362	168.6701
eat-4	3	1	4	5	40.9559	59.4674
eat-4	3	1	4	6	89.4635	26.1346
eat-4	3	1	4	7	138.0331	22.3675
eat-4	3	1	4	8	80.1396	60.3884
eat-4	3	1	4	9	161.5864	33.7303
eat-4	3	1	4	10	96.3442	20.2736
eat-4	3	1	4	11	107.4562	61.4309
eat-4	3	1	4	12	130.6205	110.3316
eat-4	3	1	4	13	68.7474	16.4111
eat-4	3	1	4	14	89.6063	12.1079
eat-4	3	1	4	15	107.1672	45.0723
eat-4	3	1	4	16	114.3371	65.8499
eat-4	3	1	4	17	90.3735	63.9979
eat-4	3	1	4	18	28.5167	142.1726
eat-4	3	1	4	19	88.7354	28.5546
eat-4	3	1	4	20	123.3078	70.5656
eat-4	3	1	4	21	140.3266	16.2040
eat-4	3	1	4	22	46.3980	52.4233
eat-4	3	1	4	23	131.2833	19.5821
eat-4	3	1	4	24	68.2980	37.6144
eat-4	3	1	4	25	73.5134	11.7215
eat-4	3	2	1	1	14.6219	144.9690
eat-4	3	2	1	2	22.8087	182.2940
eat-4	3	2	1	3	94.6598	64.8682
eat-4	3	2	1	4	26.6492	161.6400
eat-4	3	2	1	5	16.7989	241.7127
eat-4	3	2	1	6	12.6955	101.3563
eat-4	3	2	1	7	85.0031	72.8945
eat-4	3	2	1	8	114.2902	26.4770
eat-4	3	2	1	9	85.5559	107.2073
eat-4	3	2	1	10	50.0196	75.2526
eat-4	3	2	1	11	28.3056	129.1885
eat-4	3	2	1	12	73.9642	60.8446
eat-4	3	2	1	13	52.7786	94.0980
eat-4	3	2	1	14	16.2122	134.9683
eat-4	3	2	1	15	85.1378	46.6255
eat-4	3	2	1	16	30.3656	104.5731
eat-4	3	2	1	17	75.4828	41.6498
eat-4	3	2	1	18	92.6066	81.1446
eat-4	3	2	1	19	79.8824	75.8417
eat-4	3	2	1	20	20.8692	135.2932
eat-4	3	2	1	21	69.0755	23.3433
eat-4	3	2	1	22	15.2213	262.9687
eat-4	3	2	1	23	23.6339	234.6982
eat-4	3	2	1	24	41.2575	219.6634
eat-4	3	2	1	25	74.4365	147.2073
eat-4	3	2	1	26	36.7860	73.1228
eat-4	3	2	1	27	45.5188	72.8063
eat-4	3	2	1	28	57.0758	60.9127
eat-4	3	2	1	29	74.2344	175.0544
eat-4	3	2	1	30	23.9526	107.5355
eat-4	3	2	1	31	30.6696	93.3833
eat-4	3	2	1	32	86.2937	52.2105
eat-4	3	2	2	1	53.3898	63.4553
eat-4	3	2	2	2	29.7641	68.0406
eat-4	3	2	2	3	57.2099	92.3916
eat-4	3	2	2	4	131.9186	56.8526
eat-4	3	2	2	5	95.2982	54.4080
eat-4	3	2	2	6	48.7891	52.1602
eat-4	3	2	2	7	20.5177	151.5372
eat-4	3	2	2	8	8.4346	172.3734
eat-4	3	2	2	9	20.8388	85.6837
eat-4	3	2	2	10	64.7050	57.8521
eat-4	3	2	2	11	94.8273	49.5769

eat-4	3	2	2	12	54.5475	125.4160
eat-4	3	2	2	13	21.6174	173.1166
eat-4	3	2	2	14	16.0903	157.0018
eat-4	3	2	2	15	40.7205	77.2690
eat-4	3	2	2	16	43.2870	48.5833
eat-4	3	2	2	17	86.4878	71.3256
eat-4	3	2	2	18	28.2280	85.9592
eat-4	3	2	2	19	26.1585	127.4378
eat-4	3	2	2	20	123.3868	4.9254
eat-4	3	2	2	21	96.2111	54.5869
eat-4	3	2	2	22	75.9175	125.5460
eat-4	3	2	2	23	18.1208	144.8085
eat-4	3	2	2	24	66.3982	43.5352
eat-4	3	2	2	25	66.2146	19.8956
eat-4	3	2	2	26	80.9150	10.5218
eat-4	3	2	2	27	58.5960	89.0129
eat-4	3	2	2	28	88.2447	115.2089
eat-4	3	2	3	1	12.9949	89.0570
eat-4	3	2	3	2	53.0725	39.6538
eat-4	3	2	3	3	28.2736	127.2996
eat-4	3	2	3	4	82.9419	33.6599
eat-4	3	2	3	5	18.8000	118.8549
eat-4	3	2	3	6	77.9190	28.2478
eat-4	3	2	3	7	51.5085	165.2259
eat-4	3	2	3	8	90.4657	61.6931
eat-4	3	2	3	9	12.9875	138.0185
eat-4	3	2	3	10	97.0725	57.8871
eat-4	3	2	3	11	76.7392	86.5452
eat-4	3	2	3	12	40.8757	145.3622
eat-4	3	2	3	13	80.9977	55.9262
eat-4	3	2	3	14	133.0490	119.4232
eat-4	3	2	3	15	34.2180	39.9139
eat-4	3	2	3	16	28.6429	45.9831
eat-4	3	2	3	17	109.3871	96.3674
eat-4	3	2	3	18	116.9584	79.9195
eat-4	3	2	3	19	86.2936	51.4295
eat-4	3	2	3	20	24.9924	52.3089
eat-4	3	2	3	21	26.4097	186.9293
eat-4	3	2	3	22	86.1954	1.7971
eat-4	3	2	3	23	92.8512	125.7059
eat-4	3	2	3	24	55.3695	51.1430
eat-4	3	2	3	25	40.5395	197.4342
eat-4	3	2	3	26	16.0667	154.2641
eat-4	3	2	3	27	52.5529	175.8355
eat-4	3	2	3	28	65.4441	8.3813
eat-4	3	2	3	29	96.2463	118.4376
eat-4	3	2	3	30	103.7254	219.6406
eat-4	3	2	4	1	50.0337	41.7997
eat-4	3	2	4	2	84.7357	48.8347
eat-4	3	2	4	3	36.6904	68.1059
eat-4	3	2	4	4	11.1132	76.7018
eat-4	3	2	4	5	43.9404	118.7505
eat-4	3	2	4	6	15.7194	159.0672
eat-4	3	2	4	7	98.4431	65.0968
eat-4	3	2	4	8	62.4805	41.1302
eat-4	3	2	4	9	71.3324	56.7879
eat-4	3	2	4	10	77.1283	61.7542
eat-4	3	2	4	11	101.1356	111.3914
eat-4	3	2	4	12	21.3975	121.8521
eat-4	3	2	4	13	114.3469	100.8228
eat-4	3	2	4	14	54.9752	161.1789
eat-4	3	2	4	15	143.1690	101.1498
eat-4	3	2	4	16	110.6260	33.9336
eat-4	3	2	4	17	77.6111	160.0442
eat-4	3	2	4	18	103.7098	18.1408
eat-4	3	2	4	19	35.9437	227.4712
eat-4	3	2	4	20	36.8582	124.1850
eat-4	3	2	4	21	21.0889	158.2353

eat-4	3	2	4	22	50.3569	69.6414
eat-4	3	2	4	23	115.9286	45.4347
eat-4	3	2	4	24	89.9395	94.2323
eat-4	3	2	4	25	97.4483	8.7939
eat-4	3	2	4	26	7.8590	132.4573
eat-4	3	2	4	27	23.6907	225.6054
eat-4	3	2	4	28	32.7768	243.5649
eat-4	3	2	4	29	32.8300	98.3965
eat-4	3	3	1	1	33.3355	131.1844
eat-4	3	3	1	2	26.5226	249.4843
eat-4	3	3	1	3	31.9432	156.2462
eat-4	3	3	1	4	39.5961	78.4462
eat-4	3	3	1	5	25.8868	172.0787
eat-4	3	3	1	6	12.0084	194.5359
eat-4	3	3	1	7	25.3429	122.4948
eat-4	3	3	1	8	56.7996	95.1026
eat-4	3	3	1	9	128.0162	44.3102
eat-4	3	3	1	10	102.6742	22.3957
eat-4	3	3	1	11	39.4392	108.9929
eat-4	3	3	1	12	37.4889	162.2951
eat-4	3	3	1	13	120.9118	16.8306
eat-4	3	3	1	14	23.5068	76.6924
eat-4	3	3	1	15	14.6147	194.4398
eat-4	3	3	1	16	16.5860	240.7330
eat-4	3	3	1	17	19.3605	118.8041
eat-4	3	3	1	18	62.4062	44.6063
eat-4	3	3	1	19	147.0800	22.0506
eat-4	3	3	1	20	47.1169	190.9305
eat-4	3	3	1	21	39.4616	149.4544
eat-4	3	3	2	1	60.5237	119.8844
eat-4	3	3	2	2	14.7953	110.4440
eat-4	3	3	2	3	43.2346	202.1521
eat-4	3	3	2	4	44.4383	170.0895
eat-4	3	3	2	5	35.2521	205.1804
eat-4	3	3	2	6	87.5475	38.9722
eat-4	3	3	2	7	39.6697	48.8630
eat-4	3	3	2	8	20.8380	115.0248
eat-4	3	3	2	9	50.8090	122.2720
eat-4	3	3	2	10	83.5780	81.3785
eat-4	3	3	2	11	61.5323	106.0433
eat-4	3	3	2	12	40.4948	144.4331
eat-4	3	3	2	13	53.9363	146.6883
eat-4	3	3	2	14	15.4196	193.9478
eat-4	3	3	2	15	101.0962	57.7476
eat-4	3	3	2	16	45.7659	121.8701
eat-4	3	3	2	17	43.4397	107.3654
eat-4	3	3	2	18	18.6781	121.3835
eat-4	3	3	2	19	37.7172	166.9184
eat-4	3	3	2	20	34.6723	171.7128
eat-4	3	3	3	1	45.6303	137.0081
eat-4	3	3	3	2	14.2593	103.0954
eat-4	3	3	3	3	131.1069	44.4684
eat-4	3	3	3	4	22.6764	176.1004
eat-4	3	3	3	5	78.8688	66.2511
eat-4	3	3	3	6	95.3456	15.3519
eat-4	3	3	3	7	103.6988	17.8978
eat-4	3	3	3	8	81.3969	38.7128
eat-4	3	3	3	9	86.0972	57.3390
eat-4	3	3	3	10	28.8883	72.6297
eat-4	3	3	3	11	99.1872	66.0520
eat-4	3	3	3	12	67.7757	49.5012
eat-4	3	3	3	13	41.2697	62.9537
eat-4	3	3	3	14	37.7573	93.4355
eat-4	3	3	3	15	143.0967	38.7327
eat-4	3	3	3	16	70.0415	34.6272
eat-4	3	3	3	17	36.3342	145.9719
eat-4	3	3	3	18	30.1080	90.5776
eat-4	3	3	3	19	89.6871	32.0322

eat-4	3	3	3	20	40.5352	98.5880
eat-4	3	3	3	21	29.6741	203.1219
eat-4	3	3	3	22	25.3475	218.7739
eat-4	3	3	3	23	22.9741	50.7365
eat-4	3	3	3	24	42.3189	63.8772
eat-4	3	3	3	25	87.9205	29.8876
eat-4	3	3	3	26	18.2239	135.5256
eat-4	3	3	3	27	91.9389	49.0867
eat-4	3	3	3	28	41.8123	46.5102
eat-4	3	3	3	29	65.8528	26.2934
eat-4	3	3	4	1	63.0013	134.4400
eat-4	3	3	4	2	16.9923	159.9328
eat-4	3	3	4	3	31.0369	179.9540
eat-4	3	3	4	4	98.5202	47.6282
eat-4	3	3	4	5	14.8785	103.2881
eat-4	3	3	4	6	9.3730	249.4419
eat-4	3	3	4	7	46.2876	110.0428
eat-4	3	3	4	8	62.6770	48.7747
eat-4	3	3	4	9	67.5113	50.9257
eat-4	3	3	4	10	48.9711	99.9821
eat-4	3	3	4	11	52.8960	110.5613
eat-4	3	3	4	12	64.9902	78.8444
eat-4	3	3	4	13	45.6780	83.6880
eat-4	3	3	4	14	47.7227	70.7923
eat-4	3	3	4	15	87.8600	100.4802
eat-4	3	3	4	16	74.0736	211.2129
eat-4	3	3	4	17	50.7431	58.2256
eat-4	3	3	4	18	104.5226	17.0697
eat-4	3	3	4	19	70.7587	60.3680
eat-4	3	3	4	20	81.1179	144.5627
eat-4	3	3	4	21	80.8007	24.9080
eat-4	3	3	4	22	98.6345	33.0830
eat-4	3	3	4	23	77.3474	107.2366
eat-4	3	3	4	24	59.9674	58.4860
eat-4	3	3	4	25	26.6032	92.7644
eat-4	3	3	4	26	89.5365	46.1938
eat-4	3	3	4	27	36.3094	185.8401
eat-4	3	3	4	28	37.7862	33.3438
eat-4	3	3	4	29	10.1239	186.2337
eat-4	3	3	4	30	86.2426	46.9222
eat-4	3	3	4	31	56.7181	79.9165
eat-4	3	3	4	32	139.2923	33.7622
eat-4	3	3	4	33	124.1216	23.3610
eat-4	3	3	4	34	97.5396	1.8557
eat-4	3	3	4	35	153.4046	138.2025
eat-4	3	3	4	36	30.3111	161.1662
eat-4	3	3	4	37	13.4971	67.3330
eat-4	3	3	4	38	100.0654	69.1162
eat-4	3	3	4	39	38.2353	120.8176
eat-4	6	1	1	1	29.3038	173.0640
eat-4	6	1	1	2	44.4861	102.5613
eat-4	6	1	1	3	94.2071	92.6622
eat-4	6	1	1	4	49.4634	170.5081
eat-4	6	1	1	5	92.3493	13.3961
eat-4	6	1	1	6	88.0174	109.9104
eat-4	6	1	1	7	84.4481	92.6761
eat-4	6	1	1	8	87.2215	60.9236
eat-4	6	1	1	9	93.3123	112.5445
eat-4	6	1	1	10	52.4569	200.6221
eat-4	6	1	1	11	86.4913	31.6608
eat-4	6	1	1	12	11.6156	210.5428
eat-4	6	1	1	13	62.1123	136.6580
eat-4	6	1	1	14	108.6854	43.8434
eat-4	6	1	1	15	70.3181	49.9092
eat-4	6	1	1	16	32.0869	250.2434
eat-4	6	1	1	17	24.5712	284.5949
eat-4	6	1	1	18	97.2393	23.4652
eat-4	6	1	2	1	12.9686	153.8740

eat-4	6	1	2	2	26.6901	161.6641
eat-4	6	1	2	3	80.6158	51.5623
eat-4	6	1	2	4	15.2787	107.8041
eat-4	6	1	2	5	67.2526	10.4834
eat-4	6	1	2	6	118.8579	107.9422
eat-4	6	1	2	7	81.9641	60.2892
eat-4	6	1	2	8	140.7078	134.8055
eat-4	6	1	2	9	68.5165	136.4160
eat-4	6	1	2	10	76.0061	88.3109
eat-4	6	1	2	11	83.0616	11.7061
eat-4	6	1	2	12	97.0538	152.2929
eat-4	6	1	2	13	51.9910	32.1459
eat-4	6	1	2	14	43.3265	105.0083
eat-4	6	1	2	15	51.5930	152.8888
eat-4	6	1	2	16	99.8604	83.2752
eat-4	6	1	2	17	102.7911	198.8551
eat-4	6	1	2	18	118.0626	58.2661
eat-4	6	1	2	19	58.8767	98.7479
eat-4	6	1	2	20	44.1616	152.4480
eat-4	6	1	2	21	14.6221	152.7852
eat-4	6	1	3	1	22.3149	151.4107
eat-4	6	1	3	2	44.5908	64.6532
eat-4	6	1	3	3	60.0141	114.7319
eat-4	6	1	3	4	43.2059	173.3687
eat-4	6	1	3	5	118.4497	97.7992
eat-4	6	1	3	6	88.2033	249.4667
eat-4	6	1	3	7	51.8696	187.7337
eat-4	6	1	3	8	57.3180	243.0424
eat-4	6	1	3	9	90.6635	197.3264
eat-4	6	1	3	10	81.9542	38.1679
eat-4	6	1	3	11	46.2249	213.2915
eat-4	6	1	3	12	97.8427	37.2066
eat-4	6	1	3	13	75.9431	103.2954
eat-4	6	1	3	14	9.3595	171.7926
eat-4	6	1	3	15	70.5587	46.1853
eat-4	6	1	3	16	90.9836	10.5130
eat-4	6	1	3	17	49.8847	89.4866
eat-4	6	1	3	18	76.7234	341.3312
eat-4	6	1	3	19	73.0941	63.6556
eat-4	6	1	3	20	19.9390	257.9200
eat-4	6	1	3	21	84.0756	79.9057
eat-4	6	1	3	22	69.6150	142.7079
eat-4	6	1	3	23	41.1971	135.2611
eat-4	6	1	3	24	86.4352	41.6455
eat-4	6	1	4	1	26.5851	101.2293
eat-4	6	1	4	2	41.7434	239.1177
eat-4	6	1	4	3	64.2902	80.7418
eat-4	6	1	4	4	51.6327	141.0809
eat-4	6	1	4	5	65.3301	75.0593
eat-4	6	1	4	6	79.6538	16.4421
eat-4	6	1	4	7	84.6145	82.4349
eat-4	6	1	4	8	96.8228	62.2456
eat-4	6	1	4	9	60.8847	73.4140
eat-4	6	1	4	10	83.8301	86.0197
eat-4	6	1	4	11	73.5114	98.5393
eat-4	6	1	4	12	94.5301	181.4558
eat-4	6	1	4	13	56.5092	137.4629
eat-4	6	1	4	14	67.7318	76.7047
eat-4	6	1	4	15	105.1689	47.4518
eat-4	6	1	4	16	136.2340	174.5139
eat-4	6	1	4	17	61.8421	234.6231
eat-4	6	1	4	18	73.7096	22.1429
eat-4	6	1	4	19	95.0001	114.4620
eat-4	6	1	4	20	59.1023	151.4799
eat-4	6	1	4	21	10.1595	269.4042
eat-4	6	1	4	22	158.2233	65.3607
eat-4	6	1	4	23	96.4370	7.9829
eat-4	6	1	4	24	101.8612	29.7057

eat-4	6	1	4	25	107.6831	21.2387
eat-4	6	1	4	26	21.0816	116.4009
eat-4	6	1	4	27	77.5371	43.1597
eat-4	6	2	1	1	11.5920	227.0107
eat-4	6	2	1	2	59.1368	137.8847
eat-4	6	2	1	3	68.1586	125.2298
eat-4	6	2	1	4	17.3195	233.2616
eat-4	6	2	1	5	54.7771	152.9545
eat-4	6	2	1	6	9.7378	210.6420
eat-4	6	2	1	7	49.5789	55.6761
eat-4	6	2	1	8	78.8061	142.3585
eat-4	6	2	1	9	60.7460	181.4747
eat-4	6	2	1	10	41.5993	213.1100
eat-4	6	2	1	11	26.1579	232.2903
eat-4	6	2	2	1	22.3751	160.1155
eat-4	6	2	2	2	31.3902	160.7756
eat-4	6	2	2	3	33.8389	204.9583
eat-4	6	2	2	4	19.9430	194.3618
eat-4	6	2	2	5	29.4837	195.2002
eat-4	6	2	2	6	64.7593	211.8710
eat-4	6	2	2	7	36.5923	219.3387
eat-4	6	2	2	8	16.2570	215.3879
eat-4	6	2	2	9	26.1419	208.3746
eat-4	6	2	2	10	30.0120	256.2611
eat-4	6	2	2	11	90.3772	40.3306
eat-4	6	2	2	12	103.2348	2.2637
eat-4	6	2	2	13	26.0147	208.6572
eat-4	6	2	2	14	30.1606	229.7264
eat-4	6	2	2	15	89.8144	47.2558
eat-4	6	2	2	16	37.3676	233.9270
eat-4	6	2	2	17	54.4548	248.5387
eat-4	6	2	2	18	36.6375	162.5547
eat-4	6	2	2	19	75.9936	192.8784
eat-4	6	2	2	20	9.7226	232.4245
eat-4	6	2	2	21	99.5463	102.1456
eat-4	6	2	2	22	76.5053	158.8819
eat-4	6	2	2	23	11.6303	145.1593
eat-4	6	2	2	24	34.3838	212.1685
eat-4	6	2	2	25	101.4919	1.8558
eat-4	6	2	2	26	79.3934	36.8417
eat-4	6	2	3	1	35.3420	143.8437
eat-4	6	2	3	2	26.1550	184.7006
eat-4	6	2	3	3	19.7675	214.8662
eat-4	6	2	3	4	88.5816	53.0839
eat-4	6	2	3	5	19.7471	205.8288
eat-4	6	2	3	6	9.7965	234.6533
eat-4	6	2	3	7	25.6055	255.2025
eat-4	6	2	3	8	13.1318	205.7929
eat-4	6	2	3	9	26.6251	281.2442
eat-4	6	2	3	10	80.2194	28.3680
eat-4	6	2	3	11	110.6768	180.9411
eat-4	6	2	3	12	59.2357	128.2977
eat-4	6	2	3	13	72.4614	103.0831
eat-4	6	2	3	14	49.3708	261.5920
eat-4	6	2	3	15	94.8521	8.8939
eat-4	6	2	3	16	54.9141	64.7313
eat-4	6	2	3	17	82.8758	1.7735
eat-4	6	2	3	18	31.7022	229.6422
eat-4	6	2	3	19	62.7952	184.5959
eat-4	6	2	3	20	22.5595	208.4112
eat-4	6	2	3	21	40.6693	270.0174
eat-4	6	2	3	22	55.2036	108.8857
eat-4	6	2	3	23	40.1671	217.5849
eat-4	6	2	3	24	109.9695	140.3698
eat-4	6	2	3	25	23.0523	154.6924
eat-4	6	2	3	26	46.8258	213.6074
eat-4	6	2	3	27	45.1958	223.6490
eat-4	6	2	3	28	88.3740	64.7391

eat-4	6	2	3	29	34.8416	214.8561
eat-4	6	2	3	30	53.5481	88.1343
eat-4	6	2	3	31	64.3935	266.9802
eat-4	6	2	3	32	90.3282	126.0007
eat-4	6	2	3	33	35.9729	241.8068
eat-4	6	2	3	34	22.2536	159.5389
eat-4	6	2	3	35	47.1103	205.5556
eat-4	6	2	3	36	32.6446	182.0286
eat-4	6	2	3	37	14.5504	149.1445
eat-4	6	2	3	38	20.6505	123.7153
eat-4	6	2	3	39	67.7860	199.5921
eat-4	6	2	3	40	95.6157	177.3415
eat-4	6	2	3	41	45.8043	48.4036
eat-4	6	2	3	42	42.7292	143.3399
eat-4	6	2	3	43	56.7606	76.5108
eat-4	6	2	3	44	41.7657	126.7090
eat-4	6	2	3	45	16.5446	85.8908
eat-4	6	2	4	1	65.6164	90.6465
eat-4	6	2	4	2	52.5189	132.0327
eat-4	6	2	4	3	76.3582	122.4900
eat-4	6	2	4	4	25.4729	174.1529
eat-4	6	2	4	5	38.0621	186.6420
eat-4	6	2	4	6	72.6229	49.4981
eat-4	6	2	4	7	38.4269	221.9564
eat-4	6	2	4	8	29.8400	214.1631
eat-4	6	2	4	9	68.0056	93.0485
eat-4	6	2	4	10	33.2286	212.6883
eat-4	6	2	4	11	57.2335	124.6315
eat-4	6	2	4	12	102.3692	44.4236
eat-4	6	2	4	13	79.5009	193.4337
eat-4	6	2	4	14	105.3306	141.2094
eat-4	6	2	4	15	8.9087	239.6937
eat-4	6	3	4	16	105.0878	141.9595
eat-4	6	2	4	17	113.8886	244.2388
eat-4	6	2	4	18	63.6963	155.1785
eat-4	6	2	4	19	33.9520	206.5955
eat-4	6	2	4	20	54.5061	195.1250
eat-4	6	2	4	21	75.3573	186.9015
eat-4	6	2	4	22	80.6147	3.3757
eat-4	6	2	4	23	98.9416	150.5113
eat-4	6	2	4	24	40.9691	193.2396
eat-4	6	2	4	25	54.6889	280.3342
eat-4	6	2	4	26	59.5894	176.3221
eat-4	6	2	4	27	91.4824	154.0612
eat-4	6	2	4	28	79.4759	79.0709
eat-4	6	2	4	29	128.4224	156.5278
eat-4	6	2	4	30	39.2121	194.6930
eat-4	6	2	4	31	95.2562	129.0044
eat-4	6	2	4	32	36.7275	219.8921
eat-4	6	2	4	33	136.3005	191.3940
eat-4	6	2	4	34	27.0217	226.8708
eat-4	6	2	4	35	29.3600	239.7177
eat-4	6	2	4	36	72.7113	206.2591
eat-4	6	2	4	37	74.4038	227.4099
eat-4	6	2	4	38	19.8127	179.7808
eat-4	6	2	4	39	14.4154	150.4092
eat-4	6	3	1	1	62.1036	168.1922
eat-4	6	3	1	2	68.5079	147.2178
eat-4	6	3	1	3	63.5455	130.5812
eat-4	6	3	1	4	30.9046	273.3093
eat-4	6	3	1	5	25.2819	207.6171
eat-4	6	3	1	6	19.8398	236.9716
eat-4	6	3	1	7	84.1917	94.5538
eat-4	6	3	1	8	73.7782	175.4328
eat-4	6	3	1	9	42.1172	185.8053
eat-4	6	3	1	10	97.0823	34.6458
eat-4	6	3	1	11	72.4404	180.9882
eat-4	6	3	1	12	70.6078	166.0915

eat-4	6	3	1	13	61.0454	149.8322
eat-4	6	3	1	14	73.7786	29.9302
eat-4	6	3	1	15	71.0757	142.5657
eat-4	6	3	1	16	68.3820	209.2808
eat-4	6	3	1	17	76.9410	160.9230
eat-4	6	3	1	18	20.1963	262.8731
eat-4	6	3	2	1	47.7699	160.3829
eat-4	6	3	2	2	30.6136	231.3301
eat-4	6	3	2	3	66.1399	169.5354
eat-4	6	3	2	4	134.1327	50.1739
eat-4	6	3	2	5	92.8613	98.1446
eat-4	6	3	2	6	82.7934	64.8219
eat-4	6	3	2	7	34.1468	231.5439
eat-4	6	3	2	8	46.9358	202.5730
eat-4	6	3	2	9	60.9241	114.2495
eat-4	6	3	2	10	64.9471	101.6411
eat-4	6	3	2	11	72.6312	128.5786
eat-4	6	3	2	12	74.6210	172.2510
eat-4	6	3	2	13	46.7426	216.4607
eat-4	6	3	2	14	48.6579	93.6584
eat-4	6	3	2	15	48.4289	207.8350
eat-4	6	3	2	16	55.4547	200.8416
eat-4	6	3	2	17	67.7820	228.3100
eat-4	6	3	2	18	86.8197	62.1698
eat-4	6	3	2	19	14.5681	103.6378
eat-4	6	3	2	20	60.6592	39.5263
eat-4	6	3	2	21	61.3047	232.0076
eat-4	6	3	3	1	66.1303	30.0325
eat-4	6	3	3	2	25.0747	196.0109
eat-4	6	3	3	3	27.3354	224.7090
eat-4	6	3	3	4	24.9347	184.1348
eat-4	6	3	3	5	92.5478	74.1443
eat-4	6	3	3	6	24.0667	224.7679
eat-4	6	3	3	7	146.7707	132.9565
eat-4	6	3	3	8	33.4111	224.6562
eat-4	6	3	3	9	27.9802	183.2180
eat-4	6	3	3	10	135.6276	47.3743
eat-4	6	3	3	11	9.2630	199.4120
eat-4	6	3	3	12	56.9953	87.3694
eat-4	6	3	3	13	20.4688	209.0612
eat-4	6	3	3	14	93.1015	48.7402
eat-4	6	3	3	15	28.7107	238.5732
eat-4	6	3	3	16	87.7762	146.2696
eat-4	6	3	3	17	72.1971	97.1722
eat-4	6	3	3	18	42.1631	183.5369
eat-4	6	3	3	19	43.9070	229.1260
eat-4	6	3	3	20	86.0587	68.2976
eat-4	6	3	3	21	56.8311	92.6835
eat-4	6	3	3	22	62.9995	86.5854
eat-4	6	3	3	23	109.9911	51.6540
eat-4	6	3	3	24	28.7752	188.9918
eat-4	6	3	3	25	99.0997	51.4238
eat-4	6	3	3	26	57.8927	190.2468
eat-4	6	3	3	27	63.9871	49.9210
eat-4	6	3	3	28	68.8234	42.2676
eat-4	6	3	3	29	63.2375	178.4239
eat-4	6	3	3	30	53.0209	125.0487
eat-4	6	3	3	31	35.4974	143.5936
eat-4	6	3	3	32	17.3516	108.7312
eat-4	6	3	3	33	79.0195	66.9672
eat-4	6	3	3	34	25.1082	226.1531
eat-4	6	3	4	1	54.1992	44.9651
eat-4	6	3	4	2	23.2511	200.7693
eat-4	6	3	4	3	32.9399	162.0999
eat-4	6	3	4	4	85.1090	87.4578
eat-4	6	3	4	5	44.2273	179.4594
eat-4	6	3	4	6	6.5800	193.8388
eat-4	6	3	4	7	13.8754	175.1386

eat-4	6	3	4	8	74.4827	53.0655
eat-4	6	3	4	9	14.0116	187.0706
eat-4	6	3	4	10	40.1393	97.1277
eat-4	6	3	4	11	49.4531	195.0543
eat-4	6	3	4	12	38.5518	105.0335
eat-4	6	3	4	13	96.3199	82.1369
eat-4	6	3	4	14	18.8305	234.8860
eat-4	6	3	4	15	105.4732	123.8619
eat-4	6	3	4	16	70.4126	138.3920
eat-4	6	3	4	17	163.0757	77.6691
eat-4	6	3	4	18	60.6312	59.5696
eat-4	6	3	4	19	124.2631	73.7415
eat-4	6	3	4	20	105.9723	40.9381
eat-4	6	3	4	21	48.0369	221.5869
eat-4	6	3	4	22	87.6887	18.6069
eat-4	6	3	4	23	87.2434	26.2527
eat-4	6	3	4	24	146.2291	89.6477
eat-4	6	3	4	25	27.7793	241.8403
eat-4	6	3	4	26	89.4778	54.4336
eat-4	6	3	4	27	57.7654	219.5727
eat-4	6	3	4	28	49.3025	179.6106
eat-4	6	3	4	29	30.9649	201.4049
eat-4	6	3	4	30	23.2471	230.1306
eat-4	6	3	4	31	82.4293	64.3194
eat-4	6	3	4	32	80.2490	87.6202
eat-4	6	3	4	33	28.2517	209.0044
eat-4	6	3	4	34	48.0022	79.0637
eat-4	6	3	4	35	30.3766	164.5685
eat-4	6	3	4	36	106.0879	124.3322
eat-4	6	3	4	37	76.1157	47.6944
eat-4	6	3	4	38	25.5469	277.6054
eat-4	6	3	4	39	12.1093	193.9318
eat-4	6	3	4	40	105.4378	70.3646
eat-4	6	3	4	41	70.8833	118.8968
eat-4	6	3	4	42	51.4806	160.6422
eat-4	6	3	4	43	122.1231	207.0469
str-2	0	1	1	1	85.5604	216.7996
str-2	0	1	1	2	54.9237	243.3883
str-2	0	1	1	3	77.4241	44.8116
str-2	0	1	1	4	88.2333	2.7928
str-2	0	1	1	5	118.3617	113.1714
str-2	0	1	1	6	81.5738	55.4712
str-2	0	1	1	7	99.5404	96.4757
str-2	0	1	1	8	83.9956	150.8969
str-2	0	1	1	9	75.3795	141.3913
str-2	0	1	1	10	103.6488	128.2083
str-2	0	1	1	11	134.0833	281.5870
str-2	0	1	1	12	133.2254	151.8088
str-2	0	1	1	13	77.2897	84.2381
str-2	0	1	1	14	108.7248	82.2741
str-2	0	1	1	15	59.1354	147.5250
str-2	0	1	1	16	90.7645	138.1828
str-2	0	1	1	17	34.3290	142.5408
str-2	0	1	1	18	107.9939	111.3652
str-2	0	1	1	19	65.7321	16.6408
str-2	0	1	1	20	111.4521	171.8530
str-2	0	1	2	1	29.5360	312.7855
str-2	0	1	2	2	142.5834	225.6816
str-2	0	1	2	3	77.3558	223.6881
str-2	0	1	2	4	129.5684	131.1063
str-2	0	1	2	5	98.6734	147.5956
str-2	0	1	2	6	74.8768	1.7576
str-2	0	1	2	7	91.1416	177.3084
str-2	0	1	2	8	83.0228	85.7088
str-2	0	1	2	9	82.6385	52.1726
str-2	0	1	2	10	62.4181	142.5296
str-2	0	1	2	11	96.5187	127.6374
str-2	0	1	2	12	136.5495	280.7970

str-2	0	1	2	13	91.9389	127.4463
str-2	0	1	2	14	109.7334	174.3379
str-2	0	1	2	15	79.5717	203.7624
str-2	0	1	2	16	89.9963	176.6459
str-2	0	1	2	17	71.6077	129.8681
str-2	0	1	2	18	74.2016	153.1870
str-2	0	1	2	19	85.4503	283.8216
str-2	0	1	2	20	79.0427	114.2105
str-2	0	1	2	21	39.7862	177.0125
str-2	0	1	2	22	91.9504	120.2768
str-2	0	1	2	23	106.4944	192.3114
str-2	0	1	3	1	77.9081	221.0418
str-2	0	1	3	2	77.6329	18.3742
str-2	0	1	3	3	52.9883	157.0330
str-2	0	1	3	4	103.4188	1.9208
str-2	0	1	3	5	90.2132	1.8851
str-2	0	1	3	6	62.4360	185.8981
str-2	0	1	3	7	18.5363	169.1977
str-2	0	1	3	8	52.0530	158.4143
str-2	0	1	3	9	140.9466	51.8317
str-2	0	1	3	10	22.1897	102.9211
str-2	0	1	3	11	110.3284	82.5447
str-2	0	1	3	12	88.3455	81.7522
str-2	0	1	3	13	80.4245	107.6077
str-2	0	1	3	14	87.2385	230.0139
str-2	0	1	3	15	170.1716	144.0570
str-2	0	1	3	16	156.2566	183.2091
str-2	0	1	3	17	36.3080	264.8786
str-2	0	1	3	18	115.0655	221.0941
str-2	0	1	3	19	127.9409	222.8103
str-2	0	1	3	20	91.9742	57.1952
str-2	0	1	4	1	38.2691	239.4605
str-2	0	1	4	2	20.1289	185.3143
str-2	0	1	4	3	115.0743	196.8313
str-2	0	1	4	4	80.7614	67.2031
str-2	0	1	4	5	93.2771	1.5998
str-2	0	1	4	6	69.0657	186.3859
str-2	0	1	4	7	113.4444	181.2252
str-2	0	1	4	8	93.9218	161.3262
str-2	0	1	4	9	56.0225	167.0053
str-2	0	1	4	10	90.5341	137.6845
str-2	0	1	4	11	116.2720	172.1785
str-2	0	1	4	12	162.2426	78.5854
str-2	0	1	4	13	160.0153	49.6447
str-2	0	1	4	14	33.1592	260.3865
str-2	0	1	4	15	113.8508	203.6738
str-2	0	1	4	16	100.4585	207.3385
str-2	0	1	4	17	149.2499	62.4361
str-2	0	1	4	18	86.3102	2.0834
str-2	0	1	4	19	122.4443	215.9715
str-2	0	1	4	20	77.8551	229.9397
str-2	0	1	4	21	73.0652	277.8240
str-2	0	2	1	1	93.4071	154.1923
str-2	0	2	1	2	95.2983	3.3098
str-2	0	2	1	3	79.4983	141.8681
str-2	0	2	1	4	84.5349	146.4024
str-2	0	2	1	5	73.2359	171.4562
str-2	0	2	1	6	104.6516	84.0822
str-2	0	2	1	7	62.7542	96.6805
str-2	0	2	1	8	133.6421	143.8919
str-2	0	2	1	9	96.9411	111.6763
str-2	0	2	1	10	87.7218	184.1235
str-2	0	2	1	11	75.9407	169.6026
str-2	0	2	1	12	76.8452	83.5941
str-2	0	2	2	1	32.3746	132.5322
str-2	0	2	2	2	82.7049	2.0118
str-2	0	2	2	3	99.8376	91.3254
str-2	0	2	2	4	69.0680	14.7418

str-2	0	2	2	5	108.9806	84.2320
str-2	0	2	2	6	87.6348	99.9728
str-2	0	2	2	7	97.6869	62.3810
str-2	0	2	2	8	129.5192	193.9121
str-2	0	2	2	9	71.8879	150.0075
str-2	0	2	2	10	59.5593	120.4443
str-2	0	2	2	11	84.7161	107.5807
str-2	0	2	2	12	145.8736	91.3011
str-2	0	2	2	13	90.2350	96.9997
str-2	0	2	2	14	118.6622	130.2284
str-2	0	2	3	1	41.5754	50.3644
str-2	0	2	3	2	31.0265	142.8728
str-2	0	2	3	3	110.6229	100.0521
str-2	0	2	3	4	90.1182	2.5281
str-2	0	2	3	5	86.8403	152.9320
str-2	0	2	3	6	99.2661	54.6297
str-2	0	2	3	7	117.1298	109.6516
str-2	0	2	3	8	97.2097	15.3939
str-2	0	2	3	9	115.8954	126.9068
str-2	0	2	3	10	25.1320	119.9146
str-2	0	2	3	11	95.2461	196.6356
str-2	0	2	3	12	112.4245	142.4794
str-2	0	2	3	13	88.5571	108.0096
str-2	0	2	3	14	103.9359	154.5268
str-2	0	2	3	15	70.2604	166.5023
str-2	0	2	3	16	101.3058	97.2697
str-2	0	2	3	17	12.2673	139.6866
str-2	0	2	4	1	85.0473	81.3215
str-2	0	2	4	2	57.7663	206.3645
str-2	0	2	4	3	84.3515	3.1530
str-2	0	2	4	4	97.5816	67.8542
str-2	0	2	4	5	71.8585	125.6436
str-2	0	2	4	6	139.9030	213.6487
str-2	0	2	4	7	65.7088	133.6192
str-2	0	2	4	8	51.4955	104.3037
str-2	0	2	4	9	87.9802	132.3288
str-2	0	2	4	10	152.5031	198.4639
str-2	0	2	4	11	65.1587	242.6264
str-2	0	2	4	12	145.6445	192.7625
str-2	0	2	4	13	93.3843	9.6369
str-2	0	2	4	14	41.7432	97.2057
str-2	0	2	4	15	85.6345	129.5909
str-2	0	2	4	16	84.8455	141.4391
str-2	0	2	4	17	74.8208	25.2831
str-2	0	2	4	18	105.3355	95.7683
str-2	0	2	4	19	105.8573	144.7322
str-2	0	2	4	20	108.5993	55.4136
str-2	0	3	1	1	83.8607	77.0614
str-2	0	3	1	2	102.5705	137.2876
str-2	0	3	1	3	96.7486	132.9101
str-2	0	3	1	4	122.6338	150.7907
str-2	0	3	1	5	63.8706	87.2282
str-2	0	3	1	6	85.1469	80.4004
str-2	0	3	1	7	75.2106	75.6192
str-2	0	3	1	8	65.9978	297.4935
str-2	0	3	1	9	103.3645	155.5995
str-2	0	3	1	10	95.4835	144.5590
str-2	0	3	1	11	67.7320	222.9260
str-2	0	3	1	12	93.4372	120.5155
str-2	0	3	1	13	53.8007	196.7557
str-2	0	3	1	14	82.6557	295.5469
str-2	0	3	1	15	101.3459	66.7455
str-2	0	3	1	16	45.6841	97.9334
str-2	0	3	1	17	108.3235	128.6857
str-2	0	3	2	1	32.7296	269.8902
str-2	0	3	2	2	62.5973	166.3829
str-2	0	3	2	3	104.4709	150.5558
str-2	0	3	2	4	69.7115	118.6557

str-2	0	3	2	5	79.8575	49.6850
str-2	0	3	2	6	152.1833	247.3730
str-2	0	3	2	7	67.1290	78.8119
str-2	0	3	2	8	73.0422	56.8555
str-2	0	3	2	9	146.7861	234.1530
str-2	0	3	2	10	66.7296	182.2171
str-2	0	3	2	11	129.5064	85.7585
str-2	0	3	2	12	112.6844	41.3016
str-2	0	3	2	13	46.4595	111.9745
str-2	0	3	2	14	79.9383	139.2968
str-2	0	3	2	15	97.8509	139.9451
str-2	0	3	2	16	78.1500	147.5975
str-2	0	3	2	17	69.3396	43.8797
str-2	0	3	2	18	119.9578	200.5024
str-2	0	3	2	19	140.9795	187.9422
str-2	0	3	3	1	73.9999	155.4940
str-2	0	3	3	2	105.4738	236.2663
str-2	0	3	3	3	99.8742	43.6998
str-2	0	3	3	4	39.5262	146.8549
str-2	0	3	3	5	24.6050	162.4195
str-2	0	3	3	6	109.4713	94.5418
str-2	0	3	3	7	126.0694	124.3963
str-2	0	3	3	8	75.1382	45.5762
str-2	0	3	3	9	132.7199	12.7093
str-2	0	3	3	10	104.8868	199.4944
str-2	0	3	3	11	52.9449	47.1311
str-2	0	3	3	12	161.0379	167.4715
str-2	0	3	4	1	132.6810	86.3685
str-2	0	3	4	2	98.5395	132.5344
str-2	0	3	4	3	128.0601	11.9182
str-2	0	3	4	4	9.9615	172.0122
str-2	0	3	4	5	116.2531	140.5867
str-2	0	3	4	6	50.3244	94.1394
str-2	0	3	4	7	84.8856	87.5638
str-2	0	3	4	8	37.5185	175.1602
str-2	0	3	4	9	72.2689	159.0796
str-2	0	3	4	10	118.8999	130.0851
str-2	0	3	4	11	37.3354	151.5331
str-2	0	3	4	12	83.8448	181.0227
str-2	1.5	1	1	1	9.2151	229.2855
str-2	1.5	1	1	2	18.6121	182.8427
str-2	1.5	1	1	3	35.8224	106.4268
str-2	1.5	1	1	4	9.8459	191.0673
str-2	1.5	1	1	5	62.9177	126.2894
str-2	1.5	1	1	6	13.3275	225.5070
str-2	1.5	1	1	7	8.9731	215.4286
str-2	1.5	1	1	8	6.4885	132.9704
str-2	1.5	1	1	9	64.1498	31.9339
str-2	1.5	1	1	10	26.4123	194.2748
str-2	1.5	1	1	11	38.0716	285.4933
str-2	1.5	1	1	12	34.3315	267.6942
str-2	1.5	1	1	13	33.0995	179.8956
str-2	1.5	1	1	14	13.5954	240.2335
str-2	1.5	1	2	1	6.1310	261.9680
str-2	1.5	1	2	2	10.3577	194.2245
str-2	1.5	1	2	3	4.4590	272.2461
str-2	1.5	1	2	4	18.3070	166.6197
str-2	1.5	1	2	5	7.7868	185.3412
str-2	1.5	1	2	6	7.1057	157.5077
str-2	1.5	1	2	7	8.3745	295.7991
str-2	1.5	1	2	8	16.2690	104.9552
str-2	1.5	1	2	9	6.8498	160.0515
str-2	1.5	1	2	10	7.3310	191.8299
str-2	1.5	1	2	11	24.3531	180.1044
str-2	1.5	1	2	12	14.2943	172.8616
str-2	1.5	1	2	13	13.8627	259.5801
str-2	1.5	1	2	14	7.0385	249.2109
str-2	1.5	1	2	15	22.9753	145.7095

str-2	1.5	1	2	16	6.0387	196.0921
str-2	1.5	1	2	17	23.0167	218.9105
str-2	1.5	1	2	18	11.1735	189.5600
str-2	1.5	1	2	19	3.5061	217.1032
str-2	1.5	1	2	20	19.2704	175.4749
str-2	1.5	1	3	1	7.7022	192.7966
str-2	1.5	1	3	2	12.7184	298.7985
str-2	1.5	1	3	3	7.0479	262.8349
str-2	1.5	1	3	4	27.4962	173.4510
str-2	1.5	1	3	5	36.3359	164.3470
str-2	1.5	1	3	6	8.2350	199.4232
str-2	1.5	1	3	7	16.6340	66.8162
str-2	1.5	1	3	8	5.6527	165.6925
str-2	1.5	1	3	9	22.0089	328.1090
str-2	1.5	1	3	10	8.8904	152.5442
str-2	1.5	1	3	11	4.7723	240.9273
str-2	1.5	1	3	12	12.3142	192.4078
str-2	1.5	1	3	13	17.8907	248.0633
str-2	1.5	1	3	14	19.2080	162.0509
str-2	1.5	1	3	15	29.6729	202.3106
str-2	1.5	1	3	16	9.3013	213.2377
str-2	1.5	1	3	17	10.0032	242.0934
str-2	1.5	1	3	18	13.1991	205.5218
str-2	1.5	1	3	19	13.0319	225.0808
str-2	1.5	1	3	20	18.9371	199.4633
str-2	1.5	1	3	21	11.4488	197.0709
str-2	1.5	1	3	22	45.0610	88.8466
str-2	1.5	1	3	23	9.0732	202.3075
str-2	1.5	1	3	24	10.1415	279.8266
str-2	1.5	1	3	25	72.0871	6.9930
str-2	1.5	1	3	26	23.4292	227.9221
str-2	1.5	1	3	27	36.2349	196.4018
str-2	1.5	1	3	28	24.6429	188.4466
str-2	1.5	1	3	29	46.6735	170.6507
str-2	1.5	1	3	30	6.9403	212.4528
str-2	1.5	1	3	31	11.5352	138.9166
str-2	1.5	1	3	32	5.7181	163.0343
str-2	1.5	1	3	33	104.0987	10.2098
str-2	1.5	1	3	34	33.7591	124.0196
str-2	1.5	1	3	35	28.6770	273.3080
str-2	1.5	1	3	36	5.9012	186.8857
str-2	1.5	1	4	1	19.5578	218.2311
str-2	1.5	1	4	2	30.9338	162.0715
str-2	1.5	1	4	3	6.8535	153.4207
str-2	1.5	1	4	4	35.5848	108.5991
str-2	1.5	1	4	5	9.2761	219.7601
str-2	1.5	1	4	6	26.8351	258.6280
str-2	1.5	1	4	7	24.6448	47.0882
str-2	1.5	1	4	8	5.0156	225.0036
str-2	1.5	1	4	9	5.4686	186.6067
str-2	1.5	1	4	10	13.5385	239.0183
str-2	1.5	1	4	11	18.5363	160.8052
str-2	1.5	1	4	12	11.0506	115.8527
str-2	1.5	1	4	13	13.3479	256.6158
str-2	1.5	1	4	14	13.4680	160.2738
str-2	1.5	1	4	15	6.6769	160.7036
str-2	1.5	1	4	16	5.9540	188.9525
str-2	1.5	1	4	17	90.7278	17.8176
str-2	1.5	1	4	18	5.3396	204.7641
str-2	1.5	1	4	19	11.4142	237.7237
str-2	1.5	1	4	20	15.6807	118.7627
str-2	1.5	1	4	21	17.4930	219.8471
str-2	1.5	1	4	22	9.5296	168.7359
str-2	1.5	1	4	23	45.0871	90.8159
str-2	1.5	1	4	24	68.0495	103.8074
str-2	1.5	1	4	25	19.7878	51.9015
str-2	1.5	1	4	26	5.6447	226.9866
str-2	1.5	1	4	27	10.1835	229.0380

str-2	1.5	1	4	28	30.6049	178.1064
str-2	1.5	1	4	29	11.8128	138.7755
str-2	1.5	2	4	30	40.8632	166.6877
str-2	1.5	2	1	1	15.2055	169.3618
str-2	1.5	2	1	2	39.5676	173.8835
str-2	1.5	2	1	3	18.6860	335.5495
str-2	1.5	2	1	4	47.1001	220.7560
str-2	1.5	2	1	5	10.4282	214.7624
str-2	1.5	2	1	6	66.9282	96.0833
str-2	1.5	2	1	7	7.8685	283.8397
str-2	1.5	2	1	8	64.2708	117.7303
str-2	1.5	2	1	9	14.8904	234.7173
str-2	1.5	2	1	10	16.7923	333.5286
str-2	1.5	2	2	1	10.3583	286.8399
str-2	1.5	2	2	2	3.8850	257.6724
str-2	1.5	2	2	3	9.8443	385.2497
str-2	1.5	2	2	4	22.4672	263.9109
str-2	1.5	2	2	5	6.5242	315.3766
str-2	1.5	2	2	6	5.2411	231.3486
str-2	1.5	2	2	7	9.5930	279.5540
str-2	1.5	2	2	8	12.5818	316.9870
str-2	1.5	2	2	9	6.4614	303.3905
str-2	1.5	2	2	10	26.7892	206.0214
str-2	1.5	2	2	11	9.5769	244.6562
str-2	1.5	2	2	12	38.0942	172.9125
str-2	1.5	2	2	13	8.0798	339.9081
str-2	1.5	2	2	14	28.8568	223.5377
str-2	1.5	2	2	15	8.7505	171.3815
str-2	1.5	2	2	16	16.2711	265.0771
str-2	1.5	2	2	17	79.6343	66.9603
str-2	1.5	2	2	18	42.5983	207.9854
str-2	1.5	2	2	19	40.0506	46.0641
str-2	1.5	2	2	20	26.7228	374.1432
str-2	1.5	2	2	21	17.5716	200.2187
str-2	1.5	2	2	22	8.1477	341.8982
str-2	1.5	2	2	23	33.6614	200.8589
str-2	1.5	2	2	24	16.6041	369.7279
str-2	1.5	2	2	25	18.0995	204.0977
str-2	1.5	2	2	26	18.9530	286.5996
str-2	1.5	2	2	27	37.1750	271.3748
str-2	1.5	2	2	28	5.7045	142.2390
str-2	1.5	2	2	29	8.3292	391.7937
str-2	1.5	2	2	30	12.1877	245.6506
str-2	1.5	2	2	31	6.6023	300.2991
str-2	1.5	2	2	32	10.4473	251.1736
str-2	1.5	2	2	33	38.9089	196.3728
str-2	1.5	2	3	1	8.7000	243.1951
str-2	1.5	2	3	2	31.7970	114.6835
str-2	1.5	2	3	3	42.7601	183.9192
str-2	1.5	2	3	4	10.0063	268.2817
str-2	1.5	2	3	5	13.7256	198.4424
str-2	1.5	2	3	6	66.8257	67.2407
str-2	1.5	2	3	7	7.9023	189.2235
str-2	1.5	2	3	8	63.4307	198.4240
str-2	1.5	2	3	9	6.9669	109.9303
str-2	1.5	2	3	10	36.5955	186.0858
str-2	1.5	2	3	11	59.4876	184.0786
str-2	1.5	2	3	12	41.9674	182.9443
str-2	1.5	2	3	13	16.6714	179.0267
str-2	1.5	2	3	14	16.3780	325.8364
str-2	1.5	2	3	15	11.2564	147.0524
str-2	1.5	2	3	16	10.3969	208.7368
str-2	1.5	2	3	17	41.7770	175.3265
str-2	1.5	2	3	18	9.1976	183.6089
str-2	1.5	2	3	19	10.7239	203.4042
str-2	1.5	2	3	20	8.0251	261.1808
str-2	1.5	2	3	21	25.5887	66.1277
str-2	1.5	2	3	22	7.4833	261.3636

str-2	1.5	2	3	23	7.8970	356.5866
str-2	1.5	2	3	24	7.9154	289.3676
str-2	1.5	2	3	25	10.9232	203.7396
str-2	1.5	2	3	26	10.3208	240.3647
str-2	1.5	2	4	1	18.1153	151.8882
str-2	1.5	2	4	2	5.8493	262.0788
str-2	1.5	2	4	3	39.2935	212.2056
str-2	1.5	2	4	4	6.2839	254.6615
str-2	1.5	2	4	5	6.7850	212.9029
str-2	1.5	2	4	6	7.9822	274.4559
str-2	1.5	2	4	7	6.8299	215.2691
str-2	1.5	2	4	8	7.7396	102.6378
str-2	1.5	2	4	9	6.3875	200.1176
str-2	1.5	2	4	10	59.4831	44.3342
str-2	1.5	2	4	11	83.8493	138.3539
str-2	1.5	2	4	12	85.7168	54.7683
str-2	1.5	2	4	13	41.4948	149.4060
str-2	1.5	2	4	14	14.3020	218.4192
str-2	1.5	2	4	15	15.7517	181.9098
str-2	1.5	2	4	16	20.3235	205.0788
str-2	1.5	2	4	17	4.2176	225.4103
str-2	1.5	2	4	18	8.8786	228.8440
str-2	1.5	2	4	19	11.2740	246.4034
str-2	1.5	2	4	20	6.5577	249.2278
str-2	1.5	2	4	21	24.4086	73.1546
str-2	1.5	2	4	22	4.4764	213.2896
str-2	1.5	2	4	23	30.3740	163.3681
str-2	1.5	2	4	24	78.3232	92.3989
str-2	1.5	2	4	25	33.2262	117.8700
str-2	1.5	3	1	1	21.8244	297.4997
str-2	1.5	3	1	2	44.7012	209.0504
str-2	1.5	3	1	3	10.7704	243.7309
str-2	1.5	3	1	4	18.3842	225.7263
str-2	1.5	3	1	5	7.4449	252.1537
str-2	1.5	3	1	6	11.4295	337.2005
str-2	1.5	3	1	7	7.7602	329.7776
str-2	1.5	3	1	8	105.0499	5.6964
str-2	1.5	3	1	9	74.7808	205.7658
str-2	1.5	3	1	10	49.5177	207.7954
str-2	1.5	3	1	11	7.1898	325.0496
str-2	1.5	3	2	1	10.3222	325.6238
str-2	1.5	3	2	2	8.2874	240.4746
str-2	1.5	3	2	3	8.3032	319.8886
str-2	1.5	3	2	4	5.8409	309.6350
str-2	1.5	3	2	5	9.7750	291.0633
str-2	1.5	3	2	6	6.4937	332.5669
str-2	1.5	3	2	7	12.6192	255.8009
str-2	1.5	3	2	8	10.2477	201.6276
str-2	1.5	3	2	9	5.3058	280.8740
str-2	1.5	3	2	10	29.1602	201.4089
str-2	1.5	3	2	11	7.2490	269.1773
str-2	1.5	3	2	12	24.2569	299.1427
str-2	1.5	3	2	13	9.1170	275.7779
str-2	1.5	3	2	14	76.1887	7.0049
str-2	1.5	3	2	15	29.7832	225.1431
str-2	1.5	3	2	16	5.4900	239.9479
str-2	1.5	3	2	17	24.2134	202.4655
str-2	1.5	3	2	18	10.5083	262.9156
str-2	1.5	3	2	19	11.1604	229.0793
str-2	1.5	3	3	1	18.0563	302.2827
str-2	1.5	3	3	2	7.0237	291.4882
str-2	1.5	3	3	3	16.6287	305.2866
str-2	1.5	3	3	4	19.1385	319.0532
str-2	1.5	3	3	5	9.9658	182.6385
str-2	1.5	3	3	6	10.6401	234.6841
str-2	1.5	3	3	7	6.7962	262.4927
str-2	1.5	3	3	8	6.6417	281.6511
str-2	1.5	3	3	9	12.8646	241.2690

str-2	1.5	3	3	10	6.4667	282.3603
str-2	1.5	3	3	11	7.3005	242.2227
str-2	1.5	3	3	12	16.1318	236.7360
str-2	1.5	3	3	13	95.2767	243.9530
str-2	1.5	3	3	14	26.0585	222.6973
str-2	1.5	3	3	15	23.7772	266.8578
str-2	1.5	3	3	16	8.2586	260.3749
str-2	1.5	3	3	17	69.4291	128.8384
str-2	1.5	3	3	18	65.0936	202.2268
str-2	1.5	3	3	19	92.8410	6.6032
str-2	1.5	3	3	20	14.4867	104.7420
str-2	1.5	3	3	21	10.3995	192.1216
str-2	1.5	3	3	22	22.5512	239.5571
str-2	1.5	3	3	23	23.8942	218.6009
str-2	1.5	3	3	24	7.7679	309.1160
str-2	1.5	3	3	25	5.1544	240.4435
str-2	1.5	3	3	26	10.0799	214.2467
str-2	1.5	3	4	1	9.1225	254.9839
str-2	1.5	3	4	2	15.6883	285.3246
str-2	1.5	3	4	3	8.6977	247.7677
str-2	1.5	3	4	4	9.0903	236.0894
str-2	1.5	3	4	5	6.7600	286.6188
str-2	1.5	3	4	6	36.7999	197.6877
str-2	1.5	3	4	7	15.9210	206.7721
str-2	1.5	3	4	8	7.9700	295.6397
str-2	1.5	3	4	9	16.3095	116.7006
str-2	1.5	3	4	10	65.6877	130.2793
str-2	1.5	3	4	11	57.9265	154.7149
str-2	1.5	3	4	12	68.5366	182.9151
str-2	1.5	3	4	13	17.9148	78.3103
str-2	1.5	3	4	14	28.2351	311.4054
str-2	1.5	3	4	15	22.9546	255.6868
str-2	1.5	3	4	16	94.4110	4.3911
str-2	1.5	3	4	17	9.5456	278.1690
str-2	1.5	3	4	18	89.3860	164.0022
str-2	3	1	1	1	20.5140	373.4836
str-2	3	1	1	2	31.2437	358.7283
str-2	3	1	1	3	19.4150	317.0395
str-2	3	1	1	4	31.6680	282.5025
str-2	3	1	1	5	14.3996	287.0956
str-2	3	1	1	6	32.0206	365.6403
str-2	3	1	1	7	6.0779	366.6666
str-2	3	1	1	8	36.0661	290.3613
str-2	3	1	1	9	21.1845	370.7643
str-2	3	1	1	10	29.0017	390.8291
str-2	3	1	1	11	99.4707	35.3208
str-2	3	1	2	1	23.7894	280.1616
str-2	3	1	2	2	22.9915	355.4073
str-2	3	1	2	3	23.0637	252.7138
str-2	3	1	2	4	7.5921	363.6062
str-2	3	1	2	5	29.0745	364.7448
str-2	3	1	2	6	17.1537	315.0905
str-2	3	1	2	7	21.7625	126.3358
str-2	3	1	2	8	34.3337	282.7197
str-2	3	1	2	9	103.7507	29.5083
str-2	3	1	2	10	29.5320	278.7079
str-2	3	1	2	11	42.9391	266.2337
str-2	3	1	2	12	14.0113	285.1304
str-2	3	1	2	13	16.2419	355.6738
str-2	3	1	2	14	22.6488	273.7475
str-2	3	1	2	15	66.6966	28.7216
str-2	3	1	2	16	16.9174	254.1606
str-2	3	1	2	17	18.1951	211.7305
str-2	3	1	2	18	29.0894	174.5262
str-2	3	1	2	19	22.4940	160.7481
str-2	3	1	2	20	29.6072	152.4548
str-2	3	1	2	21	27.6171	311.4760
str-2	3	1	2	22	47.1162	74.3928

str-2	3	1	3	1	40.6496	310.6936
str-2	3	1	3	2	11.7883	341.3703
str-2	3	1	3	3	22.8384	239.8113
str-2	3	1	3	4	5.8292	259.6746
str-2	3	1	3	5	11.5083	278.2918
str-2	3	1	3	6	10.4058	248.4789
str-2	3	1	3	7	8.2980	295.9847
str-2	3	1	3	8	12.0600	239.8187
str-2	3	1	3	9	14.5944	203.8724
str-2	3	1	3	10	38.1975	259.2720
str-2	3	1	3	11	13.2141	319.2909
str-2	3	1	3	12	21.0717	358.4911
str-2	3	1	3	13	24.2607	222.5309
str-2	3	1	3	14	23.1092	339.0482
str-2	3	1	3	15	24.5615	224.2085
str-2	3	1	3	16	14.5656	327.8846
str-2	3	1	3	17	33.8798	283.4682
str-2	3	1	3	18	36.6611	258.3106
str-2	3	1	3	19	20.0942	229.1072
str-2	3	1	3	20	26.8532	343.4005
str-2	3	1	3	21	25.7566	107.0147
str-2	3	1	4	1	9.2271	226.5343
str-2	3	1	4	2	28.7430	242.5579
str-2	3	1	4	3	10.5637	360.7606
str-2	3	1	4	4	15.8459	202.7679
str-2	3	1	4	5	11.6283	205.8423
str-2	3	1	4	6	40.0825	269.2924
str-2	3	1	4	7	28.2739	229.2298
str-2	3	1	4	8	9.1680	136.0327
str-2	3	1	4	9	117.3706	9.2100
str-2	3	2	1	1	27.5459	237.9570
str-2	3	2	1	2	36.4478	275.4445
str-2	3	2	1	3	26.1111	278.4348
str-2	3	2	1	4	36.8193	223.1040
str-2	3	2	1	5	38.2431	152.5719
str-2	3	2	1	6	42.1534	201.3728
str-2	3	2	1	7	46.5711	279.3594
str-2	3	2	1	8	15.8665	240.6495
str-2	3	2	1	9	52.0765	224.4834
str-2	3	2	1	10	13.4115	367.1943
str-2	3	2	1	11	47.1377	219.6190
str-2	3	2	1	12	23.2034	231.4595
str-2	3	2	1	13	60.8745	55.6887
str-2	3	2	1	14	42.0824	144.0652
str-2	3	2	2	1	23.8016	228.1836
str-2	3	2	2	2	16.9104	273.8792
str-2	3	2	2	3	25.5312	280.8184
str-2	3	2	2	4	10.3554	215.0603
str-2	3	2	2	5	10.9371	219.2602
str-2	3	2	2	6	22.2301	257.0631
str-2	3	2	2	7	29.1727	278.5390
str-2	3	2	2	8	12.8244	197.9046
str-2	3	2	2	9	22.9877	230.3882
str-2	3	2	2	10	16.5821	275.2312
str-2	3	2	2	11	18.5481	285.6915
str-2	3	2	2	12	66.5705	35.1306
str-2	3	2	2	13	13.0349	183.9627
str-2	3	2	2	14	10.8669	185.3987
str-2	3	2	2	15	47.8038	193.7963
str-2	3	2	2	16	24.8852	204.1869
str-2	3	2	2	17	19.3276	264.9204
str-2	3	2	2	18	22.1208	280.9849
str-2	3	2	2	19	42.7090	212.0389
str-2	3	2	2	20	24.5070	118.0942
str-2	3	2	2	21	26.7662	260.5401
str-2	3	2	2	22	28.1269	145.1301
str-2	3	2	2	23	29.1160	173.3278
str-2	3	2	2	24	35.5269	189.0853

str-2	3	2	2	25	31.1696	205.2755
str-2	3	2	2	26	13.4249	175.2831
str-2	3	2	2	27	29.7888	105.9499
str-2	3	2	3	1	26.2301	169.7222
str-2	3	2	3	2	26.2738	236.3835
str-2	3	2	3	3	17.6091	222.3725
str-2	3	2	3	4	11.1357	245.8990
str-2	3	2	3	5	14.7912	272.3078
str-2	3	2	3	6	29.2270	188.3490
str-2	3	2	3	7	26.1305	137.4305
str-2	3	2	3	8	10.0475	162.1407
str-2	3	2	3	9	19.2115	205.4231
str-2	3	2	3	10	8.9557	283.3233
str-2	3	2	3	11	34.0676	229.7012
str-2	3	2	3	12	16.0143	191.7979
str-2	3	2	3	13	26.2110	238.5376
str-2	3	2	3	14	12.0488	312.9691
str-2	3	2	3	15	16.4250	208.0399
str-2	3	2	3	16	22.8561	178.5619
str-2	3	2	3	17	20.4828	228.1315
str-2	3	2	3	18	39.4801	166.4205
str-2	3	2	3	19	51.5404	198.3445
str-2	3	2	3	20	33.1584	136.6955
str-2	3	2	3	21	16.9862	307.7019
str-2	3	2	3	22	19.2624	147.2012
str-2	3	2	3	23	16.1560	101.5170
str-2	3	2	3	24	43.7132	189.2507
str-2	3	2	3	25	38.0952	252.6323
str-2	3	2	4	1	5.1656	219.1407
str-2	3	2	4	2	6.9012	195.4037
str-2	3	2	4	3	42.1342	207.6745
str-2	3	2	4	4	35.6666	144.2609
str-2	3	2	4	5	22.9797	205.3380
str-2	3	2	4	6	15.4783	219.7673
str-2	3	2	4	7	40.9656	149.1963
str-2	3	2	4	8	78.7829	173.5770
str-2	3	2	4	9	15.8841	206.7487
str-2	3	2	4	10	58.9112	117.8233
str-2	3	2	4	11	10.8807	198.3553
str-2	3	2	4	12	28.1927	189.2277
str-2	3	2	4	13	26.2980	162.6078
str-2	3	2	4	14	13.5038	139.6687
str-2	3	2	4	15	11.4053	114.2293
str-2	3	2	4	16	30.4790	135.2500
str-2	3	3	1	1	24.2696	310.5379
str-2	3	3	1	2	28.4647	312.9452
str-2	3	3	1	3	50.4800	396.9964
str-2	3	3	1	4	22.7660	361.9776
str-2	3	3	1	5	39.4786	316.9230
str-2	3	3	1	6	34.0565	131.3975
str-2	3	3	1	7	54.3286	31.9493
str-2	3	3	1	8	40.3649	229.6293
str-2	3	3	1	9	32.7842	328.1364
str-2	3	3	1	10	22.1642	391.5988
str-2	3	3	1	11	49.6323	123.5947
str-2	3	3	2	1	11.9013	299.4517
str-2	3	3	2	2	37.7610	262.3158
str-2	3	3	2	3	8.9117	303.9239
str-2	3	3	2	4	15.0992	273.2356
str-2	3	3	2	5	25.1317	277.1195
str-2	3	3	2	6	21.8143	134.9806
str-2	3	3	2	7	32.3891	225.7240
str-2	3	3	2	8	22.2317	274.3597
str-2	3	3	2	9	16.4206	286.6623
str-2	3	3	2	10	23.0010	126.3837
str-2	3	3	2	11	35.9118	219.5827
str-2	3	3	2	12	43.8443	160.6658
str-2	3	3	2	13	17.6915	233.9070

str-2	3	3	2	14	11.6350	224.7841
str-2	3	3	2	15	14.8679	267.8310
str-2	3	3	2	16	17.1459	297.5378
str-2	3	3	2	17	41.1828	230.7894
str-2	3	3	2	18	25.9523	274.5497
str-2	3	3	2	19	39.2025	329.1013
str-2	3	3	2	20	91.3176	4.1239
str-2	3	3	2	21	41.5279	180.6852
str-2	3	3	2	22	75.2748	225.9455
str-2	3	3	2	23	20.6559	303.4509
str-2	3	3	2	24	42.4845	184.2029
str-2	3	3	3	1	20.4166	207.1608
str-2	3	3	3	2	25.9632	211.8603
str-2	3	3	3	3	14.1255	266.6752
str-2	3	3	3	4	23.5021	254.7932
str-2	3	3	3	5	22.0126	225.1224
str-2	3	3	3	6	9.0664	301.9335
str-2	3	3	3	7	36.9533	232.4486
str-2	3	3	3	8	50.9351	183.7147
str-2	3	3	3	9	26.0793	267.4940
str-2	3	3	3	10	20.4277	279.5332
str-2	3	3	3	11	8.6813	233.5362
str-2	3	3	3	12	5.1787	205.8262
str-2	3	3	3	13	10.9868	296.7101
str-2	3	3	3	14	46.7600	229.0019
str-2	3	3	3	15	11.6330	294.0776
str-2	3	3	3	16	25.3798	127.6058
str-2	3	3	3	17	31.4981	250.9734
str-2	3	3	3	18	17.2005	342.5972
str-2	3	3	3	19	15.7459	249.2009
str-2	3	3	3	20	24.2417	230.0268
str-2	3	3	4	1	23.9282	317.7303
str-2	3	3	4	2	15.8525	273.4563
str-2	3	3	4	3	17.5297	243.8696
str-2	3	3	4	4	38.1233	187.0063
str-2	3	3	4	5	35.7737	137.8287
str-2	3	3	4	6	23.0525	259.2914
str-2	3	3	4	7	42.5180	159.1190
str-2	3	3	4	8	22.6667	219.8722
str-2	3	3	4	9	18.4314	298.9401
str-2	3	3	4	10	55.9617	120.2087
str-2	3	3	4	11	50.5197	95.4170
str-2	3	3	4	12	23.9760	280.6975
str-2	3	3	4	13	16.8208	268.6031
str-2	3	3	4	14	37.7336	219.3055
str-2	6	1	1	1	46.4424	262.3381
str-2	6	1	1	2	19.5976	316.2146
str-2	6	1	1	3	10.9731	276.7520
str-2	6	1	1	4	31.8800	262.9366
str-2	6	1	1	5	25.2499	270.2489
str-2	6	1	1	6	58.5274	181.7730
str-2	6	1	1	7	72.4733	198.9798
str-2	6	1	1	8	10.3519	297.0020
str-2	6	1	1	9	53.9271	275.9825
str-2	6	1	1	10	35.0980	289.6453
str-2	6	1	2	1	22.3174	203.4526
str-2	6	1	2	2	11.8882	196.8034
str-2	6	1	2	3	22.9002	179.6935
str-2	6	1	2	4	34.7758	325.7030
str-2	6	1	2	5	51.3246	131.1220
str-2	6	1	2	6	19.2994	214.4829
str-2	6	1	2	7	45.4196	183.1932
str-2	6	1	2	8	98.7327	2.4406
str-2	6	1	2	9	30.0402	293.2594
str-2	6	1	2	10	20.6326	167.0598
str-2	6	1	2	11	19.5142	208.3688
str-2	6	1	2	12	30.0175	244.2849
str-2	6	1	2	13	55.4257	270.9982

str-2	6	1	2	14	37.1913	222.1692
str-2	6	1	2	15	28.1181	291.6379
str-2	6	1	2	16	63.0179	179.4102
str-2	6	1	2	17	35.4386	254.2927
str-2	6	1	2	18	17.5739	184.5460
str-2	6	1	2	19	22.1736	188.3532
str-2	6	1	2	20	47.6796	217.0565
str-2	6	1	2	21	39.2737	235.1628
str-2	6	1	2	22	31.0357	199.5930
str-2	6	1	2	23	21.8410	201.5021
str-2	6	1	2	24	43.1789	100.3365
str-2	6	1	2	25	46.8690	199.8812
str-2	6	1	2	26	28.4023	216.1845
str-2	6	1	2	27	17.8018	153.7174
str-2	6	1	3	1	14.6799	149.4365
str-2	6	1	3	2	20.0333	165.6703
str-2	6	1	3	3	43.5696	243.2925
str-2	6	1	3	4	11.5208	122.1905
str-2	6	1	3	5	43.0619	199.1051
str-2	6	1	3	6	14.5451	187.6039
str-2	6	1	3	7	7.7068	161.5267
str-2	6	1	3	8	69.4842	59.5352
str-2	6	1	3	9	17.2319	87.4734
str-2	6	1	3	10	19.5335	158.3159
str-2	6	1	3	11	27.7399	201.2771
str-2	6	1	3	12	29.2217	220.3245
str-2	6	1	3	13	9.6502	174.8993
str-2	6	1	3	14	19.7525	253.1173
str-2	6	1	3	15	58.5562	187.3246
str-2	6	1	3	16	34.5180	136.1674
str-2	6	1	3	17	26.4344	167.0060
str-2	6	1	3	18	16.7290	168.5274
str-2	6	1	3	19	19.4826	207.1402
str-2	6	1	3	20	19.8549	198.2407
str-2	6	1	3	21	24.6870	195.2504
str-2	6	1	3	22	39.0131	111.8719
str-2	6	1	3	23	21.6952	96.3360
str-2	6	1	3	24	49.9826	51.9675
str-2	6	1	3	25	14.6044	151.5358
str-2	6	1	3	26	31.6184	172.2024
str-2	6	1	3	27	38.0136	192.8384
str-2	6	1	4	1	42.9200	202.4852
str-2	6	1	4	2	38.8279	132.4190
str-2	6	1	4	3	20.1868	184.1194
str-2	6	1	4	4	13.1474	183.5289
str-2	6	1	4	5	42.0226	195.5420
str-2	6	1	4	6	53.0802	126.6453
str-2	6	1	4	7	27.9996	204.0528
str-2	6	1	4	8	40.2573	150.0835
str-2	6	1	4	9	19.8565	95.1229
str-2	6	1	4	10	44.8492	132.4229
str-2	6	1	4	11	55.3167	33.7343
str-2	6	1	4	12	47.4306	161.9114
str-2	6	1	4	13	28.0595	108.3378
str-2	6	1	4	14	41.4503	147.7748
str-2	6	1	4	15	20.6896	182.8578
str-2	6	1	4	16	27.0064	191.3881
str-2	6	1	4	17	61.0896	162.6772
str-2	6	1	4	18	29.9175	92.7395
str-2	6	2	1	1	25.9956	218.0404
str-2	6	2	1	2	28.0002	259.2201
str-2	6	2	1	3	53.6343	231.7167
str-2	6	2	1	4	12.3589	196.0124
str-2	6	2	1	5	73.9427	76.2736
str-2	6	2	1	6	12.9550	185.0074
str-2	6	2	2	1	20.5791	131.9642
str-2	6	2	2	2	18.1814	239.8417
str-2	6	2	2	3	13.4453	197.4239

str-2	6	2	2	4	29.1796	217.4424
str-2	6	2	2	5	22.4722	173.0411
str-2	6	2	2	6	27.1363	160.7941
str-2	6	2	2	7	19.0823	153.0984
str-2	6	2	2	8	30.7522	170.3878
str-2	6	2	2	9	28.7592	146.3492
str-2	6	2	2	10	22.0794	74.4679
str-2	6	2	2	11	26.3681	165.1907
str-2	6	2	2	12	19.5517	223.3543
str-2	6	2	2	13	44.2562	192.6679
str-2	6	2	2	14	50.4568	261.1199
str-2	6	2	2	15	56.0651	290.1632
str-2	6	2	2	16	33.8763	141.4961
str-2	6	2	2	17	47.8349	230.7896
str-2	6	2	2	18	22.2497	217.5675
str-2	6	2	2	19	14.9182	171.6368
str-2	6	2	2	20	23.2498	217.4476
str-2	6	2	2	21	24.4954	207.3521
str-2	6	2	2	22	32.3910	159.3542
str-2	6	2	2	23	51.6881	226.1440
str-2	6	2	2	24	44.2925	143.4097
str-2	6	2	2	25	98.3048	190.9366
str-2	6	2	3	1	30.4400	254.4952
str-2	6	2	3	2	19.2246	145.6041
str-2	6	2	3	3	13.8416	222.8757
str-2	6	2	3	4	44.0226	173.8531
str-2	6	2	3	5	26.9524	184.5346
str-2	6	2	3	6	24.7791	130.4037
str-2	6	2	3	7	34.2325	65.6629
str-2	6	2	3	8	13.4845	199.1632
str-2	6	2	3	9	38.1074	214.1789
str-2	6	2	3	10	28.1301	124.6811
str-2	6	2	3	11	31.0634	224.5745
str-2	6	2	3	12	80.1533	86.1752
str-2	6	2	3	13	34.6499	69.2948
str-2	6	2	3	14	84.5660	46.6554
str-2	6	2	3	15	81.7638	320.8056
str-2	6	2	3	16	23.8392	162.4969
str-2	6	2	3	17	42.2313	181.8922
str-2	6	2	3	18	69.2993	409.7387
str-2	6	2	3	19	15.6074	161.6153
str-2	6	2	3	20	18.3680	156.9185
str-2	6	2	3	21	41.4871	150.6525
str-2	6	2	3	22	21.0454	253.6245
str-2	6	2	3	23	30.4866	209.1093
str-2	6	2	4	1	27.4229	165.7830
str-2	6	2	4	2	43.9924	125.4561
str-2	6	2	4	3	54.6058	236.5523
str-2	6	2	4	4	21.4295	141.2712
str-2	6	2	4	5	35.7993	84.5043
str-2	6	2	4	6	17.5886	117.9755
str-2	6	2	4	7	32.3291	145.6544
str-2	6	2	4	8	50.5284	146.1220
str-2	6	2	4	9	51.9975	121.8804
str-2	6	2	4	10	39.8330	158.1938
str-2	6	2	4	11	57.5846	134.8610
str-2	6	2	4	12	23.8400	85.4481
str-2	6	2	4	13	48.0102	143.0957
str-2	6	2	4	14	70.9062	326.3320
str-2	6	2	4	15	38.4112	163.2064
str-2	6	2	4	16	22.8795	255.7265
str-2	6	2	4	17	42.7139	171.4115
str-2	6	2	4	18	82.8351	116.4864
str-2	6	2	4	19	51.6590	195.9706
str-2	6	2	4	20	44.7964	226.8533
str-2	6	3	1	1	36.0072	265.1630
str-2	6	3	1	2	44.6852	261.9789
str-2	6	3	1	3	30.4732	50.5239

str-2	6	3	1	4	64.0734	291.9772
str-2	6	3	1	5	19.1054	293.4053
str-2	6	3	1	6	45.8920	290.1804
str-2	6	3	1	7	14.5705	231.1342
str-2	6	3	1	8	42.5345	344.4299
str-2	6	3	1	9	43.1199	321.4522
str-2	6	3	1	10	41.8259	252.1941
str-2	6	3	2	1	46.6408	270.9555
str-2	6	3	2	2	20.7870	293.1359
str-2	6	3	2	3	27.0397	195.6804
str-2	6	3	2	4	23.0436	193.4416
str-2	6	3	2	5	7.1849	268.9074
str-2	6	3	2	6	41.0939	237.1459
str-2	6	3	2	7	25.6357	270.8100
str-2	6	3	2	8	56.8576	248.1064
str-2	6	3	2	9	23.5730	273.7071
str-2	6	3	2	10	49.7333	238.0313
str-2	6	3	2	11	20.7013	254.2787
str-2	6	3	2	12	24.6743	232.0418
str-2	6	3	2	13	27.4303	122.7749
str-2	6	3	2	14	44.9844	295.8727
str-2	6	3	2	15	35.9927	266.2757
str-2	6	3	2	16	30.4064	161.1077
str-2	6	3	2	17	50.1255	226.7076
str-2	6	3	2	18	18.4056	243.7639
str-2	6	3	2	19	40.5017	190.5372
str-2	6	3	2	20	36.9486	229.1567
str-2	6	3	2	21	34.4046	242.6981
str-2	6	3	2	22	27.9128	243.7987
str-2	6	3	2	23	6.8035	306.7775
str-2	6	3	3	1	59.7080	201.7035
str-2	6	3	3	2	26.5245	160.9113
str-2	6	3	3	3	52.2310	196.9371
str-2	6	3	3	4	30.9622	167.9221
str-2	6	3	3	5	36.6931	201.8838
str-2	6	3	3	6	14.6695	238.1426
str-2	6	3	3	7	22.5948	248.9631
str-2	6	3	3	8	39.3016	112.7271
str-2	6	3	3	9	18.6631	219.6781
str-2	6	3	3	10	80.3379	108.6642
str-2	6	3	3	11	63.3604	42.5093
str-2	6	3	3	12	34.4278	108.7375
str-2	6	3	3	13	75.1329	38.9780
str-2	6	3	3	14	28.9894	163.3756
str-2	6	3	3	15	24.0059	204.5478
str-2	6	3	3	16	23.7605	193.4416
str-2	6	3	3	17	50.8873	183.0367
str-2	6	3	3	18	16.9216	220.5180
str-2	6	3	3	19	40.5450	201.9857
str-2	6	3	3	20	27.9659	241.6182
str-2	6	3	3	21	48.1387	339.8189
str-2	6	3	3	22	82.8518	14.5533
str-2	6	3	3	23	30.3627	273.9622
str-2	6	3	3	24	17.1170	145.1948
str-2	6	3	3	25	50.1959	335.2280
str-2	6	3	3	26	10.9558	185.9953
str-2	6	3	3	27	63.1073	359.1213
str-2	6	3	3	28	21.6493	316.6821
str-2	6	3	3	29	32.9335	154.0708
str-2	6	3	4	1	45.3935	108.8834
str-2	6	3	4	2	42.3752	138.0101
str-2	6	3	4	3	14.7732	174.5651
str-2	6	3	4	4	23.3653	105.7338
str-2	6	3	4	5	55.1003	349.8167
str-2	6	3	4	6	36.7213	228.6477
str-2	6	3	4	7	37.9904	184.9385
str-2	6	3	4	8	58.8208	133.1255
str-2	6	3	4	9	37.3303	140.6816

str-2	6	3	4	10	67.1674	164.3807
str-2	6	3	4	11	64.0912	240.8734
str-2	6	3	4	12	30.8929	210.7722
str-2	6	3	4	13	24.1983	136.4186
str-2	6	3	4	14	60.9946	39.2696
str-2	6	3	4	15	76.1182	81.7303
str-2	6	3	4	16	21.5282	28.3206
str-2	6	3	4	17	85.6488	19.0735
str-2	6	3	4	18	13.1272	248.6733
str-2	6	3	4	19	30.4955	116.8857
str-2	6	3	4	20	37.6754	174.4511
str-2	6	3	4	21	26.9959	130.9944
str-2	6	3	4	22	13.5006	182.4598
str-2	6	3	4	23	39.4666	95.2806
str-2	6	3	4	24	46.1158	191.7385
str-2	6	3	4	25	59.8857	171.4662
AWC::caspase	0	1	1	1	89.2314	154.8845
AWC::caspase	0	1	1	2	127.8337	117.8688
AWC::caspase	0	1	1	3	27.6805	213.2405
AWC::caspase	0	1	1	4	61.5193	116.6846
AWC::caspase	0	1	1	5	121.0048	167.6800
AWC::caspase	0	1	1	6	143.3592	258.3690
AWC::caspase	0	1	1	7	89.6890	145.1211
AWC::caspase	0	1	1	8	66.7622	92.0132
AWC::caspase	0	1	1	9	104.8747	65.0801
AWC::caspase	0	1	1	10	11.6656	170.9844
AWC::caspase	0	1	1	11	85.0318	102.5879
AWC::caspase	0	1	1	12	94.6400	149.6206
AWC::caspase	0	1	2	1	100.3905	185.3323
AWC::caspase	0	1	2	2	73.3072	122.9578
AWC::caspase	0	1	2	3	69.0672	146.1996
AWC::caspase	0	1	2	4	143.4962	131.1229
AWC::caspase	0	1	2	5	123.7638	101.5049
AWC::caspase	0	1	2	6	59.9537	113.0690
AWC::caspase	0	1	2	7	71.2146	222.3273
AWC::caspase	0	1	2	8	166.9625	115.6755
AWC::caspase	0	1	2	9	140.1113	197.0832
AWC::caspase	0	1	2	10	119.7639	205.8269
AWC::caspase	0	1	2	11	106.3419	218.7336
AWC::caspase	0	1	2	12	78.3504	244.3488
AWC::caspase	0	1	2	13	7.2625	193.0531
AWC::caspase	0	1	3	1	96.3769	75.3183
AWC::caspase	0	1	3	2	138.0426	175.0769
AWC::caspase	0	1	3	3	89.0319	175.9572
AWC::caspase	0	1	3	4	145.1626	205.9158
AWC::caspase	0	1	3	5	32.0249	211.6949
AWC::caspase	0	1	3	6	45.7919	128.1320
AWC::caspase	0	1	3	7	85.2411	193.5228
AWC::caspase	0	1	3	8	99.6518	162.3263
AWC::caspase	0	1	3	9	69.2111	194.3363
AWC::caspase	0	1	3	10	86.7477	182.4656
AWC::caspase	0	1	3	11	105.6520	196.0887
AWC::caspase	0	1	3	12	52.5528	255.9501
AWC::caspase	0	1	3	13	146.9584	160.5309
AWC::caspase	0	1	3	14	99.3486	132.9067
AWC::caspase	0	1	3	15	106.0025	75.5459
AWC::caspase	0	1	3	16	41.8999	48.4798
AWC::caspase	0	1	3	17	130.9209	221.4676
AWC::caspase	0	1	3	18	73.1799	172.2118
AWC::caspase	0	1	3	19	21.7013	188.4807
AWC::caspase	0	1	4	1	93.1422	59.6936
AWC::caspase	0	1	4	2	38.4272	73.2521
AWC::caspase	0	1	4	3	119.2700	179.8580
AWC::caspase	0	1	4	4	24.3190	91.7000
AWC::caspase	0	1	4	5	15.5960	131.7170
AWC::caspase	0	1	4	6	164.9872	139.7661
AWC::caspase	0	1	4	7	149.8239	209.2116
AWC::caspase	0	1	4	8	157.7152	229.0569

AWC::caspase	0	1	4	9	144.6475	152.4854
AWC::caspase	0	1	4	10	61.3969	177.9837
AWC::caspase	0	1	4	11	150.4891	178.2999
AWC::caspase	0	1	4	12	21.3483	129.7295
AWC::caspase	0	1	4	13	146.6425	192.8655
AWC::caspase	0	2	1	1	101.7616	145.0825
AWC::caspase	0	2	1	2	22.5585	176.9514
AWC::caspase	0	2	1	3	91.4656	123.6709
AWC::caspase	0	2	1	4	149.8781	27.7141
AWC::caspase	0	2	1	5	99.8897	93.1024
AWC::caspase	0	2	1	6	89.8973	125.4030
AWC::caspase	0	2	1	7	84.4455	54.4521
AWC::caspase	0	2	1	8	69.0688	140.5065
AWC::caspase	0	2	1	9	90.5850	182.4593
AWC::caspase	0	2	1	10	112.2628	38.7496
AWC::caspase	0	2	1	11	63.0981	72.2316
AWC::caspase	0	2	1	12	67.3617	160.3187
AWC::caspase	0	2	2	1	76.9482	174.3679
AWC::caspase	0	2	2	2	51.2633	72.7887
AWC::caspase	0	2	2	3	31.6150	19.3265
AWC::caspase	0	2	2	4	83.8334	32.1301
AWC::caspase	0	2	2	5	90.7639	123.9530
AWC::caspase	0	2	2	6	112.8047	38.2789
AWC::caspase	0	2	2	7	93.2866	70.4795
AWC::caspase	0	2	2	8	113.6028	211.7326
AWC::caspase	0	2	2	9	106.6947	89.6064
AWC::caspase	0	2	2	10	84.1689	117.7624
AWC::caspase	0	2	2	11	100.9272	176.2545
AWC::caspase	0	2	2	12	99.0323	77.8110
AWC::caspase	0	2	2	13	106.2566	33.8257
AWC::caspase	0	2	2	14	98.9504	160.9855
AWC::caspase	0	2	3	1	112.6069	4.2306
AWC::caspase	0	2	3	2	61.0482	102.2729
AWC::caspase	0	2	3	3	62.7394	53.9806
AWC::caspase	0	2	3	4	44.4904	197.6980
AWC::caspase	0	2	3	5	89.3303	142.5489
AWC::caspase	0	2	3	6	67.5283	95.9208
AWC::caspase	0	2	3	7	29.6125	149.1683
AWC::caspase	0	2	3	8	66.4096	170.2777
AWC::caspase	0	2	3	9	109.9338	58.4514
AWC::caspase	0	2	3	10	144.0342	94.4353
AWC::caspase	0	2	3	11	76.1707	74.5349
AWC::caspase	0	2	3	12	50.4753	201.2412
AWC::caspase	0	2	3	13	101.6615	253.3219
AWC::caspase	0	2	3	14	58.6302	144.7801
AWC::caspase	0	2	3	15	91.2074	152.1436
AWC::caspase	0	2	3	16	130.2400	172.1978
AWC::caspase	0	2	3	17	104.4223	139.8624
AWC::caspase	0	2	4	1	111.9799	137.8022
AWC::caspase	0	2	4	2	121.7033	7.8739
AWC::caspase	0	2	4	3	103.1407	77.1239
AWC::caspase	0	2	4	4	16.8752	234.6347
AWC::caspase	0	2	4	5	72.3746	63.8844
AWC::caspase	0	2	4	6	133.1371	179.2320
AWC::caspase	0	2	4	7	106.3142	63.4433
AWC::caspase	0	2	4	8	156.9692	197.1702
AWC::caspase	0	2	4	9	78.1734	57.8908
AWC::caspase	0	2	4	10	98.0991	155.5614
AWC::caspase	0	2	4	11	45.4218	182.8611
AWC::caspase	0	2	4	12	71.7976	106.7744
AWC::caspase	0	2	4	13	95.5283	194.5228
AWC::caspase	0	2	4	14	70.8716	39.8179
AWC::caspase	0	2	4	15	24.6441	231.1743
AWC::caspase	0	2	4	16	169.2732	206.8474
AWC::caspase	0	3	1	1	72.7893	18.5476
AWC::caspase	0	3	1	2	51.3348	88.7222
AWC::caspase	0	3	1	3	103.0060	8.8009
AWC::caspase	0	3	1	4	91.5912	99.6423

AWC::caspase	0	3	1	5	125.2926	187.0505
AWC::caspase	0	3	1	6	148.3835	186.1350
AWC::caspase	0	3	1	7	104.6338	34.6378
AWC::caspase	0	3	1	8	72.0242	126.2931
AWC::caspase	0	3	2	1	40.4934	152.5185
AWC::caspase	0	3	2	2	123.4773	132.6190
AWC::caspase	0	3	2	3	74.3434	153.3958
AWC::caspase	0	3	2	4	91.6373	207.6550
AWC::caspase	0	3	2	5	69.8938	146.6255
AWC::caspase	0	3	2	6	34.5779	151.7952
AWC::caspase	0	3	2	7	99.0765	71.6638
AWC::caspase	0	3	2	8	145.3170	132.5291
AWC::caspase	0	3	2	9	144.4636	19.7722
AWC::caspase	0	3	2	10	119.2735	141.2825
AWC::caspase	0	3	2	11	62.3073	99.3039
AWC::caspase	0	3	2	12	117.8224	170.1331
AWC::caspase	0	3	2	13	93.1612	197.0891
AWC::caspase	0	3	2	14	82.0092	46.1043
AWC::caspase	0	3	2	15	68.1539	149.7675
AWC::caspase	0	3	2	16	124.5590	139.3706
AWC::caspase	0	3	2	17	38.9620	202.0920
AWC::caspase	0	3	2	18	71.8059	160.3490
AWC::caspase	0	3	3	1	41.6425	117.8328
AWC::caspase	0	3	3	2	104.4376	129.2971
AWC::caspase	0	3	3	3	85.2859	164.2649
AWC::caspase	0	3	3	4	43.2867	162.7549
AWC::caspase	0	3	3	5	94.3920	161.8692
AWC::caspase	0	3	3	6	57.2289	196.7068
AWC::caspase	0	3	3	7	92.5092	128.5229
AWC::caspase	0	3	3	8	147.5109	37.8533
AWC::caspase	0	3	3	9	83.6449	4.8937
AWC::caspase	0	3	3	10	75.3425	180.0084
AWC::caspase	0	3	3	11	158.4262	175.5780
AWC::caspase	0	3	3	12	78.0781	48.6995
AWC::caspase	0	3	3	13	95.5081	184.1627
AWC::caspase	0	3	3	14	63.4312	192.8234
AWC::caspase	0	3	3	15	74.9639	213.2426
AWC::caspase	0	3	3	16	57.2614	255.4531
AWC::caspase	0	3	3	17	71.8891	184.9167
AWC::caspase	0	3	3	18	99.2106	131.3622
AWC::caspase	0	3	3	19	151.8689	200.8904
AWC::caspase	0	3	3	20	97.9543	140.3738
AWC::caspase	0	3	3	21	104.0068	194.3605
AWC::caspase	0	3	3	22	47.1865	157.2140
AWC::caspase	0	3	3	23	20.3160	158.9594
AWC::caspase	0	3	3	24	76.1913	187.0734
AWC::caspase	0	3	3	25	56.9604	117.5946
AWC::caspase	0	3	3	26	56.2926	128.7815
AWC::caspase	0	3	3	27	88.9168	2.2516
AWC::caspase	0	3	4	1	26.1124	158.7611
AWC::caspase	0	3	4	2	52.8750	180.3648
AWC::caspase	0	3	4	3	109.0460	161.9733
AWC::caspase	0	3	4	4	133.3275	148.1524
AWC::caspase	0	3	4	5	76.0628	157.5181
AWC::caspase	0	3	4	6	92.7085	136.2329
AWC::caspase	0	3	4	7	91.9432	96.0198
AWC::caspase	0	3	4	8	31.4740	145.4920
AWC::caspase	0	3	4	9	20.6100	214.4765
AWC::caspase	0	3	4	10	58.7674	112.4113
AWC::caspase	0	3	4	11	100.6366	85.4135
AWC::caspase	0	3	4	12	95.1576	154.7494
AWC::caspase	0	3	4	13	72.9087	220.1728
AWC::caspase	0	3	4	14	108.5681	23.4459
AWC::caspase	0	3	4	15	83.2248	3.8798
AWC::caspase	0	3	4	16	109.3637	43.2081
AWC::caspase	0	3	4	17	89.4649	158.7929
AWC::caspase	0	3	4	18	91.4717	2.3074
AWC::caspase	0	3	4	19	73.4945	196.8326

AWC::caspase	0	3	4	20	151.8113	171.8654
AWC::caspase	0	3	4	21	89.0063	201.0092
AWC::caspase	0	3	4	22	27.2560	185.2598
AWC::caspase	0	3	4	23	135.6470	224.5007
AWC::caspase	0	3	4	24	31.7289	49.1451
AWC::caspase	0	3	4	25	61.3220	133.7392
AWC::caspase	0	3	4	26	28.0705	212.0767
AWC::caspase	0	3	4	27	67.5862	66.1114
AWC::caspase	0	3	4	28	157.5226	141.6927
AWC::caspase	0	3	4	29	144.2797	175.3725
AWC::caspase	0	3	4	30	88.7342	47.8392
AWC::caspase	0	3	4	31	128.0099	103.6942
AWC::caspase	1.5	1	1	1	22.0179	229.1040
AWC::caspase	1.5	1	1	2	26.8984	224.1658
AWC::caspase	1.5	1	1	3	36.7592	185.5627
AWC::caspase	1.5	1	1	4	27.2795	206.8907
AWC::caspase	1.5	1	1	5	103.2540	167.3773
AWC::caspase	1.5	1	1	6	22.6564	215.8683
AWC::caspase	1.5	1	1	7	87.5461	3.0892
AWC::caspase	1.5	1	1	8	41.9314	142.9911
AWC::caspase	1.5	1	1	9	75.8984	152.2559
AWC::caspase	1.5	1	1	10	28.7143	246.8109
AWC::caspase	1.5	1	2	1	8.2376	269.4043
AWC::caspase	1.5	1	2	2	37.8714	237.0862
AWC::caspase	1.5	1	2	3	10.1538	240.1520
AWC::caspase	1.5	1	2	4	11.7161	255.6419
AWC::caspase	1.5	1	2	5	33.7146	200.7474
AWC::caspase	1.5	1	2	6	9.1771	232.8041
AWC::caspase	1.5	1	2	7	18.2258	247.2654
AWC::caspase	1.5	1	2	8	11.9000	225.5300
AWC::caspase	1.5	1	2	9	13.8508	210.0079
AWC::caspase	1.5	1	2	10	11.8651	196.2804
AWC::caspase	1.5	1	2	11	50.5609	98.6761
AWC::caspase	1.5	1	2	12	61.7207	183.1771
AWC::caspase	1.5	1	2	13	16.5938	228.8435
AWC::caspase	1.5	1	2	14	8.3163	263.7429
AWC::caspase	1.5	1	2	15	140.8265	6.0900
AWC::caspase	1.5	1	2	16	11.9300	200.2563
AWC::caspase	1.5	1	2	17	21.8942	257.8439
AWC::caspase	1.5	1	2	18	16.4377	120.8475
AWC::caspase	1.5	1	2	19	11.1417	232.4574
AWC::caspase	1.5	1	3	1	13.0463	213.8560
AWC::caspase	1.5	1	3	2	10.2819	218.7443
AWC::caspase	1.5	1	3	3	18.6081	230.2097
AWC::caspase	1.5	1	3	4	10.4993	236.2467
AWC::caspase	1.5	1	3	5	5.3743	266.7052
AWC::caspase	1.5	1	3	6	35.8486	204.9765
AWC::caspase	1.5	1	3	7	8.6802	173.0007
AWC::caspase	1.5	1	3	8	17.1320	240.8249
AWC::caspase	1.5	1	3	9	38.7113	283.8856
AWC::caspase	1.5	1	3	10	30.3232	178.9430
AWC::caspase	1.5	1	3	11	84.8506	112.3186
AWC::caspase	1.5	1	3	12	13.1791	203.1592
AWC::caspase	1.5	1	3	13	25.4483	164.2853
AWC::caspase	1.5	1	3	14	10.2930	171.1897
AWC::caspase	1.5	1	3	15	10.2620	210.7008
AWC::caspase	1.5	1	3	16	12.8284	213.1967
AWC::caspase	1.5	1	3	17	48.1156	86.7589
AWC::caspase	1.5	1	3	18	6.7768	149.2983
AWC::caspase	1.5	1	3	19	34.8169	98.4680
AWC::caspase	1.5	1	3	20	17.3076	182.4267
AWC::caspase	1.5	1	3	21	43.3565	206.8792
AWC::caspase	1.5	1	3	22	35.6758	118.7366
AWC::caspase	1.5	1	3	23	20.7252	175.4959
AWC::caspase	1.5	1	3	24	20.5982	243.0167
AWC::caspase	1.5	1	3	25	13.7282	289.6557
AWC::caspase	1.5	1	3	26	31.9430	160.7695
AWC::caspase	1.5	1	3	27	33.0725	226.7251

AWC::caspase	1.5	1	3	28	7.6494	202.5227
AWC::caspase	1.5	1	3	29	17.5085	141.1858
AWC::caspase	1.5	1	4	1	15.7966	230.3758
AWC::caspase	1.5	1	4	2	31.4789	188.5920
AWC::caspase	1.5	1	4	3	94.1023	4.8007
AWC::caspase	1.5	1	4	4	17.4106	194.3452
AWC::caspase	1.5	1	4	5	30.9686	112.0525
AWC::caspase	1.5	1	4	6	34.5309	251.0103
AWC::caspase	1.5	1	4	7	6.9967	225.7610
AWC::caspase	1.5	1	4	8	13.2899	170.0139
AWC::caspase	1.5	1	4	9	21.8198	186.3176
AWC::caspase	1.5	1	4	10	32.7558	163.9542
AWC::caspase	1.5	1	4	11	41.0343	130.3131
AWC::caspase	1.5	1	4	12	50.2984	142.3318
AWC::caspase	1.5	1	4	13	7.8588	177.6101
AWC::caspase	1.5	1	4	14	8.7580	158.7425
AWC::caspase	1.5	1	4	15	7.5606	242.1102
AWC::caspase	1.5	1	4	16	62.0971	138.4278
AWC::caspase	1.5	1	4	17	33.3999	67.0543
AWC::caspase	1.5	1	4	18	40.8532	84.3535
AWC::caspase	1.5	1	4	19	44.5779	142.5625
AWC::caspase	1.5	1	4	20	18.2814	102.8521
AWC::caspase	1.5	1	4	21	22.2519	148.3297
AWC::caspase	1.5	1	4	22	23.6250	96.2890
AWC::caspase	1.5	1	4	23	42.7640	194.6199
AWC::caspase	1.5	1	4	24	54.5692	79.6651
AWC::caspase	1.5	2	1	1	15.9946	225.5600
AWC::caspase	1.5	2	1	2	11.6155	253.9252
AWC::caspase	1.5	2	1	3	28.5526	257.1890
AWC::caspase	1.5	2	1	4	19.3992	275.6507
AWC::caspase	1.5	2	1	5	45.6866	152.8810
AWC::caspase	1.5	2	1	6	48.4034	194.7744
AWC::caspase	1.5	2	1	7	27.3911	274.4031
AWC::caspase	1.5	2	1	8	24.4232	297.5315
AWC::caspase	1.5	2	1	9	59.1815	229.7519
AWC::caspase	1.5	2	1	10	33.8827	202.3973
AWC::caspase	1.5	2	1	11	26.1056	273.2051
AWC::caspase	1.5	2	1	12	19.6998	256.9153
AWC::caspase	1.5	2	1	13	10.9372	264.6092
AWC::caspase	1.5	2	1	14	25.6861	228.7995
AWC::caspase	1.5	2	1	15	14.5877	254.8729
AWC::caspase	1.5	2	1	16	14.6285	203.7736
AWC::caspase	1.5	2	1	17	23.1976	267.2859
AWC::caspase	1.5	2	1	18	15.5720	225.6028
AWC::caspase	1.5	2	1	19	33.6702	181.2667
AWC::caspase	1.5	2	1	20	12.1551	246.3493
AWC::caspase	1.5	2	1	21	27.3541	295.9072
AWC::caspase	1.5	2	2	1	46.6595	139.2192
AWC::caspase	1.5	2	2	2	17.2123	240.6946
AWC::caspase	1.5	2	2	3	20.4610	233.6611
AWC::caspase	1.5	2	2	4	20.4556	210.5795
AWC::caspase	1.5	2	2	5	16.3715	201.9837
AWC::caspase	1.5	2	2	6	22.5782	197.1899
AWC::caspase	1.5	2	2	7	15.6772	249.8707
AWC::caspase	1.5	2	2	8	21.1982	292.7659
AWC::caspase	1.5	2	2	9	27.1293	164.1958
AWC::caspase	1.5	2	2	10	11.3619	195.6988
AWC::caspase	1.5	2	2	11	18.1844	223.8702
AWC::caspase	1.5	2	2	12	46.2304	146.2163
AWC::caspase	1.5	2	2	13	17.5046	191.3036
AWC::caspase	1.5	2	2	14	77.3474	69.8551
AWC::caspase	1.5	2	2	15	18.6815	294.8342
AWC::caspase	1.5	2	2	16	44.7642	172.6963
AWC::caspase	1.5	2	2	17	35.0883	131.7890
AWC::caspase	1.5	2	2	18	19.3798	254.7455
AWC::caspase	1.5	2	2	19	7.3047	219.8066
AWC::caspase	1.5	2	2	20	92.2058	26.2595
AWC::caspase	1.5	2	2	21	17.3801	229.9522

AWC::caspase	1.5	2	2	22	10.9093	224.4329
AWC::caspase	1.5	2	2	23	5.4057	265.1133
AWC::caspase	1.5	2	2	24	7.4978	262.4297
AWC::caspase	1.5	2	2	25	15.5676	263.1792
AWC::caspase	1.5	2	2	26	29.1356	195.8226
AWC::caspase	1.5	2	2	27	20.4293	278.1931
AWC::caspase	1.5	2	2	28	9.2763	182.1379
AWC::caspase	1.5	2	3	1	19.1843	255.0323
AWC::caspase	1.5	2	3	2	21.8987	186.8148
AWC::caspase	1.5	2	3	3	11.6311	236.8967
AWC::caspase	1.5	2	3	4	11.4011	174.2547
AWC::caspase	1.5	2	3	5	6.2103	168.1499
AWC::caspase	1.5	2	3	6	34.9252	226.7920
AWC::caspase	1.5	2	3	7	16.1348	202.8960
AWC::caspase	1.5	2	3	8	13.7043	304.4343
AWC::caspase	1.5	2	3	9	10.8041	305.7564
AWC::caspase	1.5	2	3	10	36.5946	186.0258
AWC::caspase	1.5	2	3	11	9.8116	246.5664
AWC::caspase	1.5	2	3	12	47.1452	144.1306
AWC::caspase	1.5	2	3	13	11.7890	187.7519
AWC::caspase	1.5	2	3	14	34.1053	198.7729
AWC::caspase	1.5	2	3	15	43.1700	154.5670
AWC::caspase	1.5	2	3	16	18.8035	244.1793
AWC::caspase	1.5	2	3	17	11.5221	228.6255
AWC::caspase	1.5	2	3	18	11.6190	172.4473
AWC::caspase	1.5	2	3	19	14.2593	185.5765
AWC::caspase	1.5	2	3	20	18.5013	213.8406
AWC::caspase	1.5	2	3	21	21.3254	205.2098
AWC::caspase	1.5	2	3	22	7.3805	160.2196
AWC::caspase	1.5	2	3	23	10.6952	161.2896
AWC::caspase	1.5	2	3	24	25.7702	88.6946
AWC::caspase	1.5	2	3	25	13.2447	199.1578
AWC::caspase	1.5	2	3	26	19.4181	200.1086
AWC::caspase	1.5	2	4	1	16.0180	272.8646
AWC::caspase	1.5	2	4	2	9.5237	230.4252
AWC::caspase	1.5	2	4	3	31.5069	214.0589
AWC::caspase	1.5	2	4	4	15.8196	185.5004
AWC::caspase	1.5	2	4	5	18.8029	200.8452
AWC::caspase	1.5	2	4	6	16.8871	240.4828
AWC::caspase	1.5	2	4	7	37.5894	222.2110
AWC::caspase	1.5	2	4	8	41.2336	212.8123
AWC::caspase	1.5	2	4	9	36.6523	169.4683
AWC::caspase	1.5	2	4	10	32.7390	205.3898
AWC::caspase	1.5	2	4	11	55.9275	113.2010
AWC::caspase	1.5	2	4	12	40.2679	173.2185
AWC::caspase	1.5	2	4	13	22.7802	202.0863
AWC::caspase	1.5	2	4	14	33.6419	212.1052
AWC::caspase	1.5	2	4	15	12.1184	170.2757
AWC::caspase	1.5	3	4	16	25.9877	209.4808
AWC::caspase	1.5	3	1	1	49.3723	118.9328
AWC::caspase	1.5	3	1	2	21.9870	147.8701
AWC::caspase	1.5	3	1	3	9.0279	214.0042
AWC::caspase	1.5	3	1	4	27.9369	203.8450
AWC::caspase	1.5	3	1	5	10.1748	306.7829
AWC::caspase	1.5	3	1	6	11.0885	215.7503
AWC::caspase	1.5	3	1	7	22.2120	134.7242
AWC::caspase	1.5	3	1	8	30.8298	231.6866
AWC::caspase	1.5	3	1	9	13.1904	265.0242
AWC::caspase	1.5	3	1	10	43.8736	172.8457
AWC::caspase	1.5	3	2	1	18.6620	256.4142
AWC::caspase	1.5	3	2	2	26.6228	265.4459
AWC::caspase	1.5	3	2	3	22.1256	232.5471
AWC::caspase	1.5	3	2	4	24.6719	220.8110
AWC::caspase	1.5	3	2	5	19.7603	222.1311
AWC::caspase	1.5	3	2	6	14.2465	218.0519
AWC::caspase	1.5	3	2	7	7.7977	161.7033
AWC::caspase	1.5	3	2	8	11.2242	170.0579
AWC::caspase	1.5	3	2	9	27.4833	124.5758

AWC::caspase	1.5	3	2	10	21.3638	262.2538
AWC::caspase	1.5	3	2	11	11.0334	249.8544
AWC::caspase	1.5	3	2	12	21.6178	237.8825
AWC::caspase	1.5	3	2	13	25.2711	239.3005
AWC::caspase	1.5	3	2	14	19.9526	179.4159
AWC::caspase	1.5	3	2	15	94.9735	28.1254
AWC::caspase	1.5	3	2	16	11.7010	213.9921
AWC::caspase	1.5	3	2	17	15.0041	215.5863
AWC::caspase	1.5	3	2	18	8.2886	235.5372
AWC::caspase	1.5	3	2	19	14.4756	173.8392
AWC::caspase	1.5	3	2	20	19.4829	204.5247
AWC::caspase	1.5	3	3	1	29.9081	227.0572
AWC::caspase	1.5	3	3	2	14.6717	234.3079
AWC::caspase	1.5	3	3	3	36.2899	251.2289
AWC::caspase	1.5	3	3	4	15.8807	234.2605
AWC::caspase	1.5	3	3	5	21.9549	171.5123
AWC::caspase	1.5	3	3	6	24.6741	193.5890
AWC::caspase	1.5	3	3	7	16.4863	229.0696
AWC::caspase	1.5	3	3	8	9.0617	244.0855
AWC::caspase	1.5	3	3	9	23.7438	198.0974
AWC::caspase	1.5	3	3	10	12.3463	200.5650
AWC::caspase	1.5	3	3	11	20.1735	148.7067
AWC::caspase	1.5	3	3	12	64.0261	69.3042
AWC::caspase	1.5	3	3	13	22.0054	264.6634
AWC::caspase	1.5	3	3	14	9.7818	154.0970
AWC::caspase	1.5	3	3	15	8.4650	299.3306
AWC::caspase	1.5	3	3	16	34.5106	165.3267
AWC::caspase	1.5	3	3	17	20.7898	183.7042
AWC::caspase	1.5	3	3	18	6.0795	164.4576
AWC::caspase	1.5	3	3	19	12.8802	231.4379
AWC::caspase	1.5	3	3	20	4.4298	155.5262
AWC::caspase	1.5	3	3	21	20.5075	244.7250
AWC::caspase	1.5	3	3	22	8.3044	215.8701
AWC::caspase	1.5	3	3	23	26.7861	215.0899
AWC::caspase	1.5	3	3	24	14.3176	128.4933
AWC::caspase	1.5	3	4	1	44.4459	206.7335
AWC::caspase	1.5	3	4	2	30.4493	175.0719
AWC::caspase	1.5	3	4	3	10.5030	244.2977
AWC::caspase	1.5	3	4	4	14.1370	162.6033
AWC::caspase	1.5	3	4	5	36.5218	219.3374
AWC::caspase	1.5	3	4	6	17.1290	235.4768
AWC::caspase	1.5	3	4	7	21.2218	231.7352
AWC::caspase	1.5	3	4	8	6.6185	204.9817
AWC::caspase	1.5	3	4	9	25.8269	246.0394
AWC::caspase	1.5	3	4	10	17.4389	269.6917
AWC::caspase	1.5	3	4	11	24.3254	271.5785
AWC::caspase	1.5	3	4	12	56.6719	188.1914
AWC::caspase	1.5	3	4	13	43.2418	126.2182
AWC::caspase	1.5	3	4	14	38.5257	213.2556
AWC::caspase	1.5	3	4	15	29.8557	175.2304
AWC::caspase	1.5	3	4	16	17.1012	178.2242
AWC::caspase	1.5	3	4	17	8.3605	215.5981
AWC::caspase	1.5	3	4	18	32.5865	206.6695
AWC::caspase	1.5	3	4	19	13.7013	132.2263
AWC::caspase	1.5	3	4	20	6.4299	233.2834
AWC::caspase	1.5	3	4	21	28.6201	192.3195
AWC::caspase	1.5	3	4	22	8.7280	192.7081
AWC::caspase	1.5	3	4	23	7.0315	240.7710
AWC::caspase	1.5	3	4	24	28.2810	199.0349
AWC::caspase	3	1	1	1	21.8191	180.7408
AWC::caspase	3	1	1	2	21.7173	225.5349
AWC::caspase	3	1	1	3	36.3737	240.3080
AWC::caspase	3	1	1	4	99.8505	5.2889
AWC::caspase	3	1	1	5	16.8638	248.9197
AWC::caspase	3	1	1	6	40.6288	225.0637
AWC::caspase	3	1	1	7	9.2779	270.7813
AWC::caspase	3	1	1	8	15.6855	227.6014
AWC::caspase	3	1	1	9	15.6580	242.7576

AWC::caspase	3	1	1	10	29.3169	283.7152
AWC::caspase	3	1	2	1	24.2619	271.1775
AWC::caspase	3	1	2	2	29.4970	224.0446
AWC::caspase	3	1	2	3	24.6549	224.5191
AWC::caspase	3	1	2	4	15.6265	290.5561
AWC::caspase	3	1	2	5	44.0096	294.2360
AWC::caspase	3	1	2	6	23.9339	262.0217
AWC::caspase	3	1	2	7	15.3655	248.4242
AWC::caspase	3	1	2	8	22.2789	225.2225
AWC::caspase	3	1	2	9	15.9145	218.1819
AWC::caspase	3	1	2	10	19.5438	245.4422
AWC::caspase	3	1	2	11	11.0198	275.2529
AWC::caspase	3	1	2	12	19.5614	256.2597
AWC::caspase	3	1	2	13	91.9982	3.7939
AWC::caspase	3	1	2	14	94.5267	3.0410
AWC::caspase	3	1	2	15	92.8965	3.8887
AWC::caspase	3	1	2	16	81.1914	3.7978
AWC::caspase	3	1	2	17	53.1871	179.8282
AWC::caspase	3	1	2	18	39.4180	240.8121
AWC::caspase	3	1	2	19	29.4696	152.3398
AWC::caspase	3	1	2	20	14.5866	249.8664
AWC::caspase	3	1	2	21	10.1924	247.9711
AWC::caspase	3	1	2	22	39.0835	55.8883
AWC::caspase	3	1	3	1	40.2911	219.2882
AWC::caspase	3	1	3	2	12.1522	247.9309
AWC::caspase	3	1	3	3	38.0347	213.2192
AWC::caspase	3	1	3	4	8.7332	220.5167
AWC::caspase	3	1	3	5	19.5726	229.5679
AWC::caspase	3	1	3	6	22.4471	276.0938
AWC::caspase	3	1	3	7	10.9290	239.1193
AWC::caspase	3	1	3	8	38.1690	258.6937
AWC::caspase	3	1	3	9	35.2163	131.9669
AWC::caspase	3	1	3	10	39.2865	242.0324
AWC::caspase	3	1	3	11	37.5804	160.3292
AWC::caspase	3	1	3	12	37.4545	199.0047
AWC::caspase	3	1	3	13	16.1203	242.4176
AWC::caspase	3	1	3	14	36.2709	265.0979
AWC::caspase	3	1	3	15	47.5980	234.6950
AWC::caspase	3	1	4	1	47.3057	173.9478
AWC::caspase	3	1	4	2	42.0009	191.2695
AWC::caspase	3	1	4	3	41.3692	120.7208
AWC::caspase	3	1	4	4	11.1591	199.4641
AWC::caspase	3	1	4	5	62.7699	145.2821
AWC::caspase	3	1	4	6	20.0687	108.4768
AWC::caspase	3	1	4	7	96.2509	209.3652
AWC::caspase	3	2	1	1	40.2398	124.4121
AWC::caspase	3	2	1	2	30.0216	169.6790
AWC::caspase	3	2	1	3	75.8977	79.8807
AWC::caspase	3	2	1	4	30.8982	231.7927
AWC::caspase	3	2	1	5	37.9020	214.9057
AWC::caspase	3	2	1	6	28.5112	216.2074
AWC::caspase	3	2	1	7	106.1465	6.7787
AWC::caspase	3	2	1	8	40.6120	195.7727
AWC::caspase	3	2	1	9	26.5669	231.5654
AWC::caspase	3	2	1	10	15.1812	194.9155
AWC::caspase	3	2	1	11	30.0510	234.3781
AWC::caspase	3	2	1	12	18.9294	211.7709
AWC::caspase	3	2	2	1	31.9564	219.8718
AWC::caspase	3	2	2	2	32.9469	178.3529
AWC::caspase	3	2	2	3	32.7286	231.8947
AWC::caspase	3	2	2	4	18.5555	221.5285
AWC::caspase	3	2	2	5	21.7431	129.4870
AWC::caspase	3	2	2	6	11.1578	215.4047
AWC::caspase	3	2	2	7	23.6318	281.4362
AWC::caspase	3	2	2	8	17.4213	260.3312
AWC::caspase	3	2	2	9	18.2762	217.2222
AWC::caspase	3	2	2	10	26.5087	209.8688
AWC::caspase	3	2	2	11	33.3539	169.0860

AWC::caspase	3	2	2	12	31.0407	196.4807
AWC::caspase	3	2	2	13	49.7529	105.7210
AWC::caspase	3	2	2	14	24.0023	211.8967
AWC::caspase	3	2	2	15	98.7249	4.7665
AWC::caspase	3	2	2	16	31.8010	197.0023
AWC::caspase	3	2	2	17	26.5382	230.1192
AWC::caspase	3	2	2	18	14.9566	207.5108
AWC::caspase	3	2	2	19	37.4518	225.5015
AWC::caspase	3	2	3	1	40.2531	165.4884
AWC::caspase	3	2	3	2	38.0174	215.2842
AWC::caspase	3	2	3	3	21.6447	195.4248
AWC::caspase	3	2	3	4	14.6780	261.8932
AWC::caspase	3	2	3	5	24.4861	129.1449
AWC::caspase	3	2	3	6	14.5352	284.9300
AWC::caspase	3	2	3	7	14.4179	211.9754
AWC::caspase	3	2	3	8	42.6914	197.3811
AWC::caspase	3	2	3	9	21.3780	243.9758
AWC::caspase	3	2	3	10	38.3389	147.6597
AWC::caspase	3	2	3	11	34.2141	180.0449
AWC::caspase	3	2	3	12	79.5013	108.1027
AWC::caspase	3	2	3	13	18.8853	264.0651
AWC::caspase	3	2	3	14	22.0844	243.8036
AWC::caspase	3	2	3	15	26.5746	192.9414
AWC::caspase	3	2	3	16	73.3067	111.4728
AWC::caspase	3	2	3	17	90.3333	3.8398
AWC::caspase	3	2	3	18	70.8114	25.7203
AWC::caspase	3	2	3	19	24.6634	95.0778
AWC::caspase	3	2	3	20	82.5593	6.4170
AWC::caspase	3	2	4	1	15.9176	245.7370
AWC::caspase	3	2	4	2	33.4314	198.6681
AWC::caspase	3	2	4	3	24.9742	209.7320
AWC::caspase	3	2	4	4	9.1870	246.9898
AWC::caspase	3	2	4	5	42.8900	136.3960
AWC::caspase	3	2	4	6	27.2997	169.7491
AWC::caspase	3	2	4	7	32.6878	211.0359
AWC::caspase	3	2	4	8	45.1070	162.6762
AWC::caspase	3	2	4	9	92.9919	20.6514
AWC::caspase	3	2	4	10	36.4138	254.7751
AWC::caspase	3	2	4	11	44.7908	152.4238
AWC::caspase	3	2	4	12	33.4112	150.8744
AWC::caspase	3	2	4	13	29.3201	139.1987
AWC::caspase	3	2	4	14	50.4132	110.3973
AWC::caspase	3	2	4	15	28.4365	241.0244
AWC::caspase	3	3	1	1	32.6929	221.7110
AWC::caspase	3	3	1	2	28.6666	220.5670
AWC::caspase	3	3	1	3	29.0817	252.8357
AWC::caspase	3	3	1	4	40.0248	216.0085
AWC::caspase	3	3	1	5	32.6551	145.4501
AWC::caspase	3	3	1	6	35.6106	248.1618
AWC::caspase	3	3	1	7	43.0117	228.2011
AWC::caspase	3	3	1	8	25.3447	266.0854
AWC::caspase	3	3	1	9	40.1565	224.2716
AWC::caspase	3	3	1	10	39.2559	216.5913
AWC::caspase	3	3	1	11	25.5256	234.8378
AWC::caspase	3	3	1	12	26.2084	226.6503
AWC::caspase	3	3	1	13	27.6575	237.1073
AWC::caspase	3	3	1	14	16.2995	263.3995
AWC::caspase	3	3	1	15	11.6605	236.9030
AWC::caspase	3	3	1	16	35.7872	217.8877
AWC::caspase	3	3	1	17	34.5352	258.1636
AWC::caspase	3	3	2	1	34.2121	224.5285
AWC::caspase	3	3	2	2	26.7853	247.4587
AWC::caspase	3	3	2	3	30.6890	227.5289
AWC::caspase	3	3	2	4	24.9084	276.4195
AWC::caspase	3	3	2	5	9.4397	243.4818
AWC::caspase	3	3	2	6	31.2713	257.7510
AWC::caspase	3	3	2	7	25.7565	258.8490
AWC::caspase	3	3	2	8	32.5116	220.2927

AWC::caspase	3	3	2	9	35.0193	246.4069
AWC::caspase	3	3	2	10	37.2446	198.4894
AWC::caspase	3	3	2	11	11.1118	239.3472
AWC::caspase	3	3	2	12	37.4578	203.3236
AWC::caspase	3	3	2	13	21.9499	272.2119
AWC::caspase	3	3	3	1	27.5905	270.1406
AWC::caspase	3	3	3	2	56.3683	191.3057
AWC::caspase	3	3	3	3	46.4258	188.4268
AWC::caspase	3	3	3	4	34.8073	230.8372
AWC::caspase	3	3	3	5	30.2889	287.5580
AWC::caspase	3	3	3	6	22.1767	248.9513
AWC::caspase	3	3	3	7	30.1390	286.7168
AWC::caspase	3	3	3	8	20.2155	241.0570
AWC::caspase	3	3	3	9	40.3168	135.7808
AWC::caspase	3	3	3	10	28.6974	262.5534
AWC::caspase	3	3	3	11	9.5153	224.4149
AWC::caspase	3	3	3	12	41.7620	236.3791
AWC::caspase	3	3	3	13	29.7555	169.1289
AWC::caspase	3	3	3	14	31.2364	280.2126
AWC::caspase	3	3	3	15	12.2515	229.0655
AWC::caspase	3	3	3	16	60.7184	111.1798
AWC::caspase	3	3	3	17	13.1095	247.6672
AWC::caspase	3	3	4	1	26.0662	208.6091
AWC::caspase	3	3	4	2	7.3409	247.4578
AWC::caspase	3	3	4	3	15.3907	295.5401
AWC::caspase	3	3	4	4	30.2764	216.1420
AWC::caspase	3	3	4	5	24.2402	248.6057
AWC::caspase	3	3	4	6	21.5589	237.2911
AWC::caspase	3	3	4	7	44.8806	219.5990
AWC::caspase	3	3	4	8	52.0010	162.4363
AWC::caspase	3	3	4	9	79.3420	84.9693
AWC::caspase	3	3	4	10	47.8427	146.0468
AWC::caspase	3	3	4	11	11.8434	285.7877
AWC::caspase	3	3	4	12	8.8084	266.2905
AWC::caspase	3	3	4	13	36.9728	268.0791
AWC::caspase	6	1	1	1	33.0217	253.3050
AWC::caspase	6	1	1	2	31.3269	173.5319
AWC::caspase	6	1	1	3	42.1763	209.9019
AWC::caspase	6	1	1	4	46.8150	168.5278
AWC::caspase	6	1	1	5	59.9642	72.0719
AWC::caspase	6	1	1	6	111.6054	4.9380
AWC::caspase	6	1	1	7	26.1353	214.0625
AWC::caspase	6	1	1	8	32.2373	207.9127
AWC::caspase	6	1	2	1	21.0879	225.1907
AWC::caspase	6	1	2	2	30.9708	220.6779
AWC::caspase	6	1	2	3	40.7096	255.9674
AWC::caspase	6	1	2	4	26.2350	229.8933
AWC::caspase	6	1	2	5	40.6489	273.3757
AWC::caspase	6	1	2	6	33.7591	224.7802
AWC::caspase	6	1	2	7	37.7842	209.0183
AWC::caspase	6	1	2	8	41.8671	221.2616
AWC::caspase	6	1	2	9	31.6148	230.7283
AWC::caspase	6	1	2	10	15.2861	241.0738
AWC::caspase	6	1	2	11	83.3301	113.5061
AWC::caspase	6	1	2	12	19.5321	233.7045
AWC::caspase	6	1	2	13	22.0538	184.2681
AWC::caspase	6	1	2	14	41.6243	248.4912
AWC::caspase	6	1	3	1	61.3921	287.2237
AWC::caspase	6	1	3	2	39.0731	191.7142
AWC::caspase	6	1	3	3	19.9932	248.8195
AWC::caspase	6	1	3	4	48.5038	124.2512
AWC::caspase	6	1	3	5	32.4687	160.8383
AWC::caspase	6	1	3	6	29.0540	232.3005
AWC::caspase	6	1	3	7	38.5755	217.6907
AWC::caspase	6	1	3	8	29.9220	199.5698
AWC::caspase	6	1	3	9	41.6388	253.1336
AWC::caspase	6	1	3	10	34.2048	241.6640
AWC::caspase	6	1	3	11	99.3445	20.1752

AWC::caspase	6	1	3	12	28.5400	161.1853
AWC::caspase	6	1	3	13	75.4010	100.5421
AWC::caspase	6	1	3	14	25.6155	250.0821
AWC::caspase	6	1	3	15	38.8558	182.3788
AWC::caspase	6	1	3	16	43.5193	181.4132
AWC::caspase	6	1	3	17	44.6156	222.2600
AWC::caspase	6	1	3	18	14.6049	196.8242
AWC::caspase	6	1	3	19	33.6856	234.7481
AWC::caspase	6	1	3	20	36.9741	202.3201
AWC::caspase	6	1	3	21	49.9266	206.8426
AWC::caspase	6	1	4	1	58.7527	153.9497
AWC::caspase	6	1	4	2	53.5770	159.7118
AWC::caspase	6	1	4	3	42.8064	205.1149
AWC::caspase	6	1	4	4	18.8391	201.5182
AWC::caspase	6	1	4	5	11.7870	171.6060
AWC::caspase	6	1	4	6	51.0634	199.7735
AWC::caspase	6	1	4	7	19.2677	164.5861
AWC::caspase	6	1	4	8	27.9675	244.9473
AWC::caspase	6	1	4	9	27.2932	204.8068
AWC::caspase	6	1	4	10	24.1694	129.9679
AWC::caspase	6	1	4	11	39.1510	223.9911
AWC::caspase	6	1	4	12	71.0269	147.4205
AWC::caspase	6	1	4	13	34.6311	208.0863
AWC::caspase	6	1	4	14	70.8514	139.1888
AWC::caspase	6	1	4	15	60.7099	165.6853
AWC::caspase	6	1	4	16	95.5989	24.8729
AWC::caspase	6	1	4	17	49.1724	191.6798
AWC::caspase	6	1	4	18	49.6262	110.4682
AWC::caspase	6	1	4	19	61.7442	211.9848
AWC::caspase	6	1	4	20	38.5989	248.6888
AWC::caspase	6	1	4	21	66.9124	105.7658
AWC::caspase	6	1	4	22	48.5637	252.3121
AWC::caspase	6	1	4	23	67.5894	246.9220
AWC::caspase	6	1	4	24	25.2002	196.9887
AWC::caspase	6	1	4	25	136.2197	120.5540
AWC::caspase	6	1	4	26	40.2835	247.6643
AWC::caspase	6	1	4	27	28.0644	191.6940
AWC::caspase	6	2	1	1	61.9073	236.7463
AWC::caspase	6	2	1	2	38.4306	216.9295
AWC::caspase	6	2	1	3	35.2254	182.2400
AWC::caspase	6	2	1	4	51.7126	204.0710
AWC::caspase	6	2	1	5	25.4321	189.2689
AWC::caspase	6	2	1	6	25.3224	215.5322
AWC::caspase	6	2	1	7	51.8761	159.5786
AWC::caspase	6	2	1	8	49.7416	250.9146
AWC::caspase	6	2	1	9	47.3536	173.7918
AWC::caspase	6	2	1	10	50.7420	217.2314
AWC::caspase	6	2	1	11	14.2478	221.5365
AWC::caspase	6	2	1	12	42.4926	214.0221
AWC::caspase	6	2	1	13	51.4659	175.4195
AWC::caspase	6	2	1	14	67.0595	231.0000
AWC::caspase	6	2	1	15	54.8262	249.4555
AWC::caspase	6	2	1	16	54.2283	226.9909
AWC::caspase	6	2	1	17	50.1856	266.9883
AWC::caspase	6	2	1	18	59.1245	175.4974
AWC::caspase	6	2	2	1	41.3632	257.6206
AWC::caspase	6	2	2	2	43.1316	224.1708
AWC::caspase	6	2	2	3	29.5905	197.9501
AWC::caspase	6	2	2	4	17.6455	154.9451
AWC::caspase	6	2	2	5	11.8132	116.7059
AWC::caspase	6	2	2	6	26.7956	210.5599
AWC::caspase	6	2	2	7	34.5037	202.2387
AWC::caspase	6	2	2	8	30.4266	196.4669
AWC::caspase	6	2	2	9	25.9806	233.2759
AWC::caspase	6	2	2	10	40.2417	251.3352
AWC::caspase	6	2	2	11	24.7567	181.5405
AWC::caspase	6	2	2	12	35.9016	199.8401
AWC::caspase	6	2	2	13	34.2855	281.8575

AWC::caspase	6	2	2	14	49.7442	246.2794
AWC::caspase	6	2	2	15	22.0704	249.2490
AWC::caspase	6	2	2	16	17.7376	235.9740
AWC::caspase	6	2	2	17	7.2552	191.7838
AWC::caspase	6	2	2	18	17.7983	155.0484
AWC::caspase	6	2	2	19	49.3348	193.1910
AWC::caspase	6	2	2	20	15.8205	142.6969
AWC::caspase	6	2	2	21	22.3463	165.9628
AWC::caspase	6	2	2	22	44.8426	240.6437
AWC::caspase	6	2	2	23	48.7164	232.9602
AWC::caspase	6	2	2	24	5.1110	156.3021
AWC::caspase	6	2	3	1	46.7156	224.6425
AWC::caspase	6	2	3	2	28.6164	262.8752
AWC::caspase	6	2	3	3	19.1669	191.6039
AWC::caspase	6	2	3	4	33.9037	268.3057
AWC::caspase	6	2	3	5	44.3475	162.7066
AWC::caspase	6	2	3	6	27.4651	247.4973
AWC::caspase	6	2	3	7	27.0603	203.4967
AWC::caspase	6	2	3	8	43.8058	251.7485
AWC::caspase	6	2	3	9	43.9675	166.2134
AWC::caspase	6	2	3	10	27.5779	216.0440
AWC::caspase	6	2	3	11	32.9461	143.4173
AWC::caspase	6	2	3	12	35.2359	254.8891
AWC::caspase	6	2	3	13	46.7078	187.7833
AWC::caspase	6	2	3	14	33.1969	149.2929
AWC::caspase	6	2	3	15	28.4305	193.1780
AWC::caspase	6	2	3	16	40.9064	190.3291
AWC::caspase	6	2	3	17	62.5434	209.2404
AWC::caspase	6	2	3	18	98.3232	8.9610
AWC::caspase	6	2	3	19	28.2605	231.7337
AWC::caspase	6	2	3	20	44.1574	141.4509
AWC::caspase	6	2	3	21	37.7934	260.5744
AWC::caspase	6	2	3	22	28.7113	207.5281
AWC::caspase	6	2	3	23	39.8290	140.4410
AWC::caspase	6	2	3	24	46.4462	208.7759
AWC::caspase	6	2	3	25	37.6412	176.9478
AWC::caspase	6	2	3	26	43.3015	121.2128
AWC::caspase	6	2	3	27	41.2273	142.6529
AWC::caspase	6	2	3	28	31.6629	180.2414
AWC::caspase	6	2	4	1	41.5848	234.5662
AWC::caspase	6	2	4	2	34.0605	187.7493
AWC::caspase	6	2	4	3	36.8192	214.9663
AWC::caspase	6	2	4	4	26.7427	136.0177
AWC::caspase	6	2	4	5	45.1812	171.4184
AWC::caspase	6	2	4	6	55.0064	189.0484
AWC::caspase	6	2	4	7	53.4347	167.6733
AWC::caspase	6	2	4	8	39.1043	172.7076
AWC::caspase	6	2	4	9	44.0924	276.8298
AWC::caspase	6	2	4	10	22.0986	193.6331
AWC::caspase	6	2	4	11	19.8475	203.3957
AWC::caspase	6	2	4	12	39.5114	158.9383
AWC::caspase	6	2	4	13	44.6432	137.4896
AWC::caspase	6	2	4	14	10.3530	193.8942
AWC::caspase	6	2	4	15	25.2354	193.8810
AWC::caspase	6	2	4	16	54.7050	173.2009
AWC::caspase	6	2	4	17	46.8049	314.5165
AWC::caspase	6	3	1	1	72.1817	172.6438
AWC::caspase	6	3	1	2	65.5286	190.2617
AWC::caspase	6	3	1	3	60.8580	198.0542
AWC::caspase	6	3	1	4	24.3280	169.6133
AWC::caspase	6	3	1	5	16.7551	142.1424
AWC::caspase	6	3	1	6	59.0137	192.4654
AWC::caspase	6	3	1	7	22.4890	181.0331
AWC::caspase	6	3	1	8	32.0632	156.7195
AWC::caspase	6	3	1	9	67.4518	140.1151
AWC::caspase	6	3	1	10	36.7344	171.4378
AWC::caspase	6	3	2	1	14.5982	157.8534
AWC::caspase	6	3	2	2	50.6996	240.1340

AWC::caspase	6	3	2	3	22.6332	210.8005
AWC::caspase	6	3	2	4	20.6537	200.6211
AWC::caspase	6	3	2	5	41.9767	155.1074
AWC::caspase	6	3	2	6	19.6607	248.4194
AWC::caspase	6	3	2	7	43.7734	131.5409
AWC::caspase	6	3	2	8	30.9269	211.8045
AWC::caspase	6	3	2	9	63.0385	9.0635
AWC::caspase	6	3	2	10	60.2964	236.5277
AWC::caspase	6	3	2	11	52.6016	173.0692
AWC::caspase	6	3	2	12	47.5499	177.5487
AWC::caspase	6	3	2	13	23.2197	216.0336
AWC::caspase	6	3	2	14	72.3582	185.2016
AWC::caspase	6	3	2	15	55.6791	257.0444
AWC::caspase	6	3	2	16	17.9742	185.6315
AWC::caspase	6	3	2	17	35.0192	174.7048
AWC::caspase	6	3	3	1	14.1616	145.9295
AWC::caspase	6	3	3	2	45.2198	219.0935
AWC::caspase	6	3	3	3	46.5199	189.5215
AWC::caspase	6	3	3	4	34.4911	151.8741
AWC::caspase	6	3	3	5	33.4294	140.2724
AWC::caspase	6	3	3	6	35.2020	173.8535
AWC::caspase	6	3	3	7	52.5076	216.5927
AWC::caspase	6	3	3	8	27.7243	203.2042
AWC::caspase	6	3	3	9	108.7597	78.1825
AWC::caspase	6	3	3	10	30.2145	226.0903
AWC::caspase	6	3	3	11	39.2578	129.5737
AWC::caspase	6	3	3	12	48.8337	155.0160
AWC::caspase	6	3	3	13	48.6544	126.6442
AWC::caspase	6	3	3	14	47.9144	20.7114
AWC::caspase	6	3	3	15	71.3072	31.8086
AWC::caspase	6	3	3	16	57.1243	128.4190
AWC::caspase	6	3	3	17	39.8561	116.1844
AWC::caspase	6	3	3	18	13.6155	171.4174
AWC::caspase	6	3	3	19	38.6413	208.6264
AWC::caspase	6	3	3	20	16.7413	206.1453
AWC::caspase	6	3	3	21	37.5883	131.0339
AWC::caspase	6	3	3	22	59.9528	234.7621
AWC::caspase	6	3	3	23	9.5437	198.9920
AWC::caspase	6	3	3	24	45.5133	165.3729
AWC::caspase	6	3	4	1	54.6598	165.8379
AWC::caspase	6	3	4	2	48.3966	178.9798
AWC::caspase	6	3	4	3	15.5426	205.6321
AWC::caspase	6	3	4	4	69.2918	133.6368
AWC::caspase	6	3	4	5	35.5892	192.7614
AWC::caspase	6	3	4	6	29.5995	191.4158
AWC::caspase	6	3	4	7	33.4146	160.2418
AWC::caspase	6	3	4	8	19.7934	129.6974
AWC::caspase	6	3	4	9	44.8229	154.3087
AWC::caspase	6	3	4	10	40.7748	207.2100
AWC::caspase	6	3	4	11	25.3383	147.6364
AWC::caspase	6	3	4	12	31.1137	128.2202
AWC::caspase	6	3	4	13	48.5804	183.4899
AWC::caspase	6	3	4	14	53.7521	122.8191
AWC::caspase	6	3	4	15	108.3632	231.9867
AWC::caspase	6	3	4	16	55.8206	122.1867
AWC::caspase	6	3	4	17	70.1203	106.2963
AWC::caspase	6	3	4	18	49.1621	110.7671
AWC::caspase	6	3	4	19	43.4320	94.4989
ASH::caspase	0	1	1	1	60.5429	89.1077
ASH::caspase	0	1	1	2	97.4461	210.8643
ASH::caspase	0	1	1	3	88.3634	163.8711
ASH::caspase	0	1	1	4	110.5923	171.6202
ASH::caspase	0	1	1	5	128.8501	151.1459
ASH::caspase	0	1	1	6	89.5431	156.4797
ASH::caspase	0	1	1	7	92.7573	57.6352
ASH::caspase	0	1	1	8	105.5364	194.6246
ASH::caspase	0	1	1	9	94.2493	9.4329
ASH::caspase	0	1	1	10	92.9236	106.5895

ASH::caspase	0	1	1	11	111.5700	48.7856
ASH::caspase	0	1	1	12	86.0765	118.3181
ASH::caspase	0	1	1	13	87.2713	208.1846
ASH::caspase	0	1	1	14	13.7493	203.0583
ASH::caspase	0	1	1	15	101.9841	151.9104
ASH::caspase	0	1	1	16	48.2057	155.3127
ASH::caspase	0	1	1	17	32.8489	255.9464
ASH::caspase	0	1	1	18	118.3716	141.6628
ASH::caspase	0	1	1	19	93.7263	111.5543
ASH::caspase	0	1	2	1	51.4279	211.6863
ASH::caspase	0	1	2	2	47.5294	285.6180
ASH::caspase	0	1	2	3	101.3026	5.8575
ASH::caspase	0	1	2	4	119.7416	115.0900
ASH::caspase	0	1	2	5	165.3760	207.3605
ASH::caspase	0	1	2	6	161.7189	237.8591
ASH::caspase	0	1	2	7	75.7768	276.9453
ASH::caspase	0	1	2	8	27.9298	265.2031
ASH::caspase	0	1	2	9	107.4269	118.2988
ASH::caspase	0	1	2	10	23.8552	275.7671
ASH::caspase	0	1	2	11	104.2395	209.7723
ASH::caspase	0	1	2	12	150.0141	318.6087
ASH::caspase	0	1	2	13	62.2395	96.0650
ASH::caspase	0	1	2	14	98.7316	271.4318
ASH::caspase	0	1	2	15	74.4857	192.9819
ASH::caspase	0	1	3	1	143.2600	161.5154
ASH::caspase	0	1	3	2	49.7904	240.7961
ASH::caspase	0	1	3	3	84.0922	275.8880
ASH::caspase	0	1	3	4	92.9354	5.2606
ASH::caspase	0	1	3	5	54.8073	197.5178
ASH::caspase	0	1	3	6	112.5491	223.3462
ASH::caspase	0	1	3	7	54.1019	218.2124
ASH::caspase	0	1	3	8	81.1283	247.0947
ASH::caspase	0	1	3	9	100.9784	70.5608
ASH::caspase	0	1	3	10	75.3145	189.8371
ASH::caspase	0	1	3	11	118.7606	162.1996
ASH::caspase	0	1	3	12	92.4517	74.6939
ASH::caspase	0	1	3	13	37.1842	259.8386
ASH::caspase	0	1	4	1	76.6948	189.6068
ASH::caspase	0	1	4	2	56.0652	227.5615
ASH::caspase	0	1	4	3	135.7450	161.0633
ASH::caspase	0	1	4	4	127.3101	271.7197
ASH::caspase	0	1	4	5	102.2970	26.8796
ASH::caspase	0	1	4	6	170.9128	59.6535
ASH::caspase	0	1	4	7	84.2889	296.2949
ASH::caspase	0	1	4	8	138.9104	177.7628
ASH::caspase	0	1	4	9	108.0654	242.8162
ASH::caspase	0	1	4	10	89.8262	5.3919
ASH::caspase	0	1	4	11	108.1994	282.4700
ASH::caspase	0	1	4	12	11.9134	225.5541
ASH::caspase	0	1	4	13	89.6463	188.5978
ASH::caspase	0	2	1	1	78.3234	70.9072
ASH::caspase	0	2	1	2	118.5995	163.2523
ASH::caspase	0	2	1	3	91.3161	98.0429
ASH::caspase	0	2	1	4	111.8877	135.4791
ASH::caspase	0	2	1	5	24.1229	133.2484
ASH::caspase	0	2	1	6	110.8972	119.1995
ASH::caspase	0	2	1	7	129.4368	105.8998
ASH::caspase	0	2	1	8	97.9992	52.5114
ASH::caspase	0	2	1	9	89.3905	145.2124
ASH::caspase	0	2	1	10	172.7165	175.6571
ASH::caspase	0	2	1	11	121.4639	97.7484
ASH::caspase	0	2	1	12	108.1794	128.4036
ASH::caspase	0	2	1	13	100.9606	119.2732
ASH::caspase	0	2	2	1	134.6731	70.6584
ASH::caspase	0	2	2	2	47.2845	160.1885
ASH::caspase	0	2	2	3	91.6574	94.7170
ASH::caspase	0	2	2	4	97.1004	99.1202
ASH::caspase	0	2	2	5	91.1302	102.0890

ASH::caspase	0	2	2	6	104.1852	127.0276
ASH::caspase	0	2	2	7	92.5319	109.5407
ASH::caspase	0	2	2	8	25.2064	244.3903
ASH::caspase	0	2	2	9	82.2438	82.8819
ASH::caspase	0	2	2	10	83.8536	73.5199
ASH::caspase	0	2	2	11	113.7932	187.2825
ASH::caspase	0	2	3	1	93.1553	92.7752
ASH::caspase	0	2	3	2	41.1075	208.6931
ASH::caspase	0	2	3	3	77.9114	196.5382
ASH::caspase	0	2	3	4	68.5620	151.9227
ASH::caspase	0	2	4	1	165.1583	202.1491
ASH::caspase	0	2	4	2	73.2850	139.2506
ASH::caspase	0	2	4	3	74.8898	144.1509
ASH::caspase	0	2	4	4	75.0307	134.8513
ASH::caspase	0	2	4	5	62.6482	158.3779
ASH::caspase	0	3	1	1	64.2638	85.2482
ASH::caspase	0	3	1	2	126.6484	109.7691
ASH::caspase	0	3	1	3	92.9446	85.6494
ASH::caspase	0	3	1	4	85.6679	132.5704
ASH::caspase	0	3	1	5	74.1036	158.3426
ASH::caspase	0	3	1	6	70.5344	30.6703
ASH::caspase	0	3	1	7	96.2682	105.9429
ASH::caspase	0	3	1	8	81.2539	193.1092
ASH::caspase	0	3	1	9	119.1668	88.1751
ASH::caspase	0	3	1	10	70.5989	117.0260
ASH::caspase	0	3	1	11	104.1884	98.2364
ASH::caspase	0	3	1	12	97.5623	66.9243
ASH::caspase	0	3	1	13	90.2975	113.8471
ASH::caspase	0	3	1	14	84.5447	65.1912
ASH::caspase	0	3	1	15	88.6632	124.2318
ASH::caspase	0	3	1	16	62.8002	140.4526
ASH::caspase	0	3	1	17	92.8524	97.4178
ASH::caspase	0	3	1	18	47.4891	106.6231
ASH::caspase	0	3	1	19	108.1900	104.8123
ASH::caspase	0	3	1	20	81.7016	99.6040
ASH::caspase	0	3	1	21	99.1312	119.3180
ASH::caspase	0	3	2	1	44.0721	126.6698
ASH::caspase	0	3	2	2	136.9720	128.3605
ASH::caspase	0	3	2	3	60.9570	134.0013
ASH::caspase	0	3	2	4	123.7571	127.6045
ASH::caspase	0	3	2	5	75.9830	126.6646
ASH::caspase	0	3	2	6	149.7059	147.5307
ASH::caspase	0	3	2	7	31.3988	189.4354
ASH::caspase	0	3	2	8	60.7855	89.9701
ASH::caspase	0	3	2	9	129.6849	137.8993
ASH::caspase	0	3	2	10	124.7400	130.9932
ASH::caspase	0	3	2	11	90.5268	91.8021
ASH::caspase	0	3	2	12	100.2519	26.3897
ASH::caspase	0	3	2	13	99.8734	173.0496
ASH::caspase	0	3	2	14	121.1079	154.2394
ASH::caspase	0	3	2	15	80.5697	100.9575
ASH::caspase	0	3	2	16	160.3190	155.8407
ASH::caspase	0	3	2	17	78.4167	96.0483
ASH::caspase	0	3	2	18	89.3154	89.9972
ASH::caspase	0	3	2	19	107.7170	82.3932
ASH::caspase	0	3	2	20	117.7370	109.5087
ASH::caspase	0	3	2	21	138.5415	124.1152
ASH::caspase	0	3	2	22	44.6317	211.4818
ASH::caspase	0	3	2	23	138.1926	113.6307
ASH::caspase	0	3	2	24	51.0243	30.4873
ASH::caspase	0	3	2	25	87.6667	90.9949
ASH::caspase	0	3	3	1	86.4914	140.4410
ASH::caspase	0	3	3	2	113.6608	94.0430
ASH::caspase	0	3	3	3	106.2794	18.0400
ASH::caspase	0	3	3	4	159.8571	136.8030
ASH::caspase	0	3	3	5	122.4250	86.0165
ASH::caspase	0	3	3	6	97.8577	88.9666
ASH::caspase	0	3	3	7	110.0185	152.0221

ASH::caspase	0	3	3	8	138.2861	41.9077
ASH::caspase	0	3	3	9	121.2046	148.5553
ASH::caspase	0	3	3	10	159.6844	144.9682
ASH::caspase	0	3	3	11	89.7626	181.3879
ASH::caspase	0	3	3	12	33.9098	174.1162
ASH::caspase	0	3	3	13	66.8901	110.8960
ASH::caspase	0	3	3	14	83.3831	128.6041
ASH::caspase	0	3	3	15	34.3839	113.0314
ASH::caspase	0	3	3	16	41.8108	138.9761
ASH::caspase	0	3	3	17	111.4786	131.4588
ASH::caspase	0	3	3	18	73.4559	128.1349
ASH::caspase	0	3	3	19	46.4917	107.6258
ASH::caspase	0	3	3	20	137.4254	111.8234
ASH::caspase	0	3	4	1	134.2951	153.5645
ASH::caspase	0	3	4	2	129.6266	106.3962
ASH::caspase	0	3	4	3	92.3965	9.1335
ASH::caspase	0	3	4	4	121.3291	148.5900
ASH::caspase	0	3	4	5	106.0471	117.9413
ASH::caspase	0	3	4	6	124.1169	58.8870
ASH::caspase	0	3	4	7	96.8922	142.6851
ASH::caspase	0	3	4	8	123.2818	27.3598
ASH::caspase	0	3	4	9	159.0119	125.8483
ASH::caspase	0	3	4	10	119.4754	134.1071
ASH::caspase	0	3	4	11	69.1383	96.0521
ASH::caspase	0	3	4	12	128.5585	146.3418
ASH::caspase	0	3	4	13	74.8315	121.5071
ASH::caspase	0	3	4	14	89.2634	108.5140
ASH::caspase	0	3	4	15	119.1594	137.4129
ASH::caspase	0	3	4	16	98.4966	105.7939
ASH::caspase	0	3	4	17	92.1802	1.5165
ASH::caspase	0	3	4	18	90.0483	70.6349
ASH::caspase	0	3	4	19	87.7417	139.9715
ASH::caspase	1.5	1	1	1	6.1661	224.0888
ASH::caspase	1.5	1	1	2	10.4313	240.3896
ASH::caspase	1.5	1	1	3	11.1591	236.1827
ASH::caspase	1.5	1	1	4	47.2141	184.8316
ASH::caspase	1.5	1	1	5	21.8099	174.5211
ASH::caspase	1.5	1	1	6	31.6368	155.7354
ASH::caspase	1.5	1	1	7	34.1834	154.0494
ASH::caspase	1.5	1	1	8	38.3578	184.6269
ASH::caspase	1.5	1	1	9	30.1040	289.2324
ASH::caspase	1.5	1	1	10	21.3418	170.6512
ASH::caspase	1.5	1	1	11	22.7071	194.2499
ASH::caspase	1.5	1	1	12	20.7545	207.8699
ASH::caspase	1.5	1	1	13	11.5383	190.1578
ASH::caspase	1.5	1	1	14	14.0184	342.8710
ASH::caspase	1.5	1	1	15	5.0599	250.5605
ASH::caspase	1.5	1	1	16	19.4859	286.1936
ASH::caspase	1.5	1	1	17	14.1818	232.4923
ASH::caspase	1.5	1	2	1	11.6552	317.8965
ASH::caspase	1.5	1	2	2	8.5110	219.8583
ASH::caspase	1.5	1	2	3	10.6141	278.4241
ASH::caspase	1.5	1	2	4	12.6511	217.8482
ASH::caspase	1.5	1	2	5	9.5472	211.9952
ASH::caspase	1.5	1	2	6	18.2899	252.2766
ASH::caspase	1.5	1	2	7	7.5108	284.6551
ASH::caspase	1.5	1	2	8	4.8260	322.9110
ASH::caspase	1.5	1	2	9	12.9433	326.7637
ASH::caspase	1.5	1	2	10	7.0184	166.7441
ASH::caspase	1.5	1	2	11	21.4152	167.8725
ASH::caspase	1.5	1	2	12	15.1582	199.5118
ASH::caspase	1.5	1	2	13	13.0648	113.2964
ASH::caspase	1.5	1	2	14	80.7715	113.7007
ASH::caspase	1.5	1	2	15	56.5542	72.5122
ASH::caspase	1.5	1	2	16	80.3915	185.8131
ASH::caspase	1.5	1	2	17	4.6196	195.2955
ASH::caspase	1.5	1	2	18	6.6260	146.0716
ASH::caspase	1.5	1	2	19	10.0563	240.6402

ASH::caspase	1.5	1	2	20	68.1121	67.2423
ASH::caspase	1.5	1	2	21	84.3827	75.6433
ASH::caspase	1.5	1	2	22	52.9512	173.0149
ASH::caspase	1.5	1	2	23	12.6184	288.4657
ASH::caspase	1.5	1	2	24	11.0446	307.4880
ASH::caspase	1.5	1	2	25	11.7936	317.8632
ASH::caspase	1.5	1	2	26	15.4158	200.5658
ASH::caspase	1.5	1	2	27	7.2588	221.0464
ASH::caspase	1.5	1	2	28	8.5724	316.9884
ASH::caspase	1.5	1	2	29	15.9594	275.2363
ASH::caspase	1.5	1	2	30	4.0969	229.1596
ASH::caspase	1.5	1	2	31	9.8785	390.9125
ASH::caspase	1.5	1	3	1	6.6616	185.1609
ASH::caspase	1.5	1	3	2	5.8852	222.9982
ASH::caspase	1.5	1	3	3	17.1879	231.8896
ASH::caspase	1.5	1	3	4	21.5703	291.4411
ASH::caspase	1.5	1	3	5	17.0192	126.1695
ASH::caspase	1.5	1	3	6	18.3002	245.6607
ASH::caspase	1.5	1	3	7	24.8756	316.7212
ASH::caspase	1.5	1	3	8	8.8086	217.5794
ASH::caspase	1.5	1	3	9	17.8077	133.4476
ASH::caspase	1.5	1	3	10	10.7144	198.7839
ASH::caspase	1.5	1	3	11	7.3644	272.6819
ASH::caspase	1.5	1	3	12	13.2754	212.0057
ASH::caspase	1.5	1	3	13	20.3812	222.4249
ASH::caspase	1.5	1	3	14	17.5974	156.3057
ASH::caspase	1.5	1	3	15	17.1755	158.7470
ASH::caspase	1.5	1	3	16	5.8289	199.4707
ASH::caspase	1.5	1	3	17	9.2446	124.0755
ASH::caspase	1.5	1	3	18	4.7104	187.0625
ASH::caspase	1.5	1	3	19	9.1704	101.4618
ASH::caspase	1.5	1	3	20	6.2663	148.7412
ASH::caspase	1.5	1	3	21	13.3363	118.6376
ASH::caspase	1.5	1	3	22	4.1747	98.6504
ASH::caspase	1.5	1	3	23	15.6180	279.1048
ASH::caspase	1.5	1	3	24	39.2960	171.6330
ASH::caspase	1.5	1	3	25	24.4212	101.1289
ASH::caspase	1.5	1	4	1	9.0553	184.5118
ASH::caspase	1.5	1	4	2	6.8809	228.8678
ASH::caspase	1.5	1	4	3	16.6021	156.8862
ASH::caspase	1.5	1	4	4	11.6380	199.8167
ASH::caspase	1.5	1	4	5	14.4770	163.1801
ASH::caspase	1.5	1	4	6	8.7397	153.2333
ASH::caspase	1.5	1	4	7	12.2584	124.7956
ASH::caspase	1.5	1	4	8	13.3408	109.9666
ASH::caspase	1.5	1	4	9	11.7282	201.3400
ASH::caspase	1.5	1	4	10	8.2810	139.6383
ASH::caspase	1.5	1	4	11	8.8273	182.6197
ASH::caspase	1.5	1	4	12	34.0035	110.1752
ASH::caspase	1.5	1	4	13	10.0610	165.8883
ASH::caspase	1.5	1	4	14	13.8741	120.1639
ASH::caspase	1.5	1	4	15	13.8612	129.5821
ASH::caspase	1.5	1	4	16	25.0485	92.0372
ASH::caspase	1.5	1	4	17	72.3815	114.0713
ASH::caspase	1.5	1	4	18	12.8252	215.7982
ASH::caspase	1.5	1	4	19	12.0543	230.0176
ASH::caspase	1.5	1	4	20	9.0779	112.8794
ASH::caspase	1.5	1	4	21	17.4515	121.6401
ASH::caspase	1.5	1	4	22	19.4369	124.4476
ASH::caspase	1.5	1	4	23	8.9849	178.8339
ASH::caspase	1.5	2	1	1	18.5343	222.2703
ASH::caspase	1.5	2	1	2	10.5379	212.0819
ASH::caspase	1.5	2	1	3	11.7025	225.3292
ASH::caspase	1.5	2	1	4	15.5394	247.9329
ASH::caspase	1.5	2	1	5	7.1805	245.7495
ASH::caspase	1.5	2	1	6	45.0104	170.5032
ASH::caspase	1.5	2	1	7	7.3723	293.5162
ASH::caspase	1.5	2	1	8	14.1172	195.3295

ASH::caspase	1.5	2	1	9	27.1779	211.2116
ASH::caspase	1.5	2	1	10	8.5606	205.4591
ASH::caspase	1.5	2	1	11	4.9752	205.5098
ASH::caspase	1.5	2	1	12	17.5109	215.1893
ASH::caspase	1.5	2	1	13	13.7933	325.4634
ASH::caspase	1.5	2	1	14	14.2115	167.8292
ASH::caspase	1.5	2	2	1	15.2372	231.8556
ASH::caspase	1.5	2	2	2	9.0989	279.9858
ASH::caspase	1.5	2	2	3	13.6280	176.1390
ASH::caspase	1.5	2	2	4	5.7011	179.3497
ASH::caspase	1.5	2	2	5	7.4447	142.7834
ASH::caspase	1.5	2	2	6	8.8068	246.2636
ASH::caspase	1.5	2	2	7	6.6022	267.6253
ASH::caspase	1.5	2	2	8	56.5521	153.6145
ASH::caspase	1.5	2	2	9	18.6856	200.3129
ASH::caspase	1.5	2	2	10	8.7072	319.3413
ASH::caspase	1.5	2	2	11	10.4001	308.3550
ASH::caspase	1.5	2	2	12	12.4425	216.4940
ASH::caspase	1.5	2	2	13	14.2162	156.8740
ASH::caspase	1.5	2	2	14	16.3616	282.8583
ASH::caspase	1.5	2	2	15	42.2324	217.3000
ASH::caspase	1.5	2	2	16	6.9589	188.1447
ASH::caspase	1.5	2	2	17	29.2439	121.2281
ASH::caspase	1.5	2	2	18	21.1672	187.3239
ASH::caspase	1.5	2	2	19	11.2508	319.3183
ASH::caspase	1.5	2	2	20	11.0036	210.0348
ASH::caspase	1.5	2	2	21	6.6871	246.7977
ASH::caspase	1.5	2	3	1	3.6064	270.7766
ASH::caspase	1.5	2	3	2	16.7769	221.9694
ASH::caspase	1.5	2	3	3	7.6731	130.0856
ASH::caspase	1.5	2	3	4	22.3410	80.3834
ASH::caspase	1.5	2	3	5	10.2115	248.2548
ASH::caspase	1.5	2	3	6	4.6308	183.5728
ASH::caspase	1.5	2	3	7	8.8252	219.1853
ASH::caspase	1.5	2	3	8	7.6968	151.5489
ASH::caspase	1.5	2	3	9	8.7804	137.9074
ASH::caspase	1.5	2	3	10	15.2550	150.4428
ASH::caspase	1.5	2	3	11	22.0653	73.6344
ASH::caspase	1.5	2	3	12	5.3128	218.4675
ASH::caspase	1.5	2	3	13	16.4731	136.2482
ASH::caspase	1.5	2	3	14	8.4284	99.1056
ASH::caspase	1.5	2	3	15	14.4019	107.0866
ASH::caspase	1.5	2	4	1	13.1400	261.4341
ASH::caspase	1.5	2	4	2	23.3314	228.0728
ASH::caspase	1.5	2	4	3	17.2645	51.6669
ASH::caspase	1.5	2	4	4	11.2978	202.7369
ASH::caspase	1.5	2	4	5	8.3394	114.5232
ASH::caspase	1.5	2	4	6	23.9791	158.7345
ASH::caspase	1.5	2	4	7	36.6940	94.2053
ASH::caspase	1.5	2	4	8	29.3677	68.6010
ASH::caspase	1.5	2	4	9	12.5839	164.7940
ASH::caspase	1.5	2	4	10	8.7359	229.0278
ASH::caspase	1.5	2	4	11	11.0379	161.9139
ASH::caspase	1.5	2	4	12	12.5059	208.8978
ASH::caspase	1.5	3	1	1	6.1371	174.4353
ASH::caspase	1.5	3	1	2	6.6781	239.4835
ASH::caspase	1.5	3	1	3	7.8397	170.8027
ASH::caspase	1.5	3	1	4	22.0760	206.8561
ASH::caspase	1.5	3	1	5	9.5227	172.6047
ASH::caspase	1.5	3	1	6	6.2599	196.6363
ASH::caspase	1.5	3	1	7	105.9499	2.6951
ASH::caspase	1.5	3	1	8	88.2211	27.2819
ASH::caspase	1.5	3	2	1	9.4325	154.4953
ASH::caspase	1.5	3	2	2	8.4470	101.3045
ASH::caspase	1.5	3	2	3	12.6335	136.5103
ASH::caspase	1.5	3	2	4	6.4439	139.8118
ASH::caspase	1.5	3	2	5	12.7890	74.7695
ASH::caspase	1.5	3	2	6	8.2892	107.2420

ASH::caspase	1.5	3	3	1	10.9456	159.0092
ASH::caspase	1.5	3	3	2	35.0371	160.9628
ASH::caspase	1.5	3	3	3	17.9810	153.2485
ASH::caspase	1.5	3	3	4	9.8975	92.5873
ASH::caspase	1.5	3	3	5	14.3563	146.1721
ASH::caspase	1.5	3	3	6	9.5812	143.1580
ASH::caspase	1.5	3	3	7	15.2210	164.7210
ASH::caspase	1.5	3	3	8	14.0988	143.4370
ASH::caspase	1.5	3	3	9	12.8828	124.6434
ASH::caspase	1.5	3	3	10	9.3394	127.4716
ASH::caspase	1.5	3	3	11	12.3724	140.9360
ASH::caspase	1.5	3	3	12	130.9218	94.6564
ASH::caspase	1.5	3	3	13	15.9478	101.2695
ASH::caspase	1.5	3	4	1	15.2310	91.5874
ASH::caspase	1.5	3	4	2	36.6603	131.2892
ASH::caspase	1.5	3	4	3	9.6114	148.2982
ASH::caspase	1.5	3	4	4	11.6809	133.6231
ASH::caspase	1.5	3	4	5	14.2124	133.5841
ASH::caspase	1.5	3	4	6	13.8511	162.8176
ASH::caspase	1.5	3	4	7	13.8366	87.6324
ASH::caspase	1.5	3	4	8	20.3822	163.1473
ASH::caspase	1.5	3	4	9	11.2994	137.7152
ASH::caspase	1.5	3	4	10	15.3412	134.8877
ASH::caspase	1.5	3	4	11	10.6743	124.0976
ASH::caspase	3	1	1	1	64.8387	162.1481
ASH::caspase	3	1	1	2	42.6646	285.6521
ASH::caspase	3	1	1	3	31.2261	228.6880
ASH::caspase	3	1	1	4	14.5448	179.2730
ASH::caspase	3	1	1	5	9.7102	250.6231
ASH::caspase	3	1	1	6	17.3008	263.8065
ASH::caspase	3	1	1	7	7.8109	284.0814
ASH::caspase	3	1	2	1	12.7822	275.9748
ASH::caspase	3	1	2	2	8.2546	222.8654
ASH::caspase	3	1	2	3	7.9738	137.9880
ASH::caspase	3	1	2	4	8.7614	152.9187
ASH::caspase	3	1	2	5	29.0254	158.4304
ASH::caspase	3	1	2	6	7.3233	111.9677
ASH::caspase	3	1	2	7	18.8511	202.4493
ASH::caspase	3	1	2	8	29.0897	239.9904
ASH::caspase	3	1	2	9	14.5312	184.3254
ASH::caspase	3	1	2	10	10.0388	160.3662
ASH::caspase	3	1	2	11	28.2424	154.3622
ASH::caspase	3	1	2	12	24.0163	189.7397
ASH::caspase	3	1	2	13	9.5627	196.0532
ASH::caspase	3	1	2	14	88.3925	8.7811
ASH::caspase	3	1	2	15	25.6508	215.6653
ASH::caspase	3	1	2	16	24.8816	271.0920
ASH::caspase	3	1	2	17	20.9212	38.7806
ASH::caspase	3	1	2	18	10.0415	62.7834
ASH::caspase	3	1	3	1	17.5444	163.6509
ASH::caspase	3	1	3	2	16.1567	250.0767
ASH::caspase	3	1	3	3	19.7444	181.4507
ASH::caspase	3	1	3	4	41.7882	236.3059
ASH::caspase	3	1	3	5	19.0797	190.2228
ASH::caspase	3	1	3	6	18.3630	187.0569
ASH::caspase	3	1	3	7	6.2816	304.5474
ASH::caspase	3	1	3	8	21.8275	182.6118
ASH::caspase	3	1	3	9	22.4716	138.0070
ASH::caspase	3	1	3	10	29.7476	90.5530
ASH::caspase	3	1	3	11	7.2896	100.7048
ASH::caspase	3	1	3	12	17.9864	113.3337
ASH::caspase	3	1	3	13	26.1699	124.7381
ASH::caspase	3	1	3	14	22.6864	137.0350
ASH::caspase	3	1	3	15	15.8473	185.7484
ASH::caspase	3	1	3	16	15.8387	159.2367
ASH::caspase	3	1	3	17	11.7395	118.0089
ASH::caspase	3	1	3	18	15.2336	302.8228
ASH::caspase	3	1	3	19	31.3457	228.3269

ASH::caspase	3	1	4	1	24.9787	262.9519
ASH::caspase	3	1	4	2	25.6911	149.4934
ASH::caspase	3	1	4	3	29.9699	203.0251
ASH::caspase	3	1	4	4	14.1143	173.2418
ASH::caspase	3	1	4	5	26.1270	231.9312
ASH::caspase	3	1	4	6	41.1122	234.5586
ASH::caspase	3	1	4	7	15.3276	186.7334
ASH::caspase	3	1	4	8	20.8024	46.3613
ASH::caspase	3	1	4	9	43.9195	216.8853
ASH::caspase	3	1	4	10	27.2072	144.1605
ASH::caspase	3	1	4	11	38.2766	56.6578
ASH::caspase	3	1	4	12	17.1631	226.7058
ASH::caspase	3	1	4	13	48.5738	48.9485
ASH::caspase	3	1	4	14	27.5441	222.0908
ASH::caspase	3	1	4	15	27.3606	200.1478
ASH::caspase	3	1	4	16	32.6200	172.8809
ASH::caspase	3	2	1	1	52.1713	161.0944
ASH::caspase	3	2	1	2	28.0721	222.7603
ASH::caspase	3	2	1	3	44.8741	224.3813
ASH::caspase	3	2	1	4	26.9716	246.1488
ASH::caspase	3	2	1	5	84.5035	68.3321
ASH::caspase	3	2	1	6	28.3986	182.8377
ASH::caspase	3	2	1	7	36.8064	171.0689
ASH::caspase	3	2	1	8	49.7790	256.3828
ASH::caspase	3	2	1	9	45.6723	119.1121
ASH::caspase	3	2	1	10	52.6822	123.7994
ASH::caspase	3	2	2	1	15.1778	232.4823
ASH::caspase	3	2	2	2	29.0422	189.1560
ASH::caspase	3	2	2	3	13.4878	164.1830
ASH::caspase	3	2	2	4	39.7696	189.8834
ASH::caspase	3	2	2	5	16.4544	224.3816
ASH::caspase	3	2	2	6	36.4055	161.0039
ASH::caspase	3	2	2	7	34.8246	174.9217
ASH::caspase	3	2	2	8	45.1559	242.6055
ASH::caspase	3	2	2	9	28.7142	240.6198
ASH::caspase	3	2	2	10	22.4384	259.0430
ASH::caspase	3	2	2	11	10.1802	278.9910
ASH::caspase	3	2	2	12	5.4950	219.5928
ASH::caspase	3	2	2	13	16.8819	200.4411
ASH::caspase	3	2	3	1	12.0825	240.7973
ASH::caspase	3	2	3	2	16.5098	130.1532
ASH::caspase	3	2	3	3	20.6219	168.6940
ASH::caspase	3	2	3	4	26.3120	189.8429
ASH::caspase	3	2	3	5	30.9979	149.7407
ASH::caspase	3	2	3	6	11.7142	193.2398
ASH::caspase	3	2	3	7	10.0269	184.3208
ASH::caspase	3	2	3	8	8.2165	129.0546
ASH::caspase	3	2	3	9	8.9858	227.0775
ASH::caspase	3	2	3	10	39.7703	122.6624
ASH::caspase	3	2	3	11	29.8113	239.8069
ASH::caspase	3	2	3	12	31.5202	168.5707
ASH::caspase	3	2	3	13	6.0264	160.0266
ASH::caspase	3	2	3	14	20.1212	92.7392
ASH::caspase	3	2	3	15	6.5265	231.4054
ASH::caspase	3	2	3	16	32.8441	157.5390
ASH::caspase	3	2	3	17	17.7563	190.6672
ASH::caspase	3	2	3	18	14.7554	294.9547
ASH::caspase	3	2	3	19	15.9290	238.1572
ASH::caspase	3	2	4	1	31.0926	250.3142
ASH::caspase	3	2	4	2	28.1907	200.0407
ASH::caspase	3	2	4	3	19.1958	211.4292
ASH::caspase	3	2	4	4	18.1753	228.4066
ASH::caspase	3	2	4	5	26.1663	146.1789
ASH::caspase	3	2	4	6	31.3073	182.0024
ASH::caspase	3	2	4	7	21.8507	125.9130
ASH::caspase	3	2	4	8	37.6903	98.3222
ASH::caspase	3	2	4	9	33.4596	109.0410
ASH::caspase	3	2	4	10	6.4349	181.2101

ASH::caspase	3	2	4	11	18.9369	216.4518
ASH::caspase	3	3	1	1	24.2633	180.6957
ASH::caspase	3	3	1	2	58.0846	172.2404
ASH::caspase	3	3	1	3	57.5844	220.5249
ASH::caspase	3	3	1	4	35.0762	182.6187
ASH::caspase	3	3	1	5	6.5376	189.5169
ASH::caspase	3	3	1	6	103.9779	4.1261
ASH::caspase	3	3	1	7	10.3363	251.1468
ASH::caspase	3	3	1	8	96.5859	253.7021
ASH::caspase	3	3	1	9	10.7999	199.9792
ASH::caspase	3	3	1	10	23.7184	178.4353
ASH::caspase	3	3	1	11	10.5000	228.4294
ASH::caspase	3	3	2	1	16.0090	159.8161
ASH::caspase	3	3	2	2	24.3593	248.8049
ASH::caspase	3	3	2	3	27.1873	203.4468
ASH::caspase	3	3	2	4	15.3616	177.1226
ASH::caspase	3	3	2	5	26.3604	225.6285
ASH::caspase	3	3	2	6	8.8229	226.5308
ASH::caspase	3	3	2	7	30.3764	232.8300
ASH::caspase	3	3	2	8	33.6356	238.8211
ASH::caspase	3	3	2	9	66.6134	202.0098
ASH::caspase	3	3	2	10	93.3220	5.1954
ASH::caspase	3	3	2	11	36.4660	125.8813
ASH::caspase	3	3	2	12	11.2597	220.3470
ASH::caspase	3	3	2	13	31.6561	146.3763
ASH::caspase	3	3	2	14	11.2054	249.5488
ASH::caspase	3	3	2	15	31.6260	198.1807
ASH::caspase	3	3	2	16	20.8493	220.8207
ASH::caspase	3	3	2	17	106.3806	116.8495
ASH::caspase	3	3	2	18	14.9318	192.7199
ASH::caspase	3	3	2	19	7.8081	183.0795
ASH::caspase	3	3	2	20	14.6227	188.3852
ASH::caspase	3	3	3	1	15.9822	192.1438
ASH::caspase	3	3	3	2	24.4797	141.2122
ASH::caspase	3	3	3	3	20.0568	214.3503
ASH::caspase	3	3	3	4	36.0819	148.8808
ASH::caspase	3	3	3	5	30.3213	180.6912
ASH::caspase	3	3	3	6	21.4010	171.5507
ASH::caspase	3	3	3	7	25.4948	106.6751
ASH::caspase	3	3	3	8	25.4772	213.8572
ASH::caspase	3	3	3	9	21.5196	208.3786
ASH::caspase	3	3	3	10	32.3646	232.6098
ASH::caspase	3	3	3	11	36.2386	225.8711
ASH::caspase	3	3	3	12	10.8585	127.0430
ASH::caspase	3	3	3	13	25.4672	182.4141
ASH::caspase	3	3	3	14	26.9785	205.3666
ASH::caspase	3	3	3	15	31.3559	178.1781
ASH::caspase	3	3	3	16	17.0180	112.2379
ASH::caspase	3	3	3	17	31.8708	115.9983
ASH::caspase	3	3	4	1	35.0373	178.6808
ASH::caspase	3	3	4	2	29.1984	204.4496
ASH::caspase	3	3	4	3	37.0190	208.9553
ASH::caspase	3	3	4	4	14.6652	224.0902
ASH::caspase	3	3	4	5	29.8198	176.0695
ASH::caspase	3	3	4	6	25.8262	139.4385
ASH::caspase	3	3	4	7	16.8937	169.4360
ASH::caspase	3	3	4	8	12.5641	248.8902
ASH::caspase	3	3	4	9	23.0528	218.0906
ASH::caspase	3	3	4	10	36.3013	179.3112
ASH::caspase	3	3	4	11	18.6012	114.2232
ASH::caspase	6	1	1	1	39.9499	225.6824
ASH::caspase	6	1	1	2	58.8189	140.2369
ASH::caspase	6	1	1	3	29.6683	281.6156
ASH::caspase	6	1	1	4	58.2963	241.0485
ASH::caspase	6	1	1	5	65.7278	225.5027
ASH::caspase	6	1	1	6	24.3320	213.8237
ASH::caspase	6	1	1	7	17.1738	233.5250
ASH::caspase	6	1	1	8	30.6378	262.1564

ASH::caspase	6	1	1	9	29.9562	194.2652
ASH::caspase	6	1	2	1	14.9635	176.5962
ASH::caspase	6	1	2	2	11.6156	138.7356
ASH::caspase	6	1	2	3	10.0703	244.1765
ASH::caspase	6	1	2	4	20.1426	238.1610
ASH::caspase	6	1	2	5	44.5780	217.9301
ASH::caspase	6	1	2	6	43.9500	182.1578
ASH::caspase	6	1	2	7	64.1743	301.1981
ASH::caspase	6	1	2	8	43.8967	267.9267
ASH::caspase	6	1	2	9	32.1267	116.1898
ASH::caspase	6	1	2	10	25.3083	270.1208
ASH::caspase	6	1	2	11	40.0533	189.4056
ASH::caspase	6	1	2	12	87.9015	201.7974
ASH::caspase	6	1	2	13	19.9043	99.1344
ASH::caspase	6	1	2	14	50.1481	240.2053
ASH::caspase	6	1	2	15	25.5094	166.4874
ASH::caspase	6	1	2	16	60.1264	218.2827
ASH::caspase	6	1	3	1	18.2520	235.4689
ASH::caspase	6	1	3	2	34.5445	151.1794
ASH::caspase	6	1	3	3	110.1774	78.4849
ASH::caspase	6	1	3	4	38.5456	210.0292
ASH::caspase	6	1	3	5	40.4979	202.1731
ASH::caspase	6	1	3	6	74.7231	159.0457
ASH::caspase	6	1	3	7	61.4932	238.5955
ASH::caspase	6	1	3	8	32.1692	91.6759
ASH::caspase	6	1	3	9	43.2175	198.3044
ASH::caspase	6	1	3	10	29.1314	159.4913
ASH::caspase	6	1	3	11	105.3973	312.0863
ASH::caspase	6	1	3	12	58.7385	228.1564
ASH::caspase	6	1	3	13	66.3648	237.5947
ASH::caspase	6	1	4	1	63.2123	237.0126
ASH::caspase	6	1	4	2	57.7482	214.8592
ASH::caspase	6	1	4	3	33.1933	194.8139
ASH::caspase	6	1	4	4	96.7314	151.3481
ASH::caspase	6	1	4	5	65.2582	316.7272
ASH::caspase	6	1	4	6	62.7322	160.7196
ASH::caspase	6	1	4	7	85.3035	240.7369
ASH::caspase	6	1	4	8	41.2992	210.8831
ASH::caspase	6	1	4	9	80.3347	228.4089
ASH::caspase	6	1	4	10	66.0221	190.9624
ASH::caspase	6	1	4	11	39.1473	201.2978
ASH::caspase	6	1	4	12	58.2953	132.8334
ASH::caspase	6	1	4	13	55.7689	94.2334
ASH::caspase	6	1	4	14	77.7285	206.5643
ASH::caspase	6	2	1	1	63.4426	252.5582
ASH::caspase	6	2	1	2	66.5953	376.9618
ASH::caspase	6	2	1	3	50.8336	318.8786
ASH::caspase	6	2	1	4	58.9112	334.9362
ASH::caspase	6	2	1	5	39.7249	258.5462
ASH::caspase	6	2	1	6	82.4859	43.9648
ASH::caspase	6	2	1	7	57.6102	239.4262
ASH::caspase	6	2	1	8	47.6766	257.8264
ASH::caspase	6	2	2	1	24.2138	153.2558
ASH::caspase	6	2	2	2	41.6515	213.4638
ASH::caspase	6	2	2	3	45.9615	160.8219
ASH::caspase	6	2	2	4	35.3244	240.1647
ASH::caspase	6	2	2	5	41.3825	280.8819
ASH::caspase	6	2	2	6	38.7234	193.5884
ASH::caspase	6	2	2	7	75.1836	272.3577
ASH::caspase	6	2	2	8	58.2551	130.6417
ASH::caspase	6	2	2	9	21.0213	246.0063
ASH::caspase	6	2	2	10	99.0827	21.1664
ASH::caspase	6	2	2	11	104.9446	57.6652
ASH::caspase	6	2	2	12	87.3607	85.2635
ASH::caspase	6	2	2	13	19.5511	200.3158
ASH::caspase	6	2	2	14	20.0155	212.3030
ASH::caspase	6	2	2	15	16.7755	146.4237
ASH::caspase	6	2	2	16	67.1635	281.0597

ASH::caspase	6	2	2	17	29.8368	211.7825
ASH::caspase	6	2	2	18	49.2518	168.3605
ASH::caspase	6	2	2	19	36.5800	172.1448
ASH::caspase	6	2	3	1	52.2442	201.1322
ASH::caspase	6	2	3	2	40.6841	218.0722
ASH::caspase	6	2	3	3	40.8777	226.3633
ASH::caspase	6	2	3	4	50.3557	174.0306
ASH::caspase	6	2	3	5	54.9674	230.0614
ASH::caspase	6	2	3	6	27.1627	240.2627
ASH::caspase	6	2	3	7	40.4302	155.6865
ASH::caspase	6	2	3	8	42.2048	196.2856
ASH::caspase	6	2	3	9	133.9295	150.7148
ASH::caspase	6	2	3	10	43.9672	213.5489
ASH::caspase	6	2	3	11	115.3251	189.1961
ASH::caspase	6	2	3	12	54.1057	231.7742
ASH::caspase	6	2	3	13	32.1296	240.3709
ASH::caspase	6	2	3	14	69.9676	66.3013
ASH::caspase	6	2	3	15	61.7790	302.3605
ASH::caspase	6	2	3	16	29.7749	157.2594
ASH::caspase	6	2	3	17	50.3044	187.1137
ASH::caspase	6	2	3	18	54.3600	206.6958
ASH::caspase	6	2	3	19	6.9453	195.8215
ASH::caspase	6	2	3	20	14.8182	236.6109
ASH::caspase	6	2	4	1	60.3602	191.1265
ASH::caspase	6	2	4	2	69.5811	240.6359
ASH::caspase	6	2	4	3	95.1533	56.0635
ASH::caspase	6	2	4	4	69.4197	176.7033
ASH::caspase	6	2	4	5	53.4015	166.4168
ASH::caspase	6	2	4	6	37.6412	184.8731
ASH::caspase	6	2	4	7	64.5088	146.8703
ASH::caspase	6	2	4	8	59.0726	224.3229
ASH::caspase	6	2	4	9	36.0066	193.3836
ASH::caspase	6	2	4	10	152.6998	263.6250
ASH::caspase	6	2	4	11	57.5204	113.9603
ASH::caspase	6	2	4	12	22.1190	147.7033
ASH::caspase	6	2	4	13	69.9522	230.2511
ASH::caspase	6	3	1	1	34.6107	178.8487
ASH::caspase	6	3	1	2	85.0192	110.6207
ASH::caspase	6	3	1	3	43.2682	115.8459
ASH::caspase	6	3	1	4	22.2456	157.7915
ASH::caspase	6	3	1	5	33.0655	192.2014
ASH::caspase	6	3	1	6	29.6739	187.4058
ASH::caspase	6	3	1	7	94.5835	2.0506
ASH::caspase	6	3	1	8	44.6872	198.1490
ASH::caspase	6	3	1	9	23.6661	191.1722
ASH::caspase	6	3	2	1	53.2013	126.4257
ASH::caspase	6	3	2	2	40.6010	203.6152
ASH::caspase	6	3	2	3	39.4714	162.8136
ASH::caspase	6	3	2	4	43.1749	190.3822
ASH::caspase	6	3	2	5	22.3846	167.8446
ASH::caspase	6	3	2	6	26.8905	122.2070
ASH::caspase	6	3	2	7	42.3429	197.3608
ASH::caspase	6	3	2	8	37.8545	169.7914
ASH::caspase	6	3	2	9	29.9783	153.7207
ASH::caspase	6	3	2	10	9.0949	186.0259
ASH::caspase	6	3	2	11	30.6914	188.3632
ASH::caspase	6	3	2	12	51.4279	88.4449
ASH::caspase	6	3	2	13	44.8295	211.6669
ASH::caspase	6	3	2	14	49.1493	230.4370
ASH::caspase	6	3	2	15	80.3786	5.7985
ASH::caspase	6	3	2	16	13.4674	176.6167
ASH::caspase	6	3	2	17	89.3745	17.7127
ASH::caspase	6	3	2	18	30.7798	155.2034
ASH::caspase	6	3	2	19	40.7394	235.9747
ASH::caspase	6	3	2	20	58.6093	216.4421
ASH::caspase	6	3	2	21	25.5233	163.5654
ASH::caspase	6	3	3	1	38.3276	205.8131
ASH::caspase	6	3	3	2	49.4792	191.9668

ASH::caspase	6	3	3	3	36.0784	157.1615
ASH::caspase	6	3	3	4	47.6312	181.4028
ASH::caspase	6	3	3	5	39.7240	192.5529
ASH::caspase	6	3	3	6	43.6214	167.4389
ASH::caspase	6	3	3	7	34.2839	128.0752
ASH::caspase	6	3	3	8	53.4274	183.8046
ASH::caspase	6	3	3	9	38.0585	127.7296
ASH::caspase	6	3	3	10	43.3746	100.0434
ASH::caspase	6	3	3	11	74.7384	177.4488
ASH::caspase	6	3	3	12	55.9150	63.8058
ASH::caspase	6	3	3	13	20.9207	129.4068
ASH::caspase	6	3	3	14	56.5866	184.4745
ASH::caspase	6	3	3	15	103.0285	24.5529
ASH::caspase	6	3	3	16	63.8457	24.8128
ASH::caspase	6	3	3	17	104.9096	7.1483
ASH::caspase	6	3	3	18	25.5887	132.4586
ASH::caspase	6	3	3	19	28.4467	169.0843
ASH::caspase	6	3	3	20	48.1743	171.7502
ASH::caspase	6	3	3	21	36.9882	124.1350
ASH::caspase	6	3	4	1	50.3783	176.2748
ASH::caspase	6	3	4	2	38.9919	174.6009
ASH::caspase	6	3	4	3	55.0719	138.8619
ASH::caspase	6	3	4	4	86.9973	86.7236
ASH::caspase	6	3	4	5	107.6463	79.6022
ASH::caspase	6	3	4	6	53.5515	178.6307
ASH::caspase	6	3	4	7	61.1884	147.7175
ASH::caspase	6	3	4	8	54.7642	67.6099
ASH::caspase	6	3	4	9	52.9285	190.1497
ASH::caspase	6	3	4	10	33.8801	129.1086
ASH::caspase	6	3	4	11	51.1311	62.0513
ASH::caspase	6	3	4	12	99.5747	135.2713
ASH::caspase	6	3	4	13	57.7294	99.2767
ASH::caspase	6	3	4	14	66.8856	152.5504
ASH::caspase	6	3	4	15	33.2083	138.0928
ASH::caspase	6	3	4	16	30.1881	168.0551
ASH::caspase	6	3	4	17	49.4453	148.9844
ASH::caspase	6	3	4	18	55.8617	115.6259
ASH::caspase	6	3	4	19	74.5685	11.6124
ASH::caspase	6	3	4	20	90.5959	41.3474
ASH::caspase	6	3	4	21	46.6439	178.4672
ASH::caspase	6	3	4	22	31.2850	53.4169
ASH::caspase	6	3	4	23	45.8494	179.4237
ASJ::caspase	0	1	1	1	70.6681	66.1253
ASJ::caspase	0	1	1	2	97.5443	117.4234
ASJ::caspase	0	1	1	3	75.9396	143.0016
ASJ::caspase	0	1	2	1	64.7508	153.3477
ASJ::caspase	0	1	2	2	65.2303	170.1690
ASJ::caspase	0	1	2	3	91.8264	85.9480
ASJ::caspase	0	1	2	4	123.5533	113.7233
ASJ::caspase	0	1	2	5	93.2493	146.6630
ASJ::caspase	0	1	3	1	18.4778	174.7400
ASJ::caspase	0	1	3	2	111.5433	116.1998
ASJ::caspase	0	1	3	3	54.5210	200.2233
ASJ::caspase	0	1	3	4	93.4166	154.1170
ASJ::caspase	0	1	3	5	79.0209	2.5485
ASJ::caspase	0	1	3	6	90.7980	192.4888
ASJ::caspase	0	2	4	1	65.4035	154.5035
ASJ::caspase	0	2	4	2	115.3584	247.0495
ASJ::caspase	0	2	4	3	20.0739	225.0985
ASJ::caspase	0	2	4	4	114.4543	164.4893
ASJ::caspase	0	2	4	5	30.5951	201.0807
ASJ::caspase	0	2	4	6	123.2729	144.4045
ASJ::caspase	0	2	1	1	84.7907	38.1555
ASJ::caspase	0	2	1	2	114.3287	80.2537
ASJ::caspase	0	2	1	3	109.0706	227.3172
ASJ::caspase	0	2	1	4	147.5688	113.6659
ASJ::caspase	0	2	1	5	137.6413	259.8003
ASJ::caspase	0	2	1	6	88.4369	68.0749

ASJ::caspase	0	2	1	7	46.8563	124.6052
ASJ::caspase	0	2	1	8	103.9899	204.4649
ASJ::caspase	0	2	1	9	35.6435	173.6189
ASJ::caspase	0	2	1	10	166.5936	197.4655
ASJ::caspase	0	2	1	11	19.8925	214.6786
ASJ::caspase	0	2	1	12	117.7151	196.4994
ASJ::caspase	0	2	1	13	165.3726	117.6423
ASJ::caspase	0	2	1	14	173.3591	235.7531
ASJ::caspase	0	2	1	15	59.9519	152.1310
ASJ::caspase	0	2	1	16	74.8405	71.9359
ASJ::caspase	0	2	2	1	69.3465	262.1023
ASJ::caspase	0	2	2	2	8.4980	225.5539
ASJ::caspase	0	2	2	3	86.2700	157.5146
ASJ::caspase	0	2	2	4	162.4621	201.1767
ASJ::caspase	0	2	2	5	90.7240	158.9749
ASJ::caspase	0	2	2	6	77.4268	127.7691
ASJ::caspase	0	2	2	7	70.6114	4.3864
ASJ::caspase	0	2	2	8	96.6744	104.8453
ASJ::caspase	0	2	2	9	89.9689	131.8724
ASJ::caspase	0	2	2	10	42.8000	163.8464
ASJ::caspase	0	2	2	11	86.4088	99.3903
ASJ::caspase	0	2	2	12	70.3992	33.3788
ASJ::caspase	0	2	2	13	60.9175	235.7717
ASJ::caspase	0	2	2	14	136.4362	181.0837
ASJ::caspase	0	2	3	1	111.8005	193.1770
ASJ::caspase	0	2	3	2	64.8126	214.2375
ASJ::caspase	0	2	3	3	18.6762	164.0708
ASJ::caspase	0	2	3	4	158.9056	127.7291
ASJ::caspase	0	2	3	5	85.9768	5.6449
ASJ::caspase	0	2	3	6	52.7986	172.6860
ASJ::caspase	0	2	3	7	94.1874	167.4422
ASJ::caspase	0	2	3	8	75.4712	92.9639
ASJ::caspase	0	2	3	9	60.0750	68.0388
ASJ::caspase	0	2	3	10	122.5107	211.0618
ASJ::caspase	0	2	3	11	101.6494	202.0904
ASJ::caspase	0	2	3	12	89.8343	201.6319
ASJ::caspase	0	2	3	13	39.1409	115.4429
ASJ::caspase	0	2	4	1	27.8034	217.3444
ASJ::caspase	0	2	4	2	88.7374	178.7627
ASJ::caspase	0	2	4	3	148.2871	199.3406
ASJ::caspase	0	2	4	4	119.5302	152.4489
ASJ::caspase	0	2	4	5	97.1775	22.6713
ASJ::caspase	0	2	4	6	49.4399	222.6206
ASJ::caspase	0	2	4	7	56.3962	45.2662
ASJ::caspase	0	2	4	8	85.2893	150.4442
ASJ::caspase	0	2	4	9	119.3114	61.6501
ASJ::caspase	0	2	4	10	92.5622	117.0601
ASJ::caspase	0	2	4	11	96.2840	16.8784
ASJ::caspase	0	2	4	12	136.7485	154.2244
ASJ::caspase	0	2	4	13	41.3740	240.2772
ASJ::caspase	0	3	1	1	103.8387	84.1566
ASJ::caspase	0	3	1	2	116.4634	61.0348
ASJ::caspase	0	3	1	3	85.1394	201.7067
ASJ::caspase	0	3	1	4	86.5825	68.3932
ASJ::caspase	0	3	1	5	75.0728	154.1078
ASJ::caspase	0	3	1	6	117.0548	137.1358
ASJ::caspase	0	3	2	1	145.4533	255.1942
ASJ::caspase	0	3	2	2	75.0701	156.1290
ASJ::caspase	0	3	2	3	84.9264	106.8923
ASJ::caspase	0	3	2	4	74.6145	111.6089
ASJ::caspase	0	3	2	5	117.5808	188.3636
ASJ::caspase	0	3	2	6	99.6092	69.3942
ASJ::caspase	0	3	2	7	84.0273	119.4815
ASJ::caspase	0	3	2	8	137.4528	33.0982
ASJ::caspase	0	3	2	9	80.7038	97.6071
ASJ::caspase	0	3	3	1	105.7667	107.9096
ASJ::caspase	0	3	3	2	63.0402	35.1230
ASJ::caspase	0	3	3	3	78.4458	79.1779

ASJ::caspase	0	3	3	4	120.3288	125.7993
ASJ::caspase	0	3	3	5	135.6219	191.3753
ASJ::caspase	0	3	3	6	82.8239	77.6747
ASJ::caspase	0	3	3	7	135.9730	172.6432
ASJ::caspase	0	3	3	8	127.7964	153.6579
ASJ::caspase	0	3	3	9	114.2254	124.7323
ASJ::caspase	0	3	3	10	32.0809	13.7016
ASJ::caspase	0	3	3	11	161.8102	195.3778
ASJ::caspase	0	3	3	12	96.0557	153.8907
ASJ::caspase	0	3	3	13	80.2573	131.7178
ASJ::caspase	0	3	4	1	102.1366	159.2021
ASJ::caspase	0	3	4	2	121.4193	92.5244
ASJ::caspase	0	3	4	3	119.8328	220.2626
ASJ::caspase	0	3	4	4	56.0108	176.4263
ASJ::caspase	0	3	4	5	73.6442	176.4153
ASJ::caspase	0	3	4	6	131.6578	111.4598
ASJ::caspase	0	3	4	7	93.2017	4.9375
ASJ::caspase	0	3	4	8	96.8743	186.3327
ASJ::caspase	0	3	4	9	79.4961	121.0202
ASJ::caspase	0	3	4	10	143.2437	189.0060
ASJ::caspase	1.5	1	1	1	14.0863	159.0539
ASJ::caspase	1.5	1	1	2	14.3285	227.4588
ASJ::caspase	1.5	1	1	3	10.7106	174.7437
ASJ::caspase	1.5	1	1	4	7.2797	227.7072
ASJ::caspase	1.5	1	1	5	9.5362	146.1738
ASJ::caspase	1.5	1	1	6	6.9586	209.4444
ASJ::caspase	1.5	1	1	7	11.2248	164.3047
ASJ::caspase	1.5	1	1	8	8.1423	234.1464
ASJ::caspase	1.5	1	1	9	8.2142	220.3445
ASJ::caspase	1.5	1	1	10	8.4951	78.0913
ASJ::caspase	1.5	1	1	11	5.5862	179.8450
ASJ::caspase	1.5	1	1	12	7.3682	193.0668
ASJ::caspase	1.5	1	1	13	18.4039	244.6561
ASJ::caspase	1.5	1	1	14	23.1658	20.8635
ASJ::caspase	1.5	1	2	1	6.8422	215.1660
ASJ::caspase	1.5	1	2	2	6.9206	233.6463
ASJ::caspase	1.5	1	2	3	6.1197	223.3399
ASJ::caspase	1.5	1	2	4	8.3904	225.2991
ASJ::caspase	1.5	1	2	5	20.5662	199.9465
ASJ::caspase	1.5	1	2	6	10.3915	140.0193
ASJ::caspase	1.5	1	2	7	9.8896	220.6559
ASJ::caspase	1.5	1	2	8	16.7789	211.4894
ASJ::caspase	1.5	1	2	9	11.1752	112.7016
ASJ::caspase	1.5	1	2	10	9.4629	173.3778
ASJ::caspase	1.5	1	2	11	6.1793	204.1086
ASJ::caspase	1.5	1	2	12	5.2617	237.3464
ASJ::caspase	1.5	1	2	13	5.4842	203.7767
ASJ::caspase	1.5	1	2	14	11.4857	144.3558
ASJ::caspase	1.5	1	3	1	10.5075	153.7557
ASJ::caspase	1.5	1	3	2	13.7616	239.6584
ASJ::caspase	1.5	1	3	3	14.6412	205.6972
ASJ::caspase	1.5	1	3	4	23.8346	228.2960
ASJ::caspase	1.5	1	3	5	23.8932	149.4701
ASJ::caspase	1.5	1	3	6	7.5114	188.3267
ASJ::caspase	1.5	1	3	7	12.7007	234.4633
ASJ::caspase	1.5	1	3	8	13.8826	109.7572
ASJ::caspase	1.5	1	3	9	25.7214	148.2233
ASJ::caspase	1.5	1	3	10	11.2868	123.4497
ASJ::caspase	1.5	1	3	11	19.7713	200.5849
ASJ::caspase	1.5	1	3	12	15.7393	161.8139
ASJ::caspase	1.5	1	3	13	41.8201	154.8120
ASJ::caspase	1.5	1	3	14	22.6148	98.3756
ASJ::caspase	1.5	1	3	15	97.7061	40.9698
ASJ::caspase	1.5	1	3	16	73.6027	49.4864
ASJ::caspase	1.5	1	4	1	103.2734	18.4393
ASJ::caspase	1.5	1	4	2	28.3281	253.0396
ASJ::caspase	1.5	1	4	3	21.8543	145.0969
ASJ::caspase	1.5	1	4	4	6.5935	207.0288

ASJ::caspase	1.5	1	4	5	29.9665	125.7676
ASJ::caspase	1.5	1	4	6	30.7252	219.8463
ASJ::caspase	1.5	1	4	7	16.5756	222.3080
ASJ::caspase	1.5	1	4	8	17.3905	129.3951
ASJ::caspase	1.5	1	4	9	11.7867	135.5830
ASJ::caspase	1.5	1	4	10	17.5294	85.8330
ASJ::caspase	1.5	1	4	11	10.9246	169.9096
ASJ::caspase	1.5	1	4	12	22.4450	170.6175
ASJ::caspase	1.5	1	4	13	35.6563	89.2142
ASJ::caspase	1.5	2	1	1	6.9633	278.7891
ASJ::caspase	1.5	2	1	2	8.4959	265.6501
ASJ::caspase	1.5	2	1	3	15.2367	267.3747
ASJ::caspase	1.5	2	1	4	9.0018	290.9762
ASJ::caspase	1.5	2	1	5	10.0679	325.0928
ASJ::caspase	1.5	2	1	6	6.3058	251.3593
ASJ::caspase	1.5	2	1	7	11.7711	345.9607
ASJ::caspase	1.5	2	1	8	56.8870	67.3575
ASJ::caspase	1.5	2	1	9	18.0614	251.4400
ASJ::caspase	1.5	2	1	10	10.5236	210.7050
ASJ::caspase	1.5	2	1	11	16.5595	299.3647
ASJ::caspase	1.5	2	1	12	84.9313	9.9769
ASJ::caspase	1.5	2	2	1	10.6662	218.2482
ASJ::caspase	1.5	2	2	2	11.4989	260.1955
ASJ::caspase	1.5	2	2	3	15.0711	317.8338
ASJ::caspase	1.5	2	2	4	6.5861	255.6194
ASJ::caspase	1.5	2	2	5	11.0156	210.2730
ASJ::caspase	1.5	2	2	6	14.1264	320.2485
ASJ::caspase	1.5	2	2	7	11.8594	256.9072
ASJ::caspase	1.5	2	2	8	15.4591	220.3357
ASJ::caspase	1.5	2	2	9	13.4931	315.7416
ASJ::caspase	1.5	2	2	10	9.0042	217.9087
ASJ::caspase	1.5	2	2	11	9.0467	182.8228
ASJ::caspase	1.5	2	2	12	12.5805	251.2884
ASJ::caspase	1.5	2	2	13	12.8709	246.6755
ASJ::caspase	1.5	2	3	1	17.5888	256.9041
ASJ::caspase	1.5	2	3	2	12.8643	280.2837
ASJ::caspase	1.5	2	3	3	11.4619	263.8178
ASJ::caspase	1.5	2	3	4	22.3194	251.4215
ASJ::caspase	1.5	2	3	5	20.7777	235.4471
ASJ::caspase	1.5	2	3	6	9.0709	234.2845
ASJ::caspase	1.5	2	3	7	23.8237	214.8730
ASJ::caspase	1.5	2	3	8	13.1141	264.8641
ASJ::caspase	1.5	2	3	9	8.9108	148.8562
ASJ::caspase	1.5	2	3	10	8.8068	203.6999
ASJ::caspase	1.5	2	3	11	9.0250	220.5459
ASJ::caspase	1.5	2	3	12	20.0478	300.8575
ASJ::caspase	1.5	2	4	1	10.6241	209.6333
ASJ::caspase	1.5	2	4	2	13.5055	247.5455
ASJ::caspase	1.5	2	4	3	10.2376	273.8696
ASJ::caspase	1.5	2	4	4	9.4513	190.1034
ASJ::caspase	1.5	2	4	5	15.2456	164.6039
ASJ::caspase	1.5	2	4	6	28.9418	169.1478
ASJ::caspase	1.5	2	4	7	19.4796	193.5324
ASJ::caspase	1.5	3	1	1	24.0068	262.4498
ASJ::caspase	1.5	3	1	2	17.4212	261.3298
ASJ::caspase	1.5	3	1	3	13.0673	289.7130
ASJ::caspase	1.5	3	1	4	16.0847	287.9133
ASJ::caspase	1.5	3	1	5	21.1485	279.1487
ASJ::caspase	1.5	3	1	6	48.3172	203.1729
ASJ::caspase	1.5	3	1	7	17.0006	311.4592
ASJ::caspase	1.5	3	2	1	6.9916	298.0387
ASJ::caspase	1.5	3	2	2	8.8538	274.5205
ASJ::caspase	1.5	3	2	3	6.0232	179.0550
ASJ::caspase	1.5	3	2	4	10.1712	305.2721
ASJ::caspase	1.5	3	2	5	8.1916	264.4507
ASJ::caspase	1.5	3	2	6	15.1721	292.8124
ASJ::caspase	1.5	3	2	7	10.4950	208.7304
ASJ::caspase	1.5	3	2	8	10.9234	308.3689

ASJ::caspase	1.5	3	2	9	10.2703	249.9271
ASJ::caspase	1.5	3	2	10	102.5613	9.3001
ASJ::caspase	1.5	3	2	11	24.5167	328.1661
ASJ::caspase	1.5	3	2	12	56.2088	171.2319
ASJ::caspase	1.5	3	2	13	6.8214	243.3098
ASJ::caspase	1.5	3	2	14	4.8523	278.3610
ASJ::caspase	1.5	3	2	15	8.7029	261.8967
ASJ::caspase	1.5	3	2	16	9.1364	191.7695
ASJ::caspase	1.5	3	3	1	7.6928	173.0442
ASJ::caspase	1.5	3	3	2	8.6624	258.6326
ASJ::caspase	1.5	3	3	3	10.0971	231.6039
ASJ::caspase	1.5	3	3	4	12.5072	247.8960
ASJ::caspase	1.5	3	3	5	19.3391	303.1952
ASJ::caspase	1.5	3	3	6	9.7643	296.9382
ASJ::caspase	1.5	3	3	7	7.2027	183.8716
ASJ::caspase	1.5	3	3	8	11.8636	255.0821
ASJ::caspase	1.5	3	3	9	6.1755	234.4729
ASJ::caspase	1.5	3	3	10	10.6071	272.3413
ASJ::caspase	1.5	3	3	11	16.6826	269.4581
ASJ::caspase	1.5	3	3	12	24.4255	214.4250
ASJ::caspase	1.5	3	3	13	7.0720	321.7558
ASJ::caspase	1.5	3	3	14	12.7816	254.3253
ASJ::caspase	1.5	3	3	15	14.6735	191.6563
ASJ::caspase	1.5	3	3	16	10.2200	286.6736
ASJ::caspase	1.5	3	3	17	10.6662	218.5371
ASJ::caspase	1.5	3	3	18	14.0043	178.0952
ASJ::caspase	1.5	3	4	1	14.6425	250.3364
ASJ::caspase	1.5	3	4	2	13.6664	186.3156
ASJ::caspase	1.5	3	4	3	38.5997	246.0765
ASJ::caspase	1.5	3	4	4	8.2824	180.4168
ASJ::caspase	1.5	3	4	5	19.7678	304.2650
ASJ::caspase	1.5	3	4	6	16.3317	230.5292
ASJ::caspase	1.5	3	4	7	8.9502	199.6712
ASJ::caspase	1.5	3	4	8	29.3978	152.3025
ASJ::caspase	1.5	3	4	9	7.8217	212.9597
ASJ::caspase	1.5	3	4	10	20.0535	218.8393
ASJ::caspase	1.5	3	4	11	25.9328	243.8161
ASJ::caspase	1.5	3	4	12	15.8893	234.0230
ASJ::caspase	3	1	1	1	26.9350	241.7331
ASJ::caspase	3	1	1	2	19.5678	302.4825
ASJ::caspase	3	1	1	3	17.1211	282.9129
ASJ::caspase	3	1	1	4	19.9275	251.9298
ASJ::caspase	3	1	1	5	17.4702	200.7137
ASJ::caspase	3	1	1	6	11.1096	286.3925
ASJ::caspase	3	1	1	7	23.5228	272.6130
ASJ::caspase	3	1	1	8	45.3541	136.7870
ASJ::caspase	3	1	1	9	9.4278	226.9202
ASJ::caspase	3	1	1	10	8.3340	219.6667
ASJ::caspase	3	1	1	11	17.0900	259.5577
ASJ::caspase	3	1	1	12	24.7912	273.4877
ASJ::caspase	3	1	1	13	28.0754	268.7166
ASJ::caspase	3	1	2	1	12.0373	229.9277
ASJ::caspase	3	1	2	2	13.1112	189.7122
ASJ::caspase	3	1	2	3	17.1200	243.0102
ASJ::caspase	3	1	2	4	24.1503	233.2194
ASJ::caspase	3	1	2	5	13.9591	249.7537
ASJ::caspase	3	1	2	6	17.4754	255.3524
ASJ::caspase	3	1	2	7	10.7449	216.0428
ASJ::caspase	3	1	2	8	22.5183	248.3931
ASJ::caspase	3	1	2	9	10.0517	262.7939
ASJ::caspase	3	1	2	10	19.5342	253.6451
ASJ::caspase	3	1	2	11	25.4045	256.5113
ASJ::caspase	3	1	2	12	10.2470	240.3225
ASJ::caspase	3	1	2	13	18.1112	253.4689
ASJ::caspase	3	1	2	14	9.3546	204.5562
ASJ::caspase	3	1	2	15	26.7452	265.7408
ASJ::caspase	3	1	3	1	16.9110	251.4231
ASJ::caspase	3	1	3	2	29.0281	288.3854

ASJ::caspase	3	1	3	3	29.7021	226.9566
ASJ::caspase	3	1	3	4	42.5364	234.2786
ASJ::caspase	3	1	3	5	23.3237	237.6680
ASJ::caspase	3	1	3	6	25.0023	241.4115
ASJ::caspase	3	1	3	7	7.4048	209.8409
ASJ::caspase	3	1	3	8	21.1153	230.3412
ASJ::caspase	3	1	3	9	15.1053	143.7398
ASJ::caspase	3	1	3	10	26.6225	219.2810
ASJ::caspase	3	2	1	1	16.1361	269.7149
ASJ::caspase	3	2	1	2	30.6335	259.0606
ASJ::caspase	3	2	1	3	13.1889	232.7631
ASJ::caspase	3	2	1	4	45.4912	175.9462
ASJ::caspase	3	2	1	5	18.8717	298.1813
ASJ::caspase	3	2	1	6	29.7842	275.6621
ASJ::caspase	3	2	1	7	53.2982	31.5837
ASJ::caspase	3	2	2	1	14.9521	287.9257
ASJ::caspase	3	2	2	2	13.2243	260.6431
ASJ::caspase	3	2	2	3	8.9399	207.3061
ASJ::caspase	3	2	2	4	11.9062	260.5518
ASJ::caspase	3	2	2	5	25.0936	220.7341
ASJ::caspase	3	2	2	6	11.9419	187.4537
ASJ::caspase	3	2	2	7	10.9744	242.6943
ASJ::caspase	3	2	2	8	31.4989	221.7307
ASJ::caspase	3	2	2	9	41.6352	276.5732
ASJ::caspase	3	2	2	10	26.4744	248.5901
ASJ::caspase	3	2	2	11	13.1736	232.2184
ASJ::caspase	3	2	3	1	12.8610	244.0833
ASJ::caspase	3	2	3	2	17.8029	249.4005
ASJ::caspase	3	2	3	3	14.2457	213.2170
ASJ::caspase	3	2	3	4	27.5160	300.9568
ASJ::caspase	3	2	3	5	23.0296	301.8353
ASJ::caspase	3	2	3	6	8.9363	207.2921
ASJ::caspase	3	2	3	7	35.5822	219.2282
ASJ::caspase	3	2	3	8	11.6774	233.1288
ASJ::caspase	3	2	3	9	36.3228	297.0468
ASJ::caspase	3	2	3	10	20.4247	226.9845
ASJ::caspase	3	2	3	11	48.0855	224.4398
ASJ::caspase	3	2	4	1	16.4866	211.4441
ASJ::caspase	3	2	4	2	44.3103	290.6821
ASJ::caspase	3	2	4	3	20.4412	230.6188
ASJ::caspase	3	2	4	4	10.9392	250.6968
ASJ::caspase	3	2	4	5	51.3392	264.7555
ASJ::caspase	3	2	4	6	15.8273	273.9450
ASJ::caspase	3	3	1	1	15.4628	137.4029
ASJ::caspase	3	3	1	2	19.6161	157.2593
ASJ::caspase	3	3	1	3	8.2213	146.1008
ASJ::caspase	3	3	1	4	19.1486	192.4665
ASJ::caspase	3	3	1	5	25.2277	213.3586
ASJ::caspase	3	3	1	6	17.3001	176.1688
ASJ::caspase	3	3	1	7	11.5513	218.9706
ASJ::caspase	3	3	1	8	18.0774	138.2717
ASJ::caspase	3	3	1	9	32.3781	207.2872
ASJ::caspase	3	3	1	10	29.1930	192.6246
ASJ::caspase	3	3	1	11	27.6545	124.2143
ASJ::caspase	3	3	1	12	17.4562	151.8235
ASJ::caspase	3	3	1	13	15.3666	208.4260
ASJ::caspase	3	3	1	14	19.7704	103.1268
ASJ::caspase	3	3	2	1	25.0302	181.9194
ASJ::caspase	3	3	2	2	16.6275	182.9672
ASJ::caspase	3	3	2	3	14.7664	73.4964
ASJ::caspase	3	3	2	4	17.3371	230.0148
ASJ::caspase	3	3	2	5	12.9705	131.8989
ASJ::caspase	3	3	2	6	24.6472	191.0442
ASJ::caspase	3	3	2	7	17.2174	161.6449
ASJ::caspase	3	3	2	8	15.8410	188.5404
ASJ::caspase	3	3	2	9	25.1815	122.7275
ASJ::caspase	3	3	2	10	31.9717	200.2940
ASJ::caspase	3	3	2	11	9.1847	161.6807

ASJ::caspase	3	3	2	12	19.0196	298.3678
ASJ::caspase	3	3	2	13	15.1286	139.5863
ASJ::caspase	3	3	2	14	19.2670	214.6753
ASJ::caspase	3	3	2	15	40.5890	56.9760
ASJ::caspase	3	3	3	1	27.5612	274.7812
ASJ::caspase	3	3	3	2	18.4972	148.9211
ASJ::caspase	3	3	3	3	14.9420	91.4813
ASJ::caspase	3	3	3	4	6.1780	131.3059
ASJ::caspase	3	3	3	5	19.7589	228.8634
ASJ::caspase	3	3	3	6	14.2611	175.8947
ASJ::caspase	3	3	3	7	18.8399	52.9517
ASJ::caspase	3	3	3	8	16.6855	145.2143
ASJ::caspase	3	3	3	9	16.3734	143.8917
ASJ::caspase	3	3	3	10	26.0757	135.2687
ASJ::caspase	3	3	3	11	16.2817	192.1264
ASJ::caspase	3	3	3	12	27.7824	117.8084
ASJ::caspase	3	3	3	13	12.3785	143.8586
ASJ::caspase	3	3	3	14	18.8425	96.8702
ASJ::caspase	3	3	3	15	15.0276	221.0495
ASJ::caspase	3	3	3	16	8.9061	131.6941
ASJ::caspase	3	3	4	1	40.0439	217.7404
ASJ::caspase	3	3	4	2	22.2910	202.9165
ASJ::caspase	3	3	4	3	12.8405	193.2929
ASJ::caspase	3	3	4	4	21.3583	202.6166
ASJ::caspase	3	3	4	5	10.0147	201.2078
ASJ::caspase	3	3	4	6	22.8708	151.1907
ASJ::caspase	3	3	4	7	23.6950	119.5767
ASJ::caspase	3	3	4	8	40.3800	150.8605
ASJ::caspase	3	3	4	9	16.5144	110.0661
ASJ::caspase	3	3	4	10	12.0261	102.3629
ASJ::caspase	3	3	4	11	54.1549	27.6596
ASJ::caspase	3	3	4	12	24.4710	125.6270
ASJ::caspase	3	3	4	13	13.6136	56.6465
ASJ::caspase	3	3	4	14	41.6517	142.6549
ASJ::caspase	3	3	4	15	22.4876	160.6625
ASJ::caspase	6	1	1	1	41.7333	245.1734
ASJ::caspase	6	1	1	2	21.6566	136.8016
ASJ::caspase	6	1	1	3	49.3295	239.7768
ASJ::caspase	6	1	1	4	27.4998	101.7397
ASJ::caspase	6	1	1	5	42.7235	239.9156
ASJ::caspase	6	1	1	6	48.8081	154.2476
ASJ::caspase	6	1	1	7	10.7909	177.5724
ASJ::caspase	6	1	1	8	28.0463	244.4584
ASJ::caspase	6	1	1	9	29.0594	88.1370
ASJ::caspase	6	1	1	10	51.7222	176.5363
ASJ::caspase	6	1	1	11	24.6614	121.8837
ASJ::caspase	6	1	1	12	53.2968	202.1692
ASJ::caspase	6	1	2	1	52.8645	137.6383
ASJ::caspase	6	1	2	2	59.1662	153.1634
ASJ::caspase	6	1	2	3	72.3852	6.6979
ASJ::caspase	6	1	2	4	48.5582	121.5180
ASJ::caspase	6	1	2	5	56.4482	206.7892
ASJ::caspase	6	1	2	6	40.3821	122.2902
ASJ::caspase	6	1	2	7	40.1147	166.4944
ASJ::caspase	6	1	3	1	28.3818	135.3544
ASJ::caspase	6	1	3	2	66.1996	52.0976
ASJ::caspase	6	1	3	3	86.7732	18.4131
ASJ::caspase	6	1	3	4	83.8765	70.1620
ASJ::caspase	6	1	3	5	80.1546	154.5767
ASJ::caspase	6	1	3	6	62.1756	138.4711
ASJ::caspase	6	1	3	7	96.5449	63.6188
ASJ::caspase	6	1	3	8	88.7125	161.8493
ASJ::caspase	6	1	3	9	73.4957	159.0432
ASJ::caspase	6	1	3	10	59.7762	92.9411
ASJ::caspase	6	1	3	11	79.7017	85.2513
ASJ::caspase	6	1	3	12	40.0212	131.8137
ASJ::caspase	6	1	3	13	46.6132	122.2628
ASJ::caspase	6	1	3	14	23.7500	156.4433

ASJ::caspase	6	1	3	15	34.7916 35.3024
ASJ::caspase	6	1	3	16	48.2360 183.7922
ASJ::caspase	6	1	4	1	55.5410 247.7905
ASJ::caspase	6	1	4	2	21.3949 144.2719
ASJ::caspase	6	1	4	3	31.6502 156.6217
ASJ::caspase	6	1	4	4	74.4067 68.9232
ASJ::caspase	6	1	4	5	86.9493 120.3639
ASJ::caspase	6	1	4	6	49.2751 234.4914
ASJ::caspase	6	1	4	7	87.6416 83.8395
ASJ::caspase	6	1	4	8	52.4037 69.4308
ASJ::caspase	6	1	4	9	58.3463 80.3963
ASJ::caspase	6	1	4	10	72.6173 24.2688
ASJ::caspase	6	1	4	11	64.6961 197.0906
ASJ::caspase	6	1	4	12	59.9688 91.6593
ASJ::caspase	6	1	4	13	97.8234 28.1067
ASJ::caspase	6	1	4	14	29.8638 101.2733
ASJ::caspase	6	1	4	15	96.2977 166.9496
ASJ::caspase	6	1	4	16	80.7093 59.1600
ASJ::caspase	6	1	4	17	79.0017 204.8871
ASJ::caspase	6	1	4	18	102.5796 260.2767
ASJ::caspase	6	1	4	19	62.4462 121.6169
ASJ::caspase	6	1	4	20	39.7997 41.6855
ASJ::caspase	6	1	4	21	67.5678 86.5525
ASJ::caspase	6	2	1	1	41.5140 218.0436
ASJ::caspase	6	2	1	2	37.7454 226.9848
ASJ::caspase	6	2	1	3	22.2359 223.8600
ASJ::caspase	6	2	1	4	64.3697 191.3338
ASJ::caspase	6	2	1	5	67.1113 236.0438
ASJ::caspase	6	2	1	6	35.6995 259.6772
ASJ::caspase	6	2	1	7	38.1315 233.2910
ASJ::caspase	6	2	1	8	79.8412 140.0180
ASJ::caspase	6	2	1	9	87.5767 34.7395
ASJ::caspase	6	2	1	10	14.3633 201.0231
ASJ::caspase	6	2	1	11	25.7807 232.5920
ASJ::caspase	6	2	1	12	23.4879 218.0833
ASJ::caspase	6	2	1	13	43.1856 220.5072
ASJ::caspase	6	2	2	1	26.4116 238.5355
ASJ::caspase	6	2	2	2	37.2052 113.8061
ASJ::caspase	6	2	2	3	20.0120 149.2551
ASJ::caspase	6	2	2	4	11.5936 170.3738
ASJ::caspase	6	2	2	5	16.2043 138.5932
ASJ::caspase	6	2	2	6	58.6978 130.9054
ASJ::caspase	6	2	2	7	36.0590 165.0388
ASJ::caspase	6	2	2	8	32.5259 174.7646
ASJ::caspase	6	2	2	9	29.8496 233.2084
ASJ::caspase	6	2	2	10	17.1511 177.7872
ASJ::caspase	6	2	2	11	27.1585 235.8207
ASJ::caspase	6	2	2	12	44.9951 212.0068
ASJ::caspase	6	2	2	13	95.9166 138.2081
ASJ::caspase	6	2	2	14	104.2716 3.1864
ASJ::caspase	6	2	2	15	24.7448 212.8311
ASJ::caspase	6	2	2	16	30.4581 173.1796
ASJ::caspase	6	2	2	17	46.7667 209.3239
ASJ::caspase	6	2	2	18	26.3959 239.8480
ASJ::caspase	6	2	2	19	33.1323 205.5073
ASJ::caspase	6	2	3	1	30.1984 263.7575
ASJ::caspase	6	2	3	2	32.6814 251.7577
ASJ::caspase	6	2	3	3	45.6926 250.5444
ASJ::caspase	6	2	3	4	49.1374 281.3588
ASJ::caspase	6	2	3	5	24.1891 154.2139
ASJ::caspase	6	2	3	6	47.6399 149.0905
ASJ::caspase	6	2	3	7	39.1353 129.2045
ASJ::caspase	6	2	3	8	25.5577 150.1941
ASJ::caspase	6	2	3	9	84.6223 36.8565
ASJ::caspase	6	2	3	10	66.1920 189.3197
ASJ::caspase	6	2	3	11	44.4917 157.8786
ASJ::caspase	6	2	3	12	83.7534 23.9569
ASJ::caspase	6	2	3	13	88.4940 91.9096

ASJ::caspase	6	2	4	1	29.0937	190.9086
ASJ::caspase	6	2	4	2	45.0677	196.9919
ASJ::caspase	6	2	4	3	75.7918	114.2974
ASJ::caspase	6	2	4	4	51.9520	36.7368
ASJ::caspase	6	2	4	5	46.5775	159.5624
ASJ::caspase	6	2	4	6	69.3568	19.5828
ASJ::caspase	6	2	4	7	69.9066	53.5090
ASJ::caspase	6	2	4	8	57.3015	47.1803
ASJ::caspase	6	3	1	1	30.8662	204.9346
ASJ::caspase	6	3	1	2	43.2497	149.9173
ASJ::caspase	6	3	1	3	54.3483	204.2105
ASJ::caspase	6	3	1	4	54.6465	192.1732
ASJ::caspase	6	3	1	5	18.3034	159.9351
ASJ::caspase	6	3	1	6	49.5640	174.0664
ASJ::caspase	6	3	1	7	42.6912	233.7997
ASJ::caspase	6	3	1	8	26.7218	223.0245
ASJ::caspase	6	3	1	9	36.5144	149.6691
ASJ::caspase	6	3	1	10	59.1681	236.3177
ASJ::caspase	6	3	2	1	26.7858	117.6494
ASJ::caspase	6	3	2	2	27.7333	177.1043
ASJ::caspase	6	3	2	3	42.7185	223.9510
ASJ::caspase	6	3	2	4	48.5336	137.5936
ASJ::caspase	6	3	2	5	32.4333	145.9007
ASJ::caspase	6	3	2	6	10.4728	272.6831
ASJ::caspase	6	3	2	7	65.1291	203.4365
ASJ::caspase	6	3	2	8	49.3729	127.9502
ASJ::caspase	6	3	2	9	40.6115	58.8313
ASJ::caspase	6	3	2	10	39.8592	187.8132
ASJ::caspase	6	3	2	11	50.0368	149.5556
ASJ::caspase	6	3	2	12	28.7023	203.2246
ASJ::caspase	6	3	2	13	51.8930	199.8147
ASJ::caspase	6	3	2	14	45.3351	157.3446
ASJ::caspase	6	3	2	15	45.1041	234.6665
ASJ::caspase	6	3	2	16	41.2955	168.3851
ASJ::caspase	6	3	2	17	44.7137	240.9728
ASJ::caspase	6	3	2	18	65.8430	144.1992
ASJ::caspase	6	3	2	19	39.9314	190.9382
ASJ::caspase	6	3	3	1	63.5081	149.1765
ASJ::caspase	6	3	3	2	47.2401	220.0582
ASJ::caspase	6	3	3	3	11.5567	176.4459
ASJ::caspase	6	3	3	4	20.7579	137.7271
ASJ::caspase	6	3	3	5	30.8241	142.4269
ASJ::caspase	6	3	3	6	32.2358	92.3307
ASJ::caspase	6	3	3	7	49.2787	247.0910
ASJ::caspase	6	3	3	8	43.6919	198.7250
ASJ::caspase	6	3	3	9	31.5573	145.9303
ASJ::caspase	6	3	3	10	43.2074	169.4243
ASJ::caspase	6	3	3	11	29.5704	242.4879
ASJ::caspase	6	3	3	12	19.8876	224.2912
ASJ::caspase	6	3	3	13	86.2839	5.2851
ASJ::caspase	6	3	3	14	46.7615	222.8634
ASJ::caspase	6	3	3	15	68.7962	187.2732
ASJ::caspase	6	3	3	16	36.0859	90.7201
ASJ::caspase	6	3	3	17	52.5192	241.0114
ASJ::caspase	6	3	3	18	43.6468	176.5492
ASJ::caspase	6	3	3	19	49.1228	270.4483
ASJ::caspase	6	3	4	1	27.1413	216.4038
ASJ::caspase	6	3	4	2	29.6849	63.4991
ASJ::caspase	6	3	4	3	19.4703	208.1220
ASJ::caspase	6	3	4	4	53.7728	155.0631
ASJ::caspase	6	3	4	5	60.1985	18.4098
ASJ::caspase	6	3	4	6	68.7284	191.6282
ASJ::caspase	6	3	4	7	35.9160	94.7980
ASJ::caspase	6	3	4	8	55.7013	183.9689
ASJ::caspase	6	3	4	9	85.7009	202.5013
ASJ::caspase	6	3	4	10	68.9744	213.4238
