# Supplementary material for: C. elegans Demonstrates Distinct Behaviors within a Fixed and Uniform Electric Field
Source: PLoS One. 2016 Mar 21;11(3):e0151320. doi: 10.1371/journal.pone.0151320 (PMC4801214; doi:10.1371/journal.pone.0151320)
Supplement: S2 Dataset — Tracks were scored (1) for pirouette and (0) no pirouette. (PDF) [file pone.0151320.s002.pdf]

strain	volt	rep	movie	track	pirouette
N2	0	1	1	1	1
N2	0	1	1	2	1
N2	0	1	1	3	1
N2	0	1	1	4	1
N2	0	1	1	5	1
N2	0	1	1	6	1
N2	0	1	1	7	1
N2	0	1	1	8	1
N2	0	1	1	9	1
N2	0	1	1	10	1
N2	0	1	1	11	1
N2	0	1	1	12	1
N2	0	1	1	13	1
N2	0	1	1	14	1
N2	0	1	2	1	1
N2	0	1	2	2	0
N2	0	1	2	3	0
N2	0	1	2	3	1
N2	0	1	2	4	1
N2	0	1	2	5	1
N2	0	1	2	6	0
N2	0	1	2	7	1
N2	0	1	2	8	1
N2	0	1	2	9	1
N2	0	1	2	10	1
N2	0	1	2	11	1
N2	0	1	2	12	1
N2	0	1	2	13	0
N2	0	1	3	1	1
N2	0	1	3	2	1
N2	0	1	3	3	1
N2	0	1	3	4	1
N2	0	1	3	5	1
N2	0	1	3	6	1
N2	0	1	3	7	1
N2	0	1	3	8	1
N2	0	1	3	9	0
N2	0	1	3	10	0
N2	0	1	3	11	1
N2	0	1	4	1	1
N2	0	1	4	2	1
N2	0	1	4	3	1
N2	0	1	4	4	1
N2	0	1	4	5	1
N2	0	1	4	6	0
N2	0	1	4	7	1
N2	0	1	4	8	1
N2	0	1	4	9	1

N2	0	1	4	10	1
N2	0	1	4	11	0
N2	0	1	4	12	1
N2	0	1	4	13	1
N2	0	1	4	14	0
N2	0	2	1	1	1
N2	0	2	1	2	1
N2	0	2	1	3	1
N2	0	2	1	4	1
N2	0	2	1	5	1
N2	0	2	1	6	1
N2	0	2	1	7	1
N2	0	2	1	8	1
N2	0	2	1	9	1
N2	0	2	1	10	1
N2	0	2	1	11	1
N2	0	2	1	12	1
N2	0	2	1	13	1
N2	0	2	1	14	1
N2	0	2	2	1	1
N2	0	2	2	2	1
N2	0	2	2	3	0
N2	0	2	2	4	1
N2	0	2	2	5	1
N2	0	2	2	6	1
N2	0	2	2	7	0
N2	0	2	2	8	1
N2	0	2	2	9	0
N2	0	2	2	10	1
N2	0	2	2	11	1
N2	0	2	3	1	1
N2	0	2	3	2	1
N2	0	2	3	3	1
N2	0	2	3	4	0
N2	0	2	3	5	1
N2	0	2	3	6	0
N2	0	2	4	1	0
N2	0	2	4	2	1
N2	0	2	4	3	0
N2	0	2	4	4	1
N2	0	2	4	5	0
N2	0	2	4	6	1
N2	0	2	4	7	0
N2	0	2	4	8	0
N2	0	2	4	9	1
N2	0	2	4	10	0
N2	0	2	4	11	1
N2	0	2	4	12	1
N2	0	2	4	13	1

N2	0	2	4	14	1
N2	0	2	4	15	0
N2	0	2	4	16	0
N2	0	2	4	17	0
N2	0	2	4	18	0
N2	0	3	1	1	1
N2	0	3	1	2	1
N2	0	3	1	3	1
N2	0	3	1	4	1
N2	0	3	1	5	1
N2	0	3	1	6	1
N2	0	3	1	7	1
N2	0	3	1	8	1
N2	0	3	1	9	1
N2	0	3	1	10	1
N2	0	3	1	11	1
N2	0	3	1	12	0
N2	0	3	1	13	1
N2	0	3	1	14	1
N2	0	3	1	15	1
N2	0	3	1	16	1
N2	0	3	1	17	0
N2	0	3	1	18	1
N2	0	3	1	19	1
N2	0	3	2	1	1
N2	0	3	2	2	1
N2	0	3	2	3	1
N2	0	3	2	4	1
N2	0	3	2	5	1
N2	0	3	2	6	1
N2	0	3	2	7	1
N2	0	3	2	8	1
N2	0	3	2	9	1
N2	0	3	2	10	1
N2	0	3	3	1	1
N2	0	3	3	2	0
N2	0	3	3	3	1
N2	0	3	3	4	1
N2	0	3	3	5	1
N2	0	3	3	6	1
N2	0	3	3	7	1
N2	0	3	3	8	1
N2	0	3	3	9	1
N2	0	3	3	10	1
N2	0	3	3	11	1
N2	0	3	3	12	0
N2	0	3	3	13	0
N2	0	3	3	14	1
N2	0	3	4	1	1

N2	0	3	4	2	1
N2	0	3	4	3	1
N2	0	3	4	4	1
N2	0	3	4	5	1
N2	0	3	4	6	1
N2	0	3	4	7	1
N2	0	3	4	8	1
N2	0	3	4	9	1
N2	0	3	4	10	1
N2	3	1	1	1	0
N2	3	1	1	2	0
N2	3	1	1	3	0
N2	3	1	1	4	0
N2	3	1	1	5	0
N2	3	1	1	6	0
N2	3	1	1	7	0
N2	3	1	1	8	0
N2	3	1	1	9	0
N2	3	1	1	10	0
N2	3	1	1	11	0
N2	3	1	1	12	0
N2	3	1	1	13	0
N2	3	1	2	1	0
N2	3	1	2	2	0
N2	3	1	2	3	0
N2	3	1	2	4	0
N2	3	1	2	5	0
N2	3	1	2	6	0
N2	3	1	2	7	0
N2	3	1	2	8	0
N2	3	1	2	9	0
N2	3	1	2	10	0
N2	3	1	2	11	0
N2	3	1	2	12	0
N2	3	1	2	13	0
N2	3	1	2	14	0
N2	3	1	2	15	0
N2	3	1	2	16	0
N2	3	1	2	17	0
N2	3	1	2	18	0
N2	3	1	2	19	0
N2	3	1	2	20	0
N2	3	1	3	1	0
N2	3	1	3	2	0
N2	3	1	3	3	0
N2	3	1	3	4	0
N2	3	1	3	5	0
N2	3	1	3	6	0
N2	3	1	3	7	0

N2	3	1	3	8	0
N2	3	1	3	9	0
N2	3	1	3	10	0
N2	3	1	3	11	0
N2	3	1	3	12	0
N2	3	1	3	13	0
N2	3	1	3	14	0
N2	3	1	3	15	0
N2	3	1	3	16	0
N2	3	1	3	17	0
N2	3	1	3	18	0
N2	3	1	3	19	0
N2	3	1	3	20	0
N2	3	1	3	21	0
N2	3	1	3	22	0
N2	3	1	3	23	0
N2	3	1	3	24	0
N2	3	1	3	25	0
N2	3	1	3	26	0
N2	3	1	3	27	0
N2	3	1	3	28	0
N2	3	1	3	29	0
N2	3	1	4	1	0
N2	3	1	4	2	0
N2	3	1	4	3	0
N2	3	1	4	4	0
N2	3	1	4	5	0
N2	3	1	4	6	0
N2	3	1	4	7	0
N2	3	1	4	8	0
N2	3	1	4	9	0
N2	3	1	4	10	0
N2	3	1	4	11	0
N2	3	1	4	12	0
N2	3	1	4	13	0
N2	3	1	4	14	0
N2	3	1	4	15	0
N2	3	1	4	16	0
N2	3	1	4	17	0
N2	3	1	4	18	0
N2	3	1	4	19	0
N2	3	1	4	20	0
N2	3	1	4	21	0
N2	3	1	4	22	1
N2	3	1	4	23	0
N2	3	2	1	1	0
N2	3	2	1	2	0
N2	3	2	1	3	0
N2	3	2	1	4	0

N2	3	2	1	5	1
N2	3	2	1	6	1
N2	3	2	1	7	0
N2	3	2	1	8	0
N2	3	2	1	9	0
N2	3	2	1	10	1
N2	3	2	1	11	0
N2	3	2	1	12	0
N2	3	2	1	13	0
N2	3	2	1	14	0
N2	3	2	1	15	0
N2	3	2	2	1	0
N2	3	2	2	2	0
N2	3	2	2	3	0
N2	3	2	2	4	0
N2	3	2	2	5	0
N2	3	2	2	6	0
N2	3	2	2	7	0
N2	3	2	2	8	0
N2	3	2	2	9	0
N2	3	2	2	10	0
N2	3	2	2	11	0
N2	3	2	2	12	0
N2	3	2	2	13	0
N2	3	2	2	14	0
N2	3	2	2	15	0
N2	3	2	2	16	0
N2	3	2	2	17	0
N2	3	2	2	18	0
N2	3	2	2	19	0
N2	3	2	2	20	0
N2	3	2	2	21	0
N2	3	2	2	22	0
N2	3	2	2	23	0
N2	3	2	2	24	0
N2	3	2	2	25	0
N2	3	2	2	26	0
N2	3	2	2	27	0
N2	3	2	2	28	0
N2	3	2	2	29	0
N2	3	2	2	30	0
N2	3	2	2	31	0
N2	3	2	2	32	0
N2	3	2	2	33	0
N2	3	2	2	34	0
N2	3	2	2	35	0
N2	3	2	2	36	0
N2	3	2	2	37	0
N2	3	2	3	1	0

N2	3	2	3	2	0
N2	3	2	3	3	0
N2	3	2	3	4	0
N2	3	2	3	5	0
N2	3	2	3	6	0
N2	3	2	3	7	0
N2	3	2	3	8	0
N2	3	2	3	9	0
N2	3	2	3	10	0
N2	3	2	3	11	0
N2	3	2	3	12	0
N2	3	2	3	13	0
N2	3	2	3	14	0
N2	3	2	3	15	0
N2	3	2	3	16	0
N2	3	2	3	17	0
N2	3	2	3	18	0
N2	3	2	3	19	0
N2	3	2	3	20	0
N2	3	2	3	21	0
N2	3	2	3	22	0
N2	3	2	3	23	0
N2	3	2	3	24	0
N2	3	2	3	25	0
N2	3	2	3	26	0
N2	3	2	3	27	0
N2	3	2	3	28	0
N2	3	2	3	29	0
N2	3	2	3	30	0
N2	3	2	3	31	0
N2	3	2	3	32	0
N2	3	2	3	33	0
N2	3	2	3	34	0
N2	3	2	3	35	0
N2	3	2	3	36	0
N2	3	2	3	37	0
N2	3	2	3	38	0
N2	3	2	3	39	0
N2	3	2	4	1	0
N2	3	2	4	2	0
N2	3	2	4	3	0
N2	3	2	4	4	0
N2	3	2	4	5	0
N2	3	2	4	6	0
N2	3	2	4	7	0
N2	3	2	4	8	0
N2	3	2	4	9	0
N2	3	2	4	10	0
N2	3	2	4	11	0

N2	3	2	4	12	0
N2	3	2	4	13	0
N2	3	2	4	14	0
N2	3	2	4	15	1
N2	3	2	4	16	0
N2	3	2	4	17	0
N2	3	2	4	18	0
N2	3	2	4	19	0
N2	3	2	4	20	0
N2	3	3	1	1	0
N2	3	3	1	2	0
N2	3	3	1	3	0
N2	3	3	1	4	0
N2	3	3	1	5	0
N2	3	3	1	6	0
N2	3	3	1	7	0
N2	3	3	1	8	1
N2	3	3	1	9	1
N2	3	3	1	10	1
N2	3	3	1	11	0
N2	3	3	1	12	0
N2	3	3	2	1	0
N2	3	3	2	2	0
N2	3	3	2	3	0
N2	3	3	2	4	0
N2	3	3	2	5	0
N2	3	3	2	6	0
N2	3	3	2	7	0
N2	3	3	2	8	0
N2	3	3	2	9	0
N2	3	3	2	10	0
N2	3	3	2	11	0
N2	3	3	2	12	0
N2	3	3	2	13	0
N2	3	3	2	14	0
N2	3	3	2	15	0
N2	3	3	2	16	0
N2	3	3	2	17	0
N2	3	3	2	18	0
N2	3	3	2	19	1
N2	3	3	2	20	0
N2	3	3	2	21	1
N2	3	3	2	22	0
N2	3	3	2	23	0
N2	3	3	2	24	0
N2	3	3	2	25	0
N2	3	3	2	26	0
N2	3	3	2	27	0
N2	3	3	2	28	0

N2	3	3	2	29	0
N2	3	3	2	30	0
N2	3	3	2	31	0
N2	3	3	2	32	0
N2	3	3	2	33	0
N2	3	3	2	34	0
N2	3	3	2	35	0
N2	3	3	3	1	0
N2	3	3	3	2	0
N2	3	3	3	3	0
N2	3	3	3	4	0
N2	3	3	3	5	0
N2	3	3	3	6	0
N2	3	3	3	7	1
N2	3	3	3	8	0
N2	3	3	3	9	0
N2	3	3	3	10	0
N2	3	3	3	11	0
N2	3	3	3	12	0
N2	3	3	3	13	0
N2	3	3	3	14	0
N2	3	3	3	15	0
N2	3	3	3	16	0
N2	3	3	3	17	0
N2	3	3	3	18	0
N2	3	3	3	19	0
N2	3	3	3	20	1
N2	3	3	4	1	0
N2	3	3	4	2	0
N2	3	3	4	3	0
N2	3	3	4	4	0
N2	3	3	4	5	0
N2	3	3	4	6	0
N2	3	3	4	7	0
N2	3	3	4	8	0
N2	3	3	4	9	0
N2	3	3	4	10	1
N2	3	3	4	11	0
N2	3	3	4	12	0
N2	3	3	4	13	0
N2	3	3	4	14	0
N2	3	3	4	15	0
N2	3	3	4	16	0
eat-4	0	1	1	1	0
eat-4	0	1	1	2	0
eat-4	0	1	1	3	1
eat-4	0	1	1	4	1

eat-4	0	1	1	5	0
eat-4	0	1	1	6	0
eat-4	0	1	1	7	1
eat-4	0	1	1	8	0
eat-4	0	1	1	9	0
eat-4	0	1	1	10	1
eat-4	0	1	1	11	0
eat-4	0	1	1	12	1
eat-4	0	1	1	13	0
eat-4	0	1	1	14	0
eat-4	0	1	1	15	0
eat-4	0	1	1	16	1
eat-4	0	1	1	17	0
eat-4	0	1	1	18	1
eat-4	0	1	1	19	0
eat-4	0	1	1	20	0
eat-4	0	1	1	21	0
eat-4	0	1	1	22	0
eat-4	0	1	1	23	0
eat-4	0	1	1	24	1
eat-4	0	1	1	25	1
eat-4	0	1	1	26	0
eat-4	0	1	1	27	0
eat-4	0	1	1	28	0
eat-4	0	1	1	29	0
eat-4	0	1	2	1	0
eat-4	0	1	2	2	0
eat-4	0	1	2	3	0
eat-4	0	1	2	4	1
eat-4	0	1	2	5	0
eat-4	0	1	2	6	0
eat-4	0	1	2	7	1
eat-4	0	1	2	8	1
eat-4	0	1	2	9	0
eat-4	0	1	2	10	1
eat-4	0	1	2	11	1
eat-4	0	1	2	12	0
eat-4	0	1	2	13	0
eat-4	0	1	2	14	0
eat-4	0	1	2	15	0
eat-4	0	1	2	16	0
eat-4	0	1	2	17	0
eat-4	0	1	2	18	0
eat-4	0	1	2	19	1
eat-4	0	1	2	20	0

eat-4	0	1	2	21	0
eat-4	0	1	2	22	0
eat-4	0	1	2	23	0
eat-4	0	1	2	24	0
eat-4	0	1	2	25	0
eat-4	0	1	2	26	1
eat-4	0	1	2	27	0
eat-4	0	1	2	28	1
eat-4	0	1	2	29	1
eat-4	0	1	2	30	1
eat-4	0	1	2	31	1
eat-4	0	1	2	32	1
eat-4	0	1	2	33	0
eat-4	0	1	2	34	1
eat-4	0	1	2	35	0
eat-4	0	1	2	36	1
eat-4	0	1	3	1	0
eat-4	0	1	3	2	0
eat-4	0	1	3	3	0
eat-4	0	1	3	4	0
eat-4	0	1	3	5	1
eat-4	0	1	3	6	0
eat-4	0	1	3	7	0
eat-4	0	1	3	8	1
eat-4	0	1	3	9	1
eat-4	0	1	3	10	1
eat-4	0	1	3	11	0
eat-4	0	1	3	12	1
eat-4	0	1	3	13	1
eat-4	0	1	3	14	0
eat-4	0	1	3	15	0
eat-4	0	1	3	16	1
eat-4	0	1	3	17	1
eat-4	0	1	3	18	1
eat-4	0	1	3	19	0
eat-4	0	1	3	20	0
eat-4	0	1	3	21	1
eat-4	0	1	3	22	1
eat-4	0	1	3	23	1
eat-4	0	1	3	24	1
eat-4	0	1	3	25	1
eat-4	0	1	3	26	1
eat-4	0	1	3	27	0
eat-4	0	1	3	28	0
eat-4	0	1	3	29	0

eat-4	0	1	3	30	0
eat-4	0	1	3	31	0
eat-4	0	1	3	32	1
eat-4	0	1	3	33	1
eat-4	0	1	3	34	0
eat-4	0	1	3	35	0
eat-4	0	1	3	36	1
eat-4	0	1	4	1	0
eat-4	0	1	4	2	1
eat-4	0	1	4	3	1
eat-4	0	1	4	4	0
eat-4	0	1	4	5	0
eat-4	0	1	4	6	1
eat-4	0	1	4	7	1
eat-4	0	1	4	8	0
eat-4	0	1	4	9	1
eat-4	0	1	4	10	1
eat-4	0	1	4	11	1
eat-4	0	1	4	12	1
eat-4	0	1	4	13	0
eat-4	0	1	4	14	1
eat-4	0	1	4	15	0
eat-4	0	1	4	16	1
eat-4	0	1	4	17	0
eat-4	0	1	4	18	1
eat-4	0	1	4	19	0
eat-4	0	1	4	20	0
eat-4	0	1	4	21	1
eat-4	0	1	4	22	0
eat-4	0	1	4	23	1
eat-4	0	1	4	24	0
eat-4	0	1	4	25	0
eat-4	0	1	4	26	1
eat-4	0	1	4	27	1
eat-4	0	1	4	28	0
eat-4	0	1	4	29	1
eat-4	0	1	4	30	1
eat-4	0	2	1	1	0
eat-4	0	2	1	2	0
eat-4	0	2	1	3	0
eat-4	0	2	1	4	0
eat-4	0	2	1	5	1
eat-4	0	2	1	6	0
eat-4	0	2	1	7	0
eat-4	0	2	1	8	0

eat-4	0	2	1	9	0
eat-4	0	2	1	10	0
eat-4	0	2	1	11	0
eat-4	0	2	1	12	0
eat-4	0	2	1	13	0
eat-4	0	2	1	14	1
eat-4	0	2	1	15	0
eat-4	0	2	1	16	0
eat-4	0	2	1	17	0
eat-4	0	2	1	18	0
eat-4	0	2	1	19	0
eat-4	0	2	1	20	0
eat-4	0	2	1	21	0
eat-4	0	2	1	22	0
eat-4	0	2	1	23	1
eat-4	0	2	1	24	0
eat-4	0	2	1	25	0
eat-4	0	2	1	26	1
eat-4	0	2	1	27	0
eat-4	0	2	1	28	1
eat-4	0	2	1	29	0
eat-4	0	2	1	30	0
eat-4	0	2	1	31	0
eat-4	0	2	1	32	0
eat-4	0	2	1	33	0
eat-4	0	2	1	34	0
eat-4	0	2	1	35	0
eat-4	0	2	1	36	0
eat-4	0	2	1	37	0
eat-4	0	2	1	38	1
eat-4	0	2	2	1	0
eat-4	0	2	2	2	0
eat-4	0	2	2	3	0
eat-4	0	2	2	4	0
eat-4	0	2	2	5	0
eat-4	0	2	2	6	0
eat-4	0	2	2	7	0
eat-4	0	2	2	8	0
eat-4	0	2	2	9	0
eat-4	0	2	2	10	0
eat-4	0	2	2	11	1
eat-4	0	2	2	12	1
eat-4	0	2	2	13	0
eat-4	0	2	2	14	1
eat-4	0	2	2	15	0

eat-4	0	2	2	16	0
eat-4	0	2	2	17	1
eat-4	0	2	2	18	0
eat-4	0	2	2	19	0
eat-4	0	2	2	20	0
eat-4	0	2	2	21	0
eat-4	0	2	2	22	0
eat-4	0	2	2	23	1
eat-4	0	2	2	24	0
eat-4	0	2	2	25	0
eat-4	0	2	2	26	0
eat-4	0	2	2	27	0
eat-4	0	2	2	28	1
eat-4	0	2	2	29	1
eat-4	0	2	2	30	1
eat-4	0	2	2	31	0
eat-4	0	2	2	32	0
eat-4	0	2	2	33	1
eat-4	0	2	2	34	0
eat-4	0	2	2	35	0
eat-4	0	2	2	36	1
eat-4	0	2	2	37	0
eat-4	0	2	2	38	0
eat-4	0	2	2	39	0
eat-4	0	2	2	40	0
eat-4	0	2	2	41	0
eat-4	0	2	2	42	0
eat-4	0	2	3	1	1
eat-4	0	2	3	2	1
eat-4	0	2	3	3	1
eat-4	0	2	3	4	1
eat-4	0	2	3	5	0
eat-4	0	2	3	6	0
eat-4	0	2	3	7	0
eat-4	0	2	3	8	1
eat-4	0	2	3	9	0
eat-4	0	2	3	10	0
eat-4	0	2	3	11	1
eat-4	0	2	3	12	1
eat-4	0	2	3	13	0
eat-4	0	2	3	14	0
eat-4	0	2	3	15	1
eat-4	0	2	3	16	1
eat-4	0	2	3	17	0
eat-4	0	2	3	18	0

eat-4	0	2	3	19	0
eat-4	0	2	3	20	0
eat-4	0	2	3	21	0
eat-4	0	2	3	22	0
eat-4	0	2	3	23	0
eat-4	0	2	3	24	0
eat-4	0	2	3	25	0
eat-4	0	2	3	26	0
eat-4	0	2	3	27	0
eat-4	0	2	3	28	0
eat-4	0	2	3	29	1
eat-4	0	2	3	30	1
eat-4	0	2	3	31	0
eat-4	0	2	3	32	0
eat-4	0	2	3	33	0
eat-4	0	2	3	34	0
eat-4	0	2	3	35	1
eat-4	0	2	3	36	0
eat-4	0	2	3	37	0
eat-4	0	2	3	38	0
eat-4	0	2	4	1	1
eat-4	0	2	4	2	0
eat-4	0	2	4	3	1
eat-4	0	2	4	4	0
eat-4	0	2	4	5	1
eat-4	0	2	4	6	1
eat-4	0	2	4	7	1
eat-4	0	2	4	8	0
eat-4	0	2	4	9	0
eat-4	0	2	4	10	0
eat-4	0	2	4	11	0
eat-4	0	2	4	12	1
eat-4	0	2	4	13	0
eat-4	0	2	4	14	0
eat-4	0	2	4	15	0
eat-4	0	2	4	16	1
eat-4	0	2	4	17	0
eat-4	0	2	4	18	0
eat-4	0	2	4	19	1
eat-4	0	2	4	20	1
eat-4	0	2	4	21	0
eat-4	0	2	4	22	1
eat-4	0	2	4	23	1
eat-4	0	2	4	24	0
eat-4	0	2	4	25	0

eat-4	0	2	4	26	1
eat-4	0	2	4	27	0
eat-4	0	2	4	28	0
eat-4	0	2	4	29	1
eat-4	0	2	4	30	1
eat-4	0	2	4	31	1
eat-4	0	2	4	32	0
eat-4	0	2	4	33	0
eat-4	0	2	4	34	0
eat-4	0	2	4	35	0
eat-4	0	2	4	36	0
eat-4	0	3	1	1	1
eat-4	0	3	1	2	1
eat-4	0	3	1	3	0
eat-4	0	3	1	4	0
eat-4	0	3	1	5	0
eat-4	0	3	1	6	0
eat-4	0	3	1	7	1
eat-4	0	3	1	8	1
eat-4	0	3	1	9	0
eat-4	0	3	1	10	1
eat-4	0	3	1	11	1
eat-4	0	3	1	12	1
eat-4	0	3	1	13	0
eat-4	0	3	1	14	1
eat-4	0	3	1	15	1
eat-4	0	3	1	16	1
eat-4	0	3	1	17	0
eat-4	0	3	1	18	0
eat-4	0	3	1	19	1
eat-4	0	3	1	20	0
eat-4	0	3	2	1	1
eat-4	0	3	2	2	1
eat-4	0	3	2	3	1
eat-4	0	3	2	4	1
eat-4	0	3	2	5	1
eat-4	0	3	2	6	1
eat-4	0	3	2	7	1
eat-4	0	3	2	8	1
eat-4	0	3	2	9	0
eat-4	0	3	2	10	0
eat-4	0	3	2	11	1
eat-4	0	3	2	12	1
eat-4	0	3	2	13	1
eat-4	0	3	2	14	1

eat-4	0	3	2	15	1
eat-4	0	3	2	16	0
eat-4	0	3	2	17	0
eat-4	0	3	2	18	0
eat-4	0	3	2	19	1
eat-4	0	3	2	20	1
eat-4	0	3	2	21	1
eat-4	0	3	2	22	0
eat-4	0	3	2	23	1
eat-4	0	3	2	24	1
eat-4	0	3	2	25	0
eat-4	0	3	2	26	1
eat-4	0	3	2	27	1
eat-4	0	3	2	28	0
eat-4	0	3	2	29	0
eat-4	0	3	2	30	0
eat-4	0	3	2	31	1
eat-4	0	3	2	32	0
eat-4	0	3	2	33	1
eat-4	0	3	3	1	1
eat-4	0	3	3	2	1
eat-4	0	3	3	3	1
eat-4	0	3	3	4	0
eat-4	0	3	3	5	0
eat-4	0	3	3	6	1
eat-4	0	3	3	7	1
eat-4	0	3	3	8	1
eat-4	0	3	3	9	0
eat-4	0	3	3	10	1
eat-4	0	3	3	11	1
eat-4	0	3	3	12	1
eat-4	0	3	3	13	1
eat-4	0	3	3	14	1
eat-4	0	3	3	15	0
eat-4	0	3	3	16	1
eat-4	0	3	3	17	1
eat-4	0	3	3	18	1
eat-4	0	3	3	19	0
eat-4	0	3	3	20	1
eat-4	0	3	3	21	1
eat-4	0	3	3	22	1
eat-4	0	3	3	23	0
eat-4	0	3	3	24	0
eat-4	0	3	3	25	1
eat-4	0	3	3	26	1

eat-4	0	3	3	27	0
eat-4	0	3	3	28	0
eat-4	0	3	3	29	0
eat-4	0	3	4	1	1
eat-4	0	3	4	2	1
eat-4	0	3	4	3	0
eat-4	0	3	4	4	1
eat-4	0	3	4	5	1
eat-4	0	3	4	6	0
eat-4	0	3	4	7	0
eat-4	0	3	4	8	1
eat-4	0	3	4	9	1
eat-4	0	3	4	10	1
eat-4	0	3	4	11	0
eat-4	0	3	4	12	1
eat-4	0	3	4	13	0
eat-4	0	3	4	14	1
eat-4	0	3	4	15	0
eat-4	0	3	4	16	0
eat-4	0	3	4	17	0
eat-4	0	3	4	18	1
eat-4	0	3	4	19	0
eat-4	0	3	4	20	0
eat-4	0	3	4	21	1
eat-4	0	3	4	22	0
eat-4	0	3	4	23	1
eat-4	0	3	4	24	1
eat-4	0	3	4	25	1
eat-4	0	3	4	26	0
eat-4	0	3	4	27	1
eat-4	0	3	4	28	1
eat-4	0	3	4	29	1
eat-4	0	3	4	30	0
eat-4	0	3	4	31	0
eat-4	0	3	4	32	1
eat-4	0	3	4	33	0
eat-4	0	3	4	34	0
eat-4	0	3	4	35	0
eat-4	0	3	4	36	0
eat-4	0	3	4	37	0
eat-4	0	3	4	38	1
eat-4	0	3	4	39	0
eat-4	3	1	1	1	0
eat-4	3	1	1	2	1
eat-4	3	1	1	3	0

eat-4	3	1	1	4	0
eat-4	3	1	1	5	1
eat-4	3	1	1	6	1
eat-4	3	1	1	7	1
eat-4	3	1	1	8	1
eat-4	3	1	1	9	1
eat-4	3	1	1	10	1
eat-4	3	1	1	11	1
eat-4	3	1	1	12	1
eat-4	3	1	1	13	0
eat-4	3	1	1	14	0
eat-4	3	1	1	15	0
eat-4	3	1	2	1	0
eat-4	3	1	2	2	0
eat-4	3	1	2	3	0
eat-4	3	1	2	4	0
eat-4	3	1	2	5	0
eat-4	3	1	2	6	1
eat-4	3	1	2	7	1
eat-4	3	1	2	8	1
eat-4	3	1	2	9	1
eat-4	3	1	2	10	1
eat-4	3	1	2	11	1
eat-4	3	1	2	12	1
eat-4	3	1	2	13	1
eat-4	3	1	2	14	1
eat-4	3	1	2	15	1
eat-4	3	1	2	16	1
eat-4	3	1	2	17	1
eat-4	3	1	2	18	0
eat-4	3	1	2	19	1
eat-4	3	1	2	20	1
eat-4	3	1	2	21	1
eat-4	3	1	2	22	0
eat-4	3	1	2	23	1
eat-4	3	1	2	24	1
eat-4	3	1	2	25	1
eat-4	3	1	2	26	0
eat-4	3	1	3	1	1
eat-4	3	1	3	2	1
eat-4	3	1	3	3	1
eat-4	3	1	3	4	1
eat-4	3	1	3	5	0
eat-4	3	1	3	6	1
eat-4	3	1	3	7	0

eat-4	3	1	3	8	0
eat-4	3	1	3	9	0
eat-4	3	1	3	10	1
eat-4	3	1	3	11	1
eat-4	3	1	3	12	1
eat-4	3	1	3	13	1
eat-4	3	1	3	14	1
eat-4	3	1	3	15	1
eat-4	3	1	3	16	1
eat-4	3	1	3	17	0
eat-4	3	1	3	18	1
eat-4	3	1	3	19	1
eat-4	3	1	3	20	0
eat-4	3	1	3	21	1
eat-4	3	1	3	22	1
eat-4	3	1	3	23	0
eat-4	3	1	4	1	0
eat-4	3	1	4	2	1
eat-4	3	1	4	3	1
eat-4	3	1	4	4	0
eat-4	3	1	4	5	0
eat-4	3	1	4	6	1
eat-4	3	1	4	7	1
eat-4	3	1	4	8	0
eat-4	3	1	4	9	1
eat-4	3	1	4	10	1
eat-4	3	1	4	11	1
eat-4	3	1	4	12	0
eat-4	3	1	4	13	1
eat-4	3	1	4	14	1
eat-4	3	1	4	15	1
eat-4	3	1	4	16	0
eat-4	3	1	4	17	0
eat-4	3	1	4	18	0
eat-4	3	1	4	19	1
eat-4	3	1	4	20	1
eat-4	3	1	4	21	1
eat-4	3	1	4	22	1
eat-4	3	1	4	23	1
eat-4	3	1	4	24	1
eat-4	3	1	4	25	1
eat-4	3	2	1	1	0
eat-4	3	2	1	2	0
eat-4	3	2	1	3	1
eat-4	3	2	1	4	0

eat-4	3	2	1	5	0
eat-4	3	2	1	6	0
eat-4	3	2	1	7	1
eat-4	3	2	1	8	0
eat-4	3	2	1	9	1
eat-4	3	2	1	10	1
eat-4	3	2	1	11	0
eat-4	3	2	1	12	1
eat-4	3	2	1	13	0
eat-4	3	2	1	14	0
eat-4	3	2	1	15	0
eat-4	3	2	1	16	0
eat-4	3	2	1	17	1
eat-4	3	2	1	18	1
eat-4	3	2	1	19	1
eat-4	3	2	1	20	0
eat-4	3	2	1	21	1
eat-4	3	2	1	22	0
eat-4	3	2	1	23	0
eat-4	3	2	1	24	0
eat-4	3	2	1	25	0
eat-4	3	2	1	26	1
eat-4	3	2	1	27	1
eat-4	3	2	1	28	1
eat-4	3	2	1	29	0
eat-4	3	2	1	30	0
eat-4	3	2	1	31	0
eat-4	3	2	1	32	1
eat-4	3	2	2	1	0
eat-4	3	2	2	2	0
eat-4	3	2	2	3	0
eat-4	3	2	2	4	1
eat-4	3	2	2	5	1
eat-4	3	2	2	6	1
eat-4	3	2	2	7	0
eat-4	3	2	2	8	0
eat-4	3	2	2	9	0
eat-4	3	2	2	10	1
eat-4	3	2	2	11	1
eat-4	3	2	2	12	1
eat-4	3	2	2	13	0
eat-4	3	2	2	14	0
eat-4	3	2	2	15	0
eat-4	3	2	2	16	1
eat-4	3	2	2	17	1

eat-4	3	2	2	18	1
eat-4	3	2	2	19	0
eat-4	3	2	2	20	0
eat-4	3	2	2	21	1
eat-4	3	2	2	22	1
eat-4	3	2	2	23	1
eat-4	3	2	2	24	0
eat-4	3	2	2	25	1
eat-4	3	2	2	26	1
eat-4	3	2	2	27	1
eat-4	3	2	2	28	0
eat-4	3	2	2	29	1
eat-4	3	2	3	1	0
eat-4	3	2	3	2	1
eat-4	3	2	3	3	0
eat-4	3	2	3	4	1
eat-4	3	2	3	5	0
eat-4	3	2	3	6	1
eat-4	3	2	3	7	0
eat-4	3	2	3	8	1
eat-4	3	2	3	9	0
eat-4	3	2	3	10	1
eat-4	3	2	3	11	1
eat-4	3	2	3	12	0
eat-4	3	2	3	13	1
eat-4	3	2	3	14	1
eat-4	3	2	3	15	1
eat-4	3	2	3	16	1
eat-4	3	2	3	17	1
eat-4	3	2	3	18	1
eat-4	3	2	3	19	1
eat-4	3	2	3	20	0
eat-4	3	2	3	21	0
eat-4	3	2	3	22	1
eat-4	3	2	3	23	0
eat-4	3	2	3	24	1
eat-4	3	2	3	25	0
eat-4	3	2	3	26	0
eat-4	3	2	3	27	0
eat-4	3	2	3	28	1
eat-4	3	2	3	29	0
eat-4	3	2	3	30	1
eat-4	3	2	4	1	0
eat-4	3	2	4	2	1
eat-4	3	2	4	3	1

eat-4	3	2	4	4	0
eat-4	3	2	4	5	0
eat-4	3	2	4	6	0
eat-4	3	2	4	7	1
eat-4	3	2	4	8	1
eat-4	3	2	4	9	0
eat-4	3	2	4	10	0
eat-4	3	2	4	11	0
eat-4	3	2	4	12	0
eat-4	3	2	4	13	0
eat-4	3	2	4	14	0
eat-4	3	2	4	15	0
eat-4	3	2	4	16	1
eat-4	3	2	4	17	1
eat-4	3	2	4	18	1
eat-4	3	2	4	19	0
eat-4	3	2	4	20	0
eat-4	3	2	4	21	0
eat-4	3	2	4	22	0
eat-4	3	2	4	23	1
eat-4	3	2	4	24	0
eat-4	3	2	4	25	1
eat-4	3	2	4	26	0
eat-4	3	2	4	27	0
eat-4	3	2	4	28	0
eat-4	3	2	4	29	0
eat-4	3	3	1	1	0
eat-4	3	3	1	2	0
eat-4	3	3	1	3	0
eat-4	3	3	1	4	0
eat-4	3	3	1	5	0
eat-4	3	3	1	6	0
eat-4	3	3	1	7	0
eat-4	3	3	1	8	0
eat-4	3	3	1	9	1
eat-4	3	3	1	10	1
eat-4	3	3	1	11	0
eat-4	3	3	1	12	0
eat-4	3	3	1	13	1
eat-4	3	3	1	14	0
eat-4	3	3	1	15	0
eat-4	3	3	1	16	0
eat-4	3	3	1	17	0
eat-4	3	3	1	18	1
eat-4	3	3	1	19	1

eat-4	3	3	1	20	0
eat-4	3	3	1	21	0
eat-4	3	3	2	1	1
eat-4	3	3	2	2	0
eat-4	3	3	2	3	0
eat-4	3	3	2	4	0
eat-4	3	3	2	5	0
eat-4	3	3	2	6	1
eat-4	3	3	2	7	1
eat-4	3	3	2	8	0
eat-4	3	3	2	9	0
eat-4	3	3	2	10	0
eat-4	3	3	2	11	0
eat-4	3	3	2	12	1
eat-4	3	3	2	13	0
eat-4	3	3	2	14	0
eat-4	3	3	2	15	1
eat-4	3	3	2	16	0
eat-4	3	3	2	17	0
eat-4	3	3	2	18	0
eat-4	3	3	2	19	0
eat-4	3	3	2	20	0
eat-4	3	3	3	1	0
eat-4	3	3	3	2	0
eat-4	3	3	3	3	1
eat-4	3	3	3	4	0
eat-4	3	3	3	5	1
eat-4	3	3	3	6	1
eat-4	3	3	3	7	1
eat-4	3	3	3	8	1
eat-4	3	3	3	9	1
eat-4	3	3	3	10	0
eat-4	3	3	3	11	1
eat-4	3	3	3	12	1
eat-4	3	3	3	13	1
eat-4	3	3	3	14	0
eat-4	3	3	3	15	1
eat-4	3	3	3	16	0
eat-4	3	3	3	17	0
eat-4	3	3	3	18	1
eat-4	3	3	3	19	1
eat-4	3	3	3	20	1
eat-4	3	3	3	21	0
eat-4	3	3	3	22	0
eat-4	3	3	3	23	0

eat-4	3	3	3	24	1
eat-4	3	3	3	25	1
eat-4	3	3	3	26	0
eat-4	3	3	3	27	1
eat-4	3	3	3	28	1
eat-4	3	3	3	29	1
eat-4	3	3	4	1	1
eat-4	3	3	4	2	0
eat-4	3	3	4	3	0
eat-4	3	3	4	4	1
eat-4	3	3	4	5	0
eat-4	3	3	4	6	0
eat-4	3	3	4	7	0
eat-4	3	3	4	8	1
eat-4	3	3	4	9	1
eat-4	3	3	4	10	1
eat-4	3	3	4	11	1
eat-4	3	3	4	12	0
eat-4	3	3	4	13	0
eat-4	3	3	4	14	0
eat-4	3	3	4	15	0
eat-4	3	3	4	16	0
eat-4	3	3	4	17	0
eat-4	3	3	4	18	1
eat-4	3	3	4	19	1
eat-4	3	3	4	20	0
eat-4	3	3	4	21	1
eat-4	3	3	4	22	1
eat-4	3	3	4	23	0
eat-4	3	3	4	24	1
eat-4	3	3	4	25	0
eat-4	3	3	4	26	1
eat-4	3	3	4	27	0
eat-4	3	3	4	28	1
eat-4	3	3	4	29	0
eat-4	3	3	4	30	1
eat-4	3	3	4	31	0
eat-4	3	3	4	32	1
eat-4	3	3	4	33	1
eat-4	3	3	4	34	1
eat-4	3	3	4	35	0
eat-4	3	3	4	36	0
eat-4	3	3	4	37	1
eat-4	3	3	4	38	0
eat-4	3	3	4	39	0

AWC::eat-4	0	1	1	1	1
AWC::eat-4	0	1	1	2	1
AWC::eat-4	0	1	1	3	0
AWC::eat-4	0	1	1	4	1
AWC::eat-4	0	1	1	5	1
AWC::eat-4	0	1	1	6	0
AWC::eat-4	0	1	1	7	1
AWC::eat-4	0	1	1	8	1
AWC::eat-4	0	1	1	9	0
AWC::eat-4	0	1	1	10	1
AWC::eat-4	0	1	1	11	0
AWC::eat-4	0	1	1	12	1
AWC::eat-4	0	1	1	13	1
AWC::eat-4	0	1	1	14	1
AWC::eat-4	0	1	1	15	0
AWC::eat-4	0	1	1	16	0
AWC::eat-4	0	1	1	17	1
AWC::eat-4	0	1	2	1	1
AWC::eat-4	0	1	2	2	1
AWC::eat-4	0	1	2	3	1
AWC::eat-4	0	1	2	4	0
AWC::eat-4	0	1	2	5	1
AWC::eat-4	0	1	2	6	1
AWC::eat-4	0	1	2	7	1
AWC::eat-4	0	1	2	8	0
AWC::eat-4	0	1	2	9	1
AWC::eat-4	0	1	2	10	1
AWC::eat-4	0	1	2	11	1
AWC::eat-4	0	1	2	12	1
AWC::eat-4	0	1	2	13	0
AWC::eat-4	0	1	2	14	0
AWC::eat-4	0	1	2	15	1
AWC::eat-4	0	1	2	16	0
AWC::eat-4	0	1	2	17	1
AWC::eat-4	0	1	2	18	1
AWC::eat-4	0	1	2	19	0
AWC::eat-4	0	1	2	20	1
AWC::eat-4	0	1	3	1	0
AWC::eat-4	0	1	3	2	0
AWC::eat-4	0	1	3	3	1
AWC::eat-4	0	1	3	4	1
AWC::eat-4	0	1	3	5	1
AWC::eat-4	0	1	3	6	1
AWC::eat-4	0	1	3	7	1

AWC::eat-4	0	1	3	8	0
AWC::eat-4	0	1	3	9	1
AWC::eat-4	0	1	3	10	1
AWC::eat-4	0	1	3	11	1
AWC::eat-4	0	1	3	12	1
AWC::eat-4	0	1	3	13	1
AWC::eat-4	0	1	3	14	0
AWC::eat-4	0	1	3	15	1
AWC::eat-4	0	1	3	16	1
AWC::eat-4	0	1	3	17	0
AWC::eat-4	0	1	3	18	1
AWC::eat-4	0	1	3	19	1
AWC::eat-4	0	1	3	20	0
AWC::eat-4	0	1	3	21	1
AWC::eat-4	0	1	3	22	1
AWC::eat-4	0	1	4	1	1
AWC::eat-4	0	1	4	2	1
AWC::eat-4	0	1	4	3	0
AWC::eat-4	0	1	4	4	0
AWC::eat-4	0	1	4	5	1
AWC::eat-4	0	1	4	6	0
AWC::eat-4	0	1	4	7	1
AWC::eat-4	0	1	4	8	0
AWC::eat-4	0	1	4	9	1
AWC::eat-4	0	1	4	10	1
AWC::eat-4	0	1	4	11	0
AWC::eat-4	0	1	4	12	1
AWC::eat-4	0	1	4	13	1
AWC::eat-4	0	1	4	14	1
AWC::eat-4	0	1	4	15	1
AWC::eat-4	0	1	4	16	1
AWC::eat-4	0	1	4	17	0
AWC::eat-4	0	1	4	18	0
AWC::eat-4	0	1	4	19	1
AWC::eat-4	0	1	4	20	1
AWC::eat-4	0	2	1	1	1
AWC::eat-4	0	2	1	2	1
AWC::eat-4	0	2	1	3	1
AWC::eat-4	0	2	1	4	1
AWC::eat-4	0	2	1	5	1
AWC::eat-4	0	2	1	6	1
AWC::eat-4	0	2	1	7	1
AWC::eat-4	0	2	1	8	1
AWC::eat-4	0	2	1	9	1
AWC::eat-4	0	2	1	10	1

AWC::eat-4	0	2	2	1	1
AWC::eat-4	0	2	2	2	1
AWC::eat-4	0	2	2	3	1
AWC::eat-4	0	2	2	4	1
AWC::eat-4	0	2	2	5	1
AWC::eat-4	0	2	2	6	1
AWC::eat-4	0	2	2	7	1
AWC::eat-4	0	2	2	8	1
AWC::eat-4	0	2	2	9	1
AWC::eat-4	0	2	2	10	1
AWC::eat-4	0	2	2	11	1
AWC::eat-4	0	2	2	12	1
AWC::eat-4	0	2	2	13	1
AWC::eat-4	0	2	2	14	1
AWC::eat-4	0	2	2	15	1
AWC::eat-4	0	2	2	16	1
AWC::eat-4	0	2	2	17	1
AWC::eat-4	0	2	2	18	1
AWC::eat-4	0	2	2	19	1
AWC::eat-4	0	2	2	20	1
AWC::eat-4	0	2	2	21	1
AWC::eat-4	0	2	2	22	1
AWC::eat-4	0	2	2	23	1
AWC::eat-4	0	2	2	24	1
AWC::eat-4	0	2	2	25	1
AWC::eat-4	0	2	2	26	1
AWC::eat-4	0	2	3	1	1
AWC::eat-4	0	2	3	2	1
AWC::eat-4	0	2	3	3	1
AWC::eat-4	0	2	3	4	1
AWC::eat-4	0	2	3	5	0
AWC::eat-4	0	2	3	6	1
AWC::eat-4	0	2	3	7	1
AWC::eat-4	0	2	3	8	0
AWC::eat-4	0	2	3	9	1
AWC::eat-4	0	2	3	10	1
AWC::eat-4	0	2	3	11	0
AWC::eat-4	0	2	3	12	1
AWC::eat-4	0	2	3	13	1
AWC::eat-4	0	2	3	14	1
AWC::eat-4	0	2	3	15	1
AWC::eat-4	0	2	3	16	1
AWC::eat-4	0	2	3	17	1
AWC::eat-4	0	2	3	18	0
AWC::eat-4	0	2	3	19	1

AWC::eat-4	0	2	3	20	0
AWC::eat-4	0	2	3	21	0
AWC::eat-4	0	2	4	1	1
AWC::eat-4	0	2	4	2	0
AWC::eat-4	0	2	4	3	1
AWC::eat-4	0	2	4	4	1
AWC::eat-4	0	2	4	5	0
AWC::eat-4	0	2	4	6	0
AWC::eat-4	0	2	4	7	1
AWC::eat-4	0	2	4	8	1
AWC::eat-4	0	2	4	9	1
AWC::eat-4	0	2	4	10	1
AWC::eat-4	0	2	4	11	0
AWC::eat-4	0	2	4	12	1
AWC::eat-4	0	2	4	13	1
AWC::eat-4	0	2	4	14	1
AWC::eat-4	0	2	4	15	0
AWC::eat-4	0	2	4	16	0
AWC::eat-4	0	2	4	17	0
AWC::eat-4	0	3	1	1	0
AWC::eat-4	0	3	1	2	1
AWC::eat-4	0	3	1	3	1
AWC::eat-4	0	3	1	4	1
AWC::eat-4	0	3	1	5	1
AWC::eat-4	0	3	1	6	1
AWC::eat-4	0	3	1	7	1
AWC::eat-4	0	3	1	8	1
AWC::eat-4	0	3	1	9	0
AWC::eat-4	0	3	1	10	1
AWC::eat-4	0	3	1	11	1
AWC::eat-4	0	3	1	12	0
AWC::eat-4	0	3	1	13	1
AWC::eat-4	0	3	2	1	0
AWC::eat-4	0	3	2	2	1
AWC::eat-4	0	3	2	3	1
AWC::eat-4	0	3	2	4	1
AWC::eat-4	0	3	2	5	1
AWC::eat-4	0	3	2	6	1
AWC::eat-4	0	3	2	7	1
AWC::eat-4	0	3	2	8	1
AWC::eat-4	0	3	2	9	1
AWC::eat-4	0	3	2	10	1
AWC::eat-4	0	3	2	11	1
AWC::eat-4	0	3	2	12	1
AWC::eat-4	0	3	2	13	1

AWC::eat-4	0	3	3	1	0
AWC::eat-4	0	3	3	2	1
AWC::eat-4	0	3	3	3	1
AWC::eat-4	0	3	3	4	1
AWC::eat-4	0	3	3	5	0
AWC::eat-4	0	3	3	6	1
AWC::eat-4	0	3	3	7	1
AWC::eat-4	0	3	3	8	1
AWC::eat-4	0	3	3	9	1
AWC::eat-4	0	3	3	10	1
AWC::eat-4	0	3	3	11	1
AWC::eat-4	0	3	3	12	1
AWC::eat-4	0	3	3	13	1
AWC::eat-4	0	3	4	1	1
AWC::eat-4	0	3	4	2	1
AWC::eat-4	0	3	4	3	1
AWC::eat-4	0	3	4	4	1
AWC::eat-4	0	3	4	5	1
AWC::eat-4	0	3	4	6	1
AWC::eat-4	0	3	4	7	1
AWC::eat-4	0	3	4	8	1
AWC::eat-4	0	3	4	9	1
AWC::eat-4	0	3	4	10	1
AWC::eat-4	0	3	4	11	0
AWC::eat-4	0	3	4	12	1
AWC::eat-4	3	1	1	1	1
AWC::eat-4	3	1	1	2	0
AWC::eat-4	3	1	1	3	0
AWC::eat-4	3	1	1	4	0
AWC::eat-4	3	1	1	5	0
AWC::eat-4	3	1	1	6	1
AWC::eat-4	3	1	1	7	0
AWC::eat-4	3	1	1	8	0
AWC::eat-4	3	1	1	9	0
AWC::eat-4	3	1	1	10	1
AWC::eat-4	3	1	1	11	1
AWC::eat-4	3	1	1	12	0
AWC::eat-4	3	1	1	13	1
AWC::eat-4	3	1	1	14	0
AWC::eat-4	3	1	1	15	1
AWC::eat-4	3	1	1	16	0
AWC::eat-4	3	1	1	17	0
AWC::eat-4	3	1	2	1	1
AWC::eat-4	3	1	2	2	1
AWC::eat-4	3	1	2	3	0

AWC::eat-4	3	1	2	4	1
AWC::eat-4	3	1	2	5	0
AWC::eat-4	3	1	2	6	1
AWC::eat-4	3	1	2	7	0
AWC::eat-4	3	1	2	8	1
AWC::eat-4	3	1	2	9	0
AWC::eat-4	3	1	2	10	1
AWC::eat-4	3	1	2	11	1
AWC::eat-4	3	1	2	12	0
AWC::eat-4	3	1	2	13	1
AWC::eat-4	3	1	2	14	1
AWC::eat-4	3	1	2	15	1
AWC::eat-4	3	1	2	16	1
AWC::eat-4	3	1	2	17	1
AWC::eat-4	3	1	2	18	0
AWC::eat-4	3	1	2	19	0
AWC::eat-4	3	1	2	20	0
AWC::eat-4	3	1	3	1	1
AWC::eat-4	3	1	3	2	1
AWC::eat-4	3	1	3	3	0
AWC::eat-4	3	1	3	4	1
AWC::eat-4	3	1	3	5	0
AWC::eat-4	3	1	3	6	1
AWC::eat-4	3	1	3	7	1
AWC::eat-4	3	1	3	8	1
AWC::eat-4	3	1	3	9	1
AWC::eat-4	3	1	3	10	0
AWC::eat-4	3	1	3	11	1
AWC::eat-4	3	1	3	12	1
AWC::eat-4	3	1	3	13	0
AWC::eat-4	3	1	3	14	1
AWC::eat-4	3	1	3	15	0
AWC::eat-4	3	1	3	16	1
AWC::eat-4	3	1	3	17	0
AWC::eat-4	3	1	4	1	0
AWC::eat-4	3	1	4	2	0
AWC::eat-4	3	1	4	3	1
AWC::eat-4	3	1	4	4	0
AWC::eat-4	3	1	4	5	1
AWC::eat-4	3	1	4	6	1
AWC::eat-4	3	1	4	7	1
AWC::eat-4	3	1	4	8	1
AWC::eat-4	3	1	4	9	1
AWC::eat-4	3	1	4	10	1
AWC::eat-4	3	1	4	11	1

AWC::eat-4	3	2	1	1	1
AWC::eat-4	3	2	1	2	0
AWC::eat-4	3	2	1	3	0
AWC::eat-4	3	2	1	4	0
AWC::eat-4	3	2	1	5	1
AWC::eat-4	3	2	1	6	1
AWC::eat-4	3	2	1	7	1
AWC::eat-4	3	2	1	8	0
AWC::eat-4	3	2	1	9	1
AWC::eat-4	3	2	1	10	0
AWC::eat-4	3	2	1	11	1
AWC::eat-4	3	2	2	1	1
AWC::eat-4	3	2	2	2	1
AWC::eat-4	3	2	2	3	1
AWC::eat-4	3	2	2	4	1
AWC::eat-4	3	2	2	5	0
AWC::eat-4	3	2	2	6	0
AWC::eat-4	3	2	2	7	1
AWC::eat-4	3	2	2	8	1
AWC::eat-4	3	2	2	9	1
AWC::eat-4	3	2	2	10	1
AWC::eat-4	3	2	2	11	0
AWC::eat-4	3	2	2	12	1
AWC::eat-4	3	2	2	13	1
AWC::eat-4	3	2	2	14	1
AWC::eat-4	3	2	2	15	1
AWC::eat-4	3	2	3	1	0
AWC::eat-4	3	2	3	2	1
AWC::eat-4	3	2	3	3	1
AWC::eat-4	3	2	3	4	1
AWC::eat-4	3	2	3	5	1
AWC::eat-4	3	2	3	6	0
AWC::eat-4	3	2	3	7	1
AWC::eat-4	3	2	3	8	1
AWC::eat-4	3	2	3	9	1
AWC::eat-4	3	2	3	10	1
AWC::eat-4	3	2	3	11	1
AWC::eat-4	3	2	3	12	0
AWC::eat-4	3	2	3	13	0
AWC::eat-4	3	2	3	14	0
AWC::eat-4	3	2	3	15	1
AWC::eat-4	3	2	3	16	1
AWC::eat-4	3	2	3	17	1
AWC::eat-4	3	2	3	18	0
AWC::eat-4	3	2	4	1	1

AWC::eat-4	3	2	4	2	1
AWC::eat-4	3	2	4	3	0
AWC::eat-4	3	2	4	4	1
AWC::eat-4	3	2	4	5	0
AWC::eat-4	3	2	4	6	0
AWC::eat-4	3	2	4	7	1
AWC::eat-4	3	2	4	8	1
AWC::eat-4	3	2	4	9	1
AWC::eat-4	3	2	4	10	1
AWC::eat-4	3	2	4	11	1
AWC::eat-4	3	2	4	12	1
AWC::eat-4	3	2	4	13	1
AWC::eat-4	3	2	4	14	0
AWC::eat-4	3	2	4	15	0
AWC::eat-4	3	3	1	1	0
AWC::eat-4	3	3	1	2	0
AWC::eat-4	3	3	1	3	0
AWC::eat-4	3	3	1	4	0
AWC::eat-4	3	3	1	5	1
AWC::eat-4	3	3	1	6	1
AWC::eat-4	3	3	1	7	1
AWC::eat-4	3	3	1	8	1
AWC::eat-4	3	3	1	9	1
AWC::eat-4	3	3	1	10	1
AWC::eat-4	3	3	1	11	0
AWC::eat-4	3	3	1	12	0
AWC::eat-4	3	3	1	13	1
AWC::eat-4	3	3	1	14	0
AWC::eat-4	3	3	1	15	1
AWC::eat-4	3	3	1	16	0
AWC::eat-4	3	3	1	17	1
AWC::eat-4	3	3	1	18	0
AWC::eat-4	3	3	1	19	0
AWC::eat-4	3	3	2	1	1
AWC::eat-4	3	3	2	2	1
AWC::eat-4	3	3	2	3	1
AWC::eat-4	3	3	2	4	1
AWC::eat-4	3	3	2	5	1
AWC::eat-4	3	3	2	6	1
AWC::eat-4	3	3	2	7	1
AWC::eat-4	3	3	2	8	0
AWC::eat-4	3	3	2	9	1
AWC::eat-4	3	3	2	10	0
AWC::eat-4	3	3	2	11	1
AWC::eat-4	3	3	2	12	0

AWC::eat-4	3	3	2	13	0
AWC::eat-4	3	3	2	14	0
AWC::eat-4	3	3	2	15	1
AWC::eat-4	3	3	2	16	0
AWC::eat-4	3	3	2	17	1
AWC::eat-4	3	3	3	1	1
AWC::eat-4	3	3	3	2	0
AWC::eat-4	3	3	3	3	0
AWC::eat-4	3	3	3	4	0
AWC::eat-4	3	3	3	5	1
AWC::eat-4	3	3	3	6	0
AWC::eat-4	3	3	3	7	0
AWC::eat-4	3	3	3	8	0
AWC::eat-4	3	3	3	9	0
AWC::eat-4	3	3	3	10	1
AWC::eat-4	3	3	3	11	1
AWC::eat-4	3	3	3	12	1
AWC::eat-4	3	3	3	13	1
AWC::eat-4	3	3	3	14	0
AWC::eat-4	3	3	3	15	1
AWC::eat-4	3	3	3	16	1
AWC::eat-4	3	3	3	17	1
AWC::eat-4	3	3	3	18	0
AWC::eat-4	3	3	3	19	1
AWC::eat-4	3	3	3	20	0
AWC::eat-4	3	3	4	1	1
AWC::eat-4	3	3	4	2	0
AWC::eat-4	3	3	4	3	1
AWC::eat-4	3	3	4	4	1
AWC::eat-4	3	3	4	5	1
AWC::eat-4	3	3	4	6	1
AWC::eat-4	3	3	4	7	0
AWC::eat-4	3	3	4	8	1
AWC::eat-4	3	3	4	9	1
AWC::eat-4	3	3	4	10	1
AWC::eat-4	3	3	4	11	1
AWC::eat-4	3	3	4	12	1
AWC::eat-4	3	3	4	13	0
AWC::eat-4	3	3	4	14	1
AWC::eat-4	3	3	4	15	1
AWC::eat-4	3	3	4	16	1
AWC::eat-4	3	2	4	30	0
AWC::eat-4	3	3	1	1	0
AWC::eat-4	3	3	1	2	0
AWC::eat-4	3	3	1	3	0

AWC::eat-4	3	3	1	4	0
AWC::eat-4	3	3	1	5	0
AWC::eat-4	3	3	1	6	0
AWC::eat-4	3	3	1	7	0
AWC::eat-4	3	3	1	8	0
AWC::eat-4	3	3	1	9	0
AWC::eat-4	3	3	1	10	0
AWC::eat-4	3	3	1	11	0
AWC::eat-4	3	3	1	12	0
AWC::eat-4	3	3	1	13	1
AWC::eat-4	3	3	1	14	0
AWC::eat-4	3	3	2	1	0
AWC::eat-4	3	3	2	2	0
AWC::eat-4	3	3	2	3	0
AWC::eat-4	3	3	2	4	0
AWC::eat-4	3	3	2	5	0
AWC::eat-4	3	3	2	6	0
AWC::eat-4	3	3	2	7	0
AWC::eat-4	3	3	2	8	1
AWC::eat-4	3	3	2	9	0
AWC::eat-4	3	3	2	10	0
AWC::eat-4	3	3	2	11	0
AWC::eat-4	3	3	2	12	0
AWC::eat-4	3	3	2	13	0
AWC::eat-4	3	3	2	14	0
AWC::eat-4	3	3	2	15	0
AWC::eat-4	3	3	2	16	1
AWC::eat-4	3	3	2	17	0
AWC::eat-4	3	3	2	18	0
AWC::eat-4	3	3	2	19	0
AWC::eat-4	3	3	2	20	0
AWC::eat-4	3	3	2	21	0
AWC::eat-4	3	3	2	22	0
AWC::eat-4	3	3	2	23	0
AWC::eat-4	3	3	2	24	1
AWC::eat-4	3	3	2	25	0
AWC::eat-4	3	3	2	26	0
AWC::eat-4	3	3	2	27	0
AWC::eat-4	3	3	2	28	0
AWC::eat-4	3	3	2	29	0
AWC::eat-4	3	3	2	30	0
AWC::eat-4	3	3	2	31	0
AWC::eat-4	3	3	2	32	0
AWC::eat-4	3	3	2	33	0
AWC::eat-4	3	3	3	1	0

AWC::eat-4	3	3	3	2	0
AWC::eat-4	3	3	3	3	0
AWC::eat-4	3	3	3	4	0
AWC::eat-4	3	3	3	5	0
AWC::eat-4	3	3	3	6	0
AWC::eat-4	3	3	3	7	0
AWC::eat-4	3	3	3	8	0
AWC::eat-4	3	3	3	9	0
AWC::eat-4	3	3	3	10	0
AWC::eat-4	3	3	3	11	0
AWC::eat-4	3	3	3	12	0
AWC::eat-4	3	3	3	13	0
AWC::eat-4	3	3	3	14	0
AWC::eat-4	3	3	3	15	0
AWC::eat-4	3	3	3	16	0
AWC::eat-4	3	3	3	17	0
AWC::eat-4	3	3	3	18	0
AWC::eat-4	3	3	3	19	0
AWC::eat-4	3	3	3	20	0
AWC::eat-4	3	3	3	21	0
AWC::eat-4	3	3	4	1	0
AWC::eat-4	3	3	4	2	0
AWC::eat-4	3	3	4	3	1
ceh-36	0	1	1	1	1
ceh-36	0	1	1	2	1
ceh-36	0	1	1	3	1
ceh-36	0	1	1	4	1
ceh-36	0	1	1	5	1
ceh-36	0	1	1	6	0
ceh-36	0	1	2	1	1
ceh-36	0	1	2	2	0
ceh-36	0	1	2	3	0
ceh-36	0	1	2	4	0
ceh-36	0	1	2	5	1
ceh-36	0	1	2	6	0
ceh-36	0	1	2	7	0
ceh-36	0	1	2	8	0
ceh-36	0	1	2	9	1
ceh-36	0	1	2	10	1
ceh-36	0	1	2	11	1
ceh-36	0	1	2	12	1
ceh-36	0	1	2	13	1
ceh-36	0	1	3	1	0
ceh-36	0	1	3	2	1

ceh-36	0	1	3	3	1
ceh-36	0	1	3	4	1
ceh-36	0	1	3	5	1
ceh-36	0	1	3	6	0
ceh-36	0	1	3	7	1
ceh-36	0	1	3	8	1
ceh-36	0	1	3	9	1
ceh-36	0	1	3	10	1
ceh-36	0	1	3	11	1
ceh-36	0	1	3	12	1
ceh-36	0	1	3	13	1
ceh-36	0	1	3	14	1
ceh-36	0	1	3	15	1
ceh-36	0	1	4	1	1
ceh-36	0	1	4	2	1
ceh-36	0	1	4	3	1
ceh-36	0	1	4	4	1
ceh-36	0	1	4	5	1
ceh-36	0	1	4	6	1
ceh-36	0	1	4	7	1
ceh-36	0	1	4	8	1
ceh-36	0	1	4	9	1
ceh-36	0	1	4	10	1
ceh-36	0	1	4	11	1
ceh-36	0	1	4	12	0
ceh-36	0	1	4	13	1
ceh-36	0	1	4	14	1
ceh-36	0	1	4	15	1
ceh-36	0	1	4	16	0
ceh-36	0	1	4	17	1
ceh-36	0	1	4	18	0
ceh-36	0	1	4	19	1
ceh-36	0	1	4	20	1
ceh-36	0	1	4	21	0
ceh-36	0	2	1	1	1
ceh-36	0	2	1	2	1
ceh-36	0	2	1	3	1
ceh-36	0	2	1	4	1
ceh-36	0	2	1	5	1
ceh-36	0	2	1	6	0
ceh-36	0	2	1	7	1
ceh-36	0	2	2	1	0
ceh-36	0	2	2	2	1
ceh-36	0	2	2	3	1
ceh-36	0	2	2	4	1

ceh-36	0	2	2	5	1
ceh-36	0	2	2	6	1
ceh-36	0	2	2	7	1
ceh-36	0	2	2	8	1
ceh-36	0	2	3	1	0
ceh-36	0	2	3	2	1
ceh-36	0	2	3	3	1
ceh-36	0	2	3	4	1
ceh-36	0	2	3	5	1
ceh-36	0	2	3	6	1
ceh-36	0	2	3	7	1
ceh-36	0	2	3	8	1
ceh-36	0	2	3	9	1
ceh-36	0	2	3	10	1
ceh-36	0	2	3	11	0
ceh-36	0	2	3	12	1
ceh-36	0	2	3	13	1
ceh-36	0	2	3	14	0
ceh-36	0	2	3	15	1
ceh-36	0	2	3	16	1
ceh-36	0	2	4	1	1
ceh-36	0	2	4	2	0
ceh-36	0	2	4	3	1
ceh-36	0	2	4	4	1
ceh-36	0	2	4	5	1
ceh-36	0	2	4	6	1
ceh-36	0	2	4	7	1
ceh-36	0	2	4	8	1
ceh-36	0	2	4	9	1
ceh-36	0	2	4	10	1
ceh-36	0	2	4	11	0
ceh-36	0	2	4	12	1
ceh-36	0	2	4	13	1
ceh-36	0	2	4	14	1
ceh-36	0	2	4	15	0
ceh-36	0	2	4	16	1
ceh-36	0	2	4	17	1
ceh-36	0	3	1	1	1
ceh-36	0	3	1	2	1
ceh-36	0	3	1	3	1
ceh-36	0	3	1	4	0
ceh-36	0	3	1	5	1
ceh-36	0	3	1	6	1
ceh-36	0	3	1	7	0
ceh-36	0	3	1	8	0

ceh-36	0	3	1	9	1
ceh-36	0	3	1	10	1
ceh-36	0	3	1	11	0
ceh-36	0	3	1	12	1
ceh-36	0	3	2	1	0
ceh-36	0	3	2	2	1
ceh-36	0	3	2	3	1
ceh-36	0	3	2	4	0
ceh-36	0	3	2	5	1
ceh-36	0	3	2	6	0
ceh-36	0	3	2	7	1
ceh-36	0	3	2	8	1
ceh-36	0	3	2	9	1
ceh-36	0	3	2	10	1
ceh-36	0	3	2	11	1
ceh-36	0	3	2	12	1
ceh-36	0	3	2	13	0
ceh-36	0	3	2	14	1
ceh-36	0	3	2	15	0
ceh-36	0	3	2	16	1
ceh-36	0	3	2	17	0
ceh-36	0	3	3	1	1
ceh-36	0	3	3	2	1
ceh-36	0	3	3	3	0
ceh-36	0	3	3	4	1
ceh-36	0	3	3	5	0
ceh-36	0	3	3	6	1
ceh-36	0	3	3	7	1
ceh-36	0	3	3	8	1
ceh-36	0	3	3	9	1
ceh-36	0	3	3	10	1
ceh-36	0	3	3	11	1
ceh-36	0	3	3	12	1
ceh-36	0	3	4	1	0
ceh-36	0	3	4	2	0
ceh-36	0	3	4	3	1
ceh-36	0	3	4	4	0
ceh-36	0	3	4	5	1
ceh-36	0	3	4	6	1
ceh-36	0	3	4	7	1
ceh-36	0	3	4	8	1
ceh-36	0	3	4	9	1
ceh-36	0	3	4	10	1
ceh-36	0	3	4	11	1
ceh-36	0	3	4	12	0

ceh-36	0	3	4	13	1
ceh-36	0	3	4	14	0
ceh-36	0	3	4	15	1
ceh-36	0	3	4	16	1
ceh-36	0	3	4	17	1
ceh-36	0	3	4	18	1
ceh-36	0	3	4	19	1
ceh-36	3	1	1	1	0
ceh-36	3	1	1	2	1
ceh-36	3	1	1	3	1
ceh-36	3	1	1	4	1
ceh-36	3	1	1	5	1
ceh-36	3	1	1	6	0
ceh-36	3	1	1	7	1
ceh-36	3	1	2	1	0
ceh-36	3	1	2	2	0
ceh-36	3	1	2	3	0
ceh-36	3	1	2	4	0
ceh-36	3	1	2	5	0
ceh-36	3	1	2	6	0
ceh-36	3	1	2	7	0
ceh-36	3	1	2	8	0
ceh-36	3	1	2	9	1
ceh-36	3	1	2	10	1
ceh-36	3	1	2	11	1
ceh-36	3	1	2	12	1
ceh-36	3	1	2	13	0
ceh-36	3	1	2	14	0
ceh-36	3	1	2	15	1
ceh-36	3	1	3	1	1
ceh-36	3	1	3	2	0
ceh-36	3	1	3	3	1
ceh-36	3	1	3	4	0
ceh-36	3	1	3	5	1
ceh-36	3	1	3	6	0
ceh-36	3	1	3	7	1
ceh-36	3	1	3	8	0
ceh-36	3	1	3	9	1
ceh-36	3	1	3	10	1
ceh-36	3	1	3	11	1
ceh-36	3	1	3	12	1
ceh-36	3	1	3	13	1
ceh-36	3	1	3	14	0
ceh-36	3	1	3	15	1
ceh-36	3	1	3	16	1

ceh-36	3	1	3	17	0
ceh-36	3	1	4	1	0
ceh-36	3	1	4	2	0
ceh-36	3	1	4	3	1
ceh-36	3	1	4	4	0
ceh-36	3	1	4	5	1
ceh-36	3	1	4	6	1
ceh-36	3	1	4	7	0
ceh-36	3	1	4	8	0
ceh-36	3	1	4	9	1
ceh-36	3	1	4	10	1
ceh-36	3	1	4	11	1
ceh-36	3	1	4	12	1
ceh-36	3	1	4	13	1
ceh-36	3	1	4	14	0
ceh-36	3	1	4	15	1
ceh-36	3	1	4	16	1
ceh-36	3	1	4	17	1
ceh-36	3	2	1	1	0
ceh-36	3	2	1	2	1
ceh-36	3	2	1	3	0
ceh-36	3	2	1	4	1
ceh-36	3	2	1	5	1
ceh-36	3	2	1	6	1
ceh-36	3	2	1	7	1
ceh-36	3	2	1	8	1
ceh-36	3	2	1	9	0
ceh-36	3	2	1	10	1
ceh-36	3	2	2	1	0
ceh-36	3	2	2	2	0
ceh-36	3	2	2	3	0
ceh-36	3	2	2	4	0
ceh-36	3	2	2	5	1
ceh-36	3	2	2	6	1
ceh-36	3	2	2	7	1
ceh-36	3	2	2	8	1
ceh-36	3	2	2	9	1
ceh-36	3	2	2	10	0
ceh-36	3	2	2	11	0
ceh-36	3	2	2	12	0
ceh-36	3	2	2	13	0
ceh-36	3	2	2	14	0
ceh-36	3	2	2	15	1
ceh-36	3	2	3	1	0
ceh-36	3	2	3	2	1

ceh-36	3	2	3	3	0
ceh-36	3	2	3	4	0
ceh-36	3	2	3	5	0
ceh-36	3	2	3	6	0
ceh-36	3	2	3	7	0
ceh-36	3	2	3	8	0
ceh-36	3	2	3	9	0
ceh-36	3	2	3	10	0
ceh-36	3	2	3	11	1
ceh-36	3	2	3	12	1
ceh-36	3	2	3	13	1
ceh-36	3	2	3	14	1
ceh-36	3	2	3	15	0
ceh-36	3	2	3	16	1
ceh-36	3	2	3	17	0
ceh-36	3	2	3	18	0
ceh-36	3	2	3	19	1
ceh-36	3	2	3	20	1
ceh-36	3	2	3	21	0
ceh-36	3	2	3	22	1
ceh-36	3	2	3	23	1
ceh-36	3	2	3	24	0
ceh-36	3	2	4	1	0
ceh-36	3	2	4	2	1
ceh-36	3	2	4	3	0
ceh-36	3	2	4	4	0
ceh-36	3	2	4	5	0
ceh-36	3	2	4	6	0
ceh-36	3	2	4	7	1
ceh-36	3	2	4	8	1
ceh-36	3	2	4	9	1
ceh-36	3	2	4	10	1
ceh-36	3	2	4	11	1
ceh-36	3	2	4	12	1
ceh-36	3	2	4	13	1
ceh-36	3	2	4	14	1
ceh-36	3	2	4	15	1
ceh-36	3	2	4	16	0
ceh-36	3	2	4	17	1
ceh-36	3	2	4	18	0
ceh-36	3	2	4	19	0
ceh-36	3	2	4	20	0
ceh-36	3	2	4	21	0
ceh-36	3	2	4	22	0
ceh-36	3	3	1	1	1

ceh-36	3	3	1	2	0
ceh-36	3	3	1	3	0
ceh-36	3	3	1	4	1
ceh-36	3	3	1	5	0
ceh-36	3	3	1	6	1
ceh-36	3	3	1	7	0
ceh-36	3	3	1	8	1
ceh-36	3	3	1	9	0
ceh-36	3	3	2	1	0
ceh-36	3	3	2	2	0
ceh-36	3	3	2	3	0
ceh-36	3	3	2	4	0
ceh-36	3	3	2	5	1
ceh-36	3	3	2	6	0
ceh-36	3	3	2	7	0
ceh-36	3	3	2	8	0
ceh-36	3	3	2	9	1
ceh-36	3	3	2	10	0
ceh-36	3	3	2	11	0
ceh-36	3	3	2	12	0
ceh-36	3	3	2	13	0
ceh-36	3	3	2	14	1
ceh-36	3	3	2	15	1
ceh-36	3	3	3	1	0
ceh-36	3	3	3	2	0
ceh-36	3	3	3	3	0
ceh-36	3	3	3	4	1
ceh-36	3	3	3	5	0
ceh-36	3	3	3	6	1
ceh-36	3	3	3	7	0
ceh-36	3	3	3	8	0
ceh-36	3	3	3	9	1
ceh-36	3	3	3	10	0
ceh-36	3	3	3	11	1
ceh-36	3	3	3	12	0
ceh-36	3	3	3	13	0
ceh-36	3	3	3	14	0
ceh-36	3	3	3	15	0
ceh-36	3	3	3	16	0
ceh-36	3	3	3	17	0
ceh-36	3	3	3	18	1
ceh-36	3	3	3	19	0
ceh-36	3	3	3	20	0
ceh-36	3	3	3	21	0
ceh-36	3	3	3	22	0

ceh-36	3	3	3	23	0
ceh-36	3	3	3	24	1
ceh-36	3	3	3	25	0
ceh-36	3	3	3	1	1
ceh-36	3	3	3	2	1
ceh-36	3	3	3	3	0
ceh-36	3	3	3	4	0
ceh-36	3	3	3	5	1
ceh-36	3	3	3	6	1
ceh-36	3	3	3	7	1
ceh-36	3	3	3	8	0
ceh-36	3	3	3	9	1
ceh-36	3	3	3	10	1
ceh-36	3	3	3	11	1
ceh-36	3	3	3	12	1
ceh-36	3	3	3	13	1
ceh-36	3	3	3	14	1
ceh-36	3	3	3	15	0
ceh-36	3	3	3	16	0
ceh-36	3	3	3	17	1
ceh-36	3	3	3	18	1
ceh-36	3	3	3	19	0
ceh-36	3	3	3	20	1
nsy-1 ($AWC^{ON/ON}$)	0	1	1	1	1
nsy-1 ($AWC^{ON/ON}$)	0	1	1	2	1
nsy-1 ($AWC^{ON/ON}$)	0	1	1	3	1
nsy-1 ($AWC^{ON/ON}$)	0	1	1	4	1
nsy-1 ($AWC^{ON/ON}$)	0	1	1	5	1
nsy-1 ($AWC^{ON/ON}$)	0	1	1	6	1
nsy-1 ($AWC^{ON/ON}$)	0	1	1	7	1
nsy-1 ($AWC^{ON/ON}$)	0	1	1	8	1
nsy-1 ($AWC^{ON/ON}$)	0	1	1	9	1
nsy-1 ($AWC^{ON/ON}$)	0	1	1	10	1
nsy-1 ($AWC^{ON/ON}$)	0	1	1	11	1
nsy-1 ($AWC^{ON/ON}$)	0	1	2	1	1
nsy-1 ($AWC^{ON/ON}$)	0	1	2	2	1
nsy-1 ($AWC^{ON/ON}$)	0	1	2	3	1
nsy-1 ($AWC^{ON/ON}$)	0	1	2	4	1
nsy-1 ($AWC^{ON/ON}$)	0	1	2	5	1
nsy-1 ($AWC^{ON/ON}$)	0	1	2	6	1
nsy-1 ($AWC^{ON/ON}$)	0	1	2	7	1
nsy-1 ($AWC^{ON/ON}$)	0	1	2	8	1
nsy-1 ($AWC^{ON/ON}$)	0	1	2	9	1

nsy-1 (AWC$^{ON/ON}$)	0	1	2	10	1
nsy-1 (AWC$^{ON/ON}$)	0	1	2	11	1
nsy-1 (AWC$^{ON/ON}$)	0	1	2	12	1
nsy-1 (AWC$^{ON/ON}$)	0	1	3	1	1
nsy-1 (AWC$^{ON/ON}$)	0	1	3	2	1
nsy-1 (AWC$^{ON/ON}$)	0	1	3	3	1
nsy-1 (AWC$^{ON/ON}$)	0	1	3	4	1
nsy-1 (AWC$^{ON/ON}$)	0	1	3	5	1
nsy-1 (AWC$^{ON/ON}$)	0	1	3	6	1
nsy-1 (AWC$^{ON/ON}$)	0	1	3	7	1
nsy-1 (AWC$^{ON/ON}$)	0	1	3	8	1
nsy-1 (AWC$^{ON/ON}$)	0	1	3	9	0
nsy-1 (AWC$^{ON/ON}$)	0	1	3	10	1
nsy-1 (AWC$^{ON/ON}$)	0	1	3	11	1
nsy-1 (AWC$^{ON/ON}$)	0	1	3	12	1
nsy-1 (AWC$^{ON/ON}$)	0	1	3	13	1
nsy-1 (AWC$^{ON/ON}$)	0	1	3	14	1
nsy-1 (AWC$^{ON/ON}$)	0	1	3	15	1
nsy-1 (AWC$^{ON/ON}$)	0	1	4	1	1
nsy-1 (AWC$^{ON/ON}$)	0	1	4	2	1
nsy-1 (AWC$^{ON/ON}$)	0	1	4	3	1
nsy-1 (AWC$^{ON/ON}$)	0	1	4	4	1
nsy-1 (AWC$^{ON/ON}$)	0	1	4	5	1
nsy-1 (AWC$^{ON/ON}$)	0	1	4	6	0
nsy-1 (AWC$^{ON/ON}$)	0	1	4	7	0
nsy-1 (AWC$^{ON/ON}$)	0	1	4	8	1
nsy-1 (AWC$^{ON/ON}$)	0	1	4	9	1
nsy-1 (AWC$^{ON/ON}$)	0	1	4	10	1
nsy-1 (AWC$^{ON/ON}$)	0	1	4	11	1
nsy-1 (AWC$^{ON/ON}$)	0	1	4	12	1
nsy-1 (AWC$^{ON/ON}$)	0	1	4	13	1
nsy-1 (AWC$^{ON/ON}$)	0	1	4	14	1
nsy-1 (AWC$^{ON/ON}$)	0	1	1	15	1
nsy-1 (AWC$^{ON/ON}$)	0	2	1	1	1
nsy-1 (AWC$^{ON/ON}$)	0	2	1	2	1
nsy-1 (AWC$^{ON/ON}$)	0	2	1	3	1
nsy-1 (AWC$^{ON/ON}$)	0	2	1	4	1
nsy-1 (AWC$^{ON/ON}$)	0	2	1	5	1
nsy-1 (AWC$^{ON/ON}$)	0	2	1	6	1
nsy-1 (AWC$^{ON/ON}$)	0	2	1	7	1
nsy-1 (AWC$^{ON/ON}$)	0	2	1	8	0
nsy-1 (AWC$^{ON/ON}$)	0	2	1	9	1
nsy-1 (AWC$^{ON/ON}$)	0	2	1	10	1

nsy-1 ($AWC^{ON/ON}$)	0	2	1	11	1
nsy-1 ($AWC^{ON/ON}$)	0	2	1	12	1
nsy-1 ($AWC^{ON/ON}$)	0	2	1	13	1
nsy-1 ($AWC^{ON/ON}$)	0	2	1	14	1
nsy-1 ($AWC^{ON/ON}$)	0	2	1	15	1
nsy-1 ($AWC^{ON/ON}$)	0	2	1	16	1
nsy-1 ($AWC^{ON/ON}$)	0	2	2	1	1
nsy-1 ($AWC^{ON/ON}$)	0	2	2	2	1
nsy-1 ($AWC^{ON/ON}$)	0	2	2	3	1
nsy-1 ($AWC^{ON/ON}$)	0	2	2	4	1
nsy-1 ($AWC^{ON/ON}$)	0	2	2	5	1
nsy-1 ($AWC^{ON/ON}$)	0	2	2	6	1
nsy-1 ($AWC^{ON/ON}$)	0	2	2	7	1
nsy-1 ($AWC^{ON/ON}$)	0	2	2	8	1
nsy-1 ($AWC^{ON/ON}$)	0	2	2	9	1
nsy-1 ($AWC^{ON/ON}$)	0	2	2	10	0
nsy-1 ($AWC^{ON/ON}$)	0	2	2	11	1
nsy-1 ($AWC^{ON/ON}$)	0	2	2	12	1
nsy-1 ($AWC^{ON/ON}$)	0	2	2	13	1
nsy-1 ($AWC^{ON/ON}$)	0	2	2	14	1
nsy-1 ($AWC^{ON/ON}$)	0	2	2	15	1
nsy-1 ($AWC^{ON/ON}$)	0	2	2	16	1
nsy-1 ($AWC^{ON/ON}$)	0	2	2	17	1
nsy-1 ($AWC^{ON/ON}$)	0	2	2	18	0
nsy-1 ($AWC^{ON/ON}$)	0	2	2	19	1
nsy-1 ($AWC^{ON/ON}$)	0	2	3	1	1
nsy-1 ($AWC^{ON/ON}$)	0	2	3	2	1
nsy-1 ($AWC^{ON/ON}$)	0	2	3	3	1
nsy-1 ($AWC^{ON/ON}$)	0	2	3	4	1
nsy-1 ($AWC^{ON/ON}$)	0	2	3	5	1
nsy-1 ($AWC^{ON/ON}$)	0	2	3	6	0
nsy-1 ($AWC^{ON/ON}$)	0	2	3	7	1
nsy-1 ($AWC^{ON/ON}$)	0	2	3	8	1
nsy-1 ($AWC^{ON/ON}$)	0	2	3	9	1
nsy-1 ($AWC^{ON/ON}$)	0	2	3	10	1
nsy-1 ($AWC^{ON/ON}$)	0	2	3	11	1
nsy-1 ($AWC^{ON/ON}$)	0	2	3	12	1
nsy-1 ($AWC^{ON/ON}$)	0	2	3	13	0
nsy-1 ($AWC^{ON/ON}$)	0	2	3	14	1
nsy-1 ($AWC^{ON/ON}$)	0	2	3	15	1
nsy-1 ($AWC^{ON/ON}$)	0	2	3	16	1
nsy-1 ($AWC^{ON/ON}$)	0	2	3	17	1
nsy-1 ($AWC^{ON/ON}$)	0	2	3	18	1

nsy-1 (AWC$^{ON/ON}$)	0	2	4	1	0
nsy-1 (AWC$^{ON/ON}$)	0	2	4	2	0
nsy-1 (AWC$^{ON/ON}$)	0	2	4	3	1
nsy-1 (AWC$^{ON/ON}$)	0	2	4	4	1
nsy-1 (AWC$^{ON/ON}$)	0	2	4	5	1
nsy-1 (AWC$^{ON/ON}$)	0	2	4	6	1
nsy-1 (AWC$^{ON/ON}$)	0	2	4	7	0
nsy-1 (AWC$^{ON/ON}$)	0	2	4	8	1
nsy-1 (AWC$^{ON/ON}$)	0	2	4	9	1
nsy-1 (AWC$^{ON/ON}$)	0	2	4	10	1
nsy-1 (AWC$^{ON/ON}$)	0	2	4	11	1
nsy-1 (AWC$^{ON/ON}$)	0	2	4	12	0
nsy-1 (AWC$^{ON/ON}$)	0	2	4	13	1
nsy-1 (AWC$^{ON/ON}$)	0	2	4	14	1
nsy-1 (AWC$^{ON/ON}$)	0	2	4	15	0
nsy-1 (AWC$^{ON/ON}$)	0	2	4	16	1
nsy-1 (AWC$^{ON/ON}$)	0	2	4	17	1
nsy-1 (AWC$^{ON/ON}$)	0	2	4	18	1
nsy-1 (AWC$^{ON/ON}$)	0	2	4	19	0
nsy-1 (AWC$^{ON/ON}$)	0	2	4	20	1
nsy-1 (AWC$^{ON/ON}$)	0	3	1	1	1
nsy-1 (AWC$^{ON/ON}$)	0	3	1	2	1
nsy-1 (AWC$^{ON/ON}$)	0	3	1	3	1
nsy-1 (AWC$^{ON/ON}$)	0	3	1	4	1
nsy-1 (AWC$^{ON/ON}$)	0	3	1	5	1
nsy-1 (AWC$^{ON/ON}$)	0	3	1	6	1
nsy-1 (AWC$^{ON/ON}$)	0	3	1	7	1
nsy-1 (AWC$^{ON/ON}$)	0	3	1	8	1
nsy-1 (AWC$^{ON/ON}$)	0	3	1	9	1
nsy-1 (AWC$^{ON/ON}$)	0	3	1	10	1
nsy-1 (AWC$^{ON/ON}$)	0	3	1	11	1
nsy-1 (AWC$^{ON/ON}$)	0	3	1	12	1
nsy-1 (AWC$^{ON/ON}$)	0	3	1	13	1
nsy-1 (AWC$^{ON/ON}$)	0	3	1	14	1
nsy-1 (AWC$^{ON/ON}$)	0	3	1	15	1
nsy-1 (AWC$^{ON/ON}$)	0	3	1	16	1
nsy-1 (AWC$^{ON/ON}$)	0	3	1	17	1
nsy-1 (AWC$^{ON/ON}$)	0	3	1	18	1
nsy-1 (AWC$^{ON/ON}$)	0	3	1	19	1
nsy-1 (AWC$^{ON/ON}$)	0	3	2	1	1
nsy-1 (AWC$^{ON/ON}$)	0	3	2	2	0
nsy-1 (AWC$^{ON/ON}$)	0	3	2	3	1
nsy-1 (AWC$^{ON/ON}$)	0	3	2	4	0

nsy-1 (AWC$^{ON/ON}$)	0	3	2	5	1
nsy-1 (AWC$^{ON/ON}$)	0	3	2	6	1
nsy-1 (AWC$^{ON/ON}$)	0	3	2	7	1
nsy-1 (AWC$^{ON/ON}$)	0	3	2	8	1
nsy-1 (AWC$^{ON/ON}$)	0	3	2	9	1
nsy-1 (AWC$^{ON/ON}$)	0	3	2	10	1
nsy-1 (AWC$^{ON/ON}$)	0	3	2	11	1
nsy-1 (AWC$^{ON/ON}$)	0	3	2	12	1
nsy-1 (AWC$^{ON/ON}$)	0	3	2	13	0
nsy-1 (AWC$^{ON/ON}$)	0	3	3	1	0
nsy-1 (AWC$^{ON/ON}$)	0	3	3	2	1
nsy-1 (AWC$^{ON/ON}$)	0	3	3	3	1
nsy-1 (AWC$^{ON/ON}$)	0	3	3	4	1
nsy-1 (AWC$^{ON/ON}$)	0	3	3	5	1
nsy-1 (AWC$^{ON/ON}$)	0	3	3	6	1
nsy-1 (AWC$^{ON/ON}$)	0	3	3	7	1
nsy-1 (AWC$^{ON/ON}$)	0	3	3	8	1
nsy-1 (AWC$^{ON/ON}$)	0	3	3	9	1
nsy-1 (AWC$^{ON/ON}$)	0	3	3	10	1
nsy-1 (AWC$^{ON/ON}$)	0	3	3	11	1
nsy-1 (AWC$^{ON/ON}$)	0	3	3	12	0
nsy-1 (AWC$^{ON/ON}$)	0	3	3	13	1
nsy-1 (AWC$^{ON/ON}$)	0	3	3	14	1
nsy-1 (AWC$^{ON/ON}$)	0	3	4	1	1
nsy-1 (AWC$^{ON/ON}$)	0	3	4	2	0
nsy-1 (AWC$^{ON/ON}$)	0	3	4	3	1
nsy-1 (AWC$^{ON/ON}$)	0	3	4	4	1
nsy-1 (AWC$^{ON/ON}$)	0	3	4	5	1
nsy-1 (AWC$^{ON/ON}$)	0	3	4	6	1
nsy-1 (AWC$^{ON/ON}$)	0	3	4	7	1
nsy-1 (AWC$^{ON/ON}$)	0	3	4	8	1
nsy-1 (AWC$^{ON/ON}$)	0	3	4	9	0
nsy-1 (AWC$^{ON/ON}$)	0	3	4	10	1
nsy-1 (AWC$^{ON/ON}$)	0	3	4	11	1
nsy-1 (AWC$^{ON/ON}$)	0	3	4	12	1
nsy-1 (AWC$^{ON/ON}$)	0	3	4	13	0
nsy-1 (AWC$^{ON/ON}$)	0	3	4	14	0
nsy-1 (AWC$^{ON/ON}$)	0	3	4	15	1
nsy-1 (AWC$^{ON/ON}$)	0	3	4	16	1
nsy-1 (AWC$^{ON/ON}$)	3	1	1	1	0
nsy-1 (AWC$^{ON/ON}$)	3	1	1	2	0
nsy-1 (AWC$^{ON/ON}$)	3	1	1	3	0
nsy-1 (AWC$^{ON/ON}$)	3	1	1	4	1

nsy-1 (AWC$^{ON/ON}$)	3	1	1	5	0
nsy-1 (AWC$^{ON/ON}$)	3	1	1	6	0
nsy-1 (AWC$^{ON/ON}$)	3	1	1	7	0
nsy-1 (AWC$^{ON/ON}$)	3	1	1	8	1
nsy-1 (AWC$^{ON/ON}$)	3	1	1	9	1
nsy-1 (AWC$^{ON/ON}$)	3	1	1	10	0
nsy-1 (AWC$^{ON/ON}$)	3	1	1	11	0
nsy-1 (AWC$^{ON/ON}$)	3	1	1	12	0
nsy-1 (AWC$^{ON/ON}$)	3	1	1	13	1
nsy-1 (AWC$^{ON/ON}$)	3	1	1	14	0
nsy-1 (AWC$^{ON/ON}$)	3	1	2	1	0
nsy-1 (AWC$^{ON/ON}$)	3	1	2	2	0
nsy-1 (AWC$^{ON/ON}$)	3	1	2	3	0
nsy-1 (AWC$^{ON/ON}$)	3	1	2	4	0
nsy-1 (AWC$^{ON/ON}$)	3	1	2	5	0
nsy-1 (AWC$^{ON/ON}$)	3	1	2	6	0
nsy-1 (AWC$^{ON/ON}$)	3	1	2	7	0
nsy-1 (AWC$^{ON/ON}$)	3	1	2	8	1
nsy-1 (AWC$^{ON/ON}$)	3	1	2	9	1
nsy-1 (AWC$^{ON/ON}$)	3	1	2	10	0
nsy-1 (AWC$^{ON/ON}$)	3	1	2	11	0
nsy-1 (AWC$^{ON/ON}$)	3	1	2	12	0
nsy-1 (AWC$^{ON/ON}$)	3	1	2	13	1
nsy-1 (AWC$^{ON/ON}$)	3	1	2	14	0
nsy-1 (AWC$^{ON/ON}$)	3	1	2	15	1
nsy-1 (AWC$^{ON/ON}$)	3	1	2	16	0
nsy-1 (AWC$^{ON/ON}$)	3	1	2	17	1
nsy-1 (AWC$^{ON/ON}$)	3	1	2	18	0
nsy-1 (AWC$^{ON/ON}$)	3	1	2	19	0
nsy-1 (AWC$^{ON/ON}$)	3	1	2	20	1
nsy-1 (AWC$^{ON/ON}$)	3	1	2	21	0
nsy-1 (AWC$^{ON/ON}$)	3	1	2	22	1
nsy-1 (AWC$^{ON/ON}$)	3	1	2	23	1
nsy-1 (AWC$^{ON/ON}$)	3	1	2	24	0
nsy-1 (AWC$^{ON/ON}$)	3	1	2	25	0
nsy-1 (AWC$^{ON/ON}$)	3	1	2	26	1
nsy-1 (AWC$^{ON/ON}$)	3	1	2	27	0
nsy-1 (AWC$^{ON/ON}$)	3	1	2	28	0
nsy-1 (AWC$^{ON/ON}$)	3	1	2	29	0
nsy-1 (AWC$^{ON/ON}$)	3	1	2	30	0
nsy-1 (AWC$^{ON/ON}$)	3	1	2	31	0
nsy-1 (AWC$^{ON/ON}$)	3	1	2	32	0
nsy-1 (AWC$^{ON/ON}$)	3	1	2	33	0

nsy-1 ($AWC^{ON/ON}$)	3	1	3	1	1
nsy-1 ($AWC^{ON/ON}$)	3	1	3	2	1
nsy-1 ($AWC^{ON/ON}$)	3	1	3	3	0
nsy-1 ($AWC^{ON/ON}$)	3	1	3	4	0
nsy-1 ($AWC^{ON/ON}$)	3	1	3	5	0
nsy-1 ($AWC^{ON/ON}$)	3	1	3	6	0
nsy-1 ($AWC^{ON/ON}$)	3	1	3	7	1
nsy-1 ($AWC^{ON/ON}$)	3	1	3	8	1
nsy-1 ($AWC^{ON/ON}$)	3	1	3	9	0
nsy-1 ($AWC^{ON/ON}$)	3	1	3	10	1
nsy-1 ($AWC^{ON/ON}$)	3	1	3	11	0
nsy-1 ($AWC^{ON/ON}$)	3	1	3	12	0
nsy-1 ($AWC^{ON/ON}$)	3	1	3	13	0
nsy-1 ($AWC^{ON/ON}$)	3	1	3	14	1
nsy-1 ($AWC^{ON/ON}$)	3	1	3	15	0
nsy-1 ($AWC^{ON/ON}$)	3	1	3	16	0
nsy-1 ($AWC^{ON/ON}$)	3	1	3	17	0
nsy-1 ($AWC^{ON/ON}$)	3	1	3	18	1
nsy-1 ($AWC^{ON/ON}$)	3	1	3	19	0
nsy-1 ($AWC^{ON/ON}$)	3	1	3	20	1
nsy-1 ($AWC^{ON/ON}$)	3	1	3	21	0
nsy-1 ($AWC^{ON/ON}$)	3	1	3	22	1
nsy-1 ($AWC^{ON/ON}$)	3	1	3	23	1
nsy-1 ($AWC^{ON/ON}$)	3	1	3	24	1
nsy-1 ($AWC^{ON/ON}$)	3	1	3	25	0
nsy-1 ($AWC^{ON/ON}$)	3	1	3	26	0
nsy-1 ($AWC^{ON/ON}$)	3	1	3	27	0
nsy-1 ($AWC^{ON/ON}$)	3	1	3	28	0
nsy-1 ($AWC^{ON/ON}$)	3	1	3	29	0
nsy-1 ($AWC^{ON/ON}$)	3	1	3	30	0
nsy-1 ($AWC^{ON/ON}$)	3	1	3	31	0
nsy-1 ($AWC^{ON/ON}$)	3	1	3	32	0
nsy-1 ($AWC^{ON/ON}$)	3	1	3	33	0
nsy-1 ($AWC^{ON/ON}$)	3	1	3	34	0
nsy-1 ($AWC^{ON/ON}$)	3	1	3	35	0
nsy-1 ($AWC^{ON/ON}$)	3	1	3	36	0
nsy-1 ($AWC^{ON/ON}$)	3	1	3	37	0
nsy-1 ($AWC^{ON/ON}$)	3	1	3	38	0
nsy-1 ($AWC^{ON/ON}$)	3	1	3	39	1
nsy-1 ($AWC^{ON/ON}$)	3	1	3	40	0
nsy-1 ($AWC^{ON/ON}$)	3	1	3	41	0
nsy-1 ($AWC^{ON/ON}$)	3	1	3	42	0
nsy-1 ($AWC^{ON/ON}$)	3	1	3	43	0

nsy-1 (AWC$^{ON/ON}$)	3	1	3	44	1
nsy-1 (AWC$^{ON/ON}$)	3	1	4	1	1
nsy-1 (AWC$^{ON/ON}$)	3	1	4	2	0
nsy-1 (AWC$^{ON/ON}$)	3	1	4	3	0
nsy-1 (AWC$^{ON/ON}$)	3	1	4	4	0
nsy-1 (AWC$^{ON/ON}$)	3	1	4	5	0
nsy-1 (AWC$^{ON/ON}$)	3	1	4	6	0
nsy-1 (AWC$^{ON/ON}$)	3	1	4	7	0
nsy-1 (AWC$^{ON/ON}$)	3	1	4	8	1
nsy-1 (AWC$^{ON/ON}$)	3	1	4	9	0
nsy-1 (AWC$^{ON/ON}$)	3	1	4	10	1
nsy-1 (AWC$^{ON/ON}$)	3	1	4	11	0
nsy-1 (AWC$^{ON/ON}$)	3	1	4	12	0
nsy-1 (AWC$^{ON/ON}$)	3	1	4	13	1
nsy-1 (AWC$^{ON/ON}$)	3	1	4	14	0
nsy-1 (AWC$^{ON/ON}$)	3	1	4	15	0
nsy-1 (AWC$^{ON/ON}$)	3	1	4	16	0
nsy-1 (AWC$^{ON/ON}$)	3	1	4	17	1
nsy-1 (AWC$^{ON/ON}$)	3	1	4	18	0
nsy-1 (AWC$^{ON/ON}$)	3	1	4	19	1
nsy-1 (AWC$^{ON/ON}$)	3	1	4	20	1
nsy-1 (AWC$^{ON/ON}$)	3	1	4	21	1
nsy-1 (AWC$^{ON/ON}$)	3	1	4	22	0
nsy-1 (AWC$^{ON/ON}$)	3	1	4	23	0
nsy-1 (AWC$^{ON/ON}$)	3	1	4	24	0
nsy-1 (AWC$^{ON/ON}$)	3	1	4	25	0
nsy-1 (AWC$^{ON/ON}$)	3	1	4	26	0
nsy-1 (AWC$^{ON/ON}$)	3	1	4	27	0
nsy-1 (AWC$^{ON/ON}$)	3	1	4	28	0
nsy-1 (AWC$^{ON/ON}$)	3	1	4	29	0
nsy-1 (AWC$^{ON/ON}$)	3	1	4	30	1
nsy-1 (AWC$^{ON/ON}$)	3	1	4	31	1
nsy-1 (AWC$^{ON/ON}$)	3	1	4	32	0
nsy-1 (AWC$^{ON/ON}$)	3	1	4	33	0
nsy-1 (AWC$^{ON/ON}$)	3	2	1	1	0
nsy-1 (AWC$^{ON/ON}$)	3	2	1	2	0
nsy-1 (AWC$^{ON/ON}$)	3	2	1	3	0
nsy-1 (AWC$^{ON/ON}$)	3	2	1	4	0
nsy-1 (AWC$^{ON/ON}$)	3	2	1	5	0
nsy-1 (AWC$^{ON/ON}$)	3	2	1	6	0
nsy-1 (AWC$^{ON/ON}$)	3	2	1	7	0
nsy-1 (AWC$^{ON/ON}$)	3	2	1	8	0
nsy-1 (AWC$^{ON/ON}$)	3	2	1	9	0

nsy-1 (AWC$^{ON/ON}$)	3	2	1	10	0
nsy-1 (AWC$^{ON/ON}$)	3	2	1	11	0
nsy-1 (AWC$^{ON/ON}$)	3	2	1	12	0
nsy-1 (AWC$^{ON/ON}$)	3	2	1	13	0
nsy-1 (AWC$^{ON/ON}$)	3	2	1	14	0
nsy-1 (AWC$^{ON/ON}$)	3	2	1	15	0
nsy-1 (AWC$^{ON/ON}$)	3	2	1	16	0
nsy-1 (AWC$^{ON/ON}$)	3	2	1	17	0
nsy-1 (AWC$^{ON/ON}$)	3	2	1	18	1
nsy-1 (AWC$^{ON/ON}$)	3	2	1	19	1
nsy-1 (AWC$^{ON/ON}$)	3	2	1	20	0
nsy-1 (AWC$^{ON/ON}$)	3	2	1	21	1
nsy-1 (AWC$^{ON/ON}$)	3	2	1	22	0
nsy-1 (AWC$^{ON/ON}$)	3	2	1	23	0
nsy-1 (AWC$^{ON/ON}$)	3	2	1	24	0
nsy-1 (AWC$^{ON/ON}$)	3	2	1	25	0
nsy-1 (AWC$^{ON/ON}$)	3	2	1	26	0
nsy-1 (AWC$^{ON/ON}$)	3	2	1	27	1
nsy-1 (AWC$^{ON/ON}$)	3	2	1	28	0
nsy-1 (AWC$^{ON/ON}$)	3	2	1	29	0
nsy-1 (AWC$^{ON/ON}$)	3	2	1	30	0
nsy-1 (AWC$^{ON/ON}$)	3	2	1	31	1
nsy-1 (AWC$^{ON/ON}$)	3	2	1	32	0
nsy-1 (AWC$^{ON/ON}$)	3	2	1	33	1
nsy-1 (AWC$^{ON/ON}$)	3	2	2	1	0
nsy-1 (AWC$^{ON/ON}$)	3	2	2	2	0
nsy-1 (AWC$^{ON/ON}$)	3	2	2	3	0
nsy-1 (AWC$^{ON/ON}$)	3	2	2	4	0
nsy-1 (AWC$^{ON/ON}$)	3	2	2	5	0
nsy-1 (AWC$^{ON/ON}$)	3	2	2	6	0
nsy-1 (AWC$^{ON/ON}$)	3	2	2	7	0
nsy-1 (AWC$^{ON/ON}$)	3	2	2	8	1
nsy-1 (AWC$^{ON/ON}$)	3	2	2	9	0
nsy-1 (AWC$^{ON/ON}$)	3	2	2	10	0
nsy-1 (AWC$^{ON/ON}$)	3	2	2	11	0
nsy-1 (AWC$^{ON/ON}$)	3	2	2	12	1
nsy-1 (AWC$^{ON/ON}$)	3	2	2	13	1
nsy-1 (AWC$^{ON/ON}$)	3	2	2	14	0
nsy-1 (AWC$^{ON/ON}$)	3	2	2	15	0
nsy-1 (AWC$^{ON/ON}$)	3	2	2	16	0
nsy-1 (AWC$^{ON/ON}$)	3	2	2	17	0
nsy-1 (AWC$^{ON/ON}$)	3	2	2	18	0
nsy-1 (AWC$^{ON/ON}$)	3	2	2	19	0

nsy-1 (AWC$^{ON/ON}$)	3	2	2	20	0
nsy-1 (AWC$^{ON/ON}$)	3	2	2	21	0
nsy-1 (AWC$^{ON/ON}$)	3	2	2	22	0
nsy-1 (AWC$^{ON/ON}$)	3	2	2	23	0
nsy-1 (AWC$^{ON/ON}$)	3	2	2	24	0
nsy-1 (AWC$^{ON/ON}$)	3	2	2	25	0
nsy-1 (AWC$^{ON/ON}$)	3	2	2	26	1
nsy-1 (AWC$^{ON/ON}$)	3	2	2	27	0
nsy-1 (AWC$^{ON/ON}$)	3	2	2	28	0
nsy-1 (AWC$^{ON/ON}$)	3	2	2	29	1
nsy-1 (AWC$^{ON/ON}$)	3	2	2	30	0
nsy-1 (AWC$^{ON/ON}$)	3	2	2	31	0
nsy-1 (AWC$^{ON/ON}$)	3	2	2	32	0
nsy-1 (AWC$^{ON/ON}$)	3	2	2	33	0
nsy-1 (AWC$^{ON/ON}$)	3	2	2	34	0
nsy-1 (AWC$^{ON/ON}$)	3	2	2	35	0
nsy-1 (AWC$^{ON/ON}$)	3	2	2	36	0
nsy-1 (AWC$^{ON/ON}$)	3	2	2	37	0
nsy-1 (AWC$^{ON/ON}$)	3	2	2	38	0
nsy-1 (AWC$^{ON/ON}$)	3	2	2	39	0
nsy-1 (AWC$^{ON/ON}$)	3	2	2	40	0
nsy-1 (AWC$^{ON/ON}$)	3	2	3	1	0
nsy-1 (AWC$^{ON/ON}$)	3	2	3	2	0
nsy-1 (AWC$^{ON/ON}$)	3	2	3	3	0
nsy-1 (AWC$^{ON/ON}$)	3	2	3	4	0
nsy-1 (AWC$^{ON/ON}$)	3	2	3	5	0
nsy-1 (AWC$^{ON/ON}$)	3	2	3	6	0
nsy-1 (AWC$^{ON/ON}$)	3	2	3	7	0
nsy-1 (AWC$^{ON/ON}$)	3	2	3	8	0
nsy-1 (AWC$^{ON/ON}$)	3	2	3	9	0
nsy-1 (AWC$^{ON/ON}$)	3	2	3	10	1
nsy-1 (AWC$^{ON/ON}$)	3	2	3	11	1
nsy-1 (AWC$^{ON/ON}$)	3	2	3	12	0
nsy-1 (AWC$^{ON/ON}$)	3	2	3	13	0
nsy-1 (AWC$^{ON/ON}$)	3	2	3	14	0
nsy-1 (AWC$^{ON/ON}$)	3	2	3	15	0
nsy-1 (AWC$^{ON/ON}$)	3	2	3	16	0
nsy-1 (AWC$^{ON/ON}$)	3	2	3	17	0
nsy-1 (AWC$^{ON/ON}$)	3	2	3	18	1
nsy-1 (AWC$^{ON/ON}$)	3	2	3	19	0
nsy-1 (AWC$^{ON/ON}$)	3	2	3	20	0
nsy-1 (AWC$^{ON/ON}$)	3	2	3	21	1
nsy-1 (AWC$^{ON/ON}$)	3	2	3	22	0

nsy-1 (AWC$^{ON/ON}$)	3	2	3	23	0
nsy-1 (AWC$^{ON/ON}$)	3	2	3	24	0
nsy-1 (AWC$^{ON/ON}$)	3	2	3	25	0
nsy-1 (AWC$^{ON/ON}$)	3	2	3	26	1
nsy-1 (AWC$^{ON/ON}$)	3	2	4	1	1
nsy-1 (AWC$^{ON/ON}$)	3	2	4	2	0
nsy-1 (AWC$^{ON/ON}$)	3	2	4	3	0
nsy-1 (AWC$^{ON/ON}$)	3	2	4	4	0
nsy-1 (AWC$^{ON/ON}$)	3	2	4	5	0
nsy-1 (AWC$^{ON/ON}$)	3	2	4	6	0
nsy-1 (AWC$^{ON/ON}$)	3	2	4	7	0
nsy-1 (AWC$^{ON/ON}$)	3	2	4	8	0
nsy-1 (AWC$^{ON/ON}$)	3	2	4	9	0
nsy-1 (AWC$^{ON/ON}$)	3	2	4	10	1
nsy-1 (AWC$^{ON/ON}$)	3	2	4	11	0
nsy-1 (AWC$^{ON/ON}$)	3	2	4	12	0
nsy-1 (AWC$^{ON/ON}$)	3	2	4	13	0
nsy-1 (AWC$^{ON/ON}$)	3	2	4	14	1
nsy-1 (AWC$^{ON/ON}$)	3	2	4	15	0
nsy-1 (AWC$^{ON/ON}$)	3	2	4	16	0
nsy-1 (AWC$^{ON/ON}$)	3	2	4	17	0
nsy-1 (AWC$^{ON/ON}$)	3	2	4	18	0
nsy-1 (AWC$^{ON/ON}$)	3	2	4	19	0
nsy-1 (AWC$^{ON/ON}$)	3	2	4	20	0
nsy-1 (AWC$^{ON/ON}$)	3	2	4	21	1
nsy-1 (AWC$^{ON/ON}$)	3	2	4	22	0
nsy-1 (AWC$^{ON/ON}$)	3	3	1	1	0
nsy-1 (AWC$^{ON/ON}$)	3	3	1	2	0
nsy-1 (AWC$^{ON/ON}$)	3	3	1	3	0
nsy-1 (AWC$^{ON/ON}$)	3	3	1	4	1
nsy-1 (AWC$^{ON/ON}$)	3	3	1	5	0
nsy-1 (AWC$^{ON/ON}$)	3	3	1	6	1
nsy-1 (AWC$^{ON/ON}$)	3	3	1	7	0
nsy-1 (AWC$^{ON/ON}$)	3	3	1	8	0
nsy-1 (AWC$^{ON/ON}$)	3	3	1	9	0
nsy-1 (AWC$^{ON/ON}$)	3	3	1	10	0
nsy-1 (AWC$^{ON/ON}$)	3	3	1	11	0
nsy-1 (AWC$^{ON/ON}$)	3	3	1	12	0
nsy-1 (AWC$^{ON/ON}$)	3	3	1	13	1
nsy-1 (AWC$^{ON/ON}$)	3	3	1	14	0
nsy-1 (AWC$^{ON/ON}$)	3	3	1	15	0
nsy-1 (AWC$^{ON/ON}$)	3	3	2	1	0
nsy-1 (AWC$^{ON/ON}$)	3	3	2	2	0

nsy-1 (AWC$^{ON/ON}$)	3	3	2	3	0
nsy-1 (AWC$^{ON/ON}$)	3	3	2	4	0
nsy-1 (AWC$^{ON/ON}$)	3	3	2	5	0
nsy-1 (AWC$^{ON/ON}$)	3	3	2	6	0
nsy-1 (AWC$^{ON/ON}$)	3	3	2	7	0
nsy-1 (AWC$^{ON/ON}$)	3	3	2	8	0
nsy-1 (AWC$^{ON/ON}$)	3	3	2	9	0
nsy-1 (AWC$^{ON/ON}$)	3	3	2	10	0
nsy-1 (AWC$^{ON/ON}$)	3	3	2	11	0
nsy-1 (AWC$^{ON/ON}$)	3	3	2	12	0
nsy-1 (AWC$^{ON/ON}$)	3	3	2	13	0
nsy-1 (AWC$^{ON/ON}$)	3	3	2	14	1
nsy-1 (AWC$^{ON/ON}$)	3	3	2	15	0
nsy-1 (AWC$^{ON/ON}$)	3	3	2	16	0
nsy-1 (AWC$^{ON/ON}$)	3	3	2	17	0
nsy-1 (AWC$^{ON/ON}$)	3	3	2	18	0
nsy-1 (AWC$^{ON/ON}$)	3	3	2	19	1
nsy-1 (AWC$^{ON/ON}$)	3	3	2	20	0
nsy-1 (AWC$^{ON/ON}$)	3	3	3	1	0
nsy-1 (AWC$^{ON/ON}$)	3	3	3	2	0
nsy-1 (AWC$^{ON/ON}$)	3	3	3	3	0
nsy-1 (AWC$^{ON/ON}$)	3	3	3	4	1
nsy-1 (AWC$^{ON/ON}$)	3	3	3	5	0
nsy-1 (AWC$^{ON/ON}$)	3	3	3	6	0
nsy-1 (AWC$^{ON/ON}$)	3	3	3	7	0
nsy-1 (AWC$^{ON/ON}$)	3	3	3	8	0
nsy-1 (AWC$^{ON/ON}$)	3	3	3	9	0
nsy-1 (AWC$^{ON/ON}$)	3	3	3	10	0
nsy-1 (AWC$^{ON/ON}$)	3	3	3	11	0
nsy-1 (AWC$^{ON/ON}$)	3	3	3	12	0
nsy-1 (AWC$^{ON/ON}$)	3	3	3	13	0
nsy-1 (AWC$^{ON/ON}$)	3	3	3	14	0
nsy-1 (AWC$^{ON/ON}$)	3	3	3	15	0
nsy-1 (AWC$^{ON/ON}$)	3	3	3	16	0
nsy-1 (AWC$^{ON/ON}$)	3	3	3	17	1
nsy-1 (AWC$^{ON/ON}$)	3	3	3	18	0
nsy-1 (AWC$^{ON/ON}$)	3	3	3	19	0
nsy-1 (AWC$^{ON/ON}$)	3	3	3	20	0
nsy-1 (AWC$^{ON/ON}$)	3	3	3	21	1
nsy-1 (AWC$^{ON/ON}$)	3	3	3	22	0
nsy-1 (AWC$^{ON/ON}$)	3	3	3	23	0
nsy-1 (AWC$^{ON/ON}$)	3	3	3	24	0
nsy-1 (AWC$^{ON/ON}$)	3	3	3	25	0

nsy-1 (AWC$^{ON/ON}$)	3	3	3	26	0
nsy-1 (AWC$^{ON/ON}$)	3	3	3	27	0
nsy-1 (AWC$^{ON/ON}$)	3	3	3	28	0
nsy-1 (AWC$^{ON/ON}$)	3	3	3	29	0
nsy-1 (AWC$^{ON/ON}$)	3	3	3	30	0
nsy-1 (AWC$^{ON/ON}$)	3	3	3	31	1
nsy-1 (AWC$^{ON/ON}$)	3	3	4	1	0
nsy-1 (AWC$^{ON/ON}$)	3	3	4	2	0
nsy-1 (AWC$^{ON/ON}$)	3	3	4	3	1
nsy-1 (AWC$^{ON/ON}$)	3	3	4	4	0
nsy-1 (AWC$^{ON/ON}$)	3	3	4	5	0
nsy-1 (AWC$^{ON/ON}$)	3	3	4	6	0
nsy-1 (AWC$^{ON/ON}$)	3	3	4	7	0
nsy-1 (AWC$^{ON/ON}$)	3	3	4	8	0
nsy-1 (AWC$^{ON/ON}$)	3	3	4	9	0
nsy-1 (AWC$^{ON/ON}$)	3	3	4	10	1
nsy-1 (AWC$^{ON/ON}$)	3	3	4	11	0
nsy-1 (AWC$^{ON/ON}$)	3	3	4	12	0
nsy-1 (AWC$^{ON/ON}$)	3	3	4	13	0
nsy-1 (AWC$^{ON/ON}$)	3	3	4	14	0
nsy-1 (AWC$^{ON/ON}$)	3	3	4	15	0
nsy-1 (AWC$^{ON/ON}$)	3	3	4	16	0
nsy-1 (AWC$^{ON/ON}$)	3	3	4	17	0
nsy-1 (AWC$^{ON/ON}$)	3	3	4	18	0
nsy-1 (AWC$^{ON/ON}$)	3	3	4	19	1
nsy-1 (AWC$^{ON/ON}$)	3	3	4	20	0
nsy-1 (AWC$^{ON/ON}$)	3	3	4	21	0
nsy-1 (AWC$^{ON/ON}$)	3	3	4	22	1
nsy-1 (AWC$^{ON/ON}$)	3	3	4	23	0
nsy-1 (AWC$^{ON/ON}$)	3	3	4	24	1
nsy-1 (AWC$^{ON/ON}$)	3	3	4	25	0
nsy-1 (AWC$^{ON/ON}$)	3	3	4	26	0
nsy-5/inx-19 (AWC$^{OFF/OFF}$)	0	1	1	1	1
nsy-5/inx-19 (AWC$^{OFF/OFF}$)	0	1	1	2	1
nsy-5/inx-19 (AWC$^{OFF/OFF}$)	0	1	1	3	1
nsy-5/inx-19 (AWC$^{OFF/OFF}$)	0	1	1	4	1
nsy-5/inx-19 (AWC$^{OFF/OFF}$)	0	1	1	5	1
nsy-5/inx-19 (AWC$^{OFF/OFF}$)	0	1	1	6	1
nsy-5/inx-19 (AWC$^{OFF/OFF}$)	0	1	1	7	0
nsy-5/inx-19 (AWC$^{OFF/OFF}$)	0	1	1	8	1
nsy-5/inx-19 (AWC$^{OFF/OFF}$)	0	1	1	9	1
nsy-5/inx-19 (AWC$^{OFF/OFF}$)	0	1	1	10	1

nsy-5/inx-19 ($AWC^{OFF/OFF}$)	0	1	1	11	1
nsy-5/inx-19 ($AWC^{OFF/OFF}$)	0	1	2	1	1
nsy-5/inx-19 ($AWC^{OFF/OFF}$)	0	1	2	2	1
nsy-5/inx-19 ($AWC^{OFF/OFF}$)	0	1	2	3	1
nsy-5/inx-19 ($AWC^{OFF/OFF}$)	0	1	2	4	1
nsy-5/inx-19 ($AWC^{OFF/OFF}$)	0	1	2	5	1
nsy-5/inx-19 ($AWC^{OFF/OFF}$)	0	1	2	6	1
nsy-5/inx-19 ($AWC^{OFF/OFF}$)	0	1	2	7	1
nsy-5/inx-19 ($AWC^{OFF/OFF}$)	0	1	2	8	1
nsy-5/inx-19 ($AWC^{OFF/OFF}$)	0	1	2	9	1
nsy-5/inx-19 ($AWC^{OFF/OFF}$)	0	1	2	10	1
nsy-5/inx-19 ($AWC^{OFF/OFF}$)	0	1	2	11	0
nsy-5/inx-19 ($AWC^{OFF/OFF}$)	0	1	2	12	1
nsy-5/inx-19 ($AWC^{OFF/OFF}$)	0	1	2	13	1
nsy-5/inx-19 ($AWC^{OFF/OFF}$)	0	1	2	14	1
nsy-5/inx-19 ($AWC^{OFF/OFF}$)	0	1	2	15	1
nsy-5/inx-19 ($AWC^{OFF/OFF}$)	0	1	2	16	1
nsy-5/inx-19 ($AWC^{OFF/OFF}$)	0	1	2	17	1
nsy-5/inx-19 ($AWC^{OFF/OFF}$)	0	1	2	18	1
nsy-5/inx-19 ($AWC^{OFF/OFF}$)	0	1	2	19	0
nsy-5/inx-19 ($AWC^{OFF/OFF}$)	0	1	2	20	1
nsy-5/inx-19 ($AWC^{OFF/OFF}$)	0	1	2	21	1
nsy-5/inx-19 ($AWC^{OFF/OFF}$)	0	1	2	22	1
nsy-5/inx-19 ($AWC^{OFF/OFF}$)	0	1	2	23	1
nsy-5/inx-19 ($AWC^{OFF/OFF}$)	0	1	2	24	1
nsy-5/inx-19 ($AWC^{OFF/OFF}$)	0	1	2	25	1
nsy-5/inx-19 ($AWC^{OFF/OFF}$)	0	1	2	26	0
nsy-5/inx-19 ($AWC^{OFF/OFF}$)	0	1	2	27	0
nsy-5/inx-19 ($AWC^{OFF/OFF}$)	0	1	2	28	0
nsy-5/inx-19 ($AWC^{OFF/OFF}$)	0	1	2	29	1
nsy-5/inx-19 ($AWC^{OFF/OFF}$)	0	1	2	30	1
nsy-5/inx-19 ($AWC^{OFF/OFF}$)	0	1	3	1	0
nsy-5/inx-19 ($AWC^{OFF/OFF}$)	0	1	3	2	1
nsy-5/inx-19 ($AWC^{OFF/OFF}$)	0	1	3	3	1
nsy-5/inx-19 ($AWC^{OFF/OFF}$)	0	1	3	4	1
nsy-5/inx-19 ($AWC^{OFF/OFF}$)	0	1	3	5	1
nsy-5/inx-19 ($AWC^{OFF/OFF}$)	0	1	3	6	1
nsy-5/inx-19 ($AWC^{OFF/OFF}$)	0	1	3	7	1
nsy-5/inx-19 ($AWC^{OFF/OFF}$)	0	1	3	8	1
nsy-5/inx-19 ($AWC^{OFF/OFF}$)	0	1	3	9	1
nsy-5/inx-19 ($AWC^{OFF/OFF}$)	0	1	3	10	1
nsy-5/inx-19 ($AWC^{OFF/OFF}$)	0	1	3	11	1
nsy-5/inx-19 ($AWC^{OFF/OFF}$)	0	1	3	12	1

nsy-5/inx-19 (AWC$^{OFF/OFF}$)	0	1	3	13	1
nsy-5/inx-19 (AWC$^{OFF/OFF}$)	0	1	3	14	1
nsy-5/inx-19 (AWC$^{OFF/OFF}$)	0	1	3	15	1
nsy-5/inx-19 (AWC$^{OFF/OFF}$)	0	1	3	16	1
nsy-5/inx-19 (AWC$^{OFF/OFF}$)	0	1	3	17	1
nsy-5/inx-19 (AWC$^{OFF/OFF}$)	0	1	3	18	1
nsy-5/inx-19 (AWC$^{OFF/OFF}$)	0	1	3	19	1
nsy-5/inx-19 (AWC$^{OFF/OFF}$)	0	1	3	20	1
nsy-5/inx-19 (AWC$^{OFF/OFF}$)	0	1	3	21	1
nsy-5/inx-19 (AWC$^{OFF/OFF}$)	0	1	3	22	1
nsy-5/inx-19 (AWC$^{OFF/OFF}$)	0	1	3	23	1
nsy-5/inx-19 (AWC$^{OFF/OFF}$)	0	1	3	24	0
nsy-5/inx-19 (AWC$^{OFF/OFF}$)	0	1	3	25	1
nsy-5/inx-19 (AWC$^{OFF/OFF}$)	0	1	3	26	1
nsy-5/inx-19 (AWC$^{OFF/OFF}$)	0	1	4	1	1
nsy-5/inx-19 (AWC$^{OFF/OFF}$)	0	1	4	2	1
nsy-5/inx-19 (AWC$^{OFF/OFF}$)	0	1	4	3	1
nsy-5/inx-19 (AWC$^{OFF/OFF}$)	0	1	4	4	1
nsy-5/inx-19 (AWC$^{OFF/OFF}$)	0	1	4	5	1
nsy-5/inx-19 (AWC$^{OFF/OFF}$)	0	1	4	6	0
nsy-5/inx-19 (AWC$^{OFF/OFF}$)	0	1	4	7	1
nsy-5/inx-19 (AWC$^{OFF/OFF}$)	0	1	4	8	1
nsy-5/inx-19 (AWC$^{OFF/OFF}$)	0	1	4	9	1
nsy-5/inx-19 (AWC$^{OFF/OFF}$)	0	1	4	10	1
nsy-5/inx-19 (AWC$^{OFF/OFF}$)	0	1	4	11	1
nsy-5/inx-19 (AWC$^{OFF/OFF}$)	0	1	4	12	1
nsy-5/inx-19 (AWC$^{OFF/OFF}$)	0	1	4	13	1
nsy-5/inx-19 (AWC$^{OFF/OFF}$)	0	1	4	14	1
nsy-5/inx-19 (AWC$^{OFF/OFF}$)	0	1	4	15	1
nsy-5/inx-19 (AWC$^{OFF/OFF}$)	0	1	4	16	1
nsy-5/inx-19 (AWC$^{OFF/OFF}$)	0	1	4	17	1
nsy-5/inx-19 (AWC$^{OFF/OFF}$)	0	1	4	18	1
nsy-5/inx-19 (AWC$^{OFF/OFF}$)	0	1	4	19	1
nsy-5/inx-19 (AWC$^{OFF/OFF}$)	0	1	4	20	1
nsy-5/inx-19 (AWC$^{OFF/OFF}$)	0	1	4	21	1
nsy-5/inx-19 (AWC$^{OFF/OFF}$)	0	1	4	22	1
nsy-5/inx-19 (AWC$^{OFF/OFF}$)	0	1	4	23	1
nsy-5/inx-19 (AWC$^{OFF/OFF}$)	0	1	4	24	1
nsy-5/inx-19 (AWC$^{OFF/OFF}$)	0	1	4	25	1
nsy-5/inx-19 (AWC$^{OFF/OFF}$)	0	1	4	26	1
nsy-5/inx-19 (AWC$^{OFF/OFF}$)	0	1	4	27	1
nsy-5/inx-19 (AWC$^{OFF/OFF}$)	0	1	4	28	0
nsy-5/inx-19 (AWC$^{OFF/OFF}$)	0	2	1	1	1

nsy-5/inx-19 ($AWC^{OFF/OFF}$)	0	2	1	2	1
nsy-5/inx-19 ($AWC^{OFF/OFF}$)	0	2	1	3	0
nsy-5/inx-19 ($AWC^{OFF/OFF}$)	0	2	1	4	0
nsy-5/inx-19 ($AWC^{OFF/OFF}$)	0	2	1	5	0
nsy-5/inx-19 ($AWC^{OFF/OFF}$)	0	2	1	6	0
nsy-5/inx-19 ($AWC^{OFF/OFF}$)	0	2	1	7	1
nsy-5/inx-19 ($AWC^{OFF/OFF}$)	0	2	1	8	1
nsy-5/inx-19 ($AWC^{OFF/OFF}$)	0	2	1	9	0
nsy-5/inx-19 ($AWC^{OFF/OFF}$)	0	2	1	10	1
nsy-5/inx-19 ($AWC^{OFF/OFF}$)	0	2	1	11	1
nsy-5/inx-19 ($AWC^{OFF/OFF}$)	0	2	1	12	1
nsy-5/inx-19 ($AWC^{OFF/OFF}$)	0	2	1	13	1
nsy-5/inx-19 ($AWC^{OFF/OFF}$)	0	2	1	14	1
nsy-5/inx-19 ($AWC^{OFF/OFF}$)	0	2	1	15	1
nsy-5/inx-19 ($AWC^{OFF/OFF}$)	0	2	1	16	1
nsy-5/inx-19 ($AWC^{OFF/OFF}$)	0	2	1	17	1
nsy-5/inx-19 ($AWC^{OFF/OFF}$)	0	2	2	1	1
nsy-5/inx-19 ($AWC^{OFF/OFF}$)	0	2	2	2	1
nsy-5/inx-19 ($AWC^{OFF/OFF}$)	0	2	2	3	1
nsy-5/inx-19 ($AWC^{OFF/OFF}$)	0	2	2	4	1
nsy-5/inx-19 ($AWC^{OFF/OFF}$)	0	2	2	5	1
nsy-5/inx-19 ($AWC^{OFF/OFF}$)	0	2	2	6	1
nsy-5/inx-19 ($AWC^{OFF/OFF}$)	0	2	2	7	1
nsy-5/inx-19 ($AWC^{OFF/OFF}$)	0	2	2	8	1
nsy-5/inx-19 ($AWC^{OFF/OFF}$)	0	2	2	9	1
nsy-5/inx-19 ($AWC^{OFF/OFF}$)	0	2	2	10	1
nsy-5/inx-19 ($AWC^{OFF/OFF}$)	0	2	2	11	1
nsy-5/inx-19 ($AWC^{OFF/OFF}$)	0	2	2	12	0
nsy-5/inx-19 ($AWC^{OFF/OFF}$)	0	2	2	13	1
nsy-5/inx-19 ($AWC^{OFF/OFF}$)	0	2	2	14	1
nsy-5/inx-19 ($AWC^{OFF/OFF}$)	0	2	2	15	1
nsy-5/inx-19 ($AWC^{OFF/OFF}$)	0	2	2	16	1
nsy-5/inx-19 ($AWC^{OFF/OFF}$)	0	2	2	17	0
nsy-5/inx-19 ($AWC^{OFF/OFF}$)	0	2	2	18	0
nsy-5/inx-19 ($AWC^{OFF/OFF}$)	0	2	2	19	1
nsy-5/inx-19 ($AWC^{OFF/OFF}$)	0	2	2	20	0
nsy-5/inx-19 ($AWC^{OFF/OFF}$)	0	2	2	21	0
nsy-5/inx-19 ($AWC^{OFF/OFF}$)	0	2	2	22	1
nsy-5/inx-19 ($AWC^{OFF/OFF}$)	0	2	3	1	1
nsy-5/inx-19 ($AWC^{OFF/OFF}$)	0	2	3	2	1
nsy-5/inx-19 ($AWC^{OFF/OFF}$)	0	2	3	3	1
nsy-5/inx-19 ($AWC^{OFF/OFF}$)	0	2	3	4	1
nsy-5/inx-19 ($AWC^{OFF/OFF}$)	0	2	3	5	1

nsy-5/inx-19 ($AWC^{OFF/OFF}$)	0	2	3	6	0
nsy-5/inx-19 ($AWC^{OFF/OFF}$)	0	2	3	7	1
nsy-5/inx-19 ($AWC^{OFF/OFF}$)	0	2	3	8	1
nsy-5/inx-19 ($AWC^{OFF/OFF}$)	0	2	3	9	0
nsy-5/inx-19 ($AWC^{OFF/OFF}$)	0	2	3	10	0
nsy-5/inx-19 ($AWC^{OFF/OFF}$)	0	2	3	11	1
nsy-5/inx-19 ($AWC^{OFF/OFF}$)	0	2	3	12	1
nsy-5/inx-19 ($AWC^{OFF/OFF}$)	0	2	3	13	1
nsy-5/inx-19 ($AWC^{OFF/OFF}$)	0	2	3	14	0
nsy-5/inx-19 ($AWC^{OFF/OFF}$)	0	2	3	15	0
nsy-5/inx-19 ($AWC^{OFF/OFF}$)	0	2	3	16	1
nsy-5/inx-19 ($AWC^{OFF/OFF}$)	0	2	3	17	1
nsy-5/inx-19 ($AWC^{OFF/OFF}$)	0	2	3	18	1
nsy-5/inx-19 ($AWC^{OFF/OFF}$)	0	2	3	19	0
nsy-5/inx-19 ($AWC^{OFF/OFF}$)	0	2	4	1	1
nsy-5/inx-19 ($AWC^{OFF/OFF}$)	0	2	4	2	1
nsy-5/inx-19 ($AWC^{OFF/OFF}$)	0	2	4	3	1
nsy-5/inx-19 ($AWC^{OFF/OFF}$)	0	2	4	4	1
nsy-5/inx-19 ($AWC^{OFF/OFF}$)	0	2	4	5	1
nsy-5/inx-19 ($AWC^{OFF/OFF}$)	0	2	4	6	1
nsy-5/inx-19 ($AWC^{OFF/OFF}$)	0	2	4	7	1
nsy-5/inx-19 ($AWC^{OFF/OFF}$)	0	2	4	8	1
nsy-5/inx-19 ($AWC^{OFF/OFF}$)	0	2	4	9	1
nsy-5/inx-19 ($AWC^{OFF/OFF}$)	0	2	4	10	1
nsy-5/inx-19 ($AWC^{OFF/OFF}$)	0	2	4	11	1
nsy-5/inx-19 ($AWC^{OFF/OFF}$)	0	2	4	12	1
nsy-5/inx-19 ($AWC^{OFF/OFF}$)	0	2	4	13	1
nsy-5/inx-19 ($AWC^{OFF/OFF}$)	0	2	4	14	1
nsy-5/inx-19 ($AWC^{OFF/OFF}$)	0	2	4	15	1
nsy-5/inx-19 ($AWC^{OFF/OFF}$)	0	2	4	16	1
nsy-5/inx-19 ($AWC^{OFF/OFF}$)	0	2	4	17	1
nsy-5/inx-19 ($AWC^{OFF/OFF}$)	0	2	4	18	1
nsy-5/inx-19 ($AWC^{OFF/OFF}$)	0	2	4	19	1
nsy-5/inx-19 ($AWC^{OFF/OFF}$)	0	2	4	20	0
nsy-5/inx-19 ($AWC^{OFF/OFF}$)	0	2	4	21	1
nsy-5/inx-19 ($AWC^{OFF/OFF}$)	0	2	4	22	1
nsy-5/inx-19 ($AWC^{OFF/OFF}$)	0	2	4	23	1
nsy-5/inx-19 ($AWC^{OFF/OFF}$)	0	2	4	24	0
nsy-5/inx-19 ($AWC^{OFF/OFF}$)	0	2	4	25	0
nsy-5/inx-19 ($AWC^{OFF/OFF}$)	0	3	1	1	1
nsy-5/inx-19 ($AWC^{OFF/OFF}$)	0	3	1	2	1
nsy-5/inx-19 ($AWC^{OFF/OFF}$)	0	3	1	3	0
nsy-5/inx-19 ($AWC^{OFF/OFF}$)	0	3	1	4	1

nsy-5/inx-19 (AWC$^{OFF/OFF}$)	0	3	1	5	0
nsy-5/inx-19 (AWC$^{OFF/OFF}$)	0	3	1	6	0
nsy-5/inx-19 (AWC$^{OFF/OFF}$)	0	3	1	7	0
nsy-5/inx-19 (AWC$^{OFF/OFF}$)	0	3	1	8	1
nsy-5/inx-19 (AWC$^{OFF/OFF}$)	0	3	1	9	0
nsy-5/inx-19 (AWC$^{OFF/OFF}$)	0	3	1	10	1
nsy-5/inx-19 (AWC$^{OFF/OFF}$)	0	3	1	11	1
nsy-5/inx-19 (AWC$^{OFF/OFF}$)	0	3	1	12	1
nsy-5/inx-19 (AWC$^{OFF/OFF}$)	0	3	1	13	0
nsy-5/inx-19 (AWC$^{OFF/OFF}$)	0	3	1	14	0
nsy-5/inx-19 (AWC$^{OFF/OFF}$)	0	3	1	15	0
nsy-5/inx-19 (AWC$^{OFF/OFF}$)	0	3	1	16	1
nsy-5/inx-19 (AWC$^{OFF/OFF}$)	0	3	1	17	0
nsy-5/inx-19 (AWC$^{OFF/OFF}$)	0	3	1	18	0
nsy-5/inx-19 (AWC$^{OFF/OFF}$)	0	3	1	19	1
nsy-5/inx-19 (AWC$^{OFF/OFF}$)	0	3	1	20	1
nsy-5/inx-19 (AWC$^{OFF/OFF}$)	0	3	1	21	1
nsy-5/inx-19 (AWC$^{OFF/OFF}$)	0	3	1	22	0
nsy-5/inx-19 (AWC$^{OFF/OFF}$)	0	3	1	23	1
nsy-5/inx-19 (AWC$^{OFF/OFF}$)	0	3	1	24	0
nsy-5/inx-19 (AWC$^{OFF/OFF}$)	0	3	2	1	1
nsy-5/inx-19 (AWC$^{OFF/OFF}$)	0	3	2	2	1
nsy-5/inx-19 (AWC$^{OFF/OFF}$)	0	3	2	3	1
nsy-5/inx-19 (AWC$^{OFF/OFF}$)	0	3	2	4	1
nsy-5/inx-19 (AWC$^{OFF/OFF}$)	0	3	2	5	1
nsy-5/inx-19 (AWC$^{OFF/OFF}$)	0	3	2	6	1
nsy-5/inx-19 (AWC$^{OFF/OFF}$)	0	3	2	7	0
nsy-5/inx-19 (AWC$^{OFF/OFF}$)	0	3	2	8	0
nsy-5/inx-19 (AWC$^{OFF/OFF}$)	0	3	2	9	1
nsy-5/inx-19 (AWC$^{OFF/OFF}$)	0	3	2	10	1
nsy-5/inx-19 (AWC$^{OFF/OFF}$)	0	3	2	11	1
nsy-5/inx-19 (AWC$^{OFF/OFF}$)	0	3	2	12	1
nsy-5/inx-19 (AWC$^{OFF/OFF}$)	0	3	2	13	1
nsy-5/inx-19 (AWC$^{OFF/OFF}$)	0	3	2	14	0
nsy-5/inx-19 (AWC$^{OFF/OFF}$)	0	3	2	15	0
nsy-5/inx-19 (AWC$^{OFF/OFF}$)	0	3	2	16	1
nsy-5/inx-19 (AWC$^{OFF/OFF}$)	0	3	2	17	0
nsy-5/inx-19 (AWC$^{OFF/OFF}$)	0	3	2	18	0
nsy-5/inx-19 (AWC$^{OFF/OFF}$)	0	3	2	19	1
nsy-5/inx-19 (AWC$^{OFF/OFF}$)	0	3	2	20	1
nsy-5/inx-19 (AWC$^{OFF/OFF}$)	0	3	2	21	0
nsy-5/inx-19 (AWC$^{OFF/OFF}$)	0	3	2	22	1
nsy-5/inx-19 (AWC$^{OFF/OFF}$)	0	3	3	1	1

nsy-5/inx-19 (AWC$^{OFF/OFF}$)	0	3	3	2	1
nsy-5/inx-19 (AWC$^{OFF/OFF}$)	0	3	3	3	0
nsy-5/inx-19 (AWC$^{OFF/OFF}$)	0	3	3	4	1
nsy-5/inx-19 (AWC$^{OFF/OFF}$)	0	3	3	5	1
nsy-5/inx-19 (AWC$^{OFF/OFF}$)	0	3	3	6	1
nsy-5/inx-19 (AWC$^{OFF/OFF}$)	0	3	3	7	1
nsy-5/inx-19 (AWC$^{OFF/OFF}$)	0	3	3	8	1
nsy-5/inx-19 (AWC$^{OFF/OFF}$)	0	3	3	9	0
nsy-5/inx-19 (AWC$^{OFF/OFF}$)	0	3	3	10	0
nsy-5/inx-19 (AWC$^{OFF/OFF}$)	0	3	3	11	1
nsy-5/inx-19 (AWC$^{OFF/OFF}$)	0	3	3	12	1
nsy-5/inx-19 (AWC$^{OFF/OFF}$)	0	3	3	13	1
nsy-5/inx-19 (AWC$^{OFF/OFF}$)	0	3	3	14	0
nsy-5/inx-19 (AWC$^{OFF/OFF}$)	0	3	3	15	1
nsy-5/inx-19 (AWC$^{OFF/OFF}$)	0	3	3	16	0
nsy-5/inx-19 (AWC$^{OFF/OFF}$)	0	3	3	17	1
nsy-5/inx-19 (AWC$^{OFF/OFF}$)	0	3	3	18	0
nsy-5/inx-19 (AWC$^{OFF/OFF}$)	0	3	3	19	1
nsy-5/inx-19 (AWC$^{OFF/OFF}$)	0	3	3	20	1
nsy-5/inx-19 (AWC$^{OFF/OFF}$)	0	3	4	1	0
nsy-5/inx-19 (AWC$^{OFF/OFF}$)	0	3	4	2	1
nsy-5/inx-19 (AWC$^{OFF/OFF}$)	0	3	4	3	1
nsy-5/inx-19 (AWC$^{OFF/OFF}$)	0	3	4	4	1
nsy-5/inx-19 (AWC$^{OFF/OFF}$)	0	3	4	5	1
nsy-5/inx-19 (AWC$^{OFF/OFF}$)	0	3	4	6	1
nsy-5/inx-19 (AWC$^{OFF/OFF}$)	0	3	4	7	1
nsy-5/inx-19 (AWC$^{OFF/OFF}$)	0	3	4	8	1
nsy-5/inx-19 (AWC$^{OFF/OFF}$)	0	3	4	9	1
nsy-5/inx-19 (AWC$^{OFF/OFF}$)	0	3	4	10	1
nsy-5/inx-19 (AWC$^{OFF/OFF}$)	0	3	4	11	1
nsy-5/inx-19 (AWC$^{OFF/OFF}$)	0	3	4	12	1
nsy-5/inx-19 (AWC$^{OFF/OFF}$)	0	3	4	13	1
nsy-5/inx-19 (AWC$^{OFF/OFF}$)	0	3	4	14	0
nsy-5/inx-19 (AWC$^{OFF/OFF}$)	0	3	4	15	0
nsy-5/inx-19 (AWC$^{OFF/OFF}$)	0	3	4	16	1
nsy-5/inx-19 (AWC$^{OFF/OFF}$)	0	3	4	17	0
nsy-5/inx-19 (AWC$^{OFF/OFF}$)	0	3	4	18	1
nsy-5/inx-19 (AWC$^{OFF/OFF}$)	0	3	4	19	0
nsy-5/inx-19 (AWC$^{OFF/OFF}$)	3	1	1	1	0
nsy-5/inx-19 (AWC$^{OFF/OFF}$)	3	1	1	2	0
nsy-5/inx-19 (AWC$^{OFF/OFF}$)	3	1	1	3	0
nsy-5/inx-19 (AWC$^{OFF/OFF}$)	3	1	1	4	0
nsy-5/inx-19 (AWC$^{OFF/OFF}$)	3	1	1	5	0

nsy-5/inx-19 ($AWC^{OFF/OFF}$)	3	1	1	6	0
nsy-5/inx-19 ($AWC^{OFF/OFF}$)	3	1	1	7	1
nsy-5/inx-19 ($AWC^{OFF/OFF}$)	3	1	1	8	0
nsy-5/inx-19 ($AWC^{OFF/OFF}$)	3	1	1	9	1
nsy-5/inx-19 ($AWC^{OFF/OFF}$)	3	1	1	10	0
nsy-5/inx-19 ($AWC^{OFF/OFF}$)	3	1	1	11	1
nsy-5/inx-19 ($AWC^{OFF/OFF}$)	3	1	1	12	1
nsy-5/inx-19 ($AWC^{OFF/OFF}$)	3	1	1	13	1
nsy-5/inx-19 ($AWC^{OFF/OFF}$)	3	1	2	1	1
nsy-5/inx-19 ($AWC^{OFF/OFF}$)	3	1	2	2	1
nsy-5/inx-19 ($AWC^{OFF/OFF}$)	3	1	2	3	1
nsy-5/inx-19 ($AWC^{OFF/OFF}$)	3	1	2	4	1
nsy-5/inx-19 ($AWC^{OFF/OFF}$)	3	1	2	5	1
nsy-5/inx-19 ($AWC^{OFF/OFF}$)	3	1	2	6	1
nsy-5/inx-19 ($AWC^{OFF/OFF}$)	3	1	2	7	1
nsy-5/inx-19 ($AWC^{OFF/OFF}$)	3	1	2	8	1
nsy-5/inx-19 ($AWC^{OFF/OFF}$)	3	1	2	9	1
nsy-5/inx-19 ($AWC^{OFF/OFF}$)	3	1	2	10	1
nsy-5/inx-19 ($AWC^{OFF/OFF}$)	3	1	2	11	1
nsy-5/inx-19 ($AWC^{OFF/OFF}$)	3	1	2	12	1
nsy-5/inx-19 ($AWC^{OFF/OFF}$)	3	1	2	13	1
nsy-5/inx-19 ($AWC^{OFF/OFF}$)	3	1	2	14	1
nsy-5/inx-19 ($AWC^{OFF/OFF}$)	3	1	2	15	1
nsy-5/inx-19 ($AWC^{OFF/OFF}$)	3	1	2	16	0
nsy-5/inx-19 ($AWC^{OFF/OFF}$)	3	1	2	17	1
nsy-5/inx-19 ($AWC^{OFF/OFF}$)	3	1	2	18	1
nsy-5/inx-19 ($AWC^{OFF/OFF}$)	3	1	2	19	1
nsy-5/inx-19 ($AWC^{OFF/OFF}$)	3	1	2	20	0
nsy-5/inx-19 ($AWC^{OFF/OFF}$)	3	1	2	21	1
nsy-5/inx-19 ($AWC^{OFF/OFF}$)	3	1	3	1	1
nsy-5/inx-19 ($AWC^{OFF/OFF}$)	3	1	3	2	1
nsy-5/inx-19 ($AWC^{OFF/OFF}$)	3	1	3	3	1
nsy-5/inx-19 ($AWC^{OFF/OFF}$)	3	1	3	4	1
nsy-5/inx-19 ($AWC^{OFF/OFF}$)	3	1	3	5	0
nsy-5/inx-19 ($AWC^{OFF/OFF}$)	3	1	3	6	1
nsy-5/inx-19 ($AWC^{OFF/OFF}$)	3	1	3	7	1
nsy-5/inx-19 ($AWC^{OFF/OFF}$)	3	1	3	8	0
nsy-5/inx-19 ($AWC^{OFF/OFF}$)	3	1	3	9	1
nsy-5/inx-19 ($AWC^{OFF/OFF}$)	3	1	3	10	1
nsy-5/inx-19 ($AWC^{OFF/OFF}$)	3	1	3	11	1
nsy-5/inx-19 ($AWC^{OFF/OFF}$)	3	1	3	12	0
nsy-5/inx-19 ($AWC^{OFF/OFF}$)	3	1	3	13	1
nsy-5/inx-19 ($AWC^{OFF/OFF}$)	3	1	3	14	0

nsy-5/inx-19 (AWC$^{OFF/OFF}$)	3	1	3	15	1
nsy-5/inx-19 (AWC$^{OFF/OFF}$)	3	1	3	16	1
nsy-5/inx-19 (AWC$^{OFF/OFF}$)	3	1	3	17	1
nsy-5/inx-19 (AWC$^{OFF/OFF}$)	3	1	3	18	1
nsy-5/inx-19 (AWC$^{OFF/OFF}$)	3	1	3	19	1
nsy-5/inx-19 (AWC$^{OFF/OFF}$)	3	1	4	1	0
nsy-5/inx-19 (AWC$^{OFF/OFF}$)	3	1	4	2	1
nsy-5/inx-19 (AWC$^{OFF/OFF}$)	3	1	4	3	1
nsy-5/inx-19 (AWC$^{OFF/OFF}$)	3	1	4	4	1
nsy-5/inx-19 (AWC$^{OFF/OFF}$)	3	1	4	5	1
nsy-5/inx-19 (AWC$^{OFF/OFF}$)	3	1	4	6	1
nsy-5/inx-19 (AWC$^{OFF/OFF}$)	3	1	4	7	1
nsy-5/inx-19 (AWC$^{OFF/OFF}$)	3	1	4	8	1
nsy-5/inx-19 (AWC$^{OFF/OFF}$)	3	1	4	9	1
nsy-5/inx-19 (AWC$^{OFF/OFF}$)	3	1	4	10	0
nsy-5/inx-19 (AWC$^{OFF/OFF}$)	3	1	4	11	1
nsy-5/inx-19 (AWC$^{OFF/OFF}$)	3	1	4	12	1
nsy-5/inx-19 (AWC$^{OFF/OFF}$)	3	1	4	13	1
nsy-5/inx-19 (AWC$^{OFF/OFF}$)	3	1	4	14	1
nsy-5/inx-19 (AWC$^{OFF/OFF}$)	3	1	4	15	1
nsy-5/inx-19 (AWC$^{OFF/OFF}$)	3	1	4	16	0
nsy-5/inx-19 (AWC$^{OFF/OFF}$)	3	1	4	17	1
nsy-5/inx-19 (AWC$^{OFF/OFF}$)	3	1	4	18	0
nsy-5/inx-19 (AWC$^{OFF/OFF}$)	3	1	4	19	0
nsy-5/inx-19 (AWC$^{OFF/OFF}$)	3	1	4	20	1
nsy-5/inx-19 (AWC$^{OFF/OFF}$)	3	1	4	21	1
nsy-5/inx-19 (AWC$^{OFF/OFF}$)	3	1	4	22	1
nsy-5/inx-19 (AWC$^{OFF/OFF}$)	3	1	4	23	1
nsy-5/inx-19 (AWC$^{OFF/OFF}$)	3	1	4	24	0
nsy-5/inx-19 (AWC$^{OFF/OFF}$)	3	1	4	25	1
nsy-5/inx-19 (AWC$^{OFF/OFF}$)	3	1	4	26	1
nsy-5/inx-19 (AWC$^{OFF/OFF}$)	3	1	4	27	1
nsy-5/inx-19 (AWC$^{OFF/OFF}$)	3	1	4	28	1
nsy-5/inx-19 (AWC$^{OFF/OFF}$)	3	1	4	29	1
nsy-5/inx-19 (AWC$^{OFF/OFF}$)	3	1	4	30	1
nsy-5/inx-19 (AWC$^{OFF/OFF}$)	3	1	4	31	1
nsy-5/inx-19 (AWC$^{OFF/OFF}$)	3	1	4	32	1
nsy-5/inx-19 (AWC$^{OFF/OFF}$)	3	2	1	1	0
nsy-5/inx-19 (AWC$^{OFF/OFF}$)	3	2	1	2	0
nsy-5/inx-19 (AWC$^{OFF/OFF}$)	3	2	1	3	0
nsy-5/inx-19 (AWC$^{OFF/OFF}$)	3	2	1	4	0
nsy-5/inx-19 (AWC$^{OFF/OFF}$)	3	2	1	5	1
nsy-5/inx-19 (AWC$^{OFF/OFF}$)	3	2	1	6	1

nsy-5/inx-19 (AWC$^{OFF/OFF}$)	3	2	1	7	1
nsy-5/inx-19 (AWC$^{OFF/OFF}$)	3	2	1	8	1
nsy-5/inx-19 (AWC$^{OFF/OFF}$)	3	2	1	9	0
nsy-5/inx-19 (AWC$^{OFF/OFF}$)	3	2	1	10	1
nsy-5/inx-19 (AWC$^{OFF/OFF}$)	3	2	1	11	1
nsy-5/inx-19 (AWC$^{OFF/OFF}$)	3	2	1	12	0
nsy-5/inx-19 (AWC$^{OFF/OFF}$)	3	2	1	13	0
nsy-5/inx-19 (AWC$^{OFF/OFF}$)	3	2	1	14	0
nsy-5/inx-19 (AWC$^{OFF/OFF}$)	3	2	1	15	1
nsy-5/inx-19 (AWC$^{OFF/OFF}$)	3	2	2	1	0
nsy-5/inx-19 (AWC$^{OFF/OFF}$)	3	2	2	2	1
nsy-5/inx-19 (AWC$^{OFF/OFF}$)	3	2	2	3	1
nsy-5/inx-19 (AWC$^{OFF/OFF}$)	3	2	2	4	1
nsy-5/inx-19 (AWC$^{OFF/OFF}$)	3	2	2	5	1
nsy-5/inx-19 (AWC$^{OFF/OFF}$)	3	2	2	6	1
nsy-5/inx-19 (AWC$^{OFF/OFF}$)	3	2	2	7	1
nsy-5/inx-19 (AWC$^{OFF/OFF}$)	3	2	2	8	1
nsy-5/inx-19 (AWC$^{OFF/OFF}$)	3	2	2	9	1
nsy-5/inx-19 (AWC$^{OFF/OFF}$)	3	2	2	10	1
nsy-5/inx-19 (AWC$^{OFF/OFF}$)	3	2	2	11	1
nsy-5/inx-19 (AWC$^{OFF/OFF}$)	3	2	2	12	1
nsy-5/inx-19 (AWC$^{OFF/OFF}$)	3	2	2	13	1
nsy-5/inx-19 (AWC$^{OFF/OFF}$)	3	2	2	14	1
nsy-5/inx-19 (AWC$^{OFF/OFF}$)	3	2	2	15	1
nsy-5/inx-19 (AWC$^{OFF/OFF}$)	3	2	2	16	1
nsy-5/inx-19 (AWC$^{OFF/OFF}$)	3	2	2	17	1
nsy-5/inx-19 (AWC$^{OFF/OFF}$)	3	2	2	18	1
nsy-5/inx-19 (AWC$^{OFF/OFF}$)	3	2	2	19	0
nsy-5/inx-19 (AWC$^{OFF/OFF}$)	3	2	3	1	1
nsy-5/inx-19 (AWC$^{OFF/OFF}$)	3	2	3	2	1
nsy-5/inx-19 (AWC$^{OFF/OFF}$)	3	2	3	3	0
nsy-5/inx-19 (AWC$^{OFF/OFF}$)	3	2	3	4	1
nsy-5/inx-19 (AWC$^{OFF/OFF}$)	3	2	3	5	0
nsy-5/inx-19 (AWC$^{OFF/OFF}$)	3	2	3	6	1
nsy-5/inx-19 (AWC$^{OFF/OFF}$)	3	2	3	7	1
nsy-5/inx-19 (AWC$^{OFF/OFF}$)	3	2	3	8	1
nsy-5/inx-19 (AWC$^{OFF/OFF}$)	3	2	3	9	0
nsy-5/inx-19 (AWC$^{OFF/OFF}$)	3	2	3	10	0
nsy-5/inx-19 (AWC$^{OFF/OFF}$)	3	2	3	11	1
nsy-5/inx-19 (AWC$^{OFF/OFF}$)	3	2	3	12	1
nsy-5/inx-19 (AWC$^{OFF/OFF}$)	3	2	3	13	1
nsy-5/inx-19 (AWC$^{OFF/OFF}$)	3	2	3	14	1
nsy-5/inx-19 (AWC$^{OFF/OFF}$)	3	2	3	15	1

nsy-5/inx-19 (AWC$^{OFF/OFF}$)	3	2	3	16	1
nsy-5/inx-19 (AWC$^{OFF/OFF}$)	3	2	4	1	1
nsy-5/inx-19 (AWC$^{OFF/OFF}$)	3	2	4	2	0
nsy-5/inx-19 (AWC$^{OFF/OFF}$)	3	2	4	3	1
nsy-5/inx-19 (AWC$^{OFF/OFF}$)	3	2	4	4	1
nsy-5/inx-19 (AWC$^{OFF/OFF}$)	3	2	4	5	1
nsy-5/inx-19 (AWC$^{OFF/OFF}$)	3	2	4	6	0
nsy-5/inx-19 (AWC$^{OFF/OFF}$)	3	2	4	7	1
nsy-5/inx-19 (AWC$^{OFF/OFF}$)	3	2	4	8	1
nsy-5/inx-19 (AWC$^{OFF/OFF}$)	3	2	4	9	1
nsy-5/inx-19 (AWC$^{OFF/OFF}$)	3	2	4	10	1
nsy-5/inx-19 (AWC$^{OFF/OFF}$)	3	2	4	11	0
nsy-5/inx-19 (AWC$^{OFF/OFF}$)	3	2	4	12	0
nsy-5/inx-19 (AWC$^{OFF/OFF}$)	3	3	1	1	1
nsy-5/inx-19 (AWC$^{OFF/OFF}$)	3	3	1	2	0
nsy-5/inx-19 (AWC$^{OFF/OFF}$)	3	3	1	3	0
nsy-5/inx-19 (AWC$^{OFF/OFF}$)	3	3	1	4	1
nsy-5/inx-19 (AWC$^{OFF/OFF}$)	3	3	1	5	1
nsy-5/inx-19 (AWC$^{OFF/OFF}$)	3	3	1	6	0
nsy-5/inx-19 (AWC$^{OFF/OFF}$)	3	3	1	7	1
nsy-5/inx-19 (AWC$^{OFF/OFF}$)	3	3	1	8	1
nsy-5/inx-19 (AWC$^{OFF/OFF}$)	3	3	1	9	0
nsy-5/inx-19 (AWC$^{OFF/OFF}$)	3	3	1	10	0
nsy-5/inx-19 (AWC$^{OFF/OFF}$)	3	3	1	11	1
nsy-5/inx-19 (AWC$^{OFF/OFF}$)	3	3	1	12	1
nsy-5/inx-19 (AWC$^{OFF/OFF}$)	3	3	2	1	1
nsy-5/inx-19 (AWC$^{OFF/OFF}$)	3	3	2	2	0
nsy-5/inx-19 (AWC$^{OFF/OFF}$)	3	3	2	3	0
nsy-5/inx-19 (AWC$^{OFF/OFF}$)	3	3	2	4	1
nsy-5/inx-19 (AWC$^{OFF/OFF}$)	3	3	2	5	1
nsy-5/inx-19 (AWC$^{OFF/OFF}$)	3	3	2	6	1
nsy-5/inx-19 (AWC$^{OFF/OFF}$)	3	3	2	7	1
nsy-5/inx-19 (AWC$^{OFF/OFF}$)	3	3	2	8	0
nsy-5/inx-19 (AWC$^{OFF/OFF}$)	3	3	2	9	0
nsy-5/inx-19 (AWC$^{OFF/OFF}$)	3	3	2	10	1
nsy-5/inx-19 (AWC$^{OFF/OFF}$)	3	3	2	11	1
nsy-5/inx-19 (AWC$^{OFF/OFF}$)	3	3	2	12	0
nsy-5/inx-19 (AWC$^{OFF/OFF}$)	3	3	2	13	1
nsy-5/inx-19 (AWC$^{OFF/OFF}$)	3	3	2	14	0
nsy-5/inx-19 (AWC$^{OFF/OFF}$)	3	3	2	15	1
nsy-5/inx-19 (AWC$^{OFF/OFF}$)	3	3	2	16	1
nsy-5/inx-19 (AWC$^{OFF/OFF}$)	3	3	2	17	0
nsy-5/inx-19 (AWC$^{OFF/OFF}$)	3	3	2	18	0

nsy-5/inx-19 (AWC$^{OFF/OFF}$)	3	3	2	19	0
nsy-5/inx-19 (AWC$^{OFF/OFF}$)	3	3	2	20	0
nsy-5/inx-19 (AWC$^{OFF/OFF}$)	3	3	2	21	0
nsy-5/inx-19 (AWC$^{OFF/OFF}$)	3	3	3	1	1
nsy-5/inx-19 (AWC$^{OFF/OFF}$)	3	3	3	2	1
nsy-5/inx-19 (AWC$^{OFF/OFF}$)	3	3	3	3	0
nsy-5/inx-19 (AWC$^{OFF/OFF}$)	3	3	3	4	1
nsy-5/inx-19 (AWC$^{OFF/OFF}$)	3	3	3	5	1
nsy-5/inx-19 (AWC$^{OFF/OFF}$)	3	3	3	6	1
nsy-5/inx-19 (AWC$^{OFF/OFF}$)	3	3	3	7	1
nsy-5/inx-19 (AWC$^{OFF/OFF}$)	3	3	3	8	1
nsy-5/inx-19 (AWC$^{OFF/OFF}$)	3	3	3	9	0
nsy-5/inx-19 (AWC$^{OFF/OFF}$)	3	3	3	10	1
nsy-5/inx-19 (AWC$^{OFF/OFF}$)	3	3	3	11	1
nsy-5/inx-19 (AWC$^{OFF/OFF}$)	3	3	3	12	0
nsy-5/inx-19 (AWC$^{OFF/OFF}$)	3	3	3	13	1
nsy-5/inx-19 (AWC$^{OFF/OFF}$)	3	3	3	14	1
nsy-5/inx-19 (AWC$^{OFF/OFF}$)	3	3	3	15	1
nsy-5/inx-19 (AWC$^{OFF/OFF}$)	3	3	3	16	1
nsy-5/inx-19 (AWC$^{OFF/OFF}$)	3	3	3	17	0
nsy-5/inx-19 (AWC$^{OFF/OFF}$)	3	3	4	1	1
nsy-5/inx-19 (AWC$^{OFF/OFF}$)	3	3	4	2	1
nsy-5/inx-19 (AWC$^{OFF/OFF}$)	3	3	4	3	1
nsy-5/inx-19 (AWC$^{OFF/OFF}$)	3	3	4	4	0
nsy-5/inx-19 (AWC$^{OFF/OFF}$)	3	3	4	5	1
nsy-5/inx-19 (AWC$^{OFF/OFF}$)	3	3	4	6	1
nsy-5/inx-19 (AWC$^{OFF/OFF}$)	3	3	4	7	1
nsy-5/inx-19 (AWC$^{OFF/OFF}$)	3	3	4	8	0
nsy-5/inx-19 (AWC$^{OFF/OFF}$)	3	3	4	9	1
nsy-5/inx-19 (AWC$^{OFF/OFF}$)	3	3	4	10	0
nsy-5/inx-19 (AWC$^{OFF/OFF}$)	3	3	4	11	0
nsy-5/inx-19 (AWC$^{OFF/OFF}$)	3	3	4	12	0
nsy-5/inx-19 (AWC$^{OFF/OFF}$)	3	3	4	13	0
nsy-5/inx-19 (AWC$^{OFF/OFF}$)	3	3	4	14	1
nsy-5/inx-19 (AWC$^{OFF/OFF}$)	3	3	4	15	1
nsy-5/inx-19 (AWC$^{OFF/OFF}$)	3	3	4	16	1
nsy-5/inx-19 (AWC$^{OFF/OFF}$)	3	3	4	17	0
nsy-5/inx-19 (AWC$^{OFF/OFF}$)	3	3	4	18	1
nsy-5/inx-19 (AWC$^{OFF/OFF}$)	3	3	4	19	0
nsy-5/inx-19 (AWC$^{OFF/OFF}$)	3	3	4	20	1
AWC::caspase	0	1	1	1	1
AWC::caspase	0	1	1	2	1

AWC::caspase	0	1	1	3	0
AWC::caspase	0	1	1	4	1
AWC::caspase	0	1	1	5	0
AWC::caspase	0	1	1	6	1
AWC::caspase	0	1	1	7	1
AWC::caspase	0	1	1	8	1
AWC::caspase	0	1	1	9	1
AWC::caspase	0	1	1	10	0
AWC::caspase	0	1	1	11	1
AWC::caspase	0	1	1	12	1
AWC::caspase	0	1	2	1	0
AWC::caspase	0	1	2	2	1
AWC::caspase	0	1	2	3	1
AWC::caspase	0	1	2	4	0
AWC::caspase	0	1	2	5	1
AWC::caspase	0	1	2	6	1
AWC::caspase	0	1	2	7	0
AWC::caspase	0	1	2	8	0
AWC::caspase	0	1	2	9	0
AWC::caspase	0	1	2	10	1
AWC::caspase	0	1	2	11	0
AWC::caspase	0	1	2	12	1
AWC::caspase	0	1	2	13	0
AWC::caspase	0	1	3	1	1
AWC::caspase	0	1	3	2	0
AWC::caspase	0	1	3	3	1
AWC::caspase	0	1	3	4	0
AWC::caspase	0	1	3	5	0
AWC::caspase	0	1	3	6	0
AWC::caspase	0	1	3	7	1
AWC::caspase	0	1	3	8	0
AWC::caspase	0	1	3	9	1
AWC::caspase	0	1	3	10	0
AWC::caspase	0	1	3	11	1
AWC::caspase	0	1	3	12	0
AWC::caspase	0	1	3	13	0
AWC::caspase	0	1	3	14	0
AWC::caspase	0	1	3	15	1
AWC::caspase	0	1	3	16	1
AWC::caspase	0	1	3	17	0
AWC::caspase	0	1	3	18	1
AWC::caspase	0	1	3	19	0
AWC::caspase	0	1	4	1	1
AWC::caspase	0	1	4	2	1
AWC::caspase	0	1	4	3	1

AWC::caspase	0	1	4	4	1
AWC::caspase	0	1	4	5	0
AWC::caspase	0	1	4	6	0
AWC::caspase	0	1	4	7	0
AWC::caspase	0	1	4	8	0
AWC::caspase	0	1	4	9	0
AWC::caspase	0	1	4	10	1
AWC::caspase	0	1	4	11	0
AWC::caspase	0	1	4	12	0
AWC::caspase	0	1	4	13	1
AWC::caspase	0	2	1	1	1
AWC::caspase	0	2	1	2	1
AWC::caspase	0	2	1	3	1
AWC::caspase	0	2	1	4	1
AWC::caspase	0	2	1	5	1
AWC::caspase	0	2	1	6	1
AWC::caspase	0	2	1	7	1
AWC::caspase	0	2	1	8	1
AWC::caspase	0	2	1	9	1
AWC::caspase	0	2	1	10	1
AWC::caspase	0	2	1	11	1
AWC::caspase	0	2	1	12	1
AWC::caspase	0	2	2	1	1
AWC::caspase	0	2	2	2	0
AWC::caspase	0	2	2	3	1
AWC::caspase	0	2	2	4	1
AWC::caspase	0	2	2	5	1
AWC::caspase	0	2	2	6	1
AWC::caspase	0	2	2	7	1
AWC::caspase	0	2	2	8	1
AWC::caspase	0	2	2	9	1
AWC::caspase	0	2	2	10	1
AWC::caspase	0	2	2	11	0
AWC::caspase	0	2	2	12	1
AWC::caspase	0	2	2	13	1
AWC::caspase	0	2	2	14	1
AWC::caspase	0	2	3	1	1
AWC::caspase	0	2	3	2	1
AWC::caspase	0	2	3	3	1
AWC::caspase	0	2	3	4	1
AWC::caspase	0	2	3	5	0
AWC::caspase	0	2	3	6	1
AWC::caspase	0	2	3	7	1
AWC::caspase	0	2	3	8	1
AWC::caspase	0	2	3	9	1

AWC::caspase	0	2	3	10	1
AWC::caspase	0	2	3	11	1
AWC::caspase	0	2	3	12	0
AWC::caspase	0	2	3	13	0
AWC::caspase	0	2	3	14	0
AWC::caspase	0	2	3	15	1
AWC::caspase	0	2	3	16	0
AWC::caspase	0	2	3	17	0
AWC::caspase	0	2	4	1	1
AWC::caspase	0	2	4	2	1
AWC::caspase	0	2	4	3	1
AWC::caspase	0	2	4	4	0
AWC::caspase	0	2	4	5	1
AWC::caspase	0	2	4	6	1
AWC::caspase	0	2	4	7	1
AWC::caspase	0	2	4	8	0
AWC::caspase	0	2	4	9	1
AWC::caspase	0	2	4	10	1
AWC::caspase	0	2	4	11	1
AWC::caspase	0	2	4	12	1
AWC::caspase	0	2	4	13	0
AWC::caspase	0	2	4	14	1
AWC::caspase	0	2	4	15	0
AWC::caspase	0	2	4	16	0
AWC::caspase	0	3	1	1	1
AWC::caspase	0	3	1	2	1
AWC::caspase	0	3	1	3	1
AWC::caspase	0	3	1	4	1
AWC::caspase	0	3	1	5	0
AWC::caspase	0	3	1	6	0
AWC::caspase	0	3	1	7	1
AWC::caspase	0	3	1	8	1
AWC::caspase	0	3	2	1	1
AWC::caspase	0	3	2	2	1
AWC::caspase	0	3	2	3	1
AWC::caspase	0	3	2	4	1
AWC::caspase	0	3	2	5	1
AWC::caspase	0	3	2	6	1
AWC::caspase	0	3	2	7	1
AWC::caspase	0	3	2	8	1
AWC::caspase	0	3	2	9	1
AWC::caspase	0	3	2	10	1
AWC::caspase	0	3	2	11	1
AWC::caspase	0	3	2	12	1
AWC::caspase	0	3	2	13	1

AWC::caspase	0	3	2	14	1
AWC::caspase	0	3	2	15	1
AWC::caspase	0	3	2	16	1
AWC::caspase	0	3	2	17	1
AWC::caspase	0	3	2	18	0
AWC::caspase	0	3	3	1	1
AWC::caspase	0	3	3	2	0
AWC::caspase	0	3	3	3	1
AWC::caspase	0	3	3	4	0
AWC::caspase	0	3	3	5	1
AWC::caspase	0	3	3	6	1
AWC::caspase	0	3	3	7	1
AWC::caspase	0	3	3	8	1
AWC::caspase	0	3	3	9	1
AWC::caspase	0	3	3	10	1
AWC::caspase	0	3	3	11	0
AWC::caspase	0	3	3	12	1
AWC::caspase	0	3	3	13	1
AWC::caspase	0	3	3	14	0
AWC::caspase	0	3	3	15	1
AWC::caspase	0	3	3	16	0
AWC::caspase	0	3	3	17	1
AWC::caspase	0	3	3	18	1
AWC::caspase	0	3	3	19	0
AWC::caspase	0	3	3	20	1
AWC::caspase	0	3	3	21	1
AWC::caspase	0	3	3	22	0
AWC::caspase	0	3	3	23	1
AWC::caspase	0	3	3	24	0
AWC::caspase	0	3	3	25	1
AWC::caspase	0	3	3	26	1
AWC::caspase	0	3	3	27	1
AWC::caspase	0	3	4	1	0
AWC::caspase	0	3	4	2	0
AWC::caspase	0	3	4	3	0
AWC::caspase	0	3	4	4	0
AWC::caspase	0	3	4	5	1
AWC::caspase	0	3	4	6	0
AWC::caspase	0	3	4	7	1
AWC::caspase	0	3	4	8	1
AWC::caspase	0	3	4	9	0
AWC::caspase	0	3	4	10	0
AWC::caspase	0	3	4	11	1
AWC::caspase	0	3	4	12	1
AWC::caspase	0	3	4	13	1

AWC::caspase	0	3	4	14	1
AWC::caspase	0	3	4	15	1
AWC::caspase	0	3	4	16	1
AWC::caspase	0	3	4	17	1
AWC::caspase	0	3	4	18	1
AWC::caspase	0	3	4	19	1
AWC::caspase	0	3	4	20	0
AWC::caspase	0	3	4	21	1
AWC::caspase	0	3	4	22	0
AWC::caspase	0	3	4	23	0
AWC::caspase	0	3	4	24	1
AWC::caspase	0	3	4	25	1
AWC::caspase	0	3	4	26	0
AWC::caspase	0	3	4	27	1
AWC::caspase	0	3	4	28	0
AWC::caspase	0	3	4	29	1
AWC::caspase	0	3	4	30	1
AWC::caspase	0	3	4	31	1
AWC::caspase	3	1	1	1	0
AWC::caspase	3	1	1	2	0
AWC::caspase	3	1	1	3	0
AWC::caspase	3	1	1	4	1
AWC::caspase	3	1	1	5	0
AWC::caspase	3	1	1	6	0
AWC::caspase	3	1	1	7	0
AWC::caspase	3	1	1	8	0
AWC::caspase	3	1	1	9	0
AWC::caspase	3	1	1	10	0
AWC::caspase	3	1	2	1	0
AWC::caspase	3	1	2	2	0
AWC::caspase	3	1	2	3	0
AWC::caspase	3	1	2	4	0
AWC::caspase	3	1	2	5	0
AWC::caspase	3	1	2	6	0
AWC::caspase	3	1	2	7	0
AWC::caspase	3	1	2	8	0
AWC::caspase	3	1	2	9	0
AWC::caspase	3	1	2	10	0
AWC::caspase	3	1	2	11	0
AWC::caspase	3	1	2	12	0
AWC::caspase	3	1	2	13	1
AWC::caspase	3	1	2	14	1
AWC::caspase	3	1	2	15	1
AWC::caspase	3	1	2	16	1
AWC::caspase	3	1	2	17	1

AWC::caspase	3	1	2	18	0
AWC::caspase	3	1	2	19	0
AWC::caspase	3	1	2	20	0
AWC::caspase	3	1	2	21	0
AWC::caspase	3	1	2	22	0
AWC::caspase	3	1	3	1	0
AWC::caspase	3	1	3	2	0
AWC::caspase	3	1	3	3	0
AWC::caspase	3	1	3	4	0
AWC::caspase	3	1	3	5	0
AWC::caspase	3	1	3	6	0
AWC::caspase	3	1	3	7	0
AWC::caspase	3	1	3	8	0
AWC::caspase	3	1	3	9	0
AWC::caspase	3	1	3	10	0
AWC::caspase	3	1	3	11	0
AWC::caspase	3	1	3	12	0
AWC::caspase	3	1	3	13	0
AWC::caspase	3	1	3	14	0
AWC::caspase	3	1	3	15	0
AWC::caspase	3	1	4	1	0
AWC::caspase	3	1	4	2	0
AWC::caspase	3	1	4	3	0
AWC::caspase	3	1	4	4	0
AWC::caspase	3	1	4	5	1
AWC::caspase	3	1	4	6	0
AWC::caspase	3	1	4	7	0
AWC::caspase	3	2	1	1	1
AWC::caspase	3	2	1	2	1
AWC::caspase	3	2	1	3	1
AWC::caspase	3	2	1	4	0
AWC::caspase	3	2	1	5	0
AWC::caspase	3	2	1	6	0
AWC::caspase	3	2	1	7	1
AWC::caspase	3	2	1	8	0
AWC::caspase	3	2	1	9	0
AWC::caspase	3	2	1	10	0
AWC::caspase	3	2	1	11	0
AWC::caspase	3	2	1	12	0
AWC::caspase	3	2	2	1	0
AWC::caspase	3	2	2	2	0
AWC::caspase	3	2	2	3	0
AWC::caspase	3	2	2	4	0
AWC::caspase	3	2	2	5	0
AWC::caspase	3	2	2	6	0

AWC::caspase	3	2	2	7	0
AWC::caspase	3	2	2	8	0
AWC::caspase	3	2	2	9	0
AWC::caspase	3	2	2	10	0
AWC::caspase	3	2	2	11	1
AWC::caspase	3	2	2	12	0
AWC::caspase	3	2	2	13	1
AWC::caspase	3	2	2	14	0
AWC::caspase	3	2	2	15	1
AWC::caspase	3	2	2	16	0
AWC::caspase	3	2	2	17	0
AWC::caspase	3	2	2	18	0
AWC::caspase	3	2	2	19	0
AWC::caspase	3	2	2	20	1
AWC::caspase	3	2	3	1	0
AWC::caspase	3	2	3	2	0
AWC::caspase	3	2	3	3	0
AWC::caspase	3	2	3	4	0
AWC::caspase	3	2	3	5	0
AWC::caspase	3	2	3	6	0
AWC::caspase	3	2	3	7	0
AWC::caspase	3	2	3	8	0
AWC::caspase	3	2	3	9	0
AWC::caspase	3	2	3	10	0
AWC::caspase	3	2	3	11	0
AWC::caspase	3	2	3	12	1
AWC::caspase	3	2	3	13	0
AWC::caspase	3	2	3	14	0
AWC::caspase	3	2	3	15	0
AWC::caspase	3	2	3	16	1
AWC::caspase	3	2	3	17	1
AWC::caspase	3	2	3	18	1
AWC::caspase	3	2	3	19	0
AWC::caspase	3	2	3	20	1
AWC::caspase	3	2	4	1	0
AWC::caspase	3	2	4	2	0
AWC::caspase	3	2	4	3	0
AWC::caspase	3	2	4	4	0
AWC::caspase	3	2	4	5	0
AWC::caspase	3	2	4	6	0
AWC::caspase	3	2	4	7	0
AWC::caspase	3	2	4	8	1
AWC::caspase	3	2	4	9	1
AWC::caspase	3	2	4	10	0
AWC::caspase	3	2	4	11	0

AWC::caspase	3	2	4	12	0
AWC::caspase	3	2	4	13	0
AWC::caspase	3	2	4	14	0
AWC::caspase	3	2	4	15	0
AWC::caspase	3	3	1	1	0
AWC::caspase	3	3	1	2	0
AWC::caspase	3	3	1	3	0
AWC::caspase	3	3	1	4	0
AWC::caspase	3	3	1	5	0
AWC::caspase	3	3	1	6	0
AWC::caspase	3	3	1	7	0
AWC::caspase	3	3	1	8	0
AWC::caspase	3	3	1	9	0
AWC::caspase	3	3	1	10	0
AWC::caspase	3	3	1	11	0
AWC::caspase	3	3	1	12	0
AWC::caspase	3	3	1	13	0
AWC::caspase	3	3	1	14	0
AWC::caspase	3	3	1	15	0
AWC::caspase	3	3	1	16	0
AWC::caspase	3	3	1	17	0
AWC::caspase	3	3	2	1	0
AWC::caspase	3	3	2	2	0
AWC::caspase	3	3	2	3	0
AWC::caspase	3	3	2	4	0
AWC::caspase	3	3	2	5	0
AWC::caspase	3	3	2	6	0
AWC::caspase	3	3	2	7	0
AWC::caspase	3	3	2	8	0
AWC::caspase	3	3	2	9	0
AWC::caspase	3	3	2	10	0
AWC::caspase	3	3	2	11	0
AWC::caspase	3	3	2	12	1
AWC::caspase	3	3	2	13	0
AWC::caspase	3	3	3	1	1
AWC::caspase	3	3	3	2	0
AWC::caspase	3	3	3	3	1
AWC::caspase	3	3	3	4	0
AWC::caspase	3	3	3	5	0
AWC::caspase	3	3	3	6	0
AWC::caspase	3	3	3	7	0
AWC::caspase	3	3	3	8	0
AWC::caspase	3	3	3	9	1
AWC::caspase	3	3	3	10	0
AWC::caspase	3	3	3	11	0

AWC::caspase	3	3	3	12	0
AWC::caspase	3	3	3	13	0
AWC::caspase	3	3	3	14	0
AWC::caspase	3	3	3	15	0
AWC::caspase	3	3	3	16	1
AWC::caspase	3	3	3	17	0
AWC::caspase	3	3	4	1	0
AWC::caspase	3	3	4	2	0
AWC::caspase	3	3	4	3	0
AWC::caspase	3	3	4	4	0
AWC::caspase	3	3	4	5	0
AWC::caspase	3	3	4	6	0
AWC::caspase	3	3	4	7	0
AWC::caspase	3	3	4	8	0
AWC::caspase	3	3	4	9	1
AWC::caspase	3	3	4	10	0
AWC::caspase	3	3	4	11	0
AWC::caspase	3	3	4	12	0
AWC::caspase	3	3	4	13	0
ASH::caspase	0	1	1	1	1
ASH::caspase	0	1	1	2	1
ASH::caspase	0	1	1	3	1
ASH::caspase	0	1	1	4	1
ASH::caspase	0	1	1	5	1
ASH::caspase	0	1	1	6	1
ASH::caspase	0	1	1	7	1
ASH::caspase	0	1	1	8	1
ASH::caspase	0	1	1	9	1
ASH::caspase	0	1	1	10	1
ASH::caspase	0	1	1	11	1
ASH::caspase	0	1	1	12	1
ASH::caspase	0	1	1	13	0
ASH::caspase	0	1	1	14	0
ASH::caspase	0	1	1	15	1
ASH::caspase	0	1	1	16	1
ASH::caspase	0	1	1	17	0
ASH::caspase	0	1	1	18	0
ASH::caspase	0	1	1	19	1
ASH::caspase	0	1	2	1	0
ASH::caspase	0	1	2	2	0
ASH::caspase	0	1	2	3	1
ASH::caspase	0	1	2	4	1
ASH::caspase	0	1	2	5	0
ASH::caspase	0	1	2	6	0

ASH::caspase	0	1	2	7	0
ASH::caspase	0	1	2	8	1
ASH::caspase	0	1	2	9	0
ASH::caspase	0	1	2	10	0
ASH::caspase	0	1	2	11	1
ASH::caspase	0	1	2	12	1
ASH::caspase	0	1	2	13	1
ASH::caspase	0	1	2	14	1
ASH::caspase	0	1	2	15	1
ASH::caspase	0	1	3	1	0
ASH::caspase	0	1	3	2	0
ASH::caspase	0	1	3	3	1
ASH::caspase	0	1	3	4	1
ASH::caspase	0	1	3	5	1
ASH::caspase	0	1	3	6	0
ASH::caspase	0	1	3	7	0
ASH::caspase	0	1	3	8	0
ASH::caspase	0	1	3	9	1
ASH::caspase	0	1	3	10	0
ASH::caspase	0	1	3	11	1
ASH::caspase	0	1	3	12	1
ASH::caspase	0	1	3	13	0
ASH::caspase	0	1	4	1	1
ASH::caspase	0	1	4	2	0
ASH::caspase	0	1	4	3	0
ASH::caspase	0	1	4	4	0
ASH::caspase	0	1	4	5	1
ASH::caspase	0	1	4	6	0
ASH::caspase	0	1	4	7	1
ASH::caspase	0	1	4	8	0
ASH::caspase	0	1	4	9	0
ASH::caspase	0	1	4	10	1
ASH::caspase	0	1	4	11	1
ASH::caspase	0	1	4	12	0
ASH::caspase	0	1	4	13	1
ASH::caspase	0	2	1	1	1
ASH::caspase	0	2	1	2	1
ASH::caspase	0	2	1	3	1
ASH::caspase	0	2	1	4	1
ASH::caspase	0	2	1	5	0
ASH::caspase	0	2	1	6	1
ASH::caspase	0	2	1	7	1
ASH::caspase	0	2	1	8	1
ASH::caspase	0	2	1	9	1
ASH::caspase	0	2	1	10	0

ASH::caspase	0	2	1	11	1
ASH::caspase	0	2	1	12	1
ASH::caspase	0	2	1	13	1
ASH::caspase	0	2	2	1	1
ASH::caspase	0	2	2	2	1
ASH::caspase	0	2	2	3	1
ASH::caspase	0	2	2	4	1
ASH::caspase	0	2	2	5	1
ASH::caspase	0	2	2	6	1
ASH::caspase	0	2	2	7	1
ASH::caspase	0	2	2	8	0
ASH::caspase	0	2	2	9	1
ASH::caspase	0	2	2	10	1
ASH::caspase	0	2	2	11	0
ASH::caspase	0	2	3	1	1
ASH::caspase	0	2	3	2	0
ASH::caspase	0	2	3	3	0
ASH::caspase	0	2	3	4	1
ASH::caspase	0	2	4	1	0
ASH::caspase	0	2	4	2	1
ASH::caspase	0	2	4	3	1
ASH::caspase	0	2	4	4	1
ASH::caspase	0	2	4	5	1
ASH::caspase	0	3	1	1	1
ASH::caspase	0	3	1	2	1
ASH::caspase	0	3	1	3	1
ASH::caspase	0	3	1	4	1
ASH::caspase	0	3	1	5	1
ASH::caspase	0	3	1	6	1
ASH::caspase	0	3	1	7	1
ASH::caspase	0	3	1	8	1
ASH::caspase	0	3	1	9	1
ASH::caspase	0	3	1	10	1
ASH::caspase	0	3	1	11	1
ASH::caspase	0	3	1	12	1
ASH::caspase	0	3	1	13	1
ASH::caspase	0	3	1	14	1
ASH::caspase	0	3	1	15	1
ASH::caspase	0	3	1	16	1
ASH::caspase	0	3	1	17	1
ASH::caspase	0	3	1	18	1
ASH::caspase	0	3	1	19	0
ASH::caspase	0	3	1	20	1
ASH::caspase	0	3	1	21	1
ASH::caspase	0	3	2	1	1

ASH::caspase	0	3	2	2	1
ASH::caspase	0	3	2	3	1
ASH::caspase	0	3	2	4	1
ASH::caspase	0	3	2	5	1
ASH::caspase	0	3	2	6	0
ASH::caspase	0	3	2	7	0
ASH::caspase	0	3	2	8	1
ASH::caspase	0	3	2	9	1
ASH::caspase	0	3	2	10	1
ASH::caspase	0	3	2	11	1
ASH::caspase	0	3	2	12	1
ASH::caspase	0	3	2	13	1
ASH::caspase	0	3	2	14	1
ASH::caspase	0	3	2	15	1
ASH::caspase	0	3	2	16	0
ASH::caspase	0	3	2	17	1
ASH::caspase	0	3	2	18	1
ASH::caspase	0	3	2	19	1
ASH::caspase	0	3	2	20	1
ASH::caspase	0	3	2	21	0
ASH::caspase	0	3	2	22	0
ASH::caspase	0	3	2	23	1
ASH::caspase	0	3	2	24	1
ASH::caspase	0	3	2	25	1
ASH::caspase	0	3	3	1	0
ASH::caspase	0	3	3	2	1
ASH::caspase	0	3	3	3	1
ASH::caspase	0	3	3	4	0
ASH::caspase	0	3	3	5	1
ASH::caspase	0	3	3	6	1
ASH::caspase	0	3	3	7	1
ASH::caspase	0	3	3	8	1
ASH::caspase	0	3	3	9	1
ASH::caspase	0	3	3	10	0
ASH::caspase	0	3	3	11	1
ASH::caspase	0	3	3	12	0
ASH::caspase	0	3	3	13	1
ASH::caspase	0	3	3	14	1
ASH::caspase	0	3	3	15	1
ASH::caspase	0	3	3	16	0
ASH::caspase	0	3	3	17	0
ASH::caspase	0	3	3	18	1
ASH::caspase	0	3	3	19	0
ASH::caspase	0	3	3	20	1
ASH::caspase	0	3	4	1	0

ASH::caspase	0	3	4	2	1
ASH::caspase	0	3	4	3	1
ASH::caspase	0	3	4	4	0
ASH::caspase	0	3	4	5	1
ASH::caspase	0	3	4	6	1
ASH::caspase	0	3	4	7	1
ASH::caspase	0	3	4	8	1
ASH::caspase	0	3	4	9	1
ASH::caspase	0	3	4	10	0
ASH::caspase	0	3	4	11	1
ASH::caspase	0	3	4	12	0
ASH::caspase	0	3	4	13	1
ASH::caspase	0	3	4	14	1
ASH::caspase	0	3	4	15	1
ASH::caspase	0	3	4	16	1
ASH::caspase	0	3	4	17	1
ASH::caspase	0	3	4	18	1
ASH::caspase	0	3	4	19	0
ASH::caspase	3	1	1	1	0
ASH::caspase	3	1	1	2	0
ASH::caspase	3	1	1	3	0
ASH::caspase	3	1	1	4	0
ASH::caspase	3	1	1	5	0
ASH::caspase	3	1	1	6	0
ASH::caspase	3	1	1	7	0
ASH::caspase	3	1	2	1	0
ASH::caspase	3	1	2	2	0
ASH::caspase	3	1	2	3	0
ASH::caspase	3	1	2	4	0
ASH::caspase	3	1	2	5	0
ASH::caspase	3	1	2	6	0
ASH::caspase	3	1	2	7	0
ASH::caspase	3	1	2	8	0
ASH::caspase	3	1	2	9	0
ASH::caspase	3	1	2	10	0
ASH::caspase	3	1	2	11	0
ASH::caspase	3	1	2	12	0
ASH::caspase	3	1	2	13	0
ASH::caspase	3	1	2	14	1
ASH::caspase	3	1	2	15	0
ASH::caspase	3	1	2	16	0
ASH::caspase	3	1	2	17	0
ASH::caspase	3	1	2	18	0
ASH::caspase	3	1	3	1	0
ASH::caspase	3	1	3	2	0

ASH::caspase	3	1	3	3	0
ASH::caspase	3	1	3	4	0
ASH::caspase	3	1	3	5	0
ASH::caspase	3	1	3	6	0
ASH::caspase	3	1	3	7	0
ASH::caspase	3	1	3	8	0
ASH::caspase	3	1	3	9	0
ASH::caspase	3	1	3	10	1
ASH::caspase	3	1	3	11	0
ASH::caspase	3	1	3	12	1
ASH::caspase	3	1	3	13	0
ASH::caspase	3	1	3	14	0
ASH::caspase	3	1	3	15	0
ASH::caspase	3	1	3	16	0
ASH::caspase	3	1	3	17	0
ASH::caspase	3	1	3	18	0
ASH::caspase	3	1	3	19	0
ASH::caspase	3	1	4	1	0
ASH::caspase	3	1	4	2	0
ASH::caspase	3	1	4	3	0
ASH::caspase	3	1	4	4	0
ASH::caspase	3	1	4	5	0
ASH::caspase	3	1	4	6	0
ASH::caspase	3	1	4	7	0
ASH::caspase	3	1	4	8	0
ASH::caspase	3	1	4	9	0
ASH::caspase	3	1	4	10	0
ASH::caspase	3	1	4	11	1
ASH::caspase	3	1	4	12	0
ASH::caspase	3	1	4	13	1
ASH::caspase	3	1	4	14	0
ASH::caspase	3	1	4	15	0
ASH::caspase	3	1	4	16	0
ASH::caspase	3	2	1	1	0
ASH::caspase	3	2	1	2	0
ASH::caspase	3	2	1	3	0
ASH::caspase	3	2	1	4	0
ASH::caspase	3	2	1	5	1
ASH::caspase	3	2	1	6	0
ASH::caspase	3	2	1	7	0
ASH::caspase	3	2	1	8	0
ASH::caspase	3	2	1	9	0
ASH::caspase	3	2	1	10	0
ASH::caspase	3	2	2	1	0
ASH::caspase	3	2	2	2	0

ASH::caspase	3	2	2	3	0
ASH::caspase	3	2	2	4	0
ASH::caspase	3	2	2	5	0
ASH::caspase	3	2	2	6	1
ASH::caspase	3	2	2	7	0
ASH::caspase	3	2	2	8	0
ASH::caspase	3	2	2	9	0
ASH::caspase	3	2	2	10	0
ASH::caspase	3	2	2	11	0
ASH::caspase	3	2	2	12	0
ASH::caspase	3	2	2	13	0
ASH::caspase	3	2	3	1	0
ASH::caspase	3	2	3	2	0
ASH::caspase	3	2	3	3	0
ASH::caspase	3	2	3	4	0
ASH::caspase	3	2	3	5	0
ASH::caspase	3	2	3	6	0
ASH::caspase	3	2	3	7	0
ASH::caspase	3	2	3	8	0
ASH::caspase	3	2	3	9	0
ASH::caspase	3	2	3	10	0
ASH::caspase	3	2	3	11	0
ASH::caspase	3	2	3	12	0
ASH::caspase	3	2	3	13	0
ASH::caspase	3	2	3	14	1
ASH::caspase	3	2	3	15	0
ASH::caspase	3	2	3	16	0
ASH::caspase	3	2	3	17	0
ASH::caspase	3	2	3	18	0
ASH::caspase	3	2	3	19	0
ASH::caspase	3	2	4	1	0
ASH::caspase	3	2	4	2	0
ASH::caspase	3	2	4	3	0
ASH::caspase	3	2	4	4	0
ASH::caspase	3	2	4	5	0
ASH::caspase	3	2	4	6	0
ASH::caspase	3	2	4	7	0
ASH::caspase	3	2	4	8	0
ASH::caspase	3	2	4	9	0
ASH::caspase	3	2	4	10	0
ASH::caspase	3	2	4	11	0
ASH::caspase	3	3	1	1	0
ASH::caspase	3	3	1	2	0
ASH::caspase	3	3	1	3	0
ASH::caspase	3	3	1	4	0

ASH::caspase	3	3	1	5	0
ASH::caspase	3	3	1	6	1
ASH::caspase	3	3	1	7	0
ASH::caspase	3	3	1	8	0
ASH::caspase	3	3	1	9	0
ASH::caspase	3	3	1	10	0
ASH::caspase	3	3	1	11	0
ASH::caspase	3	3	2	1	0
ASH::caspase	3	3	2	2	0
ASH::caspase	3	3	2	3	0
ASH::caspase	3	3	2	4	0
ASH::caspase	3	3	2	5	0
ASH::caspase	3	3	2	6	0
ASH::caspase	3	3	2	7	0
ASH::caspase	3	3	2	8	0
ASH::caspase	3	3	2	9	1
ASH::caspase	3	3	2	10	1
ASH::caspase	3	3	2	11	0
ASH::caspase	3	3	2	12	0
ASH::caspase	3	3	2	13	0
ASH::caspase	3	3	2	14	0
ASH::caspase	3	3	2	15	0
ASH::caspase	3	3	2	16	0
ASH::caspase	3	3	2	17	1
ASH::caspase	3	3	2	18	0
ASH::caspase	3	3	2	19	0
ASH::caspase	3	3	2	20	0
ASH::caspase	3	3	3	1	0
ASH::caspase	3	3	3	2	0
ASH::caspase	3	3	3	3	0
ASH::caspase	3	3	3	4	1
ASH::caspase	3	3	3	5	0
ASH::caspase	3	3	3	6	0
ASH::caspase	3	3	3	7	0
ASH::caspase	3	3	3	8	0
ASH::caspase	3	3	3	9	0
ASH::caspase	3	3	3	10	0
ASH::caspase	3	3	3	11	0
ASH::caspase	3	3	3	12	0
ASH::caspase	3	3	3	13	0
ASH::caspase	3	3	3	14	0
ASH::caspase	3	3	3	15	0
ASH::caspase	3	3	3	16	0
ASH::caspase	3	3	3	17	0
ASH::caspase	3	3	4	1	0

ASH::caspase	3	3	4	2	0
ASH::caspase	3	3	4	3	0
ASH::caspase	3	3	4	4	0
ASH::caspase	3	3	4	5	0
ASH::caspase	3	3	4	6	0
ASH::caspase	3	3	4	7	0
ASH::caspase	3	3	4	8	0
ASH::caspase	3	3	4	9	0
ASH::caspase	3	3	4	10	0
ASH::caspase	3	3	4	11	0
ASJ::caspase	0	1	1	1	1
ASJ::caspase	0	1	1	2	1
ASJ::caspase	0	1	1	3	1
ASJ::caspase	0	1	2	1	1
ASJ::caspase	0	1	2	2	1
ASJ::caspase	0	1	2	3	1
ASJ::caspase	0	1	2	4	1
ASJ::caspase	0	1	2	5	1
ASJ::caspase	0	1	3	1	1
ASJ::caspase	0	1	3	2	1
ASJ::caspase	0	1	3	3	0
ASJ::caspase	0	1	3	4	1
ASJ::caspase	0	1	3	5	1
ASJ::caspase	0	1	3	6	1
ASJ::caspase	0	2	4	1	1
ASJ::caspase	0	2	4	2	0
ASJ::caspase	0	2	4	3	0
ASJ::caspase	0	2	4	4	1
ASJ::caspase	0	2	4	5	1
ASJ::caspase	0	2	4	6	0
ASJ::caspase	0	2	1	1	1
ASJ::caspase	0	2	1	2	1
ASJ::caspase	0	2	1	3	0
ASJ::caspase	0	2	1	4	0
ASJ::caspase	0	2	1	5	1
ASJ::caspase	0	2	1	6	1
ASJ::caspase	0	2	1	7	1
ASJ::caspase	0	2	1	8	0
ASJ::caspase	0	2	1	9	1
ASJ::caspase	0	2	1	10	0
ASJ::caspase	0	2	1	11	0
ASJ::caspase	0	2	1	12	0
ASJ::caspase	0	2	1	13	0
ASJ::caspase	0	2	1	14	0

ASJ::caspase	0	2	1	15	1
ASJ::caspase	0	2	1	16	1
ASJ::caspase	0	2	2	1	0
ASJ::caspase	0	2	2	2	0
ASJ::caspase	0	2	2	3	1
ASJ::caspase	0	2	2	4	0
ASJ::caspase	0	2	2	5	1
ASJ::caspase	0	2	2	6	1
ASJ::caspase	0	2	2	7	1
ASJ::caspase	0	2	2	8	1
ASJ::caspase	0	2	2	9	1
ASJ::caspase	0	2	2	10	0
ASJ::caspase	0	2	2	11	1
ASJ::caspase	0	2	2	12	1
ASJ::caspase	0	2	2	13	0
ASJ::caspase	0	2	2	14	1
ASJ::caspase	0	2	3	1	0
ASJ::caspase	0	2	3	2	1
ASJ::caspase	0	2	3	3	0
ASJ::caspase	0	2	3	4	1
ASJ::caspase	0	2	3	5	1
ASJ::caspase	0	2	3	6	1
ASJ::caspase	0	2	3	7	0
ASJ::caspase	0	2	3	8	1
ASJ::caspase	0	2	3	9	1
ASJ::caspase	0	2	3	10	0
ASJ::caspase	0	2	3	11	0
ASJ::caspase	0	2	3	12	0
ASJ::caspase	0	2	3	13	1
ASJ::caspase	0	2	4	1	0
ASJ::caspase	0	2	4	2	0
ASJ::caspase	0	2	4	3	0
ASJ::caspase	0	2	4	4	1
ASJ::caspase	0	2	4	5	1
ASJ::caspase	0	2	4	6	1
ASJ::caspase	0	2	4	7	1
ASJ::caspase	0	2	4	8	1
ASJ::caspase	0	2	4	9	1
ASJ::caspase	0	2	4	10	1
ASJ::caspase	0	2	4	11	1
ASJ::caspase	0	2	4	12	0
ASJ::caspase	0	2	4	13	0
ASJ::caspase	0	3	1	1	1
ASJ::caspase	0	3	1	2	1
ASJ::caspase	0	3	1	3	1

ASJ::caspase	0	3	1	4	1
ASJ::caspase	0	3	1	5	0
ASJ::caspase	0	3	1	6	0
ASJ::caspase	0	3	2	1	0
ASJ::caspase	0	3	2	2	1
ASJ::caspase	0	3	2	3	1
ASJ::caspase	0	3	2	4	1
ASJ::caspase	0	3	2	5	1
ASJ::caspase	0	3	2	6	1
ASJ::caspase	0	3	2	7	1
ASJ::caspase	0	3	2	8	1
ASJ::caspase	0	3	2	9	1
ASJ::caspase	0	3	3	1	1
ASJ::caspase	0	3	3	2	1
ASJ::caspase	0	3	3	3	1
ASJ::caspase	0	3	3	4	1
ASJ::caspase	0	3	3	5	0
ASJ::caspase	0	3	3	6	1
ASJ::caspase	0	3	3	7	1
ASJ::caspase	0	3	3	8	1
ASJ::caspase	0	3	3	9	1
ASJ::caspase	0	3	3	10	1
ASJ::caspase	0	3	3	11	0
ASJ::caspase	0	3	3	12	1
ASJ::caspase	0	3	3	13	1
ASJ::caspase	0	3	3	14	1
ASJ::caspase	0	3	4	1	1
ASJ::caspase	0	3	4	2	1
ASJ::caspase	0	3	4	3	0
ASJ::caspase	0	3	4	4	0
ASJ::caspase	0	3	4	5	1
ASJ::caspase	0	3	4	6	0
ASJ::caspase	0	3	4	7	1
ASJ::caspase	0	3	4	8	1
ASJ::caspase	0	3	4	9	1
ASJ::caspase	0	3	4	10	0
ASJ::caspase	3	1	1	1	0
ASJ::caspase	3	1	1	2	0
ASJ::caspase	3	1	1	3	0
ASJ::caspase	3	1	1	4	0
ASJ::caspase	3	1	1	5	0
ASJ::caspase	3	1	1	6	0
ASJ::caspase	3	1	1	7	0
ASJ::caspase	3	1	1	8	1
ASJ::caspase	3	1	1	9	0

ASJ::caspase	3	1	1	10	0
ASJ::caspase	3	1	1	11	0
ASJ::caspase	3	1	1	12	0
ASJ::caspase	3	1	1	13	0
ASJ::caspase	3	1	2	1	0
ASJ::caspase	3	1	2	2	0
ASJ::caspase	3	1	2	3	0
ASJ::caspase	3	1	2	4	0
ASJ::caspase	3	1	2	5	0
ASJ::caspase	3	1	2	6	0
ASJ::caspase	3	1	2	7	0
ASJ::caspase	3	1	2	8	0
ASJ::caspase	3	1	2	9	0
ASJ::caspase	3	1	2	10	0
ASJ::caspase	3	1	2	11	0
ASJ::caspase	3	1	2	12	0
ASJ::caspase	3	1	2	13	0
ASJ::caspase	3	1	2	14	0
ASJ::caspase	3	1	2	15	0
ASJ::caspase	3	1	3	1	0
ASJ::caspase	3	1	3	2	0
ASJ::caspase	3	1	3	3	0
ASJ::caspase	3	1	3	4	0
ASJ::caspase	3	1	3	5	0
ASJ::caspase	3	1	3	6	0
ASJ::caspase	3	1	3	7	0
ASJ::caspase	3	1	3	8	0
ASJ::caspase	3	1	3	9	0
ASJ::caspase	3	1	3	10	0
ASJ::caspase	3	2	1	1	0
ASJ::caspase	3	2	1	2	0
ASJ::caspase	3	2	1	3	0
ASJ::caspase	3	2	1	4	0
ASJ::caspase	3	2	1	5	0
ASJ::caspase	3	2	1	6	0
ASJ::caspase	3	2	1	7	1
ASJ::caspase	3	2	2	1	0
ASJ::caspase	3	2	2	2	0
ASJ::caspase	3	2	2	3	0
ASJ::caspase	3	2	2	4	0
ASJ::caspase	3	2	2	5	0
ASJ::caspase	3	2	2	6	0
ASJ::caspase	3	2	2	7	0
ASJ::caspase	3	2	2	8	0
ASJ::caspase	3	2	2	9	1

ASJ::caspase	3	2	2	10	0
ASJ::caspase	3	2	2	11	0
ASJ::caspase	3	2	3	1	0
ASJ::caspase	3	2	3	2	0
ASJ::caspase	3	2	3	3	0
ASJ::caspase	3	2	3	4	0
ASJ::caspase	3	2	3	5	0
ASJ::caspase	3	2	3	6	0
ASJ::caspase	3	2	3	7	0
ASJ::caspase	3	2	3	8	0
ASJ::caspase	3	2	3	9	0
ASJ::caspase	3	2	3	10	0
ASJ::caspase	3	2	3	11	0
ASJ::caspase	3	2	4	1	0
ASJ::caspase	3	2	4	2	0
ASJ::caspase	3	2	4	3	0
ASJ::caspase	3	2	4	4	0
ASJ::caspase	3	2	4	5	0
ASJ::caspase	3	2	4	6	0
ASJ::caspase	3	3	1	1	0
ASJ::caspase	3	3	1	2	0
ASJ::caspase	3	3	1	3	0
ASJ::caspase	3	3	1	4	0
ASJ::caspase	3	3	1	5	0
ASJ::caspase	3	3	1	6	0
ASJ::caspase	3	3	1	7	0
ASJ::caspase	3	3	1	8	0
ASJ::caspase	3	3	1	9	0
ASJ::caspase	3	3	1	10	0
ASJ::caspase	3	3	1	11	0
ASJ::caspase	3	3	1	12	0
ASJ::caspase	3	3	1	13	0
ASJ::caspase	3	3	1	14	0
ASJ::caspase	3	3	2	1	0
ASJ::caspase	3	3	2	2	0
ASJ::caspase	3	3	2	3	1
ASJ::caspase	3	3	2	4	0
ASJ::caspase	3	3	2	5	0
ASJ::caspase	3	3	2	6	0
ASJ::caspase	3	3	2	7	0
ASJ::caspase	3	3	2	8	0
ASJ::caspase	3	3	2	9	0
ASJ::caspase	3	3	2	10	0
ASJ::caspase	3	3	2	11	0
ASJ::caspase	3	3	2	12	0

ASJ::caspase	3	3	2	13	0
ASJ::caspase	3	3	2	14	1
ASJ::caspase	3	3	2	15	1
ASJ::caspase	3	3	3	1	0
ASJ::caspase	3	3	3	2	0
ASJ::caspase	3	3	3	3	0
ASJ::caspase	3	3	3	4	0
ASJ::caspase	3	3	3	5	0
ASJ::caspase	3	3	3	6	0
ASJ::caspase	3	3	3	7	1
ASJ::caspase	3	3	3	8	0
ASJ::caspase	3	3	3	9	0
ASJ::caspase	3	3	3	10	0
ASJ::caspase	3	3	3	11	0
ASJ::caspase	3	3	3	12	0
ASJ::caspase	3	3	3	13	0
ASJ::caspase	3	3	3	14	0
ASJ::caspase	3	3	3	15	0
ASJ::caspase	3	3	3	16	0
ASJ::caspase	3	3	4	1	0
ASJ::caspase	3	3	4	2	0
ASJ::caspase	3	3	4	3	0
ASJ::caspase	3	3	4	4	0
ASJ::caspase	3	3	4	5	0
ASJ::caspase	3	3	4	6	0
ASJ::caspase	3	3	4	7	0
ASJ::caspase	3	3	4	8	0
ASJ::caspase	3	3	4	9	0
ASJ::caspase	3	3	4	10	0
ASJ::caspase	3	3	4	11	1
ASJ::caspase	3	3	4	12	0
ASJ::caspase	3	3	4	13	0
ASJ::caspase	3	3	4	14	0
ASJ::caspase	3	3	4	15	0
