# Supplementary material for: C. elegans Demonstrates Distinct Behaviors within a Fixed and Uniform Electric Field
Source: PLoS One. 2016 Mar 21;11(3):e0151320. doi: 10.1371/journal.pone.0151320 (PMC4801214; doi:10.1371/journal.pone.0151320)
Supplement: S3 Dataset — (PDF) [file pone.0151320.s003.pdf]

Measurements for Field Strength versus Angle at Different Applied Field Strengths

V/cm

1.5

	1	2	3	4	5	6	7	8	9	10	11	12	13
A	NA	NA	NA	NA	2.41	2.98	3.76	4.25	5.06	NA	NA	NA	NA
B	NA	NA	NA	NA	2.27	2.97	3.62	4.34	5.05	5.94	6.39	NA	NA
C	NA	NA	Na	NA	1.9	2.66	3.49	4.44	5.28	6.05	6.75	NA	NA
D	GRD	NA	NA	NA	2.13	2.84	3.46	4.25	5.07	5.87	6.79	7.38	NA
E	NA	NA	NA	NA	2.08	2.76	3.45	4.33	5.16	6.27	6.84	7.68	8.28
F	NA	NA	NA	NA	2.02	2.76	3.67	4.3	5.11	5.92	6.78	7.53	8.41
G	NA	NA	NA	NA	2.14	2.65	3.52	4.35	5.05	6.07	6.85	7.68	8.54
H	-0.24	0.13	0.76	1.37	2.09	2.83	3.75	4.37	5.12	6.31	6.8	7.62	8.5
I	-0.16	0.23	0.8	1.48	2.1	2.65	3.64	4.38	5.2	6.04	6.88	7.92	8.29
J	-0.11	0.26	0.81	1.4	1.94	2.72	3.55	4.32	5.19	6.22	6.81	7.6	NA
K	0.12	0.53	0.92	1.57	2	2.94	3.51	4.52	5.05	6.11	6.85	7.55	NA

3

	1	2	3	4	5	6	7	8	9	10	11	12	13
A	NA	NA	NA	NA	4.39	5.32	6.09	7.17	8.38	NA	NA	NA	NA
B	NA	NA	NA	NA	4.12	5.29	5.97	7.21	8.34	9.48	10.25	NA	NA
C	NA	NA	NA	NA	3.85	4.82	5.75	7.13	8.39	9.38	10.96	NA	NA
D	GRD	NA	NA	NA	3.75	4.58	5.72	6.88	8.21	9.5	10.97	12.42	NA
E	NA	NA	NA	NA	3.56	4.62	5.45	6.7	8.13	9.46	11.1	12.65	13.66
F	NA	NA	NA	NA	3.62	4.23	5.32	6.4	7.82	9.08	10.88	12.74	13.74
G	NA	NA	NA	NA	3.56	4.18	5.28	6.41	7.83	9.47	10.63	12.82	13.87
H	-0.4	0.32	1.38	2.38	3.45	4.36	5.56	6.74	7.87	9.26	10.68	12.48	13.86
I	-0.21	0.53	1.37	2.49	3.4	4.39	5.65	6.7	7.88	9.28	10.62	12.14	13.49
J	0	0.6	1.45	2.42	3.44	4.54	5.79	6.88	8.19	9.13	10.38	11.89	NA
K	0.3	1.13	1.7	2.61	3.63	4.43	5.64	6.93	8.01	9.62	10.8	11.82	NA

6

	1	2	3	4	5	6	7	8	9	10	11	12	13
A	NA	NA	NA	NA	6.88	8.89	10.88	12.88	15.23	NA	NA	NA	NA
B	NA	NA	NA	NA	6.63	8.9	10.65	13.16	15.27	17.05	19.19	NA	NA
C	NA	NA	NA	NA	6.46	8.8	10.38	12.95	15.11	17.08	19.46	NA	NA
D	GRD	NA	NA	NA	6.33	8.55	10.35	13.16	15.23	17.43	19.45	21.4	NA
E	NA	NA	NA	NA	6.34	8.69	10.28	12.78	15.37	17.39	19.44	21.2	23.3
F	NA	NA	NA	NA	6.25	8.4	10.15	12.58	15.12	17.51	19.52	21.4	23.4
G	NA	NA	NA	NA	6.75	8.57	10.27	12.68	14.96	17.57	19.53	21.4	23.5
H	-0.3	1.07	2.96	4.6	6.5	8.26	10.23	12.72	15.1	17.4	19.14	21.4	23.8
I	0.07	1.41	2.87	4.75	6.38	8.44	10.35	12.28	14.96	17.22	19.19	21.4	23
J	0.42	1.35	2.83	4.43	6.35	8.12	10.4	12.46	14.8	16.87	18.87	21.4	NA
K	0.23	1.98	3.03	4.77	6.75	8.2	10.15	12.47	14.38	17.09	18.98	21.5	NA

9

	1	2	3	4	5	6	7	8	9	10	11	12	13
A	NA	NA	NA	NA	10.15	12.67	15.4	17.98	21.6	NA	NA	NA	NA
B	NA	NA	NA	NA	9.8	12.68	15.29	18.21	21.7	24.5	28.6	NA	NA
C	NA	NA	NA	NA	9.47	12.65	15.08	18.28	21.1	24.4	28.9	NA	NA

D	GRD	NA	NA	NA	9.25	12.18	15.3	18.5	21.5	25.2	28.9	32.1	NA
E	NA	NA	NA	NA	9.39	12.56	15.23	18.25	21.6	25.2	29.5	33.1	36
F	NA	NA	NA	NA	9.09	12.29	15.27	18.04	21.8	25.6	29.3	32.5	36.2
G	NA	NA	NA	NA	9.08	12.52	15.62	18.55	21.6	25.7	28.7	32.9	36.8
H	-0.85	0.73	2.87	5.98	8.78	12.48	15.6	18.32	22	24.9	28.6	32	37.3
I	-0.39	1.2	2.84	6.45	8.78	12.6	15.58	17.95	22.2	25	28.4	32.1	36.1
J	0.06	1.24	2.98	6.23	8.96	12.57	15.59	18.25	22	25.2	28.9	31.7	NA
K	0.4	1.96	3.28	6.6	9.57	12.4	14.98	18.33	21.2	25.2	28.2	31.6	NA
