# Supplementary figures and images for: C. elegans Demonstrates Distinct Behaviors within a Fixed and Uniform Electric Field
Source: PLoS One. 2016 Mar 21;11(3):e0151320. doi: 10.1371/journal.pone.0151320 (PMC4801214; doi:10.1371/journal.pone.0151320)

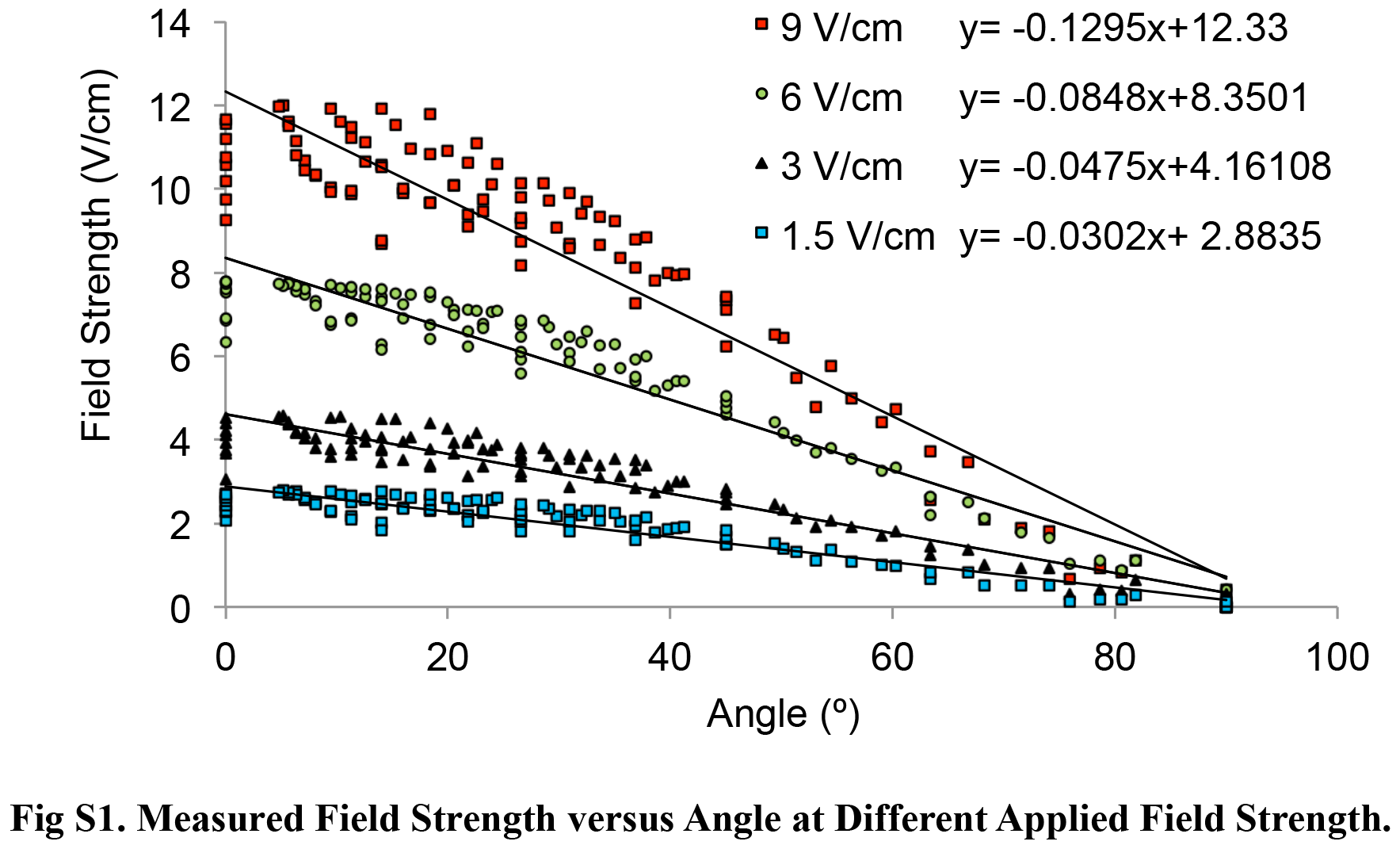

Supplement: S1 Fig — Voltages were measured at 2.5 mm increments across an agar disc using a grid that contained copper pins at multiple angles and distances from a reference point. The field strength was determined by the voltage and distance for each angle measured. Only distances ≥ 1 cm between measurement points for each angle were plotted. These data points were used to calculate measured field strength at the different applied field strengths of 1.5, 3, 6 and 9 V/cm. The measured field strength versus angle was plotted in a scatter graph in order to characterize their linear relationship. The slope formula was used to determine the specific field strength for the mean animal trajectories at voltages 6 V/cm and 9 V/cm, which corresponded to 4.95 and 5.08, respectively. (TIF) [file pone.0151320.s004.tif]

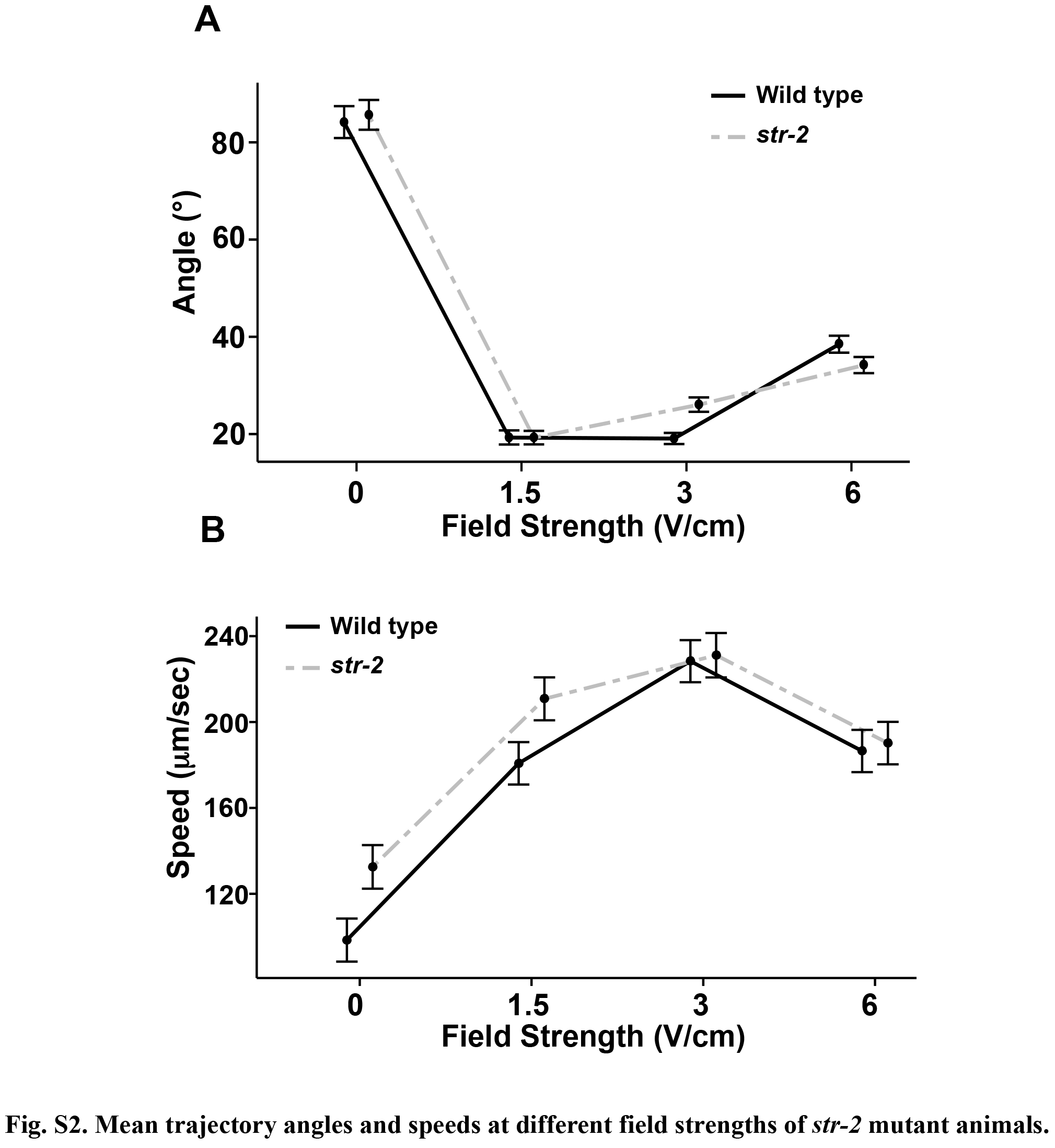

Supplement: S2 Fig — (A) Mean trajectory angles of str-2 mutant animals. (B) Mean speed of str-2 mutant animals. Error bars represent SEM; number of individual animal tracks per genotype (N) ≥ 154. (TIF) [file pone.0151320.s005.tif]
